# Minimal Peroxide Exposure of Neuronal Cells Induces Multifaceted Adaptive Responses

**DOI:** 10.1371/journal.pone.0014352

**Published:** 2010-12-17

**Authors:** Wayne Chadwick, Yu Zhou, Sung-Soo Park, Liyun Wang, Nicholas Mitchell, Matthew D. Stone, Kevin G. Becker, Bronwen Martin, Stuart Maudsley

**Affiliations:** 1 Receptor Pharmacology Unit, National Institute on Aging, National Institutes of Health, Baltimore, Maryland, United States of America; 2 Department of Biology, Saint Bonaventure University, Saint Bonaventure, New York, United States of America; 3 Gene Expression and Genomics Unit, Research Resources Branch, National Institute on Aging, National Institutes of Health, Baltimore, Maryland, United States of America; 4 Metabolism Unit, National Institute on Aging, National Institutes of Health, Baltimore, Maryland, United States of America; Cardiff University, United Kingdom

## Abstract

Oxidative exposure of cells occurs naturally and may be associated with cellular damage and dysfunction. Protracted low level oxidative exposure can induce accumulated cell disruption, affecting multiple cellular functions. Accumulated oxidative exposure has also been proposed as one of the potential hallmarks of the physiological/pathophysiological aging process. We investigated the multifactorial effects of long-term minimal peroxide exposure upon SH-SY5Y neural cells to understand how they respond to the continued presence of oxidative stressors. We show that minimal protracted oxidative stresses induce complex molecular and physiological alterations in cell functionality. Upon chronic exposure to minimal doses of hydrogen peroxide, SH-SY5Y cells displayed a multifactorial response to the stressor. To fully appreciate the peroxide-mediated cellular effects, we assessed these adaptive effects at the genomic, proteomic and cellular signal processing level. Combined analyses of these multiple levels of investigation revealed a complex cellular adaptive response to the protracted peroxide exposure. This adaptive response involved changes in cytoskeletal structure, energy metabolic shifts towards glycolysis and selective alterations in transmembrane receptor activity. Our analyses of the global responses to chronic stressor exposure, at multiple biological levels, revealed a viable neural phenotype in-part reminiscent of aged or damaged neural tissue. Our paradigm indicates how cellular physiology can subtly change in different contexts and potentially aid the appreciation of stress response adaptations.

## Introduction

Cellular adaptations to environmental changes are likely to be highly complex and involve many of the basic cellular functions. It is crucial for cellular/organismal homeostasis during lifespan that molecular systems can adapt and retain functionality despite long-term variation of environment. Aging is a complex multifactorial process, unique in its exact etiology to each individual. There are however several key factors common amongst current hypotheses of aging, one of them being accumulated oxidative stresses. The Harman free radical/oxidative stress theory of aging underpins one of the most popular concepts regarding the biochemical/molecular factors in aging [1]. Harman proposed that physiological iron and other metals would cause reactive oxygen species (ROS) to form in cells as a by-product of normal redox reactions. ROS are a by-product of a variety of pathways in aerobic metabolism. The mitochondrial electron transport chain accounts for the majority of the total oxygen metabolized by the cell, and the by-products produced by the electron transport chain (*e.g*., superoxide anion radicals, hydrogen peroxide, and hydroxyl radicals) are potential sources of oxidative damage to the mitochondrion itself and other cellular compartments. Endogenous ROS-scavenging pathways represent an antioxidant defense system, including both small molecules (tocopherols, vitamin C, glutathione, *etc.*) and antioxidant enzymes (the superoxide dismutases (SOD), the glutathione peroxidases, catalase). The balance between these pathways determines the absolute level of oxidative stress. In aging, complex accumulated systemic imbalances may result in the generation of excess free radicals that overwhelm cellular antioxidant defenses, thereby causing oxidative stress [2]. Aging has also been associated with both a disruption of mitochondrial function [3] along with the steady increase in ROS species, a state that may seem paradoxical as the mitochondria may be the prime source of the ROS. Therefore, it is likely that there are complex interactions between the ROS generating and buffering systems in the aging process. Studies have shown an age-related increase in oxidative damage to a variety of molecules, lipid, protein or DNA, in multiple organisms [4]–[6]. Age-dependent oxidative damage has been implicated in the pathology of age-related disorders in multiple organ systems, *e.g.* sporadic and familial Alzheimer's disease, Huntington's and Parkinson's disease, amyotrophic lateral sclerosis, cardiovascular disease, Type II diabetes and cancer [7]–[12]. Experimental excessive ROS stress can trigger cellular senescence in multiple human cell lines [13], [14]. After exposure to high concentrations of hydrogen peroxide (0.2–1 M) human cells undergo premature senescence, demonstrate lack of response to mitogenic stimuli and show significant changes in gene expression [15], [16]. Metabolic inhibitors, *e.g.* oligomycin or antimycin A, also induce ROS production and induce cellular senescence, demonstrating that defective mitochondria are involved in oxidative cellular senescence [17]. High concentration (0.25 M), acute (90 minute) peroxide exposure has also been shown to switch energy generation in human cells from aerobic metabolism to glycolysis. This functional energetic shift appears to be an important hallmark of aged tissues in numerous species, as proposed by the epigenetic oxidative redox shift theory of aging [18]–[21]. The disruption of energy regulation therefore may be a hallmark of aging and neurodegeneration [22]–[24] however, the specific molecular connections between these two events still remain to be comprehensively identified. From a therapeutic point of view, interventions ameliorating aging/neurodegeneration-related pathologies have therefore been targeted to modulating anti-oxidant mechanisms as well as inflammatory processes, DNA repair mechanisms and modulation of neurotrophic receptor systems [25]–[28]. Disruption of the neurotrophin brain-derived neurotrophic factor (BDNF) activity has been associated with aging and multiple neurodegenerative diseases that demonstrate oxidative pathological aspects [29]–[35]. It has also been shown that many other profound deficits in other receptor systems, *e.g.* cholinergic, serotoninergic, dopaminergic, histaminic, are also implicated in aging and neurodegeneration processes [36]–[39].

Cell death and atrophy have been strongly associated with the aging process and neurological disorders, however in some cases cognitive impairment and aging may occur without this pronounced tissue pathology. Therefore in these cases one could hypothesize that the exposure of cells to non-lethal oxidative stresses for a considerable period of time may be associated with aging [40]. This facet of oxidative stress-induced pathophysiology is the crux of this study. We have employed minimal levels of oxidative stress to mimic the low level of consistent stress potentially experienced by cells aging normally or in the early stages of neurodegenerative disorders. Our decision to focus on the most minimal levels is aimed at trying to improve our knowledge of the earliest effects of oxidative insults. We feel that it is important to fully comprehend the cellular adaptive responses during the early effects of oxidative insults, as it is likely that therapeutics will be most efficacious at this time (*i.e*. before widespread neuronal cell death). We show that neural cells respond strongly to even minimal oxidative stresses in a manner that affects energy metabolism, calcium regulation, tyrosine kinase activity and receptor-mediated responses and protein expression that in-part mimics that of aged/damaged neuronal tissue. Therefore, this model may be a useful tool for investigating the development of novel pharmacotherapeutics specifically targeted to cells experiencing a non-lethal oxidative stress environment.

## Results

### Ultrastructural, mitochondrial and calcium homeostatic alterations induced by chronic minimal peroxide (CMP)

**Figure 1:**
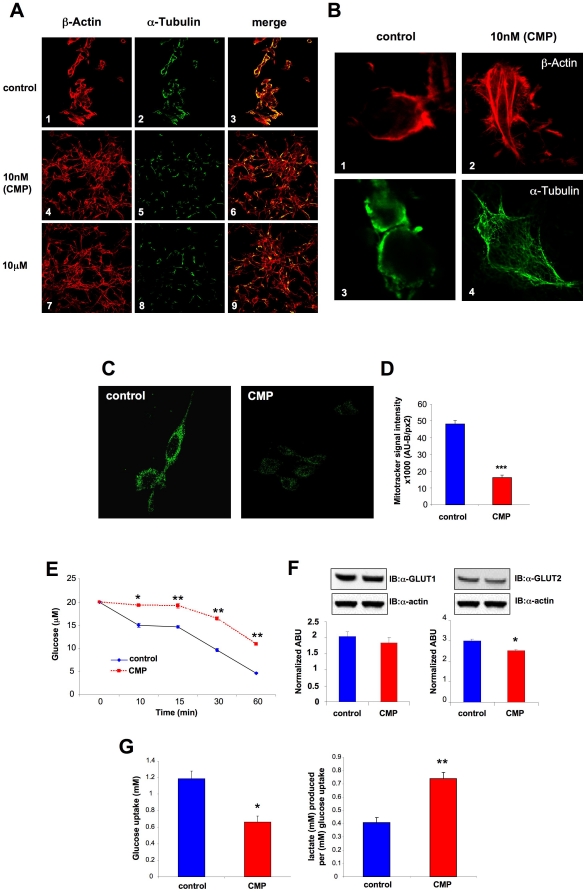
Peroxide treatment of SH-SY5Y cells affects cellular architecture, mitochondrial expression and glucose uptake. **A** SH-SY5Y cells treated with various concentrations of hydrogen peroxide for seven days or vehicle control (*control* – 1–3; 10 nM chronic minimal peroxide ‘CMP’ – 4–6; 10 µM – 7–9) were subsequently stained with Alexafluor-568-phalloidin, for β-actin visualization and counterstained with anti-α-tubulin (middle column). A merge of the fluorescent signals created is also represented. **B** High resolution depiction of effects upon cytoskeletal (1, 2) and microtubular (3, 4) architecture of CMP (10 nM hydrogen peroxide, 7 days) or vehicle (control) treatment. **C** CMP- (10 nM hydrogen peroxide, 7 days) or vehicle-treated (control) SH-SY5Y cells stained with Mitotracker (10 nM). **D** Quantitated Mitotracker fluorescence from control or CMP-treated cells. Results represent mean ± standard error mean (SEM) of Mitotracker fluorescence from 10 cells/microscopic fields (n = 3 fields). Mitotracker signal intensity ((AU-B)/px^2^) is quantified as arbitrary units (AU) – background (B) per square visual pixel (px^2^). **E** Glucose-deprived SH-SY5Y cells treated with either vehicle control (control) or CMP were assessed for bolus (20 µM) glucose uptake (n = 3). Glucose concentration of the extracellular media was assessed at the time points indicated after bolus application. **F** CMP-mediated alteration of GLUT1 or GLUT2 expression. GLUT expression measured as normalized arbitrary absorbance units (ABU). **G** CMP-mediated reduction of cellular glucose uptake after 4 hours of bolus glucose (5 mM: left panel) and lactate production was measured and normalized to the glucose uptake over a 4 hour period (right panel). Statistical significance was measured using a Student's t-test (GraphPad Prism v.3): * - p<0.05; ** - p<0.01; *** -p<0.001. This notation is used consistently throughout.

Application of various doses of H_2_O_2_ (10 nM or 10 µM) for seven days to SH-SY5Y cells resulted in a strong re-organization of both actin and tubulin into highly ordered superstructures (Figure 1A). Similar structural changes, compared to control cells, were seen for 10 nM (Figure 1A, 4, 5, 6) and 10 µM H_2_O_2_ (Figure 1A: panels 7, 8, 9) indicating that qualitatively similar effects were seen with the chronic minimal peroxide (CMP: 10 nM H_2_O_2_, seven day exposure) and higher peroxide doses. Under higher resolution, CMP treatment, compared to control, induced the formation of classical actin stress fibres (Figure 1B, 1–2) polymerized microtubule structures (Figure 1B, 3–4). Along with cytoskeletal alterations we also assessed the effects of the CMP paradigm upon another primary cellular compartment often linked with oxidative stress, *i.e.* mitochondria. Using Mitotracker (10 nM) to identify mitochondrial expression we noted a significant reduction of Mitotracker-fluorescence (Figure 1B,C) in cells exposed to the CMP paradigm. The extant mitochondria in CMP-treated cells appeared functional when assessed using tetramethylrhodamine ethyl ester-associated fluorescence (data not shown). In addition to this alteration of energy-controlling organelles we also investigated the ability of CMP cells to uptake and utilize glucose. CMP-treated cells, compared to control, demonstrated a significant reduction of glucose uptake (Figure 1D) as well as reductions of membrane GLUT 1 and 2 transporter expression (Figure 1E). In addition, we determined the metabolic fate of glucose in CMP cells and found that CMP treatment induced a proportional increase in lactate production for a given level of glucose uptake after introduction of a 5 mM bolus dose (4 hours) (Figure 1F).

### CMP-induced alterations of calcium homeostasis

**Figure 2:**
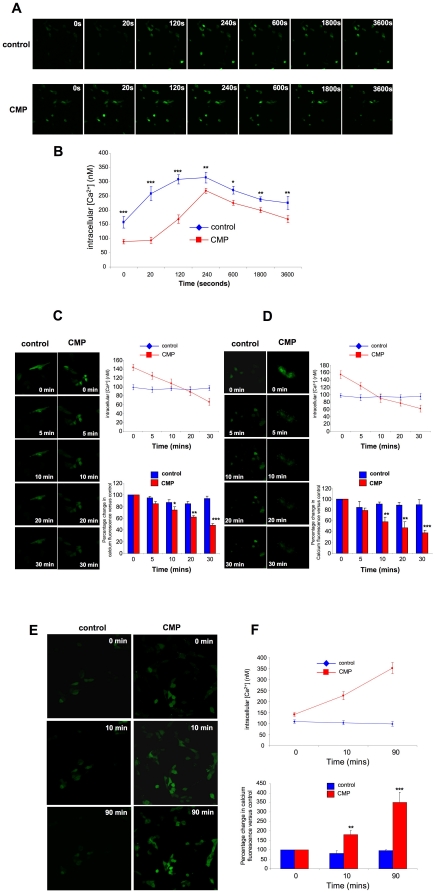
Chronic minimal peroxide treatment modification of calcium homeostasis. **A** Basal and glutamate (50 µM)-stimulated increase of intracellular Fluo-4 fluorescence intensity in control or CMP cells. **B** Graphical representation of calculated intracellular calcium (nM) changes following 50 µM glutamate exposure (60 minutes) for CMP or control SH-SY5Y cells. Mean ± SEM fluorescent calcium measurements were taken from 10 cells/microscopic field (n = 3 fields). **C** Changes in intracellular calcium Fluo-4 fluorescent intensity resulting from 10 µM nifedipine exposure (30 minutes) for CMP or control SH-SY5Y cells. The associated histograms depict the resultant changes of calcium levels and intracellular Fluo-4 intensity (relative to time = 0 minutes), over time in response to nifedipine treatment. Fluo-4 intensity and calcium measurements for each time point were the average from 10 cells/microscopic field (n = 3 fields). **D** Changes in intracellular Fluo-4 fluorescent intensity resulting from 1 µM ω-conotoxin exposure (30 minutes) for CMP or control SH-SY5Y cells. The associated histograms depict the resultant changes of calcium levels and intracellular Fluo-4 intensity (relative to time = 0 minutes), over time in response to nifedipine treatment. Fluo-4 intensity and calcium measurements for each time point were the average from 10 cells/microscopic field (n = 3 fields). **E** Confocal Fluo-4 intensity images from control-treated SH-SY5Y cells or CMP-treated SH-SY5Y cells at various time points before (t = 0) and after (t = 10, 90 minutes) exposure to the SERCA channel blocker, CPA (100 µM). Panel **F**, intracellular calcium concentration and percentage changes in calcium Fluo-4 intensity versus their individual controls (t = 0) for control or CMP-treated cells. Values depicted represent the mean ± SEM, measured as mean from 10 cells/microscopic field: n = 3 fields.

Using the membrane-permeant calcium sensitive dye, Fluo-4AM, a significantly elevated resting calcium level was identified in CMP-treated SH-SY5Y cells (Figure 2A). CMP cells were still able to respond to excitatory glutamate however. In both CMP and control cells glutamate stimulation resulted in long-lasting increases in Fluo-4AM signal intensity (Figure 2B) with the maximal Fluo-4AM signals occurring more rapidly in CMP cells compared to control. We investigated two ion channel mechanisms that could contribute to the high resting calcium level in the CMP cells, *i.e.* the plasma membrane voltage-gated calcium channels (VGCC) and the sarco/endoplasmic reticulum calcium channels (SERCA). VGCCs facilitate increases of cytoplasmic calcium via influx from the extracellular space, while SERCA channels actively modulate resting cytoplasmic calcium levels by removal of cytoplasmic calcium to endoplasmic reticulum stores. Selective chemical blockade of the L- and N-type VGCCs, with nifedipine and ω-conotoxin GVIA respectively, was used to investigate the role of VGCCs in the elevated Fluo-4AM intensity in CMP cells. After steady-state Fluo-4AM loading, L- or N-type VGCC blockade, with nifedipine (10 µM: Figure 2C) or conotoxin GVIA (Figure 2D) respectively, induced a progressive reduction (5–20 minutes) of Fluo-4AM fluorescence in CMP compared to control cells (Figure 2C, D). Therefore the high resting Fluo-4AM cytoplasmic intensity in CMP cells is in-part mediated by L and N-type VGCC influx. A chemical inhibitor of the SERCA 1 channel, cyclopiazonic acid (CPA: 100 µM), was used to investigate the role of cytoplasmic clearing in CMP cells. Upon application of CPA to the Fluo-4AM-loaded CMP cells, a strong increase in the cytoplasmic Fluo-4AM fluorescence, compared to control cells, was observed suggesting an elevated calcium transfer process in CMP cells (Figure 2E, F). To investigate the ability of CMP cells to maintain cellular function in the face of changes in calcium flux we assessed basic electrophysiological properties of CMP cells. CMP and control cells were current clamped and subjected to 20 pA steps of current (−20 to +100). Both control and CMP cells exhibited abortive action potentials typical of undifferentiated SH-SY5Y cells (Figure S1A [41]), indicating that CMP treatment had not modified whole-cell excitability. To assess changes in K^+^ channel conductance, cells were voltage clamped at −40 mV and exposed to +10 mV voltage steps (range, −40 mV to +120 mV). Steady state K^+^ channel conductance I/V plots for CMP and control cells (Figure S1B) demonstrated that K^+^ channel conductances were unchanged with CMP treatment. We also investigated whether CMP treatment affected plasma membrane integrity. Classical passive membrane properties of cells: resting membrane potential, input resistance, access resistance and membrane capacitance are useful measures for assessing cell health, membrane integrity, and membrane size. No significant differences in any of these membrane parameters between CMP or control cells was evident, indicating a relatively subtle action of the CMP paradigm (Figure S1C).

### CMP induces differential responsiveness to receptor ligands

**Figure 3:**
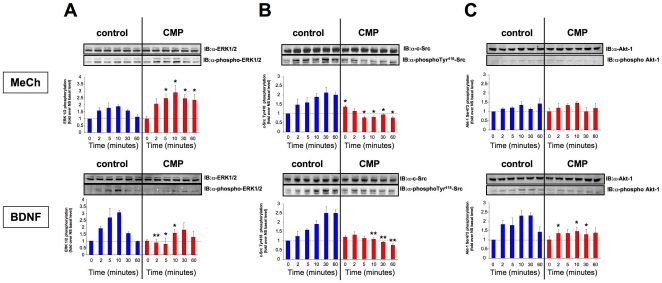
CMP treatment of SH-SY5Y cells affects intermediary cell metabolic responses to stimulatory ligands. Representative western blots and associated histograms depict the changes in ERK1/2 (**A**), c-Src (**B**) and Akt-1 (**C**) activation in response to β-methylcholine (MeCh, 10 nM) or brain-derived neurotrophic factor (BDNF, 10 ng/mL) stimulating ligands in both control (blue bars) or CMP-treated (red bars) SH-SY5Y cells. The time courses (0-60 minutes) for stimulation are denoted in the associated histograms depicting the mean ± SEM from at least three separate experiments. Statistical significance is indicated for changes in kinase activity in the CMP state relative to their time-matched control in vehicle-treated (control) cells. Statistical significance was measured using a Student's t-test (GraphPad Prism v.3): * - p<0.05; ** - p<0.01.

As we have assessed the electrical excitability of the CMP cells, we next investigated the ability of CMP cells to respond to exogenous chemical stimulants. We investigated the activity of multiple transmembrane receptor systems that possess neuromodulatory or neurotrophic actions. Stimulation of control or CMP cells with dopamine (DA), β-methylcholine (MeCh), histamine (HA), lysophosphatidic acid (LPA), anandamide (ADA) and brain-derived neurotrophic factor (BDNF) was measured by assessment of ligand-induced extracellular signal-regulated kinase 1/2 (ERK1/2), c-Src or Akt-1 activation. In CMP-treated cells a significant potentiation of ERK1/2 activation induced by MeCh, HA, LPA and ADA (Figure 3A: DA, HA, LPA, ADA-Figure S2) was evident. In contrast to ERK1/2 results, MeCh-mediated c-Src auto-tyrosine-418 phosphorylation was diminished in CMP cells compared to control (Figure 3B: DA, HA, LPA, ADA-Figure S2). BDNF-induced ERK1/2 and c-Src activation were attenuated in CMP cells compared to control (Figure 3A,B). BDNF-induced c-Src activation was also diminished in CMP cells versus control (Figure 3B). A prime molecular function of BDNF is the activation of neuroprotective Akt-1. As with many GPCR-based ligands little basal Akt-1 activation was noted with MeCh (Figure 3C). BDNF-induced Akt-1 activation in CMP cells was severely attenuated compared to control cells (Figure 3C). The differential activity of the CMP paradigm between the GPCR ligand activity and BDNF presented an interesting divergence in action of CMP.

### CMP-mediated transcriptome alterations in SH-SY5Y cells

The effects of the CMP protocol upon basal gene transcription was investigated (Table S1). Inspection of the basal transcriptome differences between CMP and control cells revealed the significant regulation of multiple genes implicated in neurodegenerative processes (*SPAST*, *SACS*, *FKTN*, *DOPEY2*, *ALS2CR14*, *RTN3*, *BLZF1*, *SFRS11*, *AHR*, *ARPP-19*, *B2M*), control of cytoskeletal dynamics (*PTMA*, *DAPP1*, *HMMR*, *CAPZA1*) and age-related energetic/metabolic changes (*TSC1*, *LARS*, *MSTO1*, *LEP*, *SBDS*). Using unbiased functional Gene Ontology/KEGG pathway gene clustering we revealed strong population of GO-term groups involved in protein synthesis (*cysteinyl-tRNA aminoacylation*), energy regulation (*glycoprotein catabolism*), nucleo-cellular transport processes (*regulation of NF-κB import into nucleus*) and neuronal differentiation (*Notch signaling pathway*) (Table 1). KEGG pathway analysis of CMP-regulated genes revealed significant population of functional signaling pathways including: protein synthesis (*Ribosome*); neurophysiology (*Alzheimer*'*s disease*, *neurodegenerative disorders*, *long-term depression*); energy regulation (*urea cycle/metabolism of amino acids*); metabolic signaling (*insulin/mTOR signaling pathways*) and pathways linked to cellular architecture (*focal adhesion*) (Table 2). Using MeSH-database analysis of the gene-disorder association strengths of the CMP-controlled geneset we found that the geneset is likely to be linked to energy regulatory (*Type II diabetes*), neurodegenerative (*dementia*, *Parkinson*'*s disease*, *mental retardation*) and cardiovascular disorders (*coronary artery disease*, *adult respiratory distress syndrome*) typically found in subjects experiencing long-term stress effects (Table 3). We also used a new bioinformatic approach (latent semantic indexing, see *Materials and Methods*) to illustrate the functional connectivity between multiple CMP-regulated genes. We constructed an interrogation-term matrix for the significantly regulated CMP geneset (Table S1). This matrix investigated links between potentially CMP-related functions (‘*energy regulation’*, ‘*Ca^2+^ regulation’*, ‘*glucose metabolism*’, ‘*mitochondria*’, ‘*aging*’, ‘*oxidation*’ ‘*neurodegeneration*’ and ‘*stress*’) and the transcriptomic response (Figure S3: Table S1). Two genes were linked to seven out of eight terms (*DNM1L*, *ENDOG*) and five genes were linked to six terms (*MINPP1*, *TSPO*, *SPTLC1*, *NNT*, *IHPK1*). Interestingly all of these have been implicated in long-term stress responses, aging and age-related neurodegenerative disorders.

**Table 1:** Gene Ontology (biological process) analysis of CMP-mediated geneset alteration compared to control SH-SY5Y cell geneset.

GO-*bp*	Enrichment R (O/E)	Probability (p)	Hybrid
***Protein synthesis***			
cysteinyl-tRNA aminoacylation	50	4.79E-04	165.9832
snRNA processing	8.33	2.30E-02	13.64681
translation	3.44	1.09E-04	13.63125
mRNA catabolism	6	1.32E-02	11.27656
transcription from RNA polymerase III promoter	5.45	1.66E-02	9.700411
nuclear mRNA splicing\, via spliceosome	3.23	1.11E-03	9.543607
translational initiation	3.94	8.57E-03	8.144056
tRNA aminoacylation for protein translation	4.26	1.41E-02	7.884327
rRNA processing	3.09	2.28E-02	5.073981
***Cell cycle control***			
G2 phase of mitotic cell cycle	22.22	2.77E-03	56.8281
mitotic spindle checkpoint	15.38	6.74E-03	33.39521
interphase of mitotic cell cycle	3.82	9.86E-03	7.66339
interphase	3.62	1.20E-02	6.953364
negative regulation of progression through cell cycle	2.44	1.20E-02	4.686798
mitosis	2.33	2.20E-02	3.862155
M phase	2.05	3.26E-02	3.047904
***Energy regulation***			
glycoprotein catabolism	12.5	1.50E-03	35.29886
aerobic respiration	4.29	3.21E-02	6.407093
energy reserve metabolism	3.8	4.34E-02	5.177539
***Cellular transport***			
axon cargo transport	13.33	9.29E-03	27.08635
proline biosynthesis	13.33	9.29E-03	27.08635
regulation of NF-kappaB import into nucleus	8.33	2.30E-02	13.64681
regulation of transcription factor import into nucleus	5.71	4.67E-02	7.598201
protein import into nucleus	3.7	1.13E-02	7.20361
***Neuronal differentiation***			
positive regulation of cell differentiation	9.68	3.14E-03	24.22972
regulation of neuron differentiation	9.09	1.91E-02	15.62541
negative regulation of cytokine biosynthesis	9.09	1.91E-02	15.62541
regulation of neurogenesis	6.06	4.14E-02	8.380978
Notch signaling pathway	4.05	3.75E-02	5.775173
cell motility	2.36	5.21E-03	5.388263
microtubule-based process	2.4	1.92E-02	4.120077
generation of neurons	2.66	4.00E-02	3.71852
***DNA metabolism***			
base-excision repair	6	1.32E-02	11.27656
DNA recombination	3.95	1.93E-03	10.72205
pyrimidine nucleotide metabolism	4.41	2.96E-02	6.741604
nucleosome assembly	3.13	1.25E-02	5.956672
DNA replication	2.11	3.73E-02	3.013694

**Table 2:** KEGG pathway analysis of CMP-mediated geneset alteration compared to control SH-SY5Y cell geneset.

KEGG Pathway	Enrichment R (O/E)	Probability (p)	Hybrid
***Protein synthesis***			
Ribosome	8.6059	2.70E-07	56.52903
Aminoacyl-tRNA biosynthesis	7.9957	6.15E-03	17.67949
Folate biosynthesis	6.3546	1.17E-02	12.27591
Ubiquitin mediated proteolysis	5.7637	1.52E-02	10.47931
***Neurophysiology***			
Alzheimer's disease	11.8017	1.99E-03	31.87813
Dorso-ventral axis formation	9.5329	3.72E-03	23.15977
Neurodegenerative Disorders	7.2904	7.98E-03	15.29524
Long-term depression	4.3483	1.37E-02	8.102098
Gap junction	3.888	1.99E-02	6.614059
***Cellular metabolism***			
Sulfur metabolism	12.7065	1.04E-02	25.19657
Urea cycle and metabolism of amino groups	6.8847	3.38E-02	10.12797
Inositol phosphate metabolism	5.3879	1.82E-02	9.374561
Arginine and proline metabolism	4.6765	2.64E-02	7.381369
Pyrimidine metabolism	3.713	2.31E-02	6.075909
Purine metabolism	2.8293	3.28E-02	4.199038
***Cellular signaling***			
Insulin signaling pathway	4.4487	1.07E-03	13.21538
mTOR signaling pathway	5.2733	1.93E-02	9.040771
Fc epsilon RI signaling pathway	4.4658	1.25E-02	8.498819
Hedgehog signaling pathway	4.59	2.77E-02	7.149018
***Cellular architecture***			
Cell cycle	4.4659	2.36E-03	11.73231
Focal adhesion	3.4786	2.31E-03	9.170939
Cell Communication	3.9342	9.12E-03	8.025788
Apoptosis	4.08	1.69E-02	7.230222
ECM-receptor interaction	4.0302	1.77E-02	7.061018
Regulation of actin cytoskeleton	2.0969	9.21E-02	2.171844

**Table 3:** Medical subject heading term analysis of CMP-mediated geneset alteration compared to control SH-SY5Y cells geneset.

MeSH Disease Terms	Z Score
***Musculoskeletal***	
ARTHRITIS__JUVENILE_RHEUMATOID	4.300434
OSTEOARTHRITIS	0.461885
***Diabetes – Energy regulatory***	
DIABETES_MELLITUS__TYPE_2	2.002813
DIABETIC_NEPHROPATHIES	1.562933
HYPERINSULINISM	1.006024
HYPERTRIGLYCERIDEMIA	0.79994
***Neurodegeneration***	
DEMENTIA	1.969758
CEREBROVASCULAR_ACCIDENT	1.812081
ATROPHY	1.423247
PARKINSON_DISEASE	1.275841
BRAIN_ISCHEMIA	1.071663
CEREBELLAR_ATAXIA	0.746189
CHARCOT-MARIE-TOOTH_DISEASE	0.714724
MENTAL_RETARDATION	0.414224
***DNA instability***	
CHROMOSOME_ABERRATIONS	1.870107
***Cardiovascular disorders***	
CORONARY_ARTERY_DISEASE	1.845112
MYOCARDIAL_INFARCTION	1.434788
CAROTID_ARTERY_DISEASES	1.33356
RESPIRATORY_DISTRESS_SYNDROME__ADULT	1.211369
VASCULAR_DISEASES	1.035383
***Protein metabolism disorders***	
PROTEINURIA	1.843715
ALBUMINURIA	1.579696
***Neoplastic disorders***	
SKIN_NEOPLASMS	1.3896
PHARYNGEAL_NEOPLASMS	1.318447
METAPLASIA	1.08451
LYMPHOMA	0.903678
ADENOMATOUS_POLYPOSIS_COLI	0.893777
***Renal disorders***	
HEPATITIS__TOXIC	1.048206

Cumulative Z scores are derived from the positive associations of significantly regulated genes that populate the denoted MeSH groups obtained from the National Library of Medicine (http://www.ncbi.nlm.nih.gov/mesh).

**Figure 4:**
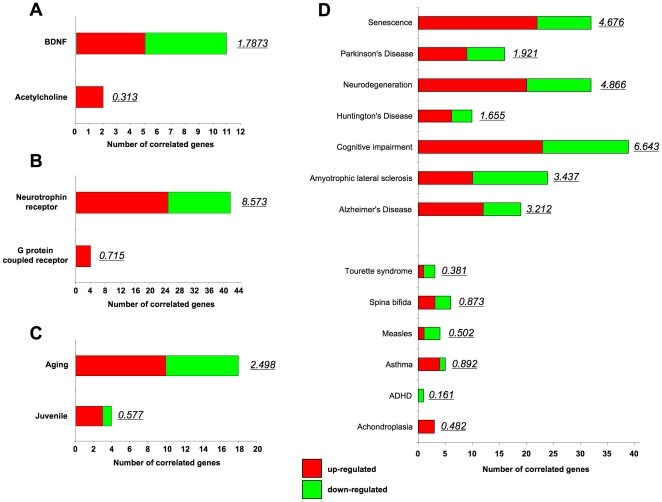
CMP treatment exerts a biased effect upon gene transcription in SH-SY5Y cells. **A–C** The CMP-responsive significantly-regulated geneset was interrogated using latent semantic indexing (GeneIndexer) of input terms. The number of implicitly correlated genes (≥0.1 latent semantic indexing correlation score) are denoted for each interrogation term (red bar section = upregulated genes; green bar section = downregulated genes) as well as the total correlation score for those genes (italic next to colored bar). The specific interrogation term (**A**- BDNF vs. Acetylcholine: **B**- Neurotrophin receptor vs. G protein-coupled receptor: **C**- Aging vs. juvenile) are denoted in each panel **A–C**. **D** Latent semantic indexing scores and number of genes associated with aging/degeneration disease-related interrogation terms (*senescence*, *Parkinson*'*s disease*, *neurodegeneration*, *Huntington*'*s disease*, *Cognitive impairment*, *Amyotrophic lateral sclerosis*, *Alzheimer*'*s disease*) compared to ageing-independent disorders (*Tourette syndrome*, *Spina bifida*, *measles*, *asthma*, *ADHD*, *achondroplasia*). The number of implicitly-associated genes are indicated in the colored horizontal bars as well as the cumulated latent semantic indexing scores (italic).

As we noted a divergent effect of CMP-treatment upon BDNF versus MeCh signaling, we investigated whether the basal CMP-transcriptome effects or early gene responses to these ligands also demonstrated this. Firstly, several important functional genes linked to neurotrophic receptor tyrosine kinase signaling were observed to be downregulated by CMP, *e.g. BEX1*, *TOB1*, *GSK3β*, *GRB2* and *SH3KBP1* (Table S1). We used latent semantic indexing in an ‘oppositional’ experimental approach (GeneIndexer-*Materials and Methods*) to identify trends in the CMP-controlled geneset that may underpin the potential bias in BDNF versus MeCh signaling and also illustrate the overall phenotype of the CMP-mediated transcriptome. Interrogation of the CMP-induced geneset with the oppositional terms ‘*BDNF*’ or ‘*acetylcholine*’ demonstrated that eleven significantly CMP-regulated genes were implicitly associated with ‘*BDNF’* (cumulative LSI correlation score of 1.787) compared to only 2 genes associated with ‘*acetylcholine*’ (LSI score, 0.313) (Figure 4A; Table S2). Interrogation with the terms ‘*neurotrophin receptor*’ (42 genes, cumulative LSI correlation score of 8.573: Table S2) versus ‘*G protein-coupled receptor*’ (4 genes, cumulative LSI correlation score of 0.715) also demonstrated a bias of the CMP-induced geneset (Figure 4B). As our CMP regimen is designed primarily to mimic long-term oxidative stress responses, that may occur in the aging process, we then used different interrogation terms to investigate these potential effects of our process. Interrogation of the CMP dataset with the terms ‘aging’ versus ‘juvenile’, showed that the significant gene number and LSI score was considerably greater for ‘*aging*’ (18 genes, LSI score 2.498) compared to ‘*juvenile*’ (4 genes, LSI score 0.577) (Figure 4C, Table S2). We also compared the interrogation of the CMP dataset with aging-related neurological disease terms versus terms for diseases not usually linked to the aged. Considerably higher gene representative (number) and LSI correlation scores were observed for terms linked to aging-related disorders and pathophysiology (*e.*g. Alzheimer's disease) compared to terms linked to disorders that tend to be prevalent in the young or are congenital (*e.g.* ‘*attention-deficit deficiency disorder’*) (Figure 4D, Table S2).

### CMP treatment affects dynamic genomic responses to ligand stimulants

**Figure 5:**
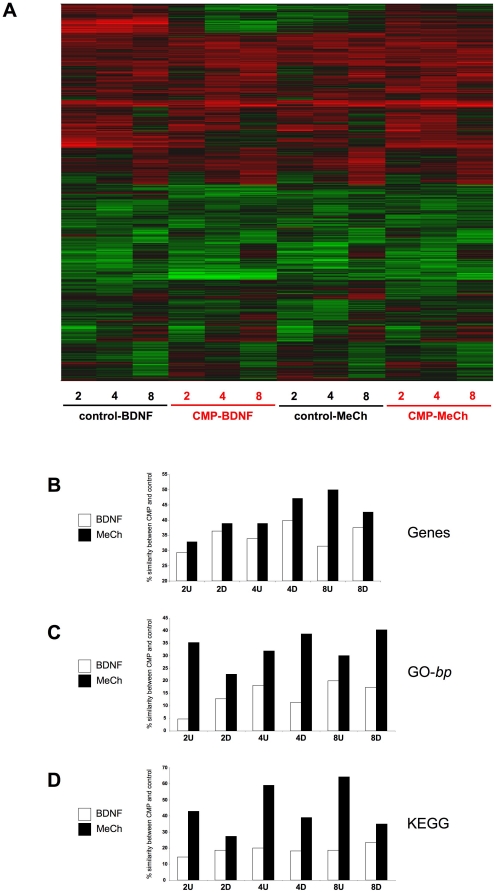
CMP treatment differentially affects early genomic responses to BDNF and MeCh. **A** Heatmap diagram indicating differences in global gene regulation (red-upregulation: green-downregulation) in control (black under bar) and CMP states (red under bar) for 2, 4 and 8 hours of either BDNF or MeCh stimulation. **B** Significantly-regulated gene identity conservation between the CMP and control state for BDNF- (white bars) or MeCh-stimulated (black bars) genesets separated into upregulated (2 hours up-2U; 4 hours up-4U; 8 hours up-8U) or downregulated groups (2 hours down-2D; 4 hours down-4D; 8 hours down-8D). The percentage conservance between CMP and control state of significantly-regulated genes was calculated as the percentage of total genes regulated in the specific CMP conditioned cells (2U, 4U, 8U and 2D, 4D, 8D) that were also significantly regulated in the control state. **E** Significantly-enriched Gene Ontology biological process (GO-bp) term group conservance between CMP and control state SH-SY5Y cells treated with either BDNF or MeCh. Histogram creation and structure is identical to that in panel **D**. **F** Significantly-enriched KEGG pathway conservance between CMP and control state SH-SY5Y cells treated with either BDNF or MeCh. Histogram creation and structure is identical to that in panel **D**.

**Figure 6:**
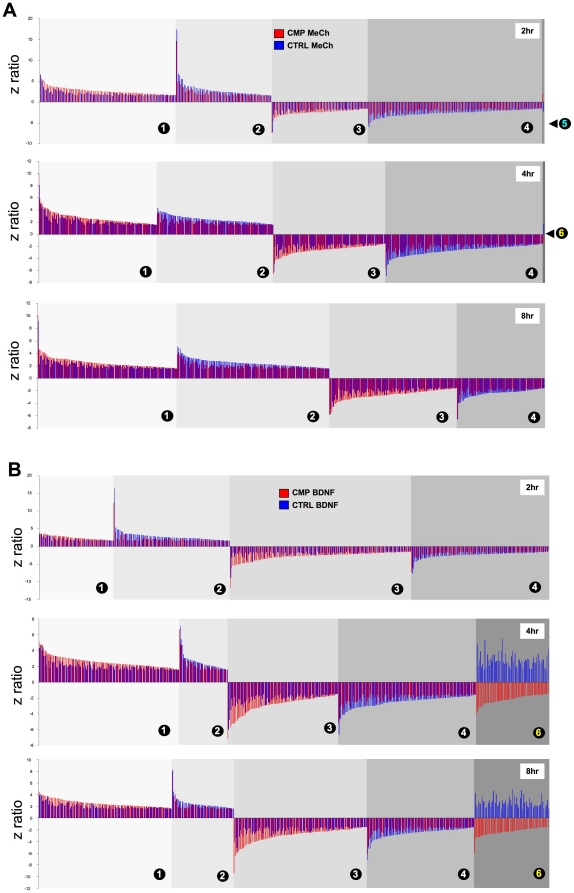
Differential effects of CMP upon BDNF- or MeCh-regulated genes common to control or CMP cellular states. **A** MeCh-significantly regulated (indicated by z ratio) genes common between CMP and control (CTRL)-treated SH-SY5Y cells. The top panel depicts polarity-dependent series (1–5) of MeCh-regulated genes after 2 hours of stimulation, middle panel represents common CMP-control gene series (1–6) after 4 hours of stimulation while the lowest panel represents common CMP-control gene series (1–4) after 8 hours of stimulation. **B** depicts analogous genomic data to panel **A** but for BDNF instead of MeCh. In both **A** and **B** the CMP-(MeCh or BDNF) regulated gene expression levels are denoted in red while the control-(MeCh or BDNF) regulated gene expression levels are denoted in blue. For both BDNF or MeCh the gene regulation series are as follows: series 1 = upregulated in CMP and control with z ratio CMP>CTRL; series 2 = upregulated in CMP/control with z ratio CTRL>CMP; series 3 = downregulated in CMP/control with z ratio CMP<CTRL; series 4 = downregulated in CMP/control with z ratio CTRL<CMP; series 5 = upregulated in CMP-downregulated in CTRL; series 6 = downregulated in CMP-upregulated in control.

In addition to investigating the static genomic effects of the CMP paradigm we investigated how the static changes we observed may affect dynamic cellular responses, or previously identified bias, to activating ligands. Control or CMP-treated cells were treated with MeCh or BDNF for 2, 4 or 8 hrs and the transcriptional effects were subsequently measured (Tables S3, S4, S5, S6, S7, S8 MeCh; Tables S9, S10, S11, S12, S13, S14 BDNF). Organizing genes, significantly regulated (up-red: down-green) by either BDNF or MeCh stimulation in both control or CMP cells, into heatmaps demonstrated several areas of considerable regulatory deviation between these two states for BDNF compared to MeCh treatment (Figure 5A). BDNF- or MeCh-induced genesets were separated into up (U)- or down (D)-regulated groups across the multiple time points (2, 4, 8 hours). To assess the effects of CMP on BDNF or MeCh-induced gene transcription, we calculated the percentage of regulated gene identity retained between the ligand-induced transcriptional effects in control compared to CMP cells (Figure 5B). In addition to analyzing the ligand-mediated gene regulation we also clustered the BDNF and MeCh-regulated genes into significantly-populated GO-term (Figure 5C) and KEGG pathway groups (Figure 5D). Comparing the percentage similarity (between CMP and control conditions) of BDNF and MeCh-mediated gene regulation, GO-term group population and KEGG pathway population, we found that the BDNF-mediated effects were consistently the most affected by CMP treatment (Figure 5B–D). At each time point there was less percentage similarity between BDNF effects in CMP cells compared to control cells. Many of the BDNF/MeCh-regulated genes controlled in both CMP and control states (Figure S4). We therefore assessed if there were any differences in the polarity (up or downregulation) of control for BDNF or MeCh in CMP versus control states. Genes regulated by BDNF or MeCh in both CMP and control states were organized into coherent series (numbered 1–6) based on the polarity (up or down), magnitude of regulation and time scale of regulation (Figure 6A, B: Tables S15, S16, S17 (MeCh) and S18–S20 (BDNF)). For MeCh stimulation, only two genes regulated in both CMP and control conditions demonstrated a reversal of their polarity (Figure 6A). In contrast, for BDNF stimulation, the polarity of regulation was reversed for seventy nine genes between CMP and control states (Figure 6B). Many of these ‘reversed-polarity’ BDNF-controlled genes are involved in protein metabolism/synthesis (*e.g. RPL14*), energy regulation (*LEP*, *DLDH*) the aging process (*HMGB2*, *PTMA*) as well as age-related neurodegeneration (*YY1*, *HIF1A*, *TIA1*, *RTN3*). We also noted that within the specific regulation series (1–4: Figure S5) the modulus of the differences in gene regulation z-ratios (between CMP and control states) was greater for BDNF-controlled compared to MeCh-controlled genes (Figure S5). Therefore, at multiple and detailed levels of investigation the CMP protocol exerts a preferential alteration of neurotrophin receptor activity compared to GPCR activity. The repetition of these subtle effects in multiple biochemical aspects may be indicative of how signaling connectivity and adaptive responses can pervade diverse aspects of cellular function, especially in cases of stressful perturbations.

### Minimal oxidative stress induces signaling network and proteomic alterations in SH-SY5Y cells

**Figure 7:**
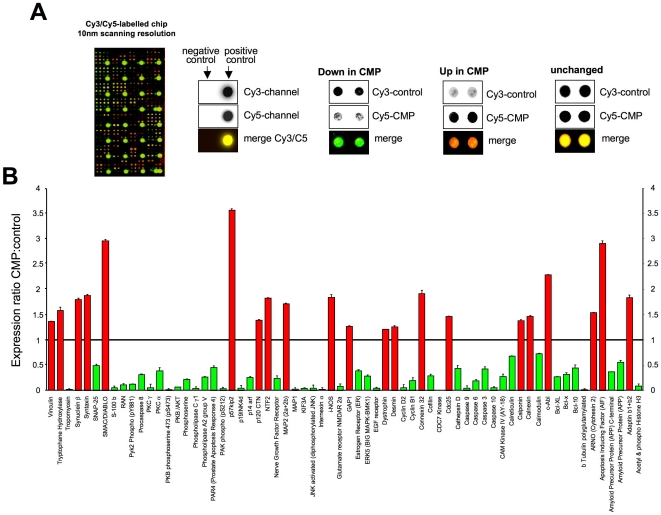
Global cellular signal transduction analysis of neural response to chronic minimal hydrogen peroxide exposure. **A** Representative Cy3/Cy5 protein-labeled cell signaling Panorama® antibody microarray chip. Exemplary insets from experimental antibody chips are highlighted indicating relative changes in Cy3 or Cy5 reactivity for the negative (unlabelled bovine serum albumin deposited on chip) and positive control (Cy3/Cy5-labeled bovine serum albumin deposited on chip) chip area as well as for factors identified that demonstrate increased, decreased or an unchanged expression between control and CMP conditions. Individual channel (Cy3 or Cy5 emission) fluorescent intensities are displayed along with the merge colored channel. **B** Expression ratios (mean ± SEM (n = 4 arrays)) between proteins extracted from CMP-treated SH-SY5Y cells compared to control. Protein expression greater in CMP compared to control (red bars) demonstrated an expression ratio of greater than 1.0 while an expression ratio significantly less than unity indicated a reduced expression level induced by CMP (green bars).

As we have demonstrated specific signaling and transcriptomic changes, we next assessed CMP-mediated protein alterations at the signaling network and global proteomic level. Panorama® Cell signaling array antibody chips were used to investigate the specific changes simultaneously in a wide array of signal transduction factors. Control or CMP-treated whole-cell lysates were separately labeled with either Cy3 or Cy5 and then mixed and hybridized with the cell signaling array chips. The criteria for designation of significant up or down-regulation of proteins using the array is described in the *Materials and Methods* section. A sample of a scanned signaling array is depicted in Figure 7A. The relative protein expression changes between the control and CMP samples were calculated for multiple experiments (Figure 7B; Table S21). In accordance with our previous data (Figure 3, Figure S2) we found a generalized suppressive effect of CMP upon signaling functions (66.7% of singificantly-regulated proteins were downregulated: Figure 7). A CMP-mediated regulation of proteins involved in neuronal signaling and transmission (*tryptophan hydroxylase*; *syntaxin*; *synuclein*; *MAP2*; *iNOS*; *connexin 32*; *adaptin*) and calcium homeostasis/regulation (*calnexin*) was noted. In addition a strong CMP-mediated alteration in the expression of proteins that possess a duality of function, *i.e.* positively promote neuronal activity as well as facilitate the initiation of cell death activities (*SMAC/DIABLO*; *c-ABL*; *Apoptosis-inducing factor*; *CDC25*; *p57KIP2*) was observed. To generate a predicted functional network of these CMP-regulated factors we employed a network-developing algorithm using Ingenuity Pathway Analysis (see *Materials and Methods*). The highest probability predicted functional network contained fourteen specific CMP-regulated proteins (*APP*, *CALR*, *CANX*, *CTNND1*, *EGFR*, *ESR1*, *GJB1*, *H3F3A* (*includes EG:3020*), *MAP1A*, *MAPK8*, *NOS2*, *NUTF2*, *RAN*, *TUBA4A*) and was most closely related to the following physiological functions: *Nervous System Development and Function*; *Cell Morphology*; *Cell-To-Cell Signaling and Interaction* (Figure S6, Table S22).

**Figure 8:**
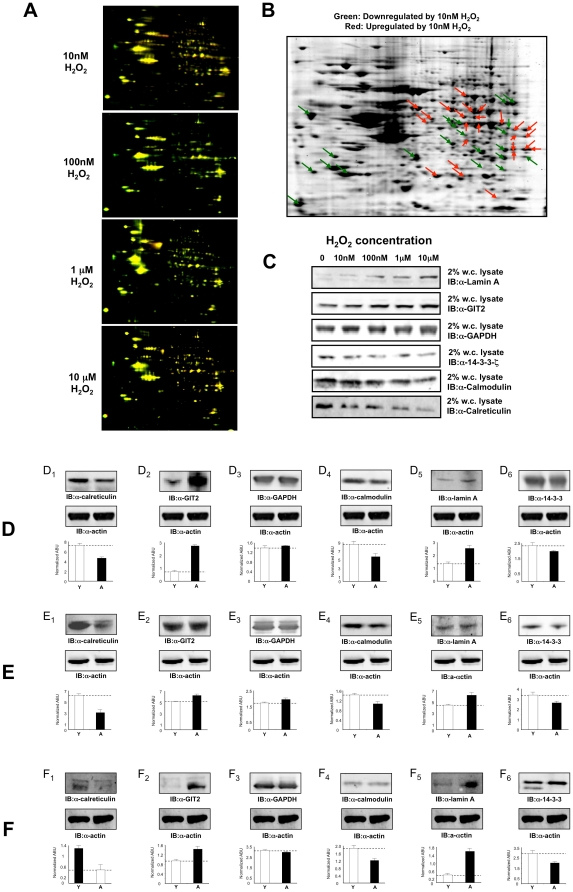
Differential proteomic effects of hydrogen peroxide exposure upon SH-SY5Y cells and age-related protein alterations in multiple species. **A** Multiple differential in-gel two-dimensional electrophoresis (DIGE) images of SH-SY5Y whole cell lysates treated with specific concentrations (10 nM–10 µM) of hydrogen peroxide (Cy5-red) over seven days, compared to control cells (Cy3-green) receiving vehicle (PBS) treatment. **B** depicts a coomassie-stained, two-dimensional gel of Cy5- or Cy3-labeled protein puncta, derived from SH-SY5Y cells exposed to 10 nM peroxide or vehicle control for seven days. Arrows represent puncta that were subsequently excised for mass spectrometric identification. **C** Western blot validation of proteins identified by tandem mass spectrometry of excised puncta. Whole-cell (w.c.) lysates from peroxide-treated (seven days) SH-SY5Y cells were probed with the specific antisera denoted. **D** Normalized expression pattern of validated proteins from panel C in young (Y) or aged (A) rat cortex. D_1_–D_6_ indicate expression of calreticulin, GIT2, GAPDH, calmodulin, lamin and 14-3-3 respectively. Panels E (E_1_–E_6_) and F (F_1_–F_6_) depict analogous protein expression pattern investigation for the same proteins in young (Y) or aged (A) rhesus macaque or human cortex respectively. Protein expression is quantified as actin-normalized arbitrary absorbance units (ABU).

To appreciate the potential adaptive cellular responses to the CMP paradigm in a non-platform-based proteomic format, we employed two-dimensional in-gel electrophoresis (DIGE) to investigate CMP-induced global proteomic changes. Global proteomic changes were assessed for extended exposure to multiple peroxide concentrations, including our CMP paradigm (10 nM, seven days: Figure 8A). We found that >80% of the proteins altered by peroxide treatment were seen consistently across the different peroxide concentrations. Proteins with reliable (n = 6 per H_2_O_2_ concentration: ≥20% expression change) and quantitatively significant expression changes in response to the specific CMP protocol were excised and identified using LC-MS/MS (ThermoFinnigan LXQ) (Figure 8B). The identities of the reliably-regulated CMP-controlled proteins are displayed in Tables 4 and 5. To predict the global functional nature of the CMP-regulated protein set, we employed an unbiased bioinformatic approach. CMP-regulated proteins were clustered (see *Materials and Methods* for criteria) into gene ontology (GO) groups (Table 6) and KEGG (Kyoto Encyclopedia of Genes and Genomes) signaling pathways (Table 7) using parametric gene set enrichment analysis (WebGestalt). The most significantly CMP- populated GO-*biological process* term clusters were linked to: energy regulation (*anaerobic glycolysis*); neuronal development (*regulation of synaptic plasticity*, *neuron differentiation*); small G protein signaling (*Rho protein signal transduction*); translational control (*translational elongation*); protein metabolism (*cytoskeleton and organelle organization and biogenesis*) and cell cycle/apoptosis (Table 6). The most significantly CMP-populated KEGG pathways demonstrated a strong metabolic (*glycolysis/gluconeogenesis*), disease-related (Huntington’s Disease) and cell-signaling (*calcium signaling pathway*, *Wnt signaling pathway*) association (Table 7). We also found that the genes encoding the CMP-regulated proteins were heavily clustered on human chromosome 2 (Figure S7). Using chromosomal gene-disease locus association (www.genecards.org), we identified 246 individual disease-related loci found on human chromosome 2, and out of the 91 loci affected by CMP, 46 were associated with glucose/energy regulation and 45 were associated with neurodegenerative/neurological mechanisms. This may suggest the presence of coherently-regulated gene/protein groups that respond to long-term peroxide-mediated stress. To transfer our proteomic DIGE findings from CMP treatment to more standardized techniques, we randomly chose several proteins (calreticulin, GIT-2, GAPDH, calmodulin, lamin, 14-3-3 zeta) whose expression was elevated or reduced by CMP treatment to demonstrate their direct peroxide responsiveness. We found a strong recapitulation of the DIGE-measured effects on both the up- or downregulated proteins with western blot analysis (Figure 8C Tables 4–5). To further investigate these proteins, and how they may relate to long-term stress exposure, we assessed their expression patterns in young versus aged mammalian cortical tissue from multiple sources, *i.e.* rat, rhesus macaque and human. We hypothesized that the aged animals, compared to the younger, were more likely to have experienced longer term exposure to stressors such as oxygen radicals. Therefore *young* versus *aged* tissues may possess an analogous protein expression profile to *control* versus *CMP* cells respectively. When applying our analytical protein panel to rat (Figure 8D: *young*, 4 months; *aged*, 22–24 months), rhesus macaque (Figure 8E: *young*, 15–17 years; *aged*, 31–35 years) and human (Figure 8F: *young*, 22–25 years; *aged*, 82–85 years) cortical tissue, we noted similar (except for GAPDH regulation in human cortex) protein expression changes to those we observed using our CMP model (Figure 8C, Tables 4–5).

**Table 4:** Down-regulated proteins identified by MS/MS mass spectrometry from DIGE gels following CMP treatment.

Identified Protein	Accession number	Gene Symbol
14-3-3 protein epsilon	IPI00000816.1	YWHAE
14-3-3 protein eta	IPI00216319.3	YWHAH
14-3-3 protein gamma	IPI00220642.7	YWHAG
14-3-3 protein theta	IPI00018146.1	YWHAQ
14-3-3 protein zeta/delta	IPI00021263.3	YWHAZ
29 kDa protein (Tropomyosin 3)	IPI00178083.2	TPM3
3-hydroxyisobutyrate dehydrogenase, mitochondrial precursor	IPI00013860.3	HIBADH
Aldose reductase	IPI00413641.7	ARM
Alpha-centractin	IPI00029468.1	ARP1
Calmodulin	IPI00075248.11	PHKD
Calreticulin precursor	IPI00020599.1	CALR
Citrate synthase, mitochondrial precursor	IPI00025366.4	CS
Elongation factor 1-beta	IPI00178440.3	EEF1B2
Elongation factor 1-gamma	IPI00000875.6	EEF1G
Gamma-enolase	IPI00216171.3	ENO2
heterogeneous nuclear ribonucleoprotein D-like	IPI00011274.2	HNRPDL
Isoform 1 of Calcyclin-binding protein	IPI00395627.3	CACYBP
Isoform 1 of F-actin capping protein subunit beta	IPI00026185.5	CAPPA3
Isoform 1 of Heterogeneous nuclear ribonucleoprotein D	IPI00028888.1	HNRPD
Isoform 1 of L-lactate dehydrogenase A chain	IPI00217966.7	LDHA
Isoform 1 of Tropomyosin alpha-4 chain	IPI00010779.4	TPM4
Isoform 1 of Tropomyosin-1 alpha chain	IPI00014581.1	TPM1
Alpha-enolase	IPI00465248.5	ENO1
Isoform M1 of Pyruvate kinase isozymes M1/M2	IPI00220644.8	PKM1
Isoform M2 of Pyruvate kinase isozymes M1/M2	IPI00479186.5	PKM2
L-lactate dehydrogenase B chain	IPI00219217.3	LDHB
Lung cancer oncogene 7	IPI00641950.3	GNB2L1
Pirin	IPI00012575.1	PIRIN
Proliferation-associated protein 2G4	IPI00299000.5	PA2G4
Proteasome subunit alpha type 4	IPI00299155.5	PSMA4
Rab GDP dissociation inhibitor alpha	IPI00010154.3	RABGDIA
Rab GDP dissociation inhibitor beta	IPI00031461.1	RABGDIB
RcTPM3 (Fragment)	IPI00377005.1	TPM3
Rho GDP-dissociation inhibitor 1	IPI00003815.3	ARHGDIA
T-complex protein 1 subunit delta	IPI00302927.6	TCP1D
T-complex protein 1 subunit eta	IPI00018465.1	TCPIE
tropomyosin 1 alpha chain isoform 2	IPI00000230.6	TPM1
U1 small nuclear ribonucleoprotein A	IPI00012382.3	SNRP70
Voltage-dependent anion-selective channel protein 1	IPI00216308.5	VDAC1

**Table 5:** Up-regulated proteins identified by MS/MS mass spectrometry from DIGE gels following CMP treatment.

Identified Protein	Accession number	Gene Symbol
Alpha-enolase	IPI00013769.1	ENO1B
Beta-centractin	IPI00029469.1	ARP1B
Bifunctional purine biosynthesis protein PURH	IPI00289499.3	PURH
Cofilin-1	IPI00012011.6	CFL-1
Cytoplasmic dynein 1 light intermediate chain 2	IPI00011592.3	DNCH2
D-3-phosphoglycerate dehydrogenase	IPI00011200.5	PHGDH
Glyceraldehyde-3-phosphate dehydrogenase	IPI00219018.7	GAPDH
Heat-shock protein beta-1	IPI00025512.2	HSP70-1B
Heterogeneous nuclear ribonucleoprotein H	IPI00013881.6	HNRPH1
HNRPA2B1 protein	IPI00386854.5	HNRPA2B1
Inosine-5′-monophosphate dehydrogenase 2	IPI00291510.3	IMPDH2
Isocitrate dehydrogenase [NADP], mitochondrial precursor	IPI00011107.2	IDH1
Isoform 1 of Cytosol aminopeptidase	IPI00419237.3	DNPEP
Isoform 1 of RuvB-like 1	IPI00021187.4	RUVBL1
Isoform 2 of ARF GTPase-activating protein GIT2	IPI00186690.6	GIT2
Isoform A of Lamin-A/C	IPI00021405.3	LMNA
Isoform Long of Eukaryotic translation initiation factor 4H	IPI00014263.1	EIF4H
Isoform Long of Glucose-6-phosphate 1-dehydrogenase	IPI00216008.4	H6PD
Isoform Mitochondrial of Fumarate hydratase, mitochondrial precursor	IPI00296053.3	FH
Isoform Short of Proteasome subunit alpha type 1	IPI00016832.1	PSMA1
paraspeckle protein 1	IPI00103525.1	PSP1
Peroxiredoxin-6	IPI00220301.5	PRDX6
Phosphoglycerate kinase 1	IPI00169383.3	PGK1
Platelet-activating factor acetylhydrolase IB subunit gamma	IPI00014808.1	PAFAH1B3
Poly(rC)-binding protein 1	IPI00016610.2	PCBP1
poly(rC)-binding protein 2 isoform b	IPI00012066.2	PCBP2
Pre-mRNA-processing factor 19	IPI00004968.1	PRP19
Proteasome subunit alpha type 6	IPI00029623.1	PSMA6
Protein disulfide-isomerase A3 precursor	IPI00025252.1	PDIA3
RuvB-like 2	IPI00009104.7	RUVBL2
Septin-11	IPI00019376.6	SEPT11
Aldehyde dehydrogenase family 7 member A1	IPI00221234.5	ALDH7A1
Phosphoglycerate mutase 1	IPI00453476.2	PGAMB
Stress-induced-phosphoprotein 1	IPI00013894.1	STIP1
T-complex protein 1 subunit beta	IPI00297779.7	TCP1B
Torsin A interacting protein 1	IPI00644766.2	TOR1AIP
Ubiquinol-cytochrome-c reductase complex core protein 2, mitochondrial precursor	IPI00305383.1	UQCRC2

**Table 6:** Gene Ontology-biological process term analysis of consistently-regulated proteins in CMP cells compared to control.

GO-bp	Enrichment R (O/E)	Probability (p)	Hybrid
***Energy regulation***			
anaerobic glycolysis	100	1.16E-04	393.5542
tricarboxylic acid cycle intermediate metabolism	50	8.63E-07	303.1995
pentose-phosphate shunt	66.67	4.13E-04	225.6146
tricarboxylic acid cycle	37.5	7.39E-05	154.9258
***Neurophysiology***			
regulation of neuron differentiation	66.67	5.16E-04	219.1676
regulation of synaptic plasticity	50	6.29E-04	160.0675
neuron differentiation	7.14	3.11E-02	10.76169
***Cellular signaling***			
regulation of GTPase activity	21.43	3.74E-04	73.44336
negative regulation of protein kinase activity	16.67	6.82E-03	36.11081
Rho protein signal transduction	6.67	3.70E-02	9.550095
***Translation regulation***			
translational elongation	25	2.82E-03	63.74377
mRNA catabolism	25	2.82E-03	63.74377
nucleotide metabolism	6.06	4.08E-03	14.4794
RNA processing	4.26	2.58E-03	11.0265
purine nucleotide biosynthesis	7.14	3.11E-02	10.76169
purine nucleotide metabolism	6.9	3.33E-02	10.19513
regulation of translation	6.25	4.17E-02	8.62415
***Cell cycle***			
regulation of mitosis	12.5	1.05E-02	24.73513
regulation of progression through cell cycle	3.77	4.66E-03	8.790185
***Cell death***			
apoptosis	3.55	3.09E-03	8.910647
***Protein metabolism***			
cytoskeleton organization and biogenesis	3.88	8.80E-03	7.975407
organelle organization and biogenesis	2.65	9.26E-03	5.388481
regulation of protein metabolism	3.8	4.38E-02	5.162398
protein folding	3.7	4.67E-02	4.923528

**Table 7:** KEGG pathway analysis of consistently-regulated proteins in CMP cells compared to control.

KEGG pathway	Enrichment R (O/E)	Probability (p)	Hybrid
***Energy regulation***			
Glycolysis/Gluconeogenesis	67.189	3.73E-10	633.4793
Reductive carboxylate cycle (CO2 fixation)	120.482	1.22E-04	471.5228
Citrate cycle (TCA cycle)	73.349	9.29E-06	369.094
***Metabolism/biosynthesis***			
Phenylalanine, tyrosine and tryptophan biosynthesis	109.89	1.47E-04	421.1739
Pyruvate metabolism	49.587	3.10E-05	223.5689
Cysteine metabolism	60.06	5.08E-04	197.8462
Cell cycle	29.779	7.33E-07	182.6947
Propanoate metabolism	41.322	1.08E-03	122.5858
Purine metabolism			38.6742
***Pathophysiology***			
Huntington's disease	52.91	6.58E-04	168.348
Pathogenic Escherichia coli infection - EPEC	30.722	1.95E-03	83.25556
Pathogenic Escherichia coli infection - EHEC	30.722	1.95E-03	83.25556
Antigen processing and presentation	18.365	5.36E-03	41.70502
***Cell signaling***			
Insulin signaling pathway	10.1678	1.66E-02	18.09758
Wnt signaling pathway	9.241	1.99E-02	15.72217
Calcium signaling pathway	7.9618	2.26E-02	12.59317

## Discussion

We have attempted to create a viable cell model of long-term, low-level oxidative stress and use this to investigate, at multiple levels, the alterations of cellular function and physiology imposed by this tolerable insult. The chronic minimal peroxide (CMP) protocol induced adaptive changes in SH-SY5Y cells that could aid research into long-term cellular damage and stress *in vivo*. The CMP regimen caused profound cytoskeletal re-organization, reduction of mitochondrial expression, attenuation of glucose uptake elevated basal calcium and c-Src activity, as well as changes in the transcription of genes and expression of proteins linked to energy alteration and neurodegenerative processes. In contrast to more deleterious levels of peroxide exposure (≥0.1 M), CMP did not significantly affect membrane electrophysiological parameters or responses to excitatory amino acids. This subtle model of long-term cellular stress may provide a useful addition to bioscreening processes, especially when studying highly complex events such as ligand response profiles or protracted physiological processes. Obviously different cellular models of long-term cellular stress could be employed. Each model will possess its own unique advantages and drawbacks. The advantages of using a clonal cell culture, such as SH-SY5Y cells, are that cultures will be consistent, easy to maintain, possess a human phenotype and can be readily used for large-scale drug-screening. Some of their drawbacks include, variation in cell phenotype with passage and their neuroblastoma lineage. On the other hand, primary neuronal culture systems can provide a strong neuronal phenotype, however they may not be suitable for large-scale drug screening for the following reasons, *i.e.* costly and labor-intensive preparation, variation of culture and cell populations between animals and embryonic tissue usage. It is clear that different models offer different possibilities and not one will be able to suit all needs. The choice of the model system will be strongly influenced by the nature of the intended investigation.

CMP treatment, at multiple levels was able to engender a strong recapitulation of multiple aspects of long-term cell stress that could represent a limited molecular proxy for aging or neurodegenerative processes. CMP treatment mimicked aging/neurodegenerative changes in cytoskeletal architecture [42], [43], impaired mitochondrial activity and biogenesis [44]–[48], altered glucose uptake/mobilization [49]–[53], disrupted calcium homeostasis [54]–[61], and alterations in energy management systems [62]–[64]. In addition, multiple CMP-induced genomic changes were linked to protracted stress, energy and calcium regulation as well as neurodegenerative processes, *e.g. DNM1L* [65], [66], *SPTLC1* [67], [68]) and *ENDOG* [69]. Relatively novel CMP-controlled factors linked to neurodegeneration were uncovered, *e.g.* APAF1-interacting protein (*APIP*: [70], [71]) which typically protects against hypoxic-related cell damage but was downregulated in response to CMP (Table S1). Inositol hexakisphosphate kinase 1 (*IHPK1*), which catalyses the creation of higher forms (hexaphosphates) from inositol (1,4,5) trisphosphate, has been linked to cellular and somatic energy management [72]–[75]. We noted a CMP-mediated potentiation of *IHPK1* expression (Table S1: Figure S3) and it has been demonstrated that elevated cortical expression of *IHPK1* occurs in an in-bred murine model of accelerated aging (senescence-accelerated mouse/prone 8 (SAMP8: [76]). The SAMP8 mouse demonstrates reduced lifespan compared to control normal-aging SAMR1 mice [76] as well as age-related cognitive decline in a manner reminiscent of Alzheimer's disease [77], [78]. Application of therapeutics, demonstrated to improve ischemia-induced disrupted spatial cognition and central neuronal cholinergic function [79], to this model were shown to reduce the elevated levels of cortical *IHPK1*, resulting in amelioration of the SAMP8 cognitive deficits [80].

Suggesting a potential systemic connection between long-term oxidative stressors and age-related disorders we noted a strong clustering of the genes for CMP-controlled proteins on chromosome 2. This chromosomal region contains a considerable number of loci that have been associated with the induction of age/stress-related diseases [81]–[86]. In addition, using an antisera chip approach we demonstrated CMP-induced expression changes of signaling proteins closely associated with long-term stress and aging/neurodegenerative processes including: synuclein; syntaxin; *SNAP-25*; *p57KIP2*; *RAN*; *AKT-1*; p120 catenin; *iNOS*; estrogen receptor; epidermal growth factor receptor; dystrophin; desmin; connexin 32; cofilin; calnexin; c-Jun N-terminal kinase; c-ABL [87]–[105]. Employing two-dimensional DIGE we were able to generate a specific CMP proteomic response *‘signature’*, independent of pre-set protein targets. Bioinformatic analysis of this CMP protein expression ‘*signature*’ (*i.e*. reliably CMP-altered proteins) revealed a strong energy-regulatory and potentially degenerative phenotype of this dataset (Tables 6, 7). We assessed whether protein targets, identified using DIGE, (calreticulin, GIT2, GAPDH, calmodulin, lamin, 14-3-3 zeta: Figure 8) could be indicative of advancing cellular stress associated with age. With investigation of the age-related expression profile of these targets, many of which are associated with long-term stress or aging itself [106]–[110], in young versus aged rats, primates and humans, we noted strong analogy between control versus CMP with young versus aged states (Figure 8C–F). In addition to these signaling protein alterations we also demonstrated a CMP-mediated elevation in basal c-Src activity and GPCR-mediated ERK1/2 responses, both of which are linked to the aging/neurodegenerative process [111]–[116]. While GPCR activity in CMP cells was both potentiated (ERK1/2) and attenuated (c-Src), for different signaling effectors, neurotrophic BDNF signaling however was consistently attenuated in the CMP state. Unbiased genomic analysis of the effects of the CMP protocol demonstrated a geneset bias towards disruption of neurotrophin signaling versus GPCR-based signaling (Figures 4, 5, 6) and was more indicative of cells in an aging/neurodegenerative state, compared to a young healthy state (Figure 4). CMP caused a more profound disruption of the early genotropic output from BDNF compared to MeCh stimulation at multiple levels of investigation (Figure 5–6). Multiple genes upregulated by BDNF in control conditions were downregulated by BDNF in CMP conditions (Figure 6, Tables S18, S19, S20). Many of these genes possess multifactorial roles in the aging/neurodegeneration process, *e.g. PTMA*, *WSB2*, *RPL7*, *HMGB2*, *DLDH* and *DDX17* [117]–[125]. In the CMP state the direction of BDNF-mediated regulation of the transcription factor *YY1* [126] was reversed. In CMP cells BDNF downregulated *YY1* transcription factor while in control cells BDNF upregulated its expression. *YY1* expression can control iron-transferrin homeostasis in an age-dependent manner [127], regulate cell senescence [128] and Alzheimer's disease-related amyloid processing [129]. Disruption of BDNF-regulated *YY1* transcription could therefore exert multiple deleterious neural effects. BDNF transcriptional regulation of T-complex 1 (*TCP-1*) was also reversed by CMP treatment (reduction in CMP state, potentiation in control state). *TCP-1* was first identified as a molecular chaperone for tubulin. *TCP-1* disruptions are associated with the accelerated aging in SAMP8 animals [130], [131], synaptic disruption in murine Huntington's disease models [132] and is downregulated (as in CMP state) in aging rat synapses [133]. BDNF-mediated upregulation of hypoxia-induced factor-1 (*HIF1A*) in control cells was also reversed with CMP cellular treatment (Figure 6B). BDNF typically controls *HIF1A* expression through a phosphoinositide 3-kinase/mTOR-dependent mechanism [134]. *HIF1A* has been associated with age-related degenerative disorders, *e.g.* Alzheimer's and Parkinson's disease [135] and genetic ablation of its expression significantly accelerates the onset of cellular senescence [136]. Conversely, dietarily- or genetically-induced elevation of *HIF1A* in aged animals exerts strong neuroprotective effects and can extend lifespan [137], [138]. Therefore, the CMP-induced reversal of BDNF-mediated *HIF1A* regulation reproduces the pro-aging *HIF1A* profile observed by multiple researchers. Altered neurotrophin receptor signaling has been linked to pathophysiologies associated with stress and aging in multiple systems including animal models as well as human epidemiological and post-mortem data [29], [139]–[148]. This may suggest that neurotrophin dysregulation may potentially be an important early effect in the degenerative or aging processes. While this aging-neurotrophin association has been the subject of intense research it is likely in the future that additional, equally important, stress-response mechanisms will also be uncovered. For this study we have focused on BDNF and five other GPCR ligands that have been implicated in aging and neurodegenerative disease. Clearly future work is needed that will carefully characterize the effects of CMP upon numerous other receptor-based cell signaling systems.

In conclusion, the CMP process creates a viable cell model for the study of cellular responses to protracted, tolerable oxidative stress. While limited by the nature of *in vitro* cultured neural cells we have seen that molecular alterations induced by CMP may in-part mirror those molecular changes seen in brain tissues that have experienced long-term stress. In some ways this model may assist in the understanding of the complex series of molecular mechanisms involved in aging and neurodegenerative disease. It is highly unlikely that any cellular model can completely recapitulate such a complex multidimensional process, however the CMP model recapitulates some of the correlated facets of the physiological and pathophysiological aging process in experimental animals and humans. The CMP model demonstrates that at multiple levels of experimental interrogation systemic alterations of cellular function, that may act in concert to allostatically [149] create a multi-dimensional phenotype that could be useful for research into aging and neurodegeneration. The CMP phenotype demonstrates itself at multiple levels of functionality, indicating a profound depth of coherent cellular regulation. A greater understanding of this multifactorial phenotype could lead to improved cell-based assays for the screening and development of therapeutics that target aging/neurodegenerative disorders.

## Materials and Methods

### Cell Culture and Treatment

SH-SY5Y, obtained from ATCC (http://www.atcc.org/), cells were maintained at 37°C in a humidified 5% CO_2_ incubator in AMEM/F12 supplemented with 10% FBS, 100 units/mL penicillin/100 units/mL streptomycin. Cells of passage number 5–15, from ATCC, were used for all experiments to prevent any alteration of growth or response phenotype. During experimentation no significant changes of required passaging rates or gross morphology were noted. Cells were treated with either phosphate-buffered saline (PBS) (control), 10 nM, 100 nM, 1 µM or 10 µM hydrogen peroxide (H_2_O_2_: Sigma Aldrich, St Louis MO) for seven days. Cell growth media and applied hydrogen peroxide treatments were changed daily.

### Mammalian Tissue Analysis

Animal care and experimental procedures followed NIH guidelines and were approved by the National Institute on Aging Animal Care and Use Committee. Male Sprague-Dawley rats (ages 4 months, n = 3: 24 months, n = 3) were euthanized humanely with isoflurane and cortical tissue was excised as previously described [150]. Cortical tissue from male Rhesus Macaque (ages 15–17 years, n = 3: 31–35 years, n = 3) was obtained from the National Institute on Aging Division of Aging Biology and the Wisconsin National Primate Research Center. Human male post-mortem cortical tissue (ages 22–25, n = 3: 82–85 years, n = 3) was obtained from an approved tissue source, *i.e.* Integrated Laboratory Service–Biotech (Chestertown MD). Tissue lysates were prepared from the mammalian tissues using the Qproteome™ tissue fractionation kit (Qiagen). Lysate concentrations were measured using a standard BCA assay (Pierce-ThermoElectron) and normalized to a 1 mg/mL concentration before resolution by SDS-PAGE.

### Western Blotting

For western blotting protocols cell monolayers were washed once in ice-cold PBS and then lysed in an NP-40 based buffer (250 mM NaCl, 5 mM HEPES, 10% v/v glycerol, 0.5% NP-40, 2 mM EDTA (pH 8.0), 100 µM NaVO_4_) supplemented with a complete mini protease inhibitor cocktail (Roche Diagnostics, Indianapolis, IN) [151]. Lysate concentrations were measured using a standard BCA assay and normalized to 1 mg/mL total protein then resolved using gel electrophoresis followed by electrotransfer to polyvinylenedifluoride (PVDF: Perkin Elmer; Waltham, MA). PVDF membranes were blocked for one hour at room temperature in 4% non-fat milk (Santa Cruz; Santa Cruz CA) before application of specific primary antisera. The presence of primary antibody reactivity with the PVDF membrane was detected by the application of a 1∶5000 dilution of a species-specific alkaline phosphatase-conjugated secondary antibody (Sigma, St. Louis, MO). PVDF-bound immunecomplexes of secondary and primary antibodies were subsequently detected using enzyme-linked chemifluorescence (ECF: GE Healthcare; Pittsburgh PA). Chemifluorescent signals from membranes were quantified using a Typhoon 9410 phosphorimager (GE Healthcare; Pittsburgh PA). Specific primary antisera used were obtained from the following sources: lamin-A, glyceraldehyde-3-phosphate dehydrogenase (GAPDH), calmodulin, calreticulin, extracellular signal-regulated kinase (ERK), c-Src, 14-3-3 zeta and beta-actin - Santa Cruz Biotechnology, Santa Cruz, CA; GIT-2 - NeuroMab, San Jose CA; tyrosine-418 phosphorylated c-Src - Invitrogen, Carlsbad CA; phospho-ERK, phospho-Akt-1 and Akt-1 - Cell Signaling Technology, Danvers MA).

### Two-Dimensional Differential In Gel Electrophoresis (DIGE)

Growth media was aspirated and cells were washed twice with PBS before being lysed in 7 M urea, 4% CHAPS and 30 mM Tris, pH 8.5 [152]. Samples were agitated (30 minutes, RT) then sonicated on ice and centrifuged (10minutes, 10000×g). Supernatant was then applied to a 3000 Da molecular weight cut-off centrifugation spin-filter (Millipore, Billerica MA) to remove excess salts. Protein concentrations were re-determined with a BCA assay and 15 µg of total protein from each sample was labeled with 0.3 µL (120pmol) of Cy3 or Cy5 (GE Healthcare, Pittsburgh PA) according to the manufacturer's instructions. A pre-mixed aliquot of equal protein for control and test sample was labeled with 0.3 µL (120pmol) of internal standard Cy2. A total of 5 µg protein from each H_2_O_2_ treatment was mixed with 5 µg of total protein from the control group and 5 µg internal standard, allowing for a total of 15 µg protein to be loaded onto each gel. The solution was mixed with 125 µL of rehydration buffer (7 M Urea; 4% CHAPS; 2 M thiourea; 1% carrier ampholytes, pH 3–10, and 10 mM DTT) and incubated with a 7 cm immobiline dry strip, pH 3–10 NL (GE Healthcare). Gels were actively loaded at 50 V for 2 hrs using a Protean IEF cell (Bio-Rad) before being subjected to isoelectric focusing as follows: 0–250 V linear increase for 15 min; 250 V–500 V linear increase for 15 min; 500 V–1000 V rapid increase for 60 min; 1000 V–8000 V rapid increase for 60 min and a linear increase to 8000 V for 8000 Vhrs. The strips were then reduced (1% DTT, Sigma) in equilibration buffer (50 mM Bis-Tris, 6 M Urea, 30% glycerol, 2% SDS, pH 6.8) for 15 min at room temperature followed by alkylation (2.5% iodoacetamide, Sigma) in equilibration buffer for 15 min at room temperature with constant agitation. Second dimension gels (4–12% Bis-Tris acrylamide gels: Invitrogen Corporation) were separated at 80 V and scanned on a phosphorimager (Typhoon 9410, GE Healthcare Systems).

### Linear Ion-Trap Tandem Mass Spectrometry

Gel spots demonstrating consistent (reliable direction of alteration, *i.e.* up or downregulated by ≥20%, n = 6) differential expression from DIGE staining were excised, incubated with iodoacetamide and digested with sequence-grade trypsin (Promega Corp., Madison WI) overnight at 37°C. After digestion, peptides were extracted twice with extensive vortexing upon addition of 50/45/5 ACN/H_2_O/Formic Acid. Peptide digests were dried with vacuum centrifugation, resuspended with 0.1% trifluoroacetic acid and cleaned via C18 ziptips (Millipore, Billerica MA). Peptides were then diluted with 2% ACN, 98% H_2_O, 0.1% formic acid and loaded with a Surveyor (ThermoFinnigan, Waltham MA) autosampler onto a 10 cm×75 micron C18 column poured ‘in-house’. Peptides were eluted with an ACN gradient delivered with a Surveyor (ThermoFinnigan) HPLC at 300 nL/min from 2–40% over 40 minutes. Eluting ions were analyzed using an LXQ ion trap mass spectrometer (ThermoFinnigan) in a data-dependent manner. The 3 most abundant ions from a full scan were selected for fragmentation via collision-induced dissociation. Data was extracted and searched using BioWorks software and the Sequest algorithm (ThermoScientific) against an IPI human protein database. Identified peptides were filtered using Xcorr cutoff values of 2.5 and 3.0 for +2 and +3 charges. Resulting protein identifications were further validated by matching theoretical mass and pI (isoelectric point) values to their respective spots from the two-dimensional gel.

### Ligand Stimulation

Cells were serum-deprived overnight prior to ligand stimulations with either: 10 nM dopamine, anandamide, lysophosphatidic acid (LPA), histamine, β-methylcholine (all obtained from Calbiochem, Gibbstown NJ) or 10 ng/mL brain derived neurotrophic factor (BDNF: Invitrogen, Carlsbad CA) for 0, 2, 5, 10, 30 and 60 minutes. Stimulation was terminated by aspiration of culture media followed by one wash in ice-cold PBS and then subsequent lysis in 80 µL of the previously mentioned NP-40-based lysis buffer. Cells were scraped, agitated (40minutes, 4°C) then clarified by centrifugation (14000×g, 15minutes, 4°C). Supernatant concentrations were determined by BCA assay, normalized to 1 mg/mL, then processed for blotting as described previously (Western Blotting).

### Confocal Microscopy

Control or CMP SH-SY5Y cells were seeded in 8-well chamber slides (Nunc Technologies, Rochester NY). For cell fixation, cells were serum-deprived overnight, washed with PBS and fixed in ice cold 100% methanol (10 minutes, −20°C). Monolayers were washed with PBS and incubated in an NP-40-based permeabilization buffer (PBS, 10% fetal calf serum, 1% bovine serum albumin, 0.2% Nonidet P-40, 30 minutes[153]). Fixed cells were then blocked in PBS containing 10% fetal calf serum and 1% bovine serum albumin (60 minutes, RT, agitation). Monolayers were then incubated with 1∶50 dilution of Alexafluor-568-phalloidin (Molecular Probes) for 1 hour followed by a 1∶1000 dilution of α-tubulin (Sigma: 60 minutes, RT). Cells were then incubated with a 1∶2000 dilution of fluoroscein isothiocynate-conjugated anti-mouse antibody for 1 hour at room temperature. Monolayers were washed with PBS (3×5 min) between primary and secondary antibody incubation. For mitochondrial staining slides were washed and fixed as before, and incubated with 10 µM Mitotracker (Invitrogen, Carlsbad CA) for 20 minutes. Monolayers were washed with PBS (3×5 min) before being visualized using an Olympus Fluoview 3000 confocal microscope. To quantify alterations in Mitotracker fluorescence, 100 microscopic fields were averaged for their fluorescent intensity (Fuji Image Systems, ImageGauge v. 3.5) and averaged between control and CMP conditions. Mitochondrial membrane potentials were also measured, in live cells, using fluorescent probe analysis after cellular loading with tetramethylrhodamine ethyl ester (TMRE: Invitrogen, Carlsbad CA).

### Fluorescent Fluo-4 Imaging

Cells were seeded in a 35 mm glass-bottom culture dish (MatTek Corporation, Ashland MA). Before dye-loading, cells were serum-deprived overnight before equilibration in ACSF (124 mM NaCl, 5 mM KCl, 1.25 mM NaH_2_PO_4_, 2 mM MgSO_4_, 10 mM glucose) and supplemented with 5 µM Fluo-4AM (Invitrogen, Carlsbad CA: 20minutes, 37°C). Cells were then washed three times with fresh ACSF and imaged. For each experiment, either 100 µM CPA, 10 µM nifedipine, 1 µM ω-conotoxin or 50 µM glutamate was added to the cells at intervals depicted in the relevant figures. Using digitization and quantification (Fuji Image Systems Ltd.: L-Process and ImageGauge) of multiple cells (n = 10) from multiple microscopic fields (n≥3) we were able to generate relative intensity measurements for Fluo-4 fluorescent intensity (Arbitrary Units-Background/square pixels: (AU-B/px^2^)) for control or CMP cells. Measurement of calcium levels was then performed as described previously [154].

### Glucose Uptake Assay

SH-SY5Y cells, control or CMP-treated, were glucose- and serum-deprived in Krebs Ringer buffer (25 mM NaCl, 5 mM KCl, 1.25 mM NaH_2_PO_4,_ 2 mM CaCl_2,_ 1 mM MgCl_2,_ 25 mM NaHCO_3_) at 37°C and 5% CO_2_ for 40 min on the day of experimentation. To determine glucose uptake rates, 20 µM glucose was added to the Krebs Ringer buffer and 30 µL aliquots were taken at 0, 10, 15, 30, 60 and 120 min. Glucose concentration from each aliquot was determined using a fluorescent glucose assay kit (BioVision, # K606-100) as per manufacturer's instructions. Cells were lysed at the end of the experiment and protein concentration was determined through a BCA assay. Glucose uptake curves were then normalized for each experiment against the respective total protein concentration measured. To determine lactate production, cells were serum starved for 40 minutes, in Krebs Ringer buffer, before being exposed to 5 mM glucose for 4 hrs. The lactate produced and the glucose taken up by the cells was measured using colorimetric lactate (BioVision, # K627-100) and glucose (BioVision, # K606-100) assay kits, per manufacturer's instructions. Cells were lysed at the end of the experiment and protein concentration was determined through a BCA assay. The glucose uptake curve was then normalized against the protein concentration.

### Signaling Network Antibody Microarray

Preparation of protein samples, labeling, application to the Panorama® Cell Signaling Array chip and data analysis was performed according to manufacturers' instructions using the proprietary solutions and equipment provided (Panorama® Cell Signaling Antibody Microarray, Sigma, St. Louis MO). Briefly, cell lysates from control and H_2_O_2_ treatment batches were labeled with Cy3 or Cy5 dye (GE Healthcare, Waltham MA) and the two samples were applied simultaneously at equal protein concentrations to the array. To ensure that comparisons of the two complex samples of whole cell lysates were not confounded by gross alterations in the proteome, we first assessed the global protein expression using coomassie staining of the input samples as well as specific antibody staining with anti-β-actin sera (data not shown). The differential fluorescent signal intensity for each antibody spot was then recorded with a phosphorimager (Typhoon 9410, GE Healthcare). Each chip image was manually assessed for quality of spot detection and quantitation by at least two independent observers. Dye swapping (control or peroxide treated were labeled in different experiments with either Cy3 or Cy5) was employed and internal standards (bovine serum albumin, actin) were used to normalize the fluorescent intensities for each antibody. For each antibody spot, fluorescent emission intensity was recorded across the two primary, Cy3 (570 nm) or Cy5 (670 nm), emission wavelengths. Ratios of the individual spot intensities (Cy-dye independent as dye-sample swapping was performed) of CMP compared to control samples were recorded (n = 4). To consider differential expression, the mean of the four spot ratios had to be significantly different (greater or less than) to unity as well as the internal controls (p<0.05, non-paired Student's t-test). Only significantly regulated spots/antibody identifications were considered for further analysis.

### RNA extraction and Fluorometric Gene Array Analysis

RNA extraction and microarray analysis were carried out as described previously [155]. RNA was isolated using the Qiagen RNeasy Mini Kit (Qiagen, Inc. Valencia CA). RNA quality and quantity was checked using an Agilent 2100 bio-analyzer and the RNA 6000 nano-chips. Total RNA was used to generate biotin-labeled cRNA using the Illumina TotalPrep RNA Amplification Kit (Ambion; Austin, TX). A total of 0.75 µg of biotin-labeled cRNA was hybridized at 58°C for 16 hours to Illumina's Sentrix MouseRef-8 Expression Bead-Chips (Illumina, San Diego, CA). Arrays were then washed, blocked and labeled cRNA was detected by staining with streptavidin-Cy3. Arrays were then scanned using an Illumina BeadStation 500× Genetic Analysis Systems scanner and the image data extracted using the Illumina BeadStudio software, Version 3.0. All microarray data is MIAME compliant and the raw data has been deposited in a MIAME compliant database.

### Bioinformatic analysis

Parametric analysis of datasets generated was conducted using WebGestalt (http://bioinfo.vanderbilt.edu/webgestalt/). Analysis was employed to identify the gene ontology (GO) term and KEGG (Kyoto encyclopedia of genes and genomes) pathway groups that most accurately represented the phenotypes of input datasets. We employed the following parameters for the significant inclusion of specific GO term and KEGG pathway groups, *i.e.* each group needed a minimum population of two genes/proteins from the input dataset and also possess a probability significance of enrichment compared to a background dataset of less than 0.05 (hypergeometric test). The degree of enrichment (R: expressed as a ratio) of each GO term/KEGG pathway was calculated as follows: R = O/E where O is the observed protein/gene number in the KEGG pathway/GO term cluster, E is the expected gene/protein number in the KEGG pathway (Expected number of proteins in a specific KEGG pathway for a specific experimental protein set  =  Total number of proteins in the KEGG pathway for the reference set x Total number of proteins in the experimental set/Total number of proteins in the reference set). Using the Network Explorer function in Ingenuity Pathway Analysis (IPA v8.5), the most significantly populated gene networks for a specific geneset were calculated based on the percentage population (by network eligible focus molecules from input dataset), mediated by the gene identities of the input datasets, of the resultant gene networks. A comprehensive overview of the use of network algorithms and significance generation is given in [156]. Genes were only considered to be network eligible if they were known to interact with at least one other molecule in the network. In each case more than two genes were required to adequately populate a given network with at least a probability (*p*) value of less than 0.05. GeneIndexer (Computable Genomix LLC, Memphis TN), was employed to indicate the degree of correlation of input interrogation terms with significantly-regulated genes in a given dataset. GeneIndexer generates correlation scores (0.1 minimal cut-off to 1.0) for a specific gene in the geneset to the interrogation term using a species-specific scientific abstract database containing over one million human-curated entries. The basal transcriptional dataset (CMP versus control) was interrogated with terms that would potentially reveal a neurotrophin-based or age-related bias in the CMP-mediated transcriptome set. To generate the GeneIndexer matrix, only genes that were linked to at least two interrogation terms were considered (Figure S4).

### Electrophysiology

Whole-cell patch-clamp recordings were performed with an Axopatch 200B (Axon Instruments, Union City, CA, USA) amplifier at RT. SH-SY5Y cells were plated at a density of 15,000 per 15 mm round glass coverslip (Warner Instruments Inc., Hamden, CT). Coverslips containing cells were placed in a recording chamber mounted on an inverted microscope (BX51WI; Olympus, Tokyo, Japan). Cells were perfused with a standard extracellular recording solution (in mM: NaCl 135, KCl 5, NaH_2_PO_4_ 2, CaCl_2_ 2, MgCl_2_ 1, NaHCO_3_ 25, D-Glucose 10, pH 7.35, 300–315 mOsm). Patch pipettes were manufactured from borosilicate capillary glass (G150F-4; Warner Instruments Inc.) using a micropipette puller (P-97; Sutter Instruments, Novato, CA, USA). Patch pipette resistance was 3–5 MΩ when filled with intracellular recording solution (in mM: potassium gluconate 120, NaCl 5, EGTA 0.1, HEPES 10, KCl 20, MgCl_2_ 4, phosphocreatine 10, magnesium-ATP 4, sodium-GTP 0.3, pH 7.3, 290 mOsm). Whole-cell currents were recorded at 10 kHz and low-pass filtered at 2 kHz, using Pclamp 10 Software (Axon Instruments). Recordings were transferred via a DAC (Digidata 1440A; Axon Instruments) to a Dell PC and analyzed offline with Clampfit 10 software (Axon Instruments). SHY-5Y resting membrane properties were assessed during hyperpolarizing 10 mV steps. Resting membrane potentials were determined by switching to I = 0 mode, immediately following whole-cell configuration. Unless indicated otherwise, cells were voltage clamped at −40 mV with series and pipette capacitance compensation.

## Supporting Information

Figure S1 Chronic minimal peroxide treatment does not significantly affect membrane electrophysiological properties. A Current clamp family recordings from control- or CMP-treated SH-SY5Y cells subjected to injection of steps from 20pA to +100pA. B Steady-state current/voltage relationships gained in voltage-clamp mode for control or CMP SH-SY5Y cells subjected to voltage steps from −40 mV to +140 mV. C Classical cell membrane parameters assessed for both control and CMP cells included resting membrane potential (Vrest), input resistance (Rinput), access resistance (Raccess) and membrane capacitance (Cm).
(0.84 MB TIF)

Figure S2 CMP affects multiple receptor systems with respect to c-Src and extracellular signal-regulated kinase (ERK1/2) activation. A. Dopamine- (DA, 10 nM), histamine- (HA, 10 nM), lysophospahtidic acid- (LPA, 10 nM) and anandamide- (ADA, 10 nM) mediated c-Src tyrosine-418 autophosphorylation in control (blue bars) or CMP-treated (red bars) cells for the respective times specified. B. Dopamine- (DA, 10 nM), histamine- (HA, 10 nM), lysophospahtidic acid- (LPA, 10 nM) and anandamide- (ADA, 10 nM) mediated ERK1/2 activation in control (blue bars) or CMP-treated (red bars) cells for the respective times specified. Statistical significance is indicated for changes in kinase activity in the CMP state relative to their time-matched control in vehicle-treated (control) cells. Statistical significance was measured using a Student's t-test (GraphPad Prism v.3): * - p<0.05; ** - p<0.01.
(1.81 MB TIF)

Figure S3 Textual correlation of multiple CMP-regulated factors in SH-SY5Y cells. A. Heatmap representation of CMP-significantly-regulated genes (gene symbol identities list on the left of the heatmap) and their multiple correlation with various Latent Semantic Indexing interrogation terms (Energy regulation, Ca2+ regulation, Glucose metabolism, Mitochondria, Aging, Oxidation, Neurodegeneration, Stress). A red block indicates that the specific gene correlates implicitly (≥0.1 correlation score) with the interrogation term and was upregulated in response to CMP, while green indicates a downregulated implicitly correlated (≥0.1 correlation score) gene. A black block represents a lack of correlation between the specific gene and interrogation term.
(1.57 MB TIF)

Figure S4 Venn diagram analysis of BDNF- or MeCh-induced transcriptional regulation. A Venn diagram analysis of global significantly-regulated genes with either 2, 4, or 8 hours of BDNF stimulation (CMP-red circle, Control-blue circle). B Venn diagram analysis of global significantly-regulated genes with either 2, 4, or 8 hours of MeCh stimulation (CMP-red circle, Control-blue circle). Genes commonly regulated in control and CMP conditions are indicated by black numbers.
(2.36 MB TIF)

Figure S5 Response series-specific moduli of z ratio differences between commonly CMP-Control regulated genes. A. Modulus scores (independent of polarity of z ratio difference) for control (CTRL) and CMP differences in z ratio after 2 hours of stimulation with either MeCh or BDNF demonstrate a z ratio difference. Similar series-specific z ratio difference modulus scores for 4 (B) or 8 (C) hours of stimulation are depicted below. For both BDNF or MeCh the gene regulation series are as follows: series 1 = upregulated in CMP and control with z ratio CMP>CTRL; series 2 = upregulated in CMP/control with z ratio CTRL>CMP; series 3 = downregulated in CMP/control with z ratio CMP<CTRL; series 4 = downregulated in CMP/control with z ratio CTRL<CMP. Values in each histogram are representations of mean ± SEM (n = 3). Statistical significance was assessed using a Student's t-test. * p<0.05, ** p<0.01.
(1.11 MB TIF)

Figure S6 Signal transduction network analysis of CMP-regulated factors in SH-SY5Y cells. Using an un-biased signaling network-generating algorithm (Ingenuity Pathway Analysis) the most likely protein-protein interaction cluster was created from the significantly altered signaling proteins in response to the CMP protocol. The figure depicts the highest scoring functional interaction network created from the CMP-controlled signaling factors. Proteins denoted in capitalized text were directly derived from the input protein set. A full description of the nature of interactions based on the connecting lines can be found at the following webpage linked to the IPA analysis module (https://analysis.ingenuity.com/pa/info/help/help.htm#ipa_help.htm). Dashed lines represent indirect protein interactions while solid lines represent empirically measured direct interactions.
(1.41 MB TIF)

Figure S7 Chromosomal loci distribution of CMP-regulated gene products. A Human chromosomal location of genes controlling expression of specific CMP-regulated proteins. The panel depicts a schematic representation of the human chromosomal basepair (bp) gene loci (red + symbol) of proteins reliably regulated by chronic minimal peroxide (CMP) treatment. B histogram depicting the chromosomal frequency of genes encoding CMP hydrogen peroxide-regulated proteins in SH-SY5Y cells.
(0.91 MB TIF)

Table S1 Significantly regulated basal-state genes induced by implementation of the CMP paradigm in SH-SY5Y cells. Z ratios for each specific gene represent the mean of three independent analyzed cell lysates from CMP-treated cells compared to control-treated (CTR) counterparts.
(0.62 MB DOC)

Table S2 Latent semantic indexing textual interrogation of basal state CMP-dependent transcriptome alterations in SH-SY5Y cells. Latent semantic indexing (LSI) correlation scores as well as relevant gene symbols and genetic definition are denoted for each specific underlined interrogation term. Genes identified using LSI correlation that were upregulated compared to control by the CMP protocol are denoted in bold while those downregulated compared to control by CMP are denoted in italic.
(0.44 MB DOC)

Table S3 MeCh-significantly regulated genes after 2 hours of stimulation in the control state SH-SY5Y cells. Each significantly regulated gene is described via its accession number (ACCESSION), Gene Symbol (SYMBOL), Illumina array transcript designation (TRANSCRIPT). For each gene the z-ratio of expression compared to control vehicle-treated cells after 2 hours of ligand stimulation is displayed (CTL MeCh 2).
(0.94 MB DOC)

Table S4 MeCh-significantly regulated genes after 4 hours of stimulation in the control state SH-SY5Y cells. Each significantly regulated gene is described via its accession number (ACCESSION), Gene Symbol (SYMBOL), Illumina array transcript designation (TRANSCRIPT). For each gene the z-ratio of expression compared to un-treated cells after 4 hours of ligand stimulation is displayed (CTL MeCh 4).
(1.26 MB DOC)

Table S5 MeCh-significantly regulated genes after 8 hours of stimulation in the control state SH-SY5Y cells. Each significantly regulated gene is described via its accession number (ACCESSION), Gene Symbol (SYMBOL), Illumina array transcript designation (TRANSCRIPT). For each gene the z-ratio of expression compared to untreated cells after 8 hours of ligand stimulation is displayed (CTL MeCh 8).
(0.98 MB DOC)

Table S6 MeCh-significantly regulated genes after 2 hours of stimulation in the CMP state SH-SY5Y cells. Each significantly regulated gene is described via its accession number (ACCESSION), Gene Symbol (SYMBOL), Illumina array transcript designation (TRANSCRIPT). For each gene the z-ratio of expression compared to untreated cells after 2 hours of ligand stimulation is displayed (CMP MeCh 2).
(1.03 MB DOC)

Table S7 MeCh-significantly regulated genes after 4 hours of stimulation in the CMP state SH-SY5Y cells. Each significantly regulated gene is described via its accession number (ACCESSION), Gene Symbol (SYMBOL), Illumina array transcript designation (TRANSCRIPT). For each gene the z-ratio of expression compared to untreated cells after 4 hours of ligand stimulation is displayed (CMP MeCh 4).
(1.20 MB DOC)

Table S8 MeCh-significantly regulated genes after 8 hours of stimulation in the CMP state SH-SY5Y cells. Each significantly regulated gene is described via its accession number (ACCESSION), Gene Symbol (SYMBOL), Illumina array transcript designation (TRANSCRIPT). For each gene the z-ratio of expression compared to untreated cells after 8 hours of ligand stimulation is displayed (CMP MeCh 8).
(0.99 MB DOC)

Table S9 BDNF-significantly regulated genes after 2 hours of stimulation in the control state SH-SY5Y cells. Each significantly regulated gene is described via its accession number (ACCESSION), Gene Symbol (SYMBOL), Illumina array transcript designation (TRANSCRIPT). For each gene the z-ratio of expression compared to untreated cells after 2 hours of ligand stimulation is displayed (CTL BDNF 2).
(1.03 MB DOC)

Table S10 BDNF-significantly regulated genes after 4 hours of stimulation in the control state SH-SY5Y cells. Each significantly regulated gene is described via its accession number (ACCESSION), Gene Symbol (SYMBOL), Illumina array transcript designation (TRANSCRIPT). For each gene the z-ratio of expression compared to untreated cells after 4 hours of ligand stimulation is displayed (CTL BDNF 4).
(1.23 MB DOC)

Table S11 BDNF-significantly regulated genes after 8 hours of stimulation in the control state SH-SY5Y cells. Each significantly regulated gene is described via its accession number (ACCESSION), Gene Symbol (SYMBOL), Illumina array transcript designation (TRANSCRIPT). For each gene the z-ratio of expression compared to untreated cells after 8 hours of ligand stimulation is displayed (CTL BDNF 8).
(1.26 MB DOC)

Table S12 BDNF-significantly regulated genes after 2 hours of stimulation in the CMP state SH-SY5Y cells. Each significantly regulated gene is described via its accession number (ACCESSION), Gene Symbol (SYMBOL), Illumina array transcript designation (TRANSCRIPT). For each gene the z-ratio of expression compared to untreated cells after 2 hours of ligand stimulation is displayed (CMP BDNF 8).
(0.94 MB DOC)

Table S13 BDNF-significantly regulated genes after 4 hours of stimulation in the CMP state SH-SY5Y cells. Each significantly regulated gene is described via its accession number (ACCESSION), Gene Symbol (SYMBOL), Illumina array transcript designation (TRANSCRIPT). For each gene the z-ratio of expression compared to untreated cells after 4 hours of ligand stimulation is displayed (CMP BDNF 4).
(1.09 MB DOC)

Table S14 BDNF-significantly regulated genes after 8 hours of stimulation in the CMP state SH-SY5Y cells. Each significantly regulated gene is described via its accession number (ACCESSION), Gene Symbol (SYMBOL), Illumina array transcript designation (TRANSCRIPT). For each gene the z-ratio of expression compared to untreated cells after 8 hours of ligand stimulation is displayed (CMP BDNF 8).
(1.15 MB DOC)

Table S15 Common 2 hour MeCh-regulated gene series. MeCh-regulated genes that were significantly elevated or reduced compared to the respective unstimulated control cells in both the control (untreated: MeCh-2h-Control vs. Control-Control) and CMP state SH-SY5Y cells (MeCh-2h-CMP vs. Control-CMP). The series number refers to the simplistic relationships between the degree of regulation of the respective genes and the cellular state (untreated or CMP). Series 1 (both upregulated) - MeCh-2h-CMP vs. Control-CMP > MeCh-2h-Control vs. Control-Control; Series 2 (both upregulated) - MeCh-2h-Control vs. Control-Control > MeCh-2h-CMP vs. Control-CMP; Series 3 (both downregulated) - MeCh-2h-Control vs. Control-Control > MeCh-2h-CMP vs. Control-CMP; Series 4 (both downregulated) - MeCh-2h-CMP vs. Control-CMP > MeCh-2h-Control vs. Control-Control; Series 5 (downregulated in control, upregulated in CMP); Series 6 (upregulated in control, down regulated in CMP).
(0.42 MB DOC)

Table S16 Common 4 hour MeCh-regulated gene series. MeCh-regulated genes that were significantly elevated or reduced compared to the respective unstimulated control cells in both the control (untreated: MeCh-4h-Control vs. Control-Control) and CMP state SH-SY5Y cells (MeCh-4h-CMP vs. Control-CMP). The series number refers to the simplistic relationships between the degree of regulation of the respective genes and the cellular state (untreated or CMP). Series 1 (both upregulated) - MeCh-4h-CMP vs. Control-CMP > MeCh-4h-Control vs. Control-Control; Series 2 (both upregulated) - MeCh-4h-Control vs. Control-Control > MeCh-4h-CMP vs. Control-CMP; Series 3 (both downregulated) - MeCh-4h-Control vs. Control-Control > MeCh-4h-CMP vs. Control-CMP; Series 4 (both downregulated) - MeCh-4h-CMP vs. Control-CMP > MeCh-4h-Control vs. Control-Control; Series 5 (downregulated in control, upregulated in CMP); Series 6 (upregulated in control, down regulated in CMP).
(0.57 MB DOC)

Table S17 Common 8 hour MeCh-regulated gene series. MeCh-regulated genes that were significantly elevated or reduced compared to the respective unstimulated control cells in both the control (untreated: MeCh-8h-Control vs. Control-Control) and CMP state SH-SY5Y cells (MeCh-8h-CMP vs. Control-CMP). The series number refers to the simplistic relationships between the degree of regulation of the respective genes and the cellular state (untreated or CMP). Series 1 (both upregulated) - MeCh-8h-CMP vs. Control-CMP > MeCh-8h-Control vs. Control-Control; Series 2 (both upregulated) - MeCh-8h-Control vs. Control-Control > MeCh-8h-CMP vs. Control-CMP; Series 3 (both downregulated) - MeCh-8h-Control vs. Control-Control > MeCh-8h-CMP vs. Control-CMP; Series 4 (both downregulated) - MeCh-8h-CMP vs. Control-CMP > MeCh-8h-Control vs. Control-Control; Series 5 (downregulated in control, upregulated in CMP); Series 6 (upregulated in control, down regulated in CMP).
(0.57 MB DOC)

Table S18 Common 2 hour BDNF-regulated gene series. BDNF-regulated genes that were significantly elevated or reduced compared to the respective unstimulated control cells in both the control (untreated: BDNF-2h-Control vs. Control-Control) and CMP state SH-SY5Y cells (BDNF-2h-CMP vs. Control-CMP). The series number refers to the simplistic relationships between the degree of regulation of the respective genes and the cellular state (untreated or CMP). Series 1 (both upregulated) - BDNF-2h-CMP vs. Control-CMP > BDNF-2h-Control vs. Control-Control; Series 2 (both upregulated) - BDNF-2h-Control vs. Control-Control > BDNF-2h-CMP vs. Control-CMP; Series 3 (both downregulated) - BDNF-2h-Control vs. Control-Control > BDNF-2h-CMP vs. Control-CMP; Series 4 (both downregulated) - BDNF-2h-CMP vs. Control-CMP > BDNF-2h-Control vs. Control-Control; Series 5 (downregulated in control, upregulated in CMP); Series 6 (upregulated in control, down regulated in CMP).
(0.46 MB DOC)

Table S19 Common 4 hour BDNF-regulated gene series. BDNF-regulated genes that were significantly elevated or reduced compared to the respective unstimulated control cells in both the control (untreated: BDNF-4h-Control vs. Control-Control) and CMP state SH-SY5Y cells (BDNF-4h-CMP vs. Control-CMP). The series number refers to the simplistic relationships between the degree of regulation of the respective genes and the cellular state (untreated or CMP). Series 1 (both upregulated) - BDNF-4h-CMP vs. Control-CMP > BDNF-4h-Control vs. Control-Control; Series 2 (both upregulated) - BDNF-4h-Control vs. Control-Control > BDNF-4h-CMP vs. Control-CMP; Series 3 (both downregulated) - BDNF-4h-Control vs. Control-Control > BDNF-4h-CMP vs. Control-CMP; Series 4 (both downregulated) - BDNF-4h-CMP vs. Control-CMP > BDNF-4h-Control vs. Control-Control; Series 5 (downregulated in control, upregulated in CMP); Series 6 (upregulated in control, down regulated in CMP).
(0.46 MB DOC)

Table S20 Common 8 hour BDNF-regulated gene series. BDNF-regulated genes that were significantly elevated or reduced compared to the respective unstimulated control cells in both the control (untreated: BDNF-8h-Control vs. Control-Control) and CMP state SH-SY5Y cells (BDNF-8h-CMP vs. Control-CMP). The series number refers to the simplistic relationships between the degree of regulation of the respective genes and the cellular state (untreated or CMP). Series 1 (both upregulated) - BDNF-8h-CMP vs. Control-CMP > BDNF-8h-Control vs. Control-Control; Series 2 (both upregulated) - BDNF-8h-Control vs. Control-Control > BDNF-8h-CMP vs. Control-CMP; Series 3 (both downregulated) - BDNF-8h-Control vs. Control-Control > BDNF-8h-CMP vs. Control-CMP; Series 4 (both downregulated) - BDNF-8h-CMP vs. Control-CMP > BDNF-8h-Control vs. Control-Control; Series 5 (downregulated in control, upregulated in CMP); Series 6 (upregulated in control, down regulated in CMP).
(0.46 MB DOC)

Table S21 Regulated proteins on Panorama cell signaling antibody array following CMP treatment. The expression ratio between that in CMP versus control (CTL) conditions was measured from multiple (n = 4) array experiments and is expressed as a mean ratio with a calculated standard error of that meam (SEM).
(0.10 MB DOC)

Table S22 Network analysis of antibody signaling array CMP-significantly regulated proteins. Specific correlated networks were generated using an un-biased algorithm using Ingenuity Pathway Analysis (IPA). The highest network scores indicate the probabilistic likelihood of the specific protein network functionally dominating the dataset. Pre-determined functional networks are significantly populated with molecules (proteins) from the input experimental dataset. The network score is a function of the number and rarity of the specific experimental Focus molecules that populate the specific network. Predicted functions are derived from proprietary IPA software.
(0.27 MB DOC)
