# Supplementary figures and images for: Minimal Peroxide Exposure of Neuronal Cells Induces Multifaceted Adaptive Responses
Source: PLoS One. 2010 Dec 17;5(12):e14352. doi: 10.1371/journal.pone.0014352 (PMC3003681; doi:10.1371/journal.pone.0014352)

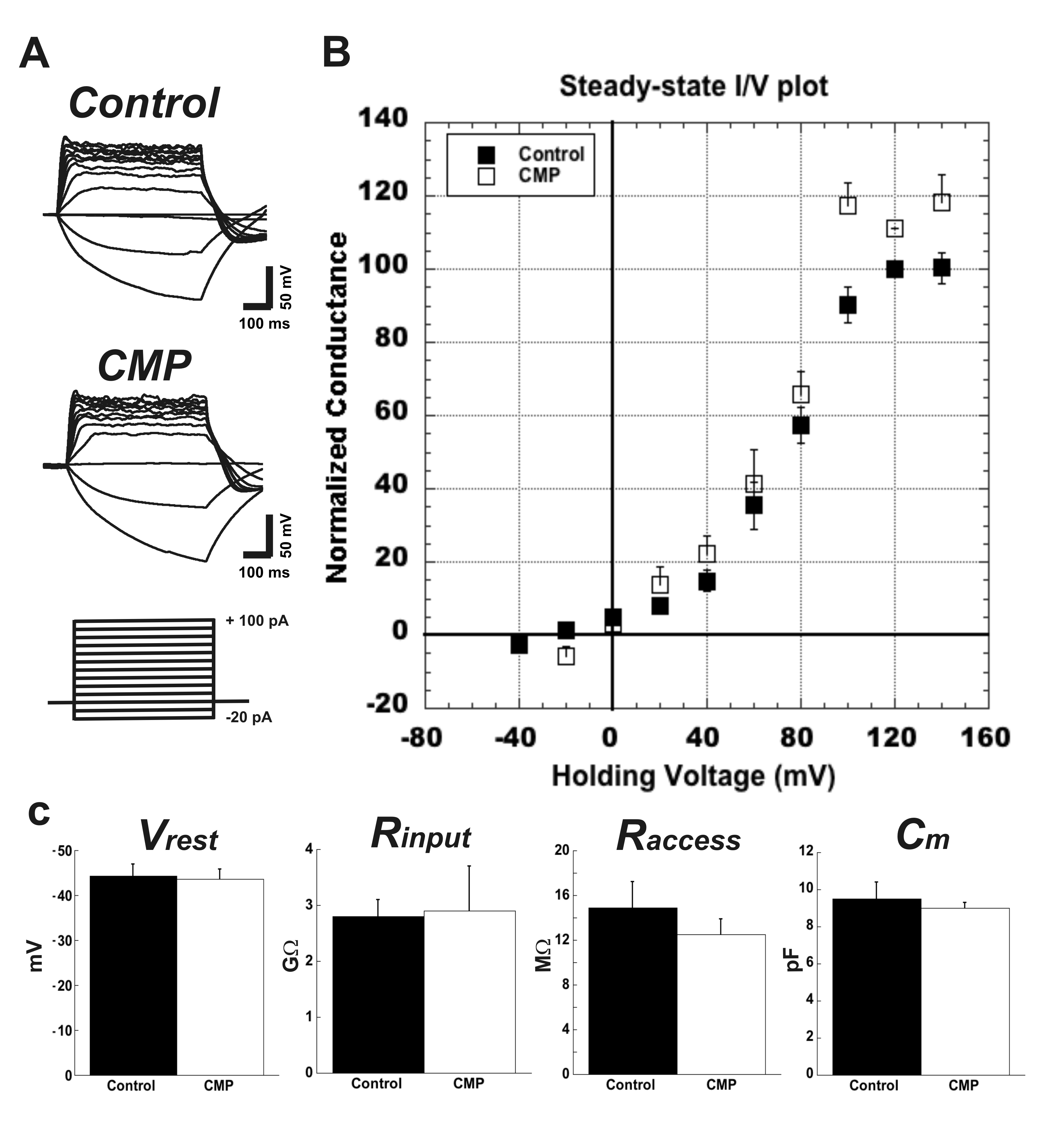

Supplement: Figure S1 — Chronic minimal peroxide treatment does not significantly affect membrane electrophysiological properties. A Current clamp family recordings from control- or CMP-treated SH-SY5Y cells subjected to injection of steps from 20pA to +100pA. B Steady-state current/voltage relationships gained in voltage-clamp mode for control or CMP SH-SY5Y cells subjected to voltage steps from −40 mV to +140 mV. C Classical cell membrane parameters assessed for both control and CMP cells included resting membrane potential (Vrest), input resistance (Rinput), access resistance (Raccess) and membrane capacitance (Cm). (0.84 MB TIF) [file pone.0014352.s001.tif]

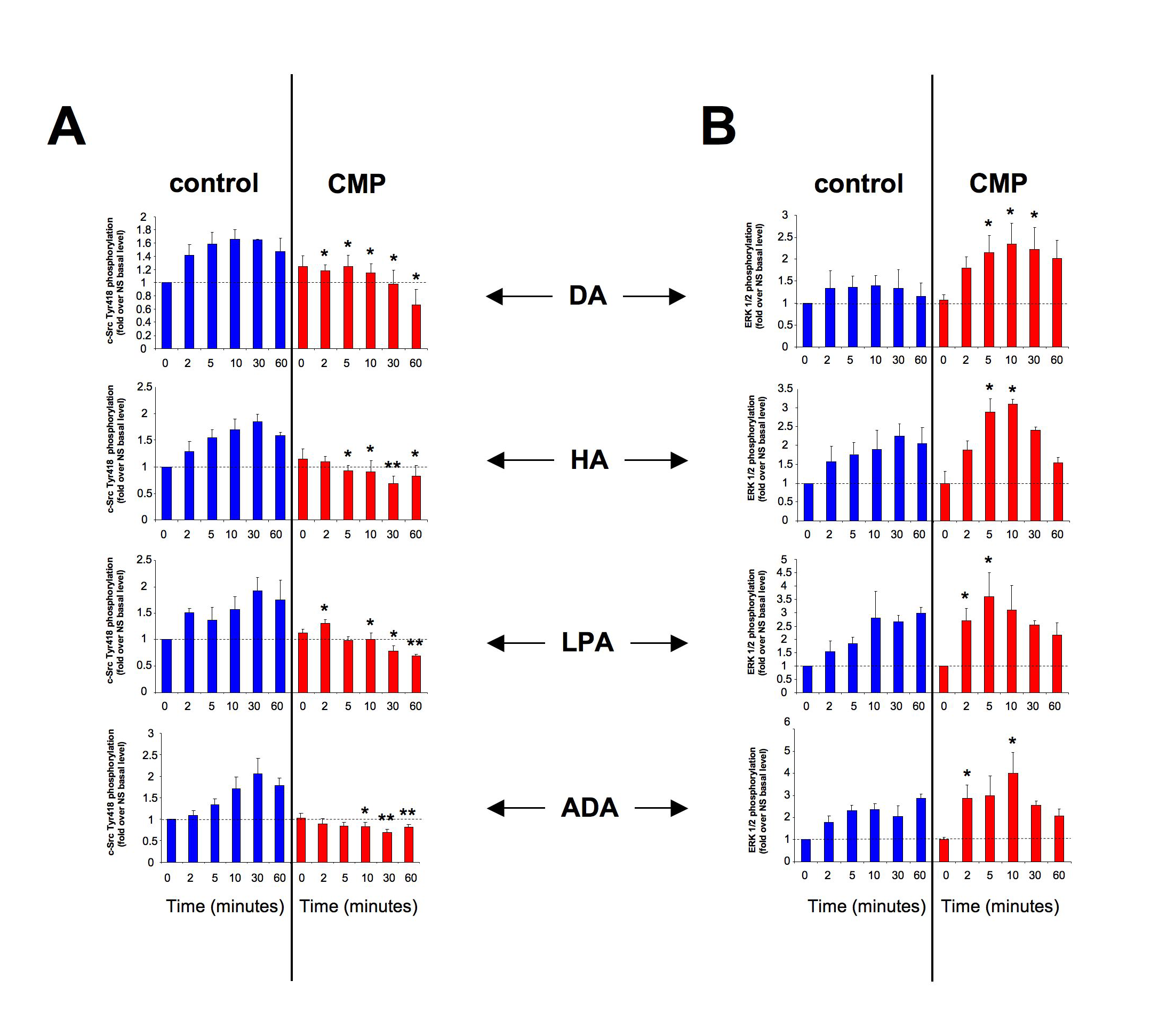

Supplement: Figure S2 — CMP affects multiple receptor systems with respect to c-Src and extracellular signal-regulated kinase (ERK1/2) activation. A. Dopamine- (DA, 10 nM), histamine- (HA, 10 nM), lysophospahtidic acid- (LPA, 10 nM) and anandamide- (ADA, 10 nM) mediated c-Src tyrosine-418 autophosphorylation in control (blue bars) or CMP-treated (red bars) cells for the respective times specified. B. Dopamine- (DA, 10 nM), histamine- (HA, 10 nM), lysophospahtidic acid- (LPA, 10 nM) and anandamide- (ADA, 10 nM) mediated ERK1/2 activation in control (blue bars) or CMP-treated (red bars) cells for the respective times specified. Statistical significance is indicated for changes in kinase activity in the CMP state relative to their time-matched control in vehicle-treated (control) cells. Statistical significance was measured using a Student's t-test (GraphPad Prism v.3): * - p<0.05; ** - p<0.01. (1.81 MB TIF) [file pone.0014352.s002.tif]

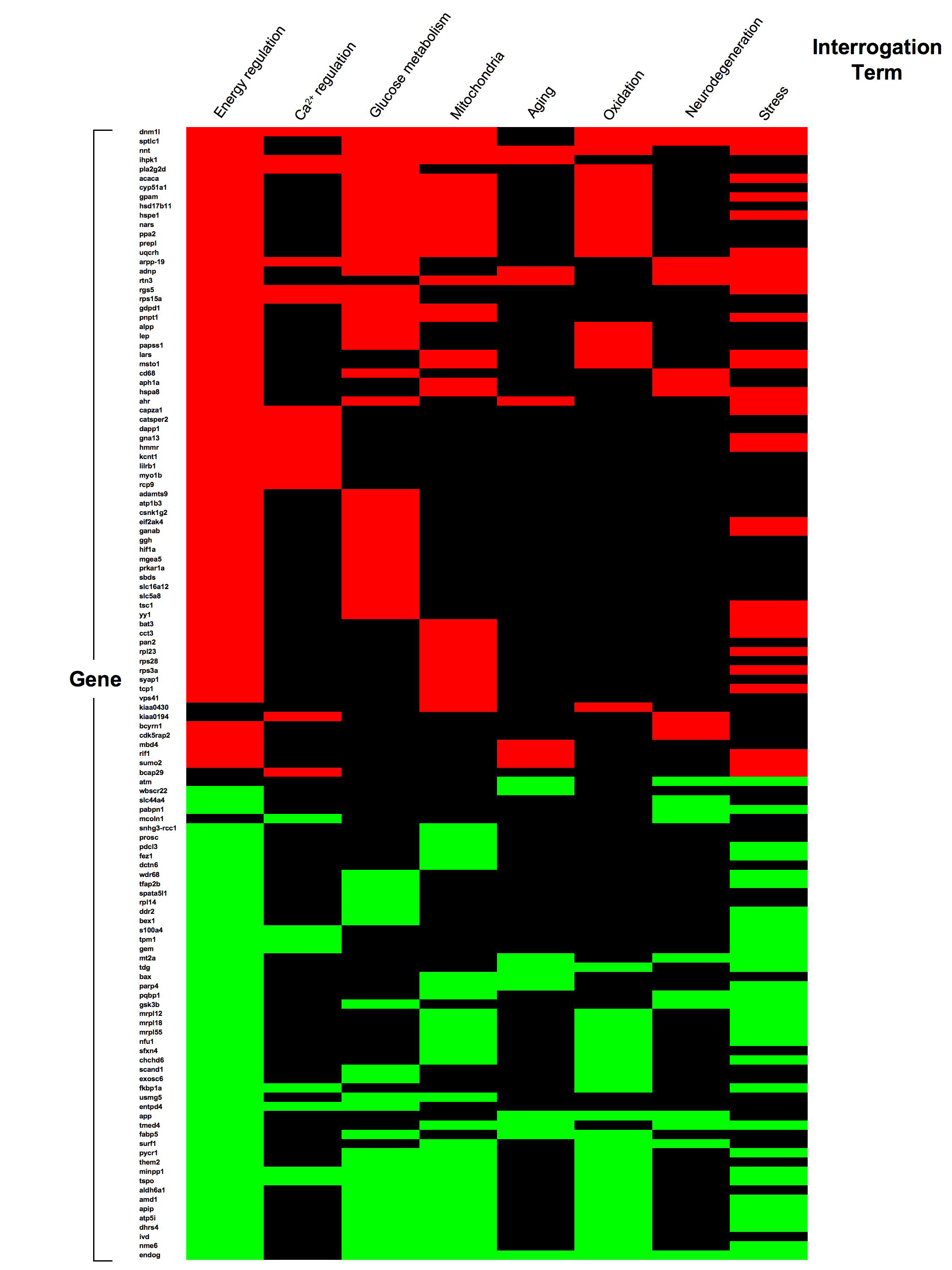

Supplement: Figure S3 — Textual correlation of multiple CMP-regulated factors in SH-SY5Y cells. A. Heatmap representation of CMP-significantly-regulated genes (gene symbol identities list on the left of the heatmap) and their multiple correlation with various Latent Semantic Indexing interrogation terms (Energy regulation, Ca2+ regulation, Glucose metabolism, Mitochondria, Aging, Oxidation, Neurodegeneration, Stress). A red block indicates that the specific gene correlates implicitly (≥0.1 correlation score) with the interrogation term and was upregulated in response to CMP, while green indicates a downregulated implicitly correlated (≥0.1 correlation score) gene. A black block represents a lack of correlation between the specific gene and interrogation term. (1.57 MB TIF) [file pone.0014352.s003.tif]

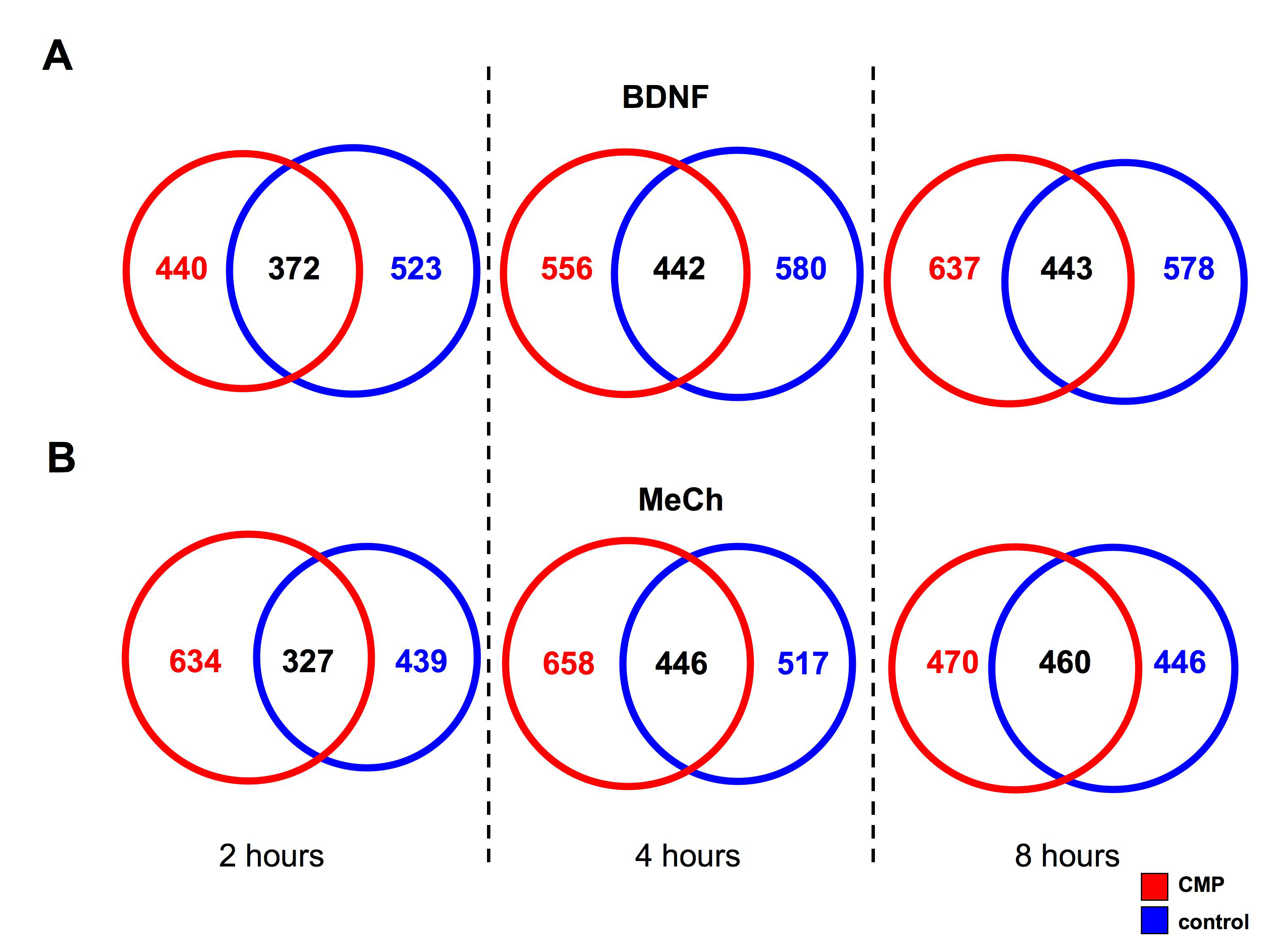

Supplement: Figure S4 — Venn diagram analysis of BDNF- or MeCh-induced transcriptional regulation. A Venn diagram analysis of global significantly-regulated genes with either 2, 4, or 8 hours of BDNF stimulation (CMP-red circle, Control-blue circle). B Venn diagram analysis of global significantly-regulated genes with either 2, 4, or 8 hours of MeCh stimulation (CMP-red circle, Control-blue circle). Genes commonly regulated in control and CMP conditions are indicated by black numbers. (2.36 MB TIF) [file pone.0014352.s004.tif]

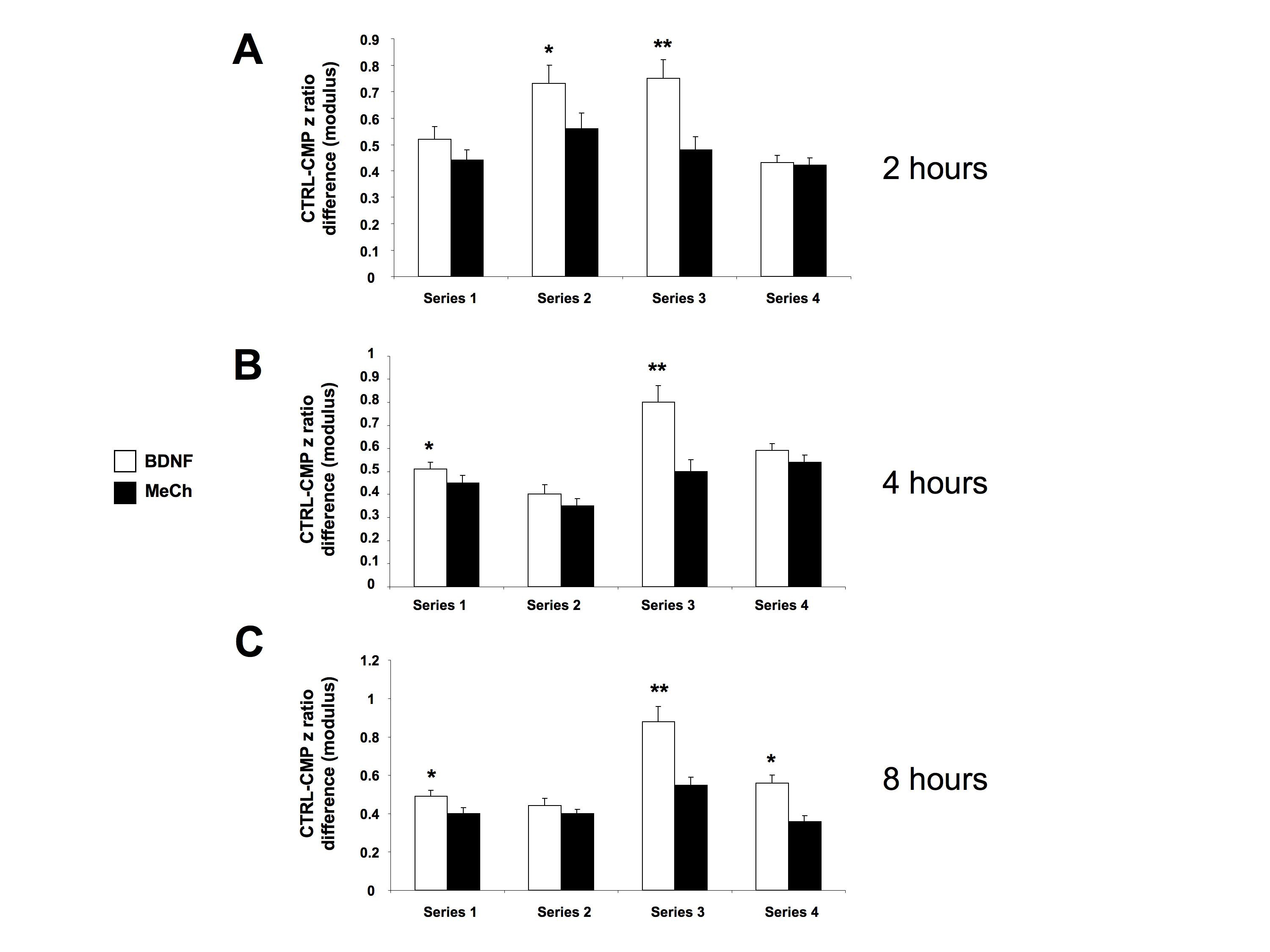

Supplement: Figure S5 — Response series-specific moduli of z ratio differences between commonly CMP-Control regulated genes. A. Modulus scores (independent of polarity of z ratio difference) for control (CTRL) and CMP differences in z ratio after 2 hours of stimulation with either MeCh or BDNF demonstrate a z ratio difference. Similar series-specific z ratio difference modulus scores for 4 (B) or 8 (C) hours of stimulation are depicted below. For both BDNF or MeCh the gene regulation series are as follows: series 1 = upregulated in CMP and control with z ratio CMP>CTRL; series 2 = upregulated in CMP/control with z ratio CTRL>CMP; series 3 = downregulated in CMP/control with z ratio CMP<CTRL; series 4 = downregulated in CMP/control with z ratio CTRL<CMP. Values in each histogram are representations of mean ± SEM (n = 3). Statistical significance was assessed using a Student's t-test. * p<0.05, ** p<0.01. (1.11 MB TIF) [file pone.0014352.s005.tif]

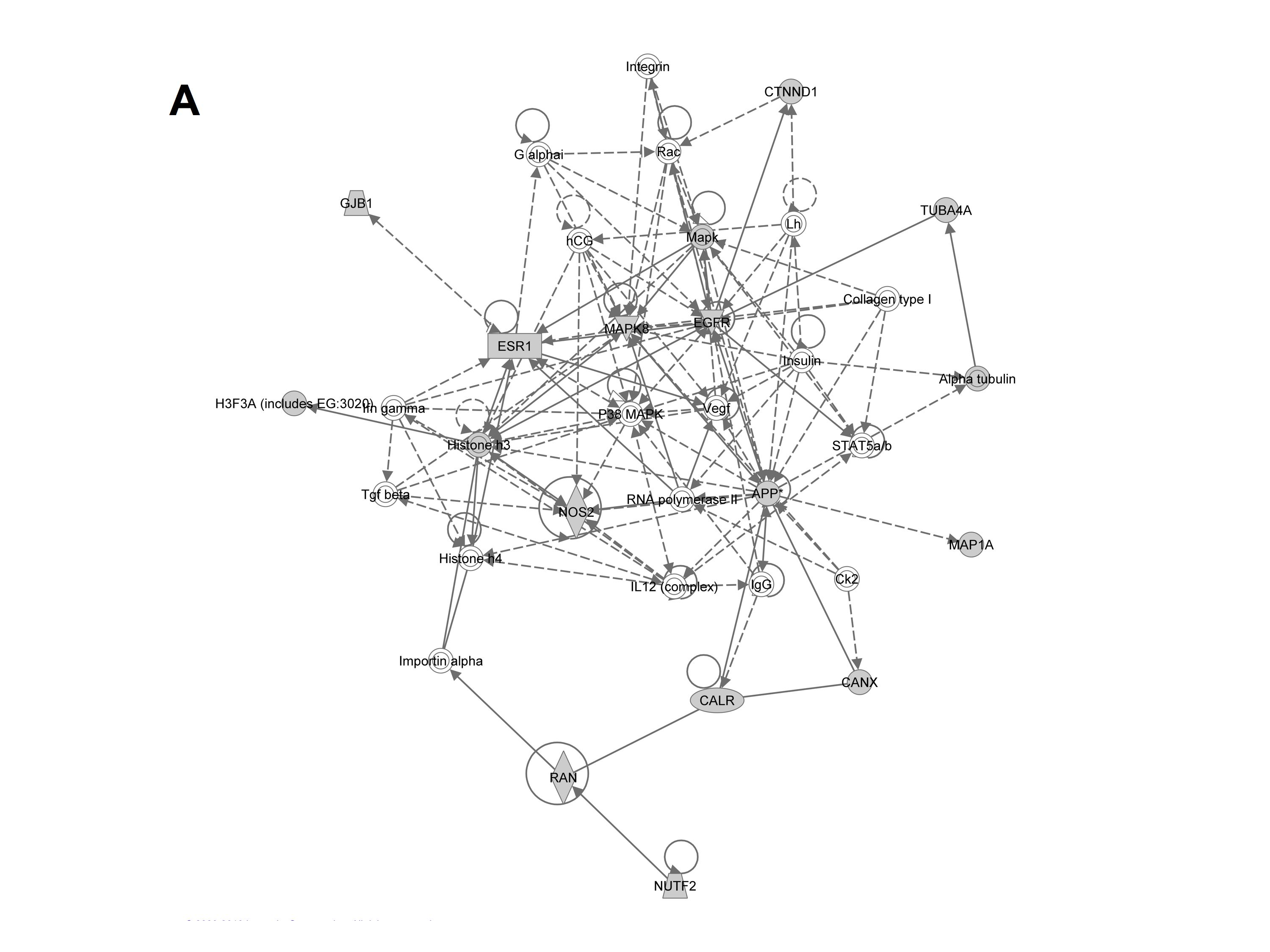

Supplement: Figure S6 — Signal transduction network analysis of CMP-regulated factors in SH-SY5Y cells. Using an un-biased signaling network-generating algorithm (Ingenuity Pathway Analysis) the most likely protein-protein interaction cluster was created from the significantly altered signaling proteins in response to the CMP protocol. The figure depicts the highest scoring functional interaction network created from the CMP-controlled signaling factors. Proteins denoted in capitalized text were directly derived from the input protein set. A full description of the nature of interactions based on the connecting lines can be found at the following webpage linked to the IPA analysis module (https://analysis.ingenuity.com/pa/info/help/help.htm#ipa_help.htm). Dashed lines represent indirect protein interactions while solid lines represent empirically measured direct interactions. (1.41 MB TIF) [file pone.0014352.s006.tif]

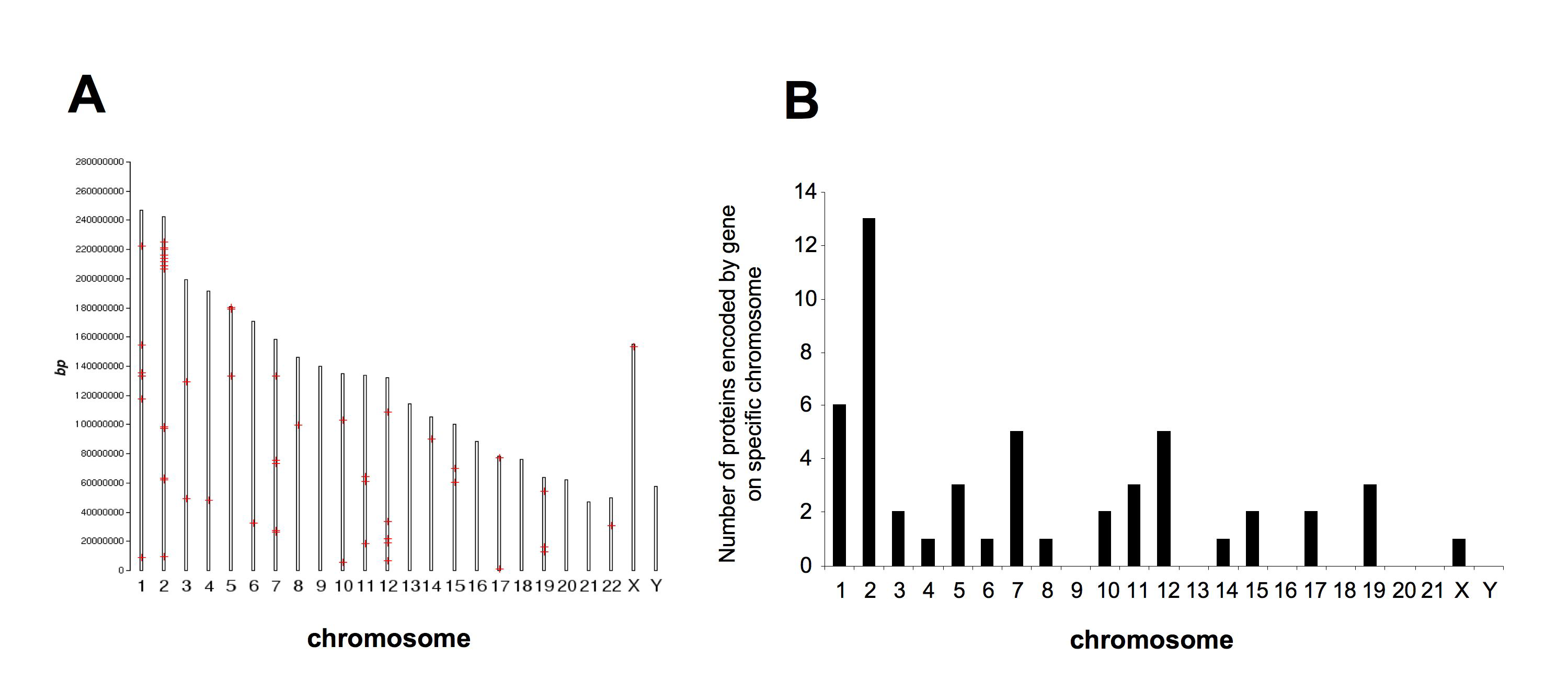

Supplement: Figure S7 — Chromosomal loci distribution of CMP-regulated gene products. A Human chromosomal location of genes controlling expression of specific CMP-regulated proteins. The panel depicts a schematic representation of the human chromosomal basepair (bp) gene loci (red + symbol) of proteins reliably regulated by chronic minimal peroxide (CMP) treatment. B histogram depicting the chromosomal frequency of genes encoding CMP hydrogen peroxide-regulated proteins in SH-SY5Y cells. (0.91 MB TIF) [file pone.0014352.s007.tif]
