# Supplementary material for: Minimal Peroxide Exposure of Neuronal Cells Induces Multifaceted Adaptive Responses
Source: PLoS One. 2010 Dec 17;5(12):e14352. doi: 10.1371/journal.pone.0014352 (PMC3003681; doi:10.1371/journal.pone.0014352)
Supplement: Table S1 — Significantly regulated basal-state genes induced by implementation of the CMP paradigm in SH-SY5Y cells. Z ratios for each specific gene represent the mean of three independent analyzed cell lysates from CMP-treated cells compared to control-treated (CTR) counterparts. (0.62 MB DOC) [file pone.0014352.s008.doc]

**Table S1. Significantly regulated basal-state genes induced by implementation of the CMP paradigm in SH-SY5Y cells**. Z ratios for each specific gene represent the mean of three independent analyzed cell lysates from CMP-treated cells compared to control-treated (CTR) counterparts.

| **ACCESSION** | **DEFINITION** | **SYMBOL** | **(zratio) CMPvsCTR** |
| --- | --- | --- | --- |
| XM_935588.1 | similar to ribosomal protein S3a | LOC641848 | 5.259917879 |
| NM_001003.2 | ribosomal protein, large, P1 | RPLP1 | 5.101129611 |
| NM_006135.1 | capping protein (actin filament) muscle Z-line, alpha 1 | CAPZA1 | 4.882072526 |
| NM_001010915.1 | protein tyrosine phosphatase-like A domain containing 2 | PTPLAD2 | 4.834199014 |
| NR_002315.1 | H3 histone, family 3A pseudogene | LOC440926 | 4.811923709 |
| NM_013361.3 | zinc finger protein 223 | ZNF223 | 4.5840888 |
| XR_017492.1 | similar to tropomyosin 3 isoform 2 | LOC644330 | 4.545144838 |
| NM_017821.3 | rhomboid, veinlet-like 2 (Drosophila) | RHBDL2 | 4.469647167 |
| NM_199436.1 | spastin, transcript variant 2 | SPAST | 4.350773382 |
| NM_178324.1 | serine palmitoyltransferase, long chain base subunit 1 | SPTLC1 | 4.334426039 |
| XM_929199.1 | hypothetical protein LOC644250 | LOC644250 | 4.332893173 |
| NM_018561.3 | ubiquitin specific peptidase 49 | USP49 | 4.288163935 |
| XR_016048.1 | hypothetical protein MGC40489 | MGC40489 | 4.277875045 |
| NM_172014.1 | tumor necrosis factor (ligand) superfamily, member 14 | TNFSF14 | 4.18105651 |
| XR_015514.1 | similar to Heterogeneous nuclear ribonucleoprotein A1 | LOC730746 | 4.152073597 |
| NM_173518.2 | chromosome 8 open reading frame 45 | C8orf45 | 4.137101173 |
| NM_173666.1 | DTW domain containing 2 | DTWD2 | 4.080189827 |
| NM_003666.2 | basic leucine zipper nuclear factor 1 (JEM-1) | BLZF1 | 4.080061198 |
| NM_012322.1 | LSM5 homolog, U6 small nuclear RNA associated (S. cerevisiae) | LSM5 | 4.070484993 |
| NM_004768.2 | splicing factor, arginine/serine-rich 11 | SFRS11 | 4.048227752 |
| NM_001099285.1 | prothymosin, alpha | PTMA | 4.021677488 |
| NM_012400.2 | phospholipase A2, group IID | PLA2G2D | 3.995925543 |
| NM_001251.2 | CD68 molecule | CD68 | 3.894415293 |
| NM_002266.2 | karyopherin alpha 2 (RAG cohort 1, importin alpha 1) | KPNA2 | 3.879900181 |
| NM_003082.2 | small nuclear RNA activating complex, polypeptide 1, 43kDa | SNAPC1 | 3.873392947 |
| NM_020704.1 | family with sequence similarity 40, member B | FAM40B | 3.861660439 |
| NR_002201.1 | ferritin, heavy polypeptide-like 3 | FTHL3 | 3.861510739 |
| NM_014363.3 | spastic ataxia of Charlevoix-Saguenay (sacsin) | SACS | 3.837203922 |
| NM_025152.1 | nucleotide binding protein-like | NUBPL | 3.825404806 |
| NM_001621.2 | aryl hydrocarbon receptor | AHR | 3.812876584 |
| NM_080386.1 | tubulin, alpha 3d | TUBA3D | 3.808436696 |
| XM_941684.2 | similar to 40S ribosomal protein S4, X isoform | LOC220433 | 3.780246322 |
| NM_145913.2 | solute carrier family 5 (iodide transporter), member 8 | SLC5A8 | 3.77870632 |
| NR_003040.1 | ribosomal protein L23a pseudogene | LOC649946 | 3.765817646 |
| XM_937113.2 | similar to ribosomal protein L5, transcript variant 1 | LOC647436 | 3.739967407 |
| NM_019119.3 | protocadherin beta 9 | PCDHB9 | 3.692564545 |
| NM_000572.2 | interleukin 10 | IL10 | 3.65986415 |
| NM_014478.4 | calcitonin gene-related peptide-receptor component protein | RCP9 | 3.615716186 |
| NM_014395.1 | dual adaptor of phosphotyrosine and 3-phosphoinositides | DAPP1 | 3.612729265 |
| XM_938089.2 | similar to large subunit ribosomal protein L36a | LOC643007 | 3.611817175 |
| XR_018327.1 | similar to protein phosphatase 1 regulatory subunit 14B | LOC648343 | 3.606811868 |
| NM_176811.2 | NLR family, pyrin domain containing 8 | NLRP8 | 3.59252146 |
| XM_930995.1 | similar to RAN-binding protein 2-like 1 isoform 2, transcript variant 10 | LOC653086 | 3.587138614 |
| NM_000383.1 | autoimmune regulator (autoimmune polyendocrinopathy candidiasis ectodermal dystrophy) | AIRE | 3.554794907 |
| XM_934113.1 | similar to Ran-binding protein 2 | LOC653489 | 3.551004404 |
| NR_002204.1 | ferritin, heavy polypeptide-like 11 | FTHL11 | 3.54315201 |
| NR_002200.1 | ferritin, heavy polypeptide-like 2 | FTHL2 | 3.527890997 |
| NM_172249.1 | colony stimulating factor 2 receptor, alpha, low-affinity (granulocyte-macrophage) | CSF2RA | 3.522036329 |
| NM_001632.3 | alkaline phosphatase, placental (Regan isozyme) | ALPP | 3.506520584 |
| NM_006731.2 | fukutin | FKTN | 3.488498629 |
| XM_292963.6 | similar to peptidylprolyl isomerase A isoform 1 | LOC643997 | 3.481418659 |
| NM_001013703.2 | eukaryotic translation initiation factor 2 alpha kinase 4 | EIF2AK4 | 3.446907892 |
| NM_138477.2 | congenital dyserythropoietic anemia, type I | CDAN1 | 3.435898765 |
| XM_938297.1 | similar to peptidylprolyl isomerase A isoform 1 | LOC402644 | 3.423373714 |
| XM_942501.1 | colony stimulating factor 2 receptor, alpha, low-affinity (granulocyte-macrophage) | CSF2RA | 3.420634567 |
| NM_018097.1 | centrosomal protein 27kDa | CEP27 | 3.384430413 |
| XM_944915.1 | protein tyrosine phosphatase type IVA, member 2, transcript variant 9 | PTP4A2 | 3.373649312 |
| XM_940333.2 | similar to large subunit ribosomal protein L36a | LOC651202 | 3.365917213 |
| NM_005721.3 | ARP3 actin-related protein 3 homolog (yeast) | ACTR3 | 3.345833903 |
| NM_001412.3 | eukaryotic translation initiation factor 1A, X-linked | EIF1AX | 3.338952711 |
| XM_936731.1 | similar to Translationally-controlled tumor protein | LOC647673 | 3.319537453 |
| NM_177965.2 | chromosome 8 open reading frame 37 | C8orf37 | 3.313067303 |
| XM_926231.1 | prostate-specific P704P | P704P | 3.295041476 |
| NM_001029862.1 | ankyrin repeat domain 30B | ANKRD30B | 3.273622859 |
| NM_006265.1 | RAD21 homolog (S. pombe) | RAD21 | 3.254049704 |
| NM_005128.2 | dopey family member 2 | DOPEY2 | 3.242299561 |
| NM_031943.1 | IFP38 | IFP38 | 3.229886876 |
| XM_375152.3 | similar to Golgi autoantigen, golgin subfamily A member 2 (Golgi matrix protein GM130), transcript variant 1 | LOC400304 | 3.224594486 |
| NM_006572.3 | guanine nucleotide binding protein (G protein), alpha 13 | GNA13 | 3.205033901 |
| NM_000230.1 | leptin (obesity homolog, mouse) | LEP | 3.196605572 |
| NR_002205.1 | ferritin, heavy polypeptide-like 12 | FTHL12 | 3.1385145 |
| NM_178231.1 | amyotrophic lateral sclerosis 2 (juvenile) chromosome region, candidate 14 | ALS2CR14 | 3.134722658 |
| NR_002204.1 | ferritin, heavy polypeptide-like 11 | FTHL11 | 3.126041649 |
| NM_013412.1 | RAB, member of RAS oncogene family-like 2A | RABL2A | 3.113121714 |
| NM_016038.2 | Shwachman-Bodian-Diamond syndrome | SBDS | 3.109013821 |
| NM_183422.1 | TSC22 domain family, member 1 | TSC22D1 | 3.103505274 |
| NM_033196.2 | zinc finger protein 682 | ZNF682 | 3.10345168 |
| NM_022483.3 | chromosome 5 open reading frame 28 | C5orf28 | 3.092432923 |
| NM_005443.4 | 3'-phosphoadenosine 5'-phosphosulfate synthase 1 | PAPSS1 | 3.088846023 |
| XM_944716.1 | hypothetical gene supported by BC042042 | LOC440704 | 3.083445462 |
| NM_133459.1 | collagen and calcium binding EGF domains 1 | CCBE1 | 3.079299937 |
| CR612552 | full-length cDNA clone XCL0BB001ZD04 of Neuroblastoma of (human) | XCL0BB001ZD04 | 3.079020724 |
| XM_939697.1 | chromosome 9 open reading frame 130 | C9orf130 | 3.045151797 |
| NM_152730.4 | chromosome 6 open reading frame 170 | C6orf170 | 3.044848201 |
| NM_003677.3 | density-regulated protein | DENR | 3.043521451 |
| NM_002291.1 | laminin, beta 1 | LAMB1 | 3.037942789 |
| XM_945045.1 | similar to Tubulin beta-4q chain, transcript variant 2 | LOC649679 | 3.030372314 |
| XM_941195.2 | similar to ribosomal protein L21 | LOC388621 | 3.018577978 |
| NM_032794.1 | solute carrier family 44, member 4 | SLC44A4 | 3.010378393 |
| XM_944104.2 | hypothetical LOC653232, transcript variant 4 | LOC653232 | 3.009388035 |
| NM_144736.3 | hypothetical protein PRO1853 | PRO1853 | 3.00576365 |
| NM_021218.1 | chromosome 9 open reading frame 80 | C9orf80 | 2.984041278 |
| NM_032753.2 | retina and anterior neural fold homeobox like 1 | RAXL1 | 2.975946554 |
| NM_000786.2 | cytochrome P450, family 51, subfamily A, polypeptide 1 | CYP51A1 | 2.968752463 |
| NM_001080484.1 | KIAA1751 | KIAA1751 | 2.951655225 |
| NM_014060.1 | malignant T cell amplified sequence 1 | MCTS1 | 2.950909334 |
| NM_032485.4 | minichromosome maintenance complex component 8 | MCM8 | 2.940820536 |
| NM_001562.2 | interleukin 18 (interferon-gamma-inducing factor) | IL18 | 2.925944428 |
| NM_172097.1 | cation channel, sperm associated 2 | CATSPER2 | 2.891814464 |
| NM_001039755.1 | hypothetical protein LOC641737 | FLJ44124 | 2.889634602 |
| NM_025084.1 | hypothetical protein FLJ22795 | FLJ22795 | 2.889178392 |
| NM_152411.2 | zinc finger protein 786 | ZNF786 | 2.888003708 |
| NM_052879.3 | La ribonucleoprotein domain family, member 4 | LARP4 | 2.884770799 |
| NM_014897.1 | zinc finger protein 652 | ZNF652 | 2.871436684 |
| NM_033412.1 | mitochondrial carrier triple repeat 1 | MCART1 | 2.854008979 |
| XM_935770.1 | similar to cell division cycle 42 | LOC641992 | 2.846257533 |
| XR_015809.1 | similar to 40S ribosomal protein S7 (S8) | LOC728973 | 2.835926189 |
| NM_001080973.1 | interleukin 17 receptor D | IL17RD | 2.834332543 |
| NM_017833.2 | chromosome 21 open reading frame 55 | C21orf55 | 2.826541172 |
| NM_001079842.1 | OCIA domain containing 1 | OCIAD1 | 2.810733682 |
| NR_002203.1 | ferritin, heavy polypeptide-like 8 | FTHL8 | 2.805394501 |
| NM_005010.3 | neuronal cell adhesion molecule | NRCAM | 2.794981395 |
| NM_138687.1 | phosphatidylinositol-4-phosphate 5-kinase, type II, beta | PIP5K2B | 2.791788166 |
| NM_207418.2 | gastric cancer up-regulated-2 | GCUD2 | 2.791326408 |
| XM_937928.1 | similar to H3 histone, family 3B | LOC347376 | 2.788803999 |
| NM_001089.1 | ATP-binding cassette, sub-family A (ABC1), member 3 | ABCA3 | 2.788225223 |
| NM_001031.4 | ribosomal protein S28 | RPS28 | 2.786174264 |
| NM_001008735.1 | high-mobility group (nonhistone chromosomal) protein 1-like 1 | HMG1L1 | 2.782516275 |
| NM_004788.2 | ubiquitination factor E4A (UFD2 homolog, yeast) | UBE4A | 2.774923044 |
| NM_001034996.1 | ribosomal protein L14 | RPL14 | 2.768571946 |
| NM_003617.2 | regulator of G-protein signaling 5 | RGS5 | 2.765666186 |
| NM_031314.1 | heterogeneous nuclear ribonucleoprotein C (C1/C2) | HNRPC | 2.745848552 |
| NM_002129.2 | high-mobility group box 2 | HMGB2 | 2.741680132 |
| NM_006107.2 | cisplatin resistance-associated overexpressed protein | CROP | 2.729100057 |
| NM_022173.1 | TIA1 cytotoxic granule-associated RNA binding protein | TIA1 | 2.722774669 |
| NM_033109.2 | polyribonucleotide nucleotidyltransferase 1 | PNPT1 | 2.720470026 |
| NR_003664.1 | Williams Beuren syndrome chromosome region 19 pseudogene | LOC389517 | 2.716063064 |
| XM_938755.2 | similar to ribosomal protein L31 | LOC653773 | 2.706700578 |
| NM_003878.1 | gamma-glutamyl hydrolase (conjugase, folylpolygammaglutamyl hydrolase) | GGH | 2.699507187 |
| NM_014396.3 | vacuolar protein sorting 41 homolog (S. cerevisiae) | VPS41 | 2.688441465 |
| NM_006004.1 | ubiquinol-cytochrome c reductase hinge protein | UQCRH | 2.677368236 |
| NM_018204.2 | cytoskeleton associated protein 2 | CKAP2 | 2.674675109 |
| NM_025189.2 | zinc finger protein 430 | ZNF430 | 2.668262365 |
| XM_933893.1 | similar to 40S ribosomal protein SA (p40) (34/67 kDa laminin receptor) | LOC389672 | 2.657515903 |
| XR_019339.1 | similar to peptidase (prosome, macropain) 26S subunit, ATPase 1 | LOC643668 | 2.645362727 |
| XM_941155.2 | similar to ribosomal protein S12 | LOC651894 | 2.635958416 |
| NR_003264.1 | succinate dehydrogenase complex, subunit A, flavoprotein pseudogene 1 | SDHALP1 | 2.635693148 |
| NM_177530.1 | sulfotransferase family, cytosolic, 1A, phenol-preferring, member 1 | SULT1A1 | 2.633373278 |
| NM_181054.1 | hypoxia-inducible factor 1, alpha subunit (basic helix-loop-helix transcription factor) | HIF1A | 2.62458737 |
| NM_032440.1 | ligand dependent nuclear receptor corepressor | LCOR | 2.61026653 |
| NM_003925.1 | methyl-CpG binding domain protein 4 | MBD4 | 2.604459917 |
| NM_014033.3 | methyltransferase like 7A | METTL7A | 2.59609421 |
| NM_006360.3 | eukaryotic translation initiation factor 3, subunit M | EIF3M | 2.586386312 |
| XM_938988.1 | similar to actin alpha 1 skeletal muscle protein | LOC402221 | 2.575526281 |
| NM_001788.4 | septin 7 | SEPT7 | 2.574313885 |
| XM_001134259.1 | similar to Triosephosphate isomerase (TIM) (Triose-phosphate isomerase), transcript variant 2 | LOC732165 | 2.570685992 |
| XM_930178.1 | similar to ribosomal protein S2 | LOC645018 | 2.562724162 |
| NM_000971.3 | ribosomal protein L7 | RPL7 | 2.541303283 |
| NM_198334.1 | glucosidase, alpha; neutral AB | GANAB | 2.540679996 |
| NM_018698.3 | nuclear transport factor 2-like export factor 2 | NXT2 | 2.520203875 |
| NM_005665.4 | ecotropic viral integration site 5 | EVI5 | 2.511346244 |
| NM_006903.4 | pyrophosphatase (inorganic) 2 | PPA2 | 2.505531437 |
| NM_022487.2 | DNA cross-link repair 1C (PSO2 homolog, S. cerevisiae) | DCLRE1C | 2.503271502 |
| XM_940209.1 | KIAA0194 protein | KIAA0194 | 2.500462426 |
| NM_000978.3 | ribosomal protein L23 | RPL23 | 2.500003516 |
| NM_001080453.1 | integrator complex subunit 1 | INTS1 | 2.492222311 |
| NM_001030.3 | ribosomal protein S27 (metallopanstimulin 1) | RPS27 | 2.469558417 |
| NR_001562.1 | annexin A2 pseudogene 1 | ANXA2P1 | 2.455468266 |
| NM_024745.2 | SHC SH2-domain binding protein 1 | SHCBP1 | 2.454489025 |
| NM_145280.3 | family with sequence similarity 119, member A | FAM119A | 2.452334623 |
| NM_006628.4 | cyclic AMP phosphoprotein, 19 kD | ARPP-19 | 2.445138213 |
| NM_024570.1 | ribonuclease H2, subunit B | RNASEH2B | 2.443230828 |
| NM_005497.3 | gap junction protein, gamma 1, 45kDa | GJC1 | 2.440339345 |
| NM_001786.2 | cell division cycle 2, G1 to S and G2 to M | CDC2 | 2.410697673 |
| NM_004238.1 | thyroid hormone receptor interactor 12 | TRIP12 | 2.4063906 |
| NM_001031710.1 | kelch-like 7 (Drosophila) | KLHL7 | 2.400068474 |
| NM_001006.3 | ribosomal protein S3A | RPS3A | 2.393393056 |
| NM_001003712.1 | oxysterol binding protein-like 8 | OSBPL8 | 2.380920658 |
| NM_139207.1 | nucleosome assembly protein 1-like 1 | NAP1L1 | 2.359016844 |
| XM_001133089.1 | similar to 60S ribosomal protein L21, transcript variant 2 | LOC731640 | 2.354540935 |
| NM_144618.1 | hypothetical protein MGC29891 | MGC29891 | 2.348689614 |
| NM_002157.1 | heat shock 10kDa protein 1 (chaperonin 10) | HSPE1 | 2.345168654 |
| NM_005754.2 | GTPase activating protein (SH3 domain) binding protein 1 | G3BP1 | 2.33245452 |
| NM_001024921.2 | ribosomal protein L9 | RPL9 | 2.31701905 |
| NM_175066.2 | DEAD (Asp-Glu-Ala-Asp) box polypeptide 51 | DDX51 | 2.315376055 |
| NM_001081637.1 | leukocyte immunoglobulin-like receptor, subfamily B (with TM and ITIM domains), member 1 | LILRB1 | 2.311038763 |
| NM_000587.2 | complement component 7 | C7 | 2.296863097 |
| NM_032796.2 | synapse associated protein 1, SAP47 homolog (Drosophila) | SYAP1 | 2.295896993 |
| NM_003906.3 | minichromosome maintenance complex component 3 associated protein | MCM3AP | 2.274822216 |
| NM_174909.3 | transmembrane protein 167 | TMEM167 | 2.272867492 |
| NM_005245.3 | FAT tumor suppressor homolog 1 (Drosophila) | FAT | 2.267290235 |
| XM_940278.1 | similar to 60S ribosomal protein L3 (L4) | LOC651149 | 2.263512093 |
| NR_002205.1 | ferritin, heavy polypeptide-like 12 | FTHL12 | 2.20772061 |
| XR_017397.1 | similar to 60S ribosomal protein L7a | LOC644029 | 2.206742649 |
| NM_003403.3 | YY1 transcription factor | YY1 | 2.204276423 |
| NM_001039797.1 | hypothetical protein LOC649598 | FLJ46309 | 2.202024158 |
| NM_004687.3 | myotubularin related protein 4 | MTMR4 | 2.201057211 |
| NM_001006.3 | ribosomal protein S3A | RPS3A | 2.199893519 |
| NM_018249.4 | CDK5 regulatory subunit associated protein 2 | CDK5RAP2 | 2.183232289 |
| NM_001031827.1 | bolA homolog 2 (E. coli) | BOLA2 | 2.182993039 |
| XM_926594.2 | similar to succinate dehydrogenase complex, subunit C isoform 3 precursor | LOC642502 | 2.180239864 |
| NM_019600.1 | KIAA1370 | KIAA1370 | 2.171496436 |
| NM_002737.2 | protein kinase C, alpha | PRKCA | 2.164367181 |
| NM_213606.1 | solute carrier family 16, member 12 (monocarboxylic acid transporter 12) | SLC16A12 | 2.156718199 |
| NM_014935.2 | pleckstrin homology domain containing, family A member 6 | PLEKHA6 | 2.154192738 |
| NM_003589.2 | cullin 4A | CUL4A | 2.150721458 |
| NM_007112.3 | thrombospondin 3 | THBS3 | 2.144690944 |
| XM_937850.1 | similar to ribosomal protein L10 | LOC285176 | 2.14294639 |
| NM_001008735.1 | high-mobility group (nonhistone chromosomal) protein 1-like 1 | HMG1L1 | 2.141401345 |
| NM_006392.2 | nucleolar protein 5A (56kDa with KKE/D repeat) | NOL5A | 2.135415461 |
| NM_002497.2 | NIMA (never in mitosis gene a)-related kinase 2 | NEK2 | 2.12796729 |
| NR_002190.1 | SUMO1 pseudogene 3 | SUMO1P3 | 2.127669458 |
| NM_012215.2 | meningioma expressed antigen 5 (hyaluronidase) | MGEA5 | 2.126089887 |
| NM_012343.3 | nicotinamide nucleotide transhydrogenase | NNT | 2.118741206 |
| NM_006054.2 | reticulon 3 | RTN3 | 2.102655587 |
| NM_005334.2 | host cell factor C1 (VP16-accessory protein) | HCFC1 | 2.081192023 |
| XM_940610.1 | similar to ribosomal protein L36 | LOC651453 | 2.073886912 |
| NM_002650.1 | phosphatidylinositol 4-kinase, catalytic, alpha polypeptide | PIK4CA | 2.073504629 |
| NM_002685.2 | exosome component 10 | EXOSC10 | 2.069117186 |
| NM_002951.2 | ribophorin II | RPN2 | 2.067985488 |
| NM_001023567.2 | golgi autoantigen, golgin subfamily a, 8B | GOLGA8B | 2.061096888 |
| NM_182920.1 | ADAM metallopeptidase with thrombospondin type 1 motif, 9 | ADAMTS9 | 2.054616996 |
| NM_006601.4 | prostaglandin E synthase 3 (cytosolic) | PTGES3 | 2.053574119 |
| NM_174921.1 | chromosome 4 open reading frame 34 | C4orf34 | 2.046486355 |
| BX647087 | mRNA; cDNA DKFZp686B24166 | DKFZp686B24166 | 2.038728594 |
| NM_001034996.1 | ribosomal protein L14 | RPL14 | 2.034131051 |
| NM_080702.2 | HLA-B associated transcript 3 | BAT3 | 2.03273012 |
| NM_173630.2 | rotatin | RTTN | 2.032608049 |
| NM_015226.1 | C-type lectin domain family 16, member A | CLEC16A | 2.031418301 |
| NM_198795.1 | tudor domain containing 1 | TDRD1 | 2.021119841 |
| NM_020724.1 | ring finger protein 150 | RNF150 | 2.020306344 |
| NM_001019.4 | ribosomal protein S15a | RPS15A | 2.017017102 |
| NM_194301.2 | GTPase activating Rap/RanGAP domain-like 1 | GARNL1 | 2.015287592 |
| NR_001283.1 | topoisomerase (DNA) I pseudogene 2 | TOP1P2 | 2.012695002 |
| NM_016245.3 | hydroxysteroid (17-beta) dehydrogenase 11 | HSD17B11 | 1.9995536 |
| NM_199053.1 | chromosome 4 open reading frame 41 | C4orf41 | 1.987413574 |
| XM_939726.2 | similar to ribosomal protein L21 | LOC388532 | 1.975634079 |
| NM_001081640.1 | protein kinase, DNA-activated, catalytic polypeptide | PRKDC | 1.965295418 |
| NM_015348.1 | transmembrane protein 131 | TMEM131 | 1.951438003 |
| XM_497072.2 | similar to Translationally-controlled tumor protein (TCTP) (p23) (Histamine-releasing factor) | LOC389787 | 1.948921323 |
| XM_938599.2 | similar to 40S ribosomal protein S26 | LOC441377 | 1.948164142 |
| XM_377933.3 | similar to eukaryotic translation elongation factor 1 alpha 2 | LOC402251 | 1.945241537 |
| NM_030881.2 | DEAD (Asp-Glu-Ala-Asp) box polypeptide 17 | DDX17 | 1.941606949 |
| NM_015690.2 | serine/threonine kinase 36, fused homolog (Drosophila) | STK36 | 1.940208403 |
| NM_018639.3 | WD repeat and SOCS box-containing 2 | WSB2 | 1.93647106 |
| NM_018451.3 | centromere protein J | CENPJ | 1.93116989 |
| NM_004897.2 | multiple inositol polyphosphate histidine phosphatase, 1 | MINPP1 | 1.930901467 |
| NM_016114.3 | ankyrin repeat and SOCS box-containing 1 | ASB1 | 1.929377136 |
| NM_016023.2 | OTU domain containing 6B | OTUD6B | 1.929021044 |
| XM_942687.1 | similar to heterogeneous nuclear ribonucleoprotein A3, transcript variant 1 | LOC654189 | 1.927681116 |
| NM_001001789.1 | chromosome 21 open reading frame 24 | C21orf24 | 1.925150422 |
| NM_001253.2 | CDC5 cell division cycle 5-like (S. pombe) | CDC5L | 1.922472496 |
| NM_001077199.1 | splicing factor, arginine/serine-rich 12 | SFRS12 | 1.918239301 |
| NM_003496.1 | transformation/transcription domain-associated protein | TRRAP | 1.913068222 |
| NM_001037533.1 | gon-4-like (C. elegans) | GON4L | 1.90684214 |
| NM_032026.1 | TatD DNase domain containing 1 | TATDN1 | 1.902070049 |
| XM_939687.2 | similar to ribosomal protein S23 | LOC653658 | 1.900387489 |
| NM_031372.1 | heterogeneous nuclear ribonucleoprotein D-like | HNRPDL | 1.88897605 |
| XM_935589.1 | similar to 40S ribosomal protein S3a (V-fos transformation effector protein) | LOC641849 | 1.884323453 |
| NM_006036.2 | prolyl endopeptidase-like | PREPL | 1.870277185 |
| NM_002734.3 | protein kinase, cAMP-dependent, regulatory, type I, alpha (tissue specific extinguisher 1) | PRKAR1A | 1.867789995 |
| NM_004048.2 | beta-2-microglobulin | B2M | 1.858769622 |
| XM_933970.1 | hypothetical protein LOC646849 | LOC646849 | 1.856161684 |
| NM_018151.3 | RAP1 interacting factor homolog (yeast) | RIF1 | 1.848588813 |
| NM_000051.3 | ataxia telangiectasia mutated | ATM | 1.848157114 |
| NM_020822.1 | potassium channel, subfamily T, member 1 | KCNT1 | 1.845567812 |
| NM_005688.2 | ATP-binding cassette, sub-family C | ABCC5 | 1.840851506 |
| NM_018116.2 | misato homolog 1 (Drosophila) | MSTO1 | 1.83646005 |
| NM_016343.3 | centromere protein F, 350/400ka (mitosin) | CENPF | 1.828316852 |
| NM_020117.9 | leucyl-tRNA synthetase | LARS | 1.826831827 |
| NM_001039705.1 | trophinin | TRO | 1.825102027 |
| NM_001001679.1 | FLJ41423 protein | FLJ41423 | 1.810708829 |
| XM_044178.10 | KIAA1211 protein, transcript variant 1 | KIAA1211 | 1.809079858 |
| NM_015938.2 | NMD3 homolog (S. cerevisiae) | NMD3 | 1.805887425 |
| NM_006527.2 | stem-loop binding protein | SLBP | 1.800890852 |
| NM_012223.2 | myosin IB | MYO1B | 1.79049998 |
| NM_016333.2 | serine/arginine repetitive matrix 2 | SRRM2 | 1.789249369 |
| NM_001418.3 | eukaryotic translation initiation factor 4 gamma, 2 | EIF4G2 | 1.78348761 |
| NM_015245.2 | ankyrin repeat and sterile alpha motif domain containing 1A | ANKS1A | 1.782732884 |
| NM_005520.1 | heterogeneous nuclear ribonucleoprotein H1 (H) | HNRPH1 | 1.778731798 |
| NM_012130.2 | claudin 14 | CLDN14 | 1.776138299 |
| NM_000368.3 | tuberous sclerosis 1 | TSC1 | 1.766460341 |
| AK026373 | cDNA: FLJ22720 fis, clone HSI14320 | FLJ22720 | 1.760682841 |
| NM_006885.3 | zinc finger homeobox 3 | ZFHX3 | 1.75733589 |
| NM_019063.2 | echinoderm microtubule associated protein like 4 | EML4 | 1.756164919 |
| NR_002182.1 | nascent-polypeptide-associated complex alpha polypeptide pseudogene 1 | NACAP1 | 1.753656115 |
| NM_198836.1 | acetyl-Coenzyme A carboxylase alpha | ACACA | 1.752660234 |
| NM_004627.2 | tryptophan rich basic protein | WRB | 1.749289839 |
| NM_001083621.1 | zinc finger and BTB domain containing 40 | ZBTB40 | 1.73945009 |
| NM_020918.2 | glycerol-3-phosphate acyltransferase, mitochondrial | GPAM | 1.738746133 |
| NM_017641.2 | kinesin family member 21A | KIF21A | 1.734519807 |
| NM_018193.2 | Fanconi anemia, complementation group I | FANCI | 1.726175443 |
| NM_012484.1 | hyaluronan-mediated motility receptor | HMMR | 1.719591858 |
| XM_001133534.1 | ATPase, Na+/K+ transporting, beta 3 polypeptide, transcript variant 2 | ATP1B3 | 1.718564701 |
| NM_001092.3 | active BCR-related gene | ABR | 1.715059065 |
| NM_153201.1 | heat shock 70kDa protein 8 | HSPA8 | 1.712948137 |
| NM_021227.2 | DC2 protein | DC2 | 1.711726902 |
| NM_014747.2 | regulating synaptic membrane exocytosis 3 | RIMS3 | 1.705357585 |
| NM_001006115.2 | inositol hexaphosphate kinase 1 | IHPK1 | 1.702108406 |
| NM_003601.2 | SWI/SNF related, matrix associated, actin dependent regulator of chromatin, subfamily a, member 5 | SMARCA5 | 1.699909759 |
| NM_001005849.1 | SMT3 suppressor of mif two 3 homolog 2 (S. cerevisiae) | SUMO2 | 1.687805298 |
| NM_015655.2 | zinc finger protein 337 | ZNF337 | 1.679966318 |
| NM_001111.3 | adenosine deaminase, RNA-specific | ADAR | 1.674603006 |
| NM_001044387.1 | zinc finger protein 557 | ZNF557 | 1.671188177 |
| NM_003086.2 | small nuclear RNA activating complex, polypeptide 4, 190kDa | SNAPC4 | 1.666669777 |
| NR_001568.1 | brain cytoplasmic RNA 1, Bc1 analog (mouse) | BCYRN1 | 1.661639319 |
| NM_015144.2 | zinc finger, CCHC domain containing 14 | ZCCHC14 | 1.66044787 |
| NM_003972.2 | BTAF1 RNA polymerase II, B-TFIID transcription factor-associated, 170kDa (Mot1 homolog, S. cerevisiae) | BTAF1 | 1.658355308 |
| NM_001319.5 | casein kinase 1, gamma 2 | CSNK1G2 | 1.65798008 |
| NM_022757.3 | coiled-coil domain containing 14 | CCDC14 | 1.652468938 |
| NM_012063.1 | dynamin 1-like | DNM1L | 1.64885879 |
| NM_005560.3 | laminin, alpha 5 | LAMA5 | 1.640084292 |
| NM_020040.3 | tubulin, beta polypeptide 4, member Q | TUBB4Q | 1.638525069 |
| NM_006437.3 | poly (ADP-ribose) polymerase family, member 4 | PARP4 | 1.63510476 |
| NM_004539.3 | asparaginyl-tRNA synthetase | NARS | 1.633656855 |
| NM_014871.2 | PAN2 polyA specific ribonuclease subunit homolog (S. cerevisiae) | PAN2 | 1.633457348 |
| XR_016986.1 | similar to peptidase (prosome, macropain) 26S subunit, ATPase 1 | LOC643668 | 1.633415366 |
| NM_002266.2 | karyopherin alpha 2 (RAG cohort 1, importin alpha 1) | KPNA2 | 1.630199632 |
| NM_181442.1 | activity-dependent neuroprotector homeobox | ADNP | 1.629953961 |
| NM_001077628.1 | anterior pharynx defective 1 homolog A (C. elegans) | APH1A | 1.629473405 |
| NM_182649.1 | proliferating cell nuclear antigen | PCNA | 1.629436488 |
| NM_018098.4 | epithelial cell transforming sequence 2 oncogene | ECT2 | 1.628891032 |
| XR_018923.1 | similar to Heterogeneous nuclear ribonucleoprotein A1 (Helix-destabilizing protein) | LOC648210 | 1.626493407 |
| NM_001031744.1 | hypothetical protein LOC158160 | LOC158160 | 1.62219059 |
| NM_000969.3 | ribosomal protein L5 | RPL5 | 1.616543139 |
| NM_022451.9 | nucleolar complex associated 3 homolog (S. cerevisiae) | NOC3L | 1.604212109 |
| NM_203459.1 | calmodulin regulated spectrin-associated protein 1-like 1 | CAMSAP1L1 | 1.602125288 |
| NM_152902.3 | TIP41, TOR signaling pathway regulator-like (S. cerevisiae) | TIPRL | 1.598753529 |
| NM_003750.2 | eukaryotic translation initiation factor 3, subunit A | EIF3A | 1.589442802 |
| NM_014367.3 | chromosome 3 open reading frame 28 | C3orf28 | 1.589386714 |
| NM_015902.4 | ubiquitin protein ligase E3 component n-recognin 5 | UBR5 | 1.583145045 |
| NM_021145.2 | cyclin D binding myb-like transcription factor 1 | DMTF1 | 1.581253593 |
| NM_001008800.1 | chaperonin containing TCP1, subunit 3 (gamma) | CCT3 | 1.579593577 |
| NM_025230.3 | WD repeat domain 23 | WDR23 | 1.579196347 |
| NM_014647.2 | KIAA0430 | KIAA0430 | 1.578329694 |
| NM_182569.1 | glycerophosphodiester phosphodiesterase domain containing 1 | GDPD1 | 1.576242059 |
| NM_012428.2 | neuroplastin | NPTN | 1.575321117 |
| NM_001407.2 | cadherin, EGF LAG seven-pass G-type receptor 3 (flamingo homolog, Drosophila) | CELSR3 | 1.569778277 |
| NM_031423.3 | NUF2, NDC80 kinetochore complex component, homolog (S. cerevisiae) | NUF2 | 1.568413282 |
| NM_031445.2 | AMME chromosomal region gene 1-like | AMMECR1L | 1.559689576 |
| NM_030752.2 | t-complex 1 | TCP1 | 1.552277451 |
| NM_012207.1 | heterogeneous nuclear ribonucleoprotein H3 (2H9) | HNRPH3 | 1.539709043 |
| NM_001412.3 | eukaryotic translation initiation factor 1A, X-linked | EIF1AX | 1.537330223 |
| NM_020748.1 | integrator complex subunit 2 | INTS2 | 1.536357498 |
| NM_145341.2 | programmed cell death 4 (neoplastic transformation inhibitor) | PDCD4 | 1.53408673 |
| NM_001011537.1 | forty-two-three domain containing 1 | FYTTD1 | 1.515846017 |
| NM_033285.2 | tumor protein p53 inducible nuclear protein 1 | TP53INP1 | 1.512799146 |
| AK095147 | cDNA FLJ37828 fis, clone BRSSN2006575 | FLJ37828 | 1.506644034 |
| NM_005238.2 | v-ets erythroblastosis virus E26 oncogene homolog 1 (avian) | ETS1 | 1.503689872 |
| NM_017948.4 | nucleolar protein 8 | NOL8 | 1.502902263 |
| NM_025191.2 | ER degradation enhancer, mannosidase alpha-like 3 | EDEM3 | 1.502238453 |
| AL157484 | mRNA; cDNA DKFZp762M127 (from clone DKFZp762M127) | DKFZp762M127 | 1.501271729 |
| NM_016626.3 | mex-3 homolog C (C. elegans) | MEX3C | 1.500787428 |
| NR_002797.1 | hypothetical protein LOC255783 | LOC255783 | -1.500072144 |
| NM_213650.1 | sideroflexin 4 | SFXN4 | -1.501151257 |
| NM_014007.2 | zinc finger and BTB domain containing 43 | ZBTB43 | -1.501788701 |
| NM_030771.1 | coiled-coil domain containing 34 | CCDC34 | -1.502295588 |
| NM_024063.1 | spermatogenesis associated 5-like 1 | SPATA5L1 | -1.502717149 |
| NM_012234.4 | RING1 and YY1 binding protein | RYBP | -1.502996825 |
| NM_001283.2 | adaptor-related protein complex 1, sigma 1 subunit | AP1S1 | -1.507955757 |
| NM_006571.2 | dynactin 6 | DCTN6 | -1.508575709 |
| NM_031892.1 | SH3-domain kinase binding protein 1 | SH3KBP1 | -1.508635359 |
| NM_017970.2 | chromosome 14 open reading frame 102 | C14orf102 | -1.510599314 |
| NM_001008405.1 | B-cell receptor-associated protein 29 | BCAP29 | -1.51219313 |
| NM_053050.2 | mitochondrial ribosomal protein L53 | MRPL53 | -1.512319918 |
| NM_016941.2 | delta-like 3 (Drosophila) | DLL3 | -1.5130053 |
| NM_005793.3 | non-metastatic cells 6, protein expressed in (nucleoside-diphosphate kinase) | NME6 | -1.513322497 |
| NM_031287.2 | splicing factor 3b, subunit 5, 10kDa | SF3B5 | -1.515996181 |
| XM_001129423.1 | hypothetical protein LOC729137 | LOC729137 | -1.518632637 |
| NM_001039708.1 | serologically defined colon cancer antigen 3 | SDCCAG3 | -1.520825032 |
| XM_498571.2 | hypothetical LOC440160 | LOC440160 | -1.521182808 |
| NM_025205.3 | mediator complex subunit 28 | MED28 | -1.52370501 |
| NM_021004.2 | dehydrogenase/reductase (SDR family) member 4 | DHRS4 | -1.527739032 |
| NM_001002018.1 | host cell factor C1 regulator 1 (XPO1 dependent) | HCFC1R1 | -1.529320404 |
| XM_941942.1 | similar to Mitochondrial import inner membrane translocase subunit Tim23 | LOC652481 | -1.531611172 |
| XM_936354.2 | similar to Protein FAM82B | LOC642197 | -1.536585205 |
| NM_180976.1 | protein phosphatase 2, regulatory subunit B', delta isoform | PPP2R5D | -1.542624933 |
| NM_017528.2 | Williams Beuren syndrome chromosome region 22 | WBSCR22 | -1.543374972 |
| NM_030917.2 | FIP1 like 1 (S. cerevisiae) | FIP1L1 | -1.545230048 |
| NM_001078650.1 | transmembrane protein 134 | TMEM134 | -1.546468003 |
| NM_024326.2 | F-box and leucine-rich repeat protein 15 | FBXL15 | -1.551171853 |
| NM_022761.2 | chromosome 11 open reading frame 1 | C11orf1 | -1.55190879 |
| NM_080605.3 | UDP-Gal:betaGal beta 1,3-galactosyltransferase polypeptide 6 | B3GALT6 | -1.552181199 |
| NM_032638.3 | GATA binding protein 2 | GATA2 | -1.552554341 |
| NM_153824.1 | pyrroline-5-carboxylate reductase 1 | PYCR1 | -1.55378704 |
| NM_138765.2 | BCL2-associated X protein | BAX | -1.554490991 |
| NM_024065.3 | phosducin-like 3 | PDCL3 | -1.558182561 |
| NM_014167.2 | coiled-coil domain containing 59 | CCDC59 | -1.571786634 |
| NM_058181.1 | chromosome 21 open reading frame 57 | C21orf57 | -1.571931909 |
| NM_015957.1 | APAF1 interacting protein | APIP | -1.576639026 |
| NM_017971.2 | mitochondrial ribosomal protein L20 | MRPL20 | -1.577209661 |
| NM_016587.2 | chromobox homolog 3 (HP1 gamma homolog, Drosophila) | CBX3 | -1.577467265 |
| NM_152379.2 | chromosome 1 open reading frame 131 | C1orf131 | -1.586992847 |
| NM_058181.1 | chromosome 21 open reading frame 57 | C21orf57 | -1.592101805 |
| NM_203284.1 | recombination signal binding protein for immunoglobulin kappa J region | RBPJ | -1.595562144 |
| NM_007100.2 | ATP synthase, H+ transporting, mitochondrial F0 complex, subunit E | ATP5I | -1.599955637 |
| NM_017907.1 | chromosome 11 open reading frame 59 | C11orf59 | -1.600169504 |
| NM_014886.3 | TGF beta-inducible nuclear protein 1 | TINP1 | -1.600645317 |
| NM_212552.2 | bolA homolog 3 (E. coli) | BOLA3 | -1.605044611 |
| NM_032111.2 | mitochondrial ribosomal protein L14 | MRPL14 | -1.606905559 |
| NM_138701.1 | chromosome 7 open reading frame 11 | C7orf11 | -1.607096518 |
| NM_013328.2 | pyrroline-5-carboxylate reductase family, member 2 | PYCR2 | -1.615738583 |
| XM_944321.1 | hypothetical LOC402560 | LOC402560 | -1.617745605 |
| NM_006515.1 | SET domain and mariner transposase fusion gene | SETMAR | -1.617897454 |
| NM_020533.1 | mucolipin 1 | MCOLN1 | -1.619215933 |
| NM_019554.2 | S100 calcium binding protein A4 | S100A4 | -1.625823211 |
| NM_052873.1 | chromosome 14 open reading frame 179 | C14orf179 | -1.626767176 |
| NM_001031703.2 | transmembrane protein 103 | TMEM103 | -1.626830617 |
| NR_002144.1 | mitogen-activated protein kinase kinase 2 pseudogene | LOC407835 | -1.631214234 |
| NM_032907.3 | ubiquitin-like 7 (bone marrow stromal cell-derived) | UBL7 | -1.631563751 |
| NM_001001795.1 | similar to RIKEN cDNA C030006K11 gene | MGC70857 | -1.63184364 |
| NM_003172.2 | surfeit 1 | SURF1 | -1.633653691 |
| NM_032993.1 | nucleolar protein family A, member 1 (H/ACA small nucleolar RNPs) | NOLA1 | -1.634044929 |
| NM_032747.2 | upregulated during skeletal muscle growth 5 homolog (mouse) | USMG5 | -1.634979483 |
| NM_003077.2 | SWI/SNF related, matrix associated, actin dependent regulator of chromatin, subfamily d, member 2 | SMARCD2 | -1.646631092 |
| NM_018473.2 | thioesterase superfamily member 2 | THEM2 | -1.648413768 |
| NM_004435.2 | endonuclease G | ENDOG | -1.64963484 |
| NM_054014.1 | FK506 binding protein 1A, 12kDa | FKBP1A | -1.650042648 |
| NM_181702.1 | GTP binding protein overexpressed in skeletal muscle | GEM | -1.653321737 |
| NM_004615.2 | tetraspanin 7 | TSPAN7 | -1.654814237 |
| XM_936354.2 | similar to Protein FAM82B | LOC642197 | -1.655253665 |
| NM_014161.2 | mitochondrial ribosomal protein L18 | MRPL18 | -1.656062435 |
| NM_005719.2 | actin related protein 2/3 complex, subunit 3, 21kDa | ARPC3 | -1.659272478 |
| NM_003368.4 | ubiquitin specific peptidase 1 | USP1 | -1.659564686 |
| NM_002712.1 | protein phosphatase 1, regulatory (inhibitor) subunit 7 | PPP1R7 | -1.659726153 |
| NM_016535.3 | zinc finger protein 581 | ZNF581 | -1.663164562 |
| NM_004175.3 | small nuclear ribonucleoprotein D3 polypeptide 18kDa | SNRPD3 | -1.666205262 |
| NM_001037675.1 | neuroblastoma breakpoint family, member 20 | NBPF20 | -1.669123205 |
| NM_001312.2 | cysteine-rich protein 2 | CRIP2 | -1.671093964 |
| NM_023011.2 | UPF3 regulator of nonsense transcripts homolog A (yeast) | UPF3A | -1.672175458 |
| NM_000076.1 | cyclin-dependent kinase inhibitor 1C (p57, Kip2) | CDKN1C | -1.674279725 |
| NM_014183.2 | dynein, light chain, roadblock-type 1 | DYNLRB1 | -1.675732034 |
| NM_199235.1 | collectin sub-family member 11 | COLEC11 | -1.676444188 |
| NM_001003795.2 | GTF2I repeat domain containing 2B | GTF2IRD2B | -1.677050731 |
| XM_944786.1 | hypothetical protein LOC650737, transcript variant 1 | LOC650737 | -1.679950095 |
| NM_000551.2 | von Hippel-Lindau tumor suppressor | VHL | -1.690984652 |
| NM_080593.1 | histone cluster 1, H2bk | HIST1H2BK | -1.692270596 |
| NM_178439.3 | germ cell-less homolog 1 (Drosophila) | GMCL1 | -1.692701441 |
| NM_004378.1 | cellular retinoic acid binding protein 1 | CRABP1 | -1.693293054 |
| NM_024301.3 | fukutin related protein | FKRP | -1.69475433 |
| NM_006191.2 | proliferation-associated 2G4, 38kDa | PA2G4 | -1.698103011 |
| NM_012110.2 | cysteine-rich hydrophobic domain 2 | CHIC2 | -1.70365692 |
| NM_004798.2 | kinesin family member 3B | KIF3B | -1.705742222 |
| NM_058219.2 | exosome component 6 | EXOSC6 | -1.706164183 |
| NM_024109.2 | chromosome 16 open reading frame 68 | C16orf68 | -1.711228271 |
| XM_001132569.1 | hypothetical protein LOC730130 | LOC730130 | -1.711780154 |
| NR_001445.1 | RNA, 7SK small nuclear | RN7SK | -1.71320693 |
| NM_001003725.1 | WD repeat domain 68 | WDR68 | -1.716528298 |
| NM_001006684.1 | transcription elongation factor A (SII)-like 8 | TCEAL8 | -1.716534709 |
| NM_016463.5 | CXXC finger 5 | CXXC5 | -1.719251616 |
| NM_003221.3 | transcription factor AP-2 beta (activating enhancer binding protein 2 beta) | TFAP2B | -1.723297455 |
| NM_032926.2 | transcription elongation factor A (SII)-like 3 | TCEAL3 | -1.72554348 |
| NM_199287.2 | coiled-coil domain containing 137 | CCDC137 | -1.738275584 |
| XM_933956.1 | similar to septin 7, transcript variant 4 | LOC644162 | -1.748345151 |
| NM_018332.3 | DEAD (Asp-Glu-Ala-As) box polypeptide 19A | DDX19A | -1.750568661 |
| NR_003041.1 | small nucleolar RNA, C/D box 13 | SNORD13 | -1.752395867 |
| NM_001444.1 | fatty acid binding protein 5 (psoriasis-associated) | FABP5 | -1.759962677 |
| NM_006357.2 | ubiquitin-conjugating enzyme E2E 3 (UBC4/5 homolog, yeast) | UBE2E3 | -1.772796731 |
| NM_001008709.1 | protein phosphatase 1, catalytic subunit, alpha isoform | PPP1CA | -1.776944407 |
| NM_024321.3 | RNA binding motif protein 42 | RBM42 | -1.780777807 |
| NM_018064.2 | chromosome 6 open reading frame 166 | C6orf166 | -1.783711755 |
| NM_174893.1 | chromosome 17 open reading frame 49 | C17orf49 | -1.78545435 |
| NM_002225.2 | isovaleryl Coenzyme A dehydrogenase | IVD | -1.787699942 |
| NM_181454.1 | mitochondrial ribosomal protein L55 | MRPL55 | -1.788999537 |
| NM_001018020.1 | tropomyosin 1 (alpha) | TPM1 | -1.790257126 |
| NM_000714.4 | translocator protein (18kDa) | TSPO | -1.790890064 |
| NM_016208.2 | vacuolar protein sorting 28 homolog (S. cerevisiae) | VPS28 | -1.793569797 |
| NM_057089.2 | adaptor-related protein complex 1, sigma 1 subunit | AP1S1 | -1.795484575 |
| NM_005953.2 | metallothionein 2A | MT2A | -1.804132006 |
| NM_001035505.1 | bolA homolog 3 (E. coli) | BOLA3 | -1.810054409 |
| NM_207350.1 | similar to FRG1 protein | MGC72104 | -1.815483857 |
| NM_022549.2 | fasciculation and elongation protein zeta 1 | FEZ1 | -1.816500427 |
| NM_001032382.1 | polyglutamine binding protein 1 | PQBP1 | -1.823066117 |
| NM_001042370.1 | TROVE domain family, member 2 | TROVE2 | -1.832978297 |
| NM_016374.5 | AT rich interactive domain 4B | ARID4B | -1.838214889 |
| NM_198486.2 | ribosomal protein L7-like 1 | RPL7L1 | -1.838922117 |
| NM_001042631.1 | hypothetical protein LOC644096 | LOC644096 | -1.851404321 |
| NM_144591.2 | chromosome 10 open reading frame 32 | C10orf32 | -1.85169748 |
| NM_138720.1 | histone cluster 1, H2bd | HIST1H2BD | -1.855105115 |
| NM_007198.2 | proline synthetase co-transcribed homolog (bacterial) | PROSC | -1.86653456 |
| NM_018476.3 | brain expressed, X-linked 1 | BEX1 | -1.872500257 |
| NM_001031711.1 | endoplasmic reticulum-golgi intermediate compartment | ERGIC1 | -1.879165976 |
| NM_199295.1 | apoptosis-inducing, TAF9-like domain 1 | APITD1 | -1.887689438 |
| NM_015444.2 | transmembrane protein 158 | TMEM158 | -1.891646034 |
| NM_182547.2 | transmembrane emp24 protein transport domain containing 4 | TMED4 | -1.8946434 |
| XM_936103.1 | similar to ATP-binding cassette, sub-family F, member 1 isoform b | LOC642033 | -1.896231856 |
| NM_018983.3 | nucleolar protein family A, member 1 (H/ACA small nucleolar RNPs) | NOLA1 | -1.89881672 |
| NM_080651.1 | mediator complex subunit 30 | MED30 | -1.906119192 |
| NM_001002755.1 | NFU1 iron-sulfur cluster scaffold homolog (S. cerevisiae) | NFU1 | -1.906903473 |
| NM_145012.3 | cyclin Y | CCNY | -1.919510898 |
| NM_005952.2 | metallothionein 1X | MT1X | -1.923978115 |
| NM_198970.1 | amino-terminal enhancer of split | AES | -1.924086511 |
| NM_178863.2 | potassium channel tetramerisation domain containing 13 | KCTD13 | -1.936036802 |
| NM_006860.2 | RAB, member of RAS oncogene family-like 4 | RABL4 | -1.944031794 |
| NM_012117.1 | chromobox homolog 5 (HP1 alpha homolog, Drosophila) | CBX5 | -1.946976648 |
| NM_004901.2 | ectonucleoside triphosphate diphosphohydrolase 4 | ENTPD4 | -1.952665259 |
| NM_001040056.1 | mitogen-activated protein kinase 3 | MAPK3 | -1.955816098 |
| NM_178439.3 | germ cell-less homolog 1 (Drosophila) | GMCL1 | -1.959514008 |
| NM_001014438.1 | cysteinyl-tRNA synthetase | CARS | -1.968032204 |
| XR_015638.1 | similar to MAPK-interacting and spindle-stabilizing protein | LOC731878 | -1.9682832 |
| NM_152274.2 | family with sequence similarity 58, member A | FAM58A | -1.970189952 |
| NM_001078172.1 | family with sequence similarity 127, member B | FAM127B | -1.971936799 |
| NM_152653.2 | ubiquitin-conjugating enzyme E2E 2 | UBE2E2 | -1.975368151 |
| NM_032343.1 | coiled-coil-helix-coiled-coil-helix domain containing 6 | CHCHD6 | -1.978147267 |
| NM_014620.4 | homeobox C4 | HOXC4 | -1.98290661 |
| CD640673 | AGENCOURT_14535501 NIH_MGC_191 | NIH_MGC_191 | -1.990353743 |
| NM_003211.3 | thymine-DNA glycosylase | TDG | -1.999256843 |
| NM_007155.4 | zona pellucida glycoprotein 3 (sperm receptor) | ZP3 | -1.999981191 |
| NM_001545.1 | immature colon carcinoma transcript 1 | ICT1 | -2.005946497 |
| NM_006471.2 | myosin regulatory light chain MRCL3 | MRCL3 | -2.019120629 |
| NM_006182.2 | discoidin domain receptor tyrosine kinase 2 | DDR2 | -2.022910226 |
| NM_002263.2 | kinesin family member C1 | KIFC1 | -2.024839509 |
| NM_020851.1 | immunoglobulin superfamily containing leucine-rich repeat 2 | ISLR2 | -2.027824598 |
| NM_002949.2 | mitochondrial ribosomal protein L12 | MRPL12 | -2.034636017 |
| NM_001006933.1 | transcription elongation factor A (SII)-like 3 | TCEAL3 | -2.043273842 |
| NM_005573.2 | lamin B1 | LMNB1 | -2.062595079 |
| NM_004643.1 | poly(A) binding protein, nuclear 1 | PABPN1 | -2.081585199 |
| NM_006455.2 | synaptonemal complex protein SC65 | SC65 | -2.08230497 |
| NM_024011.2 | cell division cycle 2-like 2 | CDC2L2 | -2.120896945 |
| NM_033445.2 | histone cluster 3, H2a | HIST3H2A | -2.139500304 |
| NM_201414.1 | amyloid beta (A4) precursor protein | APP | -2.141511272 |
| NM_016558.2 | SCAN domain containing 1 | SCAND1 | -2.150433219 |
| NM_001048197.1 | SNHG3-RCC1 | SNHG3-RCC1 | -2.191880691 |
| NM_005413.1 | sine oculis homeobox homolog 3 (Drosophila) | SIX3 | -2.194324877 |
| NM_080593.1 | histone cluster 1, H2bk | HIST1H2BK | -2.270698116 |
| NM_031453.2 | family with sequence similarity 107, member B | FAM107B | -2.291233273 |
| NM_014596.4 | zinc ribbon domain containing 1 | ZNRD1 | -2.292489637 |
| NM_001078651.1 | transmembrane protein 134 | TMEM134 | -2.306700611 |
| NM_005749.2 | transducer of ERBB2, 1 | TOB1 | -2.345835906 |
| NM_012133.2 | coatomer protein complex, subunit gamma 2 | COPG2 | -2.34934517 |
| NM_002897.3 | RNA binding motif, single stranded interacting protein 1 | RBMS1 | -2.369547311 |
| NM_184234.1 | RNA binding motif protein 39 | RBM39 | -2.382788901 |
| NM_181702.1 | GTP binding protein overexpressed in skeletal muscle | GEM | -2.411019087 |
| NM_002093.2 | glycogen synthase kinase 3 beta | GSK3B | -2.471732826 |
| NM_012433.2 | splicing factor 3b, subunit 1, 155kDa | SF3B1 | -2.525355454 |
| NM_005589.2 | aldehyde dehydrogenase 6 family, member A1 | ALDH6A1 | -2.52948049 |
| NM_002086.3 | growth factor receptor-bound protein 2 | GRB2 | -2.531228354 |
| NM_006717.2 | spindlin 1 | SPIN1 | -2.625837196 |
| NM_016374.5 | AT rich interactive domain 4B (RBP1-like) | ARID4B | -2.735320562 |
| NM_004456.3 | enhancer of zeste homolog 2 (Drosophila) | EZH2 | -2.761566957 |
| XM_930284.1 | hypothetical LOC441763 | LOC441763 | -2.902762562 |
| NM_006265.1 | RAD21 homolog (S. pombe) | RAD21 | -2.910916218 |
| NM_078629.1 | male-specific lethal 3-like 1 (Drosophila) | MSL3L1 | -3.138422741 |
| NM_001634.4 | adenosylmethionine decarboxylase 1 | AMD1 | -3.240970103 |
