# Supplementary material for: Minimal Peroxide Exposure of Neuronal Cells Induces Multifaceted Adaptive Responses
Source: PLoS One. 2010 Dec 17;5(12):e14352. doi: 10.1371/journal.pone.0014352 (PMC3003681; doi:10.1371/journal.pone.0014352)
Supplement: Table S2 — Latent semantic indexing textual interrogation of basal state CMP-dependent transcriptome alterations in SH-SY5Y cells. Latent semantic indexing (LSI) correlation scores as well as relevant gene symbols and genetic definition are denoted for each specific underlined interrogation term. Genes identified using LSI correlation that were upregulated compared to control by the CMP protocol are denoted in bold while those downregulated compared to control by CMP are denoted in italic. (0.44 MB DOC) [file pone.0014352.s009.doc]

**Table S2. Latent semantic indexing textual interrogation of basal state CMP-dependent transcriptome alterations in SH-SY5Y cells.** Latent semantic indexing (LSI) correlation scores as well as relevant gene symbols and genetic definition are denoted for each specific underlined interrogation term. Genes identified using LSI correlation that were upregulated compared to control by the CMP protocol are denoted in bold while those downregulated compared to control by CMP are denoted in *italic*.

| **BDNF** |  |  |
| --- | --- | --- |
|  |  |  |
| **LSI Correlation** | **Symbol** | **Definition** |
|  |  |  |
| 0.212 | tmem131 | **transmembrane protein 131** |
| 0.189 | adnp | **activity-dependent neuroprotector homeobox** |
| 0.181 | grb2 | *growth factor receptor-bound protein 2* |
| 0.175 | bax | *BCL2-associated X protein* |
| 0.173 | mapk3 | *mitogen-activated protein kinase 3* |
| 0.167 | pcna | **proliferating cell nuclear antigen** |
| 0.166 | bex1 | *brain expressed, X-linked 1* |
| 0.153 | arpp-19 | **cyclic AMP phosphoprotein, 19 kD** |
| 0.143 | tfap2b | *transcription factor AP-2 beta (activating enhancer binding protein 2 beta)* |
| 0.1256 | rps15a | **ribosomal protein S15a** |
| 0.1027 | gsk3b | *glycogen synthase kinase 3 beta* |
|  |  |  |
| **Acetylcholine** |  |  |
|  |  |  |
| **LSI Correlation** | **Symbol** | **Definition** |
|  |  |  |
| 0.21 | slc44a4 | **solute carrier family 44, member 4** |
| 0.103 | c6orf170 | **chromosome 6 open reading frame 170** |
|  |  |  |
| **Neurotrophin receptor** |  |  |
|  |  |  |
| **LSI Correlation** | **Symbol** | **Definition** |
|  |  |  |
| 0.356 | shcbp1 | **SHC SH2-domain binding protein 1** |
| 0.339 | anks1a | **ankyrin repeat and sterile alpha motif domain containing 1A** |
| 0.336 | tmem131 | **transmembrane protein 131** |
| 0.285 | bex1 | *brain expressed, X-linked 1* |
| 0.27 | il17rd | **interleukin 17 receptor D** |
| 0.249 | ddr2 | *discoidin domain receptor tyrosine kinase 2* |
| 0.243 | sdccag3 | *serologically defined colon cancer antigen 3* |
| 0.24 | islr2 | *immunoglobulin superfamily containing leucine-rich repeat 2* |
| 0.236 | lcor | **ligand dependent nuclear receptor corepressor** |
| 0.231 | sh3kbp1 | *SH3-domain kinase binding protein 1* |
| 0.229 | kctd13 | *potassium channel tetramerisation domain containing 13* |
| 0.22 | arpp-19 | **cyclic AMP phosphoprotein, 19 kD** |
| 0.217 | lilrb1 | **leukocyte immunoglobulin-like receptor, subfamily B (with TM and ITIM domains), member 1** |
| 0.213 | scand1 | *SCAN domain containing 1* |
| 0.211 | adnp | **activity-dependent neuroprotector homeobox** |
| 0.207 | dapp1 | **dual adaptor of phosphotyrosine and 3-phosphoinositides** |
| 0.205 | rhbdl2 | **rhomboid, veinlet-like 2 (Drosophila)** |
| 0.203 | bcap29 | *B-cell receptor-associated protein 29* |
| 0.198 | rbm39 | *RNA binding motif protein 39* |
| 0.195 | asb1 | **ankyrin repeat and SOCS box-containing 1** |
| 0.194 | rcp9 | **calcitonin gene-related peptide-receptor component protein** |
| 0.191 | csf2ra | **colony stimulating factor 2 receptor, alpha, low-affinity (granulocyte-macrophage)** |
| 0.186 | cars | *cysteinyl-tRNA synthetase* |
| 0.185 | vps28 | *vacuolar protein sorting 28 homolog (S. cerevisiae)* |
| 0.178 | wsb2 | **WD repeat and SOCS box-containing 2** |
| 0.177 | fkbp1a | *FK506 binding protein 1A, 12kDa* |
| 0.174 | eif3m | **eukaryotic translation initiation factor 3, subunit M** |
| 0.171 | rtn3 | **reticulon 3** |
| 0.168 | tnfsf14 | **tumor necrosis factor (ligand) superfamily, member 14** |
| 0.167 | med28 | *mediator complex subunit 28* |
| 0.167 | nrcam | **neuronal cell adhesion molecule** |
| 0.166 | tspo | *translocator protein (18kDa)* |
| 0.165 | ubl7 | *ubiquitin-like 7 (bone marrow stromal cell-derived)* |
| 0.162 | kiaa0194 | **kiaa0194 protein** |
| 0.158 | cd68 | **CD68 molecule** |
| 0.157 | rybp | *RING1 and YY1 binding protein* |
| 0.157 | zbtb40 | **zinc finger and BTB domain containing 40** |
| 0.156 | grb2 | *growth factor receptor-bound protein 2* |
| 0.154 | fez1 | *fasciculation and elongation protein zeta 1* |
| 0.153 | cenpj | **centromere protein J** |
| 0.152 | ptges3 | **prostaglandin E synthase 3 (cytosolic)** |
| 0.152 | hmmr | **hyaluronan-mediated motility receptor** |
|  |  |  |
| **GPCR** |  |  |
|  |  |  |
| **LSI Correlation** | **Symbol** | **Definition** |
|  |  |  |
| 0.299 | rgs5 | **regulator of G-protein signaling 5** |
| 0.179 | rcp9 | **calcitonin gene-related peptide-receptor component protein** |
| 0.126 | prkca | **protein kinase C, alpha** |
| 0.111 | gna13 | **guanine nucleotide binding protein (G protein), alpha 13** |
|  |  |  |
| **Aging** |  |  |
|  |  |  |
| **LSI Correlation** | **Symbol** | **Definition** |
|  |  |  |
| 0.216 | adnp | **activity-dependent neuroprotector homeobox** |
| 0.186 | parp4 | **poly (ADP-ribose) polymerase family, member 4** |
| 0.18 | endog | *endonuclease G* |
| 0.162 | mt2a | *metallothionein 2A* |
| 0.161 | app | *amyloid beta (A4) precursor protein* |
| 0.151 | tdg | *thymine-DNA glycosylase* |
| 0.151 | nnt | **nicotinamide nucleotide transhydrogenase** |
| 0.141 | atm | **ataxia telangiectasia mutated** |
| 0.133 | rif1 | **RAP1 interacting factor homolog (yeast)** |
| 0.131 | tmed4 | *transmembrane emp24 protein transport domain containing 4* |
| 0.126 | dclre1c | **DNA cross-link repair 1C (PSO2 homolog, S. cerevisiae)** |
| 0.115 | ahr | **aryl hydrocarbon receptor** |
| 0.113 | rtn3 | **reticulon 3** |
| 0.11 | mbd4 | **methyl-CpG binding domain protein 4** |
| 0.106 | bax | *BCL2-associated X protein* |
| 0.106 | fabp5 | *fatty acid binding protein 5 (psoriasis-associated)* |
| 0.105 | sumo2 | **SMT3 suppressor of mif two 3 homolog 2 (S. cerevisiae)** |
| 0.105 | wbscr22 | *Williams Beuren syndrome chromosome region 22* |
|  |  |  |
| **Juvenile** |  |  |
|  |  |  |
| **LSI Correlation** | **Symbol** | **Definition** |
|  |  |  |
| 0.225 | lep | **leptin (obesity homolog, mouse)** |
| 0.121 | prkdc | **protein kinase, DNA-activated, catalytic polypeptide** |
| 0.118 | zbtb40 | **zinc finger and BTB domain containing 40** |
| 0.113 | rybp | *RING1 and YY1 binding protein* |
|  |  |  |
| **Senescence** |  |  |
|  |  |  |
| **LSI Correlation** | **Symbol** | **Definition** |
|  |  |  |
| 0.303 | dmtf1 | **cyclin D binding myb-like transcription factor 1** |
| 0.254 | tp53inp1 | **tumor protein p53 inducible nuclear protein 1** |
| 0.222 | rif1 | **RAP1 interacting factor homolog (yeast)** |
| 0.205 | tmem158 | *transmembrane protein 158* |
| 0.201 | atm | **ataxia telangiectasia mutated** |
| 0.185 | rabl2a | **RAB, member of RAS oncogene family-like 2A** |
| 0.174 | trrap | **transformation/transcription domain-associated protein** |
| 0.17 | cdkn1c | *cyclin-dependent kinase inhibitor 1C (p57, Kip2)* |
| 0.157 | rpl23 | **ribosomal protein L23** |
| 0.155 | fam40b | **family with sequence similarity 40, member B** |
| 0.154 | dctn6 | *dynactin 6* |
| 0.139 | ezh2 | *enhancer of zeste homolog 2 (Drosophila)* |
| 0.133 | dclre1c | **DNA cross-link repair 1C (PSO2 homolog, S. cerevisiae)** |
| 0.131 | gmcl1 | *germ cell-less homolog 1 (Drosophila)* |
| 0.126 | rims3 | **regulating synaptic membrane exocytosis 3** |
| 0.126 | tceal3 | *transcription elongation factor A (SII)-like 3* |
| 0.124 | parp4 | **poly (ADP-ribose) polymerase family, member 4** |
| 0.122 | apitd1 | *apoptosis-inducing, TAF9-like domain 1* |
| 0.121 | sumo2 | **SMT3 suppressor of mif two 3 homolog 2 (S. cerevisiae)** |
| 0.12 | cdc2 | **cell division cycle 2, G1 to S and G2 to M** |
| 0.12 | ect2 | **epithelial cell transforming sequence 2 oncogene** |
| 0.119 | sbds | **Shwachman-Bodian-Diamond syndrome** |
| 0.117 | rbms1 | *RNA binding motif, single stranded interacting protein 1* |
| 0.116 | pdcd4 | **programmed cell death 4 (neoplastic transformation inhibitor)** |
| 0.115 | cenpf | **centromere protein F, 350/400ka (mitosin)** |
| 0.115 | hif1a | **hypoxia-inducible factor 1, alpha subunit (basic helix-loop-helix transcription factor)** |
| 0.114 | cbx5 | *chromobox homolog 5 (HP1 alpha homolog, Drosophila)* |
| 0.11 | ptges3 | **prostaglandin E synthase 3 (cytosolic)** |
| 0.109 | fanci | **Fanconi anemia, complementation group I** |
| 0.109 | rpl5 | **ribosomal protein L5** |
| 0.107 | rybp | *RING1 and YY1 binding protein* |
| 0.103 | ckap2 | **cytoskeleton associated protein 2** |
|  |  |  |
| **Parkinson's Disease** |  |  |
|  |  |  |
| **LSI Correlation** | **Symbol** | **Definition** |
|  |  |  |
| 0.183 | adnp | **activity-dependent neuroprotector homeobox** |
| 0.164 | mt2a | *metallothionein 2A* |
| 0.16 | arpp-19 | **cyclic AMP phosphoprotein, 19 kD** |
| 0.126 | spast | **spastin, transcript variant 2** |
| 0.124 | pabpn1 | *poly(A) binding protein, nuclear 1* |
| 0.123 | ube2e2 | *ubiquitin-conjugating enzyme E2E 2* |
| 0.12 | hspa8 | **heat shock 70kDa protein 8** |
| 0.106 | chchd6 | *coiled-coil-helix-coiled-coil-helix domain containing 6* |
| 0.105 | sult1a1 | **sulfotransferase family, cytosolic, 1A, phenol-preferring, member 1** |
| 0.101 | sept7 | **septin 7** |
| 0.101 | bcyrn1 | **brain cytoplasmic RNA 1, Bc1 analog (mouse)** |
| 0.101 | rnf150 | **ring finger protein 150** |
| 0.101 | mrpl55 | *mitochondrial ribosomal protein L55* |
| 0.101 | dnm1l | **dynamin 1-like** |
| 0.101 | endog | *endonuclease G* |
| 0.101 | fez1 | *fasciculation and elongation protein zeta 1* |
|  |  |  |
| **Neurodegeneration** |  |  |
|  |  |  |
| **LSI Correlation** | **Symbol** | **Definition** |
|  |  |  |
| 0.233 | pqbp1 | *polyglutamine binding protein 1* |
| 0.232 | spast | **spastin, transcript variant 2** |
| 0.221 | pabpn1 | *poly(A) binding protein, nuclear 1* |
| 0.212 | adnp | **activity-dependent neuroprotector homeobox** |
| 0.201 | rtn3 | **reticulon 3** |
| 0.197 | bcyrn1 | **brain cytoplasmic RNA 1, Bc1 analog (mouse)** |
| 0.194 | arpp-19 | **cyclic AMP phosphoprotein, 19 kD** |
| 0.188 | mcoln1 | *mucolipin 1* |
| 0.18 | mt2a | *metallothionein 2A* |
| 0.175 | slc44a4 | **solute carrier family 44, member 4** |
| 0.163 | endog | *endonuclease G* |
| 0.16 | app | *amyloid beta (A4) precursor protein* |
| 0.153 | gsk3b | *glycogen synthase kinase 3 beta* |
| 0.15 | sptlc1 | **serine palmitoyltransferase, long chain base subunit 1** |
| 0.143 | aph1a | **anterior pharynx defective 1 homolog A (C. elegans)** |
| 0.141 | cd68 | **CD68 molecule** |
| 0.139 | sacs | **spastic ataxia of Charlevoix-Saguenay (sacsin)** |
| 0.131 | dnm1l | **dynamin 1-like** |
| 0.13 | surf1 | *surfeit 1* |
| 0.127 | hspa8 | **heat shock 70kDa protein 8** |
| 0.126 | kiaa0194 | **kiaa0194 protein** |
| 0.126 | atm | **ataxia telangiectasia mutated** |
| 0.125 | tmed4 | *transmembrane emp24 protein transport domain containing 4* |
| 0.122 | klhl7 | **kelch-like 7 (Drosophila)** |
| 0.118 | cdk5rap2 | **CDK5 regulatory subunit associated protein 2** |
| 0.114 | dopey2 | **dopey family member 2** |
| 0.113 | rpl14 | **ribosomal protein L14** |
| 0.112 | bax | *BCL2-associated X protein* |
| 0.111 | tspan7 | *tetraspanin 7* |
| 0.11 | il18 | **interleukin 18 (interferon-gamma-inducing factor)** |
| 0.108 | fez1 | *fasciculation and elongation protein zeta 1* |
| 0.105 | msto1 | **misato homolog 1 (Drosophila)** |
|  |  |  |
| **Huntington's Disease** |  |  |
|  |  |  |
| **LSI Correlation** | **Symbol** | **Definition** |
|  |  |  |
| 0.301 | kiaa0194 | **kiaa0194 protein** |
| 0.247 | pqbp1 | *polyglutamine binding protein 1* |
| 0.199 | rpl14 | **ribosomal protein L14** |
| 0.183 | pabpn1 | *poly(A) binding protein, nuclear 1* |
| 0.17 | tspan7 | *tetraspanin 7* |
| 0.124 | snapc1 | small nuclear RNA activating complex, polypeptide 1, 43kDa |
| 0.107 | btaf1 | **BTAF1 RNA polymerase II, B-TFIID transcription factor-associated, 170kDa** |
| 0.107 | spast | **spastin, transcript variant 2** |
| 0.106 | med30 | *mediator complex subunit 30* |
| 0.106 | snapc4 | *small nuclear RNA activating complex, polypeptide 4, 190kDa* |
|  |  |  |
| **Cognitive impairment** |  |  |
|  |  |  |
| **LSI Correlation** | **Symbol** | **Definition** |
|  |  |  |
| 0.291 | dopey2 | **dopey family member 2** |
| 0.289 | spast | **spastin, transcript variant 2** |
| 0.249 | wbscr22 | *Williams Beuren syndrome chromosome region 22* |
| 0.245 | pqbp1 | *polyglutamine binding protein 1* |
| 0.243 | gtf2ird2b | *GTF2I repeat domain containing 2B* |
| 0.239 | slc44a4 | **solute carrier family 44, member 4** |
| 0.238 | tsc1 | **tuberous sclerosis 1** |
| 0.237 | tspan7 | *tetraspanin 7* |
| 0.219 | adnp | **activity-dependent neuroprotector homeobox** |
| 0.203 | bcyrn1 | **brain cytoplasmic RNA 1, Bc1 analog (mouse)** |
| 0.197 | app | *amyloid beta (A4) precursor protein* |
| 0.189 | fam58a | *family with sequence similarity 58, member A* |
| 0.185 | rnaseh2b | **ribonuclease H2, subunit B** |
| 0.18 | fktn | **fukutin** |
| 0.176 | sacs | **spastic ataxia of Charlevoix-Saguenay (sacsin)** |
| 0.173 | c7orf11 | *chromosome 7 open reading frame 11* |
| 0.172 | mcoln1 | *mucolipin 1* |
| 0.172 | kif21a | **kinesin family member 21A** |
| 0.172 | sbds | **Shwachman-Bodian-Diamond syndrome** |
| 0.169 | rtn3 | **reticulon 3** |
| 0.161 | fkrp | *fukutin related protein* |
| 0.155 | fez1 | *fasciculation and elongation protein zeta 1* |
| 0.152 | arpp-19 | **cyclic AMP phosphoprotein, 19 kD** |
| 0.149 | mt2a | *metallothionein 2A* |
| 0.146 | nptn | **neuroplastin** |
| 0.138 | wrb | **tryptophan rich basic protein** |
| 0.136 | aph1a | **anterior pharynx defective 1 homolog A (C. elegans)** |
| 0.131 | cdk5rap2 | **CDK5 regulatory subunit associated protein 2** |
| 0.131 | ccdc34 | *coiled-coil domain containing 34* |
| 0.128 | prepl | **prolyl endopeptidase-like** |
| 0.12 | cdan1 | **congenital dyserythropoietic anemia, type I** |
| 0.115 | aire | **autoimmune regulator (autoimmune polyendocrinopathy candidiasis ectodermal dystrophy)** |
| 0.115 | surf1 | *surfeit 1* |
| 0.111 | pabpn1 | *poly(A) binding protein, nuclear 1* |
| 0.105 | rpl14 | **ribosomal protein L14** |
| 0.104 | asb1 | **ankyrin repeat and SOCS box-containing 1** |
| 0.104 | tfap2b | *transcription factor AP-2 beta (activating enhancer binding protein 2 beta)* |
| 0.103 | larp4 | **La ribonucleoprotein domain family, member 4** |
| 0.101 | gsk3b | *glycogen synthase kinase 3 beta* |
|  |  |  |
| **Amyotrophic lateral sclerosis** | |  |
|  |  |  |
| **LSI Correlation** | **Symbol** | **Definition** |
|  |  |  |
| 0.283 | mt2a | *metallothionein 2A* |
| 0.203 | spast | **spastin, transcript variant 2** |
| 0.192 | tsc1 | **tuberous sclerosis 1** |
| 0.188 | islr2 | *immunoglobulin superfamily containing leucine-rich repeat 2* |
| 0.187 | mt1x | *metallothionein 1X* |
| 0.183 | klhl7 | **kelch-like 7 (Drosophila)** |
| 0.157 | nola1 | *nucleolar protein family A, member 1 (H/ACA small nucleolar RNPs)* |
| 0.154 | snrpd3 | *small nuclear ribonucleoprotein D3 polypeptide 18kDa* |
| 0.142 | clec16a | **C-type lectin domain family 16, member A** |
| 0.131 | adnp | **activity-dependent neuroprotector homeobox** |
| 0.127 | bax | *BCL2-associated X protein* |
| 0.127 | pabpn1 | *poly(A) binding protein, nuclear 1* |
| 0.127 | zbtb43 | *zinc finger and BTB domain containing 43* |
| 0.125 | fez1 | *fasciculation and elongation protein zeta 1* |
| 0.125 | lsm5 | **LSM5 homolog, U6 small nuclear RNA associated (S. cerevisiae)** |
| 0.119 | ccny | **cyclin Y** |
| 0.115 | kif3b | **kinesin family member 3B** |
| 0.111 | nrcam | **neuronal cell adhesion molecule** |
| 0.11 | slc44a4 | **solute carrier family 44, member 4** |
| 0.108 | pycr2 | *pyrroline-5-carboxylate reductase family, member 2* |
| 0.108 | dll3 | *delta-like 3 (Drosophila)* |
| 0.107 | pcna | **proliferating cell nuclear antigen** |
| 0.106 | bcyrn1 | **brain cytoplasmic RNA 1, Bc1 analog (mouse)** |
| 0.101 | pqbp1 | *polyglutamine binding protein 1* |
|  |  |  |
| **Alzheimer's Disease** |  |  |
|  |  |  |
| **LSI Correlation** | **Symbol** | **Definition** |
|  |  |  |
| 0.446 | aph1a | **anterior pharynx defective 1 homolog A (C. elegans)** |
| 0.375 | rtn3 | **reticulon 3** |
| 0.311 | bcyrn1 | **brain cytoplasmic RNA 1, Bc1 analog (mouse)** |
| 0.214 | adnp | **activity-dependent neuroprotector homeobox** |
| 0.169 | arpp-19 | **cyclic AMP phosphoprotein, 19 kD** |
| 0.165 | gsk3b | *glycogen synthase kinase 3 beta* |
| 0.153 | cd68 | **CD68 molecule** |
| 0.149 | app | *amyloid beta (A4) precursor protein* |
| 0.123 | sfrs12 | **splicing factor, arginine/serine-rich 12** |
| 0.121 | grb2 | *growth factor receptor-bound protein 2* |
| 0.119 | ppp2r5d | *protein phosphatase 2, regulatory subunit B', delta isoform* |
| 0.118 | cdc2 | **cell division cycle 2, G1 to S and G2 to M** |
| 0.114 | mt2a | *metallothionein 2A* |
| 0.113 | ccny | *cyclin Y* |
| 0.11 | cdk5rap2 | **CDK5 regulatory subunit associated protein 2** |
| 0.105 | fez1 | *fasciculation and elongation protein zeta 1* |
| 0.104 | mgea5 | **meningioma expressed antigen 5 (hyaluronidase)** |
| 0.102 | spast | **spastin, transcript variant 2** |
| 0.101 | osbpl8 | **oxysterol binding protein-like 8** |
|  |  |  |
| **Tourette syndrome** |  |  |
|  |  |  |
| **LSI Correlation** | **Symbol** | **Definition** |
|  |  |  |
| 0.143 | tfap2b | *transcription factor AP-2 beta (activating enhancer binding protein 2 beta)* |
| 0.123 | aph1a | **anterior pharynx defective 1 homolog A (C. elegans)** |
| 0.117 | app | *amyloid beta (A4) precursor protein* |
|  |  |  |
| **Spina bifida** |  |  |
|  |  |  |
| **LSI Correlation** | **Symbol** | **Definition** |
|  |  |  |
| 0.225 | crabp1 | *cellular retinoic acid binding protein 1* |
| 0.146 | prkca | **protein kinase C, alpha** |
| 0.134 | six3 | *sine oculis homeobox homolog 3 (Drosophila)* |
| 0.128 | sult1a1 | **sulfotransferase family, cytosolic, 1A, phenol-preferring, member 1** |
| 0.122 | rttn | **rotatin** |
| 0.122 | pycr2 | *pyrroline-5-carboxylate reductase family, member 2* |
|  |  |  |
| **Measles** |  |  |
|  |  |  |
| **LSI Correlation** | **Symbol** | **Definition** |
|  |  |  |
| 0.139 | bcap29 | *B-cell receptor-associated protein 29* |
| 0.134 | edem3 | **ER degradation enhancer, mannosidase alpha-like 3** |
| 0.115 | tmed4 | *transmembrane emp24 protein transport domain containing 4* |
| 0.114 | ict1 | *immature colon carcinoma transcript 1* |
|  |  |  |
| **Asthma** |  |  |
|  |  |  |
| **LSI Correlation** | **Symbol** | **Definition** |
|  |  |  |
| 0.193 | il18 | **interleukin 18 (interferon-gamma-inducing factor)** |
| 0.191 | csf2ra | **colony stimulating factor 2 receptor, alpha, low-affinity (granulocyte-macrophage)** |
| 0.174 | pla2g2d | **phospholipase A2, group IID** |
| 0.167 | ccbe1 | **collagen and calcium binding EGF domains 1** |
| 0.164 | ccny | *cyclin Y* |
|  |  |  |
| **Achondroplasia** |  |  |
|  |  |  |
| **Correlation** | **Symbol** | **Definition** |
|  |  |  |
| 0.193 | il17rd | **interleukin 17 receptor D** |
| 0.157 | minpp1 | **multiple inositol polyphosphate histidine phosphatase, 1** |
| 0.108 | papss1 | **3'-phosphoadenosine 5'-phosphosulfate synthase 1** |
|  |  |  |
| **ADHD** |  |  |
|  |  |  |
| **Correlation** | **Symbol** | **Definition** |
|  |  |  |
| 0.163 | tfap2b | *transcription factor AP-2 beta (activating enhancer binding protein 2 beta)* |
