# Supplementary material for: Minimal Peroxide Exposure of Neuronal Cells Induces Multifaceted Adaptive Responses
Source: PLoS One. 2010 Dec 17;5(12):e14352. doi: 10.1371/journal.pone.0014352 (PMC3003681; doi:10.1371/journal.pone.0014352)
Supplement: Table S3 — MeCh-significantly regulated genes after 2 hours of stimulation in the control state SH-SY5Y cells. Each significantly regulated gene is described via its accession number (ACCESSION), Gene Symbol (SYMBOL), Illumina array transcript designation (TRANSCRIPT). For each gene the z-ratio of expression compared to control vehicle-treated cells after 2 hours of ligand stimulation is displayed (CTL MeCh 2). (0.94 MB DOC) [file pone.0014352.s010.doc]

**Table S3. MeCh-significantly regulated genes after 2 hours of stimulation in the control state SH-SY5Y cells**. Each significantly regulated gene is described via its accession number (ACCESSION), Gene Symbol (SYMBOL), Illumina array transcript designation (TRANSCRIPT). For each gene the z-ratio of expression compared to control vehicle-treated cells after 2 hours of ligand stimulation is displayed (CTL MeCh 2).

| **ACCESSION** | **SYMBOL** | **TRANSCRIPT** | **CTL MeCh 2** |
| --- | --- | --- | --- |
| NM_001964.2 | EGR1 | ILMN_20932 | 17.39 |
| NM_001554.3 | CYR61 | ILMN_21983 | 15.53 |
| NM_001005474.1 | NFKBIZ | ILMN_16362 | 6.83 |
| NM_000584.2 | IL8 | ILMN_179575 | 6.52 |
| XM_944439.2 | LOC653994 | ILMN_38572 | 6.31 |
| NM_001300.4 | KLF6 | ILMN_17961 | 5.54 |
| NM_001008490.1 | KLF6 | ILMN_12381 | 5.5 |
| NM_005324.3 | H3F3B | ILMN_26885 | 5.1 |
| NM_004417.2 | DUSP1 | ILMN_20700 | 4.15 |
| NM_001924.2 | GADD45A | ILMN_17355 | 3.93 |
| NM_002566.4 | P2RY11 | ILMN_12237 | 3.84 |
| NM_014330.2 | PPP1R15A | ILMN_1024 | 3.74 |
| NM_033138.2 | CALD1 | ILMN_29896 | 3.72 |
| NM_020310.2 | MNT | ILMN_21283 | 3.72 |
| XM_941665.2 | LOC387763 | ILMN_43061 | 3.71 |
| NM_182776.1 | MCM7 | ILMN_1133 | 3.64 |
| NM_014817.3 | KIAA0644 | ILMN_164846 | 3.59 |
| NM_001080453.1 | INTS1 | ILMN_173681 | 3.42 |
| XM_931359.2 | LOC338758 | ILMN_37634 | 3.33 |
| NM_016028.4 | SUV420H1 | ILMN_29861 | 3.31 |
| NM_003461.4 | ZYX | ILMN_2137 | 3.29 |
| NM_001013258.1 | ZNF789 | ILMN_11535 | 3.27 |
| NM_001033506.1 | CSTF3 | ILMN_27049 | 3.23 |
| NM_001013685.1 | LOC401357 | ILMN_29013 | 3.2 |
| NM_006885.3 | ZFHX3 | ILMN_174159 | 3.13 |
| NM_002166.4 | ID2 | ILMN_28481 | 3.12 |
| NM_014380.1 | NGFRAP1 | ILMN_7162 | 3.08 |
| NM_002874.3 | RAD23B | ILMN_19346 | 3.02 |
| NM_005627.2 | SGK | ILMN_2451 | 3 |
| NM_000787.3 | DBH | ILMN_25962 | 3 |
| NM_001101.2 | ACTB | ILMN_2565 | 2.99 |
| XM_001126418.1 | LOC727935 | ILMN_181411 | 2.97 |
| XM_926036.1 | LOC653103 | ILMN_32029 | 2.94 |
| NM_012215.2 | MGEA5 | ILMN_11399 | 2.92 |
| NM_018697.3 | LANCL2 | ILMN_920 | 2.9 |
| NM_005841.1 | SPRY1 | ILMN_6281 | 2.85 |
| XR_019449.1 | LOC644422 | ILMN_166674 | 2.83 |
| XM_001131589.1 | LOC643446 | ILMN_170528 | 2.83 |
| NM_001614.2 | ACTG1 | ILMN_24353 | 2.83 |
| NM_001280.1 | CIRBP | ILMN_24327 | 2.81 |
| NM_002228.3 | JUN | ILMN_7746 | 2.79 |
| NM_005916.3 | MCM7 | ILMN_1986 | 2.79 |
| NM_001017421.1 | FKSG30 | ILMN_2393 | 2.78 |
| XM_001132569.1 | LOC730130 | ILMN_162537 | 2.76 |
| NR_000011.1 | SNORA70 | ILMN_7210 | 2.75 |
| NM_001008237.1 | TTC32 | ILMN_4829 | 2.73 |
| NM_001924.2 | GADD45A | ILMN_17355 | 2.73 |
| NM_199043.1 | C14orf102 | ILMN_22442 | 2.72 |
| NM_173042.2 | IL18BP | ILMN_30884 | 2.7 |
| NM_178517.3 | PIGW | ILMN_162681 | 2.69 |
| NM_001614.2 | ACTG1 | ILMN_24353 | 2.69 |
| NM_006372.3 | SYNCRIP | ILMN_28470 | 2.68 |
| NM_001013699.1 | LOC440093 | ILMN_19743 | 2.63 |
| NM_001040456.1 | RHBDD2 | ILMN_168345 | 2.58 |
| NM_001003725.1 | WDR68 | ILMN_19537 | 2.56 |
| NM_005560.3 | LAMA5 | ILMN_12588 | 2.55 |
| NM_006009.2 | TUBA1A | ILMN_1089 | 2.54 |
| NM_003749.2 | IRS2 | ILMN_167991 | 2.52 |
| NM_033419.3 | PERLD1 | ILMN_12215 | 2.49 |
| NM_020801.1 | ARRDC3 | ILMN_22538 | 2.49 |
| NM_021960.3 | MCL1 | ILMN_18397 | 2.48 |
| NM_002673.3 | PLXNB1 | ILMN_22628 | 2.48 |
| NM_014281.3 | PUF60 | ILMN_14897 | 2.48 |
| NM_001012626.1 | LOC285074 | ILMN_21153 | 2.47 |
| NM_206953.1 | PRAME | ILMN_8154 | 2.47 |
| NM_015995.2 | KLF13 | ILMN_16226 | 2.47 |
| NM_005654.4 | NR2F1 | ILMN_177945 | 2.47 |
| XR_019152.1 | LOC644584 | ILMN_163817 | 2.45 |
| NM_004126.3 | GNG11 | ILMN_8981 | 2.44 |
| NM_182492.1 | LRP5L | ILMN_650 | 2.42 |
| NM_006925.3 | SFRS5 | ILMN_34497 | 2.42 |
| XM_497182.3 | LOC644670 | ILMN_38310 | 2.41 |
| NM_007350.3 | PHLDA1 | ILMN_7104 | 2.4 |
| XM_938988.1 | LOC402221 | ILMN_35678 | 2.4 |
| NM_005334.2 | HCFC1 | ILMN_24237 | 2.39 |
| NM_148957.2 | TNFRSF19 | ILMN_28684 | 2.37 |
| NM_003486.5 | SLC7A5 | ILMN_25446 | 2.37 |
| NM_001040456.1 | RHBDD2 | ILMN_168345 | 2.36 |
| NM_080677.1 | DYNLL2 | ILMN_28971 | 2.35 |
| NM_003131.2 | SRF | ILMN_22299 | 2.35 |
| NM_001453.1 | FOXC1 | ILMN_23624 | 2.35 |
| NM_014694.2 | ADAMTSL2 | ILMN_697 | 2.34 |
| XM_001129527.1 | KLF11 | ILMN_168976 | 2.31 |
| NM_006720.3 | ABLIM1 | ILMN_21737 | 2.3 |
| XM_940903.2 | ZC3H5 | ILMN_40646 | 2.29 |
| NM_004516.2 | ILF3 | ILMN_12252 | 2.27 |
| NM_001024070.1 | GCH1 | ILMN_23648 | 2.25 |
| NM_006925.3 | SFRS5 | ILMN_34497 | 2.25 |
| NM_001080477.1 | ODZ3 | ILMN_179907 | 2.24 |
| NM_003110.4 | SP2 | ILMN_7882 | 2.23 |
| NM_015447.1 | CAMSAP1 | ILMN_815 | 2.23 |
| NM_021009.3 | UBC | ILMN_8850 | 2.22 |
| NM_178014.2 | TUBB | ILMN_23399 | 2.21 |
| XM_927980.1 | LOC643319 | ILMN_41137 | 2.2 |
| NM_002213.3 | ITGB5 | ILMN_24189 | 2.2 |
| NM_015949.2 | C7orf20 | ILMN_23467 | 2.2 |
| NM_016287.3 | HP1BP3 | ILMN_29502 | 2.2 |
| NM_002972.1 | SBF1 | ILMN_22729 | 2.2 |
| NM_198836.1 | ACACA | ILMN_9534 | 2.19 |
| NM_201557.2 | FHL2 | ILMN_42988 | 2.18 |
| NM_001287.3 | CLCN7 | ILMN_8600 | 2.18 |
| XM_944429.1 | LOC653994 | ILMN_38337 | 2.17 |
| NM_020724.1 | RNF150 | ILMN_26801 | 2.17 |
| NM_006731.2 | FKTN | ILMN_6512 | 2.17 |
| NM_014665.1 | LRRC14 | ILMN_166093 | 2.16 |
| NM_133465.2 | KIAA1958 | ILMN_17353 | 2.15 |
| NM_006739.3 | MCM5 | ILMN_20107 | 2.14 |
| NM_015226.1 | CLEC16A | ILMN_19348 | 2.14 |
| NM_172251.1 | MRPL54 | ILMN_1467 | 2.14 |
| NM_022308.1 | ICA1 | ILMN_12918 | 2.14 |
| NM_003086.2 | SNAPC4 | ILMN_180505 | 2.13 |
| NM_003076.3 | SMARCD1 | ILMN_16093 | 2.12 |
| NM_007372.2 | DDX42 | ILMN_1009 | 2.11 |
| NM_013262.3 | MYLIP | ILMN_178445 | 2.11 |
| NM_002936.3 | RNASEH1 | ILMN_17680 | 2.09 |
| NR_002450.1 | SNORD68 | ILMN_25967 | 2.08 |
| NM_020664.3 | DECR2 | ILMN_7935 | 2.08 |
| NM_002473.3 | MYH9 | ILMN_183555 | 2.08 |
| NM_021009.1 | UBC | ILMN_160470 | 2.08 |
| NM_000876.2 | IGF2R | ILMN_177695 | 2.07 |
| NM_014014.2 | ASCC3L1 | ILMN_18834 | 2.06 |
| NM_000093.3 | COL5A1 | ILMN_31902 | 2.05 |
| NM_017566.2 | KLHDC4 | ILMN_8527 | 2.05 |
| NM_182972.2 | IRF2BP2 | ILMN_5645 | 2.05 |
| NM_015516.3 | TSKU | ILMN_29523 | 2.03 |
| NM_020822.1 | KCNT1 | ILMN_21599 | 2.03 |
| NM_001567.2 | INPPL1 | ILMN_20903 | 2.03 |
| NM_003119.2 | SPG7 | ILMN_26332 | 2.03 |
| NM_001275.3 | CHGA | ILMN_23390 | 2.03 |
| NM_078474.2 | TM2D3 | ILMN_28191 | 2.02 |
| NM_001003795.2 | GTF2IRD2B | ILMN_12811 | 2.02 |
| NM_001014432.1 | AKT1 | ILMN_4841 | 2.02 |
| NM_130783.3 | TSPAN18 | ILMN_14181 | 2.01 |
| NM_005112.4 | WDR1 | ILMN_14401 | 2.01 |
| NM_054016.1 | FUSIP1 | ILMN_30145 | 2 |
| NM_001613.1 | ACTA2 | ILMN_6588 | 2 |
| NM_015684.2 | ATP5S | ILMN_26667 | 1.99 |
| NM_006047.4 | RBM12 | ILMN_183773 | 1.99 |
| NM_004424.3 | E4F1 | ILMN_23848 | 1.99 |
| XR_018316.1 | LOC648024 | ILMN_174671 | 1.99 |
| NM_005909.3 | MAP1B | ILMN_28251 | 1.98 |
| NM_001020820.1 | MYADM | ILMN_8340 | 1.97 |
| NM_006275.4 | SFRS6 | ILMN_24964 | 1.97 |
| NM_002477.1 | MYL5 | ILMN_21416 | 1.96 |
| NM_004228.4 | PSCD2 | ILMN_26962 | 1.95 |
| NM_021737.1 | CLCN6 | ILMN_6195 | 1.95 |
| NM_152470.2 | RNF165 | ILMN_14516 | 1.95 |
| NM_013275.4 | ANKRD11 | ILMN_28595 | 1.95 |
| NM_001012516.1 | ITM2C | ILMN_27531 | 1.95 |
| NM_175847.1 | PTBP1 | ILMN_20407 | 1.95 |
| XM_940438.2 | LOC654174 | ILMN_44671 | 1.94 |
| NM_014772.1 | KIAA0427 | ILMN_182540 | 1.94 |
| NM_024100.3 | WDR18 | ILMN_23247 | 1.94 |
| NM_201559.2 | FOXO3 | ILMN_15525 | 1.94 |
| NM_014374.1 | REPIN1 | ILMN_1054 | 1.94 |
| NM_005245.3 | FAT | ILMN_24617 | 1.92 |
| NM_006185.2 | NUMA1 | ILMN_25058 | 1.92 |
| NM_002333.1 | LRP3 | ILMN_12327 | 1.92 |
| NM_005494.2 | DNAJB6 | ILMN_26714 | 1.91 |
| NM_000617.1 | SLC11A2 | ILMN_10129 | 1.9 |
| NM_005157.3 | ABL1 | ILMN_4033 | 1.9 |
| NM_002048.1 | GAS1 | ILMN_175833 | 1.89 |
| NM_201440.1 | PPHLN1 | ILMN_4445 | 1.89 |
| NM_020175.1 | DUS3L | ILMN_3805 | 1.89 |
| NM_006110.1 | CD2BP2 | ILMN_9406 | 1.89 |
| NM_004968.2 | ICA1 | ILMN_29651 | 1.89 |
| NM_182705.2 | FAM101B | ILMN_1388 | 1.88 |
| XM_926322.1 | LOC653171 | ILMN_41501 | 1.88 |
| NM_020307.2 | CCNL1 | ILMN_14683 | 1.88 |
| NM_018708.2 | FEM1A | ILMN_2838 | 1.88 |
| NM_006988.3 | ADAMTS1 | ILMN_11081 | 1.87 |
| NM_005920.2 | MEF2D | ILMN_3465 | 1.87 |
| XM_937850.1 | LOC285176 | ILMN_43277 | 1.87 |
| NM_022720.5 | DGCR8 | ILMN_1552 | 1.87 |
| NM_001003795.2 | GTF2IRD2B | ILMN_12811 | 1.87 |
| NM_201997.1 | SF1 | ILMN_2017 | 1.86 |
| NM_014614.1 | PSME4 | ILMN_164803 | 1.86 |
| NM_017833.2 | C21orf55 | ILMN_6782 | 1.86 |
| NM_198839.1 | ACACA | ILMN_176065 | 1.85 |
| NM_170721.1 | MSI2 | ILMN_25750 | 1.84 |
| NR_002196.1 | H19 | ILMN_18538 | 1.84 |
| NM_006997.2 | TACC2 | ILMN_16130 | 1.82 |
| NM_004090.2 | DUSP3 | ILMN_180655 | 1.81 |
| NM_002819.3 | PTBP1 | ILMN_20993 | 1.81 |
| NM_000146.3 | FTL | ILMN_10967 | 1.81 |
| NM_213606.1 | SLC16A12 | ILMN_28607 | 1.81 |
| NM_015039.2 | NMNAT2 | ILMN_7389 | 1.8 |
| NM_178831.4 | GATS | ILMN_18755 | 1.79 |
| NM_001006610.1 | SIAH1 | ILMN_9220 | 1.79 |
| NM_152280.2 | SYT11 | ILMN_23967 | 1.79 |
| XM_495939.3 | KIAA1545 | ILMN_40920 | 1.78 |
| NM_012384.2 | GMEB2 | ILMN_7174 | 1.78 |
| NM_152263.2 | TPM3 | ILMN_5807 | 1.78 |
| XM_934471.1 | LOC399942 | ILMN_32988 | 1.78 |
| NM_006047.4 | RBM12 | ILMN_183773 | 1.77 |
| NM_017635.3 | SUV420H1 | ILMN_174505 | 1.77 |
| NM_024900.3 | PHF17 | ILMN_1535 | 1.77 |
| NM_006646.4 | WASF3 | ILMN_180336 | 1.77 |
| NM_003565.1 | ULK1 | ILMN_2158 | 1.77 |
| NM_020226.3 | PRDM8 | ILMN_22013 | 1.76 |
| NM_178326.2 | ATG4B | ILMN_24355 | 1.76 |
| NM_018715.1 | RCC2 | ILMN_2123 | 1.76 |
| NM_004618.3 | TOP3A | ILMN_167915 | 1.75 |
| NM_006114.1 | TOMM40 | ILMN_29459 | 1.75 |
| NM_016481.3 | C9orf156 | ILMN_12842 | 1.74 |
| XM_001133202.1 | KIAA0363 | ILMN_166209 | 1.74 |
| NM_002202.1 | ISL1 | ILMN_25965 | 1.74 |
| NM_020695.3 | REXO1 | ILMN_20923 | 1.73 |
| NM_007353.2 | GNA12 | ILMN_11787 | 1.73 |
| NM_001004322.1 | FLJ38717 | ILMN_13488 | 1.73 |
| NM_005481.2 | MED16 | ILMN_24784 | 1.73 |
| XM_926231.1 | P704P | ILMN_36679 | 1.73 |
| NM_004566.2 | PFKFB3 | ILMN_163833 | 1.73 |
| NM_004321.4 | KIF1A | ILMN_22617 | 1.73 |
| NM_005801.3 | EIF1 | ILMN_3037 | 1.73 |
| NM_014914.2 | CENTG2 | ILMN_138241 | 1.72 |
| NM_006465.2 | ARID3B | ILMN_4032 | 1.72 |
| NM_002087.2 | GRN | ILMN_18655 | 1.72 |
| NM_023009.4 | MARCKSL1 | ILMN_17241 | 1.72 |
| NM_005497.3 | GJC1 | ILMN_3556 | 1.72 |
| NM_001018052.1 | POLR3H | ILMN_8571 | 1.71 |
| NM_014913.2 | ADNP2 | ILMN_6906 | 1.71 |
| NM_003195.4 | TCEA2 | ILMN_10248 | 1.7 |
| NM_199361.1 | TPD52L2 | ILMN_24077 | 1.7 |
| NM_001006610.1 | SIAH1 | ILMN_9220 | 1.7 |
| NM_006374.3 | STK25 | ILMN_6139 | 1.7 |
| NM_016644.1 | PRR16 | ILMN_4368 | 1.7 |
| NM_001008408.3 | RBM33 | ILMN_165407 | 1.69 |
| NM_005318.2 | H1F0 | ILMN_139403 | 1.69 |
| NM_014853.2 | SGSM2 | ILMN_9226 | 1.69 |
| XM_945544.1 | UBE2Z | ILMN_137054 | 1.68 |
| NM_001089.1 | ABCA3 | ILMN_18800 | 1.68 |
| NM_013291.2 | CPSF1 | ILMN_22094 | 1.68 |
| NM_001048201.1 | UHRF1 | ILMN_162952 | 1.68 |
| NM_031263.1 | HNRPK | ILMN_16515 | 1.68 |
| NM_003496.1 | TRRAP | ILMN_18258 | 1.67 |
| XM_926402.1 | LOC643031 | ILMN_40435 | 1.67 |
| XM_944915.1 | PTP4A2 | ILMN_137656 | 1.66 |
| NM_001025091.1 | ABCF1 | ILMN_179040 | 1.66 |
| NM_001024071.1 | GCH1 | ILMN_14690 | 1.65 |
| NM_006503.2 | PSMC4 | ILMN_4085 | 1.65 |
| NM_015908.4 | ARS2 | ILMN_19647 | 1.65 |
| NM_032485.4 | MCM8 | ILMN_11171 | 1.65 |
| NM_198679.1 | RAPGEF1 | ILMN_177243 | 1.64 |
| NM_033446.1 | FAM125B | ILMN_20760 | 1.63 |
| NM_002996.3 | CX3CL1 | ILMN_9636 | 1.63 |
| NM_006796.1 | AFG3L2 | ILMN_29564 | 1.63 |
| NM_020856.2 | TSHZ3 | ILMN_19320 | 1.63 |
| NM_001008529.1 | MXRA7 | ILMN_8451 | 1.63 |
| NM_005561.2 | LAMP1 | ILMN_27826 | 1.63 |
| NM_198267.1 | ING3 | ILMN_23155 | 1.62 |
| NM_000551.2 | VHL | ILMN_21046 | 1.62 |
| NM_032527.3 | ZGPAT | ILMN_23696 | 1.62 |
| NM_022489.2 | C14orf173 | ILMN_41230 | 1.62 |
| NM_015306.1 | USP24 | ILMN_309418 | 1.61 |
| NM_030665.3 | RAI1 | ILMN_176671 | 1.61 |
| NM_001111.3 | ADAR | ILMN_20593 | 1.61 |
| XM_936103.1 | LOC642033 | ILMN_33652 | 1.6 |
| NM_001006946.1 | SDC1 | ILMN_169032 | 1.6 |
| NM_014747.2 | RIMS3 | ILMN_21581 | 1.6 |
| NM_003565.1 | ULK1 | ILMN_2158 | 1.6 |
| NM_024083.2 | ASPSCR1 | ILMN_9446 | 1.6 |
| NM_001001679.1 | FLJ41423 | ILMN_1487 | 1.59 |
| NM_133369.2 | UNC5A | ILMN_182275 | 1.59 |
| NM_002827.2 | PTPN1 | ILMN_5417 | 1.59 |
| NM_198055.1 | MZF1 | ILMN_8368 | 1.59 |
| NM_015330.1 | SPECC1L | ILMN_168707 | 1.59 |
| NM_014897.1 | ZNF652 | ILMN_3215 | 1.59 |
| NM_021569.2 | GRIN1 | ILMN_28874 | 1.57 |
| NM_152227.1 | SNX5 | ILMN_6733 | 1.57 |
| NM_005190.3 | CCNC | ILMN_11667 | 1.57 |
| NM_003583.2 | DYRK2 | ILMN_3688 | 1.57 |
| NM_002771.2 | PRSS3 | ILMN_19426 | 1.57 |
| NM_032195.1 | SON | ILMN_8462 | 1.56 |
| NM_153044.1 | FLJ35801 | ILMN_23944 | 1.56 |
| NM_001040439.1 | MAPK8IP3 | ILMN_174436 | 1.56 |
| NM_013336.3 | SEC61A1 | ILMN_9397 | 1.56 |
| NM_024077.3 | SECISBP2 | ILMN_19156 | 1.55 |
| NM_014268.1 | MAPRE2 | ILMN_8637 | 1.55 |
| NM_014376.2 | CYFIP2 | ILMN_15047 | 1.55 |
| NM_182917.2 | EIF4G1 | ILMN_5831 | 1.55 |
| NM_014603.1 | CDR2L | ILMN_26231 | 1.55 |
| NM_006958.2 | ZNF16 | ILMN_17198 | 1.54 |
| NM_001010864.1 | LOC196752 | ILMN_1138 | 1.54 |
| NM_001259.5 | CDK6 | ILMN_178275 | 1.54 |
| NM_177965.2 | C8orf37 | ILMN_11118 | 1.54 |
| XM_942509.2 | LOC652826 | ILMN_34282 | 1.53 |
| NM_002093.2 | GSK3B | ILMN_7421 | 1.53 |
| NM_005678.3 | SNURF | ILMN_17485 | 1.53 |
| NM_001025091.1 | ABCF1 | ILMN_179040 | 1.53 |
| XR_017538.1 | LOC653479 | ILMN_167529 | 1.52 |
| NM_003564.1 | TAGLN2 | ILMN_14546 | 1.52 |
| XM_939186.2 | GPSM1 | ILMN_40984 | 1.52 |
| NM_007200.3 | AKAP13 | ILMN_28017 | 1.51 |
| NM_172249.1 | CSF2RA | ILMN_5061 | 1.5 |
| NM_145863.1 | ASB3 | ILMN_24959 | -1.5 |
| NM_003449.3 | TRIM26 | ILMN_22923 | -1.51 |
| NM_175069.1 | APTX | ILMN_7416 | -1.51 |
| NM_015360.3 | SKIV2L2 | ILMN_8825 | -1.51 |
| NM_018659.2 | CYTL1 | ILMN_7848 | -1.52 |
| NM_020918.3 | GPAM | ILMN_174762 | -1.52 |
| NM_016018.4 | PHF20L1 | ILMN_164472 | -1.52 |
| NM_003715.2 | USO1 | ILMN_23419 | -1.52 |
| NM_001031684.1 | SFRS7 | ILMN_7620 | -1.52 |
| NM_030645.1 | SH3BP5L | ILMN_3697 | -1.53 |
| NM_152681.1 | FLJ38482 | ILMN_2215 | -1.53 |
| NM_012241.2 | SIRT5 | ILMN_18454 | -1.53 |
| NM_032323.1 | TMEM79 | ILMN_13555 | -1.53 |
| NM_052888.2 | LRRC37B | ILMN_3964 | -1.53 |
| NM_018145.1 | FAM82C | ILMN_9649 | -1.53 |
| NM_172002.3 | HSCB | ILMN_29188 | -1.53 |
| NM_002690.1 | POLB | ILMN_15404 | -1.53 |
| NM_000271.3 | NPC1 | ILMN_30618 | -1.54 |
| NM_012170.2 | FBXO22 | ILMN_5718 | -1.54 |
| NM_005484.2 | PARP2 | ILMN_7987 | -1.54 |
| NM_005707.1 | PDCD7 | ILMN_179659 | -1.55 |
| NM_152274.2 | FAM58A | ILMN_3352 | -1.55 |
| NM_001813.2 | CENPE | ILMN_7509 | -1.55 |
| NM_018286.2 | TMEM100 | ILMN_4881 | -1.55 |
| NM_002669.2 | PLRG1 | ILMN_22972 | -1.55 |
| NM_014751.2 | MTSS1 | ILMN_166688 | -1.56 |
| NM_153768.1 | CABYR | ILMN_9439 | -1.56 |
| NM_133646.2 | ZAK | ILMN_5666 | -1.56 |
| NM_031314.1 | HNRPC | ILMN_24356 | -1.56 |
| NM_139286.3 | CDC26 | ILMN_18022 | -1.56 |
| NM_017910.2 | FLJ20628 | ILMN_29305 | -1.57 |
| NM_018374.2 | TMEM106B | ILMN_14595 | -1.57 |
| NM_139279.3 | MCFD2 | ILMN_1677 | -1.57 |
| NM_207012.2 | AP3M1 | ILMN_4081 | -1.57 |
| NM_002396.3 | ME2 | ILMN_176679 | -1.57 |
| NM_032847.1 | C8orf76 | ILMN_8743 | -1.57 |
| NM_032936.2 | TMEM60 | ILMN_19683 | -1.57 |
| NM_016230.3 | CYB5R4 | ILMN_27429 | -1.58 |
| NM_000255.1 | MUT | ILMN_2157 | -1.58 |
| NM_007271.2 | STK38 | ILMN_8385 | -1.58 |
| NM_032376.2 | TMEM101 | ILMN_24128 | -1.58 |
| NM_170712.1 | RASSF1 | ILMN_11841 | -1.59 |
| NM_001044387.1 | ZNF557 | ILMN_180266 | -1.59 |
| NM_198567.2 | C5orf25 | ILMN_16903 | -1.59 |
| NM_020150.3 | SAR1A | ILMN_17495 | -1.59 |
| NM_014574.3 | STRN3 | ILMN_162469 | -1.59 |
| NM_022167.2 | XYLT2 | ILMN_26042 | -1.59 |
| NM_013263.2 | BRD7 | ILMN_14335 | -1.59 |
| NM_016023.2 | OTUD6B | ILMN_22397 | -1.6 |
| NM_017940.2 | NBPF1 | ILMN_163270 | -1.6 |
| NM_002451.3 | MTAP | ILMN_163674 | -1.6 |
| NM_001002002.1 | GMPR2 | ILMN_15872 | -1.6 |
| NM_014171.3 | CRIPT | ILMN_12903 | -1.6 |
| NM_024063.1 | SPATA5L1 | ILMN_4249 | -1.61 |
| NM_152705.1 | POLR1D | ILMN_28050 | -1.61 |
| NM_080730.2 | IFFO | ILMN_42149 | -1.61 |
| NM_001124.1 | ADM | ILMN_29514 | -1.61 |
| NM_016131.3 | RAB10 | ILMN_21971 | -1.61 |
| NM_018412.3 | ST7 | ILMN_16575 | -1.62 |
| XR_019071.1 | LOC642333 | ILMN_183964 | -1.62 |
| NM_004937.2 | CTNS | ILMN_162678 | -1.62 |
| NM_145052.1 | UPRT | ILMN_1103 | -1.62 |
| NM_001077498.1 | C17orf63 | ILMN_173887 | -1.62 |
| NM_173659.2 | RPUSD3 | ILMN_28804 | -1.62 |
| NM_025205.3 | MED28 | ILMN_14574 | -1.62 |
| NM_015969.2 | MRPS17 | ILMN_26133 | -1.62 |
| NR_002315.1 | LOC440926 | ILMN_19720 | -1.63 |
| NM_032448.1 | FAM120B | ILMN_10767 | -1.63 |
| NM_207304.1 | MBNL2 | ILMN_15554 | -1.64 |
| NM_198047.1 | HIBCH | ILMN_24888 | -1.64 |
| NM_020235.3 | BBX | ILMN_28437 | -1.64 |
| NM_004520.1 | KIF2A | ILMN_8115 | -1.64 |
| NM_001786.2 | CDC2 | ILMN_24793 | -1.65 |
| NM_001466.2 | FZD2 | ILMN_12499 | -1.65 |
| NM_024540.2 | MRPL24 | ILMN_29128 | -1.65 |
| NM_014044.4 | UNC50 | ILMN_19045 | -1.65 |
| NR_003105.1 | ZWILCH | ILMN_166966 | -1.65 |
| NM_013448.2 | BAZ1A | ILMN_164018 | -1.66 |
| NM_014946.3 | SPAST | ILMN_14993 | -1.66 |
| NM_001539.2 | DNAJA1 | ILMN_5819 | -1.66 |
| NM_004885.1 | NPFFR2 | ILMN_20676 | -1.67 |
| NM_001008783.1 | SLC35D3 | ILMN_16642 | -1.67 |
| NM_022037.1 | TIA1 | ILMN_30157 | -1.67 |
| NM_001918.2 | DBT | ILMN_169961 | -1.67 |
| NM_005333.2 | HCCS | ILMN_1794 | -1.68 |
| NM_015215.1 | CAMTA1 | ILMN_179380 | -1.68 |
| NM_033402.3 | LRRCC1 | ILMN_15234 | -1.68 |
| NM_013367.2 | ANAPC4 | ILMN_16297 | -1.68 |
| XM_001130192.1 | KIAA1160 | ILMN_162086 | -1.68 |
| NM_014755.1 | SERTAD2 | ILMN_4347 | -1.68 |
| NM_058216.1 | RAD51C | ILMN_2944 | -1.68 |
| NM_015423.2 | AASDHPPT | ILMN_25075 | -1.68 |
| NM_004048.2 | B2M | ILMN_19648 | -1.68 |
| NM_025058.3 | TRIM46 | ILMN_18492 | -1.69 |
| NM_021824.2 | NIF3L1 | ILMN_20423 | -1.69 |
| NM_017946.2 | FKBP14 | ILMN_18132 | -1.7 |
| NM_006441.1 | MTHFS | ILMN_1014 | -1.7 |
| NM_194298.1 | SLC16A9 | ILMN_19723 | -1.7 |
| NM_015542.2 | UPF2 | ILMN_21163 | -1.7 |
| NM_006282.2 | STK4 | ILMN_21491 | -1.7 |
| NM_005402.2 | RALA | ILMN_164730 | -1.7 |
| NM_030809.1 | FAM130A1 | ILMN_23790 | -1.71 |
| NM_023039.2 | ANKRA2 | ILMN_182144 | -1.71 |
| NM_033198.2 | PIGS | ILMN_7735 | -1.71 |
| NM_052849.2 | CCDC32 | ILMN_24282 | -1.71 |
| NM_006455.2 | SC65 | ILMN_21605 | -1.71 |
| NM_001439.2 | EXTL2 | ILMN_28046 | -1.71 |
| NM_001040181.1 | CLDND1 | ILMN_169193 | -1.71 |
| NM_025049.2 | PIF1 | ILMN_7325 | -1.72 |
| NM_001080546.1 | LOC219854 | ILMN_168339 | -1.72 |
| NM_007342.1 | NUPL2 | ILMN_2154 | -1.72 |
| NM_021800.2 | DNAJC12 | ILMN_177844 | -1.72 |
| NM_024051.2 | C7orf24 | ILMN_2391 | -1.73 |
| NM_001042631.1 | LOC644096 | ILMN_170495 | -1.73 |
| NM_001031677.2 | RAB24 | ILMN_25731 | -1.73 |
| NM_014322.2 | OPN3 | ILMN_166169 | -1.74 |
| NM_016248.2 | AKAP11 | ILMN_13368 | -1.74 |
| NM_005573.2 | LMNB1 | ILMN_4100 | -1.74 |
| NM_024516.2 | C16orf53 | ILMN_20272 | -1.74 |
| NM_001037494.1 | DYNLL1 | ILMN_14802 | -1.74 |
| NM_181705.1 | LOC90624 | ILMN_11045 | -1.75 |
| NM_014140.2 | SMARCAL1 | ILMN_19734 | -1.75 |
| NM_015984.2 | UCHL5 | ILMN_3370 | -1.75 |
| NM_016499.3 | MGC13379 | ILMN_180361 | -1.75 |
| NM_138797.1 | ANKRD54 | ILMN_21813 | -1.76 |
| NM_153812.1 | PHF13 | ILMN_27355 | -1.76 |
| NM_078629.1 | MSL3L1 | ILMN_29354 | -1.76 |
| NM_012129.2 | CLDN12 | ILMN_11012 | -1.76 |
| NM_014620.4 | HOXC4 | ILMN_16005 | -1.76 |
| NM_201262.1 | DNAJC12 | ILMN_18576 | -1.76 |
| NM_001866.2 | COX7B | ILMN_19298 | -1.76 |
| NM_013352.2 | DSE | ILMN_14589 | -1.77 |
| NM_001121.2 | ADD3 | ILMN_4026 | -1.77 |
| NM_017666.2 | ZNF280C | ILMN_3600 | -1.77 |
| NM_001004051.1 | GPRASP2 | ILMN_8440 | -1.77 |
| NM_015523.2 | REXO2 | ILMN_15016 | -1.77 |
| NM_013283.3 | MAT2B | ILMN_18923 | -1.77 |
| NM_020948.2 | MIER1 | ILMN_23077 | -1.78 |
| NM_004582.2 | RABGGTB | ILMN_25242 | -1.78 |
| NM_032172.1 | USP42 | ILMN_162869 | -1.78 |
| NM_145859.1 | PDCD10 | ILMN_26567 | -1.78 |
| NM_019852.3 | METTL3 | ILMN_13907 | -1.78 |
| NM_015344.1 | LEPROTL1 | ILMN_4515 | -1.78 |
| NM_177966.4 | PDE12 | ILMN_182612 | -1.79 |
| NM_001040057.1 | FAM133B | ILMN_181197 | -1.79 |
| NM_002717.2 | PPP2R2A | ILMN_24841 | -1.79 |
| NM_080821.2 | C20orf108 | ILMN_25852 | -1.79 |
| NM_003998.2 | NFKB1 | ILMN_161884 | -1.79 |
| NM_018256.2 | WDR12 | ILMN_14410 | -1.79 |
| NM_032346.1 | PDCD2L | ILMN_25365 | -1.8 |
| NM_153713.1 | LIX1L | ILMN_3572 | -1.8 |
| NM_005713.1 | COL4A3BP | ILMN_10635 | -1.8 |
| NM_012123.2 | MTO1 | ILMN_15725 | -1.81 |
| NM_199044.2 | NSUN4 | ILMN_23916 | -1.81 |
| NM_013229.2 | APAF1 | ILMN_29517 | -1.82 |
| NM_016001.2 | UTP18 | ILMN_17736 | -1.82 |
| NM_024331.3 | C20orf121 | ILMN_21132 | -1.82 |
| NM_001896.2 | CSNK2A2 | ILMN_16798 | -1.82 |
| NM_018121.2 | C10orf6 | ILMN_24540 | -1.83 |
| NM_133371.2 | MYOZ3 | ILMN_21305 | -1.83 |
| NM_001018109.1 | PIR | ILMN_13999 | -1.83 |
| NM_020401.2 | NUP107 | ILMN_24111 | -1.83 |
| NM_152316.1 | C11orf46 | ILMN_2540 | -1.83 |
| NM_018847.2 | KLHL9 | ILMN_20376 | -1.83 |
| NM_178314.2 | RILPL1 | ILMN_1609 | -1.84 |
| XM_938297.1 | LOC402644 | ILMN_30715 | -1.84 |
| NM_173475.1 | DCUN1D3 | ILMN_182838 | -1.85 |
| NM_001012413.1 | SGOL1 | ILMN_14464 | -1.85 |
| NM_025191.2 | EDEM3 | ILMN_15796 | -1.85 |
| NM_017850.1 | C1orf109 | ILMN_27592 | -1.85 |
| NM_020936.1 | DDX55 | ILMN_4596 | -1.85 |
| NM_001032290.1 | PSRC1 | ILMN_9176 | -1.85 |
| NM_002319.2 | LRCH4 | ILMN_139402 | -1.86 |
| NM_013388.4 | PREB | ILMN_6913 | -1.86 |
| NM_014033.3 | METTL7A | ILMN_40171 | -1.88 |
| NM_014290.1 | TDRD7 | ILMN_27692 | -1.88 |
| NM_031299.3 | CDCA3 | ILMN_18763 | -1.88 |
| NM_001033566.1 | RHOT1 | ILMN_6821 | -1.88 |
| NM_006407.3 | ARL6IP5 | ILMN_887 | -1.88 |
| NM_021800.2 | DNAJC12 | ILMN_15911 | -1.88 |
| NM_173079.1 | RUNDC1 | ILMN_12870 | -1.89 |
| NM_017612.2 | ZCCHC8 | ILMN_30318 | -1.89 |
| XM_941876.1 | BRI3BP | ILMN_139088 | -1.89 |
| NM_017953.2 | C1orf181 | ILMN_20839 | -1.89 |
| NM_005653.3 | TFCP2 | ILMN_22607 | -1.89 |
| NM_016133.2 | INSIG2 | ILMN_3152 | -1.89 |
| NM_005680.1 | TAF1B | ILMN_13234 | -1.89 |
| NM_145018.2 | C11orf82 | ILMN_11038 | -1.89 |
| NM_000945.3 | PPP3R1 | ILMN_26308 | -1.89 |
| NM_001009608.1 | C20orf94 | ILMN_24801 | -1.9 |
| NM_016010.1 | C8orf70 | ILMN_13979 | -1.9 |
| NM_145255.2 | MRPL10 | ILMN_19178 | -1.9 |
| NM_152515.2 | CKAP2L | ILMN_28483 | -1.9 |
| XM_377476.4 | MGC57346 | ILMN_165970 | -1.9 |
| NM_016076.3 | FAM152A | ILMN_176788 | -1.9 |
| NM_001024921.2 | RPL9 | ILMN_8640 | -1.9 |
| NM_022830.1 | TUT1 | ILMN_6523 | -1.91 |
| NM_003507.1 | FZD7 | ILMN_14429 | -1.91 |
| NM_018199.2 | EXDL2 | ILMN_4351 | -1.91 |
| NM_022473.1 | ZFP106 | ILMN_6305 | -1.91 |
| NM_018640.3 | LMO3 | ILMN_15180 | -1.91 |
| NM_018390.2 | PLCXD1 | ILMN_8273 | -1.92 |
| NM_001077268.1 | ZFYVE19 | ILMN_175347 | -1.92 |
| NM_006084.4 | IRF9 | ILMN_163101 | -1.92 |
| NM_004685.3 | MTMR6 | ILMN_29967 | -1.92 |
| NM_001048197.1 | SNHG3-RCC1 | ILMN_167397 | -1.92 |
| NM_001806.2 | CEBPG | ILMN_4860 | -1.92 |
| NM_003149.1 | STAC | ILMN_5832 | -1.93 |
| NM_003620.2 | PPM1D | ILMN_163927 | -1.93 |
| NM_020865.1 | DHX36 | ILMN_11905 | -1.93 |
| XM_001125680.1 | LOC730432 | ILMN_165880 | -1.94 |
| NM_033319.1 | CENPL | ILMN_21203 | -1.94 |
| XR_017252.1 | LOC284988 | ILMN_164134 | -1.94 |
| NM_031423.3 | NUF2 | ILMN_16808 | -1.95 |
| NM_018229.2 | C14orf108 | ILMN_180528 | -1.95 |
| NM_003314.1 | TTC1 | ILMN_11292 | -1.95 |
| NM_030808.3 | NDEL1 | ILMN_20362 | -1.95 |
| NM_018471.2 | ZC3H15 | ILMN_168262 | -1.95 |
| NM_016108.2 | AIG1 | ILMN_22004 | -1.96 |
| NM_015565.1 | ZNF294 | ILMN_5529 | -1.96 |
| NM_138794.2 | LYPLAL1 | ILMN_25005 | -1.96 |
| NM_001012968.2 | SPIN4 | ILMN_4105 | -1.96 |
| NM_001007793.1 | BUB3 | ILMN_5688 | -1.97 |
| NM_002013.2 | FKBP3 | ILMN_7680 | -1.97 |
| NM_001042426.1 | CENPA | ILMN_180589 | -1.97 |
| NM_153331.2 | KCTD6 | ILMN_15146 | -1.98 |
| NM_005966.3 | NAB1 | ILMN_18998 | -1.98 |
| NM_012238.3 | SIRT1 | ILMN_164649 | -1.98 |
| NM_001039141.1 | TRIOBP | ILMN_34620 | -1.98 |
| NM_003800.3 | RNGTT | ILMN_17056 | -1.98 |
| NM_052857.2 | CCDC16 | ILMN_169427 | -1.98 |
| NM_170784.1 | MKKS | ILMN_17701 | -1.98 |
| NM_015885.2 | PCF11 | ILMN_29970 | -1.99 |
| NM_021253.2 | TRIM39 | ILMN_165050 | -1.99 |
| NM_153261.4 | TMEM188 | ILMN_20778 | -1.99 |
| NM_001040285.1 | PAPD5 | ILMN_167231 | -1.99 |
| NM_001270.2 | CHD1 | ILMN_163604 | -1.99 |
| NM_003390.2 | WEE1 | ILMN_28737 | -2 |
| XM_931224.1 | LOC283683 | ILMN_45961 | -2 |
| NM_138390.2 | TMEM169 | ILMN_165130 | -2 |
| NM_006860.2 | RABL4 | ILMN_4559 | -2 |
| NM_021188.1 | ZNF410 | ILMN_11993 | -2 |
| NM_001099283.1 | ZNF239 | ILMN_306709 | -2.01 |
| NM_174921.1 | C4orf34 | ILMN_6140 | -2.01 |
| NM_001326.2 | CSTF3 | ILMN_27551 | -2.01 |
| NM_014885.3 | ANAPC10 | ILMN_2970 | -2.02 |
| NM_001042601.1 | TTC14 | ILMN_163780 | -2.02 |
| NM_002703.3 | PPAT | ILMN_6778 | -2.02 |
| NM_014802.1 | KIAA0528 | ILMN_18402 | -2.02 |
| NM_003824.2 | FADD | ILMN_11407 | -2.02 |
| NM_177968.2 | PPM1B | ILMN_29648 | -2.02 |
| NM_001827.1 | CKS2 | ILMN_14702 | -2.02 |
| NM_020748.1 | INTS2 | ILMN_1948 | -2.03 |
| NM_145274.2 | TMEM99 | ILMN_25105 | -2.03 |
| NM_016166.1 | PIAS1 | ILMN_16806 | -2.03 |
| NM_004897.2 | MINPP1 | ILMN_29353 | -2.03 |
| NM_001017369.1 | SC4MOL | ILMN_2901 | -2.03 |
| NM_016053.2 | CCDC53 | ILMN_25394 | -2.03 |
| NM_002643.3 | PIGF | ILMN_15261 | -2.03 |
| NM_014060.1 | MCTS1 | ILMN_13725 | -2.04 |
| NM_005642.2 | TAF7 | ILMN_19672 | -2.04 |
| NM_003744.5 | NUMB | ILMN_24350 | -2.04 |
| NM_145644.1 | MRPL35 | ILMN_20736 | -2.04 |
| NM_004865.2 | TBPL1 | ILMN_3787 | -2.04 |
| NM_001017928.2 | CCDC58 | ILMN_27140 | -2.05 |
| NM_006704.2 | SUGT1 | ILMN_26229 | -2.05 |
| NM_003359.2 | UGDH | ILMN_3906 | -2.05 |
| NM_006117.2 | PECI | ILMN_7427 | -2.05 |
| NM_152379.2 | C1orf131 | ILMN_9839 | -2.05 |
| NM_079837.2 | BANP | ILMN_8638 | -2.05 |
| NM_001097599.1 | TMEM22 | ILMN_306942 | -2.06 |
| NM_020153.2 | C11orf60 | ILMN_171038 | -2.06 |
| NM_007280.1 | OIP5 | ILMN_18200 | -2.06 |
| NM_001889.2 | CRYZ | ILMN_30248 | -2.06 |
| NR_002182.1 | NACAP1 | ILMN_14666 | -2.07 |
| NM_001099743.1 | GOLSYN | ILMN_308156 | -2.07 |
| NM_018004.1 | TMEM45A | ILMN_30168 | -2.07 |
| NM_018492.2 | PBK | ILMN_174875 | -2.07 |
| NM_006644.2 | HSPH1 | ILMN_1157 | -2.07 |
| NM_003729.2 | RTCD1 | ILMN_11697 | -2.08 |
| NM_004856.4 | KIF23 | ILMN_11802 | -2.08 |
| NM_012475.4 | USP21 | ILMN_18019 | -2.08 |
| NM_017785.3 | CCDC99 | ILMN_29954 | -2.08 |
| NM_002095.4 | GTF2E2 | ILMN_4316 | -2.08 |
| NM_030755.4 | TXNDC1 | ILMN_13849 | -2.09 |
| NM_012405.3 | ICMT | ILMN_31192 | -2.11 |
| NM_024632.4 | SAP30L | ILMN_18384 | -2.11 |
| NM_018204.2 | CKAP2 | ILMN_168115 | -2.11 |
| NM_017819.2 | RG9MTD1 | ILMN_26970 | -2.12 |
| NM_001002860.2 | BTBD7 | ILMN_178877 | -2.12 |
| NM_018244.3 | UQCC | ILMN_26543 | -2.12 |
| NM_005378.4 | MYCN | ILMN_178034 | -2.13 |
| NM_006145.1 | DNAJB1 | ILMN_19740 | -2.13 |
| NM_018120.3 | ARMC1 | ILMN_14242 | -2.13 |
| NM_004627.2 | WRB | ILMN_12263 | -2.13 |
| NM_006736.5 | DNAJB2 | ILMN_34421 | -2.13 |
| NM_019058.2 | DDIT4 | ILMN_13176 | -2.14 |
| NM_152408.1 | C5orf37 | ILMN_6243 | -2.15 |
| NM_001012756.1 | ZNF260 | ILMN_172733 | -2.15 |
| NM_014078.4 | MRPL13 | ILMN_17393 | -2.15 |
| NM_021970.2 | MAP2K1IP1 | ILMN_13073 | -2.15 |
| NM_032530.1 | ZNF594 | ILMN_309021 | -2.16 |
| NM_018473.2 | THEM2 | ILMN_27212 | -2.16 |
| NM_198436.1 | AURKA | ILMN_13382 | -2.16 |
| NM_138484.2 | SGOL1 | ILMN_14008 | -2.17 |
| NM_018062.2 | FANCL | ILMN_24728 | -2.17 |
| NM_001033925.1 | TIAL1 | ILMN_24357 | -2.17 |
| NM_001938.2 | DR1 | ILMN_182864 | -2.18 |
| NM_000456.2 | SUOX | ILMN_25551 | -2.18 |
| NM_031903.1 | MRPL32 | ILMN_25120 | -2.18 |
| NM_003403.3 | YY1 | ILMN_4019 | -2.18 |
| NM_016212.2 | TP53TG3 | ILMN_36480 | -2.19 |
| NM_006027.3 | EXO1 | ILMN_25997 | -2.19 |
| NM_006153.3 | NCK1 | ILMN_13975 | -2.19 |
| NM_018480.2 | TMEM126B | ILMN_18826 | -2.19 |
| NM_018443.2 | ZNF302 | ILMN_26438 | -2.19 |
| NM_024920.3 | DNAJB14 | ILMN_12080 | -2.2 |
| NM_002486.4 | NCBP1 | ILMN_23411 | -2.2 |
| NM_182972.2 | IRF2BP2 | ILMN_5645 | -2.2 |
| NM_001013690.1 | LOC401720 | ILMN_21595 | -2.21 |
| NM_021222.1 | PRUNE | ILMN_27601 | -2.21 |
| NM_198402.2 | PTPLB | ILMN_183743 | -2.21 |
| NM_024775.9 | GEMIN6 | ILMN_23187 | -2.21 |
| NM_030934.3 | C1orf25 | ILMN_163506 | -2.22 |
| NM_133458.2 | ZFP90 | ILMN_174886 | -2.22 |
| XR_018327.1 | LOC648343 | ILMN_163789 | -2.23 |
| NM_005398.4 | PPP1R3C | ILMN_4487 | -2.24 |
| NM_003729.1 | RTCD1 | ILMN_11697 | -2.24 |
| NM_001788.4 | SEPT7 | ILMN_25070 | -2.24 |
| NM_033426.2 | KIAA1737 | ILMN_24671 | -2.25 |
| NM_014779.2 | TSC22D2 | ILMN_5940 | -2.25 |
| NM_006777.3 | ZBTB33 | ILMN_12472 | -2.25 |
| NM_006101.1 | NDC80 | ILMN_14098 | -2.26 |
| NM_003584.1 | DUSP11 | ILMN_3801 | -2.26 |
| NM_004365.2 | CETN3 | ILMN_25663 | -2.26 |
| NM_017816.1 | LYAR | ILMN_23200 | -2.27 |
| NM_018846.2 | KLHL7 | ILMN_21425 | -2.27 |
| NM_016248.2 | AKAP11 | ILMN_13368 | -2.27 |
| NM_001002019.1 | PUS1 | ILMN_13055 | -2.28 |
| NM_024095.3 | ASB8 | ILMN_165486 | -2.28 |
| NM_001365.2 | DLG4 | ILMN_164548 | -2.29 |
| NM_020463.1 | SMEK2 | ILMN_21228 | -2.29 |
| NM_002692.2 | POLE2 | ILMN_19705 | -2.29 |
| NM_017816.1 | LYAR | ILMN_23200 | -2.29 |
| NM_001077394.1 | DPH5 | ILMN_175087 | -2.29 |
| NM_022079.2 | HERC4 | ILMN_8869 | -2.3 |
| NM_001007794.1 | CEPT1 | ILMN_15134 | -2.3 |
| NM_018064.2 | C6orf166 | ILMN_1311 | -2.3 |
| NM_002553.2 | ORC5L | ILMN_6212 | -2.3 |
| NM_014294.4 | TRAM1 | ILMN_3604 | -2.3 |
| NM_003318.3 | TTK | ILMN_24472 | -2.3 |
| NM_005830.2 | MRPS31 | ILMN_6293 | -2.31 |
| NM_183422.1 | TSC22D1 | ILMN_166165 | -2.32 |
| NM_019035.2 | PCDH18 | ILMN_179371 | -2.32 |
| NM_144594.1 | GTSF1 | ILMN_17221 | -2.33 |
| NM_005513.1 | GTF2E1 | ILMN_175401 | -2.34 |
| NM_001013406.1 | KRIT1 | ILMN_15411 | -2.34 |
| NM_005652.2 | TERF2 | ILMN_21134 | -2.34 |
| NM_018357.2 | LARP6 | ILMN_25584 | -2.34 |
| NM_024647.4 | NUP43 | ILMN_28463 | -2.34 |
| NM_080723.3 | NRSN1 | ILMN_178353 | -2.35 |
| NM_152524.3 | SGOL2 | ILMN_743 | -2.36 |
| NM_181702.1 | GEM | ILMN_16170 | -2.36 |
| NM_152755.1 | CNPY4 | ILMN_15383 | -2.36 |
| NM_012133.2 | COPG2 | ILMN_23766 | -2.37 |
| NM_018094.2 | GSPT2 | ILMN_20472 | -2.38 |
| NM_015994.2 | ATP6V1D | ILMN_26737 | -2.38 |
| NM_005836.2 | HRSP12 | ILMN_8062 | -2.39 |
| NM_001761.1 | CCNF | ILMN_27253 | -2.39 |
| NM_148178.1 | C9orf23 | ILMN_3926 | -2.4 |
| NM_025004.1 | CCDC15 | ILMN_4688 | -2.4 |
| NM_032320.5 | BTBD10 | ILMN_30066 | -2.4 |
| NM_006597.3 | HSPA8 | ILMN_181529 | -2.4 |
| NM_178439.3 | GMCL1 | ILMN_3285 | -2.42 |
| NM_080632.1 | UPF3B | ILMN_174905 | -2.43 |
| NM_001827.1 | CKS2 | ILMN_14702 | -2.43 |
| NM_138807.2 | C3orf31 | ILMN_9705 | -2.45 |
| NM_018186.2 | C1orf112 | ILMN_5134 | -2.46 |
| NM_152912.3 | MTIF3 | ILMN_16655 | -2.46 |
| NM_001007239.1 | KIAA0859 | ILMN_25045 | -2.46 |
| NM_001007157.1 | PHF14 | ILMN_2096 | -2.47 |
| NM_020799.2 | STAMBPL1 | ILMN_1387 | -2.48 |
| NR_001283.1 | TOP1P2 | ILMN_4755 | -2.48 |
| NM_133462.2 | TTC14 | ILMN_6310 | -2.48 |
| NM_198467.1 | RSBN1L | ILMN_10323 | -2.49 |
| NM_152415.1 | VPS37A | ILMN_12702 | -2.49 |
| NM_014117.2 | C16orf72 | ILMN_4283 | -2.5 |
| NM_014941.1 | MORC2 | ILMN_12502 | -2.5 |
| NM_170783.1 | ZNRD1 | ILMN_1419 | -2.5 |
| NM_014750.3 | DLG7 | ILMN_4880 | -2.5 |
| NM_025115.1 | C8orf41 | ILMN_19175 | -2.51 |
| NM_022494.1 | ZDHHC6 | ILMN_1193 | -2.51 |
| NM_033426.2 | KIAA1737 | ILMN_176915 | -2.52 |
| NM_031445.2 | AMMECR1L | ILMN_10683 | -2.52 |
| NM_181708.1 | BCDIN3D | ILMN_18065 | -2.53 |
| NM_024095.3 | ASB8 | ILMN_165486 | -2.54 |
| NM_003475.2 | RASSF7 | ILMN_12457 | -2.54 |
| NM_181837.1 | ORC3L | ILMN_3770 | -2.55 |
| NM_000819.3 | GART | ILMN_22974 | -2.55 |
| NM_018297.2 | NGLY1 | ILMN_15318 | -2.56 |
| NM_018410.3 | HJURP | ILMN_29337 | -2.56 |
| NM_025203.1 | C2orf44 | ILMN_12339 | -2.57 |
| NM_024297.2 | PHF23 | ILMN_20271 | -2.59 |
| NM_018364.3 | RSBN1 | ILMN_174594 | -2.59 |
| NM_004623.2 | TTC4 | ILMN_6668 | -2.59 |
| NM_145647.2 | WDR67 | ILMN_20846 | -2.6 |
| NM_015634.2 | KIAA1279 | ILMN_8497 | -2.6 |
| NM_018428.2 | UTP6 | ILMN_18247 | -2.6 |
| NM_005087.2 | FXR1 | ILMN_18674 | -2.6 |
| NM_006190.3 | ORC2L | ILMN_182860 | -2.61 |
| NM_001077395.1 | DPH5 | ILMN_181061 | -2.61 |
| NM_016042.2 | EXOSC3 | ILMN_174330 | -2.61 |
| NM_006416.3 | SLC35A1 | ILMN_23284 | -2.62 |
| NM_024945.2 | RMI1 | ILMN_11713 | -2.63 |
| NM_020345.3 | NKIRAS1 | ILMN_28229 | -2.63 |
| NM_153331.2 | KCTD6 | ILMN_15146 | -2.63 |
| NM_018131.3 | CEP55 | ILMN_6470 | -2.63 |
| NM_019005.3 | FLJ20323 | ILMN_12567 | -2.63 |
| NM_015608.2 | C10orf137 | ILMN_22392 | -2.64 |
| NM_000645.2 | AGL | ILMN_1173 | -2.64 |
| NM_153201.1 | HSPA8 | ILMN_14829 | -2.64 |
| NM_173647.2 | RNF149 | ILMN_10320 | -2.65 |
| NM_020234.4 | DTWD1 | ILMN_3248 | -2.65 |
| NM_198391.1 | FLRT3 | ILMN_23273 | -2.65 |
| NM_014177.1 | C18orf55 | ILMN_9697 | -2.66 |
| NM_012210.3 | TRIM32 | ILMN_14426 | -2.68 |
| NM_030805.2 | LMAN2L | ILMN_1985 | -2.68 |
| NM_022173.1 | TIA1 | ILMN_29910 | -2.68 |
| NM_199229.1 | RPE | ILMN_9823 | -2.68 |
| NR_001449.1 | TRK1 | ILMN_6493 | -2.69 |
| NM_022451.9 | NOC3L | ILMN_11360 | -2.69 |
| NM_014184.2 | CNIH4 | ILMN_9903 | -2.69 |
| NM_170783.1 | ZNRD1 | ILMN_1419 | -2.7 |
| NM_001033925.1 | TIAL1 | ILMN_24357 | -2.7 |
| NM_024945.1 | RMI1 | ILMN_11713 | -2.7 |
| NM_015475.3 | FAM98A | ILMN_16819 | -2.71 |
| NM_007342.1 | NUPL2 | ILMN_2154 | -2.71 |
| XM_930579.2 | LOC653820 | ILMN_40990 | -2.73 |
| NM_032358.2 | CCDC77 | ILMN_23011 | -2.74 |
| NM_182919.1 | TICAM1 | ILMN_11434 | -2.75 |
| NM_001006622.1 | WDR33 | ILMN_6581 | -2.76 |
| NM_152360.2 | ZNF573 | ILMN_23003 | -2.76 |
| NM_153018.2 | ZFP3 | ILMN_42182 | -2.77 |
| NM_014305.2 | TGDS | ILMN_30985 | -2.77 |
| NM_024071.2 | ZFYVE21 | ILMN_1317 | -2.78 |
| NM_012460.2 | TIMM9 | ILMN_9968 | -2.8 |
| NM_016551.1 | TM7SF3 | ILMN_7797 | -2.81 |
| NM_181702.1 | GEM | ILMN_16170 | -2.81 |
| NM_013316.2 | CNOT4 | ILMN_22777 | -2.81 |
| NM_017915.2 | C12orf48 | ILMN_42497 | -2.83 |
| XM_938089.2 | LOC643007 | ILMN_31054 | -2.83 |
| NM_022893.2 | BCL11A | ILMN_17359 | -2.84 |
| NM_000628.3 | IL10RB | ILMN_26097 | -2.84 |
| NM_005783.3 | TXNDC9 | ILMN_24064 | -2.84 |
| NM_198401.2 | ANKRD46 | ILMN_9031 | -2.87 |
| NM_014039.2 | C11orf54 | ILMN_4783 | -2.89 |
| NM_001014286.2 | FAM48A | ILMN_1616 | -2.89 |
| NM_032138.3 | KBTBD7 | ILMN_181309 | -2.92 |
| NM_015462.3 | NOL11 | ILMN_5347 | -2.92 |
| NM_005926.2 | MFAP1 | ILMN_20656 | -2.93 |
| NM_001012643.2 | LOC339344 | ILMN_6535 | -2.94 |
| NM_207035.1 | C1orf63 | ILMN_22593 | -2.95 |
| NM_015314.2 | KIAA0895 | ILMN_28455 | -2.95 |
| NM_014596.4 | ZNRD1 | ILMN_20009 | -2.96 |
| NM_014487.3 | ZNF330 | ILMN_6878 | -2.99 |
| NM_138798.1 | MITD1 | ILMN_27516 | -3 |
| NM_020147.2 | THAP10 | ILMN_182683 | -3.02 |
| NM_000856.3 | GUCY1A3 | ILMN_11680 | -3.03 |
| NM_015948.2 | SLC35B3 | ILMN_20545 | -3.03 |
| NM_001040708.1 | HEY1 | ILMN_164416 | -3.05 |
| NM_016374.5 | ARID4B | ILMN_162934 | -3.07 |
| NM_173510.1 | CCDC117 | ILMN_21814 | -3.08 |
| NM_024057.2 | NUP37 | ILMN_4147 | -3.08 |
| NM_001009894.2 | C12orf29 | ILMN_26574 | -3.09 |
| NM_002158.3 | FOXN2 | ILMN_167513 | -3.1 |
| NM_001037163.1 | MGC12966 | ILMN_182436 | -3.12 |
| NM_020892.1 | DTX2 | ILMN_21612 | -3.13 |
| NM_024090.1 | ELOVL6 | ILMN_11340 | -3.13 |
| NM_052879.3 | LARP4 | ILMN_2132 | -3.14 |
| NM_006630.1 | ZNF234 | ILMN_29233 | -3.15 |
| NM_003002.1 | SDHD | ILMN_6353 | -3.15 |
| NM_024546.3 | RNF219 | ILMN_38012 | -3.18 |
| NM_001007278.1 | TRIM13 | ILMN_14225 | -3.21 |
| NM_198434.1 | AURKA | ILMN_12352 | -3.21 |
| NM_001007278.1 | TRIM13 | ILMN_14225 | -3.23 |
| NM_004503.3 | HOXC6 | ILMN_15669 | -3.24 |
| NM_022346.3 | NCAPG | ILMN_23620 | -3.24 |
| NM_001033503.1 | SAR1B | ILMN_16595 | -3.25 |
| NM_001007157.1 | PHF14 | ILMN_2096 | -3.26 |
| NM_007198.2 | PROSC | ILMN_23472 | -3.28 |
| NM_006299.3 | ZNF193 | ILMN_10151 | -3.29 |
| NM_016561.1 | BFAR | ILMN_23440 | -3.31 |
| NM_004428.2 | EFNA1 | ILMN_14320 | -3.33 |
| NM_018343.1 | RIOK2 | ILMN_16482 | -3.34 |
| NM_015942.3 | MTERFD1 | ILMN_24756 | -3.34 |
| NM_001007230.1 | SPOP | ILMN_12838 | -3.35 |
| NM_201280.1 | MUTED | ILMN_21576 | -3.36 |
| NM_003452.2 | ZNF189 | ILMN_4798 | -3.4 |
| NM_183399.1 | RNF14 | ILMN_7292 | -3.45 |
| NM_024585.2 | ARMC7 | ILMN_163623 | -3.51 |
| NM_005681.2 | TAF1A | ILMN_8114 | -3.53 |
| NM_144726.1 | RNF145 | ILMN_27136 | -3.56 |
| NM_032280.1 | ZCCHC9 | ILMN_25119 | -3.58 |
| NM_006004.1 | UQCRH | ILMN_138507 | -3.77 |
| NM_016618.1 | KRCC1 | ILMN_25337 | -3.79 |
| NM_006630.1 | ZNF234 | ILMN_29233 | -3.81 |
| NM_007167.2 | ZMYM6 | ILMN_1275 | -3.84 |
| NM_015942.3 | MTERFD1 | ILMN_174209 | -3.93 |
| NM_033091.1 | TRIM4 | ILMN_8530 | -4.1 |
| NM_016277.3 | RAB23 | ILMN_177407 | -4.11 |
| NM_004316.2 | ASCL1 | ILMN_23892 | -4.11 |
| NM_016042.2 | EXOSC3 | ILMN_174330 | -4.34 |
| NM_138316.2 | PANK1 | ILMN_406 | -4.39 |
| NM_004982.2 | KCNJ8 | ILMN_29993 | -4.66 |
| NM_001039937.1 | INTS6 | ILMN_38649 | -5.01 |
| NM_005345.4 | HSPA1A | ILMN_6623 | -5.98 |
| NM_005346.3 | HSPA1B | ILMN_25549 | -7.33 |
