# Supplementary material for: Minimal Peroxide Exposure of Neuronal Cells Induces Multifaceted Adaptive Responses
Source: PLoS One. 2010 Dec 17;5(12):e14352. doi: 10.1371/journal.pone.0014352 (PMC3003681; doi:10.1371/journal.pone.0014352)
Supplement: Table S4 — MeCh-significantly regulated genes after 4 hours of stimulation in the control state SH-SY5Y cells. Each significantly regulated gene is described via its accession number (ACCESSION), Gene Symbol (SYMBOL), Illumina array transcript designation (TRANSCRIPT). For each gene the z-ratio of expression compared to un-treated cells after 4 hours of ligand stimulation is displayed (CTL MeCh 4). (1.26 MB DOC) [file pone.0014352.s011.doc]

**Table S4. MeCh-significantly regulated genes after 4 hours of stimulation in the control state SH-SY5Y cells**. Each significantly regulated gene is described via its accession number (ACCESSION), Gene Symbol (SYMBOL), Illumina array transcript designation (TRANSCRIPT). For each gene the z-ratio of expression compared to un-treated cells after 4 hours of ligand stimulation is displayed (CTL MeCh 4).

| **ACCESSION** | **SYMBOL** | **TRANSCRIPT** | **CTL MeCh 4** |
| --- | --- | --- | --- |
| NM_000584.2 | IL8 | ILMN_179575 | 8.12 |
| NM_005324.3 | H3F3B | ILMN_26885 | 5.02 |
| NM_148957.2 | TNFRSF19 | ILMN_28684 | 4.63 |
| XM_944439.2 | LOC653994 | ILMN_38572 | 4.42 |
| NM_002673.3 | PLXNB1 | ILMN_22628 | 4.33 |
| NM_014817.3 | KIAA0644 | ILMN_164846 | 4.23 |
| NM_016028.4 | SUV420H1 | ILMN_29861 | 4.08 |
| NM_001033506.1 | CSTF3 | ILMN_27049 | 4.05 |
| NM_080491.1 | GAB2 | ILMN_3317 | 3.85 |
| NM_033138.2 | CALD1 | ILMN_29896 | 3.84 |
| NM_015995.2 | KLF13 | ILMN_16226 | 3.81 |
| NM_018697.3 | LANCL2 | ILMN_920 | 3.71 |
| NM_003086.2 | SNAPC4 | ILMN_180505 | 3.68 |
| NM_001040456.1 | RHBDD2 | ILMN_168345 | 3.68 |
| NM_003461.4 | ZYX | ILMN_2137 | 3.67 |
| NM_002160.2 | TNC | ILMN_14948 | 3.58 |
| NM_001040456.1 | RHBDD2 | ILMN_168345 | 3.56 |
| NM_012401.2 | PLXNB2 | ILMN_308861 | 3.53 |
| NM_002473.3 | MYH9 | ILMN_183555 | 3.44 |
| NM_024663.3 | NPEPL1 | ILMN_175218 | 3.42 |
| NM_182492.1 | LRP5L | ILMN_650 | 3.41 |
| NM_006997.2 | TACC2 | ILMN_16130 | 3.36 |
| NM_003461.4 | ZYX | ILMN_2137 | 3.33 |
| NM_000479.2 | AMH | ILMN_171371 | 3.32 |
| NM_003110.4 | SP2 | ILMN_7882 | 3.31 |
| NM_020310.2 | MNT | ILMN_21283 | 3.25 |
| NM_001080453.1 | INTS1 | ILMN_173681 | 3.21 |
| NM_020724.1 | RNF150 | ILMN_26801 | 3.17 |
| NM_000617.1 | SLC11A2 | ILMN_10129 | 3.11 |
| NM_002213.3 | ITGB5 | ILMN_24189 | 3.1 |
| XM_001126418.1 | LOC727935 | ILMN_181411 | 3.08 |
| NM_000199.2 | SGSH | ILMN_7542 | 3.07 |
| NM_005245.3 | FAT | ILMN_24617 | 3.06 |
| NM_017635.3 | SUV420H1 | ILMN_174505 | 3.04 |
| NM_005189.1 | CBX2 | ILMN_28525 | 3.03 |
| NM_170695.2 | TGIF1 | ILMN_162784 | 3.01 |
| NM_006739.3 | MCM5 | ILMN_20107 | 2.98 |
| NM_001300.4 | KLF6 | ILMN_17961 | 2.98 |
| NM_001008408.3 | RBM33 | ILMN_165407 | 2.97 |
| NM_001008237.1 | TTC32 | ILMN_4829 | 2.94 |
| XM_001134411.1 | LOC441150 | ILMN_176720 | 2.93 |
| NM_012215.2 | MGEA5 | ILMN_11399 | 2.88 |
| NM_002975.2 | CLEC11A | ILMN_29894 | 2.87 |
| NM_201557.2 | FHL2 | ILMN_42988 | 2.86 |
| NM_014000.2 | VCL | ILMN_27566 | 2.86 |
| NM_017757.2 | ZNF407 | ILMN_12747 | 2.83 |
| NM_201555.1 | FHL2 | ILMN_21541 | 2.83 |
| XM_001134215.1 | PDPR | ILMN_162295 | 2.82 |
| NM_001008490.1 | KLF6 | ILMN_12381 | 2.79 |
| NM_002874.3 | RAD23B | ILMN_19346 | 2.78 |
| NM_005194.2 | CEBPB | ILMN_4674 | 2.76 |
| NM_001012626.1 | LOC285074 | ILMN_21153 | 2.75 |
| NM_014717.1 | ZNF536 | ILMN_179125 | 2.73 |
| NM_152493.2 | ZNF362 | ILMN_7745 | 2.73 |
| NM_002566.4 | P2RY11 | ILMN_12237 | 2.72 |
| NM_018948.2 | ERRFI1 | ILMN_4328 | 2.72 |
| NM_001080485.1 | ZNF275 | ILMN_180340 | 2.71 |
| NM_016481.3 | C9orf156 | ILMN_12842 | 2.7 |
| XM_495939.3 | KIAA1545 | ILMN_40920 | 2.69 |
| XM_931359.2 | LOC338758 | ILMN_37634 | 2.69 |
| NM_001001391.1 | CD44 | ILMN_10947 | 2.69 |
| XM_942544.2 | INTS1 | ILMN_38896 | 2.69 |
| NM_005334.2 | HCFC1 | ILMN_24237 | 2.69 |
| NM_020796.3 | SEMA6A | ILMN_11282 | 2.68 |
| NM_152470.2 | RNF165 | ILMN_14516 | 2.67 |
| NM_003565.1 | ULK1 | ILMN_2158 | 2.67 |
| NM_033419.3 | PERLD1 | ILMN_12215 | 2.65 |
| NM_001006115.2 | IHPK1 | ILMN_8379 | 2.65 |
| NM_004075.2 | CRY1 | ILMN_6263 | 2.65 |
| XM_926036.1 | LOC653103 | ILMN_32029 | 2.64 |
| NM_001280.1 | CIRBP | ILMN_24327 | 2.63 |
| NM_024735.2 | FBXO31 | ILMN_17806 | 2.61 |
| NM_022748.10 | TNS3 | ILMN_17676 | 2.59 |
| NM_001013258.1 | ZNF789 | ILMN_11535 | 2.56 |
| NM_000787.3 | DBH | ILMN_25962 | 2.56 |
| NM_006494.1 | ERF | ILMN_14193 | 2.54 |
| NM_004566.2 | PFKFB3 | ILMN_163833 | 2.54 |
| NM_206852.1 | RTN1 | ILMN_3435 | 2.54 |
| NM_004145.2 | MYO9B | ILMN_25414 | 2.52 |
| NM_005157.3 | ABL1 | ILMN_4033 | 2.52 |
| NM_006372.3 | SYNCRIP | ILMN_28470 | 2.51 |
| NM_022450.2 | RHBDF1 | ILMN_20892 | 2.5 |
| NM_015447.1 | CAMSAP1 | ILMN_815 | 2.49 |
| NM_004090.2 | DUSP3 | ILMN_180655 | 2.49 |
| NM_177401.4 | MIDN | ILMN_6472 | 2.49 |
| NM_020920.2 | CHD8 | ILMN_3472 | 2.48 |
| NM_018202.3 | TMEM57 | ILMN_30191 | 2.47 |
| NM_033446.1 | FAM125B | ILMN_20760 | 2.47 |
| NM_173042.2 | IL18BP | ILMN_30884 | 2.46 |
| NM_001008237.1 | TTC32 | ILMN_4829 | 2.46 |
| NM_014380.1 | NGFRAP1 | ILMN_7162 | 2.45 |
| NR_003659.1 | FAM39DP | ILMN_307683 | 2.45 |
| NM_024909.1 | C6orf134 | ILMN_21139 | 2.44 |
| NM_022720.5 | DGCR8 | ILMN_1552 | 2.44 |
| NM_014747.2 | RIMS3 | ILMN_21581 | 2.44 |
| NM_001013685.1 | LOC401357 | ILMN_29013 | 2.43 |
| NM_017741.3 | C4orf30 | ILMN_172318 | 2.43 |
| NM_032199.1 | ARID5B | ILMN_165822 | 2.43 |
| NM_001009937.1 | SLC25A26 | ILMN_15004 | 2.41 |
| NM_170721.1 | MSI2 | ILMN_25750 | 2.4 |
| XM_945571.1 | ANKRD13D | ILMN_138370 | 2.4 |
| NM_006185.2 | NUMA1 | ILMN_25058 | 2.4 |
| NM_001017421.1 | FKSG30 | ILMN_2393 | 2.4 |
| NM_020808.3 | SIPA1L2 | ILMN_167573 | 2.39 |
| NM_015684.2 | ATP5S | ILMN_26667 | 2.38 |
| NM_002938.2 | RNF4 | ILMN_176496 | 2.38 |
| XM_379215.2 | LOC132241 | ILMN_37830 | 2.38 |
| NM_004126.3 | GNG11 | ILMN_8981 | 2.38 |
| NM_015338.4 | ASXL1 | ILMN_183479 | 2.35 |
| NM_001095.2 | ACCN2 | ILMN_27416 | 2.33 |
| NR_000011.1 | SNORA70 | ILMN_7210 | 2.33 |
| NM_003972.2 | BTAF1 | ILMN_8616 | 2.33 |
| NM_001496.3 | GFRA3 | ILMN_8392 | 2.32 |
| NM_001614.2 | ACTG1 | ILMN_24353 | 2.32 |
| NM_014901.4 | RNF44 | ILMN_20290 | 2.31 |
| NM_003622.2 | PPFIBP1 | ILMN_172147 | 2.3 |
| NM_012256.2 | ZNF212 | ILMN_14026 | 2.3 |
| NM_005688.2 | ABCC5 | ILMN_25223 | 2.28 |
| NM_001024070.1 | GCH1 | ILMN_23648 | 2.27 |
| NM_153188.2 | TNPO1 | ILMN_29083 | 2.27 |
| NM_003076.3 | SMARCD1 | ILMN_16093 | 2.27 |
| NM_002473.3 | MYH9 | ILMN_183555 | 2.26 |
| NM_014694.2 | ADAMTSL2 | ILMN_697 | 2.25 |
| NM_014330.2 | PPP1R15A | ILMN_1024 | 2.25 |
| NM_004634.2 | BRPF1 | ILMN_17537 | 2.25 |
| NM_014667.1 | VGLL4 | ILMN_29344 | 2.25 |
| NM_020248.2 | CTNNBIP1 | ILMN_23888 | 2.23 |
| NM_015085.3 | GARNL4 | ILMN_163593 | 2.22 |
| NM_022910.1 | NDRG4 | ILMN_8824 | 2.22 |
| NM_003565.1 | ULK1 | ILMN_2158 | 2.22 |
| NM_002936.3 | RNASEH1 | ILMN_17680 | 2.22 |
| NM_024077.3 | SECISBP2 | ILMN_19156 | 2.21 |
| NM_001003795.2 | GTF2IRD2B | ILMN_12811 | 2.21 |
| NM_013313.3 | YPEL1 | ILMN_26647 | 2.2 |
| NM_024048.2 | MGC3020 | ILMN_29369 | 2.2 |
| NM_002972.1 | SBF1 | ILMN_22729 | 2.2 |
| NM_006123.2 | IDS | ILMN_17605 | 2.2 |
| NM_001003805.1 | ATP5S | ILMN_1363 | 2.2 |
| NM_021090.3 | MTMR3 | ILMN_27578 | 2.2 |
| NM_152398.2 | OCIAD2 | ILMN_18246 | 2.2 |
| NM_001287.3 | CLCN7 | ILMN_8600 | 2.2 |
| NM_002938.2 | RNF4 | ILMN_26467 | 2.19 |
| NM_020822.1 | KCNT1 | ILMN_21599 | 2.19 |
| NM_001003803.1 | ATP5S | ILMN_27869 | 2.18 |
| NM_152679.2 | SLC10A4 | ILMN_1323 | 2.18 |
| NM_001020820.1 | MYADM | ILMN_8340 | 2.18 |
| NM_023072.1 | ZSWIM4 | ILMN_167043 | 2.18 |
| NM_024301.3 | FKRP | ILMN_173675 | 2.17 |
| NM_012068.3 | ATF5 | ILMN_6490 | 2.17 |
| NM_016644.1 | PRR16 | ILMN_4368 | 2.16 |
| NM_006045.1 | ATP9A | ILMN_176431 | 2.15 |
| NM_006465.2 | ARID3B | ILMN_4032 | 2.12 |
| NM_004424.3 | E4F1 | ILMN_23848 | 2.12 |
| NM_013336.3 | SEC61A1 | ILMN_9397 | 2.12 |
| XM_928464.1 | LOC146517 | ILMN_32888 | 2.11 |
| NM_001677.3 | ATP1B1 | ILMN_25542 | 2.11 |
| NM_021737.1 | CLCN6 | ILMN_6195 | 2.1 |
| NM_024109.2 | C16orf68 | ILMN_12265 | 2.1 |
| NM_013276.2 | SHPK | ILMN_22706 | 2.1 |
| NM_001144.4 | AMFR | ILMN_22219 | 2.1 |
| NM_004078.1 | CSRP1 | ILMN_25451 | 2.1 |
| NM_006009.2 | TUBA1A | ILMN_1089 | 2.1 |
| NM_020859.1 | SHRM | ILMN_16821 | 2.09 |
| NM_001567.2 | INPPL1 | ILMN_20903 | 2.09 |
| NM_144781.1 | PDCD2 | ILMN_16269 | 2.09 |
| NM_012463.2 | ATP6V0A2 | ILMN_23163 | 2.08 |
| NM_014014.2 | ASCC3L1 | ILMN_18834 | 2.08 |
| NM_001081559.1 | CPSF4 | ILMN_178236 | 2.08 |
| NM_133471.1 | KIAA1949 | ILMN_308966 | 2.07 |
| NM_004393.2 | DAG1 | ILMN_16432 | 2.06 |
| NM_001080477.1 | ODZ3 | ILMN_179907 | 2.06 |
| NM_020695.3 | REXO1 | ILMN_20923 | 2.05 |
| NM_199169.1 | PMEPA1 | ILMN_13834 | 2.05 |
| NM_153451.2 | ORAOV1 | ILMN_5733 | 2.05 |
| NM_003131.2 | SRF | ILMN_22299 | 2.05 |
| NM_001024071.1 | GCH1 | ILMN_14690 | 2.04 |
| NM_014281.3 | PUF60 | ILMN_14897 | 2.04 |
| NM_175847.1 | PTBP1 | ILMN_20407 | 2.04 |
| NM_199043.1 | C14orf102 | ILMN_22442 | 2.03 |
| NM_003290.1 | TPM4 | ILMN_9334 | 2.03 |
| NM_017514.2 | PLXNA3 | ILMN_162939 | 2.03 |
| NM_177952.1 | PPM1A | ILMN_10552 | 2.02 |
| NM_015330.1 | SPECC1L | ILMN_168707 | 2.02 |
| NM_152322.2 | BTBD11 | ILMN_506 | 2.01 |
| NM_080677.1 | DYNLL2 | ILMN_28971 | 2.01 |
| NM_001101.2 | ACTB | ILMN_2565 | 2.01 |
| NM_015124.2 | GRAMD4 | ILMN_12136 | 2 |
| XM_937850.1 | LOC285176 | ILMN_43277 | 2 |
| XM_938988.1 | LOC402221 | ILMN_35678 | 2 |
| NM_015144.2 | ZCCHC14 | ILMN_32176 | 2 |
| NM_022766.4 | CERK | ILMN_24122 | 2 |
| NM_002374.3 | MAP2 | ILMN_38764 | 1.99 |
| NM_005920.2 | MEF2D | ILMN_3465 | 1.99 |
| NM_152557.3 | ZNF746 | ILMN_25894 | 1.99 |
| NM_181784.1 | SPRED2 | ILMN_12131 | 1.98 |
| NM_172251.1 | MRPL54 | ILMN_1467 | 1.98 |
| NM_002862.3 | PYGB | ILMN_21544 | 1.97 |
| NM_014838.2 | ZBED4 | ILMN_8641 | 1.97 |
| XM_001127981.1 | LOC728014 | ILMN_169164 | 1.97 |
| XM_290799.7 | ARHGAP23 | ILMN_162296 | 1.96 |
| NM_182705.2 | FAM101B | ILMN_1388 | 1.96 |
| NM_005081.2 | ZNF142 | ILMN_11618 | 1.96 |
| NM_024900.3 | PHF17 | ILMN_1535 | 1.96 |
| NM_006047.4 | RBM12 | ILMN_183773 | 1.96 |
| NM_001013699.1 | LOC440093 | ILMN_19743 | 1.96 |
| NM_015077.2 | SARM1 | ILMN_23861 | 1.95 |
| NM_178831.4 | GATS | ILMN_18755 | 1.95 |
| NM_030665.3 | RAI1 | ILMN_176671 | 1.94 |
| NM_018622.5 | PARL | ILMN_163763 | 1.93 |
| NM_017566.2 | KLHDC4 | ILMN_8527 | 1.92 |
| NM_198679.1 | RAPGEF1 | ILMN_177243 | 1.91 |
| NM_003045.3 | SLC7A1 | ILMN_162673 | 1.91 |
| NM_002333.1 | LRP3 | ILMN_12327 | 1.91 |
| NM_005561.2 | LAMP1 | ILMN_27826 | 1.91 |
| NM_006715.2 | MAN2C1 | ILMN_685 | 1.9 |
| NM_138440.2 | VASN | ILMN_31069 | 1.9 |
| NM_001012614.1 | CTBP1 | ILMN_21952 | 1.9 |
| NM_012154.2 | EIF2C2 | ILMN_25413 | 1.9 |
| NM_004321.4 | KIF1A | ILMN_22617 | 1.9 |
| NM_152280.2 | SYT11 | ILMN_23967 | 1.89 |
| NM_001101.2 | ACTB | ILMN_2565 | 1.89 |
| NM_006885.3 | ZFHX3 | ILMN_174159 | 1.88 |
| NM_001003725.1 | WDR68 | ILMN_19537 | 1.87 |
| NM_001259.5 | CDK6 | ILMN_178275 | 1.87 |
| NM_014947.3 | FOXJ3 | ILMN_26064 | 1.86 |
| NM_006773.3 | DDX18 | ILMN_22238 | 1.86 |
| NM_001924.2 | GADD45A | ILMN_17355 | 1.85 |
| NM_017821.3 | RHBDL2 | ILMN_20003 | 1.85 |
| NM_006731.2 | FKTN | ILMN_6512 | 1.85 |
| NM_203499.1 | DDX42 | ILMN_1880 | 1.84 |
| NM_014869.3 | IQSEC1 | ILMN_21247 | 1.83 |
| NM_006029.4 | PNMA1 | ILMN_18607 | 1.83 |
| NM_031263.1 | HNRPK | ILMN_16515 | 1.83 |
| NM_024319.2 | C1orf35 | ILMN_28904 | 1.82 |
| NM_138774.2 | C19orf22 | ILMN_15785 | 1.82 |
| NM_001003795.2 | GTF2IRD2B | ILMN_12811 | 1.82 |
| NM_139235.3 | NOL6 | ILMN_7349 | 1.82 |
| NM_001025091.1 | ABCF1 | ILMN_179040 | 1.81 |
| NM_018708.2 | FEM1A | ILMN_2838 | 1.81 |
| NM_006110.1 | CD2BP2 | ILMN_9406 | 1.81 |
| NM_001031617.2 | COX19 | ILMN_15655 | 1.8 |
| NM_002627.3 | PFKP | ILMN_16104 | 1.8 |
| NM_017999.4 | RNF31 | ILMN_170206 | 1.79 |
| NM_014717.1 | ZNF536 | ILMN_179125 | 1.79 |
| NM_015949.2 | C7orf20 | ILMN_23467 | 1.79 |
| XM_930411.1 | LOC645099 | ILMN_37678 | 1.79 |
| NM_016143.3 | NSFL1C | ILMN_20493 | 1.79 |
| NM_012384.2 | GMEB2 | ILMN_7174 | 1.79 |
| NM_014268.1 | MAPRE2 | ILMN_8637 | 1.79 |
| NM_002228.3 | JUN | ILMN_7746 | 1.78 |
| NM_173073.2 | SLC35C2 | ILMN_14167 | 1.78 |
| NM_005342.2 | HMGB3 | ILMN_8326 | 1.78 |
| NM_004454.1 | ETV5 | ILMN_12676 | 1.78 |
| NM_003119.2 | SPG7 | ILMN_26332 | 1.78 |
| NM_017456.1 | PSCD1 | ILMN_16992 | 1.78 |
| NM_203288.1 | RP9 | ILMN_23874 | 1.78 |
| NM_138930.2 | DIABLO | ILMN_19433 | 1.77 |
| NM_015444.2 | TMEM158 | ILMN_13668 | 1.77 |
| NM_001111.3 | ADAR | ILMN_20593 | 1.77 |
| XM_940903.2 | ZC3H5 | ILMN_40646 | 1.76 |
| NM_003610.3 | RAE1 | ILMN_24358 | 1.76 |
| NM_002650.1 | PIK4CA | ILMN_20581 | 1.76 |
| NM_003899.2 | ARHGEF7 | ILMN_29199 | 1.76 |
| NM_032621.2 | BEX2 | ILMN_24134 | 1.76 |
| NM_032421.2 | CLIP2 | ILMN_14847 | 1.75 |
| XM_931434.2 | LOC400027 | ILMN_35789 | 1.75 |
| NM_001481.1 | GAS8 | ILMN_26809 | 1.74 |
| NM_003458.3 | BSN | ILMN_22754 | 1.74 |
| NM_002819.3 | PTBP1 | ILMN_20993 | 1.74 |
| NM_014338.3 | PISD | ILMN_28266 | 1.73 |
| NM_032520.3 | GNPTG | ILMN_28173 | 1.73 |
| NM_018622.5 | PARL | ILMN_163763 | 1.73 |
| NM_001065.2 | TNFRSF1A | ILMN_173343 | 1.72 |
| XM_001127981.1 | LOC728014 | ILMN_169164 | 1.72 |
| NM_001025243.1 | IRAK1 | ILMN_23652 | 1.72 |
| NM_004518.2 | KCNQ2 | ILMN_136951 | 1.72 |
| NM_031845.2 | MAP2 | ILMN_38825 | 1.71 |
| NM_002604.1 | PDE7A | ILMN_3430 | 1.71 |
| NM_015477.1 | SIN3A | ILMN_14108 | 1.71 |
| NM_001002878.1 | THOC5 | ILMN_13820 | 1.71 |
| NM_002926.3 | RGS12 | ILMN_161894 | 1.7 |
| NM_177972.1 | TUB | ILMN_11520 | 1.7 |
| NM_182533.2 | C1orf86 | ILMN_2880 | 1.7 |
| NM_015001.2 | SPEN | ILMN_180751 | 1.7 |
| NM_006925.3 | SFRS5 | ILMN_34497 | 1.7 |
| NM_001012516.1 | ITM2C | ILMN_27531 | 1.7 |
| NM_025250.2 | TTYH3 | ILMN_22026 | 1.69 |
| NM_172014.1 | TNFSF14 | ILMN_9666 | 1.69 |
| NM_003275.2 | TMOD1 | ILMN_1052 | 1.69 |
| NM_172249.1 | CSF2RA | ILMN_5061 | 1.69 |
| NM_201559.2 | FOXO3 | ILMN_15525 | 1.69 |
| NM_003108.3 | SOX11 | ILMN_28038 | 1.69 |
| NM_177965.2 | C8orf37 | ILMN_11118 | 1.69 |
| NM_000093.3 | COL5A1 | ILMN_31902 | 1.68 |
| NM_001357.2 | DHX9 | ILMN_7196 | 1.68 |
| XM_936103.1 | LOC642033 | ILMN_33652 | 1.68 |
| NM_001002878.1 | THOC5 | ILMN_13820 | 1.68 |
| NM_001014432.1 | AKT1 | ILMN_4841 | 1.68 |
| NM_001048201.1 | UHRF1 | ILMN_162952 | 1.67 |
| NM_001031685.2 | TP53BP2 | ILMN_9205 | 1.66 |
| NM_014329.3 | EDC4 | ILMN_21643 | 1.66 |
| NM_017833.2 | C21orf55 | ILMN_6782 | 1.66 |
| NM_003821.5 | RIPK2 | ILMN_19402 | 1.65 |
| NM_025241.1 | UBXD1 | ILMN_10771 | 1.65 |
| NM_006426.1 | DPYSL4 | ILMN_175746 | 1.65 |
| NM_005319.3 | HIST1H1C | ILMN_18282 | 1.64 |
| NM_003342.4 | UBE2G1 | ILMN_179729 | 1.64 |
| NM_013291.2 | CPSF1 | ILMN_22094 | 1.64 |
| NM_004596.3 | SNRPA | ILMN_10495 | 1.64 |
| NM_014603.1 | CDR2L | ILMN_26231 | 1.64 |
| NM_015570.1 | AUTS2 | ILMN_4348 | 1.64 |
| NM_032195.1 | SON | ILMN_8462 | 1.63 |
| NM_182612.1 | PDDC1 | ILMN_26571 | 1.63 |
| NM_014862.3 | ARNT2 | ILMN_13881 | 1.63 |
| NM_007221.2 | PMF1 | ILMN_32628 | 1.63 |
| NM_020225.1 | STOX2 | ILMN_5597 | 1.63 |
| NM_013275.4 | ANKRD11 | ILMN_28595 | 1.63 |
| NM_007182.4 | RASSF1 | ILMN_8297 | 1.62 |
| NM_145701.1 | CDCA4 | ILMN_5601 | 1.62 |
| NM_080702.2 | BAT3 | ILMN_4429 | 1.62 |
| NM_016185.2 | HN1 | ILMN_3023 | 1.62 |
| NM_014853.2 | SGSM2 | ILMN_9226 | 1.62 |
| NM_053000.1 | TIGA1 | ILMN_2113 | 1.62 |
| NM_016466.4 | ANKRD39 | ILMN_23002 | 1.61 |
| NM_032389.3 | ARFGAP2 | ILMN_12944 | 1.61 |
| NM_015516.3 | TSKU | ILMN_29523 | 1.61 |
| NM_023080.1 | C8orf33 | ILMN_15901 | 1.6 |
| NM_019609.3 | CPXM1 | ILMN_26242 | 1.6 |
| NM_006571.2 | DCTN6 | ILMN_29012 | 1.6 |
| NM_005494.2 | DNAJB6 | ILMN_26714 | 1.6 |
| NM_018031.2 | WDR6 | ILMN_16845 | 1.6 |
| NM_005964.1 | MYH10 | ILMN_23305 | 1.6 |
| NM_007118.2 | TRIO | ILMN_23876 | 1.59 |
| NM_015113.3 | ZZEF1 | ILMN_11865 | 1.59 |
| NM_001089.1 | ABCA3 | ILMN_18800 | 1.59 |
| NM_016114.3 | ASB1 | ILMN_11707 | 1.59 |
| NM_032924.3 | ZNF3 | ILMN_25682 | 1.58 |
| NM_153812.1 | PHF13 | ILMN_27355 | 1.58 |
| NM_030806.3 | C1orf21 | ILMN_26434 | 1.58 |
| NM_016333.2 | SRRM2 | ILMN_21088 | 1.58 |
| NM_001013845.1 | CXorf40B | ILMN_170421 | 1.57 |
| NM_001037639.1 | PARL | ILMN_13356 | 1.57 |
| NM_006114.1 | TOMM40 | ILMN_29459 | 1.57 |
| NM_023009.4 | MARCKSL1 | ILMN_17241 | 1.57 |
| NM_032626.5 | RBBP6 | ILMN_19179 | 1.56 |
| NM_178496.2 | C3orf59 | ILMN_14619 | 1.56 |
| NM_006607.2 | PTTG2 | ILMN_25893 | 1.56 |
| NM_006445.3 | PRPF8 | ILMN_19289 | 1.56 |
| NM_015497.2 | TMEM87A | ILMN_181695 | 1.56 |
| XM_929980.2 | LOC647000 | ILMN_34401 | 1.56 |
| NM_001037283.1 | EIF3B | ILMN_1524 | 1.56 |
| NM_172097.1 | CATSPER2 | ILMN_23478 | 1.56 |
| NM_001013690.1 | LOC401720 | ILMN_21595 | 1.55 |
| NM_024653.3 | PRKRIP1 | ILMN_13077 | 1.55 |
| NM_005572.3 | LMNA | ILMN_12442 | 1.55 |
| NM_005243.2 | EWSR1 | ILMN_17011 | 1.55 |
| NM_015908.4 | ARS2 | ILMN_19647 | 1.55 |
| NM_000146.3 | FTL | ILMN_10967 | 1.55 |
| NM_001079514.1 | UBN1 | ILMN_172742 | 1.54 |
| NM_001407.2 | CELSR3 | ILMN_162782 | 1.54 |
| NM_001029862.1 | ANKRD30B | ILMN_7263 | 1.54 |
| NM_032970.2 | SEC22C | ILMN_28430 | 1.53 |
| NM_004526.2 | MCM2 | ILMN_183916 | 1.53 |
| NM_032309.2 | CHCHD5 | ILMN_23956 | 1.53 |
| NM_005475.1 | SH2B3 | ILMN_5130 | 1.52 |
| NM_139029.1 | CD151 | ILMN_44771 | 1.52 |
| XM_940209.1 | KIAA0194 | ILMN_37512 | 1.52 |
| NM_001430.3 | EPAS1 | ILMN_26360 | 1.52 |
| NM_014187.2 | HSPC171 | ILMN_20854 | 1.52 |
| NM_015180.4 | SYNE2 | ILMN_183979 | 1.51 |
| NM_015711.2 | GLTSCR1 | ILMN_18273 | 1.51 |
| NM_001093771.1 | TXNRD1 | ILMN_306750 | 1.51 |
| NM_002771.2 | PRSS3 | ILMN_19426 | 1.51 |
| NM_015164.1 | PLEKHM2 | ILMN_308799 | 1.51 |
| NM_014972.1 | TCF25 | ILMN_16231 | 1.51 |
| NM_006646.4 | WASF3 | ILMN_180336 | 1.5 |
| NM_024519.2 | FAM65A | ILMN_17641 | 1.5 |
| NM_012343.3 | NNT | ILMN_183201 | -1.5 |
| NM_018464.2 | CISD1 | ILMN_4843 | -1.5 |
| NM_182523.1 | C3orf68 | ILMN_4406 | -1.51 |
| NM_015017.3 | USP33 | ILMN_176756 | -1.51 |
| NM_001274.3 | CHEK1 | ILMN_25327 | -1.51 |
| XM_929862.1 | LOC646900 | ILMN_44661 | -1.51 |
| NM_013440.3 | PILRB | ILMN_177817 | -1.51 |
| NM_207012.2 | AP3M1 | ILMN_4081 | -1.51 |
| NM_001018109.1 | PIR | ILMN_13999 | -1.51 |
| NM_000414.1 | HSD17B4 | ILMN_23623 | -1.51 |
| NM_001040181.1 | CLDND1 | ILMN_169193 | -1.51 |
| NM_014629.2 | ARHGEF10 | ILMN_11481 | -1.52 |
| NM_022173.1 | TIA1 | ILMN_29910 | -1.52 |
| NM_152415.1 | VPS37A | ILMN_12702 | -1.52 |
| NM_006164.2 | NFE2L2 | ILMN_9669 | -1.52 |
| NM_002601.2 | PDE6D | ILMN_2430 | -1.52 |
| NM_024948.2 | C10orf97 | ILMN_20283 | -1.52 |
| NM_138444.3 | KCTD12 | ILMN_18501 | -1.52 |
| NM_021824.2 | NIF3L1 | ILMN_20423 | -1.52 |
| NM_003314.1 | TTC1 | ILMN_11292 | -1.53 |
| NM_001035513.1 | SDHC | ILMN_14364 | -1.53 |
| NM_001786.2 | CDC2 | ILMN_24793 | -1.53 |
| NM_001099283.1 | ZNF239 | ILMN_306709 | -1.53 |
| NM_002079.1 | GOT1 | ILMN_19908 | -1.53 |
| NM_016086.2 | STYXL1 | ILMN_5068 | -1.53 |
| NM_003916.3 | AP1S2 | ILMN_3812 | -1.53 |
| NM_005087.2 | FXR1 | ILMN_18674 | -1.53 |
| NM_012170.2 | FBXO22 | ILMN_5718 | -1.54 |
| NM_003149.1 | STAC | ILMN_5832 | -1.54 |
| NM_001204.5 | BMPR2 | ILMN_29007 | -1.54 |
| NM_178136.1 | POLDIP3 | ILMN_21842 | -1.54 |
| NM_012428.2 | NPTN | ILMN_175751 | -1.54 |
| NM_031452.2 | FAM103A1 | ILMN_28104 | -1.54 |
| NM_014847.2 | UBAP2L | ILMN_163836 | -1.54 |
| NM_181503.1 | EXOSC8 | ILMN_1976 | -1.54 |
| NM_021132.1 | PPP3CB | ILMN_13398 | -1.54 |
| XM_930284.1 | LOC441763 | ILMN_36192 | -1.54 |
| NM_015449.2 | C1orf43 | ILMN_933 | -1.54 |
| NM_002157.1 | HSPE1 | ILMN_2612 | -1.54 |
| NM_012063.1 | DNM1L | ILMN_15529 | -1.55 |
| NM_080597.2 | OSBPL1A | ILMN_10951 | -1.55 |
| NM_016401.2 | C11orf73 | ILMN_22672 | -1.55 |
| NM_000240.2 | MAOA | ILMN_183788 | -1.55 |
| NM_019080.1 | NDFIP2 | ILMN_23410 | -1.55 |
| NM_015344.1 | LEPROTL1 | ILMN_4515 | -1.55 |
| NM_017948.4 | NOL8 | ILMN_25734 | -1.56 |
| NM_024920.3 | DNAJB14 | ILMN_12080 | -1.56 |
| NM_138794.2 | LYPLAL1 | ILMN_25005 | -1.56 |
| XM_925818.1 | LOC642282 | ILMN_41968 | -1.56 |
| NM_052849.2 | CCDC32 | ILMN_24282 | -1.56 |
| NM_024122.2 | APOO | ILMN_11248 | -1.56 |
| NM_018142.2 | INTS10 | ILMN_29623 | -1.56 |
| NM_004336.2 | BUB1 | ILMN_10590 | -1.56 |
| NM_001002246.1 | ANAPC11 | ILMN_5565 | -1.56 |
| NM_006636.3 | MTHFD2 | ILMN_23782 | -1.56 |
| NM_003234.1 | TFRC | ILMN_12909 | -1.56 |
| NM_023071.1 | SPATS2 | ILMN_10985 | -1.57 |
| NM_014033.3 | METTL7A | ILMN_40171 | -1.57 |
| NM_016107.3 | ZFR | ILMN_11954 | -1.57 |
| NM_000382.2 | ALDH3A2 | ILMN_15882 | -1.57 |
| NM_003945.3 | ATP6V0E1 | ILMN_8923 | -1.57 |
| NR_001449.1 | TRK1 | ILMN_6493 | -1.58 |
| NM_014169.2 | CHMP4A | ILMN_19959 | -1.58 |
| NM_015463.1 | C2orf32 | ILMN_1437 | -1.58 |
| NM_012342.2 | BAMBI | ILMN_8469 | -1.58 |
| NM_001040181.1 | CLDND1 | ILMN_169193 | -1.58 |
| NM_001126.2 | ADSS | ILMN_9592 | -1.58 |
| NM_005455.3 | ZRANB2 | ILMN_23948 | -1.58 |
| NM_152305.1 | KTELC1 | ILMN_15488 | -1.59 |
| NM_153208.1 | IQCK | ILMN_19518 | -1.59 |
| NM_003342.4 | UBE2G1 | ILMN_179729 | -1.59 |
| NM_003359.2 | UGDH | ILMN_3906 | -1.59 |
| NM_001031703.2 | TMEM103 | ILMN_40105 | -1.59 |
| NM_025205.3 | MED28 | ILMN_14574 | -1.59 |
| NM_014142.2 | NUDT5 | ILMN_1656 | -1.59 |
| NM_006547.2 | IGF2BP3 | ILMN_26370 | -1.59 |
| NM_153713.1 | LIX1L | ILMN_3572 | -1.6 |
| NM_001007793.1 | BUB3 | ILMN_5688 | -1.6 |
| XR_019071.1 | LOC642333 | ILMN_183964 | -1.6 |
| NM_018230.2 | NUP133 | ILMN_18277 | -1.6 |
| NM_019048.1 | ASNSD1 | ILMN_15624 | -1.6 |
| NM_005188.2 | CBL | ILMN_172998 | -1.61 |
| NM_148178.1 | C9orf23 | ILMN_3926 | -1.61 |
| NM_001001660.2 | LYRM5 | ILMN_21147 | -1.61 |
| NM_020918.3 | GPAM | ILMN_174762 | -1.61 |
| NM_005048.2 | PTH2R | ILMN_19398 | -1.61 |
| NM_006630.1 | ZNF234 | ILMN_29233 | -1.61 |
| NM_031885.2 | BBS2 | ILMN_12583 | -1.61 |
| NM_002703.3 | PPAT | ILMN_6778 | -1.61 |
| NM_002528.4 | NTHL1 | ILMN_15981 | -1.61 |
| NM_018847.2 | KLHL9 | ILMN_20376 | -1.61 |
| NM_005402.2 | RALA | ILMN_164730 | -1.61 |
| NM_001037675.1 | NBPF20 | ILMN_26956 | -1.61 |
| NM_145247.4 | C10orf78 | ILMN_1251 | -1.62 |
| NM_018685.2 | ANLN | ILMN_20921 | -1.62 |
| NM_001079669.1 | TMTC4 | ILMN_169613 | -1.62 |
| NM_032117.2 | MND1 | ILMN_28552 | -1.62 |
| NM_001359.1 | DECR1 | ILMN_11646 | -1.62 |
| NM_003633.1 | ENC1 | ILMN_15041 | -1.62 |
| NM_021633.2 | KLHL12 | ILMN_165002 | -1.62 |
| NM_005896.2 | IDH1 | ILMN_14217 | -1.63 |
| NM_016098.1 | BRP44L | ILMN_4349 | -1.63 |
| NM_144638.1 | TMEM42 | ILMN_27701 | -1.63 |
| NM_024604.1 | RPAP3 | ILMN_18478 | -1.63 |
| XM_934985.1 | LOC400879 | ILMN_31001 | -1.63 |
| NM_212552.2 | BOLA3 | ILMN_28776 | -1.63 |
| NM_006554.3 | MTX2 | ILMN_17112 | -1.63 |
| XM_001132317.1 | LOC401397 | ILMN_162098 | -1.63 |
| NM_013388.4 | PREB | ILMN_6913 | -1.64 |
| NM_024928.3 | OBFC1 | ILMN_29568 | -1.64 |
| NM_001931.2 | DLAT | ILMN_20014 | -1.64 |
| NM_018390.2 | PLCXD1 | ILMN_8273 | -1.64 |
| NM_139207.1 | NAP1L1 | ILMN_5405 | -1.64 |
| NM_016505.2 | ZCCHC17 | ILMN_164919 | -1.64 |
| NM_003344.2 | UBE2H | ILMN_163352 | -1.64 |
| NM_003403.3 | YY1 | ILMN_4019 | -1.64 |
| NM_181573.1 | RFC4 | ILMN_20643 | -1.64 |
| NR_003105.1 | ZWILCH | ILMN_166966 | -1.64 |
| NM_033406.2 | FBXO3 | ILMN_26668 | -1.65 |
| NM_138443.2 | CCDC5 | ILMN_18943 | -1.65 |
| NM_032280.1 | ZCCHC9 | ILMN_25119 | -1.65 |
| NM_032361.1 | THOC3 | ILMN_17969 | -1.65 |
| NM_032439.1 | PHYHIPL | ILMN_22045 | -1.65 |
| NR_002734.1 | PTTG3 | ILMN_6114 | -1.65 |
| NM_001039091.1 | PRPS2 | ILMN_23417 | -1.66 |
| NM_014223.2 | NFYC | ILMN_5936 | -1.66 |
| NM_018359.1 | UFSP2 | ILMN_19806 | -1.66 |
| NM_025137.3 | SPG11 | ILMN_165427 | -1.66 |
| NM_022477.2 | NDRG3 | ILMN_8447 | -1.66 |
| NM_032041.1 | NCALD | ILMN_13197 | -1.66 |
| NM_016076.3 | FAM152A | ILMN_176788 | -1.66 |
| NM_145063.2 | C6orf130 | ILMN_24761 | -1.66 |
| XM_001133534.1 | ATP1B3 | ILMN_163124 | -1.66 |
| NM_005776.2 | CNIH | ILMN_13019 | -1.66 |
| NM_181705.1 | LOC90624 | ILMN_11045 | -1.67 |
| NM_006963.3 | ZNF22 | ILMN_165495 | -1.67 |
| NM_003744.5 | NUMB | ILMN_24350 | -1.67 |
| NM_006265.1 | RAD21 | ILMN_171453 | -1.67 |
| NM_006195.4 | PBX3 | ILMN_23493 | -1.67 |
| XM_001133534.1 | ATP1B3 | ILMN_163124 | -1.67 |
| NM_021177.3 | LSM2 | ILMN_22587 | -1.67 |
| NM_012210.3 | TRIM32 | ILMN_14426 | -1.68 |
| NM_001896.2 | CSNK2A2 | ILMN_16798 | -1.68 |
| NM_014180.2 | MRPL22 | ILMN_29349 | -1.68 |
| NM_016248.2 | AKAP11 | ILMN_13368 | -1.68 |
| NM_016447.2 | MPP6 | ILMN_163134 | -1.69 |
| NM_018199.2 | EXDL2 | ILMN_4351 | -1.69 |
| NM_004582.2 | RABGGTB | ILMN_25242 | -1.69 |
| NM_018204.2 | CKAP2 | ILMN_168115 | -1.69 |
| NM_138787.2 | C11orf74 | ILMN_16125 | -1.69 |
| NM_001006684.1 | TCEAL8 | ILMN_12677 | -1.69 |
| NM_006838.3 | METAP2 | ILMN_9736 | -1.69 |
| NM_033402.3 | LRRCC1 | ILMN_15234 | -1.7 |
| NM_020342.1 | SLC39A10 | ILMN_13415 | -1.7 |
| NM_207352.2 | CYP4V2 | ILMN_30285 | -1.7 |
| NM_001011537.1 | FYTTD1 | ILMN_5513 | -1.7 |
| XM_930884.1 | LOC653080 | ILMN_32261 | -1.7 |
| NM_002717.2 | PPP2R2A | ILMN_24841 | -1.7 |
| NM_016603.1 | C5orf5 | ILMN_24500 | -1.7 |
| NM_005192.2 | CDKN3 | ILMN_4098 | -1.7 |
| NM_007111.3 | TFDP1 | ILMN_18662 | -1.7 |
| NM_031423.3 | NUF2 | ILMN_16808 | -1.71 |
| NM_007299.2 | BRCA1 | ILMN_1010 | -1.71 |
| NM_014252.1 | SLC25A15 | ILMN_139066 | -1.71 |
| NM_002413.3 | MGST2 | ILMN_8759 | -1.71 |
| NM_002396.3 | ME2 | ILMN_176679 | -1.71 |
| NM_001786.2 | CDC2 | ILMN_24793 | -1.71 |
| NM_015702.1 | C2orf25 | ILMN_8271 | -1.71 |
| NM_018844.2 | BCAP29 | ILMN_24686 | -1.72 |
| NM_174921.1 | C4orf34 | ILMN_6140 | -1.72 |
| NM_005896.2 | IDH1 | ILMN_14217 | -1.72 |
| NM_001067.2 | TOP2A | ILMN_19849 | -1.72 |
| NM_000282.2 | PCCA | ILMN_6045 | -1.73 |
| NM_080653.3 | ATP6V1E2 | ILMN_5551 | -1.73 |
| NM_003642.2 | HAT1 | ILMN_24074 | -1.73 |
| NM_015127.3 | CLCC1 | ILMN_19789 | -1.73 |
| NM_133375.2 | DIS3L | ILMN_29373 | -1.73 |
| NM_024527.4 | ABHD8 | ILMN_23791 | -1.73 |
| NM_152902.3 | TIPRL | ILMN_13476 | -1.73 |
| NM_017934.4 | PHIP | ILMN_171544 | -1.73 |
| NM_016245.2 | HSD17B11 | ILMN_12219 | -1.73 |
| NM_177983.1 | PPM1G | ILMN_878 | -1.74 |
| NM_002755.2 | MAP2K1 | ILMN_164648 | -1.74 |
| NM_024570.1 | RNASEH2B | ILMN_20578 | -1.74 |
| NM_003659.1 | AGPS | ILMN_138634 | -1.74 |
| NM_006837.2 | COPS5 | ILMN_480 | -1.74 |
| NM_138458.2 | WDR92 | ILMN_37809 | -1.75 |
| NM_004886.3 | APBA3 | ILMN_4538 | -1.75 |
| NM_017819.2 | RG9MTD1 | ILMN_26970 | -1.75 |
| NM_001042581.1 | SNUPN | ILMN_176923 | -1.75 |
| NM_013263.2 | BRD7 | ILMN_14335 | -1.75 |
| NM_016248.2 | AKAP11 | ILMN_13368 | -1.75 |
| NM_003100.2 | SNX2 | ILMN_164977 | -1.75 |
| NM_018334.3 | LRRN3 | ILMN_174401 | -1.75 |
| NM_004239.1 | TRIP11 | ILMN_402 | -1.76 |
| NM_024632.4 | SAP30L | ILMN_18384 | -1.76 |
| NM_053067.1 | UBQLN1 | ILMN_9768 | -1.76 |
| NM_020186.1 | ACN9 | ILMN_6296 | -1.76 |
| NM_006638.2 | RPP40 | ILMN_164636 | -1.76 |
| NM_018718.1 | TSGA14 | ILMN_11000 | -1.76 |
| NM_006190.3 | ORC2L | ILMN_182860 | -1.77 |
| NM_013367.2 | ANAPC4 | ILMN_16297 | -1.77 |
| NM_006036.2 | PREPL | ILMN_19664 | -1.77 |
| NM_006703.2 | NUDT3 | ILMN_25244 | -1.77 |
| NM_023940.2 | RASL11B | ILMN_7453 | -1.77 |
| NM_024540.2 | MRPL24 | ILMN_29128 | -1.77 |
| NM_001037277.1 | GGPS1 | ILMN_4748 | -1.77 |
| NM_005873.2 | RGS19 | ILMN_42727 | -1.78 |
| NM_001037442.1 | RUFY3 | ILMN_28746 | -1.78 |
| NM_021971.1 | GMPPB | ILMN_3929 | -1.78 |
| NM_016324.2 | ZNF274 | ILMN_6185 | -1.78 |
| NM_002896.1 | RBM4 | ILMN_11057 | -1.78 |
| NR_002182.1 | NACAP1 | ILMN_14666 | -1.78 |
| NM_021159.3 | RAP1GDS1 | ILMN_163791 | -1.78 |
| NM_001003793.1 | RBMS3 | ILMN_16411 | -1.78 |
| NM_016561.1 | BFAR | ILMN_23440 | -1.78 |
| NM_019852.3 | METTL3 | ILMN_13907 | -1.78 |
| NM_006353.2 | HMGN4 | ILMN_163483 | -1.78 |
| NM_001382.2 | DPAGT1 | ILMN_10306 | -1.79 |
| NM_001040202.1 | PAQR3 | ILMN_180841 | -1.79 |
| NM_033168.2 | B3GALNT1 | ILMN_29294 | -1.79 |
| NM_006629.3 | ZNF271 | ILMN_11823 | -1.79 |
| NM_006109.3 | PRMT5 | ILMN_41646 | -1.79 |
| NM_014473.2 | DIMT1L | ILMN_11480 | -1.79 |
| NM_022037.1 | TIA1 | ILMN_30157 | -1.79 |
| NM_177974.1 | CASC4 | ILMN_14927 | -1.8 |
| NM_020675.3 | SPC25 | ILMN_915 | -1.8 |
| NM_004567.2 | PFKFB4 | ILMN_163968 | -1.8 |
| NM_006391.1 | IPO7 | ILMN_28842 | -1.8 |
| NM_001077199.1 | SFRS12 | ILMN_180296 | -1.8 |
| NM_005032.3 | PLS3 | ILMN_1428 | -1.8 |
| NM_016390.2 | C9orf114 | ILMN_20184 | -1.8 |
| NM_007259.3 | VPS45 | ILMN_17689 | -1.8 |
| NM_014711.3 | CP110 | ILMN_179542 | -1.8 |
| NM_025191.2 | EDEM3 | ILMN_15796 | -1.81 |
| NM_018471.2 | ZC3H15 | ILMN_168262 | -1.81 |
| NM_024945.1 | RMI1 | ILMN_11713 | -1.81 |
| NM_020449.2 | THOC2 | ILMN_162047 | -1.81 |
| NM_024775.9 | GEMIN6 | ILMN_23187 | -1.81 |
| NR_002308.1 | LOC442454 | ILMN_309609 | -1.81 |
| NM_138418.2 | C16orf14 | ILMN_9509 | -1.82 |
| NM_018186.2 | C1orf112 | ILMN_5134 | -1.82 |
| NM_006828.2 | ASCC3 | ILMN_174599 | -1.82 |
| NM_017735.3 | TTC27 | ILMN_24080 | -1.82 |
| NM_052865.2 | C20orf72 | ILMN_24256 | -1.82 |
| NM_001048172.1 | MUTYH | ILMN_164733 | -1.82 |
| NM_002669.2 | PLRG1 | ILMN_22972 | -1.82 |
| NM_003677.3 | DENR | ILMN_181187 | -1.82 |
| NM_004734.2 | DCLK1 | ILMN_178525 | -1.83 |
| NM_032180.1 | FLJ13305 | ILMN_5829 | -1.83 |
| XM_936354.2 | LOC642197 | ILMN_44406 | -1.83 |
| NM_020749.3 | MTUS1 | ILMN_4658 | -1.83 |
| NM_000390.2 | CHM | ILMN_15865 | -1.83 |
| NM_006807.3 | CBX1 | ILMN_162583 | -1.83 |
| NM_015702.1 | C2orf25 | ILMN_8271 | -1.83 |
| NM_002094.2 | GSPT1 | ILMN_5039 | -1.83 |
| NM_130811.1 | SNAP25 | ILMN_30021 | -1.83 |
| NM_144641.1 | PPM1M | ILMN_4690 | -1.84 |
| NM_175069.1 | APTX | ILMN_7416 | -1.84 |
| NM_018229.2 | C14orf108 | ILMN_22880 | -1.84 |
| NM_003924.2 | PHOX2B | ILMN_172224 | -1.84 |
| NM_001010982.2 | AFMID | ILMN_5520 | -1.85 |
| NM_153768.1 | CABYR | ILMN_9439 | -1.85 |
| NM_181839.1 | PKIA | ILMN_19703 | -1.85 |
| NM_020782.1 | KLHDC5 | ILMN_174169 | -1.85 |
| NM_005389.1 | PCMT1 | ILMN_26580 | -1.85 |
| NM_021127.1 | PMAIP1 | ILMN_25637 | -1.86 |
| NM_001077268.1 | ZFYVE19 | ILMN_175347 | -1.86 |
| NM_020186.1 | ACN9 | ILMN_6296 | -1.86 |
| NM_080651.1 | MED30 | ILMN_7158 | -1.86 |
| XM_945579.1 | LOC649555 | ILMN_31701 | -1.86 |
| NM_001042549.1 | NSL1 | ILMN_164300 | -1.86 |
| NM_015200.1 | PDS5A | ILMN_30113 | -1.87 |
| NM_006712.3 | FASTK | ILMN_11299 | -1.87 |
| XM_936354.2 | LOC642197 | ILMN_44406 | -1.87 |
| NM_144563.2 | RPIA | ILMN_23078 | -1.87 |
| NM_014161.2 | MRPL18 | ILMN_14120 | -1.87 |
| NM_024516.2 | C16orf53 | ILMN_20272 | -1.88 |
| NM_014885.3 | ANAPC10 | ILMN_2970 | -1.88 |
| NM_198527.2 | HDDC3 | ILMN_29602 | -1.88 |
| NM_014939.2 | KIAA1012 | ILMN_20638 | -1.88 |
| NM_001009551.1 | CNIH | ILMN_12906 | -1.88 |
| XM_371655.3 | LOC389137 | ILMN_163284 | -1.89 |
| NM_020116.2 | FSTL5 | ILMN_178729 | -1.89 |
| NM_180981.1 | MRPL52 | ILMN_3474 | -1.89 |
| NM_015462.3 | NOL11 | ILMN_5347 | -1.89 |
| NM_001002800.1 | SMC4 | ILMN_16070 | -1.89 |
| NM_006004.1 | UQCRH | ILMN_138507 | -1.89 |
| NM_006601.4 | PTGES3 | ILMN_3176 | -1.89 |
| NM_001827.1 | CKS2 | ILMN_14702 | -1.89 |
| NM_001031677.2 | RAB24 | ILMN_25731 | -1.9 |
| NM_018982.3 | YIPF1 | ILMN_19321 | -1.9 |
| NM_012460.2 | TIMM9 | ILMN_9968 | -1.9 |
| NM_005339.3 | HIP2 | ILMN_24744 | -1.9 |
| NM_001009894.2 | C12orf29 | ILMN_26574 | -1.9 |
| NM_018492.2 | PBK | ILMN_174875 | -1.9 |
| NM_177949.1 | ARMCX2 | ILMN_13791 | -1.9 |
| NM_015986.2 | CRLF3 | ILMN_22668 | -1.91 |
| NM_020150.3 | SAR1A | ILMN_17495 | -1.91 |
| NM_005926.2 | MFAP1 | ILMN_20656 | -1.91 |
| NM_175071.1 | APTX | ILMN_6739 | -1.91 |
| NM_080546.3 | SLC44A1 | ILMN_23525 | -1.91 |
| NM_201443.1 | TEAD4 | ILMN_21735 | -1.92 |
| NM_022473.1 | ZFP106 | ILMN_6305 | -1.92 |
| NM_197956.1 | C9orf90 | ILMN_16848 | -1.92 |
| NM_014060.1 | MCTS1 | ILMN_13725 | -1.92 |
| NM_058246.3 | DNAJB6 | ILMN_7651 | -1.92 |
| NM_018640.3 | LMO3 | ILMN_15180 | -1.92 |
| NM_015938.3 | NMD3 | ILMN_25169 | -1.93 |
| NM_001005498.2 | RHBDF2 | ILMN_23030 | -1.93 |
| NM_013235.3 | RNASEN | ILMN_11283 | -1.93 |
| NM_000016.2 | ACADM | ILMN_161879 | -1.93 |
| NM_021800.2 | DNAJC12 | ILMN_15911 | -1.93 |
| NM_138484.2 | SGOL1 | ILMN_14008 | -1.94 |
| NM_024945.2 | RMI1 | ILMN_11713 | -1.94 |
| NM_015935.4 | KIAA0859 | ILMN_172647 | -1.94 |
| NM_019592.5 | RNF20 | ILMN_3024 | -1.94 |
| NM_024056.2 | TMEM106C | ILMN_7003 | -1.94 |
| NM_001112.2 | ADARB1 | ILMN_30004 | -1.95 |
| NM_033117.2 | RBM18 | ILMN_8277 | -1.95 |
| NM_133371.2 | MYOZ3 | ILMN_21305 | -1.95 |
| NM_021222.1 | PRUNE | ILMN_27601 | -1.95 |
| NM_007308.1 | SNCA | ILMN_2235 | -1.95 |
| NM_016129.2 | COPS4 | ILMN_22296 | -1.95 |
| NM_152740.2 | HIBADH | ILMN_28448 | -1.95 |
| NM_006118.3 | HAX1 | ILMN_27167 | -1.95 |
| NM_016332.2 | SEPX1 | ILMN_7309 | -1.95 |
| NM_030771.1 | CCDC34 | ILMN_2645 | -1.95 |
| NM_022087.2 | GALNT11 | ILMN_5237 | -1.96 |
| NM_018098.4 | ECT2 | ILMN_6436 | -1.96 |
| NM_006713.2 | SUB1 | ILMN_27043 | -1.96 |
| NM_005805.3 | PSMD14 | ILMN_5019 | -1.96 |
| NM_176787.4 | PIGN | ILMN_163610 | -1.97 |
| NM_203291.1 | RBBP8 | ILMN_1238 | -1.97 |
| NM_025155.1 | PAAF1 | ILMN_13656 | -1.97 |
| NM_001827.1 | CKS2 | ILMN_14702 | -1.97 |
| NM_018229.2 | C14orf108 | ILMN_180528 | -1.98 |
| NM_001031744.1 | LOC158160 | ILMN_21155 | -1.98 |
| NR_003144.1 | LOC723972 | ILMN_180363 | -1.98 |
| NM_017910.2 | FLJ20628 | ILMN_29305 | -1.98 |
| NM_001204.5 | BMPR2 | ILMN_169269 | -1.98 |
| NM_001040876.1 | ABCE1 | ILMN_180762 | -1.98 |
| NM_012224.1 | NEK1 | ILMN_26831 | -1.98 |
| NM_022334.3 | ITGB1BP1 | ILMN_13169 | -1.99 |
| NM_022902.2 | SLC30A5 | ILMN_24834 | -1.99 |
| NM_001004051.1 | GPRASP2 | ILMN_8440 | -1.99 |
| NM_033001.1 | GTF2I | ILMN_3161 | -1.99 |
| NM_172177.1 | MRPL42 | ILMN_11502 | -1.99 |
| NM_001012413.1 | SGOL1 | ILMN_14464 | -2 |
| NM_014109.2 | ATAD2 | ILMN_172027 | -2 |
| NM_031885.2 | BBS2 | ILMN_12583 | -2 |
| NM_005642.2 | TAF7 | ILMN_19672 | -2 |
| XM_942289.1 | LOC652685 | ILMN_46541 | -2 |
| NM_032448.1 | FAM120B | ILMN_10767 | -2 |
| NM_006759.3 | UGP2 | ILMN_24416 | -2 |
| NM_144594.1 | GTSF1 | ILMN_17221 | -2.01 |
| NM_006101.1 | NDC80 | ILMN_14098 | -2.01 |
| NM_017953.2 | C1orf181 | ILMN_20839 | -2.01 |
| NM_022048.3 | CSNK1G1 | ILMN_19512 | -2.01 |
| NM_032497.1 | ZNF559 | ILMN_11090 | -2.01 |
| NM_001071775.1 | LOC440145 | ILMN_163591 | -2.01 |
| NM_015423.2 | AASDHPPT | ILMN_25075 | -2.01 |
| NM_000599.2 | IGFBP5 | ILMN_168089 | -2.01 |
| NM_003601.2 | SMARCA5 | ILMN_173755 | -2.02 |
| NM_016308.1 | CMPK1 | ILMN_12452 | -2.02 |
| NM_177424.2 | STX12 | ILMN_18776 | -2.02 |
| NM_176866.2 | PPA2 | ILMN_15275 | -2.02 |
| NM_139048.2 | HLTF | ILMN_6020 | -2.02 |
| NM_024079.4 | ALG8 | ILMN_28615 | -2.02 |
| NM_014184.2 | CNIH4 | ILMN_9903 | -2.02 |
| NM_016018.4 | PHF20L1 | ILMN_164472 | -2.03 |
| NM_001039141.1 | TRIOBP | ILMN_34620 | -2.03 |
| NM_021925.2 | C2orf43 | ILMN_2189 | -2.03 |
| NM_005536.2 | IMPA1 | ILMN_1396 | -2.03 |
| NM_000527.2 | LDLR | ILMN_10126 | -2.03 |
| NM_020871.3 | LRCH2 | ILMN_163747 | -2.04 |
| NM_181876.2 | PPP2R2C | ILMN_15268 | -2.04 |
| NM_006602.2 | TCFL5 | ILMN_12278 | -2.04 |
| NM_006608.1 | PHTF1 | ILMN_25225 | -2.04 |
| NM_016308.1 | CMPK1 | ILMN_12452 | -2.04 |
| NM_138361.3 | LRSAM1 | ILMN_21244 | -2.04 |
| NM_020123.2 | TM9SF3 | ILMN_17679 | -2.04 |
| NM_005131.2 | THOC1 | ILMN_19739 | -2.04 |
| NM_014168.2 | METTL5 | ILMN_9336 | -2.04 |
| NM_015271.2 | TRIM2 | ILMN_24873 | -2.04 |
| NM_133458.2 | ZFP90 | ILMN_174886 | -2.04 |
| NM_207037.1 | TCF12 | ILMN_182697 | -2.04 |
| NM_001005368.1 | ZNF32 | ILMN_181781 | -2.05 |
| NM_021970.2 | MAP2K1IP1 | ILMN_13073 | -2.05 |
| NM_003129.3 | SQLE | ILMN_183123 | -2.05 |
| NM_024647.4 | NUP43 | ILMN_28463 | -2.05 |
| NM_002247.2 | KCNMA1 | ILMN_24236 | -2.06 |
| XM_926112.2 | LOC441155 | ILMN_37470 | -2.06 |
| NM_001008390.1 | CGGBP1 | ILMN_163683 | -2.06 |
| NM_152755.1 | CNPY4 | ILMN_15383 | -2.07 |
| NM_016618.1 | KRCC1 | ILMN_25337 | -2.07 |
| NM_019095.3 | CRLS1 | ILMN_14031 | -2.07 |
| NM_003729.2 | RTCD1 | ILMN_11697 | -2.08 |
| NM_015475.3 | FAM98A | ILMN_16819 | -2.08 |
| NM_138781.2 | LOC113386 | ILMN_12569 | -2.08 |
| NM_015994.2 | ATP6V1D | ILMN_26737 | -2.08 |
| NM_002028.3 | FNTB | ILMN_171968 | -2.09 |
| NM_006117.2 | PECI | ILMN_7427 | -2.09 |
| NM_001017392.2 | SFRS14 | ILMN_17110 | -2.09 |
| NM_015938.2 | NMD3 | ILMN_25169 | -2.09 |
| NM_001037494.1 | DYNLL1 | ILMN_14802 | -2.09 |
| NM_012446.2 | SSBP2 | ILMN_5320 | -2.1 |
| NM_032783.3 | CBR4 | ILMN_15505 | -2.1 |
| NM_078629.1 | MSL3L1 | ILMN_29354 | -2.1 |
| NM_032299.2 | DCUN1D5 | ILMN_18117 | -2.1 |
| NM_002598.2 | PDCD2 | ILMN_5469 | -2.1 |
| XM_941876.1 | BRI3BP | ILMN_139088 | -2.11 |
| XM_936354.2 | LOC642197 | ILMN_44406 | -2.11 |
| NM_001080415.1 | SR140 | ILMN_169874 | -2.11 |
| NM_007342.1 | NUPL2 | ILMN_2154 | -2.11 |
| NM_002396.3 | ME2 | ILMN_176679 | -2.11 |
| NM_006178.1 | NSF | ILMN_23282 | -2.11 |
| NM_032574.2 | DPY30 | ILMN_18534 | -2.11 |
| NM_001788.4 | 7-Sep | ILMN_25070 | -2.12 |
| NM_001007239.1 | KIAA0859 | ILMN_25045 | -2.12 |
| NM_014161.2 | MRPL18 | ILMN_14120 | -2.12 |
| NM_022652.2 | DUSP6 | ILMN_5926 | -2.13 |
| NM_015969.2 | MRPS17 | ILMN_26133 | -2.13 |
| NM_012484.1 | HMMR | ILMN_17450 | -2.13 |
| NM_001042401.1 | C21orf51 | ILMN_179828 | -2.14 |
| NM_004130.2 | GYG1 | ILMN_22958 | -2.14 |
| NM_015525.2 | IBTK | ILMN_164723 | -2.14 |
| NM_024051.2 | C7orf24 | ILMN_2391 | -2.15 |
| NM_018122.3 | DARS2 | ILMN_183877 | -2.16 |
| NM_018443.2 | ZNF302 | ILMN_26438 | -2.16 |
| NM_001005369.1 | MTIF2 | ILMN_165311 | -2.17 |
| NM_001077394.1 | DPH5 | ILMN_175087 | -2.18 |
| NM_001007230.1 | SPOP | ILMN_12838 | -2.18 |
| NM_016551.1 | TM7SF3 | ILMN_7797 | -2.19 |
| NM_018353.3 | C14orf106 | ILMN_5745 | -2.19 |
| NM_014039.2 | C11orf54 | ILMN_4783 | -2.2 |
| NR_001445.1 | RN7SK | ILMN_14457 | -2.2 |
| NM_005830.2 | MRPS31 | ILMN_6293 | -2.2 |
| NM_002130.6 | HMGCS1 | ILMN_18980 | -2.2 |
| NM_138720.1 | HIST1H2BD | ILMN_17622 | -2.21 |
| NM_015252.2 | EHBP1 | ILMN_8575 | -2.21 |
| NM_016042.2 | EXOSC3 | ILMN_174330 | -2.21 |
| NM_004866.4 | SCAMP1 | ILMN_169565 | -2.21 |
| NM_022346.3 | NCAPG | ILMN_23620 | -2.21 |
| NM_005476.3 | GNE | ILMN_29772 | -2.22 |
| NM_183399.1 | RNF14 | ILMN_7292 | -2.22 |
| NM_152624.4 | DCP2 | ILMN_179808 | -2.22 |
| NM_004593.1 | SFRS10 | ILMN_22721 | -2.22 |
| NM_001099222.1 | IFT74 | ILMN_306953 | -2.23 |
| NM_003787.1 | NOL4 | ILMN_16134 | -2.23 |
| NM_152912.3 | MTIF3 | ILMN_16655 | -2.23 |
| NM_022757.3 | CCDC14 | ILMN_8560 | -2.23 |
| NM_032490.4 | C14orf142 | ILMN_166160 | -2.24 |
| NM_001031812.2 | CSNK1G3 | ILMN_17895 | -2.24 |
| NM_018357.2 | LARP6 | ILMN_25584 | -2.24 |
| NM_032138.3 | KBTBD7 | ILMN_181309 | -2.25 |
| NM_004982.2 | KCNJ8 | ILMN_29993 | -2.25 |
| NM_024516.2 | C16orf53 | ILMN_20272 | -2.25 |
| NM_018130.2 | SHQ1 | ILMN_29855 | -2.26 |
| NM_003503.2 | CDC7 | ILMN_20584 | -2.26 |
| NM_001008405.1 | BCAP29 | ILMN_24800 | -2.26 |
| NM_002906.3 | RDX | ILMN_28633 | -2.26 |
| XM_377476.4 | MGC57346 | ILMN_165970 | -2.26 |
| NM_001037317.1 | PAP2D | ILMN_181783 | -2.26 |
| NM_018256.2 | WDR12 | ILMN_14410 | -2.26 |
| NM_032476.2 | MRPS6 | ILMN_17239 | -2.26 |
| NM_182533.1 | C1orf86 | ILMN_2880 | -2.27 |
| NM_005653.3 | TFCP2 | ILMN_22607 | -2.28 |
| NM_018170.3 | P15RS | ILMN_174036 | -2.28 |
| XM_939682.1 | LOC149448 | ILMN_36821 | -2.29 |
| NM_016071.2 | MRPS33 | ILMN_4243 | -2.29 |
| NM_052879.3 | LARP4 | ILMN_2132 | -2.29 |
| NM_021800.2 | DNAJC12 | ILMN_177844 | -2.29 |
| NM_020401.2 | NUP107 | ILMN_24111 | -2.3 |
| NM_030881.2 | DDX17 | ILMN_28024 | -2.3 |
| NM_015314.2 | KIAA0895 | ILMN_28455 | -2.31 |
| NM_003129.3 | SQLE | ILMN_521 | -2.32 |
| NM_018244.3 | UQCC | ILMN_26543 | -2.32 |
| NR_001283.1 | TOP1P2 | ILMN_4755 | -2.33 |
| NM_016625.2 | RSRC1 | ILMN_14978 | -2.33 |
| NM_032728.2 | PPAPDC3 | ILMN_25638 | -2.34 |
| NM_001077395.1 | DPH5 | ILMN_181061 | -2.34 |
| NM_138316.2 | PANK1 | ILMN_406 | -2.34 |
| NM_006963.3 | ZNF22 | ILMN_165495 | -2.34 |
| NM_032290.2 | ANKRD32 | ILMN_15362 | -2.35 |
| NM_020147.2 | THAP10 | ILMN_182683 | -2.35 |
| NM_004866.4 | SCAMP1 | ILMN_169565 | -2.35 |
| NM_178812.2 | MTDH | ILMN_8610 | -2.36 |
| NM_032772.3 | ZNF503 | ILMN_2048 | -2.36 |
| NM_006366.2 | CAP2 | ILMN_27367 | -2.37 |
| NM_030934.3 | C1orf25 | ILMN_163506 | -2.37 |
| NM_175875.3 | SIX5 | ILMN_21099 | -2.37 |
| NM_001003722.1 | GLE1 | ILMN_19199 | -2.37 |
| NM_079837.2 | BANP | ILMN_8638 | -2.38 |
| NM_181837.1 | ORC3L | ILMN_3770 | -2.39 |
| NM_005644.2 | TAF12 | ILMN_3797 | -2.4 |
| NM_173659.2 | RPUSD3 | ILMN_28804 | -2.4 |
| NM_177968.2 | PPM1B | ILMN_29648 | -2.4 |
| NM_145274.2 | TMEM99 | ILMN_25105 | -2.41 |
| NM_173510.1 | CCDC117 | ILMN_21814 | -2.41 |
| XM_374020.4 | LOC375295 | ILMN_45377 | -2.41 |
| NM_199246.1 | CCNG1 | ILMN_9417 | -2.41 |
| NM_022496.3 | ACTR6 | ILMN_21794 | -2.41 |
| NM_016131.3 | RAB10 | ILMN_21971 | -2.41 |
| NM_018170.2 | P15RS | ILMN_174036 | -2.41 |
| NM_020119.3 | ZC3HAV1 | ILMN_13243 | -2.42 |
| NM_138798.1 | MITD1 | ILMN_27516 | -2.42 |
| NM_006282.2 | STK4 | ILMN_21491 | -2.42 |
| NM_003512.3 | HIST1H2AC | ILMN_26493 | -2.43 |
| NM_152713.2 | STT3A | ILMN_17585 | -2.43 |
| NM_012485.1 | HMMR | ILMN_16900 | -2.43 |
| NM_001080546.1 | LOC219854 | ILMN_168339 | -2.44 |
| NM_003746.1 | DNCL1 | ILMN_137049 | -2.44 |
| NM_033091.1 | TRIM4 | ILMN_8530 | -2.45 |
| XM_930579.2 | LOC653820 | ILMN_40990 | -2.45 |
| NM_153682.2 | PIGP | ILMN_18625 | -2.45 |
| NM_020242.1 | KIF15 | ILMN_6188 | -2.45 |
| NM_003628.3 | PKP4 | ILMN_11784 | -2.46 |
| NM_001166.3 | BIRC2 | ILMN_23760 | -2.46 |
| NM_000051.3 | ATM | ILMN_162851 | -2.47 |
| XM_931224.1 | LOC283683 | ILMN_45961 | -2.47 |
| NM_017489.1 | TERF1 | ILMN_164297 | -2.47 |
| NM_018131.3 | CEP55 | ILMN_6470 | -2.47 |
| NM_004365.2 | CETN3 | ILMN_25663 | -2.47 |
| NM_006036.3 | PREPL | ILMN_177009 | -2.48 |
| NM_198038.1 | NUDT9 | ILMN_12448 | -2.48 |
| NM_003002.1 | SDHD | ILMN_6353 | -2.48 |
| NM_004398.2 | DDX10 | ILMN_20779 | -2.48 |
| XM_498571.2 | LOC440160 | ILMN_33035 | -2.48 |
| NM_001813.2 | CENPE | ILMN_7509 | -2.49 |
| NM_014171.3 | CRIPT | ILMN_12903 | -2.49 |
| NM_005842.2 | SPRY2 | ILMN_19344 | -2.49 |
| NM_001031684.1 | SFRS7 | ILMN_7620 | -2.49 |
| NM_018062.2 | FANCL | ILMN_24728 | -2.5 |
| NM_003137.3 | SRPK1 | ILMN_19425 | -2.5 |
| XM_944321.1 | LOC402560 | ILMN_42108 | -2.51 |
| NM_207380.1 | C15orf52 | ILMN_1132 | -2.52 |
| NM_000913.3 | OPRL1 | ILMN_6491 | -2.53 |
| NM_178314.2 | RILPL1 | ILMN_1609 | -2.53 |
| NM_207418.2 | GCUD2 | ILMN_19354 | -2.53 |
| NM_020234.4 | DTWD1 | ILMN_3248 | -2.53 |
| NM_006117.2 | PECI | ILMN_7427 | -2.53 |
| NM_005833.2 | RABEPK | ILMN_4050 | -2.53 |
| NM_020153.2 | C11orf60 | ILMN_171038 | -2.54 |
| NM_005810.3 | KLRG1 | ILMN_12613 | -2.54 |
| NM_018428.2 | UTP6 | ILMN_18247 | -2.54 |
| NM_002486.4 | NCBP1 | ILMN_23411 | -2.56 |
| NM_001007027.2 | ALG8 | ILMN_176006 | -2.56 |
| NM_014612.3 | FAM120A | ILMN_14224 | -2.56 |
| NM_001813.2 | CENPE | ILMN_171982 | -2.57 |
| NM_004456.3 | EZH2 | ILMN_25740 | -2.57 |
| NM_018480.2 | TMEM126B | ILMN_18826 | -2.57 |
| NM_013330.3 | NME7 | ILMN_23456 | -2.57 |
| NM_003318.3 | TTK | ILMN_24472 | -2.58 |
| NM_001033503.1 | SAR1B | ILMN_16595 | -2.59 |
| NM_178439.3 | GMCL1 | ILMN_3285 | -2.59 |
| NM_198401.2 | ANKRD46 | ILMN_9031 | -2.62 |
| NM_018079.3 | SRBD1 | ILMN_28720 | -2.62 |
| NM_178439.3 | GMCL1 | ILMN_3285 | -2.62 |
| NM_001967.3 | EIF4A2 | ILMN_5908 | -2.63 |
| NM_006860.2 | RABL4 | ILMN_4559 | -2.64 |
| NM_000051.3 | ATM | ILMN_162851 | -2.64 |
| NM_001039937.1 | INTS6 | ILMN_38649 | -2.64 |
| NM_015360.3 | SKIV2L2 | ILMN_8825 | -2.66 |
| NM_001033566.1 | RHOT1 | ILMN_6821 | -2.68 |
| NM_002692.2 | POLE2 | ILMN_19705 | -2.68 |
| NM_152360.2 | ZNF573 | ILMN_23003 | -2.68 |
| NM_139286.3 | CDC26 | ILMN_18022 | -2.69 |
| NM_003916.3 | AP1S2 | ILMN_3812 | -2.69 |
| NM_018004.1 | TMEM45A | ILMN_30168 | -2.7 |
| NM_001012968.2 | SPIN4 | ILMN_4105 | -2.71 |
| NM_145644.1 | MRPL35 | ILMN_20736 | -2.71 |
| NM_014672.2 | KIAA0391 | ILMN_30096 | -2.71 |
| NM_198402.2 | PTPLB | ILMN_183743 | -2.73 |
| NM_199229.1 | RPE | ILMN_9823 | -2.74 |
| NM_005869.2 | SDCCAG10 | ILMN_3741 | -2.74 |
| NM_001033566.1 | RHOT1 | ILMN_6821 | -2.74 |
| NM_198047.1 | HIBCH | ILMN_24888 | -2.75 |
| NM_000628.3 | IL10RB | ILMN_26097 | -2.76 |
| NM_032547.1 | SCOC | ILMN_29814 | -2.77 |
| NM_006644.2 | HSPH1 | ILMN_1157 | -2.77 |
| NM_018846.2 | KLHL7 | ILMN_21425 | -2.79 |
| NM_014078.4 | MRPL13 | ILMN_17393 | -2.79 |
| NM_007280.1 | OIP5 | ILMN_18200 | -2.79 |
| NM_020236.2 | MRPL1 | ILMN_22997 | -2.8 |
| NM_005398.4 | PPP1R3C | ILMN_4487 | -2.82 |
| NM_024813.1 | RPAP2 | ILMN_23904 | -2.82 |
| NM_019858.1 | GPR162 | ILMN_27943 | -2.83 |
| NM_019116.2 | UBFD1 | ILMN_179383 | -2.86 |
| NM_018227.5 | UBA6 | ILMN_16506 | -2.86 |
| NM_006745.3 | SC4MOL | ILMN_2770 | -2.87 |
| NM_001938.2 | DR1 | ILMN_182864 | -2.89 |
| NM_004627.2 | WRB | ILMN_12263 | -2.92 |
| NM_015523.2 | REXO2 | ILMN_15016 | -2.92 |
| NM_016053.2 | CCDC53 | ILMN_25394 | -2.92 |
| NM_133646.2 | ZAK | ILMN_5666 | -2.93 |
| NM_015984.2 | UCHL5 | ILMN_3370 | -2.93 |
| NM_170784.1 | MKKS | ILMN_17701 | -2.94 |
| NM_000645.2 | AGL | ILMN_1173 | -2.95 |
| NM_014254.1 | TMEM5 | ILMN_26271 | -2.96 |
| NM_001121.2 | ADD3 | ILMN_4026 | -2.97 |
| NM_003800.3 | RNGTT | ILMN_17056 | -2.97 |
| NM_018164.1 | C12orf11 | ILMN_14707 | -2.98 |
| NM_198336.1 | INSIG1 | ILMN_12839 | -2.98 |
| NM_014750.3 | DLG7 | ILMN_4880 | -2.98 |
| NM_001017369.1 | SC4MOL | ILMN_2901 | -2.99 |
| NM_201280.1 | MUTED | ILMN_21576 | -3 |
| NM_002166.4 | ID2 | ILMN_28481 | -3 |
| NM_016048.1 | ISOC1 | ILMN_15311 | -3.02 |
| NM_013352.2 | DSE | ILMN_14589 | -3.03 |
| NM_007167.2 | ZMYM6 | ILMN_1275 | -3.03 |
| NM_024057.2 | NUP37 | ILMN_4147 | -3.04 |
| NM_001326.2 | CSTF3 | ILMN_27551 | -3.06 |
| NM_004252.2 | SLC9A3R1 | ILMN_1421 | -3.08 |
| NM_138720.1 | HIST1H2BD | ILMN_17622 | -3.08 |
| NM_017812.2 | CHCHD3 | ILMN_23539 | -3.11 |
| NM_014322.2 | OPN3 | ILMN_166169 | -3.14 |
| NM_030805.2 | LMAN2L | ILMN_1985 | -3.17 |
| NM_002643.3 | PIGF | ILMN_15261 | -3.19 |
| NM_002095.4 | GTF2E2 | ILMN_4316 | -3.22 |
| NM_018473.2 | THEM2 | ILMN_27212 | -3.27 |
| NM_003400.3 | XPO1 | ILMN_16600 | -3.27 |
| NM_213596.1 | FOXN4 | ILMN_25618 | -3.28 |
| NM_058216.1 | RAD51C | ILMN_2944 | -3.3 |
| NM_001889.2 | CRYZ | ILMN_30248 | -3.3 |
| NM_003715.2 | USO1 | ILMN_23419 | -3.31 |
| NM_001008566.1 | TPST2 | ILMN_13248 | -3.34 |
| NM_032226.2 | ZCCHC7 | ILMN_21489 | -3.34 |
| NM_006416.3 | SLC35A1 | ILMN_23284 | -3.37 |
| NM_001008783.1 | SLC35D3 | ILMN_16642 | -3.43 |
| NM_024678.3 | NARS2 | ILMN_13605 | -3.43 |
| NM_001007794.1 | CEPT1 | ILMN_15134 | -3.44 |
| NM_033212.2 | CCDC102A | ILMN_12942 | -3.47 |
| NM_001007157.1 | PHF14 | ILMN_2096 | -3.47 |
| NM_018480.2 | TMEM126B | ILMN_18826 | -3.5 |
| NM_079837.2 | BANP | ILMN_8638 | -3.56 |
| NM_198391.1 | FLRT3 | ILMN_23273 | -3.6 |
| NM_001040142.1 | SCN2A | ILMN_167124 | -3.62 |
| NM_014941.1 | MORC2 | ILMN_12502 | -3.63 |
| NM_020463.1 | SMEK2 | ILMN_21228 | -3.66 |
| NM_000856.3 | GUCY1A3 | ILMN_11680 | -3.69 |
| NM_058216.1 | RAD51C | ILMN_2944 | -3.7 |
| NM_138807.2 | C3orf31 | ILMN_9705 | -3.72 |
| NM_001539.2 | DNAJA1 | ILMN_5819 | -3.75 |
| NM_006597.3 | HSPA8 | ILMN_181529 | -3.75 |
| NM_017915.2 | C12orf48 | ILMN_42497 | -3.78 |
| NM_002166.4 | ID2 | ILMN_28481 | -3.79 |
| NM_000819.3 | GART | ILMN_22974 | -3.8 |
| NM_007198.2 | PROSC | ILMN_23472 | -3.91 |
| NM_033115.2 | MGC16169 | ILMN_16160 | -3.94 |
| NM_002167.2 | ID3 | ILMN_6829 | -3.97 |
| NM_014140.2 | SMARCAL1 | ILMN_19734 | -4.02 |
| NM_024090.1 | ELOVL6 | ILMN_11340 | -4.04 |
| NM_015948.2 | SLC35B3 | ILMN_20545 | -4.07 |
| NM_153201.1 | HSPA8 | ILMN_14829 | -4.23 |
| NM_004316.2 | ASCL1 | ILMN_23892 | -4.3 |
| NM_005836.2 | HRSP12 | ILMN_8062 | -4.36 |
| NM_080723.3 | NRSN1 | ILMN_178353 | -4.92 |
| NM_001007157.1 | PHF14 | ILMN_2096 | -5.07 |
| NM_005346.3 | HSPA1B | ILMN_25549 | -6.13 |
| NM_005345.4 | HSPA1A | ILMN_6623 | -6.9 |
