# Supplementary material for: Minimal Peroxide Exposure of Neuronal Cells Induces Multifaceted Adaptive Responses
Source: PLoS One. 2010 Dec 17;5(12):e14352. doi: 10.1371/journal.pone.0014352 (PMC3003681; doi:10.1371/journal.pone.0014352)
Supplement: Table S5 — MeCh-significantly regulated genes after 8 hours of stimulation in the control state SH-SY5Y cells. Each significantly regulated gene is described via its accession number (ACCESSION), Gene Symbol (SYMBOL), Illumina array transcript designation (TRANSCRIPT). For each gene the z-ratio of expression compared to untreated cells after 8 hours of ligand stimulation is displayed (CTL MeCh 8). (0.98 MB DOC) [file pone.0014352.s012.doc]

**Table S5. MeCh-significantly regulated genes after 8 hours of stimulation in the control state SH-SY5Y cells**. Each significantly regulated gene is described via its accession number (ACCESSION), Gene Symbol (SYMBOL), Illumina array transcript designation (TRANSCRIPT). For each gene the z-ratio of expression compared to un-treated cells after 8 hours of ligand stimulation is displayed (CTL MeCh 8).

| **ACCESSION** | **SYMBOL** | **TRANSCRIPT** | **CTL MeCh 8** |
| --- | --- | --- | --- |
| NM_000584.2 | IL8 | ILMN_179575 | 9.13 |
| NM_006157.2 | NELL1 | ILMN_2560 | 5.11 |
| NM_001040456.1 | RHBDD2 | ILMN_168345 | 4.92 |
| NM_001496.3 | GFRA3 | ILMN_8392 | 4.71 |
| NM_001040456.1 | RHBDD2 | ILMN_168345 | 4.1 |
| NM_002673.3 | PLXNB1 | ILMN_22628 | 3.96 |
| NM_000199.2 | SGSH | ILMN_7542 | 3.86 |
| NM_002160.2 | TNC | ILMN_14948 | 3.78 |
| NM_001001391.1 | CD44 | ILMN_10947 | 3.69 |
| NM_001077188.1 | HS6ST2 | ILMN_182242 | 3.65 |
| NM_003461.4 | ZYX | ILMN_2137 | 3.57 |
| NM_001012643.2 | LOC339344 | ILMN_6535 | 3.54 |
| NM_152398.2 | OCIAD2 | ILMN_18246 | 3.53 |
| NM_024663.3 | NPEPL1 | ILMN_175218 | 3.52 |
| NM_001033506.1 | CSTF3 | ILMN_27049 | 3.45 |
| NM_025058.3 | TRIM46 | ILMN_18492 | 3.4 |
| NM_004598.3 | SPOCK1 | ILMN_25886 | 3.4 |
| NM_033446.1 | FAM125B | ILMN_20760 | 3.33 |
| NM_001440.2 | EXTL3 | ILMN_10725 | 3.29 |
| NM_015433.2 | FAM119B | ILMN_17350 | 3.27 |
| NM_080491.1 | GAB2 | ILMN_3317 | 3.26 |
| NM_007346.2 | OGFR | ILMN_12520 | 3.24 |
| NM_015245.2 | ANKS1A | ILMN_25376 | 3.21 |
| NM_014771.2 | RNF40 | ILMN_5177 | 3.21 |
| NM_004145.2 | MYO9B | ILMN_25414 | 3.18 |
| NM_006045.1 | ATP9A | ILMN_176431 | 3.16 |
| NM_015690.2 | STK36 | ILMN_15506 | 3.16 |
| XM_001132754.1 | LOC728734 | ILMN_169578 | 3.15 |
| NM_002862.3 | PYGB | ILMN_21544 | 3.14 |
| NM_001347.2 | DGKQ | ILMN_27065 | 3.11 |
| NM_003458.3 | BSN | ILMN_22754 | 3.11 |
| NM_003200.1 | TCF3 | ILMN_173421 | 3.1 |
| NM_002230.1 | JUP | ILMN_3789 | 3.09 |
| NM_015016.1 | MAST3 | ILMN_308510 | 3.01 |
| NM_207035.1 | C1orf63 | ILMN_22593 | 3 |
| NM_005781.4 | TNK2 | ILMN_5336 | 2.99 |
| NM_002473.3 | MYH9 | ILMN_183555 | 2.98 |
| NM_001037533.1 | GON4L | ILMN_14180 | 2.96 |
| NM_014727.1 | MLL4 | ILMN_28047 | 2.96 |
| XM_942544.2 | INTS1 | ILMN_38896 | 2.94 |
| NM_004787.1 | SLIT2 | ILMN_12361 | 2.94 |
| XM_495939.3 | KIAA1545 | ILMN_40920 | 2.93 |
| NM_173854.4 | SLC41A1 | ILMN_2825 | 2.93 |
| NM_017514.2 | PLXNA3 | ILMN_162939 | 2.92 |
| NM_015144.2 | ZCCHC14 | ILMN_32176 | 2.89 |
| NM_022910.1 | NDRG4 | ILMN_8824 | 2.88 |
| NM_001078.2 | VCAM1 | ILMN_3875 | 2.84 |
| NM_007055.2 | POLR3A | ILMN_175644 | 2.83 |
| NM_014811.3 | KIAA0649 | ILMN_9360 | 2.83 |
| NM_015289.2 | VPS39 | ILMN_5610 | 2.83 |
| NM_017921.1 | NPLOC4 | ILMN_12904 | 2.82 |
| NM_016143.3 | NSFL1C | ILMN_20493 | 2.81 |
| NM_133328.2 | DEDD2 | ILMN_12562 | 2.81 |
| NM_015085.3 | GARNL4 | ILMN_163593 | 2.8 |
| NM_001040439.1 | MAPK8IP3 | ILMN_174436 | 2.8 |
| NM_012401.2 | PLXNB2 | ILMN_308861 | 2.8 |
| NM_020897.1 | HCN3 | ILMN_20127 | 2.79 |
| NM_025251.1 | KIAA1688 | ILMN_28510 | 2.79 |
| NM_153812.1 | PHF13 | ILMN_27355 | 2.79 |
| NM_004567.2 | PFKFB4 | ILMN_163968 | 2.79 |
| NM_014974.1 | DIP2C | ILMN_16576 | 2.78 |
| NM_024909.1 | C6orf134 | ILMN_21139 | 2.76 |
| NM_020773.1 | TBC1D14 | ILMN_165668 | 2.76 |
| NM_198925.1 | SEMA4B | ILMN_25258 | 2.76 |
| NM_019024.1 | HEATR5B | ILMN_25274 | 2.76 |
| NM_138499.3 | PWWP2B | ILMN_17964 | 2.76 |
| NM_002737.2 | PRKCA | ILMN_24085 | 2.75 |
| NM_015353.1 | KCTD2 | ILMN_13572 | 2.74 |
| NM_006185.2 | NUMA1 | ILMN_25058 | 2.73 |
| NM_002473.3 | MYH9 | ILMN_183555 | 2.73 |
| NM_007171.3 | POMT1 | ILMN_18145 | 2.72 |
| NM_002335.1 | LRP5 | ILMN_19887 | 2.72 |
| NM_032421.2 | CLIP2 | ILMN_14847 | 2.71 |
| NM_007144.2 | PCGF2 | ILMN_11878 | 2.71 |
| NM_001007246.1 | BRWD1 | ILMN_28841 | 2.71 |
| NM_012197.2 | RABGAP1 | ILMN_7110 | 2.7 |
| NM_178526.1 | SLC25A42 | ILMN_5992 | 2.7 |
| NM_014000.2 | VCL | ILMN_27566 | 2.69 |
| NM_020133.2 | AGPAT4 | ILMN_24920 | 2.69 |
| XM_001134411.1 | LOC441150 | ILMN_176720 | 2.69 |
| NM_002609.3 | PDGFRB | ILMN_25767 | 2.68 |
| NM_024048.2 | MGC3020 | ILMN_29369 | 2.68 |
| NM_020941.1 | KIAA1602 | ILMN_309224 | 2.67 |
| NM_006123.2 | IDS | ILMN_17605 | 2.66 |
| NM_013241.2 | FHOD1 | ILMN_14837 | 2.66 |
| NM_019892.3 | INPP5E | ILMN_11866 | 2.66 |
| NM_002254.6 | KIF3C | ILMN_22353 | 2.65 |
| NM_004444.4 | EPHB4 | ILMN_11176 | 2.65 |
| NM_005245.3 | FAT | ILMN_24617 | 2.65 |
| NM_032809.2 | FAM73B | ILMN_14652 | 2.63 |
| NM_021174.4 | KIAA1967 | ILMN_163008 | 2.63 |
| NM_024735.2 | FBXO31 | ILMN_17806 | 2.61 |
| NM_002959.4 | SORT1 | ILMN_165748 | 2.61 |
| NM_020859.1 | SHRM | ILMN_16821 | 2.6 |
| NM_004402.2 | DFFB | ILMN_14684 | 2.59 |
| NM_032017.1 | STK40 | ILMN_25410 | 2.59 |
| NM_003461.4 | ZYX | ILMN_2137 | 2.59 |
| NM_001002878.1 | THOC5 | ILMN_13820 | 2.58 |
| NM_001095.2 | ACCN2 | ILMN_27416 | 2.58 |
| NM_001013635.2 | LOC387856 | ILMN_30286 | 2.57 |
| NM_019024.1 | HEATR5B | ILMN_183109 | 2.57 |
| NM_015079.2 | TBC1D2B | ILMN_14085 | 2.56 |
| NM_153350.2 | FBXL16 | ILMN_17900 | 2.56 |
| NM_001042486.1 | DLGAP4 | ILMN_182055 | 2.56 |
| NM_002911.3 | UPF1 | ILMN_38133 | 2.55 |
| NM_014772.1 | KIAA0427 | ILMN_182540 | 2.55 |
| NM_182970.2 | RIMS4 | ILMN_16896 | 2.54 |
| NM_005224.2 | ARID3A | ILMN_18757 | 2.54 |
| NM_017991.3 | FLJ10081 | ILMN_469 | 2.52 |
| NM_006285.2 | TESK1 | ILMN_5444 | 2.49 |
| NM_177972.1 | TUB | ILMN_11520 | 2.48 |
| XM_371461.4 | KIAA1671 | ILMN_42090 | 2.48 |
| NM_001520.2 | GTF3C1 | ILMN_29638 | 2.48 |
| NM_012398.1 | PIP5K1C | ILMN_178370 | 2.48 |
| NM_004428.2 | EFNA1 | ILMN_14320 | 2.47 |
| NM_052918.3 | SORCS1 | ILMN_181700 | 2.47 |
| NM_024519.2 | FAM65A | ILMN_17641 | 2.46 |
| NM_005808.2 | CTDSPL | ILMN_510 | 2.46 |
| NM_173485.4 | TSHZ2 | ILMN_175561 | 2.45 |
| NM_001020820.1 | MYADM | ILMN_8340 | 2.44 |
| NM_015995.2 | KLF13 | ILMN_16226 | 2.44 |
| NM_005922.2 | MAP3K4 | ILMN_6743 | 2.44 |
| XM_001127981.1 | LOC728014 | ILMN_169164 | 2.44 |
| NM_144635.3 | FAM131A | ILMN_2542 | 2.44 |
| NM_173810.3 | TTC9C | ILMN_5250 | 2.44 |
| NM_006421.3 | ARFGEF1 | ILMN_164295 | 2.44 |
| NM_002972.1 | SBF1 | ILMN_22729 | 2.43 |
| NM_020983.2 | ADCY6 | ILMN_18413 | 2.43 |
| NM_001481.1 | GAS8 | ILMN_26809 | 2.42 |
| NM_006145.1 | DNAJB1 | ILMN_19740 | 2.42 |
| NM_004423.3 | DVL3 | ILMN_11726 | 2.42 |
| NM_005137.2 | DGCR2 | ILMN_6763 | 2.41 |
| NM_152322.2 | BTBD11 | ILMN_506 | 2.41 |
| NM_003938.5 | AP3D1 | ILMN_12013 | 2.4 |
| NM_006997.2 | TACC2 | ILMN_16130 | 2.4 |
| NM_003972.2 | BTAF1 | ILMN_8616 | 2.4 |
| NM_019106.4 | 3-Sep | ILMN_4065 | 2.39 |
| NM_004269.2 | MED27 | ILMN_16553 | 2.39 |
| NM_001677.3 | ATP1B1 | ILMN_25542 | 2.38 |
| NM_017999.4 | RNF31 | ILMN_170206 | 2.38 |
| NM_018174.4 | MAP1S | ILMN_25174 | 2.38 |
| NM_178831.4 | GATS | ILMN_18755 | 2.37 |
| NM_153273.3 | IHPK1 | ILMN_1661 | 2.37 |
| NM_133635.4 | POFUT2 | ILMN_24199 | 2.37 |
| NM_003906.3 | MCM3AP | ILMN_19614 | 2.37 |
| NM_015318.2 | ARHGEF18 | ILMN_4153 | 2.36 |
| NM_030665.3 | RAI1 | ILMN_176671 | 2.36 |
| NM_001032293.2 | ZNF207 | ILMN_21705 | 2.35 |
| NM_005605.3 | PPP3CC | ILMN_1406 | 2.35 |
| NM_138619.1 | GGA3 | ILMN_23307 | 2.35 |
| NM_001273.2 | CHD4 | ILMN_8213 | 2.34 |
| NM_000271.3 | NPC1 | ILMN_30618 | 2.34 |
| NM_079837.2 | BANP | ILMN_8638 | 2.34 |
| XM_001127981.1 | LOC728014 | ILMN_169164 | 2.33 |
| XM_940209.1 | KIAA0194 | ILMN_37512 | 2.33 |
| NM_004273.2 | CHST3 | ILMN_7808 | 2.33 |
| NM_020836.2 | BEGAIN | ILMN_10503 | 2.32 |
| NM_144781.1 | PDCD2 | ILMN_16269 | 2.32 |
| NM_024077.3 | SECISBP2 | ILMN_19156 | 2.32 |
| NM_003086.2 | SNAPC4 | ILMN_180505 | 2.31 |
| NM_014862.3 | ARNT2 | ILMN_13881 | 2.31 |
| NM_014014.2 | ASCC3L1 | ILMN_18834 | 2.31 |
| NM_031946.3 | CENTG3 | ILMN_29137 | 2.3 |
| NM_201575.1 | SEZ6L2 | ILMN_1006 | 2.3 |
| NM_007112.3 | THBS3 | ILMN_10000 | 2.29 |
| NM_016328.1 | GTF2IRD1 | ILMN_17028 | 2.29 |
| NM_001008219.1 | AMY1C | ILMN_28222 | 2.28 |
| NM_006011.3 | ST8SIA2 | ILMN_19287 | 2.28 |
| XM_930411.1 | LOC645099 | ILMN_37678 | 2.28 |
| NM_152511.3 | DUSP18 | ILMN_9044 | 2.28 |
| NM_020850.1 | RANBP10 | ILMN_21091 | 2.27 |
| NM_032800.1 | C1orf198 | ILMN_26304 | 2.27 |
| NM_014984.2 | AZI1 | ILMN_3856 | 2.27 |
| XM_926036.1 | LOC653103 | ILMN_32029 | 2.27 |
| NM_020248.2 | CTNNBIP1 | ILMN_23888 | 2.27 |
| NM_006795.2 | EHD1 | ILMN_17263 | 2.27 |
| NM_199245.1 | VAMP1 | ILMN_10901 | 2.27 |
| NM_001280.1 | CIRBP | ILMN_24327 | 2.26 |
| NM_019609.3 | CPXM1 | ILMN_26242 | 2.26 |
| NM_177999.1 | ASB6 | ILMN_13316 | 2.26 |
| NM_020920.2 | CHD8 | ILMN_3472 | 2.26 |
| NM_018209.2 | ARFGAP1 | ILMN_9646 | 2.26 |
| NM_020414.3 | DDX24 | ILMN_10146 | 2.26 |
| NM_145798.2 | OSBPL7 | ILMN_4611 | 2.25 |
| NM_021070.2 | LTBP3 | ILMN_918 | 2.25 |
| NM_133482.1 | RAD50 | ILMN_13599 | 2.25 |
| NM_001387.2 | DPYSL3 | ILMN_23309 | 2.25 |
| NM_000787.3 | DBH | ILMN_25962 | 2.24 |
| NM_014867.1 | KBTBD11 | ILMN_20625 | 2.24 |
| NM_014141.4 | CNTNAP2 | ILMN_176606 | 2.23 |
| NM_001080453.1 | INTS1 | ILMN_173681 | 2.21 |
| XM_290799.7 | ARHGAP23 | ILMN_162296 | 2.21 |
| NM_022167.2 | XYLT2 | ILMN_26042 | 2.21 |
| NM_197958.1 | LARP6 | ILMN_25698 | 2.21 |
| NM_022356.2 | LEPRE1 | ILMN_165693 | 2.21 |
| NM_001077621.1 | VPS37D | ILMN_172065 | 2.2 |
| NM_005688.2 | ABCC5 | ILMN_25223 | 2.2 |
| NM_178448.2 | C9orf140 | ILMN_24887 | 2.19 |
| NM_001013690.1 | LOC401720 | ILMN_21595 | 2.19 |
| NM_078481.2 | CD97 | ILMN_26363 | 2.19 |
| XM_945571.1 | ANKRD13D | ILMN_138370 | 2.18 |
| NM_138774.2 | C19orf22 | ILMN_15785 | 2.17 |
| NM_080670.2 | SLC35A4 | ILMN_8862 | 2.17 |
| NM_001012626.1 | LOC285074 | ILMN_21153 | 2.17 |
| NM_052899.2 | GPRIN1 | ILMN_15887 | 2.17 |
| NM_001079514.1 | UBN1 | ILMN_172742 | 2.16 |
| NM_016457.3 | PRKD2 | ILMN_23825 | 2.16 |
| NM_001144.4 | AMFR | ILMN_22219 | 2.16 |
| NM_017974.3 | ATG16L1 | ILMN_25151 | 2.15 |
| NM_018044.2 | NSUN5 | ILMN_895 | 2.15 |
| NM_001100422.1 | LOC26010 | ILMN_307385 | 2.15 |
| NM_173798.2 | ZCCHC12 | ILMN_7344 | 2.14 |
| NM_018416.2 | FOXJ2 | ILMN_165896 | 2.14 |
| NM_022307.1 | ICA1 | ILMN_5081 | 2.13 |
| NM_015125.3 | CIC | ILMN_179398 | 2.13 |
| NM_133635.4 | POFUT2 | ILMN_24199 | 2.13 |
| NM_001077664.1 | URG4 | ILMN_177599 | 2.13 |
| NM_015306.1 | USP24 | ILMN_309418 | 2.13 |
| NM_001002878.1 | THOC5 | ILMN_13820 | 2.12 |
| NM_019843.2 | EIF4ENIF1 | ILMN_5335 | 2.12 |
| NM_006999.3 | POLS | ILMN_866 | 2.12 |
| NM_015549.1 | PLEKHG3 | ILMN_28109 | 2.12 |
| NM_014347.1 | ZNF324 | ILMN_29920 | 2.12 |
| NM_023080.1 | C8orf33 | ILMN_15901 | 2.11 |
| NM_001012614.1 | CTBP1 | ILMN_21952 | 2.11 |
| NM_020728.1 | FAM62B | ILMN_19173 | 2.11 |
| NM_001039355.1 | SLC25A29 | ILMN_5110 | 2.11 |
| NM_015077.2 | SARM1 | ILMN_23861 | 2.11 |
| NM_015229.3 | KIAA0664 | ILMN_43322 | 2.11 |
| NM_015447.1 | CAMSAP1 | ILMN_815 | 2.11 |
| NM_003575.2 | ZNF282 | ILMN_3084 | 2.11 |
| NM_002926.3 | RGS12 | ILMN_161894 | 2.1 |
| NM_144967.2 | FLJ30058 | ILMN_29759 | 2.1 |
| NM_153047.1 | FYN | ILMN_25662 | 2.1 |
| NM_024792.1 | FAM57A | ILMN_28644 | 2.1 |
| NM_003047.2 | SLC9A1 | ILMN_166750 | 2.1 |
| NM_014603.1 | CDR2L | ILMN_26231 | 2.09 |
| NM_001009566.1 | CLSTN1 | ILMN_29098 | 2.09 |
| NM_198679.1 | RAPGEF1 | ILMN_177243 | 2.09 |
| NM_033631.2 | LUZP1 | ILMN_2667 | 2.09 |
| NM_021137.3 | TNFAIP1 | ILMN_14664 | 2.08 |
| NM_198971.1 | MIZF | ILMN_28647 | 2.08 |
| NM_014614.1 | PSME4 | ILMN_164803 | 2.08 |
| NM_003475.2 | RASSF7 | ILMN_12457 | 2.08 |
| NM_019058.2 | DDIT4 | ILMN_13176 | 2.07 |
| NM_181050.1 | AXIN1 | ILMN_6274 | 2.07 |
| NM_054013.2 | MGAT4B | ILMN_14669 | 2.07 |
| NM_203364.2 | CAPRIN1 | ILMN_9771 | 2.07 |
| NM_080730.2 | IFFO | ILMN_42149 | 2.07 |
| NM_016028.4 | SUV420H1 | ILMN_29861 | 2.07 |
| NM_173602.2 | DIP2B | ILMN_24944 | 2.07 |
| NM_003166.3 | SULT1A3 | ILMN_28760 | 2.07 |
| NM_144616.2 | JSRP1 | ILMN_9550 | 2.07 |
| NM_212469.1 | CHKA | ILMN_28401 | 2.06 |
| NM_002771.2 | PRSS3 | ILMN_19426 | 2.05 |
| NM_004468.3 | FHL3 | ILMN_183539 | 2.05 |
| NM_020447.3 | C15orf17 | ILMN_13536 | 2.05 |
| NM_016564.3 | CEND1 | ILMN_1492 | 2.05 |
| NM_004038.3 | AMY1A | ILMN_176350 | 2.05 |
| NM_020825.2 | CRAMP1L | ILMN_12672 | 2.05 |
| NM_015428.1 | ZNF473 | ILMN_167040 | 2.05 |
| NM_003355.2 | UCP2 | ILMN_14172 | 2.05 |
| NM_020655.2 | JPH3 | ILMN_26563 | 2.04 |
| NM_005886.2 | KATNB1 | ILMN_19547 | 2.04 |
| NM_033419.3 | PERLD1 | ILMN_12215 | 2.04 |
| NM_014921.3 | LPHN1 | ILMN_12017 | 2.03 |
| NM_014671.1 | UBE3C | ILMN_9296 | 2.03 |
| NM_021737.1 | CLCN6 | ILMN_6195 | 2.03 |
| NM_015500.1 | C2CD2 | ILMN_182120 | 2.03 |
| NM_015711.2 | GLTSCR1 | ILMN_18273 | 2.03 |
| NM_015113.3 | ZZEF1 | ILMN_11865 | 2.02 |
| NM_015695.2 | BRPF3 | ILMN_8164 | 2.02 |
| NM_001083585.1 | RABEP1 | ILMN_307418 | 2.02 |
| NM_173546.1 | KLHDC8B | ILMN_6513 | 2.01 |
| NM_020789.2 | IGSF9 | ILMN_22415 | 2.01 |
| NM_030808.3 | NDEL1 | ILMN_20362 | 2.01 |
| NM_024667.1 | VPS37B | ILMN_18457 | 2 |
| NM_001012516.1 | ITM2C | ILMN_27531 | 2 |
| XM_931359.2 | LOC338758 | ILMN_37634 | 2 |
| NM_006148.1 | LASP1 | ILMN_27039 | 2 |
| NM_031454.1 | SELO | ILMN_29590 | 2 |
| NM_016212.2 | TP53TG3 | ILMN_36480 | 2 |
| NM_001025243.1 | IRAK1 | ILMN_23652 | 1.99 |
| NM_015157.1 | PHLDB1 | ILMN_3997 | 1.99 |
| NM_002048.1 | GAS1 | ILMN_175833 | 1.99 |
| NM_001008408.3 | RBM33 | ILMN_165407 | 1.98 |
| NM_001819.1 | CHGB | ILMN_20449 | 1.98 |
| NM_033505.2 | SELI | ILMN_18750 | 1.98 |
| NM_001222.2 | CAMK2G | ILMN_25332 | 1.98 |
| NM_007001.1 | SLC35D2 | ILMN_17915 | 1.98 |
| NM_012119.3 | CCRK | ILMN_39653 | 1.98 |
| NM_032444.2 | BTBD12 | ILMN_23717 | 1.98 |
| NM_003589.2 | CUL4A | ILMN_28629 | 1.97 |
| NM_014329.3 | EDC4 | ILMN_21643 | 1.96 |
| NM_007055.2 | POLR3A | ILMN_1449 | 1.96 |
| NM_006426.1 | DPYSL4 | ILMN_175746 | 1.95 |
| NM_018263.4 | ASXL2 | ILMN_7971 | 1.95 |
| NM_003290.1 | TPM4 | ILMN_9334 | 1.95 |
| NM_014964.3 | EPN2 | ILMN_21232 | 1.95 |
| NM_003169.2 | SUPT5H | ILMN_15760 | 1.95 |
| NM_015338.4 | ASXL1 | ILMN_183479 | 1.95 |
| NM_005629.1 | SLC6A8 | ILMN_179044 | 1.95 |
| NM_001006115.2 | IHPK1 | ILMN_8379 | 1.94 |
| NM_145648.2 | SLC15A4 | ILMN_2279 | 1.94 |
| NM_006378.2 | SEMA4D | ILMN_24615 | 1.94 |
| NM_001076683.1 | UBTF | ILMN_170658 | 1.94 |
| NM_001031712.2 | TRMT11 | ILMN_8801 | 1.94 |
| NM_017741.3 | C4orf30 | ILMN_172318 | 1.93 |
| NM_032822.1 | FAM136A | ILMN_13374 | 1.93 |
| NM_139321.1 | ATRN | ILMN_6053 | 1.93 |
| NM_015155.1 | LARP5 | ILMN_21072 | 1.92 |
| NM_005194.2 | CEBPB | ILMN_4674 | 1.92 |
| NM_000945.3 | PPP3R1 | ILMN_26308 | 1.92 |
| NM_001003786.1 | LYK5 | ILMN_2367 | 1.92 |
| NM_001039877.1 | STRN4 | ILMN_42010 | 1.92 |
| NM_015346.2 | ZFYVE26 | ILMN_176163 | 1.92 |
| NM_004535.2 | MYT1 | ILMN_3667 | 1.91 |
| NM_003119.2 | SPG7 | ILMN_26332 | 1.91 |
| NM_005157.3 | ABL1 | ILMN_4033 | 1.91 |
| NM_213662.1 | STAT3 | ILMN_29673 | 1.91 |
| NM_006005.2 | WFS1 | ILMN_18545 | 1.91 |
| NM_006083.3 | IK | ILMN_27338 | 1.91 |
| NM_001013685.1 | LOC401357 | ILMN_29013 | 1.91 |
| NM_019106.4 | 3-Sep | ILMN_4065 | 1.91 |
| NM_014665.1 | LRRC14 | ILMN_166093 | 1.91 |
| NM_001092.3 | ABR | ILMN_23502 | 1.9 |
| NM_014975.1 | MAST1 | ILMN_14016 | 1.9 |
| NM_014747.2 | RIMS3 | ILMN_21581 | 1.9 |
| NM_207435.1 | FLJ40142 | ILMN_5279 | 1.9 |
| NM_001430.3 | EPAS1 | ILMN_26360 | 1.9 |
| XR_017397.1 | LOC644029 | ILMN_163901 | 1.9 |
| NM_001037334.1 | USP14 | ILMN_12721 | 1.89 |
| NM_006445.3 | PRPF8 | ILMN_19289 | 1.89 |
| NM_004140.3 | LLGL1 | ILMN_18915 | 1.89 |
| NM_024319.2 | C1orf35 | ILMN_28904 | 1.89 |
| NM_006715.2 | MAN2C1 | ILMN_685 | 1.89 |
| NM_001018111.1 | PODXL | ILMN_24120 | 1.89 |
| NM_001034194.1 | EXOSC9 | ILMN_26957 | 1.89 |
| NM_021090.3 | MTMR3 | ILMN_27578 | 1.89 |
| NM_078470.2 | COX15 | ILMN_13504 | 1.88 |
| NM_004311.2 | ARL3 | ILMN_15691 | 1.88 |
| NM_152716.1 | PATL1 | ILMN_11588 | 1.88 |
| NM_001032293.2 | ZNF207 | ILMN_21705 | 1.88 |
| NM_015477.1 | SIN3A | ILMN_14108 | 1.87 |
| NM_014567.2 | BCAR1 | ILMN_2703 | 1.87 |
| NM_001846.2 | COL4A2 | ILMN_179856 | 1.87 |
| NR_003659.1 | FAM39DP | ILMN_307683 | 1.86 |
| NM_016841.2 | MAPT | ILMN_3284 | 1.86 |
| NM_020452.2 | ATP8B2 | ILMN_19940 | 1.86 |
| NM_003434.3 | ZNF133 | ILMN_5864 | 1.86 |
| NM_003342.4 | UBE2G1 | ILMN_179729 | 1.86 |
| NM_032620.1 | GTPBP3 | ILMN_13264 | 1.85 |
| XM_001133202.1 | KIAA0363 | ILMN_166209 | 1.85 |
| NM_001076674.1 | TMUB2 | ILMN_176980 | 1.85 |
| NM_016948.2 | PARD6A | ILMN_23998 | 1.85 |
| NM_139179.1 | DAGLB | ILMN_1764 | 1.85 |
| NM_007185.3 | TNRC4 | ILMN_8091 | 1.85 |
| NM_024612.3 | DHX40 | ILMN_1864 | 1.85 |
| XM_928905.2 | LOC645937 | ILMN_41912 | 1.85 |
| NM_024836.1 | ZNF672 | ILMN_24640 | 1.85 |
| NM_015171.2 | XPO6 | ILMN_25747 | 1.84 |
| NM_020892.1 | DTX2 | ILMN_21612 | 1.84 |
| NM_031469.2 | SH3BGRL2 | ILMN_9801 | 1.84 |
| XR_017883.1 | FLJ90757 | ILMN_175182 | 1.83 |
| NM_023080.1 | C8orf33 | ILMN_15901 | 1.83 |
| NM_002333.1 | LRP3 | ILMN_12327 | 1.83 |
| NM_017757.2 | ZNF407 | ILMN_12747 | 1.83 |
| NM_005081.2 | ZNF142 | ILMN_11618 | 1.83 |
| NM_001692.3 | ATP6V1B1 | ILMN_28016 | 1.82 |
| NM_079837.2 | BANP | ILMN_8638 | 1.82 |
| NM_032991.2 | CASP3 | ILMN_29066 | 1.82 |
| NM_015316.2 | PPP1R13B | ILMN_13872 | 1.82 |
| NM_030803.5 | ATG16L1 | ILMN_24221 | 1.82 |
| NM_004359.1 | CDC34 | ILMN_9900 | 1.81 |
| NM_014338.3 | PISD | ILMN_28266 | 1.81 |
| NM_152705.1 | POLR1D | ILMN_28050 | 1.81 |
| NM_018223.1 | CHFR | ILMN_26892 | 1.81 |
| NM_014700.2 | RAB11FIP3 | ILMN_7754 | 1.8 |
| NM_000122.1 | ERCC3 | ILMN_17573 | 1.8 |
| NM_018697.3 | LANCL2 | ILMN_920 | 1.8 |
| NM_005572.3 | LMNA | ILMN_12442 | 1.8 |
| NM_007219.2 | RNF24 | ILMN_16137 | 1.8 |
| NM_015308.1 | FNBP4 | ILMN_25895 | 1.8 |
| NM_184231.1 | NCKIPSD | ILMN_15946 | 1.8 |
| NM_021626.1 | SCPEP1 | ILMN_22216 | 1.8 |
| NM_024525.2 | TTC13 | ILMN_164005 | 1.8 |
| NM_001001794.2 | FAM116B | ILMN_17332 | 1.79 |
| NM_018255.1 | ELP2 | ILMN_5596 | 1.79 |
| NM_006695.3 | RUNDC3A | ILMN_9016 | 1.79 |
| NM_016257.2 | HPCAL4 | ILMN_25356 | 1.79 |
| NM_016219.2 | MAN1B1 | ILMN_27649 | 1.79 |
| NM_017566.2 | KLHDC4 | ILMN_8527 | 1.79 |
| NM_022833.2 | FAM129B | ILMN_183377 | 1.79 |
| NM_015352.1 | POFUT1 | ILMN_7876 | 1.79 |
| NM_007148.3 | ZNF179 | ILMN_22425 | 1.79 |
| NM_015496.3 | KIAA1429 | ILMN_21574 | 1.79 |
| NM_005189.1 | CBX2 | ILMN_28525 | 1.78 |
| NM_052897.3 | MBD6 | ILMN_162772 | 1.78 |
| NM_001033028.1 | CYFIP1 | ILMN_6477 | 1.78 |
| NM_002657.2 | PLAGL2 | ILMN_23071 | 1.78 |
| NM_001845.4 | COL4A1 | ILMN_24359 | 1.78 |
| NM_021158.3 | TRIB3 | ILMN_21257 | 1.78 |
| NM_144999.2 | LRRC45 | ILMN_10648 | 1.78 |
| NM_022748.10 | TNS3 | ILMN_17676 | 1.78 |
| NM_017588.2 | WDR5 | ILMN_27310 | 1.77 |
| NM_022662.2 | ANAPC1 | ILMN_164277 | 1.77 |
| NM_020701.1 | ISY1 | ILMN_17522 | 1.76 |
| NM_002096.1 | GTF2F1 | ILMN_178379 | 1.76 |
| NM_024531.3 | GPR172A | ILMN_22128 | 1.76 |
| NM_153334.3 | SCARF2 | ILMN_13788 | 1.76 |
| NM_015517.3 | MIZF | ILMN_3194 | 1.76 |
| NM_177536.1 | SULT1A1 | ILMN_29763 | 1.76 |
| NM_005920.2 | MEF2D | ILMN_3465 | 1.76 |
| NM_001039705.1 | TRO | ILMN_32618 | 1.75 |
| NM_014849.2 | SV2A | ILMN_14178 | 1.75 |
| NM_015359.2 | SLC39A14 | ILMN_7176 | 1.75 |
| NM_016020.1 | TFB1M | ILMN_3033 | 1.75 |
| NM_002477.1 | MYL5 | ILMN_21416 | 1.75 |
| NM_007049.2 | BTN2A1 | ILMN_6995 | 1.75 |
| NM_175085.1 | GART | ILMN_19282 | 1.75 |
| NM_004393.2 | DAG1 | ILMN_16432 | 1.74 |
| NM_018249.4 | CDK5RAP2 | ILMN_9876 | 1.74 |
| NM_001093771.1 | TXNRD1 | ILMN_306750 | 1.74 |
| NM_017925.4 | DENND4C | ILMN_2455 | 1.74 |
| NM_152557.3 | ZNF746 | ILMN_25894 | 1.74 |
| NM_019015.1 | CSGlcA-T | ILMN_21838 | 1.74 |
| NM_001047.2 | SRD5A1 | ILMN_46190 | 1.74 |
| NM_134426.2 | SLC26A6 | ILMN_5076 | 1.74 |
| NM_021045.1 | ZNF248 | ILMN_27704 | 1.74 |
| NM_005169.2 | PHOX2A | ILMN_10858 | 1.73 |
| NM_003587.3 | DHX16 | ILMN_5547 | 1.73 |
| NM_001567.2 | INPPL1 | ILMN_20903 | 1.73 |
| NM_001012516.1 | ITM2C | ILMN_27531 | 1.73 |
| NM_001001132.1 | ITSN1 | ILMN_10040 | 1.73 |
| NM_016282.2 | AK3 | ILMN_6776 | 1.73 |
| NM_138462.2 | ZMYND19 | ILMN_797 | 1.73 |
| NM_005243.2 | EWSR1 | ILMN_17011 | 1.72 |
| NM_175859.1 | CTPS2 | ILMN_8874 | 1.72 |
| NM_032421.1 | CYLN2 | ILMN_14847 | 1.72 |
| NM_024881.3 | SLC35E1 | ILMN_23168 | 1.72 |
| NM_031845.2 | MAP2 | ILMN_38825 | 1.72 |
| NR_003083.2 | SLC6A10P | ILMN_172848 | 1.72 |
| NM_145730.1 | AP1B1 | ILMN_27296 | 1.72 |
| NM_183425.1 | RBM38 | ILMN_20092 | 1.72 |
| NM_001013258.1 | ZNF789 | ILMN_11535 | 1.72 |
| NM_015395.1 | DKFZP434B0335 | ILMN_11830 | 1.71 |
| NM_001933.3 | DLST | ILMN_162660 | 1.71 |
| NM_018992.2 | KCTD5 | ILMN_17737 | 1.71 |
| NM_014671.1 | UBE3C | ILMN_9296 | 1.71 |
| NM_002627.3 | PFKP | ILMN_16104 | 1.71 |
| NM_024884.1 | L2HGDH | ILMN_21427 | 1.71 |
| NM_016284.3 | CNOT1 | ILMN_169268 | 1.7 |
| NM_006796.1 | AFG3L2 | ILMN_29564 | 1.7 |
| NM_018246.2 | CCDC25 | ILMN_5229 | 1.7 |
| NM_001039842.1 | C17orf90 | ILMN_32693 | 1.7 |
| NM_199126.1 | ZNF585A | ILMN_9003 | 1.7 |
| NM_016376.3 | ANKFY1 | ILMN_8333 | 1.7 |
| NM_012326.2 | MAPRE3 | ILMN_1035 | 1.7 |
| NM_144498.1 | OSBPL2 | ILMN_180110 | 1.7 |
| NM_170783.1 | ZNRD1 | ILMN_1419 | 1.7 |
| NM_007184.3 | NISCH | ILMN_18778 | 1.69 |
| NM_022066.2 | UBE2O | ILMN_162853 | 1.69 |
| NM_004078.1 | CSRP1 | ILMN_25451 | 1.69 |
| NM_015332.3 | NUDCD3 | ILMN_26863 | 1.69 |
| NM_033426.2 | KIAA1737 | ILMN_24671 | 1.69 |
| NM_012143.2 | TFIP11 | ILMN_3675 | 1.69 |
| NM_003635.2 | NDST2 | ILMN_21220 | 1.69 |
| NM_022460.3 | HS1BP3 | ILMN_1874 | 1.69 |
| NM_145294.4 | WDR90 | ILMN_29490 | 1.68 |
| NM_001031685.2 | TP53BP2 | ILMN_9205 | 1.68 |
| NM_020418.2 | PCBP4 | ILMN_10036 | 1.68 |
| NM_033550.3 | TP53RK | ILMN_20784 | 1.68 |
| NM_001077523.1 | AP3D1 | ILMN_170396 | 1.67 |
| NM_002918.3 | RFX1 | ILMN_178968 | 1.67 |
| NM_004036.3 | ADCY3 | ILMN_26929 | 1.67 |
| NM_003793.2 | CTSF | ILMN_138148 | 1.67 |
| NM_001098673.1 | C12orf44 | ILMN_309179 | 1.67 |
| NM_002451.3 | MTAP | ILMN_163674 | 1.67 |
| NM_004924.3 | ACTN4 | ILMN_2269 | 1.67 |
| NM_003385.4 | VSNL1 | ILMN_14653 | 1.67 |
| NM_199169.1 | PMEPA1 | ILMN_13834 | 1.67 |
| NM_020148.2 | SPIRE1 | ILMN_2975 | 1.67 |
| NM_015164.1 | PLEKHM2 | ILMN_308799 | 1.66 |
| NM_052917.2 | GALNT13 | ILMN_180483 | 1.66 |
| NM_019001.2 | XRN1 | ILMN_8924 | 1.66 |
| NM_002376.4 | MARK3 | ILMN_182203 | 1.66 |
| XM_938667.1 | DEAF1 | ILMN_138757 | 1.66 |
| NM_001406.3 | EFNB3 | ILMN_17706 | 1.65 |
| NM_023072.1 | ZSWIM4 | ILMN_167043 | 1.64 |
| NM_018054.4 | ARHGAP17 | ILMN_9156 | 1.64 |
| NM_000319.3 | PEX5 | ILMN_29393 | 1.64 |
| NM_138452.1 | DHRS1 | ILMN_15545 | 1.64 |
| NM_174891.3 | C14orf79 | ILMN_22555 | 1.64 |
| NM_144599.3 | NIPA1 | ILMN_8853 | 1.64 |
| NM_001001787.1 | ATP1B1 | ILMN_10855 | 1.63 |
| NM_020944.2 | GBA2 | ILMN_5969 | 1.63 |
| NM_207435.1 | FLJ40142 | ILMN_5279 | 1.63 |
| NM_022830.1 | TUT1 | ILMN_6523 | 1.63 |
| NM_003846.1 | PEX11B | ILMN_20603 | 1.63 |
| NM_005089.1 | U2AF1L2 | ILMN_13409 | 1.63 |
| NM_182705.2 | FAM101B | ILMN_1388 | 1.63 |
| NM_032508.1 | TMEM185A | ILMN_24307 | 1.63 |
| NM_005177.3 | ATP6V0A1 | ILMN_28612 | 1.62 |
| NM_001035507.2 | AGBL5 | ILMN_24692 | 1.62 |
| NM_145648.1 | SLC15A4 | ILMN_2279 | 1.62 |
| NM_020807.1 | ZNF319 | ILMN_22764 | 1.62 |
| NM_015927.3 | TGFB1I1 | ILMN_27048 | 1.62 |
| NM_181724.1 | TMEM119 | ILMN_30233 | 1.61 |
| NM_015330.1 | SPECC1L | ILMN_168707 | 1.61 |
| NM_003565.1 | ULK1 | ILMN_2158 | 1.61 |
| NM_152666.1 | PLD5 | ILMN_25636 | 1.61 |
| NM_001080485.1 | ZNF275 | ILMN_180340 | 1.61 |
| NM_153756.1 | FNDC5 | ILMN_5239 | 1.61 |
| NM_013284.1 | POLM | ILMN_5705 | 1.61 |
| NM_032765.2 | TRIM52 | ILMN_13905 | 1.61 |
| NM_017818.2 | WDR8 | ILMN_13303 | 1.6 |
| NM_005940.3 | MMP11 | ILMN_16214 | 1.6 |
| NM_144736.3 | PRO1853 | ILMN_15591 | 1.6 |
| NM_001014999.1 | GIYD1 | ILMN_12782 | 1.6 |
| NM_030809.1 | FAM130A1 | ILMN_23790 | 1.6 |
| NM_001752.2 | CAT | ILMN_13962 | 1.6 |
| NM_139235.3 | NOL6 | ILMN_7349 | 1.6 |
| NM_014909.3 | VASH1 | ILMN_164012 | 1.6 |
| NM_024585.2 | ARMC7 | ILMN_163623 | 1.6 |
| NM_001013839.1 | EXOC7 | ILMN_25212 | 1.59 |
| NM_015062.3 | PPRC1 | ILMN_10445 | 1.59 |
| NM_004263.3 | SEMA4F | ILMN_177045 | 1.59 |
| NM_006371.3 | CRTAP | ILMN_180917 | 1.59 |
| NM_003836.4 | DLK1 | ILMN_13065 | 1.59 |
| NM_001040101.1 | D4S234E | ILMN_173747 | 1.58 |
| NM_004332.1 | BPHL | ILMN_27041 | 1.58 |
| NM_005147.3 | DNAJA3 | ILMN_10747 | 1.58 |
| NM_014805.2 | EPM2AIP1 | ILMN_9770 | 1.58 |
| NM_145056.1 | DACT3 | ILMN_8584 | 1.58 |
| NM_003778.3 | B4GALT4 | ILMN_1477 | 1.58 |
| NM_018403.4 | DCP1A | ILMN_27256 | 1.58 |
| NM_002406.2 | MGAT1 | ILMN_7618 | 1.57 |
| NM_005730.3 | CTDSP2 | ILMN_169361 | 1.57 |
| NM_004308.2 | ARHGAP1 | ILMN_4646 | 1.57 |
| NM_006465.2 | ARID3B | ILMN_4032 | 1.57 |
| NM_024545.2 | SAP130 | ILMN_2199 | 1.57 |
| NM_020724.1 | RNF150 | ILMN_26801 | 1.57 |
| NM_022742.3 | CCDC136 | ILMN_183005 | 1.57 |
| NM_003292.2 | TPR | ILMN_179238 | 1.57 |
| NM_001024071.1 | GCH1 | ILMN_14690 | 1.57 |
| NM_001009937.1 | SLC25A26 | ILMN_15004 | 1.57 |
| NM_018469.3 | TEX2 | ILMN_27579 | 1.57 |
| NM_145306.2 | C10orf35 | ILMN_13661 | 1.57 |
| NM_005380.4 | NBL1 | ILMN_21944 | 1.57 |
| NM_025074.4 | FRAS1 | ILMN_165073 | 1.57 |
| NM_005088.2 | SFRS17A | ILMN_26209 | 1.56 |
| NM_004634.2 | BRPF1 | ILMN_17537 | 1.56 |
| NM_014966.2 | DHX30 | ILMN_18565 | 1.56 |
| NM_000853.1 | GSTT1 | ILMN_22828 | 1.56 |
| NM_005560.3 | LAMA5 | ILMN_12588 | 1.55 |
| NM_002076.2 | GNS | ILMN_177670 | 1.55 |
| NM_020235.3 | BBX | ILMN_28437 | 1.55 |
| NM_032180.1 | FLJ13305 | ILMN_5829 | 1.55 |
| NM_001100417.1 | C14orf130 | ILMN_307543 | 1.55 |
| NM_012068.3 | ATF5 | ILMN_6490 | 1.55 |
| NM_173614.2 | NOMO2 | ILMN_1736 | 1.54 |
| NM_001037639.1 | PARL | ILMN_13356 | 1.54 |
| NM_004422.2 | DVL2 | ILMN_29320 | 1.54 |
| XM_001132495.1 | SLC26A11 | ILMN_167531 | 1.54 |
| NM_001010927.2 | TIAM2 | ILMN_9891 | 1.54 |
| NM_007075.3 | WDR45 | ILMN_25756 | 1.54 |
| NM_013336.3 | SEC61A1 | ILMN_9397 | 1.53 |
| NM_006084.4 | IRF9 | ILMN_163101 | 1.53 |
| NM_007284.3 | TWF2 | ILMN_717 | 1.53 |
| NM_138370.1 | LOC91461 | ILMN_24839 | 1.53 |
| NM_005090.2 | PLA2G4B | ILMN_6705 | 1.53 |
| NM_015340.3 | LARS2 | ILMN_11446 | 1.53 |
| NM_005436.2 | CCDC6 | ILMN_19206 | 1.53 |
| NM_002018.2 | FLII | ILMN_25947 | 1.52 |
| NM_001077442.1 | HNRNPC | ILMN_165238 | 1.52 |
| NM_002744.4 | PRKCZ | ILMN_21284 | 1.52 |
| NM_000434.2 | NEU1 | ILMN_23298 | 1.52 |
| NM_001032396.1 | PJA1 | ILMN_8226 | 1.51 |
| NM_013326.3 | C18orf8 | ILMN_162492 | 1.51 |
| NM_004206.2 | SEC22C | ILMN_15895 | 1.51 |
| NM_019887.3 | DIABLO | ILMN_21307 | 1.51 |
| NM_016463.5 | CXXC5 | ILMN_166144 | 1.51 |
| NM_006372.3 | SYNCRIP | ILMN_28470 | 1.51 |
| NM_005010.3 | NRCAM | ILMN_8955 | 1.51 |
| NM_017612.2 | ZCCHC8 | ILMN_30318 | 1.51 |
| NM_033426.2 | KIAA1737 | ILMN_176915 | 1.51 |
| XM_928464.1 | LOC146517 | ILMN_32888 | 1.51 |
| NM_001013031.1 | SORCS1 | ILMN_3669 | 1.51 |
| NM_001040034.1 | CD63 | ILMN_167218 | 1.51 |
| NM_024092.1 | TMEM109 | ILMN_7335 | 1.5 |
| NM_017946.2 | FKBP14 | ILMN_18132 | 1.5 |
| NM_133374.2 | ZNF618 | ILMN_3581 | 1.5 |
| NM_020313.2 | CIAPIN1 | ILMN_43053 | 1.5 |
| NM_006503.2 | PSMC4 | ILMN_4085 | -1.5 |
| NM_001005386.1 | ACTR2 | ILMN_163247 | -1.5 |
| NM_003746.1 | DNCL1 | ILMN_137049 | -1.51 |
| NM_018718.1 | TSGA14 | ILMN_11000 | -1.51 |
| NM_018243.2 | 11-Sep | ILMN_27161 | -1.51 |
| NM_005896.2 | IDH1 | ILMN_14217 | -1.51 |
| NM_014026.3 | DCPS | ILMN_24626 | -1.51 |
| NM_007265.1 | ECD | ILMN_25476 | -1.51 |
| NM_033168.2 | B3GALNT1 | ILMN_29294 | -1.51 |
| NM_006547.2 | IGF2BP3 | ILMN_26370 | -1.52 |
| NM_002629.2 | PGAM1 | ILMN_26357 | -1.52 |
| NM_006117.2 | PECI | ILMN_7427 | -1.52 |
| NM_152773.2 | TCTEX1D2 | ILMN_19950 | -1.53 |
| NM_018151.3 | RIF1 | ILMN_171812 | -1.53 |
| XM_173119.5 | LOC255130 | ILMN_42241 | -1.53 |
| NM_003916.3 | AP1S2 | ILMN_3812 | -1.54 |
| NM_000240.2 | MAOA | ILMN_183788 | -1.54 |
| XM_927868.1 | LOC644774 | ILMN_39269 | -1.54 |
| NM_006644.2 | HSPH1 | ILMN_1157 | -1.55 |
| NM_032594.3 | INSM2 | ILMN_3487 | -1.55 |
| NM_031314.1 | HNRPC | ILMN_24356 | -1.55 |
| NM_016101.3 | NIP7 | ILMN_28300 | -1.55 |
| NM_181702.1 | GEM | ILMN_16170 | -1.55 |
| NM_006854.3 | KDELR2 | ILMN_1810 | -1.56 |
| NM_005953.2 | MT2A | ILMN_11198 | -1.56 |
| NM_020189.4 | ENY2 | ILMN_21796 | -1.57 |
| NM_001024218.1 | GPHN | ILMN_29329 | -1.57 |
| NM_018718.1 | TSGA14 | ILMN_11000 | -1.58 |
| NM_018473.2 | THEM2 | ILMN_27212 | -1.58 |
| NM_016107.3 | ZFR | ILMN_11954 | -1.58 |
| NM_001168.2 | BIRC5 | ILMN_20443 | -1.59 |
| NM_000615.5 | NCAM1 | ILMN_7059 | -1.59 |
| NM_201443.1 | TEAD4 | ILMN_21735 | -1.59 |
| NM_001031725.3 | DDX59 | ILMN_10085 | -1.59 |
| NM_033406.2 | FBXO3 | ILMN_26668 | -1.59 |
| NM_003503.2 | CDC7 | ILMN_20584 | -1.59 |
| NM_022768.4 | RBM15 | ILMN_1342 | -1.6 |
| NM_145247.4 | C10orf78 | ILMN_1251 | -1.6 |
| NM_001012413.1 | SGOL1 | ILMN_14464 | -1.6 |
| NM_212552.2 | BOLA3 | ILMN_28776 | -1.6 |
| NM_177974.1 | CASC4 | ILMN_14927 | -1.6 |
| NM_021971.1 | GMPPB | ILMN_3929 | -1.6 |
| NM_016287.3 | HP1BP3 | ILMN_29502 | -1.6 |
| NM_004093.2 | EFNB2 | ILMN_3827 | -1.6 |
| NM_004615.2 | TSPAN7 | ILMN_20684 | -1.61 |
| NM_001914.2 | CYB5A | ILMN_25182 | -1.61 |
| NM_002525.1 | NRD1 | ILMN_1863 | -1.61 |
| NM_181876.2 | PPP2R2C | ILMN_15268 | -1.61 |
| NM_213596.1 | FOXN4 | ILMN_25618 | -1.61 |
| NM_001017371.3 | SP3 | ILMN_15110 | -1.61 |
| NM_006275.4 | SFRS6 | ILMN_24964 | -1.62 |
| NM_018137.1 | PRMT6 | ILMN_29888 | -1.62 |
| NM_001004322.1 | FLJ38717 | ILMN_13488 | -1.62 |
| NM_001959.3 | EEF1B2 | ILMN_6380 | -1.62 |
| NM_005742.2 | PDIA6 | ILMN_9638 | -1.62 |
| NM_001914.2 | CYB5A | ILMN_25182 | -1.62 |
| NM_017774.2 | CDKAL1 | ILMN_26274 | -1.62 |
| NM_080546.3 | SLC44A1 | ILMN_23525 | -1.62 |
| NM_015523.2 | REXO2 | ILMN_15016 | -1.62 |
| NM_024955.4 | FOXRED2 | ILMN_165686 | -1.63 |
| NM_003689.2 | AKR7A2 | ILMN_182370 | -1.63 |
| NM_138484.2 | SGOL1 | ILMN_14008 | -1.63 |
| NM_006649.2 | UTP14A | ILMN_177989 | -1.63 |
| NM_001018109.1 | PIR | ILMN_13999 | -1.63 |
| NM_001008405.1 | BCAP29 | ILMN_24800 | -1.63 |
| NM_003362.2 | UNG | ILMN_21638 | -1.64 |
| NM_024945.1 | RMI1 | ILMN_11713 | -1.64 |
| XM_001133534.1 | ATP1B3 | ILMN_163124 | -1.64 |
| NM_024296.3 | CCDC28B | ILMN_26263 | -1.64 |
| NM_022770.2 | GINS3 | ILMN_7033 | -1.64 |
| NM_001495.4 | GFRA2 | ILMN_24176 | -1.64 |
| NM_005190.3 | CCNC | ILMN_11667 | -1.64 |
| NM_001009608.1 | C20orf94 | ILMN_24801 | -1.64 |
| NM_006597.3 | HSPA8 | ILMN_181529 | -1.65 |
| NM_001071.1 | TYMS | ILMN_26899 | -1.65 |
| NM_006703.2 | NUDT3 | ILMN_25244 | -1.65 |
| NM_001002800.1 | SMC4 | ILMN_16070 | -1.65 |
| NM_020150.3 | SAR1A | ILMN_17495 | -1.65 |
| NM_016039.1 | C14orf166 | ILMN_14906 | -1.65 |
| NM_013354.5 | CNOT7 | ILMN_7214 | -1.66 |
| XM_937758.1 | LOC148915 | ILMN_30829 | -1.66 |
| NM_172315.1 | MEIS2 | ILMN_17528 | -1.66 |
| NM_001866.2 | COX7B | ILMN_19298 | -1.66 |
| NM_018193.2 | FANCI | ILMN_15143 | -1.66 |
| NM_001037494.1 | DYNLL1 | ILMN_14802 | -1.67 |
| NM_130811.1 | SNAP25 | ILMN_30021 | -1.67 |
| NM_001040139.1 | CKLF | ILMN_162861 | -1.67 |
| NM_024945.2 | RMI1 | ILMN_11713 | -1.67 |
| NM_016286.2 | DCXR | ILMN_17437 | -1.67 |
| NM_017835.1 | C21orf59 | ILMN_12103 | -1.67 |
| NM_024640.3 | YRDC | ILMN_2794 | -1.69 |
| NM_001031717.2 | CRELD1 | ILMN_14216 | -1.69 |
| NM_177966.4 | PDE12 | ILMN_182612 | -1.69 |
| NM_006452.3 | PAICS | ILMN_6032 | -1.7 |
| NM_000943.4 | PPIC | ILMN_19075 | -1.7 |
| NM_012261.2 | C20orf103 | ILMN_165304 | -1.7 |
| NM_001031684.1 | SFRS7 | ILMN_7620 | -1.71 |
| NM_002266.2 | KPNA2 | ILMN_14206 | -1.71 |
| NM_138501.4 | GPSN2 | ILMN_6454 | -1.71 |
| NM_003133.1 | SRP9 | ILMN_137290 | -1.71 |
| NM_032361.1 | THOC3 | ILMN_17969 | -1.72 |
| NM_005589.2 | ALDH6A1 | ILMN_24260 | -1.72 |
| NM_173517.3 | VKORC1L1 | ILMN_9384 | -1.72 |
| NM_003318.3 | TTK | ILMN_24472 | -1.72 |
| NM_005909.3 | MAP1B | ILMN_28251 | -1.74 |
| NM_005327.2 | HADH | ILMN_13258 | -1.74 |
| NM_006515.1 | SETMAR | ILMN_17510 | -1.74 |
| NM_145644.1 | MRPL35 | ILMN_20736 | -1.74 |
| NM_006623.2 | PHGDH | ILMN_5800 | -1.75 |
| NM_022361.3 | POPDC3 | ILMN_162273 | -1.75 |
| XM_001131304.1 | LOC728635 | ILMN_168315 | -1.75 |
| NM_006391.1 | IPO7 | ILMN_28842 | -1.75 |
| NM_003368.4 | USP1 | ILMN_5285 | -1.76 |
| NM_002072.2 | GNAQ | ILMN_18320 | -1.76 |
| NM_181702.1 | GEM | ILMN_16170 | -1.76 |
| NM_012485.1 | HMMR | ILMN_16900 | -1.77 |
| NM_012343.3 | NNT | ILMN_183201 | -1.77 |
| NM_145080.3 | NSMCE1 | ILMN_27090 | -1.77 |
| XM_936354.2 | LOC642197 | ILMN_44406 | -1.77 |
| NM_058216.1 | RAD51C | ILMN_2944 | -1.77 |
| NM_018122.3 | DARS2 | ILMN_183877 | -1.77 |
| NM_002013.2 | FKBP3 | ILMN_7680 | -1.77 |
| NM_014176.2 | UBE2T | ILMN_9573 | -1.78 |
| NM_001040138.1 | CKLF | ILMN_162781 | -1.78 |
| NM_005388.3 | PDCL | ILMN_34020 | -1.78 |
| NM_017887.1 | C1orf123 | ILMN_28661 | -1.79 |
| NM_004111.4 | FEN1 | ILMN_162686 | -1.8 |
| NM_001080415.1 | SR140 | ILMN_169874 | -1.8 |
| NM_004512.3 | IL11RA | ILMN_3536 | -1.8 |
| NM_003258.2 | TK1 | ILMN_30154 | -1.81 |
| NM_017996.2 | DET1 | ILMN_164954 | -1.81 |
| NM_020449.2 | THOC2 | ILMN_162047 | -1.81 |
| NM_000022.2 | ADA | ILMN_8067 | -1.81 |
| NM_013943.1 | CLIC4 | ILMN_3174 | -1.82 |
| NM_024598.2 | C16orf57 | ILMN_11644 | -1.82 |
| NM_018844.2 | BCAP29 | ILMN_24686 | -1.82 |
| NM_000321.2 | RB1 | ILMN_4636 | -1.82 |
| NM_020749.3 | MTUS1 | ILMN_4658 | -1.83 |
| NM_002613.3 | PDPK1 | ILMN_27765 | -1.83 |
| XM_929862.1 | LOC646900 | ILMN_44661 | -1.83 |
| NM_016291.2 | IHPK2 | ILMN_28157 | -1.85 |
| NM_000051.3 | ATM | ILMN_162851 | -1.85 |
| NM_018410.3 | HJURP | ILMN_29337 | -1.85 |
| NM_004219.2 | PTTG1 | ILMN_11067 | -1.85 |
| NM_017518.5 | UCHL5IP | ILMN_27285 | -1.85 |
| NM_018837.2 | SULF2 | ILMN_18271 | -1.85 |
| NM_006807.3 | CBX1 | ILMN_162583 | -1.85 |
| NM_002396.3 | ME2 | ILMN_176679 | -1.85 |
| NM_003404.3 | YWHAB | ILMN_17127 | -1.86 |
| NM_003053.1 | SLC18A1 | ILMN_23324 | -1.86 |
| NM_014736.4 | KIAA0101 | ILMN_2026 | -1.87 |
| NM_144594.1 | GTSF1 | ILMN_17221 | -1.87 |
| NM_006745.3 | SC4MOL | ILMN_2770 | -1.87 |
| NM_006636.3 | MTHFD2 | ILMN_23782 | -1.87 |
| NM_000389.2 | CDKN1A | ILMN_16780 | -1.88 |
| XM_939682.1 | LOC149448 | ILMN_36821 | -1.88 |
| NR_003105.1 | ZWILCH | ILMN_166966 | -1.88 |
| NM_024292.2 | UBL5 | ILMN_14261 | -1.88 |
| NM_001004317.2 | LIN28B | ILMN_16897 | -1.88 |
| NM_001788.4 | 7-Sep | ILMN_25070 | -1.9 |
| NM_000701.6 | ATP1A1 | ILMN_677 | -1.91 |
| NM_015609.2 | C1orf144 | ILMN_5836 | -1.91 |
| NM_001071775.1 | LOC440145 | ILMN_163591 | -1.91 |
| NM_003659.1 | AGPS | ILMN_138634 | -1.91 |
| NM_003107.2 | SOX4 | ILMN_17456 | -1.92 |
| NM_001035505.1 | BOLA3 | ILMN_29223 | -1.92 |
| XM_936354.2 | LOC642197 | ILMN_44406 | -1.92 |
| NM_005921.1 | MAP3K1 | ILMN_309540 | -1.92 |
| NM_000476.1 | AK1 | ILMN_16785 | -1.93 |
| NM_001924.2 | GADD45A | ILMN_17355 | -1.94 |
| NM_001037537.1 | PHYH | ILMN_17762 | -1.95 |
| NM_006860.2 | RABL4 | ILMN_4559 | -1.95 |
| NM_004435.2 | ENDOG | ILMN_26482 | -1.95 |
| NM_017489.1 | TERF1 | ILMN_164297 | -1.95 |
| NR_003144.1 | LOC723972 | ILMN_180363 | -1.95 |
| NM_016098.1 | BRP44L | ILMN_4349 | -1.95 |
| NM_003864.3 | SAP30 | ILMN_31250 | -1.96 |
| NM_058246.3 | DNAJB6 | ILMN_7651 | -1.97 |
| NM_001002246.1 | ANAPC11 | ILMN_5565 | -1.98 |
| NM_017812.2 | CHCHD3 | ILMN_23539 | -1.98 |
| NM_138426.2 | GLCCI1 | ILMN_22544 | -1.98 |
| NM_024122.2 | APOO | ILMN_11248 | -1.99 |
| NM_001274.3 | CHEK1 | ILMN_25327 | -1.99 |
| NM_001967.3 | EIF4A2 | ILMN_5908 | -2 |
| NM_014169.2 | CHMP4A | ILMN_19959 | -2 |
| NM_014264.3 | PLK4 | ILMN_167207 | -2 |
| NM_001540.2 | HSPB1 | ILMN_28967 | -2.01 |
| NM_006429.2 | CCT7 | ILMN_22959 | -2.01 |
| NM_139207.1 | NAP1L1 | ILMN_5405 | -2.01 |
| NM_016323.2 | HERC5 | ILMN_22093 | -2.01 |
| NM_001067.2 | TOP2A | ILMN_19849 | -2.02 |
| NM_006182.2 | DDR2 | ILMN_20698 | -2.02 |
| NM_006337.3 | MCRS1 | ILMN_176605 | -2.02 |
| NM_033117.2 | RBM18 | ILMN_8277 | -2.02 |
| NM_005238.2 | ETS1 | ILMN_173009 | -2.02 |
| NR_002182.1 | NACAP1 | ILMN_14666 | -2.02 |
| NM_001124.1 | ADM | ILMN_29514 | -2.03 |
| NM_003390.2 | WEE1 | ILMN_28737 | -2.03 |
| NM_152227.1 | SNX5 | ILMN_6733 | -2.03 |
| NM_032368.3 | LZIC | ILMN_17648 | -2.03 |
| NM_032776.1 | JMJD1C | ILMN_164120 | -2.03 |
| NM_016644.1 | PRR16 | ILMN_4368 | -2.04 |
| NM_207376.1 | LOC387882 | ILMN_23241 | -2.04 |
| NM_198391.1 | FLRT3 | ILMN_23273 | -2.04 |
| NM_017802.2 | HEATR2 | ILMN_1114 | -2.05 |
| NM_022893.2 | BCL11A | ILMN_17359 | -2.05 |
| NM_017843.3 | BCAS4 | ILMN_21706 | -2.05 |
| NM_032439.1 | PHYHIPL | ILMN_22045 | -2.05 |
| NM_001031713.2 | CCDC90A | ILMN_9159 | -2.06 |
| XM_934410.1 | LOC643995 | ILMN_31166 | -2.06 |
| NM_024333.1 | FSD1 | ILMN_13664 | -2.07 |
| NM_006022.2 | TSC22D1 | ILMN_26720 | -2.07 |
| NM_005983.2 | SKP2 | ILMN_27439 | -2.07 |
| NM_033402.3 | LRRCC1 | ILMN_15234 | -2.07 |
| NM_019071.2 | ING3 | ILMN_177083 | -2.08 |
| NM_006884.2 | SHOX2 | ILMN_40044 | -2.08 |
| NM_176787.4 | PIGN | ILMN_163610 | -2.08 |
| XM_942780.2 | SYNPO2 | ILMN_45907 | -2.08 |
| NM_078629.1 | MSL3L1 | ILMN_29354 | -2.08 |
| NM_013262.3 | MYLIP | ILMN_178445 | -2.08 |
| NM_153682.2 | PIGP | ILMN_18625 | -2.09 |
| XM_001132175.1 | SORD | ILMN_169923 | -2.09 |
| XR_016547.1 | LOC644063 | ILMN_163368 | -2.1 |
| NM_004891.2 | MRPL33 | ILMN_12897 | -2.1 |
| NM_024540.2 | MRPL24 | ILMN_29128 | -2.1 |
| NM_199235.1 | COLEC11 | ILMN_6793 | -2.11 |
| NM_017491.3 | WDR1 | ILMN_14280 | -2.11 |
| NM_206594.1 | ESRRG | ILMN_30032 | -2.11 |
| XM_944429.1 | LOC653994 | ILMN_38337 | -2.12 |
| NM_022652.2 | DUSP6 | ILMN_5926 | -2.12 |
| NM_001333.2 | CTSL2 | ILMN_22377 | -2.12 |
| NM_005318.2 | H1F0 | ILMN_139403 | -2.13 |
| NM_152515.2 | CKAP2L | ILMN_28483 | -2.13 |
| XM_944321.1 | LOC402560 | ILMN_42108 | -2.13 |
| NM_004456.3 | EZH2 | ILMN_25740 | -2.16 |
| NM_003878.1 | GGH | ILMN_9870 | -2.16 |
| NM_001040142.1 | SCN2A | ILMN_167124 | -2.16 |
| NM_001035513.1 | SDHC | ILMN_14364 | -2.18 |
| NM_002598.2 | PDCD2 | ILMN_5469 | -2.18 |
| NR_001449.1 | TRK1 | ILMN_6493 | -2.18 |
| NM_020116.2 | FSTL5 | ILMN_178729 | -2.18 |
| NM_032340.2 | C6orf125 | ILMN_21424 | -2.19 |
| NM_016086.2 | STYXL1 | ILMN_5068 | -2.19 |
| NM_001042370.1 | TROVE2 | ILMN_173505 | -2.2 |
| NM_001382.2 | DPAGT1 | ILMN_10306 | -2.2 |
| XM_930884.1 | LOC653080 | ILMN_32261 | -2.2 |
| NM_205847.1 | GMPPA | ILMN_23338 | -2.2 |
| NM_032907.3 | UBL7 | ILMN_17890 | -2.2 |
| NM_000314.4 | PTEN | ILMN_181706 | -2.21 |
| NM_017917.2 | PPP2R3C | ILMN_23821 | -2.21 |
| NM_006158.2 | NEFL | ILMN_22054 | -2.22 |
| NM_181803.1 | UBE2C | ILMN_6398 | -2.22 |
| NM_130811.1 | SNAP25 | ILMN_30021 | -2.22 |
| NM_016448.1 | DTL | ILMN_29702 | -2.22 |
| NM_001326.2 | CSTF3 | ILMN_27551 | -2.23 |
| NM_001002876.1 | CENPM | ILMN_12351 | -2.23 |
| NM_021009.1 | UBC | ILMN_160470 | -2.24 |
| NM_014241.3 | PTPLA | ILMN_24983 | -2.24 |
| NM_017970.2 | C14orf102 | ILMN_22111 | -2.24 |
| NM_022549.2 | FEZ1 | ILMN_419 | -2.24 |
| NM_003512.3 | HIST1H2AC | ILMN_26493 | -2.24 |
| NM_016625.2 | RSRC1 | ILMN_14978 | -2.24 |
| NM_002014.2 | FKBP4 | ILMN_9429 | -2.24 |
| NM_001017392.2 | SFRS14 | ILMN_17110 | -2.25 |
| NM_005613.3 | RGS4 | ILMN_15378 | -2.27 |
| NM_138809.3 | CMBL | ILMN_1485 | -2.27 |
| NM_054016.1 | FUSIP1 | ILMN_30145 | -2.27 |
| NM_001545.1 | ICT1 | ILMN_11458 | -2.27 |
| NM_002157.1 | HSPE1 | ILMN_2612 | -2.28 |
| NM_030919.2 | FAM83D | ILMN_16948 | -2.29 |
| NM_014739.2 | BCLAF1 | ILMN_163302 | -2.29 |
| NM_000051.3 | ATM | ILMN_162851 | -2.3 |
| NM_020783.2 | SYT4 | ILMN_21875 | -2.3 |
| NM_020195.1 | C14orf124 | ILMN_4144 | -2.3 |
| XM_001126211.1 | LOC727761 | ILMN_162963 | -2.31 |
| NM_181800.1 | UBE2C | ILMN_25999 | -2.32 |
| NM_001500.2 | GMDS | ILMN_16535 | -2.32 |
| NM_012241.2 | SIRT5 | ILMN_18454 | -2.32 |
| NM_002157.1 | HSPE1 | ILMN_2612 | -2.33 |
| NM_138418.2 | C16orf14 | ILMN_9509 | -2.33 |
| NM_182533.1 | C1orf86 | ILMN_2880 | -2.34 |
| NM_005824.1 | LRRC17 | ILMN_162504 | -2.35 |
| NM_022458.3 | LMBR1 | ILMN_1755 | -2.35 |
| NM_000414.1 | HSD17B4 | ILMN_23623 | -2.36 |
| NM_021127.1 | PMAIP1 | ILMN_25637 | -2.36 |
| NM_198047.1 | HIBCH | ILMN_24888 | -2.38 |
| NM_012446.2 | SSBP2 | ILMN_5320 | -2.38 |
| NM_024570.1 | RNASEH2B | ILMN_20578 | -2.38 |
| NM_012215.2 | MGEA5 | ILMN_11399 | -2.39 |
| NM_014142.2 | NUDT5 | ILMN_1656 | -2.39 |
| NM_001003793.1 | RBMS3 | ILMN_16411 | -2.39 |
| NM_181890.1 | UBE2D3 | ILMN_6282 | -2.4 |
| XM_942294.1 | LOC654254 | ILMN_31354 | -2.4 |
| NM_024051.2 | C7orf24 | ILMN_2391 | -2.43 |
| NM_033115.2 | MGC16169 | ILMN_16160 | -2.44 |
| NM_022745.3 | ATPAF1 | ILMN_175478 | -2.44 |
| NM_006712.3 | FASTK | ILMN_11299 | -2.44 |
| NM_024766.2 | C2orf34 | ILMN_14025 | -2.45 |
| NM_001040409.1 | MTHFD2 | ILMN_167475 | -2.46 |
| NM_145697.1 | CDCA1 | ILMN_17725 | -2.47 |
| XM_933956.1 | LOC644162 | ILMN_43225 | -2.48 |
| NM_001017963.2 | HSP90AA1 | ILMN_16669 | -2.49 |
| XM_936240.1 | LOC653884 | ILMN_34094 | -2.5 |
| NM_001634.4 | AMD1 | ILMN_21529 | -2.5 |
| NM_025263.2 | PRR3 | ILMN_21022 | -2.5 |
| NM_030928.2 | CDT1 | ILMN_18895 | -2.5 |
| NM_005192.2 | CDKN3 | ILMN_4098 | -2.51 |
| NM_199189.1 | MATR3 | ILMN_15287 | -2.51 |
| NM_015920.3 | RPS27L | ILMN_28649 | -2.55 |
| NM_005694.1 | COX17 | ILMN_19252 | -2.55 |
| NM_019116.2 | UBFD1 | ILMN_179383 | -2.55 |
| NM_183387.1 | EML5 | ILMN_183722 | -2.56 |
| NM_003053.1 | SLC18A1 | ILMN_23324 | -2.57 |
| NM_005032.3 | PLS3 | ILMN_1428 | -2.61 |
| NM_005348.2 | HSP90AA1 | ILMN_27537 | -2.65 |
| NM_016048.1 | ISOC1 | ILMN_15311 | -2.65 |
| NM_004316.2 | ASCL1 | ILMN_23892 | -2.67 |
| NM_002763.3 | PROX1 | ILMN_177185 | -2.67 |
| NM_002156.4 | HSPD1 | ILMN_178202 | -2.67 |
| NM_003542.3 | HIST1H4C | ILMN_30043 | -2.68 |
| NM_001123.2 | ADK | ILMN_4107 | -2.7 |
| NR_001445.1 | RN7SK | ILMN_14457 | -2.8 |
| XM_001128260.1 | LOC728715 | ILMN_172975 | -2.8 |
| NM_005842.2 | SPRY2 | ILMN_19344 | -2.8 |
| NM_012177.2 | FBXO5 | ILMN_9763 | -2.81 |
| NM_001025248.1 | DUT | ILMN_163345 | -2.81 |
| NM_001018109.1 | PIR | ILMN_13999 | -2.82 |
| NM_002247.2 | KCNMA1 | ILMN_24236 | -2.83 |
| NM_003104.3 | SORD | ILMN_162054 | -2.84 |
| NM_016937.2 | POLA1 | ILMN_181974 | -2.85 |
| NM_020640.2 | DCUN1D1 | ILMN_410 | -2.85 |
| NM_021960.3 | MCL1 | ILMN_18397 | -2.86 |
| NM_182776.1 | MCM7 | ILMN_1133 | -2.87 |
| NM_177983.1 | PPM1G | ILMN_878 | -2.88 |
| NM_016108.2 | AIG1 | ILMN_22004 | -2.88 |
| NM_006449.3 | CDC42EP3 | ILMN_166034 | -2.89 |
| NM_057159.2 | LPAR1 | ILMN_28278 | -2.89 |
| NM_007280.1 | OIP5 | ILMN_18200 | -2.91 |
| NM_017917.2 | PPP2R3C | ILMN_23821 | -2.91 |
| NM_022743.1 | SMYD3 | ILMN_29453 | -2.99 |
| XM_936215.1 | LOC653874 | ILMN_35327 | -3.04 |
| NM_000599.2 | IGFBP5 | ILMN_168089 | -3.05 |
| NM_025129.3 | FUZ | ILMN_24173 | -3.05 |
| NM_005238.2 | ETS1 | ILMN_173009 | -3.08 |
| NM_005916.3 | MCM7 | ILMN_1986 | -3.1 |
| NM_018334.3 | LRRN3 | ILMN_174401 | -3.14 |
| NM_001889.2 | CRYZ | ILMN_30248 | -3.14 |
| NM_001418.3 | EIF4G2 | ILMN_19314 | -3.14 |
| NM_001099660.1 | LRRN3 | ILMN_306943 | -3.26 |
| NM_012342.2 | BAMBI | ILMN_8469 | -3.31 |
| NM_133505.2 | DCN | ILMN_29913 | -3.43 |
| XM_374020.4 | LOC375295 | ILMN_45377 | -3.45 |
| NM_006914.3 | RORB | ILMN_7297 | -3.47 |
| XR_019449.1 | LOC644422 | ILMN_166674 | -3.55 |
| NM_001037126.1 | EXOC4 | ILMN_29454 | -3.61 |
| NM_006366.2 | CAP2 | ILMN_27367 | -3.67 |
| NM_138444.3 | KCTD12 | ILMN_18501 | -3.74 |
| NM_001382.2 | DPAGT1 | ILMN_10306 | -3.82 |
| NM_033001.1 | GTF2I | ILMN_3161 | -3.89 |
| NM_001438.2 | ESRRG | ILMN_29221 | -3.91 |
| NM_000598.4 | IGFBP3 | ILMN_28010 | -3.99 |
| NM_002167.2 | ID3 | ILMN_6829 | -4.58 |
| NM_000599.2 | IGFBP5 | ILMN_168089 | -4.9 |
| NM_002166.4 | ID2 | ILMN_28481 | -5.79 |
| NM_002166.4 | ID2 | ILMN_28481 | -6.57 |
