# Supplementary material for: Minimal Peroxide Exposure of Neuronal Cells Induces Multifaceted Adaptive Responses
Source: PLoS One. 2010 Dec 17;5(12):e14352. doi: 10.1371/journal.pone.0014352 (PMC3003681; doi:10.1371/journal.pone.0014352)
Supplement: Table S6 — MeCh-significantly regulated genes after 2 hours of stimulation in the CMP state SH-SY5Y cells. Each significantly regulated gene is described via its accession number (ACCESSION), Gene Symbol (SYMBOL), Illumina array transcript designation (TRANSCRIPT). For each gene the z-ratio of expression compared to untreated cells after 2 hours of ligand stimulation is displayed (CMP MeCh 2). (1.03 MB DOC) [file pone.0014352.s013.doc]

**Table S6. MeCh-significantly regulated genes after 2 hours of stimulation in the CMP state SH-SY5Y cells**. Each significantly regulated gene is described via its accession number (ACCESSION), Gene Symbol (SYMBOL), Illumina array transcript designation (TRANSCRIPT). For each gene the z-ratio of expression compared to un-treated cells after 2 hours of ligand stimulation is displayed (CMP MeCh 2).

| **ACCESSION** | **SYMBOL** | **TRANSCRIPT** | **CMP MeCh 2** |
| --- | --- | --- | --- |
| NM_001964.2 | EGR1 | ILMN_20932 | 14.66 |
| NM_000584.2 | IL8 | ILMN_179575 | 6.85 |
| NM_005324.3 | H3F3B | ILMN_26885 | 5.7 |
| NM_014817.3 | KIAA0644 | ILMN_164846 | 5.35 |
| XM_944439.2 | LOC653994 | ILMN_38572 | 5.06 |
| NM_012215.2 | MGEA5 | ILMN_11399 | 4.87 |
| NM_001005474.1 | NFKBIZ | ILMN_16362 | 4.84 |
| NM_018697.3 | LANCL2 | ILMN_920 | 4.32 |
| NM_001300.4 | KLF6 | ILMN_17961 | 4.23 |
| NM_001080453.1 | INTS1 | ILMN_173681 | 4.11 |
| XM_001129527.1 | KLF11 | ILMN_168976 | 3.94 |
| NM_182492.1 | LRP5L | ILMN_650 | 3.78 |
| NM_001008490.1 | KLF6 | ILMN_12381 | 3.74 |
| NM_000617.1 | SLC11A2 | ILMN_10129 | 3.74 |
| NM_001033506.1 | CSTF3 | ILMN_27049 | 3.67 |
| NM_002874.3 | RAD23B | ILMN_19346 | 3.66 |
| NM_173042.2 | IL18BP | ILMN_30884 | 3.63 |
| NM_016028.4 | SUV420H1 | ILMN_29861 | 3.58 |
| NM_005841.1 | SPRY1 | ILMN_6281 | 3.55 |
| NM_001008237.1 | TTC32 | ILMN_4829 | 3.55 |
| NM_004417.2 | DUSP1 | ILMN_20700 | 3.51 |
| NM_020801.1 | ARRDC3 | ILMN_22538 | 3.45 |
| NM_148957.2 | TNFRSF19 | ILMN_28684 | 3.42 |
| NM_006988.3 | ADAMTS1 | ILMN_11081 | 3.39 |
| XR_019339.1 | LOC643668 | ILMN_179350 | 3.39 |
| NM_004859.3 | CLTC | ILMN_171089 | 3.38 |
| NM_002673.3 | PLXNB1 | ILMN_22628 | 3.36 |
| NM_002566.4 | P2RY11 | ILMN_12237 | 3.35 |
| XM_925839.1 | LOC158301 | ILMN_38075 | 3.32 |
| NM_033138.2 | CALD1 | ILMN_29896 | 3.27 |
| NM_020724.1 | RNF150 | ILMN_26801 | 3.19 |
| NM_002213.3 | ITGB5 | ILMN_24189 | 3.18 |
| NM_148174.2 | AZIN1 | ILMN_4931 | 3.18 |
| NM_006275.4 | SFRS6 | ILMN_24964 | 3.14 |
| NM_024663.3 | NPEPL1 | ILMN_175218 | 3.14 |
| NM_005627.2 | SGK | ILMN_2451 | 3.14 |
| NM_005385.3 | NKTR | ILMN_23378 | 3.13 |
| NM_152322.2 | BTBD11 | ILMN_506 | 3.12 |
| NM_014614.1 | PSME4 | ILMN_164803 | 3.12 |
| NM_021190.1 | PTBP2 | ILMN_556 | 3.12 |
| NM_033063.1 | MAP6 | ILMN_6882 | 3.1 |
| NM_015306.1 | USP24 | ILMN_309418 | 3.09 |
| NM_014747.2 | RIMS3 | ILMN_21581 | 3.08 |
| NM_018249.4 | CDK5RAP2 | ILMN_9876 | 3.06 |
| NM_015447.1 | CAMSAP1 | ILMN_815 | 3.06 |
| XM_375152.3 | LOC400304 | ILMN_46003 | 3.06 |
| NM_153188.2 | TNPO1 | ILMN_29083 | 3.06 |
| NM_002482.2 | NASP | ILMN_21654 | 3.04 |
| NM_173630.2 | RTTN | ILMN_5471 | 3.01 |
| NM_003749.2 | IRS2 | ILMN_167991 | 2.97 |
| NM_198839.1 | ACACA | ILMN_176065 | 2.97 |
| XM_941665.2 | LOC387763 | ILMN_43061 | 2.96 |
| NM_014014.2 | ASCC3L1 | ILMN_18834 | 2.95 |
| NM_001080485.1 | ZNF275 | ILMN_180340 | 2.94 |
| NM_002923.1 | RGS2 | ILMN_26119 | 2.94 |
| NM_020310.2 | MNT | ILMN_21283 | 2.93 |
| NM_020933.2 | ZNF317 | ILMN_22884 | 2.92 |
| NM_001010915.1 | PTPLAD2 | ILMN_6355 | 2.88 |
| NM_001038702.1 | CDC42SE2 | ILMN_28719 | 2.88 |
| NM_018698.3 | NXT2 | ILMN_168294 | 2.87 |
| NM_005157.3 | ABL1 | ILMN_4033 | 2.85 |
| NM_005238.2 | ETS1 | ILMN_173009 | 2.85 |
| NM_001095.2 | ACCN2 | ILMN_27416 | 2.85 |
| XM_940209.1 | KIAA0194 | ILMN_37512 | 2.84 |
| NM_198836.1 | ACACA | ILMN_9534 | 2.83 |
| NM_001012626.1 | LOC285074 | ILMN_21153 | 2.81 |
| XM_001133842.1 | LOC729446 | ILMN_169307 | 2.8 |
| NM_004075.2 | CRY1 | ILMN_6263 | 2.8 |
| XM_926036.1 | LOC653103 | ILMN_32029 | 2.79 |
| NM_017643.1 | MBTD1 | ILMN_29908 | 2.78 |
| NM_016605.1 | FAM53C | ILMN_11637 | 2.77 |
| NM_014330.2 | PPP1R15A | ILMN_1024 | 2.75 |
| NM_005245.3 | FAT | ILMN_24617 | 2.75 |
| NM_003045.3 | SLC7A1 | ILMN_162673 | 2.75 |
| NM_001077442.1 | HNRNPC | ILMN_165238 | 2.74 |
| NM_005920.2 | MEF2D | ILMN_3465 | 2.74 |
| NM_181054.1 | HIF1A | ILMN_9514 | 2.74 |
| NM_003082.2 | SNAPC1 | ILMN_177713 | 2.72 |
| XM_370865.4 | LOC388122 | ILMN_46143 | 2.71 |
| NM_001006115.2 | IHPK1 | ILMN_8379 | 2.7 |
| NM_015348.1 | TMEM131 | ILMN_308809 | 2.68 |
| NM_032221.3 | CHD6 | ILMN_174095 | 2.66 |
| NM_001013258.1 | ZNF789 | ILMN_11535 | 2.65 |
| NM_024900.3 | PHF17 | ILMN_1535 | 2.64 |
| NM_178831.4 | GATS | ILMN_18755 | 2.64 |
| XM_937850.1 | LOC285176 | ILMN_43277 | 2.64 |
| NM_014380.1 | NGFRAP1 | ILMN_7162 | 2.62 |
| NM_178517.3 | PIGW | ILMN_162681 | 2.62 |
| NM_014382.2 | ATP2C1 | ILMN_16216 | 2.62 |
| NM_004090.2 | DUSP3 | ILMN_180655 | 2.61 |
| NM_015446.3 | AHCTF1 | ILMN_164192 | 2.61 |
| XM_935588.1 | LOC641848 | ILMN_45490 | 2.61 |
| NM_003119.2 | SPG7 | ILMN_26332 | 2.6 |
| NM_001089.1 | ABCA3 | ILMN_18800 | 2.6 |
| XM_934113.1 | LOC653489 | ILMN_42664 | 2.59 |
| NM_018708.2 | FEM1A | ILMN_2838 | 2.58 |
| NM_002915.3 | RFC3 | ILMN_11616 | 2.58 |
| NM_012400.2 | PLA2G2D | ILMN_163941 | 2.57 |
| NM_003086.2 | SNAPC4 | ILMN_180505 | 2.54 |
| NM_016481.3 | C9orf156 | ILMN_12842 | 2.53 |
| NM_152280.2 | SYT11 | ILMN_23967 | 2.52 |
| NM_080702.2 | BAT3 | ILMN_4429 | 2.52 |
| NM_030806.3 | C1orf21 | ILMN_26434 | 2.52 |
| NM_199420.3 | POLQ | ILMN_10389 | 2.51 |
| NM_005334.2 | HCFC1 | ILMN_24237 | 2.5 |
| NM_004560.2 | ROR2 | ILMN_22834 | 2.5 |
| NM_013361.3 | ZNF223 | ILMN_166150 | 2.5 |
| NM_172014.1 | TNFSF14 | ILMN_9666 | 2.48 |
| NM_006372.3 | SYNCRIP | ILMN_28470 | 2.47 |
| NM_004330.1 | BNIP2 | ILMN_9985 | 2.47 |
| NM_004768.2 | SFRS11 | ILMN_4847 | 2.46 |
| NM_015130.2 | TBC1D9 | ILMN_25527 | 2.46 |
| NM_016021.2 | UBE2J1 | ILMN_164177 | 2.45 |
| NM_031942.4 | CDCA7 | ILMN_33249 | 2.44 |
| NM_199043.1 | C14orf102 | ILMN_22442 | 2.44 |
| NM_003906.3 | MCM3AP | ILMN_19614 | 2.43 |
| XM_944915.1 | PTP4A2 | ILMN_137656 | 2.42 |
| NM_001081640.1 | PRKDC | ILMN_180402 | 2.42 |
| NM_025152.1 | NUBPL | ILMN_25397 | 2.42 |
| XR_017492.1 | LOC644330 | ILMN_164787 | 2.42 |
| NM_025074.4 | FRAS1 | ILMN_165073 | 2.41 |
| NM_006925.3 | SFRS5 | ILMN_34497 | 2.4 |
| NM_172249.1 | CSF2RA | ILMN_5061 | 2.4 |
| NM_006045.1 | ATP9A | ILMN_176431 | 2.39 |
| XM_931359.2 | LOC338758 | ILMN_37634 | 2.39 |
| NM_015226.1 | CLEC16A | ILMN_19348 | 2.39 |
| NM_014363.3 | SACS | ILMN_180142 | 2.39 |
| NM_002996.3 | CX3CL1 | ILMN_9636 | 2.38 |
| NM_001924.2 | GADD45A | ILMN_17355 | 2.37 |
| NM_172358.1 | CD46 | ILMN_4413 | 2.37 |
| NM_170721.1 | MSI2 | ILMN_25750 | 2.36 |
| XR_016986.1 | LOC643668 | ILMN_172192 | 2.36 |
| NM_003617.2 | RGS5 | ILMN_167992 | 2.36 |
| NM_024989.3 | PGAP1 | ILMN_173416 | 2.36 |
| NM_003342.4 | UBE2G1 | ILMN_179729 | 2.35 |
| NM_007055.2 | POLR3A | ILMN_1449 | 2.35 |
| NM_005665.4 | EVI5 | ILMN_17996 | 2.35 |
| NM_005921.1 | MAP3K1 | ILMN_309540 | 2.35 |
| NM_006047.4 | RBM12 | ILMN_183773 | 2.35 |
| NM_194301.2 | GARNL1 | ILMN_2823 | 2.34 |
| NM_022308.1 | ICA1 | ILMN_12918 | 2.34 |
| NM_001251.2 | CD68 | ILMN_5188 | 2.32 |
| NM_014935.2 | PLEKHA6 | ILMN_163005 | 2.32 |
| NM_006206.3 | PDGFRA | ILMN_165232 | 2.32 |
| NM_001018115.1 | FANCD2 | ILMN_11844 | 2.32 |
| NM_031469.2 | SH3BGRL2 | ILMN_9801 | 2.32 |
| NM_006925.3 | SFRS5 | ILMN_34497 | 2.31 |
| NM_001621.2 | AHR | ILMN_138365 | 2.31 |
| NM_001018052.1 | POLR3H | ILMN_8571 | 2.31 |
| NM_020822.1 | KCNT1 | ILMN_21599 | 2.31 |
| NM_033419.3 | PERLD1 | ILMN_12215 | 2.31 |
| NM_002048.1 | GAS1 | ILMN_175833 | 2.31 |
| NM_004055.4 | CAPN5 | ILMN_30845 | 2.3 |
| NM_003131.2 | SRF | ILMN_22299 | 2.3 |
| NM_201281.1 | MTMR2 | ILMN_24002 | 2.3 |
| NM_004075.2 | CRY1 | ILMN_6263 | 2.3 |
| NM_001111.3 | ADAR | ILMN_20593 | 2.29 |
| NM_020704.1 | FAM40B | ILMN_18452 | 2.29 |
| NR_000011.1 | SNORA70 | ILMN_7210 | 2.28 |
| NM_021737.1 | CLCN6 | ILMN_6195 | 2.28 |
| XM_001126418.1 | LOC727935 | ILMN_181411 | 2.28 |
| NM_139235.3 | NOL6 | ILMN_7349 | 2.28 |
| NM_001040456.1 | RHBDD2 | ILMN_168345 | 2.28 |
| NM_032440.1 | LCOR | ILMN_173510 | 2.28 |
| NM_002915.3 | RFC3 | ILMN_11616 | 2.28 |
| NM_001008408.3 | RBM33 | ILMN_165407 | 2.27 |
| NM_001029950.1 | DKFZp434K191 | ILMN_28495 | 2.27 |
| NM_182776.1 | MCM7 | ILMN_1133 | 2.27 |
| NM_015352.1 | POFUT1 | ILMN_7876 | 2.27 |
| NM_017822.3 | C12orf41 | ILMN_3399 | 2.27 |
| NM_031372.1 | HNRPDL | ILMN_15196 | 2.26 |
| NM_006516.1 | SLC2A1 | ILMN_421 | 2.26 |
| NM_005520.1 | HNRPH1 | ILMN_4782 | 2.24 |
| NM_198267.1 | ING3 | ILMN_23155 | 2.24 |
| NM_203499.1 | DDX42 | ILMN_1880 | 2.24 |
| NM_080491.1 | GAB2 | ILMN_3317 | 2.24 |
| NM_013412.1 | RABL2A | ILMN_12484 | 2.24 |
| NM_024561.3 | NARG1L | ILMN_22547 | 2.23 |
| NM_014498.2 | GOLPH4 | ILMN_179486 | 2.23 |
| NM_173602.2 | DIP2B | ILMN_179302 | 2.23 |
| NM_016114.3 | ASB1 | ILMN_11707 | 2.23 |
| NM_006159.1 | NELL2 | ILMN_26383 | 2.23 |
| NM_004788.2 | UBE4A | ILMN_175730 | 2.23 |
| NM_198679.1 | RAPGEF1 | ILMN_177243 | 2.22 |
| NM_024612.3 | DHX40 | ILMN_1864 | 2.22 |
| NM_005560.3 | LAMA5 | ILMN_12588 | 2.21 |
| NM_005128.2 | DOPEY2 | ILMN_164626 | 2.21 |
| NM_032239.2 | LARP2 | ILMN_9962 | 2.21 |
| NM_001039705.1 | TRO | ILMN_32618 | 2.2 |
| NM_006958.2 | ZNF16 | ILMN_17198 | 2.2 |
| NM_022748.10 | TNS3 | ILMN_17676 | 2.19 |
| XM_931434.2 | LOC400027 | ILMN_35789 | 2.19 |
| NM_020121.2 | UGCGL2 | ILMN_21506 | 2.19 |
| XM_930178.1 | LOC645018 | ILMN_33646 | 2.19 |
| NM_006749.3 | SLC20A2 | ILMN_29659 | 2.18 |
| NM_078470.2 | COX15 | ILMN_13504 | 2.18 |
| NM_006135.1 | CAPZA1 | ILMN_137637 | 2.18 |
| NM_032776.1 | JMJD1C | ILMN_164120 | 2.17 |
| NM_001924.2 | GADD45A | ILMN_17355 | 2.17 |
| NM_001008485.1 | SLC41A3 | ILMN_19829 | 2.17 |
| NM_003211.3 | TDG | ILMN_29212 | 2.17 |
| NM_144582.2 | TEX261 | ILMN_27405 | 2.16 |
| NM_017821.3 | RHBDL2 | ILMN_20003 | 2.16 |
| NM_152570.1 | LINGO2 | ILMN_24238 | 2.16 |
| NM_003690.3 | PRKRA | ILMN_3524 | 2.15 |
| XR_018848.1 | LOC650369 | ILMN_169499 | 2.15 |
| NM_021807.3 | EXOC4 | ILMN_28890 | 2.15 |
| NM_001387.2 | DPYSL3 | ILMN_23309 | 2.15 |
| NM_000292.1 | PHKA2 | ILMN_20799 | 2.15 |
| NM_014396.3 | VPS41 | ILMN_2386 | 2.13 |
| NM_013291.2 | CPSF1 | ILMN_22094 | 2.13 |
| NM_201440.1 | PPHLN1 | ILMN_4445 | 2.13 |
| NM_005766.2 | FARP1 | ILMN_15608 | 2.13 |
| NM_001008735.1 | HMG1L1 | ILMN_22757 | 2.13 |
| NM_001206.2 | KLF9 | ILMN_169601 | 2.12 |
| NM_001008219.1 | AMY1C | ILMN_28222 | 2.12 |
| NM_004566.2 | PFKFB3 | ILMN_163833 | 2.12 |
| NM_016284.3 | CNOT1 | ILMN_169268 | 2.11 |
| NM_016470.6 | C20orf111 | ILMN_18574 | 2.11 |
| NM_001001132.1 | ITSN1 | ILMN_10040 | 2.09 |
| NM_001013685.1 | LOC401357 | ILMN_29013 | 2.09 |
| NM_025195.2 | TRIB1 | ILMN_29203 | 2.09 |
| NM_004091.2 | E2F2 | ILMN_19730 | 2.08 |
| NM_001412.3 | EIF1AX | ILMN_22164 | 2.08 |
| NM_014641.1 | MDC1 | ILMN_177773 | 2.08 |
| NM_014694.2 | ADAMTSL2 | ILMN_697 | 2.07 |
| NM_176814.3 | ZNF800 | ILMN_163418 | 2.06 |
| NM_014422.2 | PIB5PA | ILMN_8156 | 2.06 |
| XM_928464.1 | LOC146517 | ILMN_32888 | 2.06 |
| NM_006628.4 | ARPP-19 | ILMN_2093 | 2.06 |
| NM_004634.2 | BRPF1 | ILMN_17537 | 2.05 |
| NM_002972.1 | SBF1 | ILMN_22729 | 2.05 |
| NM_199482.1 | PREI3 | ILMN_10571 | 2.05 |
| NM_022781.4 | RNF38 | ILMN_40416 | 2.04 |
| NM_019605.2 | SERTAD4 | ILMN_9918 | 2.03 |
| NM_015878.4 | AZIN1 | ILMN_4825 | 2.03 |
| NM_018097.1 | CEP27 | ILMN_15131 | 2.03 |
| NM_012260.2 | HACL1 | ILMN_180681 | 2.03 |
| NM_003110.4 | SP2 | ILMN_7882 | 2.03 |
| NM_004126.3 | GNG11 | ILMN_8981 | 2.02 |
| NM_003462.3 | DNALI1 | ILMN_24038 | 2.02 |
| NM_015124.2 | GRAMD4 | ILMN_12136 | 2.01 |
| NM_001004322.1 | FLJ38717 | ILMN_13488 | 2.01 |
| NM_001677.3 | ATP1B1 | ILMN_25542 | 2 |
| NM_003966.2 | SEMA5A | ILMN_183828 | 1.99 |
| NM_020808.3 | SIPA1L2 | ILMN_167573 | 1.99 |
| NM_003183.4 | ADAM17 | ILMN_165100 | 1.99 |
| NM_003112.3 | SP4 | ILMN_168713 | 1.99 |
| NM_002650.1 | PIK4CA | ILMN_20581 | 1.97 |
| NM_001029862.1 | ANKRD30B | ILMN_7263 | 1.97 |
| NM_033285.2 | TP53INP1 | ILMN_16203 | 1.97 |
| NM_002166.4 | ID2 | ILMN_28481 | 1.96 |
| NM_014374.1 | REPIN1 | ILMN_1054 | 1.96 |
| NM_022459.3 | XPO4 | ILMN_164187 | 1.96 |
| NM_014634.2 | PPM1F | ILMN_14794 | 1.96 |
| NM_001017980.2 | LOC203547 | ILMN_163926 | 1.96 |
| NM_014947.3 | FOXJ3 | ILMN_26064 | 1.96 |
| NM_031263.1 | HNRPK | ILMN_16515 | 1.96 |
| NM_021190.1 | PTBP2 | ILMN_556 | 1.96 |
| NM_020664.3 | DECR2 | ILMN_7935 | 1.95 |
| NM_003469.3 | SCG2 | ILMN_17827 | 1.95 |
| NM_153044.1 | FLJ35801 | ILMN_23944 | 1.95 |
| NM_003017.3 | SFRS3 | ILMN_29649 | 1.95 |
| NM_020226.3 | PRDM8 | ILMN_22013 | 1.94 |
| NM_004618.3 | TOP3A | ILMN_167915 | 1.94 |
| XM_939697.1 | C9orf130 | ILMN_30981 | 1.94 |
| XM_926231.1 | P704P | ILMN_36679 | 1.94 |
| NM_014988.1 | LIMCH1 | ILMN_3090 | 1.94 |
| NM_183422.1 | TSC22D1 | ILMN_166165 | 1.94 |
| NM_018482.2 | DDEF1 | ILMN_184045 | 1.94 |
| NM_181784.1 | SPRED2 | ILMN_12131 | 1.93 |
| NM_001092.3 | ABR | ILMN_23502 | 1.93 |
| NM_001048201.1 | UHRF1 | ILMN_162952 | 1.93 |
| NM_018416.2 | FOXJ2 | ILMN_165896 | 1.93 |
| XM_001134215.1 | PDPR | ILMN_162295 | 1.93 |
| NM_004641.2 | MLLT10 | ILMN_25545 | 1.93 |
| NM_022489.2 | C14orf173 | ILMN_41230 | 1.92 |
| NM_005462.3 | MAGEC1 | ILMN_173346 | 1.92 |
| NM_014459.2 | PCDH17 | ILMN_5341 | 1.92 |
| NM_015044.3 | GGA2 | ILMN_17168 | 1.92 |
| NM_057159.2 | LPAR1 | ILMN_28278 | 1.92 |
| NM_018325.1 | C9orf72 | ILMN_7216 | 1.91 |
| NM_003713.3 | PPAP2B | ILMN_3538 | 1.91 |
| NM_152424.1 | FLJ39827 | ILMN_19358 | 1.91 |
| NM_021145.2 | DMTF1 | ILMN_16919 | 1.91 |
| NM_206876.1 | PPP1CB | ILMN_22939 | 1.9 |
| NM_178579.1 | PSMF1 | ILMN_2299 | 1.9 |
| NM_004629.1 | FANCG | ILMN_23865 | 1.9 |
| NM_052917.2 | GALNT13 | ILMN_180483 | 1.9 |
| NM_003461.4 | ZYX | ILMN_2137 | 1.9 |
| NM_020824.2 | ARHGAP21 | ILMN_10414 | 1.9 |
| NM_018254.2 | RCOR3 | ILMN_15381 | 1.9 |
| NM_013276.2 | SHPK | ILMN_22706 | 1.89 |
| NM_001357.2 | DHX9 | ILMN_7196 | 1.89 |
| NM_145687.2 | MAP4K4 | ILMN_28871 | 1.89 |
| NM_015902.4 | UBR5 | ILMN_178959 | 1.89 |
| NM_152265.1 | BTF3L4 | ILMN_3105 | 1.88 |
| NM_013262.3 | MYLIP | ILMN_178445 | 1.88 |
| XM_936495.2 | LOC647346 | ILMN_36174 | 1.87 |
| NM_013995.1 | LAMP2 | ILMN_26730 | 1.87 |
| NM_001013703.2 | EIF2AK4 | ILMN_164547 | 1.87 |
| NM_007175.5 | ERLIN2 | ILMN_163672 | 1.87 |
| NM_201559.2 | FOXO3 | ILMN_15525 | 1.86 |
| NM_020177.2 | FEM1C | ILMN_164027 | 1.86 |
| NM_001037533.1 | GON4L | ILMN_14180 | 1.86 |
| NM_014708.3 | KNTC1 | ILMN_25890 | 1.86 |
| NM_002473.3 | MYH9 | ILMN_183555 | 1.86 |
| NM_012256.2 | ZNF212 | ILMN_14026 | 1.86 |
| NM_006447.2 | USP16 | ILMN_29622 | 1.86 |
| XM_497029.2 | LOC441408 | ILMN_31941 | 1.85 |
| NM_198431.1 | HSPA4 | ILMN_25293 | 1.85 |
| NM_030665.3 | RAI1 | ILMN_176671 | 1.85 |
| XM_001127981.1 | LOC728014 | ILMN_169164 | 1.85 |
| NM_014729.2 | TOX | ILMN_16587 | 1.85 |
| NR_002450.1 | SNORD68 | ILMN_25967 | 1.85 |
| NM_002860.3 | ALDH18A1 | ILMN_172838 | 1.85 |
| NM_015534.4 | ZZZ3 | ILMN_14976 | 1.85 |
| NM_199436.1 | SPAST | ILMN_15461 | 1.85 |
| NM_005964.1 | MYH10 | ILMN_23305 | 1.84 |
| NM_001287.3 | CLCN7 | ILMN_8600 | 1.84 |
| NM_021226.2 | ARHGAP22 | ILMN_15801 | 1.84 |
| NM_003496.1 | TRRAP | ILMN_18258 | 1.84 |
| NM_020796.3 | SEMA6A | ILMN_11282 | 1.84 |
| NM_018029.3 | FLJ10213 | ILMN_29061 | 1.84 |
| XM_930995.1 | LOC653086 | ILMN_31021 | 1.83 |
| NM_000046.2 | ARSB | ILMN_180341 | 1.83 |
| NM_001430.3 | EPAS1 | ILMN_26360 | 1.83 |
| NM_015455.3 | CNOT6 | ILMN_17926 | 1.83 |
| NM_001995.2 | ACSL1 | ILMN_12367 | 1.83 |
| NM_001023587.1 | ABCC5 | ILMN_438 | 1.83 |
| NM_178231.1 | ALS2CR14 | ILMN_947 | 1.82 |
| NM_002076.2 | GNS | ILMN_177670 | 1.82 |
| NM_144664.3 | FAM76B | ILMN_22478 | 1.82 |
| NM_001280.1 | CIRBP | ILMN_24327 | 1.81 |
| NM_003565.1 | ULK1 | ILMN_2158 | 1.81 |
| NM_004424.3 | E4F1 | ILMN_23848 | 1.81 |
| NM_017635.3 | SUV420H1 | ILMN_174505 | 1.8 |
| NM_002737.2 | PRKCA | ILMN_24085 | 1.8 |
| NM_130437.2 | DYRK1A | ILMN_18751 | 1.8 |
| NM_001845.4 | COL4A1 | ILMN_24359 | 1.79 |
| XM_001133202.1 | KIAA0363 | ILMN_166209 | 1.79 |
| NM_001014979.1 | LOC90835 | ILMN_8821 | 1.79 |
| NM_005238.2 | ETS1 | ILMN_173009 | 1.79 |
| NM_000787.3 | DBH | ILMN_25962 | 1.79 |
| NM_003580.2 | NSMAF | ILMN_183999 | 1.79 |
| NM_012433.2 | SF3B1 | ILMN_2494 | 1.79 |
| NM_001031827.1 | BOLA2 | ILMN_4509 | 1.78 |
| NM_152470.2 | RNF165 | ILMN_14516 | 1.78 |
| NM_005243.2 | EWSR1 | ILMN_17011 | 1.78 |
| NM_032195.1 | SON | ILMN_8462 | 1.78 |
| NM_001006610.1 | SIAH1 | ILMN_9220 | 1.77 |
| NM_033222.2 | PSIP1 | ILMN_5095 | 1.76 |
| NM_014871.2 | PAN2 | ILMN_661 | 1.76 |
| NM_001356.3 | DDX3X | ILMN_183040 | 1.76 |
| NM_012384.2 | GMEB2 | ILMN_7174 | 1.75 |
| NM_033407.2 | DOCK7 | ILMN_27871 | 1.75 |
| NM_032142.2 | CEP192 | ILMN_7783 | 1.75 |
| XM_926594.2 | LOC642502 | ILMN_31759 | 1.75 |
| NM_032527.3 | ZGPAT | ILMN_23696 | 1.75 |
| NM_001077440.1 | BCLAF1 | ILMN_162019 | 1.75 |
| NM_016343.3 | CENPF | ILMN_176772 | 1.75 |
| NM_007200.3 | AKAP13 | ILMN_28017 | 1.74 |
| NM_013243.2 | SCG3 | ILMN_174345 | 1.74 |
| NM_173073.2 | SLC35C2 | ILMN_14167 | 1.74 |
| NM_003565.1 | ULK1 | ILMN_2158 | 1.74 |
| NM_031954.3 | KCTD10 | ILMN_30217 | 1.74 |
| NM_003463.3 | PTP4A1 | ILMN_165831 | 1.74 |
| NM_194278.3 | C14orf43 | ILMN_166357 | 1.74 |
| NM_025084.1 | FLJ22795 | ILMN_1721 | 1.73 |
| NM_006885.3 | ZFHX3 | ILMN_174159 | 1.73 |
| NM_001319.5 | CSNK1G2 | ILMN_17274 | 1.73 |
| XM_944104.2 | LOC653232 | ILMN_41197 | 1.72 |
| NM_152398.2 | OCIAD2 | ILMN_18246 | 1.72 |
| NM_005255.1 | GAK | ILMN_21151 | 1.72 |
| NM_016577.3 | RAB6B | ILMN_177099 | 1.72 |
| NM_014268.1 | MAPRE2 | ILMN_8637 | 1.72 |
| NM_002938.2 | RNF4 | ILMN_176496 | 1.72 |
| XM_940903.2 | ZC3H5 | ILMN_40646 | 1.71 |
| NM_001567.2 | INPPL1 | ILMN_20903 | 1.71 |
| NM_203401.1 | STMN1 | ILMN_12586 | 1.71 |
| NM_001031617.2 | COX19 | ILMN_15655 | 1.71 |
| NM_020410.1 | ATP13A1 | ILMN_12379 | 1.7 |
| NM_015157.1 | PHLDB1 | ILMN_3997 | 1.7 |
| NM_019106.4 | SEPT3 | ILMN_4065 | 1.7 |
| NM_176811.2 | NLRP8 | ILMN_169055 | 1.7 |
| NM_006241.3 | PPP1R2 | ILMN_165150 | 1.7 |
| XR_017397.1 | LOC644029 | ILMN_163901 | 1.7 |
| NM_015902.4 | UBR5 | ILMN_178959 | 1.7 |
| NM_021145.2 | DMTF1 | ILMN_16919 | 1.7 |
| NM_005654.4 | NR2F1 | ILMN_177945 | 1.7 |
| NM_018116.2 | MSTO1 | ILMN_1073 | 1.69 |
| NM_053279.1 | C8orf13 | ILMN_27702 | 1.69 |
| NM_006773.3 | DDX18 | ILMN_22238 | 1.69 |
| NM_006157.2 | NELL1 | ILMN_2560 | 1.69 |
| NM_012343.3 | NNT | ILMN_183201 | 1.69 |
| NM_001024071.1 | GCH1 | ILMN_14690 | 1.69 |
| NR_003277.1 | LOC728643 | ILMN_183126 | 1.69 |
| NM_002764.2 | PRPS1 | ILMN_161881 | 1.68 |
| NM_017566.2 | KLHDC4 | ILMN_8527 | 1.68 |
| NM_201552.1 | FGL1 | ILMN_30345 | 1.68 |
| NM_033631.2 | LUZP1 | ILMN_2667 | 1.68 |
| NM_001006946.1 | SDC1 | ILMN_169032 | 1.68 |
| NM_000572.2 | IL10 | ILMN_9173 | 1.67 |
| XR_017862.1 | C9orf45 | ILMN_165273 | 1.67 |
| NM_002849.2 | PTPRR | ILMN_178122 | 1.67 |
| NM_018246.2 | CCDC25 | ILMN_5229 | 1.66 |
| NM_018566.3 | YOD1 | ILMN_19081 | 1.66 |
| NM_019119.3 | PCDHB9 | ILMN_23442 | 1.66 |
| XM_937113.2 | LOC647436 | ILMN_44829 | 1.66 |
| NM_006100.2 | ST3GAL6 | ILMN_2870 | 1.66 |
| NM_018976.3 | SLC38A2 | ILMN_10001 | 1.66 |
| NM_017757.2 | ZNF407 | ILMN_12747 | 1.65 |
| NM_032520.3 | GNPTG | ILMN_28173 | 1.65 |
| NM_015113.3 | ZZEF1 | ILMN_11865 | 1.65 |
| NM_006426.1 | DPYSL4 | ILMN_175746 | 1.65 |
| NM_018181.4 | ZNF532 | ILMN_18125 | 1.65 |
| NM_198489.1 | CCDC84 | ILMN_6803 | 1.65 |
| NM_206907.3 | PRKAA1 | ILMN_180991 | 1.65 |
| NM_020215.2 | C14orf132 | ILMN_29055 | 1.65 |
| NM_031844.2 | HNRNPU | ILMN_3074 | 1.64 |
| NM_024909.1 | C6orf134 | ILMN_21139 | 1.64 |
| NM_017896.2 | C20orf11 | ILMN_27220 | 1.64 |
| XM_292963.6 | LOC643997 | ILMN_39721 | 1.64 |
| NR_002201.1 | FTHL3 | ILMN_27691 | 1.63 |
| NR_003239.1 | SNHG11 | ILMN_165269 | 1.63 |
| XM_935802.1 | LOC653829 | ILMN_46774 | 1.63 |
| NM_145701.1 | CDCA4 | ILMN_5601 | 1.63 |
| NM_001407.2 | CELSR3 | ILMN_162782 | 1.63 |
| NM_004093.2 | EFNB2 | ILMN_3827 | 1.63 |
| NM_021009.3 | UBC | ILMN_8850 | 1.62 |
| NR_003659.1 | FAM39DP | ILMN_307683 | 1.62 |
| NM_152341.2 | PAQR4 | ILMN_20105 | 1.62 |
| NM_017991.3 | FLJ10081 | ILMN_469 | 1.62 |
| NM_018184.2 | ARL8B | ILMN_5920 | 1.62 |
| NM_003486.5 | SLC7A5 | ILMN_25446 | 1.61 |
| NM_015560.1 | OPA1 | ILMN_10977 | 1.61 |
| NM_001008393.1 | LOC201725 | ILMN_20795 | 1.61 |
| NM_005667.2 | RNF103 | ILMN_17861 | 1.61 |
| NM_020133.2 | AGPAT4 | ILMN_24920 | 1.61 |
| NM_138477.2 | CDAN1 | ILMN_168162 | 1.6 |
| XM_941155.2 | LOC651894 | ILMN_33374 | 1.6 |
| NM_022457.5 | RFWD2 | ILMN_1221 | 1.6 |
| NM_005688.2 | ABCC5 | ILMN_25223 | 1.6 |
| NM_000274.1 | OAT | ILMN_8426 | 1.6 |
| NM_017707.2 | DDEFL1 | ILMN_25222 | 1.6 |
| NM_006148.1 | LASP1 | ILMN_27039 | 1.6 |
| NM_144578.2 | C14orf32 | ILMN_24999 | 1.6 |
| XM_938988.1 | LOC402221 | ILMN_35678 | 1.59 |
| NM_023080.1 | C8orf33 | ILMN_15901 | 1.59 |
| NM_022487.2 | DCLRE1C | ILMN_28391 | 1.59 |
| NM_001077203.1 | SENP7 | ILMN_167055 | 1.59 |
| NM_004687.3 | MTMR4 | ILMN_163329 | 1.59 |
| NM_015330.1 | SPECC1L | ILMN_168707 | 1.58 |
| NM_006465.2 | ARID3B | ILMN_4032 | 1.58 |
| NM_001013251.1 | SLC3A2 | ILMN_12826 | 1.58 |
| NM_014913.2 | ADNP2 | ILMN_6906 | 1.58 |
| NM_025230.3 | WDR23 | ILMN_171432 | 1.58 |
| XM_927280.1 | LOC644033 | ILMN_39734 | 1.58 |
| NM_020695.3 | REXO1 | ILMN_20923 | 1.58 |
| NM_152641.2 | ARID2 | ILMN_163259 | 1.58 |
| NM_014264.3 | PLK4 | ILMN_167207 | 1.58 |
| NM_080677.1 | DYNLL2 | ILMN_28971 | 1.58 |
| NM_006796.1 | AFG3L2 | ILMN_29564 | 1.58 |
| NM_022805.2 | SNRPN | ILMN_11685 | 1.58 |
| NM_006030.2 | CACNA2D2 | ILMN_28162 | 1.58 |
| NM_033389.2 | SSH2 | ILMN_8279 | 1.58 |
| NM_002959.4 | SORT1 | ILMN_165748 | 1.58 |
| NM_014717.1 | ZNF536 | ILMN_179125 | 1.58 |
| NM_018639.3 | WSB2 | ILMN_162438 | 1.58 |
| NM_020783.2 | SYT4 | ILMN_21875 | 1.58 |
| NM_018150.2 | C1orf164 | ILMN_11157 | 1.57 |
| NR_002323.1 | TUG1 | ILMN_21961 | 1.57 |
| NM_018622.5 | PARL | ILMN_163763 | 1.57 |
| NM_001304.3 | CPD | ILMN_163103 | 1.57 |
| NM_006005.2 | WFS1 | ILMN_18545 | 1.57 |
| NM_014924.3 | KIAA0831 | ILMN_23945 | 1.57 |
| NM_175847.1 | PTBP1 | ILMN_20407 | 1.56 |
| XM_936467.2 | BEXL1 | ILMN_37437 | 1.56 |
| NM_182483.1 | NSFL1C | ILMN_25808 | 1.56 |
| NM_134447.1 | C19orf2 | ILMN_5049 | 1.56 |
| XM_933970.1 | LOC646849 | ILMN_31668 | 1.56 |
| NM_012302.2 | LPHN2 | ILMN_11901 | 1.56 |
| NM_016308.1 | CMPK1 | ILMN_12452 | 1.56 |
| NM_182661.1 | CERK | ILMN_2275 | 1.56 |
| NM_021127.1 | PMAIP1 | ILMN_25637 | 1.55 |
| NM_022459.4 | XPO4 | ILMN_164187 | 1.55 |
| NM_001080.3 | ALDH5A1 | ILMN_1025 | 1.55 |
| NM_015690.2 | STK36 | ILMN_15506 | 1.55 |
| NM_016338.3 | IPO11 | ILMN_20144 | 1.54 |
| NM_005189.1 | CBX2 | ILMN_28525 | 1.54 |
| NM_015338.4 | ASXL1 | ILMN_183479 | 1.54 |
| NM_015655.2 | ZNF337 | ILMN_3280 | 1.54 |
| NM_001013839.1 | EXOC7 | ILMN_25212 | 1.53 |
| NM_182776.1 | MCM7 | ILMN_1133 | 1.53 |
| NM_004036.3 | ADCY3 | ILMN_26929 | 1.53 |
| NM_003418.1 | CNBP | ILMN_9092 | 1.53 |
| NM_004467.3 | FGL1 | ILMN_25289 | 1.53 |
| NM_004598.3 | SPOCK1 | ILMN_25886 | 1.53 |
| XM_942991.2 | LOC642934 | ILMN_39429 | 1.53 |
| NM_145294.4 | WDR90 | ILMN_29490 | 1.53 |
| NM_006447.2 | USP16 | ILMN_29622 | 1.53 |
| NM_006031.4 | PCNT | ILMN_183541 | 1.53 |
| NM_017794.2 | KIAA1797 | ILMN_14720 | 1.53 |
| NM_024706.3 | ZNF668 | ILMN_14968 | 1.53 |
| NM_198334.1 | GANAB | ILMN_29263 | 1.53 |
| XM_941195.2 | LOC388621 | ILMN_42661 | 1.52 |
| NM_014395.1 | DAPP1 | ILMN_24094 | 1.52 |
| NM_015516.3 | TSKU | ILMN_29523 | 1.52 |
| NM_014746.2 | RNF144 | ILMN_15740 | 1.52 |
| NM_005342.2 | HMGB3 | ILMN_8326 | 1.52 |
| NM_004939.1 | DDX1 | ILMN_2242 | 1.52 |
| NM_005443.4 | PAPSS1 | ILMN_171260 | 1.52 |
| NM_001018160.1 | NAE1 | ILMN_19127 | 1.52 |
| NM_002293.2 | LAMC1 | ILMN_182622 | 1.51 |
| NM_020159.2 | SMARCAD1 | ILMN_3339 | 1.51 |
| NM_001010864.1 | LOC196752 | ILMN_1138 | 1.51 |
| NM_014000.2 | VCL | ILMN_27566 | 1.51 |
| NM_018335.2 | C14orf131 | ILMN_8908 | 1.51 |
| NM_006997.2 | TACC2 | ILMN_16130 | 1.51 |
| NM_001040455.1 | SIDT2 | ILMN_166785 | 1.51 |
| NM_014141.4 | CNTNAP2 | ILMN_176606 | 1.51 |
| NM_022662.2 | ANAPC1 | ILMN_164277 | 1.51 |
| NM_178496.2 | C3orf59 | ILMN_14619 | 1.51 |
| NM_004671.2 | PIAS2 | ILMN_11308 | 1.51 |
| NM_017943.2 | FBXO34 | ILMN_172741 | 1.51 |
| NM_003583.2 | DYRK2 | ILMN_3688 | 1.51 |
| NM_016542.3 | MST4 | ILMN_27038 | 1.51 |
| NM_178014.2 | TUBB | ILMN_23399 | 1.5 |
| XM_945430.1 | SSR2 | ILMN_138339 | 1.5 |
| NM_007005.3 | TLE4 | ILMN_14046 | 1.5 |
| NM_014838.2 | ZBED4 | ILMN_8641 | 1.5 |
| NM_006445.3 | PRPF8 | ILMN_19289 | 1.5 |
| NM_015906.3 | TRIM33 | ILMN_4131 | 1.5 |
| XM_044178.10 | KIAA1211 | ILMN_38895 | 1.5 |
| NM_014167.2 | CCDC59 | ILMN_12564 | -1.5 |
| XM_001130192.1 | KIAA1160 | ILMN_162086 | -1.5 |
| NM_021222.1 | PRUNE | ILMN_27601 | -1.5 |
| NM_032361.1 | THOC3 | ILMN_17969 | -1.51 |
| NM_032574.2 | DPY30 | ILMN_18534 | -1.51 |
| NM_006310.2 | NPEPPS | ILMN_184074 | -1.51 |
| NM_032638.3 | GATA2 | ILMN_20021 | -1.52 |
| NM_012289.3 | KEAP1 | ILMN_18799 | -1.52 |
| XM_926382.2 | LOC642755 | ILMN_32084 | -1.52 |
| NM_133646.2 | ZAK | ILMN_5666 | -1.52 |
| NM_153713.1 | LIX1L | ILMN_3572 | -1.52 |
| NM_144635.3 | FAM131A | ILMN_2542 | -1.52 |
| NM_032421.1 | CYLN2 | ILMN_14847 | -1.52 |
| NM_020899.2 | ZBTB4 | ILMN_5011 | -1.52 |
| XR_019071.1 | LOC642333 | ILMN_183964 | -1.53 |
| NM_001009994.1 | C6orf159 | ILMN_13118 | -1.53 |
| NM_004175.3 | SNRPD3 | ILMN_163179 | -1.53 |
| NM_201627.1 | TRIM41 | ILMN_24501 | -1.53 |
| NM_080603.3 | ZSWIM1 | ILMN_631 | -1.53 |
| NM_002528.4 | NTHL1 | ILMN_15981 | -1.53 |
| NM_032747.2 | USMG5 | ILMN_10409 | -1.53 |
| NM_014254.1 | TMEM5 | ILMN_26271 | -1.53 |
| NM_002751.5 | MAPK11 | ILMN_23755 | -1.54 |
| NM_032285.2 | MGC3207 | ILMN_3158 | -1.54 |
| NM_153273.3 | IHPK1 | ILMN_1661 | -1.54 |
| XR_016056.1 | LOC729843 | ILMN_166982 | -1.54 |
| NM_023937.2 | MRPL34 | ILMN_5839 | -1.54 |
| NM_001042588.1 | SNUPN | ILMN_178280 | -1.54 |
| NM_000913.3 | OPRL1 | ILMN_6491 | -1.54 |
| NM_003083.2 | SNAPC2 | ILMN_14587 | -1.54 |
| NM_001035505.1 | BOLA3 | ILMN_29223 | -1.55 |
| NM_016941.2 | DLL3 | ILMN_21363 | -1.55 |
| NM_174942.1 | GAS2L3 | ILMN_5609 | -1.56 |
| NM_001031713.2 | CCDC90A | ILMN_9159 | -1.56 |
| NM_024775.9 | GEMIN6 | ILMN_23187 | -1.56 |
| NM_001535.2 | PRMT2 | ILMN_10737 | -1.56 |
| NM_001042426.1 | CENPA | ILMN_180589 | -1.56 |
| NM_001039199.1 | C20orf121 | ILMN_20894 | -1.56 |
| NM_032250.1 | ANKRD20A1 | ILMN_29242 | -1.56 |
| NM_001042426.1 | CENPA | ILMN_180589 | -1.57 |
| NM_080821.2 | C20orf108 | ILMN_25852 | -1.57 |
| NM_017567.2 | NAGK | ILMN_4544 | -1.57 |
| NM_177999.1 | ASB6 | ILMN_13316 | -1.57 |
| NM_203284.1 | RBPJ | ILMN_170184 | -1.57 |
| NM_001007794.1 | CEPT1 | ILMN_15134 | -1.57 |
| NM_017812.2 | CHCHD3 | ILMN_23539 | -1.57 |
| NM_182523.1 | C3orf68 | ILMN_4406 | -1.58 |
| NM_006442.2 | DRAP1 | ILMN_9412 | -1.58 |
| NM_007083.3 | NUDT6 | ILMN_903 | -1.58 |
| NM_032448.1 | FAM120B | ILMN_10767 | -1.58 |
| XM_001129423.1 | LOC729137 | ILMN_166772 | -1.58 |
| NM_006876.1 | B3GNT6 | ILMN_16433 | -1.58 |
| NM_058216.1 | RAD51C | ILMN_2944 | -1.58 |
| NM_020449.2 | THOC2 | ILMN_162047 | -1.58 |
| NM_014260.2 | PFDN6 | ILMN_8046 | -1.59 |
| NM_001031677.2 | RAB24 | ILMN_25731 | -1.59 |
| NM_031905.2 | ARMC10 | ILMN_171553 | -1.59 |
| NM_001539.2 | DNAJA1 | ILMN_5819 | -1.59 |
| NM_024769.2 | ASAM | ILMN_27333 | -1.59 |
| NM_199487.1 | UQCC | ILMN_16175 | -1.59 |
| NM_005830.2 | MRPS31 | ILMN_6293 | -1.59 |
| NM_053067.1 | UBQLN1 | ILMN_9768 | -1.6 |
| NM_203390.2 | RBM12B | ILMN_174962 | -1.6 |
| NM_138458.2 | WDR92 | ILMN_37809 | -1.6 |
| XM_934743.2 | FBXO46 | ILMN_45822 | -1.6 |
| NM_016071.2 | MRPS33 | ILMN_4243 | -1.6 |
| NM_030628.1 | INTS5 | ILMN_12254 | -1.6 |
| NM_212552.2 | BOLA3 | ILMN_28776 | -1.6 |
| NM_018403.4 | DCP1A | ILMN_27256 | -1.6 |
| NM_198401.2 | ANKRD46 | ILMN_9031 | -1.6 |
| NM_014661.3 | FAM53B | ILMN_26314 | -1.6 |
| NM_018584.5 | CAMK2N1 | ILMN_22077 | -1.61 |
| NM_020189.4 | ENY2 | ILMN_21796 | -1.61 |
| NM_018332.3 | DDX19A | ILMN_10415 | -1.61 |
| NM_181708.1 | BCDIN3D | ILMN_18065 | -1.61 |
| NM_001013690.1 | LOC401720 | ILMN_21595 | -1.61 |
| NM_018056.1 | TMEM39B | ILMN_1056 | -1.62 |
| NM_004089.3 | TSC22D3 | ILMN_9893 | -1.62 |
| NM_032356.3 | LSMD1 | ILMN_25444 | -1.62 |
| NR_002225.2 | RPS26L | ILMN_27668 | -1.63 |
| NM_020701.1 | ISY1 | ILMN_17522 | -1.63 |
| NM_031902.3 | MRPS5 | ILMN_14369 | -1.63 |
| NM_005056.1 | JARID1A | ILMN_12150 | -1.63 |
| NM_014793.3 | LCMT2 | ILMN_183463 | -1.63 |
| NM_005905.3 | SMAD9 | ILMN_28187 | -1.64 |
| NM_014184.2 | CNIH4 | ILMN_9903 | -1.64 |
| NM_005402.2 | RALA | ILMN_164730 | -1.64 |
| NM_024710.1 | ISOC2 | ILMN_27084 | -1.64 |
| NM_018838.3 | NDUFA12 | ILMN_26981 | -1.65 |
| NM_001037494.1 | DYNLL1 | ILMN_14802 | -1.65 |
| NM_025205.3 | MED28 | ILMN_14574 | -1.65 |
| NM_194326.2 | RPS19BP1 | ILMN_8107 | -1.65 |
| NM_138794.2 | LYPLAL1 | ILMN_25005 | -1.66 |
| NM_018044.2 | NSUN5 | ILMN_895 | -1.66 |
| NM_015523.2 | REXO2 | ILMN_15016 | -1.66 |
| NM_152362.1 | TNFAIP8L1 | ILMN_3344 | -1.66 |
| NM_001031711.1 | ERGIC1 | ILMN_7272 | -1.67 |
| NM_006736.5 | DNAJB2 | ILMN_34421 | -1.67 |
| NM_020918.3 | GPAM | ILMN_174762 | -1.67 |
| NM_005380.4 | NBL1 | ILMN_21944 | -1.67 |
| NM_030917.2 | FIP1L1 | ILMN_6961 | -1.67 |
| NM_016071.2 | MRPS33 | ILMN_4243 | -1.67 |
| NM_001040668.1 | BCL2L12 | ILMN_177176 | -1.67 |
| XM_938667.1 | DEAF1 | ILMN_138757 | -1.67 |
| NM_001039847.1 | GPX4 | ILMN_33147 | -1.68 |
| NM_005740.2 | DNAL4 | ILMN_22246 | -1.68 |
| NM_032323.1 | TMEM79 | ILMN_13555 | -1.68 |
| NM_017588.2 | WDR5 | ILMN_27310 | -1.68 |
| NM_006281.2 | STK3 | ILMN_26935 | -1.68 |
| NM_001077498.1 | C17orf63 | ILMN_173887 | -1.68 |
| NM_004697.3 | PRPF4 | ILMN_22066 | -1.69 |
| NM_012289.3 | KEAP1 | ILMN_18799 | -1.69 |
| NM_015379.3 | BRI3 | ILMN_27636 | -1.69 |
| NM_003211.3 | TDG | ILMN_29212 | -1.69 |
| NM_013299.3 | SAC3D1 | ILMN_9385 | -1.69 |
| NM_173680.3 | ZNF775 | ILMN_179169 | -1.69 |
| XM_939954.2 | LOC388789 | ILMN_39285 | -1.69 |
| NM_015994.2 | ATP6V1D | ILMN_26737 | -1.69 |
| NM_021971.1 | GMPPB | ILMN_3929 | -1.69 |
| XM_944321.1 | LOC402560 | ILMN_42108 | -1.69 |
| XM_001125680.1 | LOC730432 | ILMN_165880 | -1.69 |
| NM_001008405.1 | BCAP29 | ILMN_24800 | -1.69 |
| NM_014847.2 | UBAP2L | ILMN_163836 | -1.7 |
| NM_207368.3 | LOC348262 | ILMN_643 | -1.7 |
| NM_178863.2 | KCTD13 | ILMN_18783 | -1.7 |
| NM_021244.3 | RRAGD | ILMN_5663 | -1.7 |
| NM_079837.2 | BANP | ILMN_8638 | -1.7 |
| NM_004792.2 | PPIG | ILMN_24595 | -1.7 |
| NM_014487.3 | ZNF330 | ILMN_6878 | -1.7 |
| NM_030808.3 | NDEL1 | ILMN_20362 | -1.7 |
| NM_001097599.1 | TMEM22 | ILMN_306942 | -1.7 |
| NM_001001795.1 | MGC70857 | ILMN_23947 | -1.71 |
| NM_006158.2 | NEFL | ILMN_22054 | -1.71 |
| NM_024516.2 | C16orf53 | ILMN_20272 | -1.71 |
| NM_138807.2 | C3orf31 | ILMN_9705 | -1.71 |
| NM_183425.1 | RBM38 | ILMN_20092 | -1.71 |
| NM_016297.2 | PCYOX1 | ILMN_15130 | -1.72 |
| NM_080605.3 | B3GALT6 | ILMN_170784 | -1.72 |
| NM_004422.2 | DVL2 | ILMN_29320 | -1.72 |
| NM_014800.9 | ELMO1 | ILMN_33356 | -1.72 |
| NM_001042631.1 | LOC644096 | ILMN_170495 | -1.72 |
| NM_152274.2 | FAM58A | ILMN_3352 | -1.72 |
| NM_004544.2 | NDUFA10 | ILMN_7463 | -1.72 |
| NM_030980.1 | ISG20L2 | ILMN_7800 | -1.73 |
| NM_000398.4 | CYB5R3 | ILMN_16364 | -1.73 |
| NM_002045.2 | GAP43 | ILMN_28511 | -1.73 |
| NM_005192.2 | CDKN3 | ILMN_4098 | -1.73 |
| NM_080632.1 | UPF3B | ILMN_174905 | -1.73 |
| NM_003344.2 | UBE2H | ILMN_163352 | -1.74 |
| NM_002712.1 | PPP1R7 | ILMN_29559 | -1.74 |
| NM_014046.2 | MRPS18B | ILMN_8749 | -1.74 |
| NM_020418.2 | PCBP4 | ILMN_10036 | -1.74 |
| NM_199044.2 | NSUN4 | ILMN_23916 | -1.74 |
| NM_001040056.1 | MAPK3 | ILMN_177323 | -1.75 |
| NM_031287.2 | SF3B5 | ILMN_20062 | -1.75 |
| NM_017693.2 | BIVM | ILMN_181297 | -1.75 |
| NM_015017.3 | USP33 | ILMN_176756 | -1.75 |
| NM_017953.2 | C1orf181 | ILMN_20839 | -1.75 |
| NM_018991.2 | STAG3L1 | ILMN_28376 | -1.75 |
| NM_001042549.1 | NSL1 | ILMN_164300 | -1.75 |
| NM_182533.1 | C1orf86 | ILMN_2880 | -1.75 |
| NM_138792.2 | LEO1 | ILMN_14593 | -1.75 |
| NM_005744.2 | ARIH1 | ILMN_16556 | -1.76 |
| NM_004615.2 | TSPAN7 | ILMN_20684 | -1.76 |
| NM_058246.3 | DNAJB6 | ILMN_7651 | -1.76 |
| XM_930344.2 | LOC644934 | ILMN_43758 | -1.77 |
| NR_002166.1 | SEDLP | ILMN_1258 | -1.77 |
| NM_030771.1 | CCDC34 | ILMN_2645 | -1.77 |
| NM_173475.1 | DCUN1D3 | ILMN_182838 | -1.77 |
| NM_032479.2 | MRPL36 | ILMN_22209 | -1.77 |
| NM_001012756.1 | ZNF260 | ILMN_172733 | -1.77 |
| NM_007342.1 | NUPL2 | ILMN_2154 | -1.77 |
| NM_198291.1 | SRC | ILMN_15256 | -1.77 |
| NM_017974.3 | ATG16L1 | ILMN_25151 | -1.77 |
| NM_022914.2 | ACD | ILMN_5907 | -1.77 |
| XM_928247.1 | LOC441528 | ILMN_30629 | -1.78 |
| NM_019037.2 | EXOSC4 | ILMN_25178 | -1.78 |
| NM_003358.1 | UGCG | ILMN_26228 | -1.78 |
| NM_138444.3 | KCTD12 | ILMN_18501 | -1.78 |
| NM_053067.1 | UBQLN1 | ILMN_9768 | -1.78 |
| NM_006233.4 | POLR2I | ILMN_17223 | -1.79 |
| NM_178448.2 | C9orf140 | ILMN_24887 | -1.79 |
| NM_080723.3 | NRSN1 | ILMN_178353 | -1.79 |
| NM_205847.1 | GMPPA | ILMN_23338 | -1.79 |
| NM_199235.1 | COLEC11 | ILMN_6793 | -1.8 |
| NM_004865.2 | TBPL1 | ILMN_3787 | -1.8 |
| NM_004592.2 | SFRS8 | ILMN_9797 | -1.8 |
| NM_032772.3 | ZNF503 | ILMN_2048 | -1.81 |
| NM_005707.1 | PDCD7 | ILMN_179659 | -1.81 |
| NM_002095.4 | GTF2E2 | ILMN_4316 | -1.81 |
| NM_004879.3 | EI24 | ILMN_8791 | -1.81 |
| NM_016535.3 | ZNF581 | ILMN_13004 | -1.82 |
| XM_925818.1 | LOC642282 | ILMN_41968 | -1.83 |
| NM_001013406.1 | KRIT1 | ILMN_15411 | -1.84 |
| NM_016463.5 | CXXC5 | ILMN_166144 | -1.85 |
| NM_024067.2 | C7orf26 | ILMN_9701 | -1.85 |
| NM_001077268.1 | ZFYVE19 | ILMN_175347 | -1.85 |
| NM_013300.1 | C12orf24 | ILMN_24807 | -1.85 |
| NM_014347.1 | ZNF324 | ILMN_29920 | -1.85 |
| NM_015314.2 | KIAA0895 | ILMN_28455 | -1.85 |
| NM_017816.1 | LYAR | ILMN_23200 | -1.85 |
| XM_930579.2 | LOC653820 | ILMN_40990 | -1.85 |
| NM_001002755.1 | NFU1 | ILMN_9748 | -1.86 |
| NM_003528.2 | HIST2H2BE | ILMN_28293 | -1.86 |
| NM_152240.1 | ZMAT3 | ILMN_9027 | -1.86 |
| NM_018275.3 | C7orf43 | ILMN_1100 | -1.86 |
| NM_007277.4 | EXOC3 | ILMN_6110 | -1.87 |
| NM_012453.2 | TBL2 | ILMN_25753 | -1.87 |
| NM_138484.2 | SGOL1 | ILMN_14008 | -1.87 |
| NM_002717.2 | PPP2R2A | ILMN_24841 | -1.87 |
| NM_032320.5 | BTBD10 | ILMN_30066 | -1.87 |
| NM_014498.2 | GOLPH4 | ILMN_179486 | -1.87 |
| NM_007280.1 | OIP5 | ILMN_18200 | -1.88 |
| NM_054014.1 | FKBP1A | ILMN_29213 | -1.88 |
| NM_022075.3 | LASS2 | ILMN_10647 | -1.88 |
| NM_000819.3 | GART | ILMN_22974 | -1.89 |
| NM_178314.2 | RILPL1 | ILMN_1609 | -1.89 |
| NM_005836.2 | HRSP12 | ILMN_8062 | -1.89 |
| NM_001007157.1 | PHF14 | ILMN_2096 | -1.89 |
| NM_016010.1 | C8orf70 | ILMN_13979 | -1.9 |
| XM_496446.3 | LOC440737 | ILMN_39347 | -1.9 |
| XM_927071.2 | LOC643790 | ILMN_38875 | -1.9 |
| NM_014458.3 | KLHL20 | ILMN_11595 | -1.91 |
| NM_001042370.1 | TROVE2 | ILMN_173505 | -1.91 |
| NM_001752.2 | CAT | ILMN_13962 | -1.91 |
| NM_005926.2 | MFAP1 | ILMN_20656 | -1.91 |
| NM_004643.1 | PABPN1 | ILMN_29660 | -1.92 |
| NM_005022.2 | PFN1 | ILMN_2354 | -1.92 |
| NM_006874.2 | ELF2 | ILMN_1532 | -1.92 |
| NM_170662.3 | CBLB | ILMN_18286 | -1.93 |
| NM_002918.3 | RFX1 | ILMN_178968 | -1.93 |
| NM_180976.1 | PPP2R5D | ILMN_2366 | -1.93 |
| NM_003729.2 | RTCD1 | ILMN_11697 | -1.93 |
| NM_198434.1 | AURKA | ILMN_12352 | -1.93 |
| NM_031434.2 | TMUB1 | ILMN_11025 | -1.93 |
| NR_003144.1 | LOC723972 | ILMN_180363 | -1.93 |
| NM_016166.1 | PIAS1 | ILMN_16806 | -1.93 |
| NM_001008566.1 | TPST2 | ILMN_13248 | -1.93 |
| NM_005513.1 | GTF2E1 | ILMN_175401 | -1.93 |
| NM_022740.2 | HIPK2 | ILMN_29690 | -1.94 |
| NM_002086.3 | GRB2 | ILMN_173749 | -1.94 |
| NM_001002860.2 | BTBD7 | ILMN_178877 | -1.94 |
| NM_024063.1 | SPATA5L1 | ILMN_4249 | -1.94 |
| NM_152379.2 | C1orf131 | ILMN_9839 | -1.95 |
| NM_213720.1 | C22orf16 | ILMN_25503 | -1.95 |
| NM_001487.1 | BLOC1S1 | ILMN_14526 | -1.95 |
| NM_024297.2 | PHF23 | ILMN_20271 | -1.95 |
| NM_139159.3 | DPP9 | ILMN_26244 | -1.96 |
| NM_153018.2 | ZFP3 | ILMN_42182 | -1.96 |
| NM_002263.2 | KIFC1 | ILMN_8595 | -1.97 |
| NM_152912.3 | MTIF3 | ILMN_16655 | -1.97 |
| NM_012143.2 | TFIP11 | ILMN_3675 | -1.97 |
| NM_015542.2 | UPF2 | ILMN_21163 | -1.97 |
| NM_183399.1 | RNF14 | ILMN_7292 | -1.97 |
| NM_025115.1 | C8orf41 | ILMN_19175 | -1.98 |
| NM_032138.3 | KBTBD7 | ILMN_181309 | -1.98 |
| NM_033064.3 | ATCAY | ILMN_27014 | -1.99 |
| NM_000945.3 | PPP3R1 | ILMN_26308 | -1.99 |
| NM_006597.3 | HSPA8 | ILMN_181529 | -1.99 |
| NM_032344.1 | NUDT22 | ILMN_19793 | -2 |
| NM_000076.1 | CDKN1C | ILMN_20689 | -2 |
| NM_153201.1 | HSPA8 | ILMN_14829 | -2 |
| NM_015957.1 | APIP | ILMN_15379 | -2 |
| NM_130442.2 | ELMO1 | ILMN_33821 | -2 |
| NM_001012413.1 | SGOL1 | ILMN_14464 | -2.01 |
| NM_001029.3 | RPS26 | ILMN_1912 | -2.02 |
| NM_178507.2 | OAF | ILMN_12751 | -2.02 |
| NM_002949.2 | MRPL12 | ILMN_25815 | -2.03 |
| NM_138798.1 | MITD1 | ILMN_27516 | -2.03 |
| NM_173510.1 | CCDC117 | ILMN_21814 | -2.05 |
| NM_005694.1 | COX17 | ILMN_19252 | -2.06 |
| NM_177983.1 | PPM1G | ILMN_878 | -2.06 |
| NM_017946.2 | FKBP14 | ILMN_18132 | -2.06 |
| NM_017895.6 | DDX27 | ILMN_20732 | -2.06 |
| NM_001042401.1 | C21orf51 | ILMN_179828 | -2.07 |
| NM_002335.1 | LRP5 | ILMN_19887 | -2.07 |
| NM_006602.2 | TCFL5 | ILMN_12278 | -2.07 |
| NM_004378.1 | CRABP1 | ILMN_12739 | -2.08 |
| NM_005749.2 | TOB1 | ILMN_13735 | -2.08 |
| NM_001039703.1 | NBPF10 | ILMN_45673 | -2.08 |
| NM_201280.1 | MUTED | ILMN_21576 | -2.09 |
| NM_025049.2 | PIF1 | ILMN_7325 | -2.09 |
| NM_014117.2 | C16orf72 | ILMN_4283 | -2.09 |
| NM_012117.1 | CBX5 | ILMN_25072 | -2.09 |
| NM_032673.2 | PCGF1 | ILMN_5720 | -2.1 |
| NM_138720.1 | HIST1H2BD | ILMN_17622 | -2.1 |
| NM_198391.1 | FLRT3 | ILMN_23273 | -2.1 |
| NM_012433.2 | SF3B1 | ILMN_168075 | -2.1 |
| NM_004359.1 | CDC34 | ILMN_9900 | -2.11 |
| NM_004901.2 | ENTPD4 | ILMN_19012 | -2.11 |
| NM_006704.2 | SUGT1 | ILMN_26229 | -2.11 |
| NM_015942.3 | MTERFD1 | ILMN_24756 | -2.11 |
| NM_201434.1 | RAB5C | ILMN_176672 | -2.12 |
| NM_017909.1 | RMND1 | ILMN_29019 | -2.12 |
| NM_015456.2 | COBRA1 | ILMN_5079 | -2.12 |
| XM_930694.1 | LOC642477 | ILMN_36253 | -2.12 |
| NM_003512.3 | HIST1H2AC | ILMN_26493 | -2.12 |
| NM_003475.2 | RASSF7 | ILMN_12457 | -2.12 |
| NM_017940.2 | NBPF1 | ILMN_163270 | -2.13 |
| NM_198088.1 | ZNF200 | ILMN_18094 | -2.13 |
| NM_020851.1 | ISLR2 | ILMN_19345 | -2.13 |
| NM_001078651.1 | TMEM134 | ILMN_176754 | -2.13 |
| NM_032439.1 | PHYHIPL | ILMN_22045 | -2.13 |
| NM_024095.3 | ASB8 | ILMN_165486 | -2.14 |
| NM_017612.2 | ZCCHC8 | ILMN_30318 | -2.14 |
| NM_001552.2 | IGFBP4 | ILMN_9309 | -2.14 |
| NM_057089.2 | AP1S1 | ILMN_4691 | -2.15 |
| NM_001040167.1 | LFNG | ILMN_163755 | -2.15 |
| NM_032334.1 | C8orf53 | ILMN_24637 | -2.16 |
| NM_001039141.1 | TRIOBP | ILMN_34620 | -2.16 |
| NM_024071.2 | ZFYVE21 | ILMN_1317 | -2.17 |
| NM_001283.2 | AP1S1 | ILMN_21653 | -2.17 |
| NM_001918.2 | DBT | ILMN_169961 | -2.18 |
| XM_934985.1 | LOC400879 | ILMN_31001 | -2.18 |
| NM_015634.2 | KIAA1279 | ILMN_8497 | -2.18 |
| NM_001007157.1 | PHF14 | ILMN_2096 | -2.18 |
| NM_007155.4 | ZP3 | ILMN_17555 | -2.19 |
| NM_024011.2 | CDC2L2 | ILMN_20434 | -2.19 |
| NM_138418.2 | C16orf14 | ILMN_9509 | -2.2 |
| NM_138720.1 | HIST1H2BD | ILMN_17622 | -2.21 |
| NM_000628.3 | IL10RB | ILMN_26097 | -2.22 |
| NM_020230.4 | PPAN | ILMN_25948 | -2.22 |
| NM_001042353.1 | FAM110A | ILMN_170512 | -2.22 |
| NM_001827.1 | CKS2 | ILMN_14702 | -2.22 |
| NM_001033503.1 | SAR1B | ILMN_16595 | -2.22 |
| NM_016176.2 | SDF4 | ILMN_768 | -2.23 |
| NM_012110.2 | CHIC2 | ILMN_24345 | -2.23 |
| NM_001031677.2 | RAB24 | ILMN_25731 | -2.23 |
| XM_944786.1 | LOC650737 | ILMN_40280 | -2.24 |
| NM_005873.2 | RGS19 | ILMN_42727 | -2.24 |
| NM_054014.1 | FKBP1A | ILMN_29213 | -2.24 |
| NM_199287.2 | CCDC137 | ILMN_309720 | -2.25 |
| NM_005274.1 | GNG5 | ILMN_21191 | -2.25 |
| NM_016042.2 | EXOSC3 | ILMN_174330 | -2.25 |
| NM_148178.1 | C9orf23 | ILMN_3926 | -2.25 |
| NM_001080501.1 | MGC3196 | ILMN_181711 | -2.25 |
| NM_017958.1 | PLEKHB2 | ILMN_29704 | -2.26 |
| NM_018983.3 | NOLA1 | ILMN_14204 | -2.26 |
| NM_014494.2 | TNRC6A | ILMN_3173 | -2.26 |
| NR_001449.1 | TRK1 | ILMN_6493 | -2.27 |
| NM_030805.2 | LMAN2L | ILMN_1985 | -2.27 |
| NM_001048197.1 | SNHG3-RCC1 | ILMN_167397 | -2.28 |
| NM_003077.2 | SMARCD2 | ILMN_14227 | -2.28 |
| NM_018464.2 | CISD1 | ILMN_4843 | -2.29 |
| NM_057089.2 | AP1S1 | ILMN_4691 | -2.29 |
| NM_199126.1 | ZNF585A | ILMN_9003 | -2.29 |
| XM_933956.1 | LOC644162 | ILMN_43225 | -2.29 |
| NM_020147.2 | THAP10 | ILMN_182683 | -2.29 |
| NM_201414.1 | APP | ILMN_23272 | -2.29 |
| NM_013388.4 | PREB | ILMN_6913 | -2.3 |
| NM_024057.2 | NUP37 | ILMN_4147 | -2.3 |
| NM_018847.2 | KLHL9 | ILMN_20376 | -2.31 |
| NM_145074.2 | HTRA2 | ILMN_12587 | -2.31 |
| NM_145647.2 | WDR67 | ILMN_20846 | -2.32 |
| NM_032280.1 | ZCCHC9 | ILMN_25119 | -2.32 |
| NM_202468.1 | GIPC1 | ILMN_21354 | -2.32 |
| NM_015449.2 | C1orf43 | ILMN_933 | -2.34 |
| NM_005713.1 | COL4A3BP | ILMN_10635 | -2.34 |
| NM_024095.3 | ASB8 | ILMN_165486 | -2.35 |
| NM_022087.2 | GALNT11 | ILMN_5237 | -2.35 |
| NM_207346.2 | TSEN54 | ILMN_8569 | -2.35 |
| NM_025058.3 | TRIM46 | ILMN_18492 | -2.35 |
| NM_170783.1 | ZNRD1 | ILMN_1419 | -2.36 |
| NM_006182.2 | DDR2 | ILMN_20698 | -2.36 |
| NM_005652.2 | TERF2 | ILMN_21134 | -2.36 |
| NM_138797.1 | ANKRD54 | ILMN_21813 | -2.37 |
| NM_182919.1 | TICAM1 | ILMN_11434 | -2.38 |
| NM_032728.2 | PPAPDC3 | ILMN_25638 | -2.38 |
| NM_012210.3 | TRIM32 | ILMN_14426 | -2.38 |
| NM_020153.2 | C11orf60 | ILMN_171038 | -2.39 |
| NM_006455.2 | SC65 | ILMN_21605 | -2.39 |
| NM_004365.2 | CETN3 | ILMN_25663 | -2.4 |
| NM_006717.2 | SPIN1 | ILMN_23742 | -2.4 |
| NM_005573.2 | LMNB1 | ILMN_4100 | -2.42 |
| NM_032346.1 | PDCD2L | ILMN_25365 | -2.43 |
| NM_012170.2 | FBXO22 | ILMN_5718 | -2.43 |
| NM_001040708.1 | HEY1 | ILMN_164416 | -2.43 |
| NM_001896.2 | CSNK2A2 | ILMN_16798 | -2.44 |
| NM_002613.3 | PDPK1 | ILMN_27765 | -2.44 |
| NM_012241.2 | SIRT5 | ILMN_18454 | -2.45 |
| NM_001009608.1 | C20orf94 | ILMN_24801 | -2.45 |
| XM_001132711.1 | RFNG | ILMN_168322 | -2.46 |
| NM_018473.2 | THEM2 | ILMN_27212 | -2.47 |
| NM_012475.4 | USP21 | ILMN_18019 | -2.47 |
| NM_006761.3 | YWHAE | ILMN_18524 | -2.47 |
| NM_001827.1 | CKS2 | ILMN_14702 | -2.48 |
| XM_935818.1 | FLJ20397 | ILMN_137080 | -2.48 |
| NM_013328.2 | PYCR2 | ILMN_18209 | -2.52 |
| NM_012133.2 | COPG2 | ILMN_23766 | -2.53 |
| NM_001466.2 | FZD2 | ILMN_12499 | -2.54 |
| NM_012475.4 | USP21 | ILMN_18019 | -2.54 |
| NM_006191.2 | PA2G4 | ILMN_28541 | -2.55 |
| NM_001033026.1 | C19orf6 | ILMN_167551 | -2.55 |
| NM_019116.2 | UBFD1 | ILMN_179383 | -2.56 |
| NM_017816.1 | LYAR | ILMN_23200 | -2.56 |
| NM_014941.1 | MORC2 | ILMN_12502 | -2.56 |
| NM_002067.1 | GNA11 | ILMN_25749 | -2.56 |
| NM_022830.1 | TUT1 | ILMN_6523 | -2.58 |
| NM_148973.1 | TNFRSF25 | ILMN_14916 | -2.59 |
| NM_019058.2 | DDIT4 | ILMN_13176 | -2.61 |
| NM_001007230.1 | SPOP | ILMN_12838 | -2.62 |
| NM_020234.4 | DTWD1 | ILMN_3248 | -2.62 |
| NM_016618.1 | KRCC1 | ILMN_25337 | -2.62 |
| NM_153333.2 | TCEAL8 | ILMN_12551 | -2.63 |
| NM_198970.1 | AES | ILMN_25198 | -2.63 |
| NM_018164.1 | C12orf11 | ILMN_14707 | -2.63 |
| NM_005692.3 | ABCF2 | ILMN_14116 | -2.64 |
| NM_018390.2 | PLCXD1 | ILMN_8273 | -2.64 |
| NM_138316.2 | PANK1 | ILMN_406 | -2.64 |
| NM_004428.2 | EFNA1 | ILMN_14320 | -2.65 |
| NM_000856.3 | GUCY1A3 | ILMN_11680 | -2.65 |
| XM_930284.1 | LOC441763 | ILMN_36192 | -2.66 |
| NM_002824.4 | PTMS | ILMN_12889 | -2.69 |
| NM_145899.1 | HMGA1 | ILMN_5105 | -2.71 |
| NM_018064.2 | C6orf166 | ILMN_1311 | -2.72 |
| NM_020799.2 | STAMBPL1 | ILMN_1387 | -2.75 |
| XM_941876.1 | BRI3BP | ILMN_139088 | -2.76 |
| NM_033091.1 | TRIM4 | ILMN_8530 | -2.76 |
| NM_012460.2 | TIMM9 | ILMN_9968 | -2.77 |
| NM_001037163.1 | MGC12966 | ILMN_182436 | -2.77 |
| NM_181702.1 | GEM | ILMN_16170 | -2.78 |
| NM_006391.1 | IPO7 | ILMN_28842 | -2.78 |
| NM_207350.1 | MGC72104 | ILMN_26269 | -2.79 |
| NM_004503.3 | HOXC6 | ILMN_15669 | -2.79 |
| NM_001014286.2 | FAM48A | ILMN_1616 | -2.79 |
| NM_001394.5 | DUSP4 | ILMN_17730 | -2.8 |
| NM_178439.3 | GMCL1 | ILMN_3285 | -2.8 |
| NM_079837.2 | BANP | ILMN_8638 | -2.82 |
| NM_016561.1 | BFAR | ILMN_23440 | -2.84 |
| NM_003221.3 | TFAP2B | ILMN_164377 | -2.85 |
| NM_170783.1 | ZNRD1 | ILMN_1419 | -2.86 |
| NM_201414.1 | APP | ILMN_23272 | -2.87 |
| NM_001007278.1 | TRIM13 | ILMN_14225 | -2.88 |
| XM_926249.2 | LOC642852 | ILMN_40586 | -2.88 |
| NM_014620.4 | HOXC4 | ILMN_16005 | -2.9 |
| NM_024321.3 | RBM42 | ILMN_182570 | -2.92 |
| NM_007369.2 | GPR161 | ILMN_22837 | -2.92 |
| NM_001634.4 | AMD1 | ILMN_21529 | -2.92 |
| NM_006265.1 | RAD21 | ILMN_171453 | -2.93 |
| NM_005681.2 | TAF1A | ILMN_8114 | -2.94 |
| NM_014322.2 | OPN3 | ILMN_166169 | -2.97 |
| NM_001039937.1 | INTS6 | ILMN_38649 | -2.97 |
| NM_178439.3 | GMCL1 | ILMN_3285 | -2.97 |
| NM_020892.1 | DTX2 | ILMN_21612 | -2.98 |
| NM_001365.2 | DLG4 | ILMN_164548 | -3 |
| NM_007198.2 | PROSC | ILMN_23472 | -3.03 |
| NM_004982.2 | KCNJ8 | ILMN_29993 | -3.03 |
| NM_001037675.1 | NBPF20 | ILMN_26956 | -3.04 |
| NM_001085363.1 | MEX3D | ILMN_307190 | -3.06 |
| NM_016277.3 | RAB23 | ILMN_177407 | -3.08 |
| NM_003021.3 | SGTA | ILMN_1162 | -3.09 |
| NM_001012643.2 | LOC339344 | ILMN_6535 | -3.1 |
| NM_006630.1 | ZNF234 | ILMN_29233 | -3.1 |
| XM_498571.2 | LOC440160 | ILMN_33035 | -3.12 |
| NM_007167.2 | ZMYM6 | ILMN_1275 | -3.12 |
| NM_022893.2 | BCL11A | ILMN_17359 | -3.14 |
| NM_006860.2 | RABL4 | ILMN_4559 | -3.17 |
| NM_006630.1 | ZNF234 | ILMN_29233 | -3.21 |
| NM_005413.1 | SIX3 | ILMN_26476 | -3.22 |
| NM_181702.1 | GEM | ILMN_16170 | -3.23 |
| NM_016374.5 | ARID4B | ILMN_162934 | -3.25 |
| XM_926112.2 | LOC441155 | ILMN_37470 | -3.27 |
| NM_002897.3 | RBMS1 | ILMN_18726 | -3.29 |
| NM_015942.3 | MTERFD1 | ILMN_174209 | -3.31 |
| NM_173618.1 | CCDC95 | ILMN_3654 | -3.37 |
| NM_024585.2 | ARMC7 | ILMN_163623 | -3.43 |
| NM_014596.4 | ZNRD1 | ILMN_20009 | -3.47 |
| NM_020749.3 | MTUS1 | ILMN_4658 | -3.51 |
| NM_006145.1 | DNAJB1 | ILMN_19740 | -3.64 |
| NM_004316.2 | ASCL1 | ILMN_23892 | -3.66 |
| NM_020705.1 | TBC1D24 | ILMN_34755 | -3.66 |
| NM_184234.1 | RBM39 | ILMN_20330 | -3.7 |
| NM_078629.1 | MSL3L1 | ILMN_29354 | -3.94 |
| NM_016042.2 | EXOSC3 | ILMN_174330 | -4.08 |
| NM_016374.5 | ARID4B | ILMN_162934 | -4.66 |
| NM_004456.3 | EZH2 | ILMN_25740 | -4.68 |
| NM_005345.4 | HSPA1A | ILMN_6623 | -5.22 |
| NM_005346.3 | HSPA1B | ILMN_25549 | -7.4 |
