# Supplementary material for: Minimal Peroxide Exposure of Neuronal Cells Induces Multifaceted Adaptive Responses
Source: PLoS One. 2010 Dec 17;5(12):e14352. doi: 10.1371/journal.pone.0014352 (PMC3003681; doi:10.1371/journal.pone.0014352)
Supplement: Table S7 — MeCh-significantly regulated genes after 4 hours of stimulation in the CMP state SH-SY5Y cells. Each significantly regulated gene is described via its accession number (ACCESSION), Gene Symbol (SYMBOL), Illumina array transcript designation (TRANSCRIPT). For each gene the z-ratio of expression compared to untreated cells after 4 hours of ligand stimulation is displayed (CMP MeCh 4). (1.20 MB DOC) [file pone.0014352.s014.doc]

**Table S7. MeCh-significantly regulated genes after 4 hours of stimulation in the CMP state SH-SY5Y cells**. Each significantly regulated gene is described via its accession number (ACCESSION), Gene Symbol (SYMBOL), Illumina array transcript designation (TRANSCRIPT). For each gene the z-ratio of expression compared to un-treated cells after 4 hours of ligand stimulation is displayed (CMP MeCh 4).

| **ACCESSION** | **SYMBOL** | **TRANSCRIPT** | **CMP MeCh 4** |
| --- | --- | --- | --- |
| NM_000584.2 | IL8 | ILMN_179575 | 9.97 |
| NM_005324.3 | H3F3B | ILMN_26885 | 5.87 |
| XM_944439.2 | LOC653994 | ILMN_38572 | 5.3 |
| NM_148957.2 | TNFRSF19 | ILMN_28684 | 4.99 |
| NM_016028.4 | SUV420H1 | ILMN_29861 | 4.54 |
| NM_014817.3 | KIAA0644 | ILMN_164846 | 4.44 |
| NM_000617.1 | SLC11A2 | ILMN_10129 | 4.4 |
| NM_012215.2 | MGEA5 | ILMN_11399 | 4.36 |
| NM_001033506.1 | CSTF3 | ILMN_27049 | 4.35 |
| NM_153188.2 | TNPO1 | ILMN_29083 | 4.2 |
| NM_018697.3 | LANCL2 | ILMN_920 | 3.98 |
| NM_152322.2 | BTBD11 | ILMN_506 | 3.84 |
| NM_005245.3 | FAT | ILMN_24617 | 3.69 |
| NM_001001391.1 | CD44 | ILMN_10947 | 3.67 |
| NM_024663.3 | NPEPL1 | ILMN_175218 | 3.66 |
| NM_015447.1 | CAMSAP1 | ILMN_815 | 3.59 |
| NM_033138.2 | CALD1 | ILMN_29896 | 3.56 |
| NM_020724.1 | RNF150 | ILMN_26801 | 3.55 |
| NM_182492.1 | LRP5L | ILMN_650 | 3.53 |
| NM_024629.2 | MLF1IP | ILMN_16700 | 3.48 |
| NM_002673.3 | PLXNB1 | ILMN_22628 | 3.43 |
| NM_080491.1 | GAB2 | ILMN_3317 | 3.38 |
| NM_001024646.1 | CLK1 | ILMN_27286 | 3.36 |
| NM_018948.2 | ERRFI1 | ILMN_4328 | 3.31 |
| NM_001080485.1 | ZNF275 | ILMN_180340 | 3.28 |
| NM_001040456.1 | RHBDD2 | ILMN_168345 | 3.28 |
| NM_002874.3 | RAD23B | ILMN_19346 | 3.26 |
| NM_003086.2 | SNAPC4 | ILMN_180505 | 3.26 |
| NM_020796.3 | SEMA6A | ILMN_11282 | 3.21 |
| NM_001080453.1 | INTS1 | ILMN_173681 | 3.17 |
| NM_001006115.2 | IHPK1 | ILMN_8379 | 3.16 |
| NM_002160.2 | TNC | ILMN_14948 | 3.15 |
| NM_001010915.1 | PTPLAD2 | ILMN_6355 | 3.15 |
| NM_004075.2 | CRY1 | ILMN_6263 | 3.15 |
| NM_003972.2 | BTAF1 | ILMN_8616 | 3.14 |
| XM_001126418.1 | LOC727935 | ILMN_181411 | 3.12 |
| NM_001013258.1 | ZNF789 | ILMN_11535 | 3.11 |
| NM_032199.1 | ARID5B | ILMN_165822 | 3.11 |
| NM_001031623.2 | ZNF451 | ILMN_990 | 3.1 |
| NM_172014.1 | TNFSF14 | ILMN_9666 | 3.09 |
| NM_002566.4 | P2RY11 | ILMN_12237 | 3.07 |
| NM_004071.2 | CLK1 | ILMN_162592 | 3.06 |
| NM_173042.2 | IL18BP | ILMN_30884 | 3 |
| NM_002213.3 | ITGB5 | ILMN_24189 | 3 |
| NM_001008408.3 | RBM33 | ILMN_165407 | 2.99 |
| NM_022748.10 | TNS3 | ILMN_17676 | 2.98 |
| NM_019591.2 | ZNF26 | ILMN_3233 | 2.97 |
| NM_017643.1 | MBTD1 | ILMN_29908 | 2.96 |
| NM_006275.4 | SFRS6 | ILMN_24964 | 2.96 |
| NM_017635.3 | SUV420H1 | ILMN_174505 | 2.95 |
| NM_001012626.1 | LOC285074 | ILMN_21153 | 2.95 |
| NM_001206.2 | KLF9 | ILMN_169601 | 2.94 |
| NM_015446.3 | AHCTF1 | ILMN_164192 | 2.9 |
| NM_014498.2 | GOLPH4 | ILMN_179486 | 2.89 |
| NM_004906.3 | WTAP | ILMN_24202 | 2.87 |
| XM_375152.3 | LOC400304 | ILMN_46003 | 2.87 |
| NM_014747.2 | RIMS3 | ILMN_21581 | 2.85 |
| NM_001077188.1 | HS6ST2 | ILMN_182242 | 2.84 |
| NM_005385.3 | NKTR | ILMN_23378 | 2.83 |
| NM_015906.3 | TRIM33 | ILMN_4131 | 2.81 |
| NM_152470.2 | RNF165 | ILMN_14516 | 2.77 |
| NM_203459.1 | CAMSAP1L1 | ILMN_14735 | 2.77 |
| NM_024612.3 | DHX40 | ILMN_1864 | 2.77 |
| NM_014014.2 | ASCC3L1 | ILMN_18834 | 2.75 |
| NM_002482.2 | NASP | ILMN_21654 | 2.74 |
| NM_014000.2 | VCL | ILMN_27566 | 2.71 |
| NM_001621.2 | AHR | ILMN_138365 | 2.69 |
| NM_012463.2 | ATP6V0A2 | ILMN_23163 | 2.68 |
| NM_022781.4 | RNF38 | ILMN_40416 | 2.68 |
| NM_007005.3 | TLE4 | ILMN_14046 | 2.68 |
| NM_032239.2 | LARP2 | ILMN_9962 | 2.67 |
| NM_001987.4 | ETV6 | ILMN_175744 | 2.67 |
| NM_017821.3 | RHBDL2 | ILMN_20003 | 2.67 |
| NM_030806.3 | C1orf21 | ILMN_26434 | 2.66 |
| XM_001134215.1 | PDPR | ILMN_162295 | 2.63 |
| NM_018416.2 | FOXJ2 | ILMN_165896 | 2.62 |
| XM_925839.1 | LOC158301 | ILMN_38075 | 2.62 |
| NM_201555.1 | FHL2 | ILMN_21541 | 2.61 |
| NM_005667.2 | RNF103 | ILMN_17861 | 2.61 |
| NM_003045.3 | SLC7A1 | ILMN_162673 | 2.61 |
| NM_016481.3 | C9orf156 | ILMN_12842 | 2.6 |
| NM_021190.1 | PTBP2 | ILMN_556 | 2.6 |
| XM_928464.1 | LOC146517 | ILMN_32888 | 2.59 |
| NM_003461.4 | ZYX | ILMN_2137 | 2.59 |
| NM_178231.1 | ALS2CR14 | ILMN_947 | 2.58 |
| NM_012256.2 | ZNF212 | ILMN_14026 | 2.58 |
| NM_033020.2 | TRIM33 | ILMN_3792 | 2.57 |
| NM_002210.2 | ITGAV | ILMN_182431 | 2.57 |
| NM_152280.2 | SYT11 | ILMN_23967 | 2.57 |
| NM_001013685.1 | LOC401357 | ILMN_29013 | 2.56 |
| NM_002473.3 | MYH9 | ILMN_183555 | 2.56 |
| NM_006372.3 | SYNCRIP | ILMN_28470 | 2.55 |
| NM_017644.3 | KLHL24 | ILMN_26914 | 2.55 |
| NM_013450.2 | BAZ2B | ILMN_20026 | 2.55 |
| NM_025152.1 | NUBPL | ILMN_25397 | 2.55 |
| NM_012400.2 | PLA2G2D | ILMN_163941 | 2.53 |
| NM_018254.2 | RCOR3 | ILMN_15381 | 2.53 |
| NM_148174.2 | AZIN1 | ILMN_4931 | 2.52 |
| NM_003119.2 | SPG7 | ILMN_26332 | 2.52 |
| NM_014717.1 | ZNF536 | ILMN_179125 | 2.51 |
| NM_001300.4 | KLF6 | ILMN_17961 | 2.51 |
| NM_178831.4 | GATS | ILMN_18755 | 2.51 |
| NM_198267.1 | ING3 | ILMN_23155 | 2.49 |
| NM_006465.2 | ARID3B | ILMN_4032 | 2.49 |
| NM_014363.3 | SACS | ILMN_180142 | 2.47 |
| NM_020808.3 | SIPA1L2 | ILMN_167573 | 2.47 |
| NM_018097.1 | CEP27 | ILMN_15131 | 2.47 |
| NM_004330.1 | BNIP2 | ILMN_9985 | 2.47 |
| NM_024909.1 | C6orf134 | ILMN_21139 | 2.46 |
| NM_001038702.1 | CDC42SE2 | ILMN_28719 | 2.46 |
| NM_031469.2 | SH3BGRL2 | ILMN_9801 | 2.46 |
| NM_032918.1 | RERG | ILMN_12434 | 2.45 |
| XM_944915.1 | PTP4A2 | ILMN_137656 | 2.44 |
| XM_001134215.1 | PDPR | ILMN_162295 | 2.44 |
| NM_018566.3 | YOD1 | ILMN_19081 | 2.43 |
| NM_004090.2 | DUSP3 | ILMN_180655 | 2.43 |
| NM_015130.2 | TBC1D9 | ILMN_25527 | 2.42 |
| NM_001387.2 | DPYSL3 | ILMN_23309 | 2.42 |
| NM_016114.3 | ASB1 | ILMN_11707 | 2.41 |
| NM_005157.3 | ABL1 | ILMN_4033 | 2.41 |
| NM_172249.1 | CSF2RA | ILMN_5061 | 2.41 |
| NM_014717.1 | ZNF536 | ILMN_179125 | 2.4 |
| NM_013361.3 | ZNF223 | ILMN_166150 | 2.4 |
| NM_020704.1 | FAM40B | ILMN_18452 | 2.39 |
| NM_002604.1 | PDE7A | ILMN_3430 | 2.38 |
| NM_001031712.2 | TRMT11 | ILMN_8801 | 2.38 |
| NM_003082.2 | SNAPC1 | ILMN_177713 | 2.37 |
| NM_001008237.1 | TTC32 | ILMN_4829 | 2.37 |
| NM_172358.1 | CD46 | ILMN_4413 | 2.37 |
| NM_177952.1 | PPM1A | ILMN_10552 | 2.36 |
| NM_002915.3 | RFC3 | ILMN_11616 | 2.36 |
| NM_005342.2 | HMGB3 | ILMN_8326 | 2.36 |
| NM_031263.1 | HNRPK | ILMN_16515 | 2.36 |
| NM_199043.1 | C14orf102 | ILMN_22442 | 2.35 |
| NM_005128.2 | DOPEY2 | ILMN_164626 | 2.35 |
| NM_006241.3 | PPP1R2 | ILMN_165150 | 2.33 |
| NM_015455.3 | CNOT6 | ILMN_17926 | 2.33 |
| NM_021090.3 | MTMR3 | ILMN_27578 | 2.33 |
| XM_940209.1 | KIAA0194 | ILMN_37512 | 2.33 |
| NM_014614.1 | PSME4 | ILMN_164803 | 2.33 |
| NM_004075.2 | CRY1 | ILMN_6263 | 2.31 |
| NM_006045.1 | ATP9A | ILMN_176431 | 2.3 |
| NM_022450.2 | RHBDF1 | ILMN_20892 | 2.29 |
| NM_015560.1 | OPA1 | ILMN_10977 | 2.29 |
| NM_153451.2 | ORAOV1 | ILMN_5733 | 2.29 |
| NM_024735.2 | FBXO31 | ILMN_17806 | 2.29 |
| NM_015878.4 | AZIN1 | ILMN_4825 | 2.29 |
| NM_032440.1 | LCOR | ILMN_173510 | 2.28 |
| NM_001040456.1 | RHBDD2 | ILMN_168345 | 2.28 |
| NM_152424.1 | FLJ39827 | ILMN_19358 | 2.27 |
| XM_938742.1 | SGPP2 | ILMN_139087 | 2.27 |
| NM_016021.2 | UBE2J1 | ILMN_164177 | 2.27 |
| XM_926036.1 | LOC653103 | ILMN_32029 | 2.27 |
| NM_020940.2 | KIAA1600 | ILMN_8082 | 2.27 |
| NM_001077442.1 | HNRNPC | ILMN_165238 | 2.27 |
| NM_002915.3 | RFC3 | ILMN_11616 | 2.27 |
| NM_013276.2 | SHPK | ILMN_22706 | 2.26 |
| NM_033285.2 | TP53INP1 | ILMN_16203 | 2.26 |
| XM_497182.3 | LOC644670 | ILMN_38310 | 2.26 |
| NM_025133.3 | FBXO11 | ILMN_18553 | 2.26 |
| XM_931359.2 | LOC338758 | ILMN_37634 | 2.25 |
| NM_201557.2 | FHL2 | ILMN_42988 | 2.25 |
| NM_005334.2 | HCFC1 | ILMN_24237 | 2.25 |
| NM_003622.2 | PPFIBP1 | ILMN_172147 | 2.24 |
| NM_001251.2 | CD68 | ILMN_5188 | 2.24 |
| NM_000292.1 | PHKA2 | ILMN_20799 | 2.24 |
| NM_014330.2 | PPP1R15A | ILMN_1024 | 2.24 |
| NM_002076.2 | GNS | ILMN_177670 | 2.24 |
| NM_005194.2 | CEBPB | ILMN_4674 | 2.23 |
| NM_014947.3 | FOXJ3 | ILMN_26064 | 2.23 |
| NM_005189.1 | CBX2 | ILMN_28525 | 2.23 |
| NM_144664.3 | FAM76B | ILMN_22478 | 2.22 |
| NM_001083585.1 | RABEP1 | ILMN_307418 | 2.21 |
| NM_181782.2 | NCOA7 | ILMN_9525 | 2.21 |
| NM_152594.1 | SPRED1 | ILMN_1375 | 2.21 |
| NM_002938.2 | RNF4 | ILMN_176496 | 2.2 |
| NM_130437.2 | DYRK1A | ILMN_18751 | 2.2 |
| NM_004859.3 | CLTC | ILMN_171089 | 2.19 |
| XM_945571.1 | ANKRD13D | ILMN_138370 | 2.19 |
| NM_199482.1 | PREI3 | ILMN_10571 | 2.19 |
| XM_934113.1 | LOC653489 | ILMN_42664 | 2.19 |
| NM_176811.2 | NLRP8 | ILMN_169055 | 2.19 |
| NM_178324.1 | SPTLC1 | ILMN_7889 | 2.19 |
| NM_006731.2 | FKTN | ILMN_6512 | 2.19 |
| NM_032794.1 | SLC44A4 | ILMN_14709 | 2.19 |
| NM_006494.1 | ERF | ILMN_14193 | 2.18 |
| NM_004566.2 | PFKFB3 | ILMN_163833 | 2.18 |
| NM_024900.3 | PHF17 | ILMN_1535 | 2.18 |
| NM_018257.1 | PCMTD2 | ILMN_4945 | 2.18 |
| XM_001133842.1 | LOC729446 | ILMN_169307 | 2.18 |
| NM_006472.2 | TXNIP | ILMN_9057 | 2.18 |
| NM_024077.3 | SECISBP2 | ILMN_19156 | 2.17 |
| NM_020310.2 | MNT | ILMN_21283 | 2.17 |
| NM_005920.2 | MEF2D | ILMN_3465 | 2.17 |
| NM_033389.2 | SSH2 | ILMN_8279 | 2.17 |
| NM_005921.1 | MAP3K1 | ILMN_309540 | 2.17 |
| NM_152641.2 | ARID2 | ILMN_163259 | 2.16 |
| NM_024969.2 | FAM130A2 | ILMN_20070 | 2.16 |
| NM_033631.2 | LUZP1 | ILMN_2667 | 2.16 |
| NM_002959.4 | SORT1 | ILMN_165748 | 2.15 |
| NM_018708.2 | FEM1A | ILMN_2838 | 2.15 |
| NM_033063.1 | MAP6 | ILMN_6882 | 2.14 |
| NM_199420.3 | POLQ | ILMN_10389 | 2.14 |
| NM_001013703.2 | EIF2AK4 | ILMN_164547 | 2.14 |
| NM_130437.2 | DYRK1A | ILMN_18751 | 2.14 |
| NM_033394.1 | TANC1 | ILMN_182363 | 2.14 |
| NM_052917.2 | GALNT13 | ILMN_180483 | 2.13 |
| NM_006100.2 | ST3GAL6 | ILMN_2870 | 2.13 |
| NM_033285.2 | TP53INP1 | ILMN_16203 | 2.12 |
| NM_004768.2 | SFRS11 | ILMN_4847 | 2.12 |
| XM_931434.2 | LOC400027 | ILMN_35789 | 2.11 |
| NM_178496.2 | C3orf59 | ILMN_14619 | 2.1 |
| NM_015352.1 | POFUT1 | ILMN_7876 | 2.1 |
| NM_206907.3 | PRKAA1 | ILMN_180991 | 2.1 |
| NM_031372.1 | HNRPDL | ILMN_15196 | 2.1 |
| XM_942501.1 | CSF2RA | ILMN_137685 | 2.1 |
| NM_001008219.1 | AMY1C | ILMN_28222 | 2.1 |
| NM_006283.1 | TACC1 | ILMN_20678 | 2.09 |
| NM_001008490.1 | KLF6 | ILMN_12381 | 2.09 |
| NM_003677.3 | DENR | ILMN_181187 | 2.09 |
| NM_001280.1 | CIRBP | ILMN_24327 | 2.09 |
| NM_000383.1 | AIRE | ILMN_179368 | 2.09 |
| NM_024546.2 | C13orf7 | ILMN_137373 | 2.08 |
| NM_001560.2 | IL13RA1 | ILMN_15736 | 2.08 |
| NM_016513.3 | ICK | ILMN_23886 | 2.08 |
| NM_020651.2 | PELI1 | ILMN_11771 | 2.08 |
| XR_019339.1 | LOC643668 | ILMN_179350 | 2.08 |
| NM_173797.2 | PAPD4 | ILMN_2190 | 2.08 |
| NM_133496.3 | SLC30A7 | ILMN_20389 | 2.08 |
| NM_015012.1 | TMEM41B | ILMN_8205 | 2.08 |
| XM_944716.1 | LOC440704 | ILMN_34488 | 2.08 |
| NM_003420.3 | ZNF35 | ILMN_180943 | 2.07 |
| NM_019119.3 | PCDHB9 | ILMN_23442 | 2.07 |
| NM_139235.3 | NOL6 | ILMN_7349 | 2.07 |
| NM_005494.2 | DNAJB6 | ILMN_26714 | 2.07 |
| NM_001005473.1 | PLCXD3 | ILMN_23362 | 2.07 |
| NM_006903.4 | PPA2 | ILMN_15173 | 2.07 |
| NM_018325.1 | C9orf72 | ILMN_7216 | 2.06 |
| NM_145687.2 | MAP4K4 | ILMN_28871 | 2.06 |
| NM_023080.1 | C8orf33 | ILMN_15901 | 2.06 |
| NM_032776.1 | JMJD1C | ILMN_164120 | 2.06 |
| NM_003017.3 | SFRS3 | ILMN_29649 | 2.06 |
| NM_001024071.1 | GCH1 | ILMN_14690 | 2.05 |
| NM_173515.2 | CNKSR3 | ILMN_25628 | 2.05 |
| NM_080702.2 | BAT3 | ILMN_4429 | 2.05 |
| NM_004634.2 | BRPF1 | ILMN_17537 | 2.05 |
| NM_173518.2 | C8orf45 | ILMN_22241 | 2.05 |
| NM_019007.3 | ARMCX6 | ILMN_6931 | 2.04 |
| NM_013995.1 | LAMP2 | ILMN_26730 | 2.04 |
| NM_015153.1 | PHF3 | ILMN_23658 | 2.04 |
| NM_138687.1 | PIP5K2B | ILMN_12735 | 2.04 |
| NM_018561.3 | USP49 | ILMN_24018 | 2.04 |
| NM_002813.4 | PSMD9 | ILMN_177354 | 2.03 |
| NM_001078.2 | VCAM1 | ILMN_3875 | 2.03 |
| NM_006047.4 | RBM12 | ILMN_183773 | 2.03 |
| NM_203499.1 | DDX42 | ILMN_1880 | 2.03 |
| XM_379215.2 | LOC132241 | ILMN_37830 | 2.02 |
| NM_031954.3 | KCTD10 | ILMN_30217 | 2.02 |
| NR_001562.1 | ANXA2P1 | ILMN_10494 | 2.02 |
| NM_017719.3 | SNRK | ILMN_5234 | 2.02 |
| NM_203401.1 | STMN1 | ILMN_12586 | 2.02 |
| NM_003211.3 | TDG | ILMN_29212 | 2.02 |
| NM_014395.1 | DAPP1 | ILMN_24094 | 2.02 |
| NM_175923.3 | MGC42630 | ILMN_138411 | 2.01 |
| NM_198489.1 | CCDC84 | ILMN_6803 | 2.01 |
| NM_020248.2 | CTNNBIP1 | ILMN_23888 | 2.01 |
| NM_014838.2 | ZBED4 | ILMN_8641 | 2.01 |
| NM_005238.2 | ETS1 | ILMN_173009 | 2.01 |
| NM_016605.1 | FAM53C | ILMN_11637 | 2.01 |
| NM_138477.2 | CDAN1 | ILMN_168162 | 2.01 |
| NM_020121.2 | UGCGL2 | ILMN_21506 | 2 |
| NM_020177.2 | FEM1C | ILMN_164027 | 2 |
| NM_013243.2 | SCG3 | ILMN_174345 | 2 |
| NM_018249.4 | CDK5RAP2 | ILMN_9876 | 2 |
| NM_022910.1 | NDRG4 | ILMN_8824 | 2 |
| NM_004687.3 | MTMR4 | ILMN_163329 | 2 |
| NM_001029862.1 | ANKRD30B | ILMN_7263 | 2 |
| NM_004598.3 | SPOCK1 | ILMN_25886 | 1.99 |
| NM_170695.2 | TGIF1 | ILMN_162784 | 1.99 |
| NM_017943.2 | FBXO34 | ILMN_172741 | 1.99 |
| NM_015534.4 | ZZZ3 | ILMN_14976 | 1.99 |
| NM_001093756.1 | FLJ13611 | ILMN_308292 | 1.98 |
| NM_003463.3 | PTP4A1 | ILMN_165831 | 1.98 |
| NM_020664.3 | DECR2 | ILMN_7935 | 1.98 |
| NM_006796.1 | AFG3L2 | ILMN_29564 | 1.98 |
| NM_152398.2 | OCIAD2 | ILMN_18246 | 1.98 |
| NM_001098495.1 | ZNF419 | ILMN_306789 | 1.98 |
| NR_003491.1 | MIAT | ILMN_308315 | 1.98 |
| NM_170695.2 | TGIF1 | ILMN_162784 | 1.97 |
| NM_175854.5 | PAN3 | ILMN_181888 | 1.97 |
| NM_006447.2 | USP16 | ILMN_29622 | 1.97 |
| NM_001017980.2 | LOC203547 | ILMN_163926 | 1.97 |
| NM_001029950.1 | DKFZp434K191 | ILMN_28495 | 1.96 |
| NM_033419.3 | PERLD1 | ILMN_12215 | 1.96 |
| NM_014729.2 | TOX | ILMN_16587 | 1.96 |
| NM_003110.4 | SP2 | ILMN_7882 | 1.96 |
| NM_001079673.1 | FNDC3A | ILMN_167386 | 1.96 |
| NM_001357.2 | DHX9 | ILMN_7196 | 1.96 |
| NM_004671.2 | PIAS2 | ILMN_11308 | 1.96 |
| NM_012257.3 | HBP1 | ILMN_167468 | 1.96 |
| XR_016986.1 | LOC643668 | ILMN_172192 | 1.96 |
| NM_002748.2 | MAPK6 | ILMN_22051 | 1.96 |
| NM_000572.2 | IL10 | ILMN_9173 | 1.96 |
| NM_017757.2 | ZNF407 | ILMN_12747 | 1.95 |
| NM_024610.4 | HSPBAP1 | ILMN_23171 | 1.95 |
| NM_014141.4 | CNTNAP2 | ILMN_176606 | 1.95 |
| NM_014034.1 | ASF1A | ILMN_1019 | 1.95 |
| NM_144578.2 | C14orf32 | ILMN_24999 | 1.95 |
| NM_017822.3 | C12orf41 | ILMN_3399 | 1.95 |
| NM_014382.2 | ATP2C1 | ILMN_16216 | 1.94 |
| NM_014023.3 | WDR37 | ILMN_175566 | 1.94 |
| NM_005010.3 | NRCAM | ILMN_8955 | 1.94 |
| NM_024561.3 | NARG1L | ILMN_22547 | 1.93 |
| NM_000553.3 | WRN | ILMN_168872 | 1.93 |
| NM_014380.1 | NGFRAP1 | ILMN_7162 | 1.93 |
| NM_015995.2 | KLF13 | ILMN_16226 | 1.93 |
| XM_936495.2 | LOC647346 | ILMN_36174 | 1.93 |
| NM_001009937.1 | SLC25A26 | ILMN_15004 | 1.92 |
| NM_014746.2 | RNF144 | ILMN_15740 | 1.92 |
| NM_006773.3 | DDX18 | ILMN_22238 | 1.92 |
| NM_022308.1 | ICA1 | ILMN_12918 | 1.92 |
| NR_003264.1 | SDHALP1 | ILMN_175200 | 1.92 |
| NM_001562.2 | IL18 | ILMN_167736 | 1.92 |
| NM_015124.2 | GRAMD4 | ILMN_12136 | 1.91 |
| NM_032866.3 | CGNL1 | ILMN_10819 | 1.91 |
| NM_012384.2 | GMEB2 | ILMN_7174 | 1.91 |
| NM_003342.4 | UBE2G1 | ILMN_179729 | 1.91 |
| NM_015655.2 | ZNF337 | ILMN_3280 | 1.91 |
| NM_004424.3 | E4F1 | ILMN_23848 | 1.91 |
| NM_001287.3 | CLCN7 | ILMN_8600 | 1.91 |
| NR_003664.1 | LOC389517 | ILMN_307371 | 1.91 |
| NM_018202.3 | TMEM57 | ILMN_30191 | 1.9 |
| NM_020474.2 | GALNT1 | ILMN_164550 | 1.9 |
| NM_015306.1 | USP24 | ILMN_309418 | 1.9 |
| NM_016357.3 | LIMA1 | ILMN_6603 | 1.9 |
| NM_001845.4 | COL4A1 | ILMN_24359 | 1.9 |
| NM_021942.4 | C4orf41 | ILMN_8900 | 1.9 |
| XM_929199.1 | LOC644250 | ILMN_30796 | 1.9 |
| NM_005010.3 | NRCAM | ILMN_8955 | 1.9 |
| NM_022487.2 | DCLRE1C | ILMN_28391 | 1.89 |
| NM_152679.2 | SLC10A4 | ILMN_1323 | 1.89 |
| NM_001006657.1 | WDR35 | ILMN_175554 | 1.89 |
| NM_004091.2 | E2F2 | ILMN_19730 | 1.89 |
| NM_004454.1 | ETV5 | ILMN_12676 | 1.89 |
| NM_001081640.1 | PRKDC | ILMN_180402 | 1.89 |
| NM_014897.1 | ZNF652 | ILMN_3215 | 1.89 |
| NM_170721.1 | MSI2 | ILMN_25750 | 1.88 |
| NM_015516.3 | TSKU | ILMN_29523 | 1.88 |
| NM_005665.4 | EVI5 | ILMN_17996 | 1.88 |
| NM_014268.1 | MAPRE2 | ILMN_8637 | 1.88 |
| NM_014913.2 | ADNP2 | ILMN_6906 | 1.88 |
| NM_014647.2 | KIAA0430 | ILMN_15427 | 1.88 |
| NM_002687.3 | PNN | ILMN_24088 | 1.88 |
| NM_080760.3 | DACH1 | ILMN_20187 | 1.88 |
| NM_001002878.1 | THOC5 | ILMN_13820 | 1.88 |
| NM_017896.2 | C20orf11 | ILMN_27220 | 1.88 |
| NM_001356.3 | DDX3X | ILMN_183040 | 1.88 |
| NM_002938.2 | RNF4 | ILMN_26467 | 1.87 |
| NM_152265.1 | BTF3L4 | ILMN_3105 | 1.87 |
| NM_025074.4 | FRAS1 | ILMN_165073 | 1.87 |
| NM_002923.1 | RGS2 | ILMN_26119 | 1.87 |
| XM_939697.1 | C9orf130 | ILMN_30981 | 1.86 |
| NM_002048.1 | GAS1 | ILMN_175833 | 1.86 |
| NM_001033505.1 | CSTF3 | ILMN_26942 | 1.86 |
| NM_002650.1 | PIK4CA | ILMN_20581 | 1.86 |
| NM_014667.1 | VGLL4 | ILMN_29344 | 1.86 |
| NM_013336.3 | SEC61A1 | ILMN_9397 | 1.86 |
| NM_006141.2 | DYNC1LI2 | ILMN_183290 | 1.86 |
| NM_001412.3 | EIF1AX | ILMN_22164 | 1.85 |
| NM_145913.2 | SLC5A8 | ILMN_7082 | 1.85 |
| NM_152557.3 | ZNF746 | ILMN_25894 | 1.85 |
| NM_133471.1 | KIAA1949 | ILMN_308966 | 1.85 |
| NM_003666.2 | BLZF1 | ILMN_21927 | 1.85 |
| NM_018999.2 | KIAA1128 | ILMN_173411 | 1.84 |
| NM_006421.3 | ARFGEF1 | ILMN_164295 | 1.84 |
| NM_032520.3 | GNPTG | ILMN_28173 | 1.84 |
| NM_020824.2 | ARHGAP21 | ILMN_10414 | 1.84 |
| NM_014839.3 | LPPR4 | ILMN_15660 | 1.83 |
| NM_177972.1 | TUB | ILMN_11520 | 1.83 |
| NM_017426.2 | NUP54 | ILMN_170157 | 1.83 |
| NM_001099668.1 | HIGD1A | ILMN_307999 | 1.83 |
| NM_018976.3 | SLC38A2 | ILMN_10001 | 1.83 |
| NM_003565.1 | ULK1 | ILMN_2158 | 1.83 |
| NM_017719.3 | SNRK | ILMN_5234 | 1.82 |
| NM_030665.3 | RAI1 | ILMN_176671 | 1.82 |
| NM_181784.1 | SPRED2 | ILMN_12131 | 1.81 |
| NM_001099652.1 | GPR137C | ILMN_308485 | 1.81 |
| NM_145701.1 | CDCA4 | ILMN_5601 | 1.81 |
| NM_014396.3 | VPS41 | ILMN_2386 | 1.81 |
| NM_013275.4 | ANKRD11 | ILMN_28595 | 1.81 |
| NM_015330.1 | SPECC1L | ILMN_168707 | 1.81 |
| NM_021145.2 | DMTF1 | ILMN_16919 | 1.81 |
| NM_001259.5 | CDK6 | ILMN_178275 | 1.81 |
| NM_172097.1 | CATSPER2 | ILMN_23478 | 1.81 |
| NM_014924.3 | KIAA0831 | ILMN_23945 | 1.8 |
| NM_144566.1 | ZNF700 | ILMN_7926 | 1.8 |
| NM_015477.1 | SIN3A | ILMN_14108 | 1.8 |
| NM_058172.3 | ANTXR2 | ILMN_165233 | 1.8 |
| NM_003565.1 | ULK1 | ILMN_2158 | 1.8 |
| NM_032590.3 | FBXL10 | ILMN_19365 | 1.8 |
| NM_006646.4 | WASF3 | ILMN_180336 | 1.79 |
| NM_021960.3 | MCL1 | ILMN_18397 | 1.79 |
| NM_201440.1 | PPHLN1 | ILMN_4445 | 1.79 |
| NM_006628.4 | ARPP-19 | ILMN_2093 | 1.79 |
| NM_000199.2 | SGSH | ILMN_7542 | 1.79 |
| NM_001081559.1 | CPSF4 | ILMN_178236 | 1.79 |
| NM_006999.3 | POLS | ILMN_866 | 1.78 |
| XR_018848.1 | LOC650369 | ILMN_169499 | 1.78 |
| NM_002737.2 | PRKCA | ILMN_24085 | 1.78 |
| NM_001023567.2 | GOLGA8B | ILMN_14405 | 1.78 |
| NM_017741.3 | C4orf30 | ILMN_172318 | 1.77 |
| NM_181054.1 | HIF1A | ILMN_9514 | 1.77 |
| NM_201559.2 | FOXO3 | ILMN_15525 | 1.77 |
| NM_001077440.1 | BCLAF1 | ILMN_162019 | 1.77 |
| NM_002657.2 | PLAGL2 | ILMN_23071 | 1.76 |
| NM_033446.1 | FAM125B | ILMN_20760 | 1.76 |
| NM_003966.2 | SEMA5A | ILMN_183828 | 1.76 |
| NM_001099270.1 | ZBTB34 | ILMN_307315 | 1.76 |
| NM_001144.4 | AMFR | ILMN_22219 | 1.76 |
| NM_014918.3 | CHSY1 | ILMN_17929 | 1.76 |
| NM_001632.3 | ALPP | ILMN_25184 | 1.76 |
| XM_370865.4 | LOC388122 | ILMN_46143 | 1.75 |
| NM_022768.4 | RBM15 | ILMN_1342 | 1.75 |
| XM_926231.1 | P704P | ILMN_36679 | 1.75 |
| NM_016626.3 | MEX3C | ILMN_172651 | 1.75 |
| NM_022826.2 | MARCH7 | ILMN_29166 | 1.75 |
| NM_001079842.1 | OCIAD1 | ILMN_163712 | 1.75 |
| NM_003617.2 | RGS5 | ILMN_167992 | 1.75 |
| NM_004396.2 | DDX5 | ILMN_20253 | 1.75 |
| NM_005375.2 | MYB | ILMN_15875 | 1.74 |
| NM_207304.1 | MBNL2 | ILMN_15554 | 1.74 |
| NM_003906.3 | MCM3AP | ILMN_19614 | 1.74 |
| NM_015690.2 | STK36 | ILMN_15506 | 1.74 |
| NM_178151.1 | DCX | ILMN_541 | 1.74 |
| NM_001111.3 | ADAR | ILMN_20593 | 1.74 |
| NM_016472.3 | C14orf129 | ILMN_7725 | 1.73 |
| XM_927769.1 | LOC653506 | ILMN_30995 | 1.73 |
| NM_018151.3 | RIF1 | ILMN_171812 | 1.73 |
| NM_001030272.1 | ARNTL | ILMN_6754 | 1.73 |
| NM_006159.1 | NELL2 | ILMN_26383 | 1.73 |
| NM_001005753.1 | VPS24 | ILMN_29671 | 1.73 |
| NM_002293.2 | LAMC1 | ILMN_182622 | 1.73 |
| NM_033196.2 | ZNF682 | ILMN_21081 | 1.73 |
| NM_000633.2 | BCL2 | ILMN_171007 | 1.72 |
| NM_014765.1 | TOMM20 | ILMN_20433 | 1.72 |
| NM_152834.2 | TMEM18 | ILMN_8053 | 1.71 |
| NM_006739.3 | MCM5 | ILMN_20107 | 1.71 |
| NM_007049.2 | BTN2A1 | ILMN_6995 | 1.71 |
| NM_031942.4 | CDCA7 | ILMN_33249 | 1.71 |
| NM_182646.1 | CPEB2 | ILMN_3402 | 1.7 |
| NM_006447.2 | USP16 | ILMN_29622 | 1.7 |
| NM_024653.3 | PRKRIP1 | ILMN_13077 | 1.7 |
| NM_020225.1 | STOX2 | ILMN_5597 | 1.7 |
| NM_000153.2 | GALC | ILMN_28156 | 1.7 |
| NM_016282.2 | AK3 | ILMN_6776 | 1.7 |
| NM_014839.3 | LPPR4 | ILMN_15660 | 1.7 |
| NM_001023587.1 | ABCC5 | ILMN_438 | 1.7 |
| NM_003469.3 | SCG2 | ILMN_17827 | 1.7 |
| NM_006925.3 | SFRS5 | ILMN_34497 | 1.7 |
| NM_006974.2 | ZNF33A | ILMN_4519 | 1.69 |
| NM_015902.4 | UBR5 | ILMN_178959 | 1.69 |
| NM_032856.2 | WDR73 | ILMN_16485 | 1.69 |
| NM_001003805.1 | ATP5S | ILMN_1363 | 1.69 |
| NM_016594.1 | FKBP11 | ILMN_14765 | 1.69 |
| NM_018622.5 | PARL | ILMN_163763 | 1.69 |
| NM_001079514.1 | UBN1 | ILMN_172742 | 1.69 |
| XM_927280.1 | LOC644033 | ILMN_39734 | 1.69 |
| NM_001048201.1 | UHRF1 | ILMN_162952 | 1.69 |
| NM_198431.1 | HSPA4 | ILMN_25293 | 1.69 |
| NM_015483.1 | KBTBD2 | ILMN_2507 | 1.69 |
| NM_020801.1 | ARRDC3 | ILMN_22538 | 1.68 |
| NM_152224.1 | PPEF1 | ILMN_24147 | 1.68 |
| NM_000633.2 | BCL2 | ILMN_171007 | 1.68 |
| NM_005433.3 | YES1 | ILMN_183786 | 1.68 |
| NM_005766.2 | FARP1 | ILMN_15608 | 1.68 |
| NM_001677.3 | ATP1B1 | ILMN_25542 | 1.68 |
| NM_006107.2 | CROP | ILMN_10300 | 1.68 |
| XM_495939.3 | KIAA1545 | ILMN_40920 | 1.67 |
| NM_201281.1 | MTMR2 | ILMN_24002 | 1.67 |
| NM_030633.1 | KIAA1712 | ILMN_5346 | 1.67 |
| NM_057175.3 | NARG1 | ILMN_16240 | 1.67 |
| NM_005819.4 | STX6 | ILMN_180926 | 1.67 |
| NM_021061.1 | ZNF250 | ILMN_25690 | 1.66 |
| NM_130809.2 | PRRC1 | ILMN_24905 | 1.66 |
| NM_057159.2 | LPAR1 | ILMN_28278 | 1.66 |
| NM_004526.2 | MCM2 | ILMN_183916 | 1.66 |
| NM_003934.1 | FUBP3 | ILMN_174872 | 1.66 |
| NM_006572.3 | GNA13 | ILMN_173569 | 1.66 |
| NM_022720.5 | DGCR8 | ILMN_1552 | 1.65 |
| NM_006516.1 | SLC2A1 | ILMN_421 | 1.65 |
| NM_032358.2 | CCDC77 | ILMN_23011 | 1.65 |
| NM_015001.2 | SPEN | ILMN_180751 | 1.65 |
| NM_032424.1 | KIAA1826 | ILMN_22604 | 1.65 |
| NM_006925.3 | SFRS5 | ILMN_34497 | 1.65 |
| NM_001017421.1 | FKSG30 | ILMN_2393 | 1.65 |
| NM_078470.2 | COX15 | ILMN_13504 | 1.64 |
| NM_019001.2 | XRN1 | ILMN_8924 | 1.64 |
| NM_003131.2 | SRF | ILMN_22299 | 1.64 |
| NM_198256.2 | E2F6 | ILMN_21495 | 1.64 |
| XM_930995.1 | LOC653086 | ILMN_31021 | 1.64 |
| NM_001001787.1 | ATP1B1 | ILMN_10855 | 1.64 |
| NM_033412.1 | MCART1 | ILMN_22327 | 1.64 |
| NM_003220.2 | TFAP2A | ILMN_17128 | 1.63 |
| NM_005688.2 | ABCC5 | ILMN_25223 | 1.63 |
| NM_022151.4 | MOAP1 | ILMN_165500 | 1.63 |
| NM_014366.4 | GNL3 | ILMN_18645 | 1.63 |
| NM_173666.1 | DTWD2 | ILMN_25915 | 1.63 |
| NM_001025202.2 | STAG3L2 | ILMN_5961 | 1.63 |
| NM_003621.1 | PPFIBP2 | ILMN_183115 | 1.62 |
| NM_013412.1 | RABL2A | ILMN_12484 | 1.62 |
| NM_002972.1 | SBF1 | ILMN_22729 | 1.62 |
| NM_014655.1 | SLC25A44 | ILMN_25848 | 1.62 |
| NM_032830.1 | CIRH1A | ILMN_2574 | 1.62 |
| NM_006007.1 | ZFAND5 | ILMN_22528 | 1.62 |
| NM_144582.2 | TEX261 | ILMN_27405 | 1.61 |
| NM_015396.3 | ARMC8 | ILMN_20420 | 1.61 |
| NM_001013690.1 | LOC401720 | ILMN_21595 | 1.61 |
| NM_001031617.2 | COX19 | ILMN_15655 | 1.61 |
| XM_940903.2 | ZC3H5 | ILMN_40646 | 1.61 |
| NM_015338.4 | ASXL1 | ILMN_183479 | 1.61 |
| NM_003690.3 | PRKRA | ILMN_3524 | 1.61 |
| NM_152679.2 | SLC10A4 | ILMN_1323 | 1.61 |
| NM_001008239.2 | C18orf25 | ILMN_981 | 1.61 |
| NM_025134.4 | CHD9 | ILMN_15866 | 1.61 |
| NM_032308.1 | RPAIN | ILMN_15409 | 1.61 |
| NM_004192.1 | ASMTL | ILMN_15304 | 1.6 |
| NM_032195.1 | SON | ILMN_8462 | 1.6 |
| NM_015346.2 | ZFYVE26 | ILMN_176163 | 1.6 |
| NM_199169.1 | PMEPA1 | ILMN_13834 | 1.6 |
| NM_002926.3 | RGS12 | ILMN_161894 | 1.6 |
| NM_030941.1 | LOC81691 | ILMN_17520 | 1.6 |
| NM_004641.2 | MLLT10 | ILMN_25545 | 1.6 |
| NM_001031685.2 | TP53BP2 | ILMN_9205 | 1.6 |
| NM_001002878.1 | THOC5 | ILMN_13820 | 1.6 |
| NM_152411.2 | ZNF786 | ILMN_6379 | 1.6 |
| NM_005561.2 | LAMP1 | ILMN_27826 | 1.6 |
| NM_003941.2 | WASL | ILMN_4756 | 1.6 |
| NM_032485.4 | MCM8 | ILMN_11171 | 1.6 |
| XM_932717.2 | LOC643224 | ILMN_34053 | 1.59 |
| NM_001037540.1 | SCML1 | ILMN_163338 | 1.59 |
| XM_290799.7 | ARHGAP23 | ILMN_162296 | 1.59 |
| NM_003183.4 | ADAM17 | ILMN_165100 | 1.59 |
| NM_024814.1 | CBLL1 | ILMN_24021 | 1.59 |
| NM_025135.2 | FHOD3 | ILMN_163420 | 1.59 |
| NM_003749.2 | IRS2 | ILMN_167991 | 1.59 |
| NM_206876.1 | PPP1CB | ILMN_22939 | 1.59 |
| NM_020728.1 | FAM62B | ILMN_19173 | 1.59 |
| NM_001032293.2 | ZNF207 | ILMN_21705 | 1.59 |
| NM_019110.3 | ZKSCAN4 | ILMN_29823 | 1.58 |
| NM_005207.2 | CRKL | ILMN_165503 | 1.58 |
| NM_002268.3 | KPNA4 | ILMN_21107 | 1.58 |
| NM_016284.3 | CNOT1 | ILMN_169268 | 1.58 |
| NM_022459.3 | XPO4 | ILMN_164187 | 1.57 |
| NM_015902.4 | UBR5 | ILMN_178959 | 1.57 |
| NM_014774.1 | KIAA0494 | ILMN_5593 | 1.57 |
| NM_015949.2 | C7orf20 | ILMN_23467 | 1.57 |
| NM_152562.2 | CDCA2 | ILMN_19331 | 1.57 |
| XM_945430.1 | SSR2 | ILMN_138339 | 1.57 |
| NM_198679.1 | RAPGEF1 | ILMN_177243 | 1.57 |
| NM_005238.2 | ETS1 | ILMN_173009 | 1.57 |
| NM_152570.1 | LINGO2 | ILMN_24238 | 1.57 |
| NM_020822.1 | KCNT1 | ILMN_21599 | 1.57 |
| NM_023924.3 | BRD9 | ILMN_12952 | 1.57 |
| NM_004126.3 | GNG11 | ILMN_8981 | 1.57 |
| NM_033109.2 | PNPT1 | ILMN_22316 | 1.57 |
| NM_176814.3 | ZNF800 | ILMN_163418 | 1.56 |
| NM_002128.4 | HMGB1 | ILMN_23421 | 1.56 |
| NM_014264.3 | PLK4 | ILMN_167207 | 1.56 |
| NM_144781.1 | PDCD2 | ILMN_16269 | 1.56 |
| NM_014423.3 | AFF4 | ILMN_179829 | 1.56 |
| NM_015308.1 | FNBP4 | ILMN_25895 | 1.56 |
| NM_015711.2 | GLTSCR1 | ILMN_18273 | 1.55 |
| NM_006997.2 | TACC2 | ILMN_16130 | 1.55 |
| NM_001007246.1 | BRWD1 | ILMN_28841 | 1.55 |
| NM_001037533.1 | GON4L | ILMN_14180 | 1.55 |
| NM_006628.4 | ARPP-19 | ILMN_2093 | 1.55 |
| NM_004284.3 | CHD1L | ILMN_7932 | 1.55 |
| XM_001129527.1 | KLF11 | ILMN_168976 | 1.55 |
| NM_015327.1 | SMG5 | ILMN_10815 | 1.55 |
| NM_006594.1 | AP4B1 | ILMN_20264 | 1.55 |
| NM_017991.3 | FLJ10081 | ILMN_469 | 1.55 |
| NM_002835.2 | PTPN12 | ILMN_26144 | 1.55 |
| NM_018235.1 | CNDP2 | ILMN_4139 | 1.55 |
| NM_022457.5 | RFWD2 | ILMN_1221 | 1.55 |
| XM_936467.2 | BEXL1 | ILMN_37437 | 1.55 |
| NM_152834.2 | TMEM18 | ILMN_8053 | 1.54 |
| NM_006631.2 | ZNF266 | ILMN_12055 | 1.54 |
| NM_001567.2 | INPPL1 | ILMN_20903 | 1.54 |
| NM_015144.2 | ZCCHC14 | ILMN_32176 | 1.54 |
| NM_053023.3 | ZFP91 | ILMN_7525 | 1.54 |
| NM_003472.2 | DEK | ILMN_24694 | 1.54 |
| NM_014988.1 | LIMCH1 | ILMN_3090 | 1.54 |
| NM_022483.3 | C5orf28 | ILMN_5037 | 1.54 |
| XR_018923.1 | LOC648210 | ILMN_162972 | 1.54 |
| NM_001037334.1 | USP14 | ILMN_12721 | 1.53 |
| NM_031845.2 | MAP2 | ILMN_38825 | 1.53 |
| NM_004560.2 | ROR2 | ILMN_22834 | 1.53 |
| NM_017566.2 | KLHDC4 | ILMN_8527 | 1.53 |
| NM_153756.1 | FNDC5 | ILMN_5239 | 1.53 |
| NM_001010864.1 | LOC196752 | ILMN_1138 | 1.53 |
| NM_004417.2 | DUSP1 | ILMN_20700 | 1.53 |
| NM_004618.3 | TOP3A | ILMN_167915 | 1.53 |
| NM_153188.2 | TNPO1 | ILMN_29083 | 1.53 |
| NM_025133.3 | FBXO11 | ILMN_18553 | 1.53 |
| NM_016143.3 | NSFL1C | ILMN_20493 | 1.52 |
| NM_012399.3 | PITPNB | ILMN_12439 | 1.52 |
| NM_004788.2 | UBE4A | ILMN_175730 | 1.52 |
| NM_014319.3 | LEMD3 | ILMN_178185 | 1.52 |
| NM_005520.1 | HNRPH1 | ILMN_4782 | 1.52 |
| NM_025138.3 | C13orf23 | ILMN_15197 | 1.52 |
| NM_003161.2 | RPS6KB1 | ILMN_162232 | 1.52 |
| NM_003819.2 | PABPC4 | ILMN_18446 | 1.52 |
| NM_007182.4 | RASSF1 | ILMN_8297 | 1.51 |
| NM_003804.3 | RIPK1 | ILMN_24351 | 1.51 |
| NM_145062.1 | ZUFSP | ILMN_24644 | 1.51 |
| XM_497029.2 | LOC441408 | ILMN_31941 | 1.51 |
| NM_022459.4 | XPO4 | ILMN_164187 | 1.51 |
| NM_053053.2 | TADA1L | ILMN_25791 | 1.51 |
| NM_032753.2 | RAXL1 | ILMN_21452 | 1.51 |
| NM_001304.3 | CPD | ILMN_163103 | 1.51 |
| NM_015317.1 | PUM2 | ILMN_12220 | 1.51 |
| NM_173511.2 | ALS2CR13 | ILMN_29052 | 1.51 |
| NM_182776.1 | MCM7 | ILMN_1133 | 1.51 |
| NM_001008393.1 | LOC201725 | ILMN_20795 | 1.5 |
| NM_173630.2 | RTTN | ILMN_5471 | 1.5 |
| NM_018364.3 | RSBN1 | ILMN_174594 | 1.5 |
| NM_139353.1 | TAF1C | ILMN_4122 | 1.5 |
| NM_006618.3 | JARID1B | ILMN_14812 | 1.5 |
| NM_003342.4 | UBE2G1 | ILMN_179729 | -1.5 |
| NM_001033566.1 | RHOT1 | ILMN_6821 | -1.5 |
| NM_001889.2 | CRYZ | ILMN_30248 | -1.5 |
| NM_032166.2 | ATRIP | ILMN_3211 | -1.51 |
| NM_181042.2 | PBRM1 | ILMN_16253 | -1.51 |
| NM_025128.3 | MUS81 | ILMN_29195 | -1.51 |
| NM_017653.2 | DYM | ILMN_6843 | -1.51 |
| NM_014241.3 | PTPLA | ILMN_24983 | -1.51 |
| NM_020155.2 | GPR137 | ILMN_24699 | -1.51 |
| NM_016037.2 | UTP11L | ILMN_2243 | -1.51 |
| NM_145697.1 | CDCA1 | ILMN_17725 | -1.52 |
| NM_001679.2 | ATP1B3 | ILMN_3785 | -1.52 |
| NM_030934.3 | C1orf25 | ILMN_163506 | -1.52 |
| NM_004398.2 | DDX10 | ILMN_20779 | -1.52 |
| NM_018845.2 | RAG1AP1 | ILMN_19038 | -1.52 |
| NM_053050.2 | MRPL53 | ILMN_25576 | -1.52 |
| NM_020158.3 | EXOSC5 | ILMN_6934 | -1.52 |
| NM_001382.2 | DPAGT1 | ILMN_10306 | -1.52 |
| NM_000945.3 | PPP3R1 | ILMN_26308 | -1.52 |
| NM_015935.4 | KIAA0859 | ILMN_172647 | -1.52 |
| NM_001382.2 | DPAGT1 | ILMN_10306 | -1.53 |
| NM_001031726.2 | C19orf12 | ILMN_10211 | -1.53 |
| NM_015607.2 | C1orf77 | ILMN_21997 | -1.53 |
| NM_002045.2 | GAP43 | ILMN_28511 | -1.53 |
| NM_004879.3 | EI24 | ILMN_8791 | -1.53 |
| NM_024336.1 | IRX3 | ILMN_4354 | -1.53 |
| NM_003221.3 | TFAP2B | ILMN_164377 | -1.53 |
| NM_021244.3 | RRAGD | ILMN_5663 | -1.54 |
| NM_006214.3 | PHYH | ILMN_18235 | -1.54 |
| NM_006874.2 | ELF2 | ILMN_1532 | -1.54 |
| NM_005896.2 | IDH1 | ILMN_14217 | -1.54 |
| NM_020230.4 | PPAN | ILMN_25948 | -1.54 |
| NM_058181.1 | C21orf57 | ILMN_21121 | -1.54 |
| NM_181306.1 | MRPL52 | ILMN_16276 | -1.54 |
| NM_000701.6 | ATP1A1 | ILMN_677 | -1.54 |
| NM_016319.1 | COPS7A | ILMN_13902 | -1.54 |
| NM_001007230.1 | SPOP | ILMN_12838 | -1.54 |
| NM_002156.4 | HSPD1 | ILMN_178202 | -1.55 |
| NM_152705.1 | POLR1D | ILMN_28050 | -1.55 |
| NM_018044.2 | NSUN5 | ILMN_895 | -1.55 |
| NM_005274.1 | GNG5 | ILMN_21191 | -1.55 |
| NM_016086.2 | STYXL1 | ILMN_5068 | -1.55 |
| NM_003129.3 | SQLE | ILMN_521 | -1.55 |
| NM_001487.1 | BLOC1S1 | ILMN_14526 | -1.55 |
| NM_144594.1 | GTSF1 | ILMN_17221 | -1.56 |
| NM_018079.3 | SRBD1 | ILMN_28720 | -1.56 |
| NM_014977.2 | ACIN1 | ILMN_164296 | -1.56 |
| NM_001075098.1 | MOCS1 | ILMN_176423 | -1.57 |
| NM_005378.4 | MYCN | ILMN_178034 | -1.57 |
| NM_016108.2 | AIG1 | ILMN_22004 | -1.57 |
| NM_001914.2 | CYB5A | ILMN_25182 | -1.57 |
| NM_001012643.2 | LOC339344 | ILMN_6535 | -1.57 |
| NM_002486.4 | NCBP1 | ILMN_23411 | -1.57 |
| NM_006282.2 | STK4 | ILMN_21491 | -1.57 |
| NM_032361.1 | THOC3 | ILMN_17969 | -1.57 |
| NM_000505.3 | F12 | ILMN_181878 | -1.57 |
| NM_001042532.1 | COASY | ILMN_171680 | -1.57 |
| NM_001077628.1 | APH1A | ILMN_180233 | -1.57 |
| NM_018844.2 | BCAP29 | ILMN_24686 | -1.58 |
| NM_197956.1 | C9orf90 | ILMN_16848 | -1.58 |
| NM_032459.1 | EFS | ILMN_17620 | -1.58 |
| NM_000155.2 | GALT | ILMN_1433 | -1.58 |
| NM_198336.1 | INSIG1 | ILMN_12839 | -1.58 |
| NM_003776.2 | MRPL40 | ILMN_21771 | -1.58 |
| NM_016059.3 | PPIL1 | ILMN_30246 | -1.58 |
| NM_003107.2 | SOX4 | ILMN_17456 | -1.58 |
| NM_005850.3 | SF3B4 | ILMN_12460 | -1.58 |
| NM_016098.1 | BRP44L | ILMN_4349 | -1.59 |
| NM_053067.1 | UBQLN1 | ILMN_9768 | -1.59 |
| NM_001040668.1 | BCL2L12 | ILMN_177176 | -1.59 |
| NM_016390.2 | C9orf114 | ILMN_20184 | -1.59 |
| NM_031298.2 | TMEM93 | ILMN_9888 | -1.59 |
| NM_012453.2 | TBL2 | ILMN_25753 | -1.6 |
| NM_005188.2 | CBL | ILMN_172998 | -1.6 |
| NR_000029.1 | RPL23AP7 | ILMN_3185 | -1.6 |
| XM_936103.1 | LOC642033 | ILMN_33652 | -1.6 |
| NM_033362.2 | MRPS12 | ILMN_19234 | -1.6 |
| NM_022494.1 | ZDHHC6 | ILMN_1193 | -1.6 |
| NM_003715.2 | USO1 | ILMN_23419 | -1.6 |
| NM_024099.3 | C11orf48 | ILMN_24145 | -1.6 |
| NM_018110.2 | DOK4 | ILMN_5422 | -1.6 |
| NM_016071.2 | MRPS33 | ILMN_4243 | -1.6 |
| NM_181800.1 | UBE2C | ILMN_25999 | -1.6 |
| NM_016002.2 | SCCPDH | ILMN_30353 | -1.61 |
| NM_175875.3 | SIX5 | ILMN_21099 | -1.61 |
| NM_005740.2 | DNAL4 | ILMN_22246 | -1.61 |
| NM_001535.2 | PRMT2 | ILMN_10737 | -1.61 |
| NM_021076.2 | NEFH | ILMN_9306 | -1.61 |
| NM_006807.3 | CBX1 | ILMN_162583 | -1.61 |
| NM_004864.1 | GDF15 | ILMN_2688 | -1.61 |
| NM_019095.3 | CRLS1 | ILMN_14031 | -1.61 |
| NM_001866.2 | COX7B | ILMN_19298 | -1.61 |
| NM_001002755.1 | NFU1 | ILMN_9748 | -1.62 |
| NM_020119.3 | ZC3HAV1 | ILMN_13243 | -1.62 |
| XM_929738.1 | LOC646786 | ILMN_38919 | -1.62 |
| NM_198970.1 | AES | ILMN_25198 | -1.62 |
| NM_001005498.2 | RHBDF2 | ILMN_23030 | -1.63 |
| NM_001017405.1 | MAEA | ILMN_4828 | -1.63 |
| NM_024710.1 | ISOC2 | ILMN_27084 | -1.63 |
| NM_007271.2 | STK38 | ILMN_8385 | -1.63 |
| NM_199122.1 | TBRG4 | ILMN_22982 | -1.63 |
| NM_001048172.1 | MUTYH | ILMN_164733 | -1.63 |
| NM_025205.3 | MED28 | ILMN_14574 | -1.63 |
| NM_014188.2 | SSU72 | ILMN_29116 | -1.63 |
| NM_004891.2 | MRPL33 | ILMN_12897 | -1.63 |
| NM_198038.1 | NUDT9 | ILMN_12448 | -1.64 |
| NM_024011.2 | CDC2L2 | ILMN_20434 | -1.64 |
| NM_001914.2 | CYB5A | ILMN_25182 | -1.64 |
| NM_002157.1 | HSPE1 | ILMN_2612 | -1.64 |
| NM_201443.1 | TEAD4 | ILMN_21735 | -1.65 |
| NM_001005209.1 | TMEM198 | ILMN_6726 | -1.65 |
| NM_001033026.1 | C19orf6 | ILMN_167551 | -1.65 |
| NM_012289.3 | KEAP1 | ILMN_18799 | -1.65 |
| NM_001033503.1 | SAR1B | ILMN_16595 | -1.65 |
| NM_018206.3 | VPS35 | ILMN_21093 | -1.65 |
| XM_930344.2 | LOC644934 | ILMN_43758 | -1.65 |
| NM_052848.1 | CCDC97 | ILMN_24401 | -1.66 |
| NM_001033566.1 | RHOT1 | ILMN_6821 | -1.66 |
| NM_006681.1 | NMU | ILMN_28975 | -1.66 |
| NM_018847.2 | KLHL9 | ILMN_20376 | -1.66 |
| NM_138316.2 | PANK1 | ILMN_406 | -1.66 |
| NM_001007027.2 | ALG8 | ILMN_176006 | -1.66 |
| NM_020196.2 | XAB2 | ILMN_30213 | -1.67 |
| NM_001017392.2 | SFRS14 | ILMN_17110 | -1.67 |
| NM_133375.2 | DIS3L | ILMN_29373 | -1.67 |
| NM_005830.2 | MRPS31 | ILMN_6293 | -1.67 |
| NM_016647.2 | C8orf55 | ILMN_25304 | -1.67 |
| NM_014168.2 | METTL5 | ILMN_9336 | -1.67 |
| NM_032985.4 | SEC23B | ILMN_181566 | -1.67 |
| NM_002225.2 | IVD | ILMN_13293 | -1.68 |
| XM_936354.2 | LOC642197 | ILMN_44406 | -1.68 |
| NM_001042370.1 | TROVE2 | ILMN_173505 | -1.68 |
| NM_004435.2 | ENDOG | ILMN_26482 | -1.68 |
| NM_032747.2 | USMG5 | ILMN_10409 | -1.68 |
| NM_018718.1 | TSGA14 | ILMN_11000 | -1.68 |
| NM_003211.3 | TDG | ILMN_29212 | -1.68 |
| NM_138809.3 | CMBL | ILMN_1485 | -1.68 |
| NM_014886.3 | TINP1 | ILMN_8436 | -1.68 |
| NM_004886.3 | APBA3 | ILMN_4538 | -1.69 |
| NM_015871.3 | ZNF593 | ILMN_20499 | -1.69 |
| NM_001121.2 | ADD3 | ILMN_4026 | -1.69 |
| NM_001032382.1 | PQBP1 | ILMN_24607 | -1.69 |
| XM_001130192.1 | KIAA1160 | ILMN_162086 | -1.69 |
| NM_174908.2 | CCDC50 | ILMN_12155 | -1.69 |
| NM_021254.1 | C21orf59 | ILMN_28603 | -1.69 |
| NM_006819.1 | STIP1 | ILMN_28761 | -1.69 |
| NM_001827.1 | CKS2 | ILMN_14702 | -1.69 |
| NM_016587.2 | CBX3 | ILMN_11642 | -1.69 |
| NM_032346.1 | PDCD2L | ILMN_25365 | -1.7 |
| NM_001080501.1 | MGC3196 | ILMN_181711 | -1.7 |
| NM_001003793.1 | RBMS3 | ILMN_16411 | -1.7 |
| NM_018846.2 | KLHL7 | ILMN_21425 | -1.7 |
| NM_019037.2 | EXOSC4 | ILMN_25178 | -1.7 |
| NM_006736.5 | DNAJB2 | ILMN_34421 | -1.7 |
| NM_199235.1 | COLEC11 | ILMN_6793 | -1.7 |
| NM_000527.2 | LDLR | ILMN_10126 | -1.71 |
| NM_018170.2 | P15RS | ILMN_174036 | -1.71 |
| NM_018130.2 | SHQ1 | ILMN_29855 | -1.72 |
| NM_004089.3 | TSC22D3 | ILMN_9893 | -1.72 |
| NM_002028.3 | FNTB | ILMN_171968 | -1.72 |
| NM_032343.1 | CHCHD6 | ILMN_19685 | -1.72 |
| NM_001005368.1 | ZNF32 | ILMN_181781 | -1.72 |
| NM_001031706.1 | PLEKHB2 | ILMN_179121 | -1.72 |
| NM_000100.2 | CSTB | ILMN_26819 | -1.72 |
| XM_938497.2 | C6orf52 | ILMN_42173 | -1.73 |
| NM_004982.2 | KCNJ8 | ILMN_29993 | -1.73 |
| NM_016126.1 | C1orf41 | ILMN_4683 | -1.73 |
| NM_004643.1 | PABPN1 | ILMN_29660 | -1.73 |
| NM_032340.2 | C6orf125 | ILMN_21424 | -1.73 |
| NM_138701.1 | C7orf11 | ILMN_20229 | -1.74 |
| NM_024057.2 | NUP37 | ILMN_4147 | -1.74 |
| XM_939954.2 | LOC388789 | ILMN_39285 | -1.74 |
| NM_207118.1 | GTF2H5 | ILMN_26206 | -1.74 |
| NM_004175.3 | SNRPD3 | ILMN_163179 | -1.75 |
| NM_024678.3 | NARS2 | ILMN_13605 | -1.75 |
| NM_001827.1 | CKS2 | ILMN_14702 | -1.75 |
| NM_002491.1 | NDUFB3 | ILMN_22320 | -1.75 |
| NM_032848.1 | C12orf52 | ILMN_22595 | -1.76 |
| XR_019071.1 | LOC642333 | ILMN_183964 | -1.76 |
| NM_001031717.2 | CRELD1 | ILMN_14216 | -1.76 |
| NM_001321.1 | CSRP2 | ILMN_3862 | -1.76 |
| NM_001078650.1 | TMEM134 | ILMN_183533 | -1.76 |
| NM_052873.1 | C14orf179 | ILMN_22122 | -1.76 |
| NM_006589.2 | C1orf2 | ILMN_20176 | -1.76 |
| NM_178136.1 | POLDIP3 | ILMN_21842 | -1.77 |
| NM_022075.3 | LASS2 | ILMN_10647 | -1.77 |
| NM_002067.1 | GNA11 | ILMN_25749 | -1.77 |
| NM_001040668.1 | BCL2L12 | ILMN_177176 | -1.78 |
| NM_017693.2 | BIVM | ILMN_181297 | -1.78 |
| NM_005713.1 | COL4A3BP | ILMN_10635 | -1.78 |
| NM_006554.3 | MTX2 | ILMN_17112 | -1.78 |
| XM_944786.1 | LOC650737 | ILMN_40280 | -1.78 |
| NM_002598.2 | PDCD2 | ILMN_5469 | -1.78 |
| NM_201262.1 | DNAJC12 | ILMN_18576 | -1.78 |
| NM_003021.3 | SGTA | ILMN_1162 | -1.79 |
| NM_012117.1 | CBX5 | ILMN_25072 | -1.79 |
| NM_004378.1 | CRABP1 | ILMN_12739 | -1.8 |
| NM_001077395.1 | DPH5 | ILMN_181061 | -1.8 |
| NM_201434.1 | RAB5C | ILMN_176672 | -1.8 |
| NM_005476.3 | GNE | ILMN_29772 | -1.8 |
| NM_001640.3 | APEH | ILMN_27694 | -1.8 |
| NM_021242.4 | MID1IP1 | ILMN_161908 | -1.81 |
| NM_145074.2 | HTRA2 | ILMN_12587 | -1.81 |
| NM_032490.4 | C14orf142 | ILMN_166160 | -1.81 |
| NM_182523.1 | C3orf68 | ILMN_4406 | -1.81 |
| NM_014078.4 | MRPL13 | ILMN_17393 | -1.81 |
| NM_033091.1 | TRIM4 | ILMN_8530 | -1.81 |
| NM_032448.1 | FAM120B | ILMN_10767 | -1.81 |
| NM_170784.1 | MKKS | ILMN_17701 | -1.81 |
| NM_006602.2 | TCFL5 | ILMN_12278 | -1.82 |
| NM_001040011.1 | C9orf119 | ILMN_306686 | -1.82 |
| NM_007308.1 | SNCA | ILMN_2235 | -1.82 |
| NM_005997.1 | VPS72 | ILMN_17901 | -1.82 |
| NM_032439.1 | PHYHIPL | ILMN_22045 | -1.82 |
| NM_152274.2 | FAM58A | ILMN_3352 | -1.83 |
| NM_015609.2 | C1orf144 | ILMN_5836 | -1.83 |
| NM_003083.2 | SNAPC2 | ILMN_14587 | -1.83 |
| NM_001031703.2 | TMEM103 | ILMN_40105 | -1.84 |
| NM_015386.2 | COG4 | ILMN_28901 | -1.84 |
| NM_198527.2 | HDDC3 | ILMN_29602 | -1.84 |
| NM_004422.2 | DVL2 | ILMN_29320 | -1.84 |
| NM_001842.3 | CNTFR | ILMN_21040 | -1.84 |
| XM_371655.3 | LOC389137 | ILMN_163284 | -1.84 |
| XM_938779.1 | LOC653972 | ILMN_31111 | -1.85 |
| NM_018983.3 | NOLA1 | ILMN_14204 | -1.85 |
| NM_001007793.1 | BUB3 | ILMN_5688 | -1.85 |
| NM_001813.2 | CENPE | ILMN_7509 | -1.85 |
| NM_003721.2 | RFXANK | ILMN_7789 | -1.85 |
| NM_006644.2 | HSPH1 | ILMN_1157 | -1.85 |
| NM_017865.2 | ZNF692 | ILMN_22649 | -1.86 |
| NM_005708.2 | GPC6 | ILMN_16550 | -1.86 |
| NM_012138.3 | AATF | ILMN_29906 | -1.86 |
| NM_001008405.1 | BCAP29 | ILMN_24800 | -1.86 |
| NM_020235.3 | BBX | ILMN_28437 | -1.86 |
| NM_006876.1 | B3GNT6 | ILMN_16433 | -1.87 |
| NM_016297.2 | PCYOX1 | ILMN_15130 | -1.87 |
| NM_014161.2 | MRPL18 | ILMN_14120 | -1.87 |
| NM_018480.2 | TMEM126B | ILMN_18826 | -1.87 |
| NM_007083.3 | NUDT6 | ILMN_903 | -1.88 |
| NM_003729.2 | RTCD1 | ILMN_11697 | -1.88 |
| NM_024292.2 | UBL5 | ILMN_14261 | -1.88 |
| NM_032316.3 | NICN1 | ILMN_17764 | -1.88 |
| XM_377476.4 | MGC57346 | ILMN_165970 | -1.89 |
| NM_138781.2 | LOC113386 | ILMN_12569 | -1.89 |
| NM_006963.3 | ZNF22 | ILMN_165495 | -1.89 |
| NM_145274.2 | TMEM99 | ILMN_25105 | -1.9 |
| NM_133646.2 | ZAK | ILMN_5666 | -1.9 |
| NM_000051.3 | ATM | ILMN_162851 | -1.9 |
| NM_080651.1 | MED30 | ILMN_7158 | -1.9 |
| NM_018464.2 | CISD1 | ILMN_4843 | -1.9 |
| NM_018840.2 | C20orf24 | ILMN_10676 | -1.9 |
| NM_001002019.1 | PUS1 | ILMN_13055 | -1.91 |
| NM_024540.2 | MRPL24 | ILMN_29128 | -1.91 |
| NM_018164.1 | C12orf11 | ILMN_14707 | -1.91 |
| NM_178507.2 | OAF | ILMN_12751 | -1.92 |
| NM_207380.1 | C15orf52 | ILMN_1132 | -1.92 |
| NM_006090.3 | CEPT1 | ILMN_14637 | -1.92 |
| NR_003287.1 | LOC100008589 | ILMN_177351 | -1.92 |
| NM_002601.2 | PDE6D | ILMN_2430 | -1.92 |
| NM_001466.2 | FZD2 | ILMN_12499 | -1.92 |
| NM_007100.2 | ATP5I | ILMN_14284 | -1.92 |
| NM_207350.1 | MGC72104 | ILMN_26269 | -1.92 |
| NM_006178.1 | NSF | ILMN_23282 | -1.92 |
| NM_004821.1 | HAND1 | ILMN_29799 | -1.92 |
| NM_012405.3 | ICMT | ILMN_31192 | -1.93 |
| NM_003787.1 | NOL4 | ILMN_16134 | -1.93 |
| NM_002413.3 | MGST2 | ILMN_8759 | -1.93 |
| NM_001552.2 | IGFBP4 | ILMN_9309 | -1.93 |
| NM_001002246.1 | ANAPC11 | ILMN_5565 | -1.93 |
| NM_013299.3 | SAC3D1 | ILMN_9385 | -1.93 |
| NM_004901.2 | ENTPD4 | ILMN_19012 | -1.94 |
| NM_005905.3 | SMAD9 | ILMN_28187 | -1.94 |
| NM_024296.3 | CCDC28B | ILMN_26263 | -1.94 |
| NM_004885.1 | NPFFR2 | ILMN_20676 | -1.95 |
| NM_181876.2 | PPP2R2C | ILMN_15268 | -1.95 |
| NM_004615.2 | TSPAN7 | ILMN_20684 | -1.95 |
| NM_194326.2 | RPS19BP1 | ILMN_8107 | -1.95 |
| NM_014142.2 | NUDT5 | ILMN_1656 | -1.95 |
| NM_139159.3 | DPP9 | ILMN_26244 | -1.96 |
| NM_007277.4 | EXOC3 | ILMN_6110 | -1.96 |
| NM_014046.2 | MRPS18B | ILMN_8749 | -1.96 |
| NM_018304.2 | PRR11 | ILMN_32619 | -1.96 |
| NM_007342.1 | NUPL2 | ILMN_2154 | -1.96 |
| NM_023926.3 | ZSCAN18 | ILMN_16020 | -1.96 |
| NM_201414.1 | APP | ILMN_23272 | -1.96 |
| XM_930694.1 | LOC642477 | ILMN_36253 | -1.96 |
| NM_198047.1 | HIBCH | ILMN_24888 | -1.97 |
| NM_001077268.1 | ZFYVE19 | ILMN_175347 | -1.97 |
| NM_001014812.1 | FAM96A | ILMN_13780 | -1.97 |
| NM_018357.2 | LARP6 | ILMN_25584 | -1.97 |
| NM_012111.1 | AHSA1 | ILMN_11051 | -1.97 |
| NM_001283.2 | AP1S1 | ILMN_21653 | -1.97 |
| NM_174942.1 | GAS2L3 | ILMN_5609 | -1.98 |
| NM_014260.2 | PFDN6 | ILMN_8046 | -1.98 |
| NM_022652.2 | DUSP6 | ILMN_5926 | -1.98 |
| NM_015449.2 | C1orf43 | ILMN_933 | -1.98 |
| NM_016332.2 | SEPX1 | ILMN_7309 | -1.98 |
| NM_213720.1 | C22orf16 | ILMN_25503 | -1.98 |
| NM_003924.2 | PHOX2B | ILMN_172224 | -1.98 |
| XM_927071.2 | LOC643790 | ILMN_38875 | -1.99 |
| NM_005926.2 | MFAP1 | ILMN_20656 | -1.99 |
| NM_005692.3 | ABCF2 | ILMN_14116 | -1.99 |
| XM_001126125.1 | PGAM5 | ILMN_178005 | -2 |
| NM_001048197.1 | SNHG3-RCC1 | ILMN_167397 | -2 |
| NM_012133.2 | COPG2 | ILMN_23766 | -2 |
| NM_005869.2 | SDCCAG10 | ILMN_3741 | -2 |
| NM_134268.3 | CYGB | ILMN_8058 | -2 |
| NM_006233.4 | POLR2I | ILMN_17223 | -2 |
| NM_006392.2 | NOL5A | ILMN_13841 | -2 |
| XM_936354.2 | LOC642197 | ILMN_44406 | -2.01 |
| NM_007155.4 | ZP3 | ILMN_17555 | -2.01 |
| NM_145644.1 | MRPL35 | ILMN_20736 | -2.01 |
| NM_005749.2 | TOB1 | ILMN_13735 | -2.02 |
| NM_152755.1 | CNPY4 | ILMN_15383 | -2.02 |
| NM_001124.1 | ADM | ILMN_29514 | -2.02 |
| NM_006281.2 | STK3 | ILMN_26935 | -2.03 |
| NM_001326.2 | CSTF3 | ILMN_27551 | -2.03 |
| NM_212552.2 | BOLA3 | ILMN_28776 | -2.03 |
| NM_006014.3 | LAGE3 | ILMN_1071 | -2.03 |
| NM_016374.5 | ARID4B | ILMN_162934 | -2.04 |
| NM_003628.3 | PKP4 | ILMN_11784 | -2.05 |
| NM_006717.2 | SPIN1 | ILMN_23742 | -2.05 |
| NM_146387.1 | MRPL4 | ILMN_13422 | -2.05 |
| NM_014169.2 | CHMP4A | ILMN_19959 | -2.05 |
| NM_023005.2 | BAZ1B | ILMN_16290 | -2.05 |
| NM_014964.3 | EPN2 | ILMN_21232 | -2.06 |
| NM_001017369.1 | SC4MOL | ILMN_2901 | -2.06 |
| NM_001035505.1 | BOLA3 | ILMN_29223 | -2.06 |
| NM_021177.3 | LSM2 | ILMN_22587 | -2.06 |
| NM_020851.1 | ISLR2 | ILMN_19345 | -2.07 |
| NM_002897.3 | RBMS1 | ILMN_18726 | -2.08 |
| NM_080605.3 | B3GALT6 | ILMN_170784 | -2.08 |
| NM_006191.2 | PA2G4 | ILMN_28541 | -2.08 |
| NM_017802.2 | HEATR2 | ILMN_1114 | -2.08 |
| NM_202468.1 | GIPC1 | ILMN_21354 | -2.08 |
| NR_001449.1 | TRK1 | ILMN_6493 | -2.09 |
| NM_201414.1 | APP | ILMN_23272 | -2.09 |
| NM_023937.2 | MRPL34 | ILMN_5839 | -2.09 |
| NM_032673.2 | PCGF1 | ILMN_5720 | -2.1 |
| NM_002896.1 | RBM4 | ILMN_11057 | -2.1 |
| NM_054014.1 | FKBP1A | ILMN_29213 | -2.1 |
| NM_000282.2 | PCCA | ILMN_6045 | -2.11 |
| NM_022893.2 | BCL11A | ILMN_17359 | -2.11 |
| NM_014596.4 | ZNRD1 | ILMN_20009 | -2.11 |
| NM_152524.3 | SGOL2 | ILMN_743 | -2.11 |
| NM_001896.2 | CSNK2A2 | ILMN_16798 | -2.11 |
| NM_005573.2 | LMNB1 | ILMN_4100 | -2.11 |
| NM_053067.1 | UBQLN1 | ILMN_9768 | -2.12 |
| NM_002263.2 | KIFC1 | ILMN_8595 | -2.12 |
| XM_001132711.1 | RFNG | ILMN_168322 | -2.12 |
| NM_020147.2 | THAP10 | ILMN_182683 | -2.13 |
| XM_935818.1 | FLJ20397 | ILMN_137080 | -2.13 |
| NM_003341.3 | UBE2E1 | ILMN_7115 | -2.13 |
| XM_926382.2 | LOC642755 | ILMN_32084 | -2.14 |
| NM_153682.2 | PIGP | ILMN_18625 | -2.14 |
| NM_177983.1 | PPM1G | ILMN_878 | -2.15 |
| NM_023936.1 | MRPS34 | ILMN_5723 | -2.15 |
| XM_936372.2 | LMTK3 | ILMN_35173 | -2.15 |
| NM_006761.3 | YWHAE | ILMN_18524 | -2.15 |
| NR_001445.1 | RN7SK | ILMN_14457 | -2.15 |
| NM_014161.2 | MRPL18 | ILMN_14120 | -2.15 |
| NM_138797.1 | ANKRD54 | ILMN_21813 | -2.16 |
| NM_012460.2 | TIMM9 | ILMN_9968 | -2.16 |
| NM_032476.2 | MRPS6 | ILMN_17239 | -2.16 |
| NM_013300.1 | C12orf24 | ILMN_24807 | -2.17 |
| NM_016053.2 | CCDC53 | ILMN_25394 | -2.17 |
| NM_152362.1 | TNFAIP8L1 | ILMN_3344 | -2.18 |
| NM_006455.2 | SC65 | ILMN_21605 | -2.18 |
| NM_002613.3 | PDPK1 | ILMN_27765 | -2.19 |
| NM_003800.3 | RNGTT | ILMN_17056 | -2.19 |
| NM_003400.3 | XPO1 | ILMN_16600 | -2.19 |
| NM_006145.1 | DNAJB1 | ILMN_19740 | -2.2 |
| NM_006310.2 | NPEPPS | ILMN_184074 | -2.2 |
| NM_001031684.1 | SFRS7 | ILMN_7620 | -2.2 |
| NM_180976.1 | PPP2R5D | ILMN_2366 | -2.21 |
| NM_175069.1 | APTX | ILMN_7416 | -2.21 |
| XM_001131304.1 | LOC728635 | ILMN_168315 | -2.22 |
| NM_024321.3 | RBM42 | ILMN_182570 | -2.22 |
| NM_012433.2 | SF3B1 | ILMN_168075 | -2.22 |
| NM_004544.2 | NDUFA10 | ILMN_7463 | -2.22 |
| NM_001634.4 | AMD1 | ILMN_21529 | -2.22 |
| NM_152318.2 | C12orf45 | ILMN_25959 | -2.23 |
| NM_025233.5 | COASY | ILMN_13627 | -2.23 |
| NM_207346.2 | TSEN54 | ILMN_8569 | -2.23 |
| NM_001080546.1 | LOC219854 | ILMN_168339 | -2.23 |
| NM_032361.1 | THOC3 | ILMN_17969 | -2.23 |
| NM_033115.2 | MGC16169 | ILMN_16160 | -2.24 |
| NM_153824.1 | PYCR1 | ILMN_8761 | -2.25 |
| NM_001042549.1 | NSL1 | ILMN_164300 | -2.25 |
| NM_000076.1 | CDKN1C | ILMN_20689 | -2.26 |
| NM_032344.1 | NUDT22 | ILMN_19793 | -2.27 |
| NM_213596.1 | FOXN4 | ILMN_25618 | -2.28 |
| NM_020385.2 | REXO4 | ILMN_29774 | -2.28 |
| NM_057089.2 | AP1S1 | ILMN_4691 | -2.28 |
| NM_205847.1 | GMPPA | ILMN_23338 | -2.29 |
| NM_020449.2 | THOC2 | ILMN_162047 | -2.29 |
| NM_180981.1 | MRPL52 | ILMN_3474 | -2.29 |
| NM_005694.1 | COX17 | ILMN_19252 | -2.29 |
| NM_145647.2 | WDR67 | ILMN_20846 | -2.3 |
| NM_022087.2 | GALNT11 | ILMN_5237 | -2.3 |
| NM_017958.1 | PLEKHB2 | ILMN_29704 | -2.31 |
| NM_002712.1 | PPP1R7 | ILMN_29559 | -2.31 |
| NM_005413.1 | SIX3 | ILMN_26476 | -2.31 |
| NM_014180.2 | MRPL22 | ILMN_29349 | -2.31 |
| NM_001025248.1 | DUT | ILMN_163345 | -2.31 |
| NM_005192.2 | CDKN3 | ILMN_4098 | -2.31 |
| NM_001535.2 | PRMT2 | ILMN_10737 | -2.32 |
| NM_016071.2 | MRPS33 | ILMN_4243 | -2.32 |
| NM_138444.3 | KCTD12 | ILMN_18501 | -2.32 |
| NM_016042.2 | EXOSC3 | ILMN_174330 | -2.33 |
| NM_001031677.2 | RAB24 | ILMN_25731 | -2.33 |
| NM_003077.2 | SMARCD2 | ILMN_14227 | -2.34 |
| NM_001042588.1 | SNUPN | ILMN_178280 | -2.35 |
| NM_016401.2 | C11orf73 | ILMN_22672 | -2.35 |
| NM_015523.2 | REXO2 | ILMN_15016 | -2.35 |
| NM_001008566.1 | TPST2 | ILMN_13248 | -2.36 |
| NM_013328.2 | PYCR2 | ILMN_18209 | -2.36 |
| NM_005169.2 | PHOX2A | ILMN_10858 | -2.36 |
| XM_934985.1 | LOC400879 | ILMN_31001 | -2.36 |
| NM_032574.2 | DPY30 | ILMN_18534 | -2.36 |
| NM_173659.2 | RPUSD3 | ILMN_28804 | -2.37 |
| NM_018297.2 | NGLY1 | ILMN_15318 | -2.37 |
| NM_153333.2 | TCEAL8 | ILMN_12551 | -2.37 |
| NM_003924.2 | PHOX2B | ILMN_14205 | -2.37 |
| NM_001007794.1 | CEPT1 | ILMN_15134 | -2.38 |
| NM_138361.3 | LRSAM1 | ILMN_21244 | -2.39 |
| NM_017812.2 | CHCHD3 | ILMN_23539 | -2.39 |
| NM_152912.3 | MTIF3 | ILMN_16655 | -2.4 |
| NM_002086.3 | GRB2 | ILMN_173749 | -2.4 |
| XM_933956.1 | LOC644162 | ILMN_43225 | -2.4 |
| NM_000913.3 | OPRL1 | ILMN_6491 | -2.41 |
| NM_003368.4 | USP1 | ILMN_5285 | -2.41 |
| NM_031434.2 | TMUB1 | ILMN_11025 | -2.41 |
| NM_012475.4 | USP21 | ILMN_18019 | -2.44 |
| NM_182533.1 | C1orf86 | ILMN_2880 | -2.44 |
| NM_057089.2 | AP1S1 | ILMN_4691 | -2.44 |
| NM_002528.4 | NTHL1 | ILMN_15981 | -2.45 |
| NM_005810.3 | KLRG1 | ILMN_12613 | -2.46 |
| NM_000628.3 | IL10RB | ILMN_26097 | -2.46 |
| NM_032638.3 | GATA2 | ILMN_20021 | -2.46 |
| NM_012475.4 | USP21 | ILMN_18019 | -2.47 |
| NM_016561.1 | BFAR | ILMN_23440 | -2.49 |
| NM_012170.2 | FBXO22 | ILMN_5718 | -2.5 |
| NM_004365.2 | CETN3 | ILMN_25663 | -2.5 |
| NM_016551.1 | TM7SF3 | ILMN_7797 | -2.51 |
| NM_024516.2 | C16orf53 | ILMN_20272 | -2.51 |
| NM_006391.1 | IPO7 | ILMN_28842 | -2.51 |
| NM_005644.2 | TAF12 | ILMN_3797 | -2.51 |
| NM_003746.1 | DNCL1 | ILMN_137049 | -2.52 |
| NM_058216.1 | RAD51C | ILMN_2944 | -2.53 |
| NM_001539.2 | DNAJA1 | ILMN_5819 | -2.53 |
| NM_005836.2 | HRSP12 | ILMN_8062 | -2.54 |
| NM_001042401.1 | C21orf51 | ILMN_179828 | -2.55 |
| NM_024527.4 | ABHD8 | ILMN_23791 | -2.55 |
| NM_181876.2 | PPP2R2C | ILMN_15268 | -2.56 |
| NM_001008783.1 | SLC35D3 | ILMN_16642 | -2.56 |
| NM_002095.4 | GTF2E2 | ILMN_4316 | -2.56 |
| NM_021971.1 | GMPPB | ILMN_3929 | -2.57 |
| NM_006416.3 | SLC35A1 | ILMN_23284 | -2.57 |
| NM_001031711.1 | ERGIC1 | ILMN_7272 | -2.59 |
| NM_006158.2 | NEFL | ILMN_22054 | -2.6 |
| NM_001545.1 | ICT1 | ILMN_11458 | -2.6 |
| NM_005833.2 | RABEPK | ILMN_4050 | -2.62 |
| NM_001031677.2 | RAB24 | ILMN_25731 | -2.63 |
| XM_925818.1 | LOC642282 | ILMN_41968 | -2.65 |
| NM_079837.2 | BANP | ILMN_8638 | -2.66 |
| NM_032772.3 | ZNF503 | ILMN_2048 | -2.66 |
| NM_003062.1 | SLIT3 | ILMN_18656 | -2.66 |
| NM_002335.1 | LRP5 | ILMN_19887 | -2.67 |
| NM_178439.3 | GMCL1 | ILMN_3285 | -2.67 |
| NM_198391.1 | FLRT3 | ILMN_23273 | -2.67 |
| NM_019858.1 | GPR162 | ILMN_27943 | -2.67 |
| NM_006745.3 | SC4MOL | ILMN_2770 | -2.68 |
| NM_000856.3 | GUCY1A3 | ILMN_11680 | -2.68 |
| NM_058246.3 | DNAJB6 | ILMN_7651 | -2.68 |
| NM_017895.6 | DDX27 | ILMN_20732 | -2.69 |
| NM_173529.3 | C18orf54 | ILMN_168867 | -2.7 |
| NM_007167.2 | ZMYM6 | ILMN_1275 | -2.7 |
| XM_374020.4 | LOC375295 | ILMN_45377 | -2.7 |
| NM_030771.1 | CCDC34 | ILMN_2645 | -2.71 |
| NM_001007157.1 | PHF14 | ILMN_2096 | -2.71 |
| NM_020153.2 | C11orf60 | ILMN_171038 | -2.72 |
| NM_022079.2 | HERC4 | ILMN_8869 | -2.72 |
| NM_004252.2 | SLC9A3R1 | ILMN_1421 | -2.72 |
| NM_020705.1 | TBC1D24 | ILMN_34755 | -2.74 |
| NM_005652.2 | TERF2 | ILMN_21134 | -2.75 |
| NM_024516.2 | C16orf53 | ILMN_20272 | -2.78 |
| NM_018390.2 | PLCXD1 | ILMN_8273 | -2.79 |
| NM_001078651.1 | TMEM134 | ILMN_176754 | -2.79 |
| NM_178314.2 | RILPL1 | ILMN_1609 | -2.8 |
| NM_016374.5 | ARID4B | ILMN_162934 | -2.8 |
| NM_024090.1 | ELOVL6 | ILMN_11340 | -2.81 |
| NM_032728.2 | PPAPDC3 | ILMN_25638 | -2.82 |
| XM_926370.1 | LOC642989 | ILMN_33765 | -2.82 |
| NM_007280.1 | OIP5 | ILMN_18200 | -2.84 |
| XM_930284.1 | LOC441763 | ILMN_36192 | -2.84 |
| NM_001009608.1 | C20orf94 | ILMN_24801 | -2.85 |
| NM_032226.2 | ZCCHC7 | ILMN_21489 | -2.86 |
| NM_148973.1 | TNFRSF25 | ILMN_14916 | -2.86 |
| NM_033212.2 | CCDC102A | ILMN_12942 | -2.87 |
| NM_178439.3 | GMCL1 | ILMN_3285 | -2.89 |
| NM_015948.2 | SLC35B3 | ILMN_20545 | -2.9 |
| NM_006597.3 | HSPA8 | ILMN_181529 | -2.9 |
| NM_006265.1 | RAD21 | ILMN_171453 | -2.91 |
| NM_022903.3 | CCDC71 | ILMN_21600 | -2.93 |
| NM_002643.3 | PIGF | ILMN_15261 | -2.95 |
| XM_926249.2 | LOC642852 | ILMN_40586 | -2.96 |
| NM_001037494.1 | DYNLL1 | ILMN_14802 | -2.96 |
| NM_001039141.1 | TRIOBP | ILMN_34620 | -2.99 |
| XM_944321.1 | LOC402560 | ILMN_42108 | -2.99 |
| NM_006182.2 | DDR2 | ILMN_20698 | -2.99 |
| NM_030805.2 | LMAN2L | ILMN_1985 | -3 |
| XM_498571.2 | LOC440160 | ILMN_33035 | -3 |
| NM_001007157.1 | PHF14 | ILMN_2096 | -3 |
| NM_014847.2 | UBAP2L | ILMN_163836 | -3.01 |
| NM_014140.2 | SMARCAL1 | ILMN_19734 | -3.02 |
| NM_014254.1 | TMEM5 | ILMN_26271 | -3.02 |
| NM_001365.2 | DLG4 | ILMN_164548 | -3.03 |
| NM_001039703.1 | NBPF10 | ILMN_45673 | -3.06 |
| NM_058216.1 | RAD51C | ILMN_2944 | -3.11 |
| XM_941876.1 | BRI3BP | ILMN_139088 | -3.12 |
| NM_138418.2 | C16orf14 | ILMN_9509 | -3.13 |
| NM_148178.1 | C9orf23 | ILMN_3926 | -3.14 |
| NM_199487.1 | UQCC | ILMN_16175 | -3.19 |
| NM_019116.2 | UBFD1 | ILMN_179383 | -3.21 |
| NM_007198.2 | PROSC | ILMN_23472 | -3.22 |
| NM_017489.1 | TERF1 | ILMN_164297 | -3.22 |
| NM_002166.4 | ID2 | ILMN_28481 | -3.24 |
| NM_138807.2 | C3orf31 | ILMN_9705 | -3.32 |
| NM_184234.1 | RBM39 | ILMN_20330 | -3.32 |
| NM_005873.2 | RGS19 | ILMN_42727 | -3.34 |
| NM_153201.1 | HSPA8 | ILMN_14829 | -3.35 |
| NM_079837.2 | BANP | ILMN_8638 | -3.37 |
| NM_001040142.1 | SCN2A | ILMN_167124 | -3.43 |
| NM_014322.2 | OPN3 | ILMN_166169 | -3.49 |
| NM_138720.1 | HIST1H2BD | ILMN_17622 | -3.58 |
| NM_138720.1 | HIST1H2BD | ILMN_17622 | -3.6 |
| NM_002166.4 | ID2 | ILMN_28481 | -3.65 |
| NM_018473.2 | THEM2 | ILMN_27212 | -3.66 |
| NM_020749.3 | MTUS1 | ILMN_4658 | -3.72 |
| NM_000819.3 | GART | ILMN_22974 | -3.74 |
| NM_003512.3 | HIST1H2AC | ILMN_26493 | -3.96 |
| NM_004456.3 | EZH2 | ILMN_25740 | -4.01 |
| NM_080723.3 | NRSN1 | ILMN_178353 | -4.09 |
| NM_004316.2 | ASCL1 | ILMN_23892 | -4.09 |
| NM_006860.2 | RABL4 | ILMN_4559 | -4.1 |
| NM_002167.2 | ID3 | ILMN_6829 | -4.13 |
| NM_001037675.1 | NBPF20 | ILMN_26956 | -4.18 |
| NM_014941.1 | MORC2 | ILMN_12502 | -4.27 |
| NM_078629.1 | MSL3L1 | ILMN_29354 | -4.87 |
| NM_005345.4 | HSPA1A | ILMN_6623 | -5.73 |
| NM_005346.3 | HSPA1B | ILMN_25549 | -6.54 |
