# Supplementary material for: Minimal Peroxide Exposure of Neuronal Cells Induces Multifaceted Adaptive Responses
Source: PLoS One. 2010 Dec 17;5(12):e14352. doi: 10.1371/journal.pone.0014352 (PMC3003681; doi:10.1371/journal.pone.0014352)
Supplement: Table S8 — MeCh-significantly regulated genes after 8 hours of stimulation in the CMP state SH-SY5Y cells. Each significantly regulated gene is described via its accession number (ACCESSION), Gene Symbol (SYMBOL), Illumina array transcript designation (TRANSCRIPT). For each gene the z-ratio of expression compared to untreated cells after 8 hours of ligand stimulation is displayed (CMP MeCh 8). (0.99 MB DOC) [file pone.0014352.s015.doc]

**Table S8. MeCh-significantly regulated genes after 8 hours of stimulation in the CMP state SH-SY5Y cells**. Each significantly regulated gene is described via its accession number (ACCESSION), Gene Symbol (SYMBOL), Illumina array transcript designation (TRANSCRIPT). For each gene the z-ratio of expression compared to un-treated cells after 8 hours of ligand stimulation is displayed (CMP MeCh 8).

| **ACCESSION** | **SYMBOL** | **TRANSCRIPT** | **CMP MeCh 8** |
| --- | --- | --- | --- |
| NM_000584.2 | IL8 | ILMN_179575 | 10.06 |
| NM_024663.3 | NPEPL1 | ILMN_175218 | 4.6 |
| NM_001001391.1 | CD44 | ILMN_10947 | 4.49 |
| NM_207035.1 | C1orf63 | ILMN_22593 | 4.24 |
| NM_001008219.1 | AMY1C | ILMN_28222 | 4.6 |
| NM_001077188.1 | HS6ST2 | ILMN_182242 | 4.15 |
| XM_945571.1 | ANKRD13D | ILMN_138370 | 3.92 |
| NM_001040456.1 | RHBDD2 | ILMN_168345 | 4.18 |
| NM_001496.3 | GFRA3 | ILMN_8392 | 3.87 |
| NM_006157.2 | NELL1 | ILMN_2560 | 3.84 |
| NM_001033506.1 | CSTF3 | ILMN_27049 | 3.8 |
| NM_002160.2 | TNC | ILMN_14948 | 3.54 |
| NM_015690.2 | STK36 | ILMN_15506 | 3.66 |
| NM_002673.3 | PLXNB1 | ILMN_22628 | 3.51 |
| NM_024525.2 | TTC13 | ILMN_164005 | 3.37 |
| NM_004598.3 | SPOCK1 | ILMN_25886 | 3.35 |
| NM_001040456.1 | RHBDD2 | ILMN_168345 | 3.59 |
| NM_020133.2 | AGPAT4 | ILMN_24920 | 3.22 |
| NM_175923.3 | MGC42630 | ILMN_138411 | 3.23 |
| NM_152322.2 | BTBD11 | ILMN_506 | 3.16 |
| NR_003491.1 | MIAT | ILMN_308315 | 3.43 |
| NM_024909.1 | C6orf134 | ILMN_21139 | 3.23 |
| NM_005245.3 | FAT | ILMN_24617 | 3.17 |
| NM_001095.2 | ACCN2 | ILMN_27416 | 3.17 |
| NM_005781.4 | TNK2 | ILMN_5336 | 3.25 |
| NM_020897.1 | HCN3 | ILMN_20127 | 3.16 |
| NM_017514.2 | PLXNA3 | ILMN_162939 | 3.11 |
| NM_018416.2 | FOXJ2 | ILMN_165896 | 3.12 |
| NM_001013690.1 | LOC401720 | ILMN_21595 | 3.1 |
| NM_003086.2 | SNAPC4 | ILMN_180505 | 3.28 |
| NM_006715.2 | MAN2C1 | ILMN_685 | 3.08 |
| NM_022910.1 | NDRG4 | ILMN_8824 | 3.3 |
| NM_015306.1 | USP24 | ILMN_309418 | 3.12 |
| NM_001007246.1 | BRWD1 | ILMN_28841 | 3.09 |
| NM_014000.2 | VCL | ILMN_27566 | 3.2 |
| NM_003972.2 | BTAF1 | ILMN_8616 | 3 |
| NM_004567.2 | PFKFB4 | ILMN_163968 | 2.98 |
| NM_003913.3 | PRPF4B | ILMN_139391 | 2.84 |
| XM_940209.1 | KIAA0194 | ILMN_37512 | 3.02 |
| NM_203364.2 | CAPRIN1 | ILMN_9771 | 3.05 |
| NM_181722.2 | LOC285908 | ILMN_180740 | 2.7 |
| NM_000199.2 | SGSH | ILMN_7542 | 3.13 |
| NM_003461.4 | ZYX | ILMN_2137 | 2.93 |
| NM_003458.3 | BSN | ILMN_22754 | 2.85 |
| NM_022450.2 | RHBDF1 | ILMN_20892 | 2.77 |
| NM_006421.3 | ARFGEF1 | ILMN_164295 | 2.91 |
| NM_032788.1 | ZNF514 | ILMN_14476 | 2.83 |
| NM_152398.2 | OCIAD2 | ILMN_18246 | 2.98 |
| NM_014975.1 | MAST1 | ILMN_14016 | 2.7 |
| NM_001037533.1 | GON4L | ILMN_14180 | 2.85 |
| NM_004071.2 | CLK1 | ILMN_162592 | 3.03 |
| NM_004402.2 | DFFB | ILMN_14684 | 2.75 |
| NM_005128.2 | DOPEY2 | ILMN_164626 | 2.8 |
| NM_005808.2 | CTDSPL | ILMN_510 | 2.92 |
| NM_144736.3 | PRO1853 | ILMN_15591 | 2.65 |
| NM_172014.1 | TNFSF14 | ILMN_9666 | 2.82 |
| XM_375152.3 | LOC400304 | ILMN_46003 | 2.7 |
| NM_005688.2 | ABCC5 | ILMN_25223 | 2.73 |
| NM_020892.1 | DTX2 | ILMN_21612 | 2.82 |
| NM_178831.4 | GATS | ILMN_18755 | 2.86 |
| NM_003635.2 | NDST2 | ILMN_21220 | 2.64 |
| NM_003622.2 | PPFIBP1 | ILMN_172147 | 2.58 |
| NM_001078.2 | VCAM1 | ILMN_3875 | 2.73 |
| NM_199245.1 | VAMP1 | ILMN_10901 | 2.65 |
| NM_002609.3 | PDGFRB | ILMN_25767 | 2.86 |
| NM_015433.2 | FAM119B | ILMN_17350 | 2.73 |
| NM_003082.2 | SNAPC1 | ILMN_177713 | 2.53 |
| NM_017643.1 | MBTD1 | ILMN_29908 | 2.47 |
| NM_005385.3 | NKTR | ILMN_23378 | 2.8 |
| NM_025251.1 | KIAA1688 | ILMN_28510 | 2.57 |
| NM_015308.1 | FNBP4 | ILMN_25895 | 2.83 |
| NM_005922.2 | MAP3K4 | ILMN_6743 | 2.54 |
| NM_018044.2 | NSUN5 | ILMN_895 | 2.43 |
| NM_001080453.1 | INTS1 | ILMN_173681 | 2.65 |
| NM_001017391.1 | SULT1A4 | ILMN_20533 | 2.64 |
| NM_006494.1 | ERF | ILMN_14193 | 2.47 |
| NM_006045.1 | ATP9A | ILMN_176431 | 2.69 |
| NM_025058.3 | TRIM46 | ILMN_18492 | 2.6 |
| NM_017925.4 | DENND4C | ILMN_2455 | 2.46 |
| NM_001013258.1 | ZNF789 | ILMN_11535 | 2.48 |
| NM_033063.1 | MAP6 | ILMN_6882 | 2.48 |
| XM_001132754.1 | LOC728734 | ILMN_169578 | 2.61 |
| NM_007055.2 | POLR3A | ILMN_1449 | 2.62 |
| NM_001347.2 | DGKQ | ILMN_27065 | 2.41 |
| NM_001010915.1 | PTPLAD2 | ILMN_6355 | 2.59 |
| NM_005224.2 | ARID3A | ILMN_18757 | 2.39 |
| NM_012400.2 | PLA2G2D | ILMN_163941 | 2.39 |
| NM_001008408.3 | RBM33 | ILMN_165407 | 2.54 |
| NM_025074.4 | FRAS1 | ILMN_165073 | 2.44 |
| NM_002737.2 | PRKCA | ILMN_24085 | 2.78 |
| NM_002972.1 | SBF1 | ILMN_22729 | 2.42 |
| NM_001003786.1 | LYK5 | ILMN_2367 | 2.59 |
| NM_015245.2 | ANKS1A | ILMN_25376 | 2.44 |
| NM_149379.1 | NSUN5C | ILMN_19309 | 2.63 |
| NM_003047.2 | SLC9A1 | ILMN_166750 | 2.39 |
| NM_019001.2 | XRN1 | ILMN_8924 | 2.35 |
| NM_172097.1 | CATSPER2 | ILMN_23478 | 2.86 |
| NM_025152.1 | NUBPL | ILMN_25397 | 2.52 |
| NM_015026.1 | MON2 | ILMN_19004 | 2.27 |
| NM_182492.1 | LRP5L | ILMN_650 | 2.27 |
| XM_944716.1 | LOC440704 | ILMN_34488 | 2.5 |
| NM_002926.3 | RGS12 | ILMN_161894 | 2.37 |
| NM_005341.1 | ZBTB48 | ILMN_29232 | 2.37 |
| NM_014071.2 | NCOA6 | ILMN_20599 | 2.55 |
| NM_023080.1 | C8orf33 | ILMN_15901 | 2.34 |
| NM_005090.2 | PLA2G4B | ILMN_6705 | 2.28 |
| NM_006145.1 | DNAJB1 | ILMN_19740 | 2.34 |
| NM_021942.4 | C4orf41 | ILMN_8900 | 2.47 |
| NM_001677.3 | ATP1B1 | ILMN_25542 | 2.64 |
| NM_021737.1 | CLCN6 | ILMN_6195 | 2.25 |
| NM_002473.3 | MYH9 | ILMN_183555 | 2.48 |
| NM_206852.1 | RTN1 | ILMN_3435 | 2.58 |
| NM_001006115.2 | IHPK1 | ILMN_8379 | 2.42 |
| NM_145687.2 | MAP4K4 | ILMN_28871 | 2.35 |
| NM_013361.3 | ZNF223 | ILMN_166150 | 2.39 |
| NM_024698.4 | SLC25A22 | ILMN_13935 | 2.33 |
| NM_024519.2 | FAM65A | ILMN_17641 | 2.35 |
| NM_030633.1 | KIAA1712 | ILMN_5346 | 2.18 |
| NM_001251.2 | CD68 | ILMN_5188 | 2.24 |
| NM_003906.3 | MCM3AP | ILMN_19614 | 2.31 |
| NM_030808.3 | NDEL1 | ILMN_20362 | 2.32 |
| NM_145913.2 | SLC5A8 | ILMN_7082 | 2.07 |
| NM_001481.1 | GAS8 | ILMN_26809 | 2.16 |
| NM_006767.3 | LZTR1 | ILMN_18977 | 2.41 |
| NM_178526.1 | SLC25A42 | ILMN_5992 | 2.17 |
| NM_031454.1 | SELO | ILMN_29590 | 2.15 |
| NM_175085.1 | GART | ILMN_19282 | 2.4 |
| NM_001013685.1 | LOC401357 | ILMN_29013 | 2.19 |
| NM_001080484.1 | KIAA1751 | ILMN_180591 | 2.38 |
| NM_020713.1 | ZNF512B | ILMN_173427 | 2.09 |
| NM_020447.3 | C15orf17 | ILMN_13536 | 2.24 |
| NM_014853.2 | SGSM2 | ILMN_9226 | 2.49 |
| NM_198480.2 | ZNF615 | ILMN_26774 | 2.08 |
| NM_001692.3 | ATP6V1B1 | ILMN_28016 | 2.13 |
| NM_031469.2 | SH3BGRL2 | ILMN_9801 | 2.3 |
| NM_018223.1 | CHFR | ILMN_26892 | 2.33 |
| NM_014829.2 | DDX46 | ILMN_165992 | 2.25 |
| NM_004859.3 | CLTC | ILMN_171089 | 2.08 |
| NM_078470.2 | COX15 | ILMN_13504 | 2.14 |
| NM_173854.4 | SLC41A1 | ILMN_2825 | 2.14 |
| NM_002319.2 | LRCH4 | ILMN_139402 | 2 |
| NM_020748.1 | INTS2 | ILMN_1948 | 2.12 |
| NM_001012643.2 | LOC339344 | ILMN_6535 | 2.26 |
| NM_001144.4 | AMFR | ILMN_22219 | 2.21 |
| NM_003119.2 | SPG7 | ILMN_26332 | 2.34 |
| XM_001133677.1 | LOC729264 | ILMN_170805 | 2.1 |
| NM_144781.1 | PDCD2 | ILMN_16269 | 2.3 |
| NM_134426.2 | SLC26A6 | ILMN_5076 | 2.1 |
| NM_015447.1 | CAMSAP1 | ILMN_815 | 2.18 |
| NM_178324.1 | SPTLC1 | ILMN_7889 | 2.2 |
| XM_928464.1 | LOC146517 | ILMN_32888 | 2.13 |
| NM_033631.2 | LUZP1 | ILMN_2667 | 2.15 |
| NM_020452.2 | ATP8B2 | ILMN_19940 | 2.1 |
| NM_006011.3 | ST8SIA2 | ILMN_19287 | 2.19 |
| NM_003677.3 | DENR | ILMN_181187 | 2.16 |
| NM_024048.2 | MGC3020 | ILMN_29369 | 2.11 |
| NM_004444.4 | EPHB4 | ILMN_11176 | 2.07 |
| NM_212469.1 | CHKA | ILMN_28401 | 2.11 |
| NM_024514.4 | CYP2R1 | ILMN_8966 | 2.02 |
| NM_080491.1 | GAB2 | ILMN_3317 | 2.22 |
| NM_178025.1 | GGTL3 | ILMN_23263 | 1.97 |
| NM_198489.1 | CCDC84 | ILMN_6803 | 2.01 |
| NM_145294.4 | WDR90 | ILMN_29490 | 1.95 |
| NM_002685.2 | EXOSC10 | ILMN_25853 | 2.13 |
| NM_012463.2 | ATP6V0A2 | ILMN_23163 | 1.99 |
| NM_014422.2 | PIB5PA | ILMN_8156 | 2 |
| NM_173042.2 | IL18BP | ILMN_30884 | 1.88 |
| NM_016282.2 | AK3 | ILMN_6776 | 2.21 |
| NM_001752.2 | CAT | ILMN_13962 | 2.06 |
| NM_025132.3 | WDR19 | ILMN_11749 | 2.17 |
| NM_015157.1 | PHLDB1 | ILMN_3997 | 2.1 |
| NM_018330.4 | KIAA1598 | ILMN_4741 | 2.1 |
| NM_181050.1 | AXIN1 | ILMN_6274 | 2.13 |
| NM_022167.2 | XYLT2 | ILMN_26042 | 2.13 |
| NM_012197.2 | RABGAP1 | ILMN_7110 | 2.15 |
| XM_371461.4 | KIAA1671 | ILMN_42090 | 1.96 |
| NM_001031623.2 | ZNF451 | ILMN_990 | 2.08 |
| NM_003462.3 | DNALI1 | ILMN_24038 | 2.03 |
| NM_176811.2 | NLRP8 | ILMN_169055 | 2.4 |
| NM_001040439.1 | MAPK8IP3 | ILMN_174436 | 2.11 |
| NM_001009182.1 | SIP1 | ILMN_5662 | 1.97 |
| NM_003434.3 | ZNF133 | ILMN_5864 | 2 |
| NM_014363.3 | SACS | ILMN_180142 | 2.06 |
| NM_002959.4 | SORT1 | ILMN_165748 | 2.07 |
| NM_001387.2 | DPYSL3 | ILMN_23309 | 2.27 |
| NM_015348.1 | TMEM131 | ILMN_308809 | 2.1 |
| XM_936495.2 | LOC647346 | ILMN_36174 | 2.14 |
| NM_018121.2 | C10orf6 | ILMN_24540 | 2 |
| NM_014917.2 | NTNG1 | ILMN_2708 | 2 |
| NM_022742.3 | CCDC136 | ILMN_183005 | 2.11 |
| XM_938988.1 | LOC402221 | ILMN_35678 | 2.17 |
| NM_032508.1 | TMEM185A | ILMN_24307 | 1.92 |
| NM_006123.2 | IDS | ILMN_17605 | 2.06 |
| NM_020704.1 | FAM40B | ILMN_18452 | 1.98 |
| NM_014014.2 | ASCC3L1 | ILMN_18834 | 1.91 |
| NM_015346.2 | ZFYVE26 | ILMN_176163 | 1.93 |
| NM_018697.3 | LANCL2 | ILMN_920 | 1.97 |
| NM_016143.3 | NSFL1C | ILMN_20493 | 1.93 |
| XR_016986.1 | LOC643668 | ILMN_172192 | 2.09 |
| NM_014867.1 | KBTBD11 | ILMN_20625 | 1.92 |
| XM_001133202.1 | KIAA0363 | ILMN_166209 | 2.23 |
| NM_015144.2 | ZCCHC14 | ILMN_32176 | 2.09 |
| NM_002771.2 | PRSS3 | ILMN_19426 | 2.04 |
| NM_178231.1 | ALS2CR14 | ILMN_947 | 1.85 |
| XM_939697.1 | C9orf130 | ILMN_30981 | 1.89 |
| NM_078481.2 | CD97 | ILMN_26363 | 1.89 |
| XR_016048.1 | MGC40489 | ILMN_171153 | 2.05 |
| NM_005665.4 | EVI5 | ILMN_17996 | 1.98 |
| NM_173602.2 | DIP2B | ILMN_179302 | 2.02 |
| NM_007049.2 | BTN2A1 | ILMN_6995 | 1.96 |
| NM_033505.2 | SELI | ILMN_18750 | 1.92 |
| NM_001013635.2 | LOC387856 | ILMN_30286 | 1.84 |
| NM_001029862.1 | ANKRD30B | ILMN_7263 | 2.29 |
| NM_006005.2 | WFS1 | ILMN_18545 | 2.01 |
| NM_024610.4 | HSPBAP1 | ILMN_23171 | 1.87 |
| NM_184231.1 | NCKIPSD | ILMN_15946 | 1.81 |
| NM_019106.4 | MARCH7 | ILMN_4065 | 2.04 |
| NM_006107.2 | CROP | ILMN_10300 | 2.27 |
| NM_079837.2 | BANP | ILMN_8638 | 1.9 |
| NM_016212.2 | TP53TG3 | ILMN_36480 | 1.98 |
| NM_004458.1 | ACSL4 | ILMN_12915 | 1.8 |
| XR_019339.1 | LOC643668 | ILMN_179350 | 1.96 |
| NM_001001794.2 | FAM116B | ILMN_17332 | 1.88 |
| NM_145311.1 | CRYZL1 | ILMN_17071 | 1.97 |
| NM_007171.3 | POMT1 | ILMN_18145 | 1.9 |
| NM_001024070.1 | GCH1 | ILMN_23648 | 1.85 |
| NM_133328.2 | DEDD2 | ILMN_12562 | 1.89 |
| NM_018263.4 | ASXL2 | ILMN_7971 | 2.08 |
| NM_004036.3 | ADCY3 | ILMN_26929 | 2 |
| NM_153812.1 | PHF13 | ILMN_27355 | 2.05 |
| NM_001014979.1 | LOC90835 | ILMN_8821 | 1.9 |
| NM_006285.2 | TESK1 | ILMN_5444 | 1.9 |
| NM_017806.1 | LIME1 | ILMN_28410 | 1.94 |
| NM_007112.3 | THBS3 | ILMN_10000 | 1.84 |
| NM_022494.1 | ZDHHC6 | ILMN_1193 | 2.02 |
| NM_080730.2 | IFFO | ILMN_42149 | 1.98 |
| XM_926036.1 | LOC653103 | ILMN_32029 | 1.96 |
| NM_020836.2 | BEGAIN | ILMN_10503 | 1.93 |
| NM_012119.3 | CCRK | ILMN_39653 | 1.88 |
| NM_020246.2 | SLC12A9 | ILMN_12081 | 2.03 |
| NM_000271.3 | NPC1 | ILMN_30618 | 1.85 |
| NM_019024.1 | HEATR5B | ILMN_25274 | 1.85 |
| NM_024319.2 | C1orf35 | ILMN_28904 | 1.92 |
| NM_024804.1 | ZNF669 | ILMN_26142 | 1.87 |
| NM_014909.3 | VASH1 | ILMN_164012 | 1.83 |
| NM_024881.3 | SLC35E1 | ILMN_23168 | 1.94 |
| XM_944915.1 | PTP4A2 | ILMN_137656 | 1.85 |
| NM_001020820.1 | MYADM | ILMN_8340 | 2.01 |
| NM_001079514.1 | UBN1 | ILMN_172742 | 1.99 |
| NM_014984.2 | AZI1 | ILMN_3856 | 2.06 |
| NM_019119.3 | PCDHB9 | ILMN_23442 | 1.9 |
| NM_015318.2 | ARHGEF18 | ILMN_4153 | 1.98 |
| NM_145798.2 | OSBPL7 | ILMN_4611 | 1.88 |
| NM_003621.1 | PPFIBP2 | ILMN_183115 | 1.78 |
| NM_053274.2 | GLMN | ILMN_38827 | 1.89 |
| NM_004641.2 | MLLT10 | ILMN_25545 | 1.94 |
| NM_178324.1 | SPTLC1 | ILMN_7889 | 2.3 |
| NM_173614.2 | NOMO2 | ILMN_1736 | 2.02 |
| NM_017741.3 | C4orf30 | ILMN_172318 | 1.92 |
| NM_016284.3 | CNOT1 | ILMN_169268 | 2.02 |
| NM_052897.3 | MBD6 | ILMN_162772 | 1.88 |
| NM_001040428.2 | SPATA7 | ILMN_163094 | 1.8 |
| NM_015316.2 | PPP1R13B | ILMN_13872 | 1.73 |
| NM_001092.3 | ABR | ILMN_23502 | 2.05 |
| NM_198971.1 | MIZF | ILMN_28647 | 1.69 |
| NM_024910.1 | ZNF767 | ILMN_28810 | 1.76 |
| NM_020162.2 | DHX33 | ILMN_1191 | 1.91 |
| NM_003475.2 | RASSF7 | ILMN_12457 | 1.87 |
| NM_021168.2 | RAB40C | ILMN_22367 | 2.02 |
| NM_153350.2 | FBXL16 | ILMN_17900 | 1.92 |
| NM_144635.3 | FAM131A | ILMN_2542 | 1.92 |
| NM_014329.3 | EDC4 | ILMN_21643 | 2.01 |
| NM_006999.3 | POLS | ILMN_866 | 1.8 |
| NM_004779.4 | CNOT8 | ILMN_10063 | 1.76 |
| NM_001010927.2 | TIAM2 | ILMN_9891 | 1.72 |
| XM_290799.7 | ARHGAP23 | ILMN_162296 | 1.78 |
| NM_173666.1 | DTWD2 | ILMN_25915 | 1.98 |
| NM_001031712.2 | TRMT11 | ILMN_8801 | 1.87 |
| NM_032810.2 | ATAD1 | ILMN_175726 | 1.81 |
| XR_017538.1 | LOC653479 | ILMN_167529 | 1.88 |
| NM_138452.1 | DHRS1 | ILMN_15545 | 1.72 |
| NM_032239.2 | LARP2 | ILMN_9962 | 1.69 |
| NM_004428.2 | EFNA1 | ILMN_14320 | 1.89 |
| NM_006426.1 | DPYSL4 | ILMN_175746 | 2.18 |
| NM_015001.2 | SPEN | ILMN_180751 | 1.91 |
| NM_030665.3 | RAI1 | ILMN_176671 | 1.82 |
| NM_033446.1 | FAM125B | ILMN_20760 | 1.78 |
| NM_002482.2 | NASP | ILMN_21654 | 1.93 |
| NM_019024.1 | HEATR5B | ILMN_183109 | 1.85 |
| NM_003846.1 | PEX11B | ILMN_20603 | 1.93 |
| NM_007171.2 | POMT1 | ILMN_18145 | 1.74 |
| NM_001280.1 | CIRBP | ILMN_24327 | 2.22 |
| NM_032017.1 | STK40 | ILMN_25410 | 1.89 |
| NM_003166.3 | SULT1A3 | ILMN_28760 | 1.73 |
| NM_020695.3 | REXO1 | ILMN_20923 | 1.61 |
| NM_001080485.1 | ZNF275 | ILMN_180340 | 1.77 |
| NM_001034194.1 | EXOSC9 | ILMN_26957 | 1.7 |
| NM_024884.1 | L2HGDH | ILMN_21427 | 1.66 |
| NM_002451.3 | MTAP | ILMN_163674 | 1.85 |
| NM_015477.1 | SIN3A | ILMN_14108 | 1.89 |
| NM_014811.3 | KIAA0649 | ILMN_9360 | 1.67 |
| NM_001039705.1 | TRO | ILMN_32618 | 1.96 |
| NM_001002878.1 | THOC5 | ILMN_13820 | 1.98 |
| NM_015500.1 | C2CD2 | ILMN_182120 | 1.71 |
| NM_020789.2 | IGSF9 | ILMN_22415 | 1.74 |
| NM_177536.1 | SULT1A1 | ILMN_29763 | 1.64 |
| NM_199126.1 | ZNF585A | ILMN_9003 | 1.68 |
| NM_019591.2 | ZNF26 | ILMN_3233 | 1.75 |
| NM_015085.3 | GARNL4 | ILMN_163593 | 1.93 |
| NM_001567.2 | INPPL1 | ILMN_20903 | 1.86 |
| NM_005177.3 | ATP6V0A1 | ILMN_28612 | 1.93 |
| NM_012162.1 | FBXL6 | ILMN_5340 | 1.68 |
| NM_024077.3 | SECISBP2 | ILMN_19156 | 1.74 |
| NM_173647.2 | RNF149 | ILMN_10320 | 1.79 |
| NM_002076.2 | GNS | ILMN_177670 | 2.01 |
| NM_007200.3 | AKAP13 | ILMN_28017 | 1.74 |
| NM_007144.2 | PCGF2 | ILMN_11878 | 1.85 |
| NM_001031617.2 | COX19 | ILMN_15655 | 1.71 |
| NM_013276.2 | SHPK | ILMN_22706 | 1.73 |
| NM_201281.1 | MTMR2 | ILMN_24002 | 1.65 |
| NM_005560.3 | LAMA5 | ILMN_12588 | 1.91 |
| NM_015711.2 | GLTSCR1 | ILMN_18273 | 1.64 |
| XM_001132495.1 | SLC26A11 | ILMN_167531 | 1.71 |
| NM_152280.2 | SYT11 | ILMN_23967 | 2.15 |
| NM_005089.1 | U2AF1L2 | ILMN_13409 | 1.69 |
| NM_001001132.1 | ITSN1 | ILMN_10040 | 1.76 |
| XM_942991.2 | LOC642934 | ILMN_39429 | 1.85 |
| NM_014023.3 | WDR37 | ILMN_175566 | 1.69 |
| NM_145687.2 | MAP4K4 | ILMN_28871 | 1.77 |
| NM_001621.2 | AHR | ILMN_138365 | 2.1 |
| NM_001002878.1 | THOC5 | ILMN_13820 | 1.91 |
| NM_024561.3 | NARG1L | ILMN_22547 | 1.61 |
| NM_032421.2 | CLIP2 | ILMN_14847 | 1.67 |
| NM_014141.4 | CNTNAP2 | ILMN_176606 | 1.68 |
| NM_016577.3 | RAB6B | ILMN_177099 | 1.61 |
| NM_019892.3 | INPP5E | ILMN_11866 | 1.77 |
| NM_173518.2 | C8orf45 | ILMN_22241 | 2.01 |
| NM_001761.1 | CCNF | ILMN_27253 | 1.82 |
| NM_001013845.1 | CXorf40B | ILMN_170421 | 1.61 |
| NM_014700.2 | RAB11FIP3 | ILMN_7754 | 1.77 |
| NM_013241.2 | FHOD1 | ILMN_14837 | 1.68 |
| NM_005088.2 | SFRS17A | ILMN_26209 | 1.84 |
| NM_022451.9 | NOC3L | ILMN_11360 | 1.92 |
| NM_001040101.1 | D4S234E | ILMN_173747 | 1.95 |
| NM_001013649.1 | LOC388969 | ILMN_138621 | 1.61 |
| NM_003342.4 | UBE2G1 | ILMN_179729 | 1.68 |
| NM_016437.1 | TUBG2 | ILMN_13533 | 1.59 |
| NM_173546.1 | KLHDC8B | ILMN_6513 | 1.8 |
| NM_003666.2 | BLZF1 | ILMN_21927 | 2.02 |
| NR_003659.1 | FAM39DP | ILMN_307683 | 1.83 |
| NM_005819.4 | STX6 | ILMN_180926 | 1.66 |
| NM_133371.2 | MYOZ3 | ILMN_21305 | 1.66 |
| NM_001406.3 | EFNB3 | ILMN_17706 | 1.87 |
| NM_153321.1 | PMP22 | ILMN_9212 | 1.59 |
| NM_175859.1 | CTPS2 | ILMN_8874 | 1.84 |
| NM_014727.1 | MLL4 | ILMN_28047 | 1.57 |
| NM_024735.2 | FBXO31 | ILMN_17806 | 1.58 |
| XM_001127981.1 | LOC728014 | ILMN_169164 | 1.57 |
| NM_017412.2 | FZD3 | ILMN_18644 | 1.62 |
| NM_004055.4 | CAPN5 | ILMN_30845 | 1.6 |
| NM_015327.1 | SMG5 | ILMN_10815 | 1.63 |
| NM_001032293.2 | ZNF207 | ILMN_21705 | 1.87 |
| NM_005207.2 | CRKL | ILMN_165503 | 1.87 |
| NM_013229.2 | APAF1 | ILMN_29517 | 1.62 |
| XM_926231.1 | P704P | ILMN_36679 | 1.59 |
| NM_002048.1 | GAS1 | ILMN_175833 | 1.68 |
| NM_015902.4 | UBR5 | ILMN_178959 | 1.56 |
| XM_495939.3 | KIAA1545 | ILMN_40920 | 1.51 |
| NM_020850.1 | RANBP10 | ILMN_21091 | 1.53 |
| NM_033419.3 | PERLD1 | ILMN_12215 | 1.54 |
| NM_024046.3 | CAMKV | ILMN_24127 | 1.82 |
| NR_003659.1 | FAM39DP | ILMN_307683 | 1.92 |
| NM_032620.1 | GTPBP3 | ILMN_13264 | 1.8 |
| NM_014701.2 | KIAA0256 | ILMN_23132 | 1.61 |
| NM_017775.2 | TTC19 | ILMN_26390 | 1.73 |
| NM_001024071.1 | GCH1 | ILMN_14690 | 1.64 |
| NM_000743.2 | CHRNA3 | ILMN_23268 | 1.68 |
| NM_139353.1 | TAF1C | ILMN_4122 | 1.83 |
| NM_014689.2 | DOCK10 | ILMN_15188 | 1.69 |
| NM_030954.2 | RNF170 | ILMN_24753 | 1.54 |
| NM_032883.1 | C20orf100 | ILMN_17741 | 1.79 |
| NM_148957.2 | TNFRSF19 | ILMN_28684 | 1.55 |
| NM_001083946.1 | C2orf56 | ILMN_307130 | 1.68 |
| NM_014747.2 | RIMS3 | ILMN_21581 | 1.79 |
| NM_152666.1 | PLD5 | ILMN_25636 | 1.52 |
| NM_207644.2 | C22orf36 | ILMN_169188 | 1.54 |
| NM_003185.3 | TAF4 | ILMN_167910 | 1.61 |
| NM_018246.2 | CCDC25 | ILMN_5229 | 1.79 |
| NM_017566.2 | KLHDC4 | ILMN_8527 | 1.5 |
| NM_005342.2 | HMGB3 | ILMN_8326 | 1.69 |
| NM_006148.1 | LASP1 | ILMN_27039 | 1.92 |
| NM_002293.2 | LAMC1 | ILMN_182622 | 1.81 |
| NM_013243.2 | SCG3 | ILMN_174345 | 1.61 |
| NM_014681.4 | DHX34 | ILMN_30046 | 1.52 |
| NM_174891.3 | C14orf79 | ILMN_22555 | 1.55 |
| NM_031263.1 | HNRPK | ILMN_16515 | 1.99 |
| NM_032753.2 | RAXL1 | ILMN_21452 | 1.58 |
| NM_022497.3 | MRPS25 | ILMN_20500 | 1.54 |
| NM_004566.2 | PFKFB3 | ILMN_163833 | 1.57 |
| NM_016841.2 | MAPT | ILMN_3284 | 1.58 |
| NM_021070.2 | LTBP3 | ILMN_918 | 1.58 |
| NM_015636.3 | EIF2B4 | ILMN_18552 | 1.81 |
| NM_033426.2 | KIAA1737 | ILMN_24671 | 1.54 |
| NM_152716.1 | PATL1 | ILMN_11588 | 1.68 |
| NM_018097.1 | CEP27 | ILMN_15131 | 1.64 |
| NM_015213.2 | RAB6IP1 | ILMN_24051 | 1.76 |
| NM_172358.1 | CD46 | ILMN_4413 | 1.7 |
| NM_015655.2 | ZNF337 | ILMN_3280 | 1.63 |
| NM_152424.1 | FLJ39827 | ILMN_19358 | 1.52 |
| NM_002982.3 | CCL2 | ILMN_25185 | 1.94 |
| XR_015889.1 | LOC728888 | ILMN_170249 | 1.91 |
| NM_024792.1 | FAM57A | ILMN_28644 | 1.59 |
| NM_032530.1 | ZNF594 | ILMN_309021 | 1.55 |
| NM_177972.1 | TUB | ILMN_11520 | 1.56 |
| NM_024612.3 | DHX40 | ILMN_1864 | 1.58 |
| NM_004698.1 | PRPF3 | ILMN_6388 | 1.73 |
| NM_139179.1 | DAGLB | ILMN_1764 | 1.56 |
| NM_002230.1 | JUP | ILMN_3789 | 1.64 |
| NM_145648.1 | SLC15A4 | ILMN_2279 | 1.56 |
| NM_018054.4 | ARHGAP17 | ILMN_9156 | 1.7 |
| NM_017850.1 | C1orf109 | ILMN_27592 | 1.58 |
| NM_014708.3 | KNTC1 | ILMN_25890 | 1.61 |
| NM_032520.3 | GNPTG | ILMN_28173 | 1.57 |
| NM_006392.2 | NOL5A | ILMN_13841 | 1.66 |
| NM_022662.2 | ANAPC1 | ILMN_164277 | 1.58 |
| NM_006465.2 | ARID3B | ILMN_4032 | 1.53 |
| NM_005227.2 | EFNA4 | ILMN_18185 | 1.56 |
| NM_138687.1 | PIP5K2B | ILMN_12735 | 1.82 |
| NM_182919.1 | TICAM1 | ILMN_11434 | 1.53 |
| NM_005766.2 | FARP1 | ILMN_15608 | 1.62 |
| NM_023080.1 | C8orf33 | ILMN_15901 | 1.61 |
| NM_003861.1 | WDR22 | ILMN_22204 | 1.51 |
| NM_032853.2 | MUM1 | ILMN_162947 | 1.59 |
| NM_001012516.1 | ITM2C | ILMN_27531 | 1.82 |
| NM_006083.3 | IK | ILMN_27338 | 1.66 |
| NM_032285.2 | MGC3207 | ILMN_3158 | 1.53 |
| NM_207577.1 | MAP6 | ILMN_5510 | 1.54 |
| NM_201553.1 | FGL1 | ILMN_2104 | 1.61 |
| XM_934113.1 | LOC653489 | ILMN_42664 | 1.68 |
| NR_003264.1 | SDHALP1 | ILMN_175200 | 1.83 |
| NM_021136.2 | RTN1 | ILMN_174587 | 1.87 |
| NM_032431.2 | SYVN1 | ILMN_10424 | 1.52 |
| NM_015330.1 | SPECC1L | ILMN_168707 | 1.62 |
| NM_003565.1 | ULK1 | ILMN_2158 | 1.7 |
| NM_007271.2 | STK38 | ILMN_8385 | 1.5 |
| NM_006731.2 | FKTN | ILMN_6512 | 1.96 |
| NM_030918.5 | SNX27 | ILMN_17828 | 1.63 |
| NM_005964.1 | MYH10 | ILMN_23305 | 1.81 |
| NM_001287.3 | CLCN7 | ILMN_8600 | 1.68 |
| NM_006372.3 | SYNCRIP | ILMN_28470 | 1.6 |
| NM_016333.2 | SRRM2 | ILMN_21088 | 1.79 |
| NM_003799.1 | RNMT | ILMN_23400 | 1.51 |
| NM_019609.3 | CPXM1 | ILMN_26242 | 1.52 |
| NM_032830.1 | CIRH1A | ILMN_2574 | 1.63 |
| NM_004332.1 | BPHL | ILMN_27041 | 1.58 |
| NM_020724.1 | RNF150 | ILMN_26801 | 1.71 |
| NM_000319.3 | PEX5 | ILMN_29393 | 1.58 |
| NM_021239.1 | RBM25 | ILMN_18687 | 1.59 |
| NM_020728.1 | FAM62B | ILMN_19173 | 1.61 |
| NM_079837.2 | BANP | ILMN_8638 | 1.64 |
| NM_172249.1 | CSF2RA | ILMN_5061 | 1.59 |
| NM_145645.2 | NSUN5B | ILMN_32951 | 1.51 |
| NM_001032293.2 | ZNF207 | ILMN_21705 | 1.56 |
| NM_002688.4 | SEPT5 | ILMN_38941 | 1.58 |
| NM_002650.1 | PIK4CA | ILMN_20581 | 1.51 |
| NM_006903.4 | PPA2 | ILMN_15173 | 1.71 |
| NM_005506.2 | SCARB2 | ILMN_12802 | 1.73 |
| NM_001013839.1 | EXOC7 | ILMN_25212 | 1.57 |
| NM_001003.2 | RPLP1 | ILMN_23181 | 1.69 |
| NM_015908.4 | ARS2 | ILMN_19647 | 1.68 |
| NM_053000.1 | TIGA1 | ILMN_2113 | 1.63 |
| NM_024612.3 | DHX40 | ILMN_1864 | 1.57 |
| NM_013233.2 | STK39 | ILMN_19845 | 1.57 |
| NM_014765.1 | TOMM20 | ILMN_20433 | 1.78 |
| NM_001275.3 | CHGA | ILMN_23390 | 1.82 |
| NM_013373.2 | ZDHHC8 | ILMN_29624 | 1.57 |
| NM_025161.3 | C17orf70 | ILMN_178717 | 1.53 |
| NM_015878.4 | AZIN1 | ILMN_4825 | 1.53 |
| NM_033412.1 | MCART1 | ILMN_22327 | 1.73 |
| NM_020215.2 | C14orf132 | ILMN_29055 | 1.56 |
| NM_032794.1 | SLC44A4 | ILMN_14709 | 1.76 |
| NM_001614.2 | ACTG1 | ILMN_24353 | 1.87 |
| NM_000787.3 | DBH | ILMN_25962 | 1.73 |
| NM_138477.2 | CDAN1 | ILMN_168162 | 1.73 |
| NM_022483.3 | C5orf28 | ILMN_5037 | 1.58 |
| NM_032308.1 | RPAIN | ILMN_15409 | 1.54 |
| NM_001632.3 | ALPP | ILMN_25184 | 1.52 |
| NM_017833.2 | C21orf55 | ILMN_6782 | 1.64 |
| NM_006567.3 | FARS2 | ILMN_19613 | -1.51 |
| NM_139283.1 | PPTC7 | ILMN_11800 | -1.56 |
| NM_021242.4 | MID1IP1 | ILMN_161908 | -1.54 |
| NM_002154.3 | HSPA4 | ILMN_166427 | -1.6 |
| NM_020918.3 | GPAM | ILMN_174762 | -1.51 |
| NM_080605.3 | B3GALT6 | ILMN_170784 | -1.58 |
| NM_022370.2 | ROBO3 | ILMN_24988 | -1.52 |
| NM_020195.1 | C14orf124 | ILMN_4144 | -1.59 |
| NM_016002.2 | SCCPDH | ILMN_30353 | -1.57 |
| NM_080653.3 | ATP6V1E2 | ILMN_5551 | -1.52 |
| NM_004117.2 | FKBP5 | ILMN_16562 | -1.54 |
| NM_020310.2 | MNT | ILMN_21283 | -1.59 |
| NM_000615.5 | NCAM1 | ILMN_7059 | -1.57 |
| NM_015407.3 | ABHD14A | ILMN_29464 | -1.54 |
| NM_003656.3 | CAMK1 | ILMN_21373 | -1.62 |
| XM_927071.2 | LOC643790 | ILMN_38875 | -1.58 |
| NM_004982.2 | KCNJ8 | ILMN_29993 | -1.56 |
| XM_930884.1 | LOC653080 | ILMN_32261 | -1.54 |
| NM_004378.1 | CRABP1 | ILMN_12739 | -1.57 |
| NM_199192.1 | BRE | ILMN_15533 | -1.54 |
| NM_000022.2 | ADA | ILMN_8067 | -1.67 |
| NM_018246.2 | CCDC25 | ILMN_5229 | -1.6 |
| NM_021971.1 | GMPPB | ILMN_3929 | -1.61 |
| NM_016323.2 | HERC5 | ILMN_22093 | -1.61 |
| NM_032138.3 | KBTBD7 | ILMN_181309 | -1.67 |
| NM_016183.3 | MRTO4 | ILMN_1930 | -1.58 |
| NM_006455.2 | SC65 | ILMN_21605 | -1.58 |
| NM_024104.3 | C19orf42 | ILMN_26408 | -1.55 |
| NM_000284.1 | PDHA1 | ILMN_9293 | -1.57 |
| XM_936215.1 | LOC653874 | ILMN_35327 | -1.6 |
| NM_005552.4 | KLC1 | ILMN_22293 | -1.54 |
| XM_173119.5 | LOC255130 | ILMN_42241 | -1.57 |
| NM_001010897.1 | SERP2 | ILMN_13346 | -1.56 |
| NM_000288.1 | PEX7 | ILMN_25066 | -1.72 |
| NM_001365.2 | DLG4 | ILMN_164548 | -1.65 |
| NM_017653.2 | DYM | ILMN_6843 | -1.56 |
| NM_024684.2 | C11orf67 | ILMN_26165 | -1.55 |
| NM_001024218.1 | GPHN | ILMN_29329 | -1.67 |
| NM_003805.3 | CRADD | ILMN_12877 | -1.62 |
| NM_032928.2 | TMEM141 | ILMN_27026 | -1.74 |
| NM_007236.3 | CHP | ILMN_23083 | -1.7 |
| NM_199295.1 | APITD1 | ILMN_19061 | -1.63 |
| NM_018304.2 | PRR11 | ILMN_32619 | -1.59 |
| NM_199287.2 | CCDC137 | ILMN_309720 | -1.73 |
| NM_007358.2 | MTF2 | ILMN_24749 | -1.59 |
| NM_000485.2 | APRT | ILMN_4221 | -1.55 |
| NM_002643.3 | PIGF | ILMN_15261 | -1.52 |
| NM_016448.1 | DTL | ILMN_29702 | -1.64 |
| NM_024955.4 | FOXRED2 | ILMN_165686 | -1.62 |
| NM_017843.3 | BCAS4 | ILMN_21706 | -1.74 |
| NM_153768.1 | CABYR | ILMN_9439 | -1.64 |
| NM_001042401.1 | C21orf51 | ILMN_179828 | -1.76 |
| NM_001008783.1 | SLC35D3 | ILMN_16642 | -1.72 |
| NM_022768.4 | RBM15 | ILMN_1342 | -1.76 |
| NM_002045.2 | GAP43 | ILMN_28511 | -1.61 |
| NM_001014832.1 | PAK4 | ILMN_24614 | -1.7 |
| NM_005388.3 | PDCL | ILMN_34020 | -1.8 |
| NM_003309.2 | TSPYL1 | ILMN_29963 | -1.71 |
| NM_015696.3 | GPX7 | ILMN_164820 | -1.6 |
| NM_198897.1 | FIBP | ILMN_8196 | -1.69 |
| NM_174908.2 | CCDC50 | ILMN_12155 | -1.63 |
| NM_024321.3 | RBM42 | ILMN_182570 | -1.68 |
| NM_005573.2 | LMNB1 | ILMN_4100 | -1.59 |
| XM_929420.1 | LOC653377 | ILMN_43949 | -1.5 |
| NM_006310.2 | NPEPPS | ILMN_184074 | -1.62 |
| NM_001002836.2 | ZNF787 | ILMN_24445 | -1.58 |
| NM_052848.1 | CCDC97 | ILMN_24401 | -1.75 |
| NM_001035513.1 | SDHC | ILMN_14364 | -1.71 |
| NM_017970.2 | C14orf102 | ILMN_22111 | -1.75 |
| NM_001017392.2 | SFRS14 | ILMN_17110 | -1.65 |
| NM_001009608.1 | C20orf94 | ILMN_24801 | -1.8 |
| NM_006769.2 | LMO4 | ILMN_183982 | -1.78 |
| NM_182513.1 | SPC24 | ILMN_174688 | -1.65 |
| NM_024640.3 | YRDC | ILMN_2794 | -1.54 |
| NM_022135.2 | POPDC2 | ILMN_17743 | -1.71 |
| NM_018466.3 | ALG13 | ILMN_3125 | -1.66 |
| NM_181042.2 | PBRM1 | ILMN_16253 | -1.8 |
| NM_080546.3 | SLC44A1 | ILMN_23525 | -1.6 |
| NM_000281.2 | PCBD1 | ILMN_2950 | -1.56 |
| NM_001031677.2 | RAB24 | ILMN_25731 | -1.75 |
| NM_021004.2 | DHRS4 | ILMN_10152 | -1.63 |
| NM_002225.2 | IVD | ILMN_13293 | -1.78 |
| NM_198047.1 | HIBCH | ILMN_24888 | -1.7 |
| XR_015310.1 | LOC643438 | ILMN_165861 | -1.72 |
| NM_018844.2 | BCAP29 | ILMN_24686 | -1.77 |
| NM_201414.1 | APP | ILMN_23272 | -1.57 |
| NM_001321.1 | CSRP2 | ILMN_3862 | -1.67 |
| NM_005996.3 | TBX3 | ILMN_23090 | -1.77 |
| NM_000905.2 | NPY | ILMN_11990 | -1.7 |
| NM_001005368.1 | ZNF32 | ILMN_181781 | -1.75 |
| NM_020865.1 | DHX36 | ILMN_11905 | -1.69 |
| NM_015980.3 | HMP19 | ILMN_1495 | -1.55 |
| NM_001827.1 | CKS2 | ILMN_14702 | -1.54 |
| NM_006117.2 | PECI | ILMN_7427 | -1.68 |
| NM_024292.2 | UBL5 | ILMN_14261 | -1.76 |
| NM_001382.2 | DPAGT1 | ILMN_10306 | -1.81 |
| NM_017971.2 | MRPL20 | ILMN_859 | -1.63 |
| NM_002487.2 | NDN | ILMN_23775 | -1.85 |
| NM_001080546.1 | LOC219854 | ILMN_168339 | -1.74 |
| NM_023936.1 | MRPS34 | ILMN_5723 | -1.79 |
| NM_018840.2 | C20orf24 | ILMN_10676 | -1.53 |
| NM_001037633.1 | SIL1 | ILMN_10838 | -1.82 |
| NM_006854.3 | KDELR2 | ILMN_1810 | -1.67 |
| NM_020967.2 | NCOA5 | ILMN_182789 | -1.7 |
| NM_005165.2 | ALDOC | ILMN_15767 | -1.58 |
| NM_022767.2 | ISG20L1 | ILMN_12401 | -1.71 |
| NM_153824.1 | PYCR1 | ILMN_8761 | -1.75 |
| NM_001359.1 | DECR1 | ILMN_11646 | -1.75 |
| NM_032829.1 | C12orf34 | ILMN_22158 | -1.84 |
| XM_934410.1 | LOC643995 | ILMN_31166 | -1.82 |
| NM_005850.3 | SF3B4 | ILMN_12460 | -1.51 |
| NM_014236.2 | GNPAT | ILMN_175916 | -1.62 |
| NM_005749.2 | TOB1 | ILMN_13735 | -1.76 |
| NM_006807.3 | CBX1 | ILMN_162583 | -1.67 |
| NM_022549.2 | FEZ1 | ILMN_419 | -1.87 |
| NM_001008709.1 | PPP1CA | ILMN_29100 | -1.57 |
| NM_180981.1 | MRPL52 | ILMN_3474 | -1.69 |
| NM_000156.4 | GAMT | ILMN_20028 | -1.66 |
| NM_001031835.1 | PHKB | ILMN_18544 | -1.79 |
| NM_000414.1 | HSD17B4 | ILMN_23623 | -1.64 |
| NM_004792.2 | PPIG | ILMN_24595 | -1.86 |
| NM_001037537.1 | PHYH | ILMN_17762 | -1.7 |
| NM_031314.1 | HNRPC | ILMN_24356 | -1.76 |
| NM_014176.2 | UBE2T | ILMN_9573 | -1.6 |
| NM_004885.1 | NPFFR2 | ILMN_20676 | -1.82 |
| NM_005803.2 | FLOT1 | ILMN_18913 | -1.71 |
| NM_014063.5 | DBNL | ILMN_3068 | -1.76 |
| NM_004048.2 | B2M | ILMN_19648 | -1.52 |
| NM_016142.1 | HSD17B12 | ILMN_19305 | -1.58 |
| NM_001914.2 | CYB5A | ILMN_25182 | -1.77 |
| NM_017526.2 | LEPROT | ILMN_27032 | -1.69 |
| NM_012111.1 | AHSA1 | ILMN_11051 | -1.61 |
| NM_006630.1 | ZNF234 | ILMN_29233 | -1.85 |
| NM_032772.3 | ZNF503 | ILMN_2048 | -1.8 |
| NM_001078651.1 | TMEM134 | ILMN_176754 | -1.75 |
| NM_207118.1 | GTF2H5 | ILMN_26206 | -1.61 |
| NM_004148.3 | NINJ1 | ILMN_21540 | -1.78 |
| NM_145080.3 | NSMCE1 | ILMN_27090 | -1.73 |
| NM_184234.1 | RBM39 | ILMN_20330 | -1.77 |
| NM_152912.3 | MTIF3 | ILMN_16655 | -1.81 |
| NM_001071.1 | TYMS | ILMN_26899 | -1.52 |
| NM_005952.2 | MT1X | ILMN_16629 | -1.73 |
| NM_000701.6 | ATP1A1 | ILMN_677 | -1.71 |
| NM_138794.2 | LYPLAL1 | ILMN_25005 | -1.86 |
| NM_014170.2 | GTPBP8 | ILMN_27163 | -1.74 |
| NM_012482.3 | ZNF281 | ILMN_18970 | -1.64 |
| NM_014847.2 | UBAP2L | ILMN_163836 | -1.79 |
| NM_032747.2 | USMG5 | ILMN_10409 | -1.85 |
| NM_012446.2 | SSBP2 | ILMN_5320 | -1.91 |
| NM_000814.4 | GABRB3 | ILMN_19294 | -1.7 |
| NM_012117.1 | CBX5 | ILMN_25072 | -1.62 |
| NM_005326.4 | HAGH | ILMN_22401 | -1.62 |
| XM_944786.1 | LOC650737 | ILMN_40280 | -1.73 |
| NM_001001586.1 | ATP1A1 | ILMN_2141 | -1.67 |
| NM_004470.2 | FKBP2 | ILMN_17464 | -1.75 |
| NM_014183.2 | DYNLRB1 | ILMN_6713 | -1.75 |
| NM_013299.3 | SAC3D1 | ILMN_9385 | -1.77 |
| NM_005238.2 | ETS1 | ILMN_173009 | -1.89 |
| NM_020158.3 | EXOSC5 | ILMN_6934 | -1.78 |
| NM_031298.2 | TMEM93 | ILMN_9888 | -1.69 |
| NM_002157.1 | HSPE1 | ILMN_2612 | -1.64 |
| NM_022893.2 | BCL11A | ILMN_17359 | -1.95 |
| NM_024540.2 | MRPL24 | ILMN_29128 | -1.81 |
| NM_018079.3 | SRBD1 | ILMN_28720 | -1.88 |
| NM_002491.1 | NDUFB3 | ILMN_22320 | -1.53 |
| NM_000476.1 | AK1 | ILMN_16785 | -1.99 |
| NM_032907.3 | UBL7 | ILMN_17890 | -1.9 |
| NM_000175.2 | GPI | ILMN_7872 | -1.82 |
| NM_016644.1 | PRR16 | ILMN_4368 | -1.81 |
| NM_007100.2 | ATP5I | ILMN_14284 | -1.83 |
| NM_014463.1 | LSM3 | ILMN_23516 | -1.58 |
| NM_053050.2 | MRPL53 | ILMN_25576 | -1.82 |
| NM_001382.2 | DPAGT1 | ILMN_10306 | -1.8 |
| NM_003583.3 | DYRK2 | ILMN_3688 | -1.9 |
| NM_015983.2 | UBE2D4 | ILMN_18182 | -1.83 |
| NM_153682.2 | PIGP | ILMN_18625 | -1.93 |
| NM_017996.2 | DET1 | ILMN_164954 | -2.01 |
| NM_017915.2 | C12orf48 | ILMN_42497 | -1.85 |
| NM_182810.1 | ATF4 | ILMN_23435 | -2.01 |
| NM_015480.1 | PVRL3 | ILMN_2284 | -2.01 |
| NM_001017963.1 | HSP90AA1 | ILMN_16669 | -2.02 |
| NM_012241.2 | SIRT5 | ILMN_18454 | -2.03 |
| NM_152789.2 | FAM133B | ILMN_1247 | -1.83 |
| NM_001008800.1 | CCT3 | ILMN_24878 | -1.76 |
| NM_000113.2 | TOR1A | ILMN_162390 | -1.93 |
| NM_005589.2 | ALDH6A1 | ILMN_24260 | -1.9 |
| NM_001168.2 | BIRC5 | ILMN_20443 | -1.77 |
| NM_014298.3 | QPRT | ILMN_21680 | -1.7 |
| NM_145274.2 | TMEM99 | ILMN_25105 | -2 |
| NM_001040668.1 | BCL2L12 | ILMN_177176 | -1.92 |
| NM_018718.1 | TSGA14 | ILMN_11000 | -1.84 |
| NM_018464.2 | CISD1 | ILMN_4843 | -1.76 |
| NM_001040139.1 | CKLF | ILMN_162861 | -1.81 |
| NM_024027.3 | COLEC11 | ILMN_6548 | -1.76 |
| NM_017823.3 | DUSP23 | ILMN_6272 | -1.71 |
| NM_013354.5 | CNOT7 | ILMN_7214 | -1.79 |
| NM_005733.1 | KIF20A | ILMN_14100 | -1.79 |
| NM_001878.2 | CRABP2 | ILMN_16252 | -1.89 |
| NM_000820.1 | GAS6 | ILMN_10723 | -1.88 |
| NM_003258.2 | TK1 | ILMN_30154 | -1.89 |
| NM_000314.4 | PTEN | ILMN_181706 | -1.75 |
| NM_201434.1 | RAB5C | ILMN_176672 | -1.9 |
| NM_005032.3 | PLS3 | ILMN_1428 | -1.93 |
| NM_003860.2 | BANF1 | ILMN_13154 | -1.98 |
| NM_004853.1 | STX8 | ILMN_9761 | -1.98 |
| NM_000101.2 | CYBA | ILMN_6945 | -1.85 |
| NM_181876.2 | PPP2R2C | ILMN_15268 | -2.15 |
| NM_016071.2 | MRPS33 | ILMN_4243 | -1.65 |
| NM_001039703.1 | NBPF10 | ILMN_45673 | -1.55 |
| XM_930694.1 | LOC642477 | ILMN_36253 | -1.78 |
| NM_005371.4 | METTL1 | ILMN_178633 | -2.1 |
| NM_003011.2 | SET | ILMN_180677 | -1.69 |
| NM_000051.3 | ATM | ILMN_162851 | -1.98 |
| NM_014913.2 | ADNP2 | ILMN_6906 | -1.9 |
| NM_004763.3 | ITGB1BP1 | ILMN_17602 | -1.96 |
| NM_002712.1 | PPP1R7 | ILMN_29559 | -2.04 |
| NM_000389.2 | CDKN1A | ILMN_16780 | -1.65 |
| NM_003689.2 | AKR7A2 | ILMN_182370 | -1.8 |
| NM_153201.1 | HSPA8 | ILMN_14829 | -1.5 |
| NM_002338.2 | LSAMP | ILMN_861 | -2.02 |
| NM_001040056.1 | MAPK3 | ILMN_177323 | -2.01 |
| NM_003358.1 | UGCG | ILMN_26228 | -2.06 |
| NR_003105.1 | ZWILCH | ILMN_166966 | -1.77 |
| NM_001077446.1 | TSEN34 | ILMN_177054 | -1.79 |
| NM_024051.2 | C7orf24 | ILMN_2391 | -2.07 |
| NM_018473.2 | THEM2 | ILMN_27212 | -2 |
| NM_021244.3 | RRAGD | ILMN_5663 | -2.09 |
| NM_001826.1 | CKS1B | ILMN_11313 | -1.69 |
| NM_002923.1 | RGS2 | ILMN_26119 | -1.96 |
| NM_001018109.1 | PIR | ILMN_13999 | -1.96 |
| NM_002491.1 | NDUFB3 | ILMN_22320 | -1.68 |
| NM_007155.4 | ZP3 | ILMN_17555 | -2.05 |
| NM_018847.2 | KLHL9 | ILMN_20376 | -1.9 |
| NM_004111.4 | FEN1 | ILMN_162686 | -1.75 |
| NM_003924.2 | PHOX2B | ILMN_172224 | -1.64 |
| NM_172315.1 | MEIS2 | ILMN_17528 | -1.63 |
| NM_003916.3 | AP1S2 | ILMN_3812 | -1.81 |
| NM_018297.2 | NGLY1 | ILMN_15318 | -2.01 |
| NM_001014438.1 | CARS | ILMN_172747 | -1.98 |
| NM_025129.3 | FUZ | ILMN_24173 | -2.05 |
| NM_001009570.1 | CCT7 | ILMN_24972 | -1.62 |
| NM_032638.3 | GATA2 | ILMN_20021 | -1.88 |
| NM_021127.1 | PMAIP1 | ILMN_25637 | -2.1 |
| NM_000076.1 | CDKN1C | ILMN_20689 | -2.03 |
| NM_052844.3 | WDR34 | ILMN_25161 | -2 |
| NM_019071.2 | ING3 | ILMN_177083 | -1.95 |
| NM_006327.2 | TIMM23 | ILMN_22871 | -1.89 |
| NM_032476.2 | MRPS6 | ILMN_17239 | -1.76 |
| NM_015386.2 | COG4 | ILMN_28901 | -2.09 |
| NM_004901.2 | ENTPD4 | ILMN_19012 | -2.08 |
| NM_145644.1 | MRPL35 | ILMN_20736 | -1.99 |
| NM_003776.2 | MRPL40 | ILMN_21771 | -1.95 |
| NM_006452.3 | PAICS | ILMN_6032 | -1.7 |
| NM_207376.1 | LOC387882 | ILMN_23241 | -2.03 |
| NM_003746.1 | DNCL1 | ILMN_137049 | -1.69 |
| XM_934985.1 | LOC400879 | ILMN_31001 | -1.92 |
| NM_007198.2 | PROSC | ILMN_23472 | -2.1 |
| NM_032439.1 | PHYHIPL | ILMN_22045 | -1.92 |
| NM_021170.2 | HES4 | ILMN_18566 | -2.07 |
| NM_018718.1 | TSGA14 | ILMN_11000 | -2.12 |
| NM_016625.2 | RSRC1 | ILMN_14978 | -2.17 |
| NM_006515.1 | SETMAR | ILMN_17510 | -2.05 |
| NM_014933.2 | SEC31A | ILMN_23819 | -2.12 |
| NM_000156.4 | GAMT | ILMN_20028 | -1.95 |
| XR_019449.1 | LOC644422 | ILMN_166674 | -2.01 |
| NM_001031717.2 | CRELD1 | ILMN_14216 | -2.09 |
| XM_001126211.1 | LOC727761 | ILMN_162963 | -1.93 |
| NM_001002246.1 | ANAPC11 | ILMN_5565 | -2.11 |
| NM_212552.2 | BOLA3 | ILMN_28776 | -1.95 |
| NM_014026.3 | DCPS | ILMN_24626 | -2.22 |
| NM_016098.1 | BRP44L | ILMN_4349 | -1.96 |
| NM_182547.2 | TMED4 | ILMN_30359 | -1.93 |
| NM_181803.1 | UBE2C | ILMN_6398 | -1.8 |
| NM_000485.2 | APRT | ILMN_4221 | -1.98 |
| NM_024710.1 | ISOC2 | ILMN_27084 | -2.18 |
| NM_001031827.1 | BOLA2 | ILMN_4509 | -2.04 |
| NM_001123.2 | ADK | ILMN_4107 | -2.16 |
| NM_001007214.1 | CACYBP | ILMN_16795 | -2.23 |
| NM_138787.2 | C11orf74 | ILMN_16125 | -2.04 |
| NM_014241.3 | PTPLA | ILMN_24983 | -2.16 |
| NM_003362.2 | UNG | ILMN_21638 | -1.99 |
| NM_016098.1 | BRP44L | ILMN_4349 | -2.2 |
| NM_004615.2 | TSPAN7 | ILMN_20684 | -2.24 |
| NM_006703.2 | NUDT3 | ILMN_25244 | -2.05 |
| NM_014170.2 | GTPBP8 | ILMN_27163 | -2.2 |
| NM_001007793.1 | BUB3 | ILMN_5688 | -2.17 |
| NM_001013699.1 | LOC440093 | ILMN_19743 | -1.88 |
| NM_033402.3 | LRRCC1 | ILMN_15234 | -2.28 |
| XM_496446.3 | LOC440737 | ILMN_39347 | -1.75 |
| NM_002598.2 | PDCD2 | ILMN_5469 | -2.03 |
| NM_182972.2 | IRF2BP2 | ILMN_5645 | -2.16 |
| NM_001003793.1 | RBMS3 | ILMN_16411 | -2.23 |
| NM_153682.2 | PIGP | ILMN_18625 | -2.08 |
| NM_203390.2 | RBM12B | ILMN_174962 | -2.36 |
| NM_002413.3 | MGST2 | ILMN_8759 | -2.16 |
| XM_938497.2 | C6orf52 | ILMN_42173 | -2.3 |
| XM_001129423.1 | LOC729137 | ILMN_166772 | -2.22 |
| NM_020529.1 | NFKBIA | ILMN_6745 | -1.96 |
| NM_003333.3 | UBA52 | ILMN_27795 | -2.06 |
| NM_032368.3 | LZIC | ILMN_17648 | -2.26 |
| NM_014223.2 | NFYC | ILMN_5936 | -2.25 |
| NM_024056.2 | TMEM106C | ILMN_7003 | -1.95 |
| NM_005192.2 | CDKN3 | ILMN_4098 | -2.17 |
| NM_001079863.1 | DBI | ILMN_173500 | -1.92 |
| NM_002897.3 | RBMS1 | ILMN_18726 | -2.35 |
| NM_005103.3 | FEZ1 | ILMN_28992 | -2.1 |
| NM_005318.2 | H1F0 | ILMN_139403 | -2.04 |
| NM_002315.1 | LMO1 | ILMN_29972 | -2.2 |
| NM_001002876.1 | CENPM | ILMN_12351 | -2.27 |
| NM_002157.1 | HSPE1 | ILMN_2612 | -1.89 |
| NM_153333.2 | TCEAL8 | ILMN_12551 | -2.21 |
| NM_032340.2 | C6orf125 | ILMN_21424 | -2.07 |
| NM_001048197.1 | SNHG3-RCC1 | ILMN_167397 | -2.31 |
| NM_012261.2 | C20orf103 | ILMN_165304 | -1.99 |
| NM_144594.1 | GTSF1 | ILMN_17221 | -2.43 |
| NM_054014.1 | FKBP1A | ILMN_29213 | -2.14 |
| NM_001914.2 | CYB5A | ILMN_25182 | -2.33 |
| NM_138493.2 | C6orf129 | ILMN_24513 | -2.07 |
| XM_936240.1 | LOC653884 | ILMN_34094 | -2.17 |
| XM_001131304.1 | LOC728635 | ILMN_168315 | -2.4 |
| NM_138765.2 | BAX | ILMN_11763 | -2.1 |
| NM_030928.2 | CDT1 | ILMN_18895 | -2.32 |
| NM_001634.4 | AMD1 | ILMN_21529 | -2.27 |
| NM_002014.2 | FKBP4 | ILMN_9429 | -2.19 |
| NM_012133.2 | COPG2 | ILMN_23766 | -2.33 |
| NM_014865.2 | NCAPD2 | ILMN_26621 | -2.1 |
| NM_001487.1 | BLOC1S1 | ILMN_14526 | -2.19 |
| NM_005842.2 | SPRY2 | ILMN_19344 | -2.1 |
| NM_005327.2 | HADH | ILMN_13258 | -2.17 |
| NM_018983.3 | NOLA1 | ILMN_14204 | -2.36 |
| NM_181800.1 | UBE2C | ILMN_25999 | -1.96 |
| NM_004343.2 | CALR | ILMN_18909 | -2.35 |
| NM_018137.1 | PRMT6 | ILMN_29888 | -2.32 |
| NM_024804.1 | ZNF669 | ILMN_26142 | -2.62 |
| NM_057159.2 | LPAR1 | ILMN_28278 | -2.39 |
| NM_005613.3 | RGS4 | ILMN_15378 | -2.04 |
| NM_001040138.1 | CKLF | ILMN_162781 | -2.17 |
| NM_174942.1 | GAS2L3 | ILMN_5609 | -2.49 |
| NM_005056.1 | JARID1A | ILMN_12150 | -2.25 |
| NM_138501.4 | GPSN2 | ILMN_6454 | -2.17 |
| NM_032356.3 | LSMD1 | ILMN_25444 | -2.29 |
| NM_001495.4 | GFRA2 | ILMN_24176 | -2.47 |
| NM_001889.2 | CRYZ | ILMN_30248 | -2.29 |
| NM_020116.2 | FSTL5 | ILMN_178729 | -2.46 |
| NM_004544.2 | NDUFA10 | ILMN_7463 | -2.39 |
| NM_014170.2 | GTPBP8 | ILMN_27163 | -2.47 |
| XM_498571.2 | LOC440160 | ILMN_33035 | -2.3 |
| NM_006761.3 | YWHAE | ILMN_18524 | -2.51 |
| NM_005680.1 | TAF1B | ILMN_13234 | -2.43 |
| NM_002086.3 | GRB2 | ILMN_173749 | -2.37 |
| NM_003404.3 | YWHAB | ILMN_17127 | -2.45 |
| NM_024333.1 | FSD1 | ILMN_13664 | -2.45 |
| NM_024122.2 | APOO | ILMN_11248 | -2.45 |
| NR_001449.1 | TRK1 | ILMN_6493 | -2.53 |
| NM_153026.1 | PRICKLE1 | ILMN_15149 | -2.26 |
| NM_005824.1 | LRRC17 | ILMN_162504 | -2.47 |
| NM_001842.3 | CNTFR | ILMN_21040 | -2.34 |
| NM_024296.3 | CCDC28B | ILMN_26263 | -2.45 |
| NM_181876.2 | PPP2R2C | ILMN_15268 | -2.48 |
| NM_002801.2 | PSMB10 | ILMN_29653 | -2.26 |
| NM_016086.2 | STYXL1 | ILMN_5068 | -2.38 |
| NM_016587.2 | CBX3 | ILMN_11642 | -2.2 |
| NM_022745.3 | ATPAF1 | ILMN_175478 | -2.45 |
| NM_001034841.2 | LOC162073 | ILMN_3559 | -2.41 |
| NM_198391.1 | FLRT3 | ILMN_23273 | -2.43 |
| NM_001540.2 | HSPB1 | ILMN_28967 | -2.09 |
| NM_024011.2 | CDC2L2 | ILMN_20434 | -2.49 |
| NM_004456.3 | EZH2 | ILMN_25740 | -2.48 |
| NM_013262.3 | MYLIP | ILMN_178445 | -2.52 |
| XM_001132569.1 | LOC730130 | ILMN_162537 | -2.4 |
| NM_033258.1 | GNG8 | ILMN_25463 | -2.49 |
| NM_032334.1 | C8orf53 | ILMN_24637 | -2.54 |
| NM_006191.2 | PA2G4 | ILMN_28541 | -2.41 |
| NM_001067.2 | TOP2A | ILMN_19849 | -2.24 |
| NM_152773.2 | TCTEX1D2 | ILMN_19950 | -2.52 |
| NM_001640.3 | APEH | ILMN_27694 | -2.4 |
| NM_006556.3 | PMVK | ILMN_165582 | -2.6 |
| NM_017917.2 | PPP2R3C | ILMN_23821 | -2.45 |
| NM_001124.1 | ADM | ILMN_29514 | -2.41 |
| NM_003211.3 | TDG | ILMN_29212 | -2.49 |
| NM_058246.3 | DNAJB6 | ILMN_7651 | -2.47 |
| NM_018837.2 | SULF2 | ILMN_18271 | -2.68 |
| NM_007280.1 | OIP5 | ILMN_18200 | -2.55 |
| NM_004616.2 | TSPAN8 | ILMN_578 | -2.49 |
| NM_178439.3 | GMCL1 | ILMN_3285 | -2.67 |
| NM_001031713.2 | CCDC90A | ILMN_9159 | -2.67 |
| NM_198954.1 | NUDT1 | ILMN_2361 | -2.68 |
| NM_016108.2 | AIG1 | ILMN_22004 | -2.7 |
| NM_001438.2 | ESRRG | ILMN_29221 | -2.68 |
| NM_145806.2 | ZNF511 | ILMN_15566 | -2.52 |
| NM_006429.2 | CCT7 | ILMN_22959 | -2.41 |
| NM_000856.3 | GUCY1A3 | ILMN_11680 | -2.65 |
| NM_017895.6 | DDX27 | ILMN_20732 | -2.66 |
| NM_002523.1 | NPTX2 | ILMN_22638 | -2.59 |
| NM_016048.1 | ISOC1 | ILMN_15311 | -2.4 |
| NM_138809.3 | CMBL | ILMN_1485 | -2.54 |
| NM_015609.2 | C1orf144 | ILMN_5836 | -2.7 |
| NM_080597.2 | OSBPL1A | ILMN_10951 | -2.66 |
| NR_003144.1 | LOC723972 | ILMN_180363 | -2.75 |
| NM_001283.2 | AP1S1 | ILMN_21653 | -2.48 |
| NM_012177.2 | FBXO5 | ILMN_9763 | -2.61 |
| NM_001333.2 | CTSL2 | ILMN_22377 | -2.76 |
| NM_005905.3 | SMAD9 | ILMN_28187 | -2.77 |
| NM_016937.2 | POLA1 | ILMN_181974 | -2.8 |
| NM_022903.3 | CCDC71 | ILMN_21600 | -2.84 |
| NM_004615.2 | TSPAN7 | ILMN_20684 | -2.81 |
| XM_930284.1 | LOC441763 | ILMN_36192 | -2.66 |
| NM_177983.1 | PPM1G | ILMN_878 | -2.9 |
| NM_022770.2 | GINS3 | ILMN_7033 | -2.97 |
| NM_005077.3 | TLE1 | ILMN_10669 | -2.85 |
| NM_002870.2 | RAB13 | ILMN_26464 | -2.8 |
| XM_942780.2 | SYNPO2 | ILMN_45907 | -2.8 |
| NM_001326.2 | CSTF3 | ILMN_27551 | -2.76 |
| NM_181332.1 | NLGN4X | ILMN_27075 | -3.03 |
| NM_017802.2 | HEATR2 | ILMN_1114 | -2.8 |
| XM_926249.2 | LOC642852 | ILMN_40586 | -2.91 |
| NM_003368.4 | USP1 | ILMN_5285 | -2.95 |
| NR_001445.1 | RN7SK | ILMN_14457 | -3.06 |
| NM_014169.2 | CHMP4A | ILMN_19959 | -2.9 |
| NM_181702.1 | GEM | ILMN_16170 | -2.9 |
| NM_006158.2 | NEFL | ILMN_22054 | -2.96 |
| NM_004316.2 | ASCL1 | ILMN_23892 | -2.75 |
| NM_014142.2 | NUDT5 | ILMN_1656 | -2.78 |
| NM_006324.2 | CFDP1 | ILMN_23508 | -2.95 |
| NM_057089.2 | AP1S1 | ILMN_4691 | -2.83 |
| NM_001040142.1 | SCN2A | ILMN_167124 | -2.97 |
| NM_002763.3 | PROX1 | ILMN_177185 | -2.75 |
| NM_001500.2 | GMDS | ILMN_16535 | -3.01 |
| XM_936103.1 | LOC642033 | ILMN_33652 | -3.04 |
| NM_012234.4 | RYBP | ILMN_13259 | -2.83 |
| NM_004257.3 | TGFBRAP1 | ILMN_30176 | -3.09 |
| NM_182533.1 | C1orf86 | ILMN_2880 | -3.01 |
| NM_057089.2 | AP1S1 | ILMN_4691 | -2.9 |
| NM_005694.1 | COX17 | ILMN_19252 | -2.85 |
| NM_016374.5 | ARID4B | ILMN_162934 | -2.97 |
| NM_022652.2 | DUSP6 | ILMN_5926 | -3.04 |
| NM_022743.1 | SMYD3 | ILMN_29453 | -3.07 |
| NM_024766.2 | C2orf34 | ILMN_14025 | -3.09 |
| NM_018334.3 | LRRN3 | ILMN_174401 | -2.85 |
| NM_006391.1 | IPO7 | ILMN_28842 | -3.15 |
| NM_020749.3 | MTUS1 | ILMN_4658 | -3.07 |
| NM_001099660.1 | LRRN3 | ILMN_306943 | -2.66 |
| NM_001545.1 | ICT1 | ILMN_11458 | -3.07 |
| XM_933956.1 | LOC644162 | ILMN_43225 | -3.02 |
| XM_944321.1 | LOC402560 | ILMN_42108 | -3.14 |
| NM_004891.2 | MRPL33 | ILMN_12897 | -2.88 |
| NM_031453.2 | FAM107B | ILMN_2236 | -2.9 |
| NM_017812.2 | CHCHD3 | ILMN_23539 | -3.07 |
| NM_001017963.2 | HSP90AA1 | ILMN_16669 | -2.82 |
| NM_001037675.1 | NBPF20 | ILMN_26956 | -2.82 |
| NM_002247.2 | KCNMA1 | ILMN_24236 | -3.4 |
| NM_006366.2 | CAP2 | ILMN_27367 | -3.3 |
| NM_019116.2 | UBFD1 | ILMN_179383 | -3.37 |
| NM_138418.2 | C16orf14 | ILMN_9509 | -3.39 |
| NM_001552.2 | IGFBP4 | ILMN_9309 | -3.31 |
| NM_020449.2 | THOC2 | ILMN_162047 | -3.28 |
| NM_006860.2 | RABL4 | ILMN_4559 | -3.4 |
| NM_002093.2 | GSK3B | ILMN_7421 | -3.38 |
| XM_938779.1 | LOC653972 | ILMN_31111 | -3.45 |
| NM_003542.3 | HIST1H4C | ILMN_30043 | -2.83 |
| XM_935818.1 | FLJ20397 | ILMN_137080 | -3.56 |
| NM_001025248.1 | DUT | ILMN_163345 | -3.47 |
| NM_078629.1 | MSL3L1 | ILMN_29354 | -3.45 |
| NM_006182.2 | DDR2 | ILMN_20698 | -3.45 |
| NM_017489.1 | TERF1 | ILMN_164297 | -3.55 |
| NM_001031711.1 | ERGIC1 | ILMN_7272 | -3.53 |
| NR_003041.1 | SNORD13 | ILMN_168446 | -3.84 |
| NM_133505.2 | DCN | ILMN_29913 | -3.59 |
| NM_022731.2 | NUCKS1 | ILMN_17108 | -3.71 |
| NM_006717.2 | SPIN1 | ILMN_23742 | -3.76 |
| NM_012342.2 | BAMBI | ILMN_8469 | -3.84 |
| NM_000598.4 | IGFBP3 | ILMN_28010 | -3.91 |
| NM_016374.5 | ARID4B | ILMN_162934 | -3.97 |
| NM_001018109.1 | PIR | ILMN_13999 | -3.76 |
| NM_004175.3 | SNRPD3 | ILMN_163179 | -3.82 |
| NM_000599.2 | IGFBP5 | ILMN_168089 | -3.69 |
| NM_003107.2 | SOX4 | ILMN_17456 | -3.72 |
| NR_003287.1 | LOC100008589 | ILMN_177351 | -4.1 |
| XM_374020.4 | LOC375295 | ILMN_45377 | -4.09 |
| NM_006914.3 | RORB | ILMN_7297 | -4.42 |
| NM_002167.2 | ID3 | ILMN_6829 | -4.59 |
| NM_014498.2 | GOLPH4 | ILMN_179486 | -4.76 |
| NM_138444.3 | KCTD12 | ILMN_18501 | -5.4 |
| NM_006265.1 | RAD21 | ILMN_171453 | -5.69 |
| NM_000599.2 | IGFBP5 | ILMN_168089 | -5.71 |
| NM_002166.4 | ID2 | ILMN_28481 | -5.88 |
| NM_002166.4 | ID2 | ILMN_28481 | -6.51 |
