# Supplementary material for: Minimal Peroxide Exposure of Neuronal Cells Induces Multifaceted Adaptive Responses
Source: PLoS One. 2010 Dec 17;5(12):e14352. doi: 10.1371/journal.pone.0014352 (PMC3003681; doi:10.1371/journal.pone.0014352)
Supplement: Table S9 — BDNF-significantly regulated genes after 2 hours of stimulation in the control state SH-SY5Y cells. Each significantly regulated gene is described via its accession number (ACCESSION), Gene Symbol (SYMBOL), Illumina array transcript designation (TRANSCRIPT). For each gene the z-ratio of expression compared to untreated cells after 2 hours of ligand stimulation is displayed (CTL BDNF 2). (1.03 MB DOC) [file pone.0014352.s016.doc]

**Table S9. BDNF-significantly regulated genes after 2 hours of stimulation in the control state SH-SY5Y cells**. Each significantly regulated gene is described via its accession number (ACCESSION), Gene Symbol (SYMBOL), Illumina array transcript designation (TRANSCRIPT). For each gene the z-ratio of expression compared to un-treated cells after 2 hours of ligand stimulation is displayed (CTL BDNF 2).

| **ACCESSION** | **SYMBOL** | **TRANSCRIPT** | **CTL BDNF 2** |
| --- | --- | --- | --- |
| NM_001964.2 | EGR1 | ILMN_20932 | 16.32 |
| NM_001554.3 | CYR61 | ILMN_21983 | 12.64 |
| NM_000584.2 | IL8 | ILMN_179575 | 7.31 |
| NM_005324.3 | H3F3B | ILMN_26885 | 5.27 |
| XM_935588.1 | LOC641848 | ILMN_45490 | 5.17 |
| NM_001010915.1 | PTPLAD2 | ILMN_6355 | 5.01 |
| NR_002315.1 | LOC440926 | ILMN_19720 | 4.96 |
| NM_017821.3 | RHBDL2 | ILMN_20003 | 4.94 |
| XM_370865.4 | LOC388122 | ILMN_46143 | 4.81 |
| NM_001003.2 | RPLP1 | ILMN_23181 | 4.75 |
| NM_001251.2 | CD68 | ILMN_5188 | 4.73 |
| XR_017492.1 | LOC644330 | ILMN_164787 | 4.57 |
| NM_006135.1 | CAPZA1 | ILMN_137637 | 4.41 |
| NM_001005474.1 | NFKBIZ | ILMN_16362 | 4.35 |
| NM_173666.1 | DTWD2 | ILMN_25915 | 4.33 |
| NM_003082.2 | SNAPC1 | ILMN_177713 | 4.2 |
| XM_375152.3 | LOC400304 | ILMN_46003 | 4.2 |
| NM_001300.4 | KLF6 | ILMN_17961 | 4.12 |
| XM_929199.1 | LOC644250 | ILMN_30796 | 3.96 |
| NR_003040.1 | LOC649946 | ILMN_169528 | 3.94 |
| NM_025152.1 | NUBPL | ILMN_25397 | 3.87 |
| NM_173518.2 | C8orf45 | ILMN_22241 | 3.79 |
| NM_003666.2 | BLZF1 | ILMN_21927 | 3.77 |
| NM_133459.1 | CCBE1 | ILMN_6075 | 3.76 |
| NM_178324.1 | SPTLC1 | ILMN_7889 | 3.74 |
| NM_006123.2 | IDS | ILMN_17605 | 3.72 |
| NM_012322.1 | LSM5 | ILMN_17896 | 3.71 |
| NM_001412.3 | EIF1AX | ILMN_22164 | 3.7 |
| NM_018097.1 | CEP27 | ILMN_15131 | 3.64 |
| NM_145913.2 | SLC5A8 | ILMN_7082 | 3.63 |
| NM_172014.1 | TNFSF14 | ILMN_9666 | 3.63 |
| NM_001034996.1 | RPL14 | ILMN_2719 | 3.62 |
| NM_004417.2 | DUSP1 | ILMN_20700 | 3.61 |
| XM_937113.2 | LOC647436 | ILMN_44829 | 3.6 |
| NM_012215.2 | MGEA5 | ILMN_11399 | 3.58 |
| NM_004859.3 | CLTC | ILMN_171089 | 3.56 |
| NM_004768.2 | SFRS11 | ILMN_4847 | 3.55 |
| XM_292963.6 | LOC643997 | ILMN_39721 | 3.55 |
| NM_001080973.1 | IL17RD | ILMN_179882 | 3.55 |
| NM_018561.3 | USP49 | ILMN_24018 | 3.53 |
| XR_018327.1 | LOC648343 | ILMN_163789 | 3.52 |
| NM_144736.3 | PRO1853 | ILMN_15591 | 3.51 |
| NM_005128.2 | DOPEY2 | ILMN_164626 | 3.51 |
| XM_930995.1 | LOC653086 | ILMN_31021 | 3.51 |
| NM_013361.3 | ZNF223 | ILMN_166150 | 3.49 |
| NM_033138.2 | CALD1 | ILMN_29896 | 3.47 |
| NM_001033506.1 | CSTF3 | ILMN_27049 | 3.47 |
| NM_012400.2 | PLA2G2D | ILMN_163941 | 3.47 |
| XM_944915.1 | PTP4A2 | ILMN_137656 | 3.47 |
| NM_001080484.1 | KIAA1751 | ILMN_180591 | 3.47 |
| NM_001008490.1 | KLF6 | ILMN_12381 | 3.46 |
| NR_002201.1 | FTHL3 | ILMN_27691 | 3.46 |
| XM_001134259.1 | LOC732165 | ILMN_170212 | 3.45 |
| NM_001099285.1 | PTMA | ILMN_306831 | 3.45 |
| NM_007285.6 | GABARAPL2 | ILMN_9805 | 3.43 |
| NM_172249.1 | CSF2RA | ILMN_5061 | 3.43 |
| NM_178231.1 | ALS2CR14 | ILMN_947 | 3.4 |
| XM_935770.1 | LOC641992 | ILMN_31870 | 3.37 |
| NM_033196.2 | ZNF682 | ILMN_21081 | 3.37 |
| NM_148174.2 | AZIN1 | ILMN_4931 | 3.36 |
| NM_020704.1 | FAM40B | ILMN_18452 | 3.36 |
| NR_002204.1 | FTHL11 | ILMN_16343 | 3.35 |
| NM_000572.2 | IL10 | ILMN_9173 | 3.34 |
| NM_176811.2 | NLRP8 | ILMN_169055 | 3.34 |
| NM_001080453.1 | INTS1 | ILMN_173681 | 3.33 |
| XM_937850.1 | LOC285176 | ILMN_43277 | 3.27 |
| XM_934113.1 | LOC653489 | ILMN_42664 | 3.25 |
| NM_002673.3 | PLXNB1 | ILMN_22628 | 3.24 |
| NM_152730.4 | C6orf170 | ILMN_17001 | 3.24 |
| NM_014395.1 | DAPP1 | ILMN_24094 | 3.24 |
| NM_032221.3 | CHD6 | ILMN_174095 | 3.23 |
| XM_945045.1 | LOC649679 | ILMN_34833 | 3.23 |
| NM_002166.4 | ID2 | ILMN_28481 | 3.23 |
| XM_941665.2 | LOC387763 | ILMN_43061 | 3.22 |
| NM_013412.1 | RABL2A | ILMN_12484 | 3.18 |
| NM_080386.1 | TUBA3D | ILMN_30319 | 3.18 |
| NM_001632.3 | ALPP | ILMN_25184 | 3.16 |
| NM_019119.3 | PCDHB9 | ILMN_23442 | 3.14 |
| XR_015809.1 | LOC728973 | ILMN_168278 | 3.14 |
| NM_020310.2 | MNT | ILMN_21283 | 3.13 |
| NM_002566.4 | P2RY11 | ILMN_12237 | 3.09 |
| NM_014817.3 | KIAA0644 | ILMN_164846 | 3.09 |
| XM_926231.1 | P704P | ILMN_36679 | 3.08 |
| NM_001013703.2 | EIF2AK4 | ILMN_164547 | 3.06 |
| NR_002205.1 | FTHL12 | ILMN_16447 | 3.06 |
| NM_001621.2 | AHR | ILMN_138365 | 3.06 |
| XR_016048.1 | MGC40489 | ILMN_171153 | 3.03 |
| NM_001008237.1 | TTC32 | ILMN_4829 | 3.03 |
| XM_941155.2 | LOC651894 | ILMN_33374 | 3.02 |
| XM_941684.2 | LOC220433 | ILMN_46655 | 3.02 |
| NR_001562.1 | ANXA2P1 | ILMN_10494 | 3.01 |
| XM_933893.1 | LOC389672 | ILMN_35589 | 3.01 |
| XM_936731.1 | LOC647673 | ILMN_33594 | 2.99 |
| XM_939697.1 | C9orf130 | ILMN_30981 | 2.98 |
| XM_944716.1 | LOC440704 | ILMN_34488 | 2.98 |
| NM_145280.3 | FAM119A | ILMN_15705 | 2.98 |
| NM_001030288.1 | SPN | ILMN_18556 | 2.97 |
| NM_004040.2 | RHOB | ILMN_162360 | 2.97 |
| NM_018698.3 | NXT2 | ILMN_168294 | 2.97 |
| XM_373031.3 | LOC391692 | ILMN_35197 | 2.95 |
| XM_938089.2 | LOC643007 | ILMN_31054 | 2.95 |
| XR_019339.1 | LOC643668 | ILMN_179350 | 2.95 |
| NM_001089.1 | ABCA3 | ILMN_18800 | 2.95 |
| NM_001008735.1 | HMG1L1 | ILMN_22757 | 2.95 |
| NM_001077628.1 | APH1A | ILMN_180233 | 2.93 |
| NM_002291.1 | LAMB1 | ILMN_182874 | 2.9 |
| XM_944104.2 | LOC653232 | ILMN_41197 | 2.9 |
| XM_938988.1 | LOC402221 | ILMN_35678 | 2.89 |
| NM_001029950.1 | DKFZp434K191 | ILMN_28495 | 2.87 |
| XM_928275.1 | LOC645236 | ILMN_36666 | 2.87 |
| XR_015514.1 | LOC730746 | ILMN_163533 | 2.87 |
| NM_014014.2 | ASCC3L1 | ILMN_18834 | 2.86 |
| NM_002228.3 | JUN | ILMN_7746 | 2.86 |
| NM_173042.2 | IL18BP | ILMN_30884 | 2.85 |
| NM_080702.2 | BAT3 | ILMN_4429 | 2.85 |
| NR_002203.1 | FTHL8 | ILMN_16227 | 2.84 |
| NM_021218.1 | C9orf80 | ILMN_27473 | 2.84 |
| NM_004145.2 | MYO9B | ILMN_25414 | 2.83 |
| NM_001031827.1 | BOLA2 | ILMN_4509 | 2.83 |
| NM_172097.1 | CATSPER2 | ILMN_23478 | 2.83 |
| NM_138701.1 | C7orf11 | ILMN_20229 | 2.82 |
| NM_001008237.1 | TTC32 | ILMN_4829 | 2.82 |
| XM_937928.1 | LOC347376 | ILMN_31523 | 2.81 |
| NM_022483.3 | C5orf28 | ILMN_5037 | 2.81 |
| NM_001039797.1 | FLJ46309 | ILMN_30948 | 2.81 |
| NM_000230.1 | LEP | ILMN_10827 | 2.8 |
| NM_014396.3 | VPS41 | ILMN_2386 | 2.79 |
| NM_001079842.1 | OCIAD1 | ILMN_163712 | 2.79 |
| NM_004038.3 | AMY1A | ILMN_176350 | 2.78 |
| XM_938297.1 | LOC402644 | ILMN_30715 | 2.78 |
| XM_935802.1 | LOC653829 | ILMN_46774 | 2.77 |
| NM_181054.1 | HIF1A | ILMN_9514 | 2.77 |
| NM_000383.1 | AIRE | ILMN_179368 | 2.77 |
| NM_004730.1 | ETF1 | ILMN_9222 | 2.76 |
| NR_002200.1 | FTHL2 | ILMN_15867 | 2.76 |
| NR_002803.1 | LOC283345 | ILMN_14409 | 2.76 |
| NM_003677.3 | DENR | ILMN_181187 | 2.75 |
| NM_001029862.1 | ANKRD30B | ILMN_7263 | 2.75 |
| NM_033109.2 | PNPT1 | ILMN_22316 | 2.75 |
| NM_198795.1 | TDRD1 | ILMN_162663 | 2.73 |
| NM_002266.2 | KPNA2 | ILMN_14206 | 2.73 |
| NM_005010.3 | NRCAM | ILMN_8955 | 2.73 |
| NM_001039755.1 | FLJ44124 | ILMN_44450 | 2.73 |
| NM_001035005.2 | C18orf32 | ILMN_26126 | 2.72 |
| NM_014478.4 | RCP9 | ILMN_22485 | 2.72 |
| XM_930178.1 | LOC645018 | ILMN_33646 | 2.71 |
| NM_006731.2 | FKTN | ILMN_6512 | 2.71 |
| NM_003878.1 | GGH | ILMN_9870 | 2.7 |
| XM_940610.1 | LOC651453 | ILMN_32585 | 2.7 |
| NM_005443.4 | PAPSS1 | ILMN_171260 | 2.7 |
| XM_942501.1 | CSF2RA | ILMN_137685 | 2.7 |
| NM_014897.1 | ZNF652 | ILMN_3215 | 2.7 |
| NM_032485.4 | MCM8 | ILMN_11171 | 2.7 |
| XM_944439.2 | LOC653994 | ILMN_38572 | 2.69 |
| NR_002939.2 | RUNDC2C | ILMN_39843 | 2.69 |
| NM_033412.1 | MCART1 | ILMN_22327 | 2.69 |
| NR_002204.1 | FTHL11 | ILMN_16343 | 2.67 |
| XM_940333.2 | LOC651202 | ILMN_37363 | 2.67 |
| NM_022308.1 | ICA1 | ILMN_12918 | 2.66 |
| NM_006392.2 | NOL5A | ILMN_13841 | 2.65 |
| NM_016623.3 | FAM49B | ILMN_14248 | 2.64 |
| XM_942687.1 | LOC654189 | ILMN_30702 | 2.64 |
| NM_014380.1 | NGFRAP1 | ILMN_7162 | 2.63 |
| NM_207331.2 | LOC153561 | ILMN_1879 | 2.61 |
| NM_138477.2 | CDAN1 | ILMN_168162 | 2.61 |
| NM_020409.2 | MRPL47 | ILMN_12272 | 2.6 |
| NM_033419.3 | PERLD1 | ILMN_12215 | 2.6 |
| NR_002808.1 | C14orf85 | ILMN_14639 | 2.6 |
| NM_004156.2 | PPP2CB | ILMN_21592 | 2.59 |
| NM_005665.4 | EVI5 | ILMN_17996 | 2.57 |
| XM_001129527.1 | KLF11 | ILMN_168976 | 2.57 |
| NM_199043.1 | C14orf102 | ILMN_22442 | 2.56 |
| NM_005245.3 | FAT | ILMN_24617 | 2.56 |
| NM_032796.2 | SYAP1 | ILMN_8037 | 2.56 |
| NM_000978.3 | RPL23 | ILMN_8866 | 2.56 |
| NM_016028.4 | SUV420H1 | ILMN_29861 | 2.55 |
| NM_002874.3 | RAD23B | ILMN_19346 | 2.54 |
| NM_024570.1 | RNASEH2B | ILMN_20578 | 2.53 |
| NM_138687.1 | PIP5K2B | ILMN_12735 | 2.53 |
| NM_015180.4 | SYNE2 | ILMN_183979 | 2.52 |
| NM_032389.3 | ARFGAP2 | ILMN_12944 | 2.51 |
| NM_024311.2 | MFSD11 | ILMN_7695 | 2.49 |
| NM_175066.2 | DDX51 | ILMN_165366 | 2.49 |
| NM_001562.2 | IL18 | ILMN_167736 | 2.47 |
| NM_001788.4 | 7-Sep | ILMN_25070 | 2.46 |
| NR_003277.1 | LOC728643 | ILMN_183126 | 2.46 |
| NM_032794.1 | SLC44A4 | ILMN_14709 | 2.46 |
| XM_495939.3 | KIAA1545 | ILMN_40920 | 2.45 |
| NM_001017998.2 | GNG10 | ILMN_34149 | 2.45 |
| NM_016617.1 | UFM1 | ILMN_12438 | 2.45 |
| NM_001013258.1 | ZNF789 | ILMN_11535 | 2.45 |
| NM_006903.4 | PPA2 | ILMN_15173 | 2.45 |
| NM_018697.3 | LANCL2 | ILMN_920 | 2.44 |
| NM_080491.1 | GAB2 | ILMN_3317 | 2.43 |
| NM_001030.3 | RPS27 | ILMN_5932 | 2.43 |
| NM_001080.3 | ALDH5A1 | ILMN_1025 | 2.42 |
| NM_021190.1 | PTBP2 | ILMN_556 | 2.42 |
| NM_003110.4 | SP2 | ILMN_7882 | 2.41 |
| NM_025084.1 | FLJ22795 | ILMN_1721 | 2.41 |
| NM_020040.3 | TUBB4Q | ILMN_177504 | 2.41 |
| NM_001095.2 | ACCN2 | ILMN_27416 | 2.4 |
| NM_032440.1 | LCOR | ILMN_173510 | 2.4 |
| NM_005334.2 | HCFC1 | ILMN_24237 | 2.4 |
| NM_022487.2 | DCLRE1C | ILMN_28391 | 2.39 |
| NM_014330.2 | PPP1R15A | ILMN_1024 | 2.38 |
| XR_017397.1 | LOC644029 | ILMN_163901 | 2.38 |
| NM_199436.1 | SPAST | ILMN_15461 | 2.37 |
| NM_001081637.1 | LILRB1 | ILMN_164019 | 2.37 |
| NM_006372.3 | SYNCRIP | ILMN_28470 | 2.36 |
| XM_941195.2 | LOC388621 | ILMN_42661 | 2.36 |
| NM_014363.3 | SACS | ILMN_180142 | 2.35 |
| NM_177530.1 | SULT1A1 | ILMN_4950 | 2.35 |
| XM_944991.1 | C14orf82 | ILMN_36432 | 2.34 |
| NM_032242.2 | PLXNA1 | ILMN_912 | 2.34 |
| NM_017833.2 | C21orf55 | ILMN_6782 | 2.34 |
| NM_020801.1 | ARRDC3 | ILMN_22538 | 2.33 |
| NM_003220.2 | TFAP2A | ILMN_17128 | 2.32 |
| NM_020822.1 | KCNT1 | ILMN_21599 | 2.32 |
| NM_025189.2 | ZNF430 | ILMN_24543 | 2.32 |
| NM_007112.3 | THBS3 | ILMN_10000 | 2.31 |
| NM_000479.2 | AMH | ILMN_171371 | 2.31 |
| NM_001040456.1 | RHBDD2 | ILMN_168345 | 2.31 |
| NM_006004.1 | UQCRH | ILMN_138507 | 2.31 |
| NM_001005849.1 | SUMO2 | ILMN_16713 | 2.31 |
| XM_932717.2 | LOC643224 | ILMN_34053 | 2.3 |
| NM_005627.2 | SGK | ILMN_2451 | 2.3 |
| NM_182492.1 | LRP5L | ILMN_650 | 2.29 |
| XM_926594.2 | LOC642502 | ILMN_31759 | 2.29 |
| NM_012343.3 | NNT | ILMN_183201 | 2.29 |
| NR_003264.1 | SDHALP1 | ILMN_175200 | 2.29 |
| NM_003617.2 | RGS5 | ILMN_167992 | 2.29 |
| NM_003069.2 | SMARCA1 | ILMN_181613 | 2.28 |
| NM_007118.2 | TRIO | ILMN_23876 | 2.28 |
| NM_003925.1 | MBD4 | ILMN_18891 | 2.28 |
| NM_002996.3 | CX3CL1 | ILMN_9636 | 2.26 |
| NM_031943.1 | IFP38 | ILMN_9478 | 2.26 |
| NM_001111.3 | ADAR | ILMN_20593 | 2.25 |
| NM_001031.4 | RPS28 | ILMN_992 | 2.25 |
| NM_000786.2 | CYP51A1 | ILMN_161878 | 2.24 |
| NM_177965.2 | C8orf37 | ILMN_11118 | 2.24 |
| NM_144618.1 | MGC29891 | ILMN_17631 | 2.24 |
| NM_001013685.1 | LOC401357 | ILMN_29013 | 2.22 |
| NM_001006.3 | RPS3A | ILMN_28872 | 2.22 |
| NM_002923.1 | RGS2 | ILMN_26119 | 2.21 |
| XM_001134215.1 | PDPR | ILMN_162295 | 2.21 |
| XM_938755.2 | LOC653773 | ILMN_44662 | 2.21 |
| NM_134265.2 | WSB1 | ILMN_5396 | 2.2 |
| NM_006572.3 | GNA13 | ILMN_173569 | 2.19 |
| NM_024561.3 | NARG1L | ILMN_22547 | 2.18 |
| NM_148957.2 | TNFRSF19 | ILMN_28684 | 2.18 |
| XM_935589.1 | LOC641849 | ILMN_45563 | 2.18 |
| XM_926036.1 | LOC653103 | ILMN_32029 | 2.18 |
| NM_004560.2 | ROR2 | ILMN_22834 | 2.17 |
| NM_031372.1 | HNRPDL | ILMN_15196 | 2.17 |
| NM_032753.2 | RAXL1 | ILMN_21452 | 2.16 |
| XM_001133089.1 | LOC731640 | ILMN_161930 | 2.16 |
| NM_006806.3 | BTG3 | ILMN_27215 | 2.15 |
| NM_006988.3 | ADAMTS1 | ILMN_11081 | 2.15 |
| NM_024804.1 | ZNF669 | ILMN_26142 | 2.15 |
| NM_001034996.1 | RPL14 | ILMN_2719 | 2.14 |
| NR_002205.1 | FTHL12 | ILMN_16447 | 2.14 |
| NM_001038702.1 | CDC42SE2 | ILMN_28719 | 2.13 |
| NM_014033.3 | METTL7A | ILMN_40171 | 2.13 |
| NM_001025780.1 | FAM108B1 | ILMN_181005 | 2.12 |
| NM_016114.3 | ASB1 | ILMN_11707 | 2.12 |
| NM_005721.3 | ACTR3 | ILMN_11792 | 2.12 |
| NM_152411.2 | ZNF786 | ILMN_6379 | 2.12 |
| NM_000046.2 | ARSB | ILMN_180341 | 2.11 |
| NR_001434.1 | HLA-H | ILMN_5683 | 2.11 |
| NM_005157.3 | ABL1 | ILMN_4033 | 2.11 |
| NM_020724.1 | RNF150 | ILMN_26801 | 2.11 |
| NM_001044387.1 | ZNF557 | ILMN_180266 | 2.1 |
| NR_002448.1 | SNORD36A | ILMN_20691 | 2.09 |
| NM_183422.1 | TSC22D1 | ILMN_166165 | 2.09 |
| NM_003183.4 | ADAM17 | ILMN_165100 | 2.09 |
| XM_928075.2 | LOC643287 | ILMN_37869 | 2.08 |
| NM_006958.2 | ZNF16 | ILMN_17198 | 2.07 |
| NM_014935.2 | PLEKHA6 | ILMN_163005 | 2.07 |
| NM_020368.1 | UTP3 | ILMN_22785 | 2.07 |
| NM_002129.2 | HMGB2 | ILMN_3200 | 2.07 |
| NM_014762.3 | DHCR24 | ILMN_173913 | 2.06 |
| NM_002849.2 | PTPRR | ILMN_178122 | 2.06 |
| NM_006628.4 | ARPP-19 | ILMN_2093 | 2.06 |
| NM_006265.1 | RAD21 | ILMN_171453 | 2.06 |
| NM_030793.3 | FBXO38 | ILMN_4373 | 2.05 |
| NM_002128.4 | HMGB1 | ILMN_23421 | 2.05 |
| XM_940209.1 | KIAA0194 | ILMN_37512 | 2.05 |
| NM_006885.3 | ZFHX3 | ILMN_174159 | 2.05 |
| NM_032036.2 | FAM14A | ILMN_19102 | 2.04 |
| XM_938599.2 | LOC441377 | ILMN_31681 | 2.04 |
| NM_000971.3 | RPL7 | ILMN_26351 | 2.03 |
| NM_004779.4 | CNOT8 | ILMN_10063 | 2.02 |
| NM_001008393.1 | LOC201725 | ILMN_20795 | 2.02 |
| NM_004075.2 | CRY1 | ILMN_6263 | 2.02 |
| NM_003086.2 | SNAPC4 | ILMN_180505 | 2.02 |
| NM_001017421.1 | FKSG30 | ILMN_2393 | 2.02 |
| NM_002213.3 | ITGB5 | ILMN_24189 | 2.01 |
| NM_012424.2 | RPS6KC1 | ILMN_165437 | 2.01 |
| NM_022720.5 | DGCR8 | ILMN_1552 | 2.01 |
| NM_032026.1 | TATDN1 | ILMN_17501 | 2.01 |
| NM_002951.2 | RPN2 | ILMN_30123 | 2.01 |
| NM_130473.1 | MADD | ILMN_12717 | 2 |
| NM_014712.1 | SETD1A | ILMN_24764 | 2 |
| NM_001024921.2 | RPL9 | ILMN_8640 | 2 |
| NM_005154.2 | USP8 | ILMN_15425 | 1.99 |
| NM_005238.2 | ETS1 | ILMN_173009 | 1.99 |
| NM_014614.1 | PSME4 | ILMN_164803 | 1.99 |
| NM_006360.3 | EIF3M | ILMN_19862 | 1.99 |
| NM_024745.2 | SHCBP1 | ILMN_166259 | 1.99 |
| NM_182679.1 | GPATCH4 | ILMN_839 | 1.98 |
| NM_002685.2 | EXOSC10 | ILMN_25853 | 1.98 |
| NM_004508.2 | IDI1 | ILMN_20349 | 1.97 |
| NM_213606.1 | SLC16A12 | ILMN_28607 | 1.97 |
| NM_201997.1 | SF1 | ILMN_2017 | 1.96 |
| NM_001008408.3 | RBM33 | ILMN_165407 | 1.96 |
| NM_018249.4 | CDK5RAP2 | ILMN_9876 | 1.96 |
| NM_005497.3 | GJC1 | ILMN_3556 | 1.96 |
| NM_014694.2 | ADAMTSL2 | ILMN_697 | 1.95 |
| NM_004788.2 | UBE4A | ILMN_175730 | 1.95 |
| NM_021190.1 | PTBP2 | ILMN_556 | 1.95 |
| NR_003664.1 | LOC389517 | ILMN_307371 | 1.95 |
| NM_017921.1 | NPLOC4 | ILMN_12904 | 1.94 |
| NM_014060.1 | MCTS1 | ILMN_13725 | 1.93 |
| NM_001012626.1 | LOC285074 | ILMN_21153 | 1.92 |
| NM_018479.2 | ECHDC1 | ILMN_1493 | 1.92 |
| NM_014838.2 | ZBED4 | ILMN_8641 | 1.92 |
| NM_001319.5 | CSNK1G2 | ILMN_17274 | 1.92 |
| NM_001042414.1 | PSPC1 | ILMN_183553 | 1.91 |
| NM_022459.3 | XPO4 | ILMN_164187 | 1.9 |
| NM_015124.2 | GRAMD4 | ILMN_12136 | 1.9 |
| NM_006275.4 | SFRS6 | ILMN_24964 | 1.9 |
| NM_001001789.1 | C21orf24 | ILMN_1369 | 1.9 |
| NM_015447.1 | CAMSAP1 | ILMN_815 | 1.89 |
| NM_005754.2 | G3BP1 | ILMN_1152 | 1.89 |
| NM_000617.1 | SLC11A2 | ILMN_10129 | 1.88 |
| NR_000011.1 | SNORA70 | ILMN_7210 | 1.88 |
| NM_006925.3 | SFRS5 | ILMN_34497 | 1.88 |
| NM_003104.3 | SORD | ILMN_162054 | 1.87 |
| NM_003461.4 | ZYX | ILMN_2137 | 1.87 |
| NM_003486.5 | SLC7A5 | ILMN_25446 | 1.87 |
| NM_021227.2 | DC2 | ILMN_24748 | 1.87 |
| NM_014367.3 | C3orf28 | ILMN_24382 | 1.86 |
| XM_942544.2 | INTS1 | ILMN_38896 | 1.86 |
| NM_005385.3 | NKTR | ILMN_23378 | 1.86 |
| NM_000587.2 | C7 | ILMN_15063 | 1.86 |
| NM_001677.3 | ATP1B1 | ILMN_25542 | 1.86 |
| NM_001080485.1 | ZNF275 | ILMN_180340 | 1.85 |
| NM_020695.3 | REXO1 | ILMN_20923 | 1.84 |
| NM_021960.3 | MCL1 | ILMN_18397 | 1.84 |
| NM_020859.1 | SHRM | ILMN_16821 | 1.84 |
| NM_182649.1 | PCNA | ILMN_6858 | 1.84 |
| NM_025230.3 | WDR23 | ILMN_171432 | 1.83 |
| NM_003418.1 | CNBP | ILMN_9092 | 1.83 |
| NM_001006946.1 | SDC1 | ILMN_169032 | 1.83 |
| NM_001079673.1 | FNDC3A | ILMN_167386 | 1.82 |
| NR_002197.1 | LOC143543 | ILMN_17694 | 1.82 |
| NM_014747.2 | RIMS3 | ILMN_21581 | 1.82 |
| XM_940278.1 | LOC651149 | ILMN_44210 | 1.82 |
| NM_001008735.1 | HMG1L1 | ILMN_22757 | 1.82 |
| XM_938150.2 | LOC644310 | ILMN_31967 | 1.81 |
| NM_178517.3 | PIGW | ILMN_162681 | 1.81 |
| NM_015306.1 | USP24 | ILMN_309418 | 1.81 |
| NM_006107.2 | CROP | ILMN_10300 | 1.81 |
| NM_004238.1 | TRIP12 | ILMN_21755 | 1.8 |
| NM_001079673.1 | FNDC3A | ILMN_167386 | 1.79 |
| NM_022731.2 | NUCKS1 | ILMN_17108 | 1.79 |
| NM_006527.2 | SLBP | ILMN_3687 | 1.79 |
| NM_033285.2 | TP53INP1 | ILMN_16203 | 1.79 |
| NM_003749.2 | IRS2 | ILMN_167991 | 1.79 |
| NM_022098.2 | XPNPEP3 | ILMN_23195 | 1.79 |
| XM_001126418.1 | LOC727935 | ILMN_181411 | 1.78 |
| NM_001006.3 | RPS3A | ILMN_28872 | 1.78 |
| NM_133487.1 | RAD51 | ILMN_4856 | 1.77 |
| NM_018708.2 | FEM1A | ILMN_2838 | 1.77 |
| NM_001001679.1 | FLJ41423 | ILMN_1487 | 1.76 |
| XM_928934.1 | LOC645968 | ILMN_45269 | 1.76 |
| NM_145341.2 | PDCD4 | ILMN_12916 | 1.76 |
| XM_939726.2 | LOC388532 | ILMN_45940 | 1.76 |
| NM_012130.2 | CLDN14 | ILMN_19370 | 1.75 |
| NM_006206.3 | PDGFRA | ILMN_165232 | 1.75 |
| NM_003906.3 | MCM3AP | ILMN_19614 | 1.75 |
| NM_002482.2 | NASP | ILMN_21654 | 1.75 |
| NM_001023567.2 | GOLGA8B | ILMN_14405 | 1.75 |
| NM_005779.1 | LHFPL2 | ILMN_26106 | 1.74 |
| NM_002166.4 | ID2 | ILMN_28481 | 1.73 |
| NM_001039705.1 | TRO | ILMN_32618 | 1.73 |
| XM_931359.2 | LOC338758 | ILMN_37634 | 1.72 |
| NM_001004322.1 | FLJ38717 | ILMN_13488 | 1.72 |
| NM_016265.3 | ZNF12 | ILMN_27628 | 1.72 |
| NM_013291.2 | CPSF1 | ILMN_22094 | 1.72 |
| NM_004687.3 | MTMR4 | ILMN_163329 | 1.72 |
| NM_000090.3 | COL3A1 | ILMN_182795 | 1.72 |
| NM_005921.1 | MAP3K1 | ILMN_309540 | 1.72 |
| NM_003076.3 | SMARCD1 | ILMN_16093 | 1.72 |
| NM_005646.3 | TARBP1 | ILMN_14414 | 1.71 |
| NM_022064.2 | RNF123 | ILMN_29794 | 1.71 |
| XM_001127981.1 | LOC728014 | ILMN_169164 | 1.71 |
| NM_016097.3 | IER3IP1 | ILMN_21844 | 1.71 |
| NM_001287.3 | CLCN7 | ILMN_8600 | 1.71 |
| NM_198679.1 | RAPGEF1 | ILMN_177243 | 1.7 |
| NM_203499.1 | DDX42 | ILMN_1880 | 1.7 |
| NM_018639.3 | WSB2 | ILMN_162438 | 1.69 |
| NM_017741.3 | C4orf30 | ILMN_172318 | 1.69 |
| XR_018923.1 | LOC648210 | ILMN_162972 | 1.69 |
| NM_014719.1 | FAM115A | ILMN_27255 | 1.69 |
| NM_020933.2 | ZNF317 | ILMN_22884 | 1.68 |
| NM_019015.1 | CSGlcA-T | ILMN_21838 | 1.68 |
| NM_001037533.1 | GON4L | ILMN_14180 | 1.68 |
| NM_006997.2 | TACC2 | ILMN_16130 | 1.68 |
| NM_174909.3 | TMEM167 | ILMN_6582 | 1.68 |
| NM_006836.1 | GCN1L1 | ILMN_26435 | 1.68 |
| NM_152322.2 | BTBD11 | ILMN_506 | 1.67 |
| NM_021807.3 | EXOC4 | ILMN_28890 | 1.67 |
| NM_021737.1 | CLCN6 | ILMN_6195 | 1.67 |
| NM_000743.2 | CHRNA3 | ILMN_23268 | 1.67 |
| NM_006054.2 | RTN3 | ILMN_20331 | 1.67 |
| NM_012342.2 | BAMBI | ILMN_8469 | 1.67 |
| NM_152679.2 | SLC10A4 | ILMN_1323 | 1.67 |
| NM_022781.4 | RNF38 | ILMN_40416 | 1.66 |
| NM_006516.1 | SLC2A1 | ILMN_421 | 1.66 |
| NM_013275.4 | ANKRD11 | ILMN_28595 | 1.66 |
| NM_022766.4 | CERK | ILMN_24122 | 1.66 |
| NM_182661.1 | CERK | ILMN_2275 | 1.66 |
| NM_152280.2 | SYT11 | ILMN_23967 | 1.66 |
| NM_014772.1 | KIAA0427 | ILMN_182540 | 1.65 |
| NM_003119.2 | SPG7 | ILMN_26332 | 1.65 |
| NR_002190.1 | SUMO1P3 | ILMN_16906 | 1.65 |
| NM_021569.2 | GRIN1 | ILMN_28874 | 1.64 |
| NM_015352.1 | POFUT1 | ILMN_7876 | 1.64 |
| NM_020410.1 | ATP13A1 | ILMN_12379 | 1.64 |
| NM_015338.4 | ASXL1 | ILMN_183479 | 1.64 |
| NM_030806.3 | C1orf21 | ILMN_26434 | 1.64 |
| XM_927280.1 | LOC644033 | ILMN_39734 | 1.64 |
| NM_030752.2 | TCP1 | ILMN_418 | 1.64 |
| NM_030881.2 | DDX17 | ILMN_28024 | 1.64 |
| NM_001013699.1 | LOC440093 | ILMN_19743 | 1.64 |
| NM_003463.3 | PTP4A1 | ILMN_165831 | 1.63 |
| NM_024909.1 | C6orf134 | ILMN_21139 | 1.63 |
| NM_017819.2 | RG9MTD1 | ILMN_26970 | 1.63 |
| NM_001012614.1 | CTBP1 | ILMN_21952 | 1.63 |
| NM_003565.1 | ULK1 | ILMN_2158 | 1.63 |
| NM_194278.3 | C14orf43 | ILMN_166357 | 1.62 |
| NM_005093.3 | CBFA2T2 | ILMN_42009 | 1.62 |
| NM_015226.1 | CLEC16A | ILMN_19348 | 1.62 |
| NM_001357.2 | DHX9 | ILMN_7196 | 1.61 |
| NM_152398.2 | OCIAD2 | ILMN_18246 | 1.61 |
| NM_017948.4 | NOL8 | ILMN_25734 | 1.6 |
| NM_018116.2 | MSTO1 | ILMN_1073 | 1.6 |
| NM_016245.3 | HSD17B11 | ILMN_12219 | 1.6 |
| NM_001144.4 | AMFR | ILMN_22219 | 1.6 |
| NM_001040439.1 | MAPK8IP3 | ILMN_174436 | 1.6 |
| NM_019106.4 | 3-Sep | ILMN_4065 | 1.6 |
| NM_001013251.1 | SLC3A2 | ILMN_12826 | 1.59 |
| XM_937706.1 | LOC648638 | ILMN_41215 | 1.59 |
| NM_001048201.1 | UHRF1 | ILMN_162952 | 1.59 |
| NM_033083.6 | EAF1 | ILMN_173601 | 1.58 |
| XR_016986.1 | LOC643668 | ILMN_172192 | 1.58 |
| NM_002737.2 | PRKCA | ILMN_24085 | 1.58 |
| NM_033063.1 | MAP6 | ILMN_6882 | 1.57 |
| NM_002048.1 | GAS1 | ILMN_175833 | 1.57 |
| NM_015516.3 | TSKU | ILMN_29523 | 1.57 |
| NM_020824.2 | ARHGAP21 | ILMN_10414 | 1.57 |
| NM_003045.3 | SLC7A1 | ILMN_162673 | 1.57 |
| NM_012207.1 | HNRPH3 | ILMN_8162 | 1.57 |
| XM_937154.1 | LOC648099 | ILMN_35361 | 1.56 |
| NM_198310.2 | TTC8 | ILMN_20549 | 1.56 |
| NM_198836.1 | ACACA | ILMN_9534 | 1.56 |
| NM_006185.2 | NUMA1 | ILMN_25058 | 1.56 |
| NM_173073.2 | SLC35C2 | ILMN_14167 | 1.55 |
| NM_174921.1 | C4orf34 | ILMN_6140 | 1.55 |
| NM_014839.3 | LPPR4 | ILMN_15660 | 1.54 |
| NM_014422.2 | PIB5PA | ILMN_8156 | 1.54 |
| NM_004055.4 | CAPN5 | ILMN_30845 | 1.54 |
| NM_198334.1 | GANAB | ILMN_29263 | 1.54 |
| NM_014849.2 | SV2A | ILMN_14178 | 1.54 |
| NM_006047.4 | RBM12 | ILMN_183773 | 1.54 |
| NM_017707.2 | DDEFL1 | ILMN_25222 | 1.54 |
| NM_177972.1 | TUB | ILMN_11520 | 1.54 |
| NR_003659.1 | FAM39DP | ILMN_307683 | 1.54 |
| NM_006925.3 | SFRS5 | ILMN_34497 | 1.54 |
| NM_002938.2 | RNF4 | ILMN_176496 | 1.53 |
| NM_152834.2 | TMEM18 | ILMN_8053 | 1.53 |
| NM_022652.2 | DUSP6 | ILMN_5926 | 1.53 |
| NM_194301.2 | GARNL1 | ILMN_2823 | 1.53 |
| NM_005994.3 | TBX2 | ILMN_18788 | 1.53 |
| NM_033214.2 | GK2 | ILMN_27715 | 1.52 |
| NM_021226.2 | ARHGAP22 | ILMN_15801 | 1.52 |
| NM_032924.3 | ZNF3 | ILMN_25682 | 1.52 |
| NM_015356.3 | SCRIB | ILMN_1684 | 1.52 |
| NM_001013845.1 | CXorf40B | ILMN_170421 | 1.51 |
| NM_020248.2 | CTNNBIP1 | ILMN_23888 | 1.51 |
| NM_004284.3 | CHD1L | ILMN_7932 | 1.51 |
| NM_001995.2 | ACSL1 | ILMN_12367 | 1.5 |
| NM_020755.2 | SERINC1 | ILMN_24825 | 1.5 |
| NM_005744.2 | ARIH1 | ILMN_16556 | -1.5 |
| NM_001007794.1 | CEPT1 | ILMN_15134 | -1.5 |
| NM_004378.1 | CRABP1 | ILMN_12739 | -1.5 |
| NM_032111.2 | MRPL14 | ILMN_15720 | -1.5 |
| NM_020705.1 | TBC1D24 | ILMN_34755 | -1.5 |
| NM_001010982.2 | AFMID | ILMN_5520 | -1.51 |
| NM_006010.2 | ARMET | ILMN_28822 | -1.51 |
| NM_145863.1 | ASB3 | ILMN_24959 | -1.51 |
| NM_079837.2 | BANP | ILMN_8638 | -1.51 |
| NM_001466.2 | FZD2 | ILMN_12499 | -1.51 |
| NM_198047.1 | HIBCH | ILMN_24888 | -1.51 |
| NM_005707.1 | PDCD7 | ILMN_179659 | -1.51 |
| NM_016166.1 | PIAS1 | ILMN_16806 | -1.51 |
| NM_002692.2 | POLE2 | ILMN_19705 | -1.51 |
| NM_203291.1 | RBBP8 | ILMN_1238 | -1.51 |
| NM_021253.2 | TRIM39 | ILMN_165050 | -1.51 |
| XM_942289.1 | LOC652685 | ILMN_46541 | -1.52 |
| NM_032285.2 | MGC3207 | ILMN_3158 | -1.52 |
| NM_020385.2 | REXO4 | ILMN_29774 | -1.52 |
| NM_003358.1 | UGCG | ILMN_26228 | -1.52 |
| NM_018206.3 | VPS35 | ILMN_21093 | -1.52 |
| NM_001077268.1 | ZFYVE19 | ILMN_175347 | -1.52 |
| NM_016324.2 | ZNF274 | ILMN_6185 | -1.52 |
| NM_023039.2 | ANKRA2 | ILMN_16224 | -1.53 |
| NM_001031713.2 | CCDC90A | ILMN_9159 | -1.53 |
| NR_002196.1 | H19 | ILMN_18538 | -1.53 |
| NM_052940.3 | LRRC42 | ILMN_8345 | -1.53 |
| NM_138794.2 | LYPLAL1 | ILMN_25005 | -1.53 |
| NM_174926.1 | TMEM136 | ILMN_16309 | -1.53 |
| NM_015984.2 | UCHL5 | ILMN_3370 | -1.53 |
| NM_005087.2 | FXR1 | ILMN_18674 | -1.54 |
| NM_000155.2 | GALT | ILMN_1433 | -1.54 |
| NM_018410.3 | HJURP | ILMN_29337 | -1.54 |
| NM_032476.2 | MRPS6 | ILMN_17239 | -1.54 |
| NM_002467.3 | MYC | ILMN_28130 | -1.54 |
| NM_080723.3 | NRSN1 | ILMN_178353 | -1.54 |
| NM_003744.5 | NUMB | ILMN_24350 | -1.54 |
| NM_001040285.1 | PAPD5 | ILMN_167231 | -1.54 |
| NM_003083.2 | SNAPC2 | ILMN_14587 | -1.54 |
| NM_003129.3 | SQLE | ILMN_521 | -1.54 |
| NM_018412.3 | ST7 | ILMN_16575 | -1.54 |
| NM_024292.2 | UBL5 | ILMN_14261 | -1.54 |
| NM_024097.1 | C1orf50 | ILMN_12960 | -1.55 |
| NM_022087.2 | GALNT11 | ILMN_5237 | -1.55 |
| NM_005333.2 | HCCS | ILMN_1794 | -1.55 |
| NM_021222.1 | PRUNE | ILMN_27601 | -1.55 |
| NM_032936.2 | TMEM60 | ILMN_19683 | -1.55 |
| NM_001124.1 | ADM | ILMN_29514 | -1.56 |
| NM_001009608.1 | C20orf94 | ILMN_24801 | -1.56 |
| NM_016941.2 | DLL3 | ILMN_21363 | -1.56 |
| NM_024090.1 | ELOVL6 | ILMN_11340 | -1.56 |
| NM_001040057.1 | FAM133B | ILMN_181197 | -1.56 |
| NM_203284.1 | RBPJ | ILMN_170184 | -1.56 |
| NM_005652.2 | TERF2 | ILMN_21134 | -1.56 |
| NM_014886.3 | TINP1 | ILMN_8436 | -1.56 |
| NM_005692.3 | ABCF2 | ILMN_14116 | -1.57 |
| NM_031885.2 | BBS2 | ILMN_12583 | -1.57 |
| NM_138787.2 | C11orf74 | ILMN_16125 | -1.57 |
| NM_139286.3 | CDC26 | ILMN_18022 | -1.57 |
| NM_005147.3 | DNAJA3 | ILMN_10747 | -1.57 |
| NM_006310.2 | NPEPPS | ILMN_184074 | -1.57 |
| NM_177968.2 | PPM1B | ILMN_29648 | -1.57 |
| NM_024321.3 | RBM42 | ILMN_182570 | -1.57 |
| NM_014347.1 | ZNF324 | ILMN_29920 | -1.57 |
| NM_016008.2 | DYNC2LI1 | ILMN_14439 | -1.58 |
| NM_012405.3 | ICMT | ILMN_31192 | -1.58 |
| NM_006084.4 | IRF9 | ILMN_163101 | -1.58 |
| NM_030805.2 | LMAN2L | ILMN_1985 | -1.58 |
| NM_020772.1 | NUFIP2 | ILMN_19967 | -1.58 |
| NM_152705.1 | POLR1D | ILMN_28050 | -1.58 |
| NM_003475.2 | RASSF7 | ILMN_12457 | -1.58 |
| NM_001042588.1 | SNUPN | ILMN_178280 | -1.58 |
| NM_001078651.1 | TMEM134 | ILMN_176754 | -1.58 |
| NM_024315.2 | C7orf23 | ILMN_6840 | -1.59 |
| NM_133371.2 | MYOZ3 | ILMN_21305 | -1.59 |
| NM_003192.2 | TBCC | ILMN_25053 | -1.59 |
| NM_033661.3 | WDR4 | ILMN_3481 | -1.59 |
| XM_941876.1 | BRI3BP | ILMN_139088 | -1.6 |
| NM_001545.1 | ICT1 | ILMN_11458 | -1.6 |
| NM_005833.2 | RABEPK | ILMN_4050 | -1.6 |
| NM_005868.4 | BET1 | ILMN_4222 | -1.61 |
| NM_006712.3 | FASTK | ILMN_11299 | -1.61 |
| NM_007044.2 | KATNA1 | ILMN_9099 | -1.61 |
| XM_001127763.1 | LOC730820 | ILMN_162734 | -1.61 |
| NM_080546.3 | SLC44A1 | ILMN_23525 | -1.61 |
| NM_003368.4 | USP1 | ILMN_5285 | -1.61 |
| NM_032172.1 | USP42 | ILMN_162869 | -1.61 |
| NM_000016.2 | ACADM | ILMN_161879 | -1.62 |
| NM_152408.1 | C5orf37 | ILMN_6243 | -1.62 |
| NM_032574.2 | DPY30 | ILMN_18534 | -1.62 |
| NM_007083.3 | NUDT6 | ILMN_903 | -1.62 |
| NM_016009.2 | SH3GLB1 | ILMN_10394 | -1.62 |
| NM_006022.2 | TSC22D1 | ILMN_26720 | -1.62 |
| NM_003426.2 | ZNF74 | ILMN_13074 | -1.62 |
| NM_005694.1 | COX17 | ILMN_19252 | -1.64 |
| NM_013388.4 | PREB | ILMN_6913 | -1.64 |
| NM_016037.2 | UTP11L | ILMN_2243 | -1.64 |
| NM_138446.1 | C7orf30 | ILMN_14596 | -1.65 |
| NM_005413.1 | SIX3 | ILMN_26476 | -1.65 |
| NM_032376.2 | TMEM101 | ILMN_24128 | -1.65 |
| NM_001382.2 | DPAGT1 | ILMN_10306 | -1.66 |
| NM_015176.1 | FBXO28 | ILMN_22101 | -1.66 |
| NM_017946.2 | FKBP14 | ILMN_18132 | -1.66 |
| NM_184234.1 | RBM39 | ILMN_20330 | -1.66 |
| NM_002897.3 | RBMS1 | ILMN_18726 | -1.66 |
| NM_020905.2 | RDH14 | ILMN_3300 | -1.66 |
| NM_016472.3 | C14orf129 | ILMN_7725 | -1.67 |
| NM_015607.2 | C1orf77 | ILMN_21997 | -1.67 |
| NM_006090.3 | CEPT1 | ILMN_14637 | -1.67 |
| NM_001077394.1 | DPH5 | ILMN_175087 | -1.67 |
| NM_006158.2 | NEFL | ILMN_22054 | -1.67 |
| NM_018130.2 | SHQ1 | ILMN_29855 | -1.67 |
| NM_005589.2 | ALDH6A1 | ILMN_24260 | -1.68 |
| NM_014171.3 | CRIPT | ILMN_12903 | -1.68 |
| NM_002690.1 | POLB | ILMN_15404 | -1.68 |
| NM_001008566.1 | TPST2 | ILMN_13248 | -1.68 |
| NM_134268.3 | CYGB | ILMN_8058 | -1.69 |
| NM_006736.5 | DNAJB2 | ILMN_34421 | -1.69 |
| NM_002158.3 | FOXN2 | ILMN_167513 | -1.69 |
| NM_032316.3 | NICN1 | ILMN_17764 | -1.69 |
| NM_018131.3 | CEP55 | ILMN_6470 | -1.7 |
| NM_199235.1 | COLEC11 | ILMN_6793 | -1.7 |
| NM_031300.2 | MXD3 | ILMN_21984 | -1.7 |
| NM_022830.1 | TUT1 | ILMN_6523 | -1.7 |
| NM_007219.2 | RNF24 | ILMN_16137 | -1.71 |
| NM_022173.1 | TIA1 | ILMN_29910 | -1.71 |
| NM_138358.2 | C19orf52 | ILMN_11158 | -1.72 |
| NM_012123.2 | MTO1 | ILMN_15725 | -1.72 |
| NM_002598.2 | PDCD2 | ILMN_5469 | -1.72 |
| NM_033286.1 | C15orf23 | ILMN_28790 | -1.73 |
| NM_007277.4 | EXOC3 | ILMN_6110 | -1.73 |
| NM_015935.4 | KIAA0859 | ILMN_172647 | -1.73 |
| NM_001031677.2 | RAB24 | ILMN_25731 | -1.73 |
| NM_033117.2 | RBM18 | ILMN_8277 | -1.73 |
| NM_001002246.1 | ANAPC11 | ILMN_5565 | -1.74 |
| NM_014184.2 | CNIH4 | ILMN_9903 | -1.74 |
| NM_178439.3 | GMCL1 | ILMN_3285 | -1.74 |
| XM_001131304.1 | LOC728635 | ILMN_168315 | -1.74 |
| NM_032439.1 | PHYHIPL | ILMN_22045 | -1.74 |
| NM_005644.2 | TAF12 | ILMN_3797 | -1.74 |
| NM_001037174.1 | ARL5A | ILMN_12840 | -1.75 |
| XR_001271.1 | LOC441191 | ILMN_39396 | -1.75 |
| NM_170784.1 | MKKS | ILMN_17701 | -1.75 |
| NM_001042370.1 | TROVE2 | ILMN_173505 | -1.75 |
| NM_153331.2 | KCTD6 | ILMN_15146 | -1.76 |
| NM_001037675.1 | NBPF20 | ILMN_26956 | -1.76 |
| NM_015462.3 | NOL11 | ILMN_5347 | -1.76 |
| NM_005903.5 | SMAD5 | ILMN_8262 | -1.76 |
| NM_014290.1 | TDRD7 | ILMN_27692 | -1.76 |
| NM_006337.3 | MCRS1 | ILMN_176605 | -1.77 |
| NM_017567.2 | NAGK | ILMN_4544 | -1.77 |
| NM_007280.1 | OIP5 | ILMN_18200 | -1.77 |
| NM_016535.3 | ZNF581 | ILMN_13004 | -1.77 |
| NM_148178.1 | C9orf23 | ILMN_3926 | -1.78 |
| NM_025004.1 | CCDC15 | ILMN_4688 | -1.78 |
| NM_152274.2 | FAM58A | ILMN_3352 | -1.78 |
| NM_001085363.1 | MEX3D | ILMN_307190 | -1.78 |
| NM_002896.1 | RBM4 | ILMN_11057 | -1.78 |
| NM_006416.3 | SLC35A1 | ILMN_23284 | -1.78 |
| NM_024063.1 | SPATA5L1 | ILMN_4249 | -1.78 |
| NM_080632.1 | UPF3B | ILMN_174905 | -1.78 |
| NM_145074.2 | HTRA2 | ILMN_12587 | -1.79 |
| NM_180981.1 | MRPL52 | ILMN_3474 | -1.79 |
| NM_145274.2 | TMEM99 | ILMN_25105 | -1.79 |
| NM_001002860.2 | BTBD7 | ILMN_178877 | -1.8 |
| NM_005713.1 | COL4A3BP | ILMN_10635 | -1.8 |
| NM_181528.2 | NAT5 | ILMN_43222 | -1.8 |
| NM_005997.1 | VPS72 | ILMN_17901 | -1.8 |
| NM_198970.1 | AES | ILMN_25198 | -1.81 |
| NM_016355.3 | DDX47 | ILMN_8096 | -1.81 |
| NM_021800.2 | DNAJC12 | ILMN_177844 | -1.81 |
| NM_019037.2 | EXOSC4 | ILMN_25178 | -1.81 |
| NM_017850.1 | C1orf109 | ILMN_27592 | -1.82 |
| NM_033212.2 | CCDC102A | ILMN_12942 | -1.82 |
| NM_018094.2 | GSPT2 | ILMN_20472 | -1.82 |
| NM_003628.3 | PKP4 | ILMN_11784 | -1.82 |
| NM_000051.3 | ATM | ILMN_162851 | -1.83 |
| NM_032847.1 | C8orf76 | ILMN_8743 | -1.83 |
| NM_018062.2 | FANCL | ILMN_24728 | -1.83 |
| NM_001031706.1 | PLEKHB2 | ILMN_179121 | -1.83 |
| NM_021244.3 | RRAGD | ILMN_5663 | -1.83 |
| NM_004865.2 | TBPL1 | ILMN_3787 | -1.83 |
| NM_019116.2 | UBFD1 | ILMN_179383 | -1.83 |
| NM_024945.2 | RMI1 | ILMN_11713 | -1.84 |
| NM_031287.2 | SF3B5 | ILMN_20062 | -1.84 |
| NM_031905.2 | ARMC10 | ILMN_171553 | -1.85 |
| NM_004156.2 | PPP2CB | ILMN_21592 | -1.85 |
| NM_014254.1 | TMEM5 | ILMN_26271 | -1.85 |
| NM_005749.2 | TOB1 | ILMN_13735 | -1.85 |
| NM_016561.1 | BFAR | ILMN_23440 | -1.86 |
| NM_003824.2 | FADD | ILMN_11407 | -1.86 |
| NM_014458.3 | KLHL20 | ILMN_11595 | -1.86 |
| NM_032479.2 | MRPL36 | ILMN_22209 | -1.86 |
| NM_194326.2 | RPS19BP1 | ILMN_8107 | -1.86 |
| NM_014305.2 | TGDS | ILMN_30985 | -1.86 |
| NM_030771.1 | CCDC34 | ILMN_2645 | -1.87 |
| NM_020234.4 | DTWD1 | ILMN_3248 | -1.87 |
| NM_182972.2 | IRF2BP2 | ILMN_5645 | -1.87 |
| NM_015634.2 | KIAA1279 | ILMN_8497 | -1.87 |
| NM_020345.3 | NKIRAS1 | ILMN_28229 | -1.87 |
| NM_001031684.1 | SFRS7 | ILMN_7620 | -1.87 |
| NM_001018020.1 | TPM1 | ILMN_14091 | -1.87 |
| NM_015994.2 | ATP6V1D | ILMN_26737 | -1.88 |
| NM_001042549.1 | NSL1 | ILMN_164300 | -1.88 |
| NM_013328.2 | PYCR2 | ILMN_18209 | -1.88 |
| NM_015523.2 | REXO2 | ILMN_15016 | -1.88 |
| NM_017612.2 | ZCCHC8 | ILMN_30318 | -1.88 |
| NM_198436.1 | AURKA | ILMN_13382 | -1.89 |
| NM_001896.2 | CSNK2A2 | ILMN_16798 | -1.89 |
| XM_001126750.1 | ABI2 | ILMN_162431 | -1.9 |
| NM_001918.2 | DBT | ILMN_169961 | -1.9 |
| NM_014793.3 | LCMT2 | ILMN_183463 | -1.9 |
| NM_024546.3 | RNF219 | ILMN_38012 | -1.9 |
| NM_006282.2 | STK4 | ILMN_21491 | -1.9 |
| NM_004623.2 | TTC4 | ILMN_6668 | -1.9 |
| NM_023010.2 | UPF3B | ILMN_28844 | -1.9 |
| NM_133646.2 | ZAK | ILMN_5666 | -1.9 |
| NM_017704.2 | ANKRD49 | ILMN_21580 | -1.91 |
| NM_212552.2 | BOLA3 | ILMN_28776 | -1.91 |
| NM_199044.2 | NSUN4 | ILMN_23916 | -1.91 |
| NM_006745.3 | SC4MOL | ILMN_2770 | -1.91 |
| NM_018991.2 | STAG3L1 | ILMN_28376 | -1.91 |
| NM_170695.2 | TGIF1 | ILMN_162784 | -1.91 |
| NM_022037.1 | TIA1 | ILMN_30157 | -1.91 |
| NM_033286.2 | C15orf23 | ILMN_28790 | -1.92 |
| NM_198527.2 | HDDC3 | ILMN_29602 | -1.92 |
| NM_017816.1 | LYAR | ILMN_23200 | -1.92 |
| NM_007342.1 | NUPL2 | ILMN_2154 | -1.92 |
| NM_002553.2 | ORC5L | ILMN_6212 | -1.92 |
| NM_173647.2 | RNF149 | ILMN_10320 | -1.93 |
| NM_145644.1 | MRPL35 | ILMN_20736 | -1.94 |
| NM_018364.3 | RSBN1 | ILMN_174594 | -1.94 |
| NM_012210.3 | TRIM32 | ILMN_14426 | -1.94 |
| NM_024095.3 | ASB8 | ILMN_165486 | -1.95 |
| NM_198391.1 | FLRT3 | ILMN_23273 | -1.95 |
| NM_006597.3 | HSPA8 | ILMN_181529 | -1.95 |
| NM_019006.2 | ZFAND6 | ILMN_16822 | -1.95 |
| NM_024775.9 | GEMIN6 | ILMN_23187 | -1.96 |
| NM_021971.1 | GMPPB | ILMN_3929 | -1.96 |
| NM_201280.1 | MUTED | ILMN_21576 | -1.96 |
| NM_182533.1 | C1orf86 | ILMN_2880 | -1.97 |
| NM_003584.1 | DUSP11 | ILMN_3801 | -1.97 |
| NM_138720.1 | HIST1H2BD | ILMN_17622 | -1.97 |
| NM_005810.3 | KLRG1 | ILMN_12613 | -1.97 |
| NM_003452.2 | ZNF189 | ILMN_4798 | -1.97 |
| NM_022079.2 | HERC4 | ILMN_8869 | -1.98 |
| NM_207350.1 | MGC72104 | ILMN_26269 | -1.98 |
| NM_032346.1 | PDCD2L | ILMN_25365 | -1.98 |
| NM_012460.2 | TIMM9 | ILMN_9968 | -1.98 |
| NM_021622.3 | PLEKHA1 | ILMN_9430 | -1.99 |
| NM_006860.2 | RABL4 | ILMN_4559 | -1.99 |
| NM_001012413.1 | SGOL1 | ILMN_14464 | -1.99 |
| NM_152524.3 | SGOL2 | ILMN_743 | -1.99 |
| NM_145697.1 | CDCA1 | ILMN_17725 | -2 |
| NM_005830.2 | MRPS31 | ILMN_6293 | -2 |
| NM_153018.2 | ZFP3 | ILMN_42182 | -2 |
| NM_007369.2 | GPR161 | ILMN_22837 | -2.01 |
| NM_012475.4 | USP21 | ILMN_18019 | -2.01 |
| NM_006963.3 | ZNF22 | ILMN_165495 | -2.01 |
| NM_001035505.1 | BOLA3 | ILMN_29223 | -2.02 |
| NM_020640.2 | DCUN1D1 | ILMN_410 | -2.02 |
| NM_017446.3 | MRPL39 | ILMN_4651 | -2.02 |
| NM_058216.1 | RAD51C | ILMN_2944 | -2.02 |
| NM_007308.1 | SNCA | ILMN_2235 | -2.02 |
| NM_021800.2 | DNAJC12 | ILMN_15911 | -2.03 |
| NM_003746.1 | DNCL1 | ILMN_137049 | -2.03 |
| NM_005402.2 | RALA | ILMN_164730 | -2.03 |
| NM_001514.3 | GTF2B | ILMN_544 | -2.04 |
| NM_000456.2 | SUOX | ILMN_25551 | -2.04 |
| NM_198401.2 | ANKRD46 | ILMN_9031 | -2.05 |
| NM_014177.1 | C18orf55 | ILMN_9697 | -2.05 |
| NM_001752.2 | CAT | ILMN_13962 | -2.05 |
| NM_024011.2 | CDC2L2 | ILMN_20434 | -2.05 |
| NM_012110.2 | CHIC2 | ILMN_24345 | -2.06 |
| NM_019095.3 | CRLS1 | ILMN_14031 | -2.06 |
| NM_032320.5 | BTBD10 | ILMN_30066 | -2.07 |
| NM_005513.1 | GTF2E1 | ILMN_175401 | -2.07 |
| NM_153713.1 | LIX1L | ILMN_3572 | -2.07 |
| NM_032323.1 | TMEM79 | ILMN_13555 | -2.07 |
| XM_374020.4 | LOC375295 | ILMN_45377 | -2.08 |
| NM_007342.1 | NUPL2 | ILMN_2154 | -2.08 |
| NM_002613.3 | PDPK1 | ILMN_27765 | -2.08 |
| NM_005398.4 | PPP1R3C | ILMN_4487 | -2.08 |
| NM_014620.4 | HOXC4 | ILMN_16005 | -2.09 |
| XM_926112.2 | LOC441155 | ILMN_37470 | -2.09 |
| NM_006281.2 | STK3 | ILMN_26935 | -2.1 |
| NM_014294.4 | TRAM1 | ILMN_3604 | -2.11 |
| NM_024071.2 | ZFYVE21 | ILMN_1317 | -2.11 |
| NM_012241.2 | SIRT5 | ILMN_18454 | -2.12 |
| NM_006299.3 | ZNF193 | ILMN_10151 | -2.12 |
| NM_174942.1 | GAS2L3 | ILMN_5609 | -2.13 |
| NM_004885.1 | NPFFR2 | ILMN_20676 | -2.13 |
| NM_006265.1 | RAD21 | ILMN_171453 | -2.13 |
| NM_152360.2 | ZNF573 | ILMN_23003 | -2.13 |
| NM_018343.1 | RIOK2 | ILMN_16482 | -2.15 |
| NM_001040142.1 | SCN2A | ILMN_167124 | -2.15 |
| NM_016374.5 | ARID4B | ILMN_162934 | -2.16 |
| NM_138285.3 | NUP35 | ILMN_17545 | -2.16 |
| NM_018480.2 | TMEM126B | ILMN_18826 | -2.16 |
| NM_001007278.1 | TRIM13 | ILMN_14225 | -2.16 |
| NM_152415.1 | VPS37A | ILMN_12702 | -2.16 |
| NM_001012756.1 | ZNF260 | ILMN_172733 | -2.16 |
| NM_001097599.1 | TMEM22 | ILMN_306942 | -2.18 |
| NM_003998.2 | NFKB1 | ILMN_161884 | -2.19 |
| NM_001006622.1 | WDR33 | ILMN_6581 | -2.2 |
| NM_032530.1 | ZNF594 | ILMN_309021 | -2.2 |
| NM_002095.4 | GTF2E2 | ILMN_4316 | -2.21 |
| NM_013316.2 | CNOT4 | ILMN_22777 | -2.22 |
| NM_025205.3 | MED28 | ILMN_14574 | -2.22 |
| NM_014755.1 | SERTAD2 | ILMN_4347 | -2.22 |
| NM_178439.3 | GMCL1 | ILMN_3285 | -2.24 |
| NM_000628.3 | IL10RB | ILMN_26097 | -2.24 |
| NM_183399.1 | RNF14 | ILMN_7292 | -2.29 |
| NM_001827.1 | CKS2 | ILMN_14702 | -2.3 |
| NM_016042.2 | EXOSC3 | ILMN_174330 | -2.3 |
| NM_000856.3 | GUCY1A3 | ILMN_11680 | -2.31 |
| NM_177983.1 | PPM1G | ILMN_878 | -2.31 |
| NM_000819.3 | GART | ILMN_22974 | -2.32 |
| NM_153374.1 | LYSMD2 | ILMN_19682 | -2.32 |
| NM_014941.1 | MORC2 | ILMN_12502 | -2.32 |
| NM_004428.2 | EFNA1 | ILMN_14320 | -2.33 |
| NM_020230.4 | PPAN | ILMN_25948 | -2.33 |
| NM_003729.2 | RTCD1 | ILMN_11697 | -2.34 |
| NM_001033503.1 | SAR1B | ILMN_16595 | -2.34 |
| NM_005836.2 | HRSP12 | ILMN_8062 | -2.35 |
| NM_001002019.1 | PUS1 | ILMN_13055 | -2.35 |
| NM_001042426.1 | CENPA | ILMN_180589 | -2.36 |
| NM_182919.1 | TICAM1 | ILMN_11434 | -2.36 |
| NM_145247.4 | C10orf78 | ILMN_1251 | -2.37 |
| NM_080651.1 | MED30 | ILMN_7158 | -2.37 |
| NM_078629.1 | MSL3L1 | ILMN_29354 | -2.37 |
| NM_001539.2 | DNAJA1 | ILMN_5819 | -2.38 |
| NM_024516.2 | C16orf53 | ILMN_20272 | -2.39 |
| NM_019083.1 | CCDC76 | ILMN_2869 | -2.39 |
| NM_014322.2 | OPN3 | ILMN_166169 | -2.39 |
| NM_018473.2 | THEM2 | ILMN_27212 | -2.39 |
| NM_170783.1 | ZNRD1 | ILMN_1419 | -2.39 |
| NM_198434.1 | AURKA | ILMN_12352 | -2.4 |
| NM_001014286.2 | FAM48A | ILMN_1616 | -2.4 |
| NM_138720.1 | HIST1H2BD | ILMN_17622 | -2.4 |
| NM_001007157.1 | PHF14 | ILMN_2096 | -2.4 |
| NM_170783.1 | ZNRD1 | ILMN_1419 | -2.42 |
| NM_015957.1 | APIP | ILMN_15379 | -2.43 |
| NM_001042426.1 | CENPA | ILMN_180589 | -2.43 |
| NM_138484.2 | SGOL1 | ILMN_14008 | -2.43 |
| NM_018390.2 | PLCXD1 | ILMN_8273 | -2.44 |
| NM_001394.5 | DUSP4 | ILMN_17730 | -2.45 |
| NM_014161.2 | MRPL18 | ILMN_14120 | -2.45 |
| NM_018983.3 | NOLA1 | ILMN_14204 | -2.46 |
| NM_030917.2 | FIP1L1 | ILMN_6961 | -2.48 |
| NM_001382.2 | DPAGT1 | ILMN_10306 | -2.49 |
| NM_005926.2 | MFAP1 | ILMN_20656 | -2.49 |
| NM_004503.3 | HOXC6 | ILMN_15669 | -2.54 |
| NM_018357.2 | LARP6 | ILMN_25584 | -2.54 |
| NM_007198.2 | PROSC | ILMN_23472 | -2.56 |
| NM_001827.1 | CKS2 | ILMN_14702 | -2.57 |
| NM_138798.1 | MITD1 | ILMN_27516 | -2.58 |
| NM_012170.2 | FBXO22 | ILMN_5718 | -2.6 |
| NM_001037163.1 | MGC12966 | ILMN_182436 | -2.66 |
| NM_138316.2 | PANK1 | ILMN_406 | -2.66 |
| NM_019058.2 | DDIT4 | ILMN_13176 | -2.68 |
| NM_003512.3 | HIST1H2AC | ILMN_26493 | -2.68 |
| NM_181708.1 | BCDIN3D | ILMN_18065 | -2.7 |
| NM_199229.1 | RPE | ILMN_9823 | -2.7 |
| NM_005681.2 | TAF1A | ILMN_8114 | -2.7 |
| NM_032280.1 | ZCCHC9 | ILMN_25119 | -2.7 |
| NM_022893.2 | BCL11A | ILMN_17359 | -2.71 |
| NM_001040708.1 | HEY1 | ILMN_164416 | -2.71 |
| NM_001007230.1 | SPOP | ILMN_12838 | -2.73 |
| NM_020749.3 | MTUS1 | ILMN_4658 | -2.77 |
| NM_003342.4 | UBE2G1 | ILMN_179729 | -2.78 |
| NR_001588.1 | SBDSP | ILMN_12233 | -2.79 |
| NM_079837.2 | BANP | ILMN_8638 | -2.8 |
| NM_015948.2 | SLC35B3 | ILMN_20545 | -2.8 |
| NM_020147.2 | THAP10 | ILMN_182683 | -2.8 |
| NM_030808.3 | NDEL1 | ILMN_20362 | -2.85 |
| NM_173510.1 | CCDC117 | ILMN_21814 | -2.86 |
| NM_003542.3 | HIST1H4C | ILMN_30043 | -2.86 |
| NM_024585.2 | ARMC7 | ILMN_163623 | -2.88 |
| NM_020892.1 | DTX2 | ILMN_21612 | -2.94 |
| NM_015942.3 | MTERFD1 | ILMN_24756 | -2.96 |
| NM_020799.2 | STAMBPL1 | ILMN_1387 | -2.97 |
| NM_006630.1 | ZNF234 | ILMN_29233 | -2.98 |
| NM_001634.4 | AMD1 | ILMN_21529 | -3 |
| NM_014487.3 | ZNF330 | ILMN_6878 | -3.07 |
| NM_014596.4 | ZNRD1 | ILMN_20009 | -3.08 |
| NM_016374.5 | ARID4B | ILMN_162934 | -3.1 |
| NM_004456.3 | EZH2 | ILMN_25740 | -3.11 |
| NM_024057.2 | NUP37 | ILMN_4147 | -3.13 |
| NM_006630.1 | ZNF234 | ILMN_29233 | -3.31 |
| NM_017816.1 | LYAR | ILMN_23200 | -3.33 |
| NM_015942.3 | MTERFD1 | ILMN_174209 | -3.35 |
| NM_004316.2 | ASCL1 | ILMN_23892 | -3.41 |
| NR_001445.1 | RN7SK | ILMN_14457 | -3.41 |
| NM_033091.1 | TRIM4 | ILMN_8530 | -3.44 |
| NM_181702.1 | GEM | ILMN_16170 | -3.46 |
| NM_181702.1 | GEM | ILMN_16170 | -3.48 |
| NM_001039937.1 | INTS6 | ILMN_38649 | -3.74 |
| NM_006145.1 | DNAJB1 | ILMN_19740 | -3.78 |
| NM_007167.2 | ZMYM6 | ILMN_1275 | -3.82 |
| NM_005345.4 | HSPA1A | ILMN_6623 | -4.04 |
| NM_016277.3 | RAB23 | ILMN_177407 | -4.09 |
| NM_016042.2 | EXOSC3 | ILMN_174330 | -4.4 |
| NR_001449.1 | TRK1 | ILMN_6493 | -4.4 |
| NM_005346.3 | HSPA1B | ILMN_25549 | -6.39 |
