# Supplementary material for: Minimal Peroxide Exposure of Neuronal Cells Induces Multifaceted Adaptive Responses
Source: PLoS One. 2010 Dec 17;5(12):e14352. doi: 10.1371/journal.pone.0014352 (PMC3003681; doi:10.1371/journal.pone.0014352)
Supplement: Table S10 — BDNF-significantly regulated genes after 4 hours of stimulation in the control state SH-SY5Y cells. Each significantly regulated gene is described via its accession number (ACCESSION), Gene Symbol (SYMBOL), Illumina array transcript designation (TRANSCRIPT). For each gene the z-ratio of expression compared to untreated cells after 4 hours of ligand stimulation is displayed (CTL BDNF 4). (1.23 MB DOC) [file pone.0014352.s017.doc]

**Table S10. BDNF-significantly regulated genes after 4 hours of stimulation in the control state SH-SY5Y cells**. Each significantly regulated gene is described via its accession number (ACCESSION), Gene Symbol (SYMBOL), Illumina array transcript designation (TRANSCRIPT). For each gene the z-ratio of expression compared to un-treated cells after 4 hours of ligand stimulation is displayed (CTL BDNF 4).

| **ACCESSION** | **SYMBOL** | **TRANSCRIPT** | **CTL BDNF 4** |
| --- | --- | --- | --- |
| NM_000584.2 | IL8 | ILMN_179575 | 7.14 |
| XM_935588.1 | LOC641848 | ILMN_45490 | 5.54 |
| NM_005324.3 | H3F3B | ILMN_26885 | 5.42 |
| NM_001003.2 | RPLP1 | ILMN_23181 | 5.38 |
| NR_002315.1 | LOC440926 | ILMN_19720 | 5.18 |
| NM_001099285.1 | PTMA | ILMN_306831 | 4.77 |
| NM_001024646.1 | CLK1 | ILMN_27286 | 4.74 |
| XR_017492.1 | LOC644330 | ILMN_164787 | 4.74 |
| NM_001033506.1 | CSTF3 | ILMN_27049 | 4.72 |
| NM_016028.4 | SUV420H1 | ILMN_29861 | 4.68 |
| NM_006135.1 | CAPZA1 | ILMN_137637 | 4.52 |
| NM_148957.2 | TNFRSF19 | ILMN_28684 | 4.45 |
| XM_944439.2 | LOC653994 | ILMN_38572 | 4.32 |
| XM_370865.4 | LOC388122 | ILMN_46143 | 4.29 |
| NM_006123.2 | IDS | ILMN_17605 | 4.23 |
| NM_004768.2 | SFRS11 | ILMN_4847 | 4.11 |
| NM_012322.1 | LSM5 | ILMN_17896 | 4.05 |
| NM_014817.3 | KIAA0644 | ILMN_164846 | 3.97 |
| NR_003040.1 | LOC649946 | ILMN_169528 | 3.97 |
| XM_938089.2 | LOC643007 | ILMN_31054 | 3.96 |
| NM_001024921.2 | RPL9 | ILMN_8640 | 3.95 |
| XR_015514.1 | LOC730746 | ILMN_163533 | 3.91 |
| XM_926231.1 | P704P | ILMN_36679 | 3.83 |
| NM_148174.2 | AZIN1 | ILMN_4931 | 3.82 |
| NM_004038.3 | AMY1A | ILMN_176350 | 3.82 |
| NM_002291.1 | LAMB1 | ILMN_182874 | 3.81 |
| NM_007285.6 | GABARAPL2 | ILMN_9805 | 3.78 |
| NM_001035005.2 | C18orf32 | ILMN_26126 | 3.78 |
| NM_181054.1 | HIF1A | ILMN_9514 | 3.78 |
| NM_024629.2 | MLF1IP | ILMN_16700 | 3.68 |
| XM_375152.3 | LOC400304 | ILMN_46003 | 3.68 |
| NM_004859.3 | CLTC | ILMN_171089 | 3.67 |
| XM_937113.2 | LOC647436 | ILMN_44829 | 3.67 |
| XM_933893.1 | LOC389672 | ILMN_35589 | 3.66 |
| XR_018327.1 | LOC648343 | ILMN_163789 | 3.65 |
| NM_030793.3 | FBXO38 | ILMN_4373 | 3.63 |
| NM_001412.3 | EIF1AX | ILMN_22164 | 3.58 |
| NR_002201.1 | FTHL3 | ILMN_27691 | 3.58 |
| NM_183422.1 | TSC22D1 | ILMN_166165 | 3.51 |
| NM_002874.3 | RAD23B | ILMN_19346 | 3.51 |
| XM_941684.2 | LOC220433 | ILMN_46655 | 3.51 |
| NM_000617.1 | SLC11A2 | ILMN_10129 | 3.49 |
| XM_936731.1 | LOC647673 | ILMN_33594 | 3.47 |
| NM_001008735.1 | HMG1L1 | ILMN_22757 | 3.44 |
| NM_004156.2 | PPP2CB | ILMN_21592 | 3.43 |
| NR_002204.1 | FTHL11 | ILMN_16343 | 3.41 |
| NM_033138.2 | CALD1 | ILMN_29896 | 3.39 |
| XM_292963.6 | LOC643997 | ILMN_39721 | 3.38 |
| XR_016048.1 | MGC40489 | ILMN_171153 | 3.37 |
| NM_001010915.1 | PTPLAD2 | ILMN_6355 | 3.36 |
| XM_938297.1 | LOC402644 | ILMN_30715 | 3.35 |
| NM_002673.3 | PLXNB1 | ILMN_22628 | 3.33 |
| NM_002266.2 | KPNA2 | ILMN_14206 | 3.33 |
| NM_003082.2 | SNAPC1 | ILMN_177713 | 3.29 |
| NM_018697.3 | LANCL2 | ILMN_920 | 3.29 |
| XM_940333.2 | LOC651202 | ILMN_37363 | 3.29 |
| NM_153188.2 | TNPO1 | ILMN_29083 | 3.28 |
| NM_199436.1 | SPAST | ILMN_15461 | 3.24 |
| NM_012215.2 | MGEA5 | ILMN_11399 | 3.24 |
| NM_001034996.1 | RPL14 | ILMN_2719 | 3.21 |
| NM_080386.1 | TUBA3D | ILMN_30319 | 3.18 |
| NM_006948.4 | STCH | ILMN_163590 | 3.17 |
| NM_014363.3 | SACS | ILMN_180142 | 3.17 |
| NM_003418.1 | CNBP | ILMN_9092 | 3.14 |
| NR_003277.1 | LOC728643 | ILMN_183126 | 3.14 |
| XM_935770.1 | LOC641992 | ILMN_31870 | 3.13 |
| XM_937928.1 | LOC347376 | ILMN_31523 | 3.12 |
| XM_937850.1 | LOC285176 | ILMN_43277 | 3.11 |
| NM_001031827.1 | BOLA2 | ILMN_4509 | 3.08 |
| NM_175923.3 | MGC42630 | ILMN_138411 | 3.07 |
| NM_016608.1 | ARMCX1 | ILMN_9172 | 3.06 |
| NM_080491.1 | GAB2 | ILMN_3317 | 3.06 |
| NM_001008237.1 | TTC32 | ILMN_4829 | 3.05 |
| XM_928275.1 | LOC645236 | ILMN_36666 | 3.05 |
| XM_938988.1 | LOC402221 | ILMN_35678 | 3.05 |
| NM_020409.2 | MRPL47 | ILMN_12272 | 3.04 |
| NM_017821.3 | RHBDL2 | ILMN_20003 | 3.04 |
| NM_005245.3 | FAT | ILMN_24617 | 3.02 |
| XM_930178.1 | LOC645018 | ILMN_33646 | 3.01 |
| NM_178324.1 | SPTLC1 | ILMN_7889 | 2.99 |
| XR_017397.1 | LOC644029 | ILMN_163901 | 2.98 |
| XR_019339.1 | LOC643668 | ILMN_179350 | 2.97 |
| NR_002203.1 | FTHL8 | ILMN_16227 | 2.97 |
| XM_941195.2 | LOC388621 | ILMN_42661 | 2.96 |
| XM_932717.2 | LOC643224 | ILMN_34053 | 2.92 |
| NM_006004.1 | UQCRH | ILMN_138507 | 2.92 |
| NM_001008237.1 | TTC32 | ILMN_4829 | 2.9 |
| XM_929199.1 | LOC644250 | ILMN_30796 | 2.9 |
| XM_945045.1 | LOC649679 | ILMN_34833 | 2.89 |
| NM_002129.2 | HMGB2 | ILMN_3200 | 2.89 |
| NM_004075.2 | CRY1 | ILMN_6263 | 2.88 |
| NM_172014.1 | TNFSF14 | ILMN_9666 | 2.88 |
| NM_001005849.1 | SUMO2 | ILMN_16713 | 2.88 |
| XR_015809.1 | LOC728973 | ILMN_168278 | 2.87 |
| NR_002200.1 | FTHL2 | ILMN_15867 | 2.86 |
| NR_002205.1 | FTHL12 | ILMN_16447 | 2.86 |
| NM_001006115.2 | IHPK1 | ILMN_8379 | 2.85 |
| NM_001017998.2 | GNG10 | ILMN_34149 | 2.84 |
| NM_006472.2 | TXNIP | ILMN_9057 | 2.84 |
| XM_944104.2 | LOC653232 | ILMN_41197 | 2.84 |
| NM_001080453.1 | INTS1 | ILMN_173681 | 2.83 |
| NM_003086.2 | SNAPC4 | ILMN_180505 | 2.83 |
| NM_001030288.1 | SPN | ILMN_18556 | 2.82 |
| NM_145913.2 | SLC5A8 | ILMN_7082 | 2.82 |
| NM_015130.2 | TBC1D9 | ILMN_25527 | 2.82 |
| NM_006107.2 | CROP | ILMN_10300 | 2.82 |
| NM_002128.4 | HMGB1 | ILMN_23421 | 2.81 |
| NM_005154.2 | USP8 | ILMN_15425 | 2.8 |
| NM_172249.1 | CSF2RA | ILMN_5061 | 2.8 |
| NM_003878.1 | GGH | ILMN_9870 | 2.79 |
| NM_020704.1 | FAM40B | ILMN_18452 | 2.78 |
| NM_013361.3 | ZNF223 | ILMN_166150 | 2.78 |
| NM_031372.1 | HNRPDL | ILMN_15196 | 2.78 |
| NM_005443.4 | PAPSS1 | ILMN_171260 | 2.76 |
| NM_004075.2 | CRY1 | ILMN_6263 | 2.76 |
| NM_173666.1 | DTWD2 | ILMN_25915 | 2.75 |
| NM_006265.1 | RAD21 | ILMN_171453 | 2.75 |
| NM_001012626.1 | LOC285074 | ILMN_21153 | 2.74 |
| NM_001251.2 | CD68 | ILMN_5188 | 2.74 |
| NM_201555.1 | FHL2 | ILMN_21541 | 2.73 |
| NM_014478.4 | RCP9 | ILMN_22485 | 2.73 |
| NM_031943.1 | IFP38 | ILMN_9478 | 2.72 |
| NM_012256.2 | ZNF212 | ILMN_14026 | 2.72 |
| NM_012400.2 | PLA2G2D | ILMN_163941 | 2.71 |
| NM_033083.6 | EAF1 | ILMN_173601 | 2.71 |
| NM_004788.2 | UBE4A | ILMN_175730 | 2.71 |
| NM_178231.1 | ALS2CR14 | ILMN_947 | 2.7 |
| NM_001788.4 | SEPT7 | ILMN_25070 | 2.7 |
| NM_018479.2 | ECHDC1 | ILMN_1493 | 2.69 |
| NM_001080484.1 | KIAA1751 | ILMN_180591 | 2.69 |
| NM_020724.1 | RNF150 | ILMN_26801 | 2.68 |
| NM_173518.2 | C8orf45 | ILMN_22241 | 2.68 |
| XM_001133089.1 | LOC731640 | ILMN_161930 | 2.68 |
| NM_144736.3 | PRO1853 | ILMN_15591 | 2.67 |
| NR_002182.1 | NACAP1 | ILMN_14666 | 2.67 |
| XM_497072.2 | LOC389787 | ILMN_45784 | 2.67 |
| NM_004896.2 | VPS26 | ILMN_137556 | 2.66 |
| XM_942687.1 | LOC654189 | ILMN_30702 | 2.66 |
| NM_002687.3 | PNN | ILMN_24088 | 2.66 |
| NM_006572.3 | GNA13 | ILMN_173569 | 2.66 |
| NM_014000.2 | VCL | ILMN_27566 | 2.66 |
| NM_015447.1 | CAMSAP1 | ILMN_815 | 2.65 |
| XM_938599.2 | LOC441377 | ILMN_31681 | 2.64 |
| NM_000046.2 | ARSB | ILMN_180341 | 2.63 |
| NM_013412.1 | RABL2A | ILMN_12484 | 2.63 |
| XM_944716.1 | LOC440704 | ILMN_34488 | 2.63 |
| NM_003617.2 | RGS5 | ILMN_167992 | 2.63 |
| XM_941155.2 | LOC651894 | ILMN_33374 | 2.62 |
| NM_018561.3 | USP49 | ILMN_24018 | 2.62 |
| NM_024663.3 | NPEPL1 | ILMN_175218 | 2.61 |
| NM_001077628.1 | APH1A | ILMN_180233 | 2.61 |
| NM_003972.2 | BTAF1 | ILMN_8616 | 2.61 |
| NM_002213.3 | ITGB5 | ILMN_24189 | 2.6 |
| NM_000971.3 | RPL7 | ILMN_26351 | 2.59 |
| NM_006806.3 | BTG3 | ILMN_27215 | 2.58 |
| NM_001040456.1 | RHBDD2 | ILMN_168345 | 2.58 |
| NM_000978.3 | RPL23 | ILMN_8866 | 2.58 |
| NM_000572.2 | IL10 | ILMN_9173 | 2.57 |
| NM_006275.4 | SFRS6 | ILMN_24964 | 2.57 |
| NR_002204.1 | FTHL11 | ILMN_16343 | 2.56 |
| NM_003666.2 | BLZF1 | ILMN_21927 | 2.56 |
| NM_152730.4 | C6orf170 | ILMN_17001 | 2.54 |
| NM_033285.2 | TP53INP1 | ILMN_16203 | 2.54 |
| NM_176811.2 | NLRP8 | ILMN_169055 | 2.54 |
| NM_032239.2 | LARP2 | ILMN_9962 | 2.53 |
| NM_032221.3 | CHD6 | ILMN_174095 | 2.52 |
| NR_002197.1 | LOC143543 | ILMN_17694 | 2.52 |
| NM_152322.2 | BTBD11 | ILMN_506 | 2.52 |
| NR_002205.1 | FTHL12 | ILMN_16447 | 2.51 |
| NM_001025780.1 | FAM108B1 | ILMN_181005 | 2.5 |
| NM_020317.3 | C1orf63 | ILMN_22487 | 2.5 |
| XM_294473.2 | LOC346950 | ILMN_36919 | 2.5 |
| NM_001040456.1 | RHBDD2 | ILMN_168345 | 2.5 |
| NM_021190.1 | PTBP2 | ILMN_556 | 2.49 |
| NM_021227.2 | DC2 | ILMN_24748 | 2.49 |
| NM_152679.2 | SLC10A4 | ILMN_1323 | 2.49 |
| NR_001562.1 | ANXA2P1 | ILMN_10494 | 2.48 |
| NM_025152.1 | NUBPL | ILMN_25397 | 2.48 |
| NM_002566.4 | P2RY11 | ILMN_12237 | 2.47 |
| NM_152398.2 | OCIAD2 | ILMN_18246 | 2.47 |
| NM_001080973.1 | IL17RD | ILMN_179882 | 2.47 |
| XR_018923.1 | LOC648210 | ILMN_162972 | 2.47 |
| XM_935589.1 | LOC641849 | ILMN_45563 | 2.46 |
| NM_006372.3 | SYNCRIP | ILMN_28470 | 2.46 |
| NM_003220.2 | TFAP2A | ILMN_17128 | 2.45 |
| NM_024561.3 | NARG1L | ILMN_22547 | 2.45 |
| NM_014014.2 | ASCC3L1 | ILMN_18834 | 2.45 |
| NM_017741.3 | C4orf30 | ILMN_172318 | 2.45 |
| XM_938755.2 | LOC653773 | ILMN_44662 | 2.45 |
| NM_032796.2 | SYAP1 | ILMN_8037 | 2.44 |
| NM_005128.2 | DOPEY2 | ILMN_164626 | 2.43 |
| NM_019119.3 | PCDHB9 | ILMN_23442 | 2.43 |
| NM_018204.2 | CKAP2 | ILMN_168115 | 2.43 |
| NM_014717.1 | ZNF536 | ILMN_179125 | 2.42 |
| NM_000230.1 | LEP | ILMN_10827 | 2.41 |
| NM_016038.2 | SBDS | ILMN_15766 | 2.41 |
| NM_001008393.1 | LOC201725 | ILMN_20795 | 2.4 |
| NM_001031623.2 | ZNF451 | ILMN_990 | 2.39 |
| NM_000587.2 | C7 | ILMN_15063 | 2.39 |
| NM_022459.3 | XPO4 | ILMN_164187 | 2.38 |
| NM_016570.2 | ERGIC2 | ILMN_24083 | 2.38 |
| NM_133459.1 | CCBE1 | ILMN_6075 | 2.38 |
| XM_933970.1 | LOC646849 | ILMN_31668 | 2.38 |
| NM_001095.2 | ACCN2 | ILMN_27416 | 2.37 |
| NM_017635.3 | SUV420H1 | ILMN_174505 | 2.37 |
| NM_001080.3 | ALDH5A1 | ILMN_1025 | 2.37 |
| NR_002448.1 | SNORD36A | ILMN_20691 | 2.37 |
| NM_001089.1 | ABCA3 | ILMN_18800 | 2.37 |
| NM_014056.1 | HIGD1A | ILMN_2731 | 2.37 |
| XM_934113.1 | LOC653489 | ILMN_42664 | 2.37 |
| NM_006360.3 | EIF3M | ILMN_19862 | 2.37 |
| NR_002939.2 | RUNDC2C | ILMN_39843 | 2.36 |
| XM_935802.1 | LOC653829 | ILMN_46774 | 2.36 |
| NM_003677.3 | DENR | ILMN_181187 | 2.35 |
| NM_134265.2 | WSB1 | ILMN_5396 | 2.35 |
| NM_014395.1 | DAPP1 | ILMN_24094 | 2.35 |
| NM_001001391.1 | CD44 | ILMN_10947 | 2.34 |
| NM_016623.3 | FAM49B | ILMN_14248 | 2.34 |
| XM_930995.1 | LOC653086 | ILMN_31021 | 2.34 |
| XM_001134259.1 | LOC732165 | ILMN_170212 | 2.32 |
| NM_020755.2 | SERINC1 | ILMN_24825 | 2.32 |
| NM_182492.1 | LRP5L | ILMN_650 | 2.31 |
| NM_003461.4 | ZYX | ILMN_2137 | 2.31 |
| XM_940610.1 | LOC651453 | ILMN_32585 | 2.3 |
| NM_005721.3 | ACTR3 | ILMN_11792 | 2.3 |
| NM_018639.3 | WSB2 | ILMN_162438 | 2.29 |
| NM_002923.1 | RGS2 | ILMN_26119 | 2.29 |
| NM_018698.3 | NXT2 | ILMN_168294 | 2.28 |
| NM_021090.3 | MTMR3 | ILMN_27578 | 2.28 |
| NM_016114.3 | ASB1 | ILMN_11707 | 2.27 |
| NM_198156.1 | VHL | ILMN_21388 | 2.27 |
| NM_032440.1 | LCOR | ILMN_173510 | 2.26 |
| NM_006628.4 | ARPP-19 | ILMN_2093 | 2.26 |
| XM_939726.2 | LOC388532 | ILMN_45940 | 2.26 |
| NM_033419.3 | PERLD1 | ILMN_12215 | 2.25 |
| NM_001080485.1 | ZNF275 | ILMN_180340 | 2.25 |
| NM_138477.2 | CDAN1 | ILMN_168162 | 2.25 |
| NM_133487.1 | RAD51 | ILMN_4856 | 2.24 |
| NM_024570.1 | RNASEH2B | ILMN_20578 | 2.24 |
| NM_138687.1 | PIP5K2B | ILMN_12735 | 2.24 |
| NM_001030.3 | RPS27 | ILMN_5932 | 2.24 |
| NM_015446.3 | AHCTF1 | ILMN_164192 | 2.23 |
| NM_025084.1 | FLJ22795 | ILMN_1721 | 2.22 |
| NM_032918.1 | RERG | ILMN_12434 | 2.21 |
| NM_004064.2 | CDKN1B | ILMN_175665 | 2.21 |
| NM_182661.1 | CERK | ILMN_2275 | 2.21 |
| NM_022483.3 | C5orf28 | ILMN_5037 | 2.21 |
| NM_018097.1 | CEP27 | ILMN_15131 | 2.2 |
| NM_001008219.1 | AMY1C | ILMN_28222 | 2.2 |
| NM_018948.2 | ERRFI1 | ILMN_4328 | 2.19 |
| NM_005433.3 | YES1 | ILMN_183786 | 2.19 |
| NM_020808.3 | SIPA1L2 | ILMN_167573 | 2.19 |
| NM_004071.2 | CLK1 | ILMN_162592 | 2.19 |
| NM_017644.3 | KLHL24 | ILMN_26914 | 2.19 |
| NM_005189.1 | CBX2 | ILMN_28525 | 2.19 |
| NM_001031.4 | RPS28 | ILMN_992 | 2.19 |
| NM_024546.2 | C13orf7 | ILMN_137373 | 2.18 |
| NM_182789.2 | PAIP1 | ILMN_3393 | 2.18 |
| NM_005010.3 | NRCAM | ILMN_8955 | 2.18 |
| NM_006974.2 | ZNF33A | ILMN_4519 | 2.17 |
| NM_004730.1 | ETF1 | ILMN_9222 | 2.17 |
| NM_017827.2 | SARS2 | ILMN_20962 | 2.17 |
| NM_181836.3 | TMED7 | ILMN_168156 | 2.17 |
| NM_014839.3 | LPPR4 | ILMN_15660 | 2.16 |
| NM_006157.2 | NELL1 | ILMN_2560 | 2.16 |
| NM_177965.2 | C8orf37 | ILMN_11118 | 2.16 |
| NM_206907.3 | PRKAA1 | ILMN_180991 | 2.15 |
| NM_014366.4 | GNL3 | ILMN_18645 | 2.15 |
| NM_033412.1 | MCART1 | ILMN_22327 | 2.14 |
| NM_053053.2 | TADA1L | ILMN_25791 | 2.13 |
| NM_001008408.3 | RBM33 | ILMN_165407 | 2.13 |
| NM_001031712.2 | TRMT11 | ILMN_8801 | 2.12 |
| NM_001077442.1 | HNRNPC | ILMN_165238 | 2.12 |
| NM_014396.3 | VPS41 | ILMN_2386 | 2.12 |
| NM_016424.3 | CROP | ILMN_11116 | 2.12 |
| NM_002734.3 | PRKAR1A | ILMN_18925 | 2.12 |
| NM_016472.3 | C14orf129 | ILMN_7725 | 2.11 |
| NM_015906.3 | TRIM33 | ILMN_4131 | 2.11 |
| NM_001033505.1 | CSTF3 | ILMN_26942 | 2.11 |
| NM_182679.1 | GPATCH4 | ILMN_839 | 2.1 |
| NM_006527.2 | SLBP | ILMN_3687 | 2.1 |
| NM_002938.2 | RNF4 | ILMN_176496 | 2.09 |
| NM_014924.3 | KIAA0831 | ILMN_23945 | 2.09 |
| NM_021218.1 | C9orf80 | ILMN_27473 | 2.09 |
| NM_014033.3 | METTL7A | ILMN_40171 | 2.08 |
| NM_001987.4 | ETV6 | ILMN_175744 | 2.08 |
| XM_928075.2 | LOC643287 | ILMN_37869 | 2.08 |
| NM_003110.4 | SP2 | ILMN_7882 | 2.07 |
| NM_032199.1 | ARID5B | ILMN_165822 | 2.07 |
| NM_001024948.1 | FNBP1L | ILMN_162145 | 2.07 |
| NM_198097.1 | C7orf28B | ILMN_8655 | 2.07 |
| NM_002915.3 | RFC3 | ILMN_11616 | 2.06 |
| NM_002482.2 | NASP | ILMN_21654 | 2.06 |
| NM_024612.3 | DHX40 | ILMN_1864 | 2.06 |
| NM_001017421.1 | FKSG30 | ILMN_2393 | 2.06 |
| NM_001013258.1 | ZNF789 | ILMN_11535 | 2.05 |
| NM_013450.2 | BAZ2B | ILMN_20026 | 2.05 |
| NM_003045.3 | SLC7A1 | ILMN_162673 | 2.05 |
| NM_014747.2 | RIMS3 | ILMN_21581 | 2.05 |
| NM_000232.3 | SGCB | ILMN_162593 | 2.04 |
| NM_020474.2 | GALNT1 | ILMN_164550 | 2.04 |
| NM_199053.1 | C4orf41 | ILMN_8562 | 2.04 |
| XM_944915.1 | PTP4A2 | ILMN_137656 | 2.04 |
| NM_001621.2 | AHR | ILMN_138365 | 2.04 |
| XM_931434.2 | LOC400027 | ILMN_35789 | 2.03 |
| NM_152280.2 | SYT11 | ILMN_23967 | 2.03 |
| XM_939687.2 | LOC653658 | ILMN_33948 | 2.03 |
| NM_198267.1 | ING3 | ILMN_23155 | 2.02 |
| NM_024909.1 | C6orf134 | ILMN_21139 | 2.02 |
| NM_199043.1 | C14orf102 | ILMN_22442 | 2.01 |
| XM_001126418.1 | LOC727935 | ILMN_181411 | 2.01 |
| XM_937107.1 | LOC648057 | ILMN_32589 | 2.01 |
| NM_000786.2 | CYP51A1 | ILMN_161878 | 2.01 |
| XM_931911.2 | LOC643911 | ILMN_39566 | 2.01 |
| NM_198310.2 | TTC8 | ILMN_20549 | 2.01 |
| XM_001132771.1 | FAM116A | ILMN_173112 | 2.01 |
| XR_016986.1 | LOC643668 | ILMN_172192 | 2.01 |
| NM_004779.4 | CNOT8 | ILMN_10063 | 2 |
| NM_001077188.1 | HS6ST2 | ILMN_182242 | 2 |
| NM_032026.1 | TATDN1 | ILMN_17501 | 2 |
| NM_001008735.1 | HMG1L1 | ILMN_22757 | 2 |
| NM_000383.1 | AIRE | ILMN_179368 | 2 |
| NM_024735.2 | FBXO31 | ILMN_17806 | 1.99 |
| NM_015684.2 | ATP5S | ILMN_26667 | 1.98 |
| NM_021130.3 | PPIA | ILMN_25214 | 1.98 |
| NM_015560.1 | OPA1 | ILMN_10977 | 1.98 |
| NM_001677.3 | ATP1B1 | ILMN_25542 | 1.98 |
| NM_173515.2 | CNKSR3 | ILMN_25628 | 1.97 |
| NM_018247.2 | TMEM30A | ILMN_28236 | 1.97 |
| NM_003069.2 | SMARCA1 | ILMN_181613 | 1.96 |
| NM_016481.3 | C9orf156 | ILMN_12842 | 1.96 |
| NM_201557.2 | FHL2 | ILMN_42988 | 1.96 |
| NM_152834.2 | TMEM18 | ILMN_8053 | 1.96 |
| NM_005238.2 | ETS1 | ILMN_173009 | 1.96 |
| NM_016097.3 | IER3IP1 | ILMN_21844 | 1.96 |
| NM_032036.2 | FAM14A | ILMN_19102 | 1.96 |
| NM_001034194.1 | EXOSC9 | ILMN_26957 | 1.95 |
| NM_004786.1 | TXNL1 | ILMN_7321 | 1.95 |
| NM_020796.3 | SEMA6A | ILMN_11282 | 1.95 |
| NM_005334.2 | HCFC1 | ILMN_24237 | 1.95 |
| NM_021807.3 | EXOC4 | ILMN_28890 | 1.94 |
| NM_003342.4 | UBE2G1 | ILMN_179729 | 1.94 |
| NM_001319.5 | CSNK1G2 | ILMN_17274 | 1.94 |
| NM_000969.3 | RPL5 | ILMN_4986 | 1.94 |
| NM_022748.10 | TNS3 | ILMN_17676 | 1.93 |
| XM_926036.1 | LOC653103 | ILMN_32029 | 1.93 |
| NM_014380.1 | NGFRAP1 | ILMN_7162 | 1.93 |
| NM_012302.2 | LPHN2 | ILMN_11901 | 1.93 |
| NM_001006.3 | RPS3A | ILMN_28872 | 1.93 |
| NM_006731.2 | FKTN | ILMN_6512 | 1.93 |
| XM_377933.3 | LOC402251 | ILMN_45342 | 1.93 |
| NM_001013845.1 | CXorf40B | ILMN_170421 | 1.92 |
| NM_020368.1 | UTP3 | ILMN_22785 | 1.92 |
| NM_020040.3 | TUBB4Q | ILMN_177504 | 1.92 |
| NM_152411.2 | ZNF786 | ILMN_6379 | 1.92 |
| NM_144684.1 | ZNF480 | ILMN_10153 | 1.91 |
| NM_006054.2 | RTN3 | ILMN_20331 | 1.91 |
| NM_016626.3 | MEX3C | ILMN_172651 | 1.91 |
| NM_001029862.1 | ANKRD30B | ILMN_7263 | 1.91 |
| NM_004671.2 | PIAS2 | ILMN_11308 | 1.9 |
| NM_004330.1 | BNIP2 | ILMN_9985 | 1.9 |
| NM_003104.3 | SORD | ILMN_162054 | 1.89 |
| NM_006988.3 | ADAMTS1 | ILMN_11081 | 1.89 |
| NM_032810.2 | ATAD1 | ILMN_175726 | 1.89 |
| NM_031469.2 | SH3BGRL2 | ILMN_9801 | 1.89 |
| NM_002473.3 | MYH9 | ILMN_183555 | 1.89 |
| XM_497029.2 | LOC441408 | ILMN_31941 | 1.88 |
| NM_032776.1 | JMJD1C | ILMN_164120 | 1.88 |
| NM_018622.5 | PARL | ILMN_163763 | 1.88 |
| XM_371741.5 | LOC389293 | ILMN_168546 | 1.87 |
| NM_006997.2 | TACC2 | ILMN_16130 | 1.87 |
| NM_007241.2 | SNF8 | ILMN_8401 | 1.87 |
| NM_002951.2 | RPN2 | ILMN_30123 | 1.87 |
| NM_018325.1 | C9orf72 | ILMN_7216 | 1.86 |
| NM_181784.1 | SPRED2 | ILMN_12131 | 1.86 |
| XM_928934.1 | LOC645968 | ILMN_45269 | 1.86 |
| NM_173042.2 | IL18BP | ILMN_30884 | 1.85 |
| NM_152641.2 | ARID2 | ILMN_163259 | 1.85 |
| NM_016265.3 | ZNF12 | ILMN_27628 | 1.85 |
| NM_030881.2 | DDX17 | ILMN_28024 | 1.85 |
| NM_005921.1 | MAP3K1 | ILMN_309540 | 1.85 |
| XM_931359.2 | LOC338758 | ILMN_37634 | 1.84 |
| NM_020310.2 | MNT | ILMN_21283 | 1.84 |
| NM_022457.5 | RFWD2 | ILMN_1221 | 1.84 |
| NM_012459.1 | TIMM8B | ILMN_20372 | 1.83 |
| NM_014498.2 | GOLPH4 | ILMN_179486 | 1.83 |
| NM_005157.3 | ABL1 | ILMN_4033 | 1.83 |
| NM_001412.3 | EIF1AX | ILMN_22164 | 1.83 |
| NM_172097.1 | CATSPER2 | ILMN_23478 | 1.83 |
| NM_001019.4 | RPS15A | ILMN_27642 | 1.83 |
| NM_001995.2 | ACSL1 | ILMN_12367 | 1.82 |
| XM_926594.2 | LOC642502 | ILMN_31759 | 1.82 |
| NM_001024071.1 | GCH1 | ILMN_14690 | 1.82 |
| XM_929862.1 | LOC646900 | ILMN_44661 | 1.82 |
| NM_014614.1 | PSME4 | ILMN_164803 | 1.82 |
| NM_030752.2 | TCP1 | ILMN_418 | 1.82 |
| NR_002190.1 | SUMO1P3 | ILMN_16906 | 1.82 |
| NM_001752.2 | CAT | ILMN_170506 | 1.81 |
| NM_023012.4 | RSRC2 | ILMN_17793 | 1.81 |
| XM_931217.1 | LOC283683 | ILMN_45886 | 1.81 |
| NM_014060.1 | MCTS1 | ILMN_13725 | 1.81 |
| NM_024077.3 | SECISBP2 | ILMN_19156 | 1.8 |
| NM_019007.3 | ARMCX6 | ILMN_6931 | 1.8 |
| NM_001006.3 | RPS3A | ILMN_28872 | 1.8 |
| NM_005497.3 | GJC1 | ILMN_3556 | 1.8 |
| NM_033020.2 | TRIM33 | ILMN_3792 | 1.79 |
| NM_005319.3 | HIST1H1C | ILMN_18282 | 1.79 |
| NM_005665.4 | EVI5 | ILMN_17996 | 1.79 |
| NM_001270.2 | CHD1 | ILMN_163604 | 1.79 |
| NM_001008661.1 | CCBL2 | ILMN_1120 | 1.79 |
| NM_001627.2 | ALCAM | ILMN_164638 | 1.79 |
| NM_032794.1 | SLC44A4 | ILMN_14709 | 1.79 |
| NM_002374.3 | MAP2 | ILMN_38764 | 1.78 |
| NM_004779.4 | CNOT8 | ILMN_172926 | 1.78 |
| NM_014947.3 | FOXJ3 | ILMN_26064 | 1.78 |
| NM_002650.1 | PIK4CA | ILMN_20581 | 1.78 |
| NM_003925.1 | MBD4 | ILMN_18891 | 1.78 |
| NM_001496.3 | GFRA3 | ILMN_8392 | 1.77 |
| NM_012130.2 | CLDN14 | ILMN_19370 | 1.77 |
| NM_004354.1 | CCNG2 | ILMN_10201 | 1.77 |
| NM_173073.2 | SLC35C2 | ILMN_14167 | 1.77 |
| NM_012257.3 | HBP1 | ILMN_167468 | 1.77 |
| NM_012223.2 | MYO1B | ILMN_175452 | 1.77 |
| NM_199189.1 | MATR3 | ILMN_15287 | 1.77 |
| NM_139235.3 | NOL6 | ILMN_7349 | 1.76 |
| NM_014918.3 | CHSY1 | ILMN_17929 | 1.76 |
| NM_001006946.1 | SDC1 | ILMN_169032 | 1.76 |
| NM_006903.4 | PPA2 | ILMN_15173 | 1.76 |
| NM_018566.3 | YOD1 | ILMN_19081 | 1.75 |
| NM_170721.1 | MSI2 | ILMN_25750 | 1.75 |
| NM_019591.2 | ZNF26 | ILMN_3233 | 1.75 |
| NM_002160.2 | TNC | ILMN_14948 | 1.75 |
| NM_001038702.1 | CDC42SE2 | ILMN_28719 | 1.75 |
| NM_007005.3 | TLE4 | ILMN_14046 | 1.75 |
| XM_937691.1 | LOC648622 | ILMN_42936 | 1.75 |
| NM_178517.3 | PIGW | ILMN_162681 | 1.74 |
| NM_080760.3 | DACH1 | ILMN_20187 | 1.74 |
| NM_170695.2 | TGIF1 | ILMN_162784 | 1.73 |
| NM_014729.2 | TOX | ILMN_16587 | 1.73 |
| NM_001098495.1 | ZNF419 | ILMN_306789 | 1.73 |
| XM_927280.1 | LOC644033 | ILMN_39734 | 1.73 |
| NM_173694.3 | ATP11C | ILMN_169690 | 1.73 |
| NM_014897.1 | ZNF652 | ILMN_3215 | 1.73 |
| NM_001013703.2 | EIF2AK4 | ILMN_164547 | 1.72 |
| NM_020940.2 | KIAA1600 | ILMN_8082 | 1.72 |
| NM_201440.1 | PPHLN1 | ILMN_4445 | 1.72 |
| NM_006392.2 | NOL5A | ILMN_13841 | 1.72 |
| NM_015497.2 | TMEM87A | ILMN_181695 | 1.72 |
| NM_032389.3 | ARFGAP2 | ILMN_12944 | 1.71 |
| NM_014935.2 | PLEKHA6 | ILMN_163005 | 1.71 |
| NM_025065.6 | BXDC5 | ILMN_29951 | 1.71 |
| NM_031263.1 | HNRPK | ILMN_16515 | 1.71 |
| NM_033063.1 | MAP6 | ILMN_6882 | 1.7 |
| NM_004560.2 | ROR2 | ILMN_22834 | 1.7 |
| NM_031314.1 | HNRPC | ILMN_24356 | 1.7 |
| NM_000787.3 | DBH | ILMN_25962 | 1.7 |
| NM_004566.2 | PFKFB3 | ILMN_163833 | 1.69 |
| NM_016021.2 | UBE2J1 | ILMN_164177 | 1.69 |
| NM_014319.3 | LEMD3 | ILMN_178185 | 1.69 |
| NM_017719.3 | SNRK | ILMN_5234 | 1.69 |
| NM_001033505.1 | CSTF3 | ILMN_26942 | 1.69 |
| NM_018976.3 | SLC38A2 | ILMN_10001 | 1.69 |
| NM_015570.1 | AUTS2 | ILMN_4348 | 1.69 |
| NM_014962.2 | BTBD3 | ILMN_180757 | 1.68 |
| NM_017819.2 | RG9MTD1 | ILMN_26970 | 1.68 |
| NM_177972.1 | TUB | ILMN_11520 | 1.68 |
| NM_004725.2 | BUB3 | ILMN_5562 | 1.68 |
| NM_020783.2 | SYT4 | ILMN_21875 | 1.68 |
| XR_018848.1 | LOC650369 | ILMN_169499 | 1.67 |
| NR_002308.1 | LOC442454 | ILMN_309609 | 1.67 |
| NM_001275.3 | CHGA | ILMN_23390 | 1.67 |
| XM_942501.1 | CSF2RA | ILMN_137685 | 1.66 |
| XM_940278.1 | LOC651149 | ILMN_44210 | 1.66 |
| NM_001039755.1 | FLJ44124 | ILMN_44450 | 1.66 |
| NM_001029950.1 | DKFZp434K191 | ILMN_28495 | 1.65 |
| NM_023080.1 | C8orf33 | ILMN_15901 | 1.65 |
| NM_005385.3 | NKTR | ILMN_23378 | 1.65 |
| NR_001434.1 | HLA-H | ILMN_5683 | 1.64 |
| XM_001127981.1 | LOC728014 | ILMN_169164 | 1.64 |
| NM_020925.2 | CACHD1 | ILMN_14104 | 1.64 |
| NM_130809.2 | PRRC1 | ILMN_24905 | 1.64 |
| NM_001418.3 | EIF4G2 | ILMN_19314 | 1.64 |
| NM_020933.2 | ZNF317 | ILMN_22884 | 1.63 |
| NM_001044387.1 | ZNF557 | ILMN_180266 | 1.63 |
| NM_173797.2 | PAPD4 | ILMN_2190 | 1.63 |
| NM_005637.2 | SS18 | ILMN_6637 | 1.63 |
| NM_201552.1 | FGL1 | ILMN_30345 | 1.62 |
| NM_014694.2 | ADAMTSL2 | ILMN_697 | 1.62 |
| NM_024653.3 | PRKRIP1 | ILMN_13077 | 1.62 |
| NM_152493.2 | ZNF362 | ILMN_7745 | 1.62 |
| NM_001023587.1 | ABCC5 | ILMN_438 | 1.62 |
| NM_002835.2 | PTPN12 | ILMN_26144 | 1.62 |
| NM_017833.2 | C21orf55 | ILMN_6782 | 1.62 |
| NM_013276.2 | SHPK | ILMN_22706 | 1.61 |
| NM_015678.3 | NBEA | ILMN_171545 | 1.61 |
| NM_004634.2 | BRPF1 | ILMN_17537 | 1.61 |
| NM_001356.3 | DDX3X | ILMN_183040 | 1.61 |
| NM_175066.2 | DDX51 | ILMN_165366 | 1.61 |
| NM_001300.4 | KLF6 | ILMN_17961 | 1.6 |
| NM_005342.2 | HMGB3 | ILMN_8326 | 1.6 |
| NM_001253.2 | CDC5L | ILMN_15507 | 1.6 |
| NM_080927.3 | DCBLD2 | ILMN_175741 | 1.6 |
| NM_001002878.1 | THOC5 | ILMN_13820 | 1.6 |
| XM_928464.1 | LOC146517 | ILMN_32888 | 1.59 |
| NM_014367.3 | C3orf28 | ILMN_24382 | 1.59 |
| NM_024804.1 | ZNF669 | ILMN_26142 | 1.59 |
| NM_003420.3 | ZNF35 | ILMN_180943 | 1.59 |
| NM_017426.2 | NUP54 | ILMN_170157 | 1.59 |
| NM_145255.2 | MRPL10 | ILMN_19178 | 1.59 |
| NM_014330.2 | PPP1R15A | ILMN_1024 | 1.59 |
| NM_133496.3 | SLC30A7 | ILMN_20389 | 1.59 |
| NM_006045.1 | ATP9A | ILMN_176431 | 1.59 |
| NM_001111.3 | ADAR | ILMN_20593 | 1.59 |
| NM_004508.2 | IDI1 | ILMN_20349 | 1.58 |
| NM_004477.2 | FRG1 | ILMN_11683 | 1.58 |
| NM_016026.2 | RDH11 | ILMN_26810 | 1.58 |
| NM_015534.3 | ZZZ3 | ILMN_14976 | 1.58 |
| NM_003403.3 | YY1 | ILMN_4019 | 1.58 |
| NM_006947.3 | SRP72 | ILMN_182551 | 1.58 |
| NM_003750.2 | EIF3A | ILMN_25761 | 1.58 |
| NM_153451.2 | ORAOV1 | ILMN_5733 | 1.57 |
| NM_024900.3 | PHF17 | ILMN_1535 | 1.57 |
| NM_002996.3 | CX3CL1 | ILMN_9636 | 1.57 |
| NM_020824.2 | ARHGAP21 | ILMN_10414 | 1.57 |
| NM_014717.1 | ZNF536 | ILMN_179125 | 1.56 |
| NM_022173.1 | TIA1 | ILMN_29910 | 1.56 |
| NM_001034194.1 | EXOSC9 | ILMN_26957 | 1.56 |
| NM_182491.1 | ZFAND2A | ILMN_21900 | 1.56 |
| NM_080702.2 | BAT3 | ILMN_4429 | 1.56 |
| NM_004523.2 | KIF11 | ILMN_182103 | 1.56 |
| XM_942991.2 | LOC642934 | ILMN_39429 | 1.56 |
| NM_213606.1 | SLC16A12 | ILMN_28607 | 1.56 |
| NM_005093.3 | CBFA2T2 | ILMN_42009 | 1.55 |
| NM_031954.3 | KCTD10 | ILMN_30217 | 1.55 |
| NM_022151.4 | MOAP1 | ILMN_165500 | 1.55 |
| NM_001012507.1 | C6orf173 | ILMN_25405 | 1.55 |
| NR_003659.1 | FAM39DP | ILMN_307683 | 1.55 |
| NM_016047.3 | SF3B14 | ILMN_12279 | 1.55 |
| NM_001023567.2 | GOLGA8B | ILMN_14405 | 1.55 |
| NM_001562.2 | IL18 | ILMN_167736 | 1.55 |
| NM_033109.2 | PNPT1 | ILMN_22316 | 1.55 |
| NM_012343.3 | NNT | ILMN_183201 | 1.54 |
| NM_020532.4 | RTN4 | ILMN_164893 | 1.54 |
| NM_005920.2 | MEF2D | ILMN_3465 | 1.53 |
| NM_018981.1 | DNAJC10 | ILMN_19735 | 1.53 |
| NM_024311.2 | MFSD11 | ILMN_7695 | 1.53 |
| NM_001013699.1 | LOC440093 | ILMN_19743 | 1.53 |
| NM_000344.2 | SMN1 | ILMN_18160 | 1.52 |
| NM_152424.1 | FLJ39827 | ILMN_19358 | 1.52 |
| XM_937154.1 | LOC648099 | ILMN_35361 | 1.52 |
| NM_197958.1 | LARP6 | ILMN_25698 | 1.52 |
| NM_014779.2 | TSC22D2 | ILMN_5940 | 1.52 |
| NM_007115.2 | TNFAIP6 | ILMN_11686 | 1.52 |
| NM_016185.2 | HN1 | ILMN_3023 | 1.52 |
| NM_207306.1 | KIAA0495 | ILMN_27282 | 1.51 |
| NM_178496.2 | C3orf59 | ILMN_14619 | 1.51 |
| NM_133638.2 | ADAMTS19 | ILMN_8089 | 1.51 |
| NM_006064.3 | RRAGB | ILMN_28228 | 1.51 |
| NM_001037533.1 | GON4L | ILMN_14180 | 1.51 |
| NM_020664.3 | DECR2 | ILMN_7935 | 1.51 |
| NM_004424.3 | E4F1 | ILMN_23848 | 1.51 |
| NM_003108.3 | SOX11 | ILMN_28038 | 1.51 |
| NM_005667.2 | RNF103 | ILMN_17861 | 1.5 |
| NM_001873.1 | CPE | ILMN_12337 | 1.5 |
| NM_025189.2 | ZNF430 | ILMN_24543 | 1.5 |
| NM_021732.1 | AVPI1 | ILMN_9920 | -1.5 |
| NM_153018.2 | ZFP3 | ILMN_42182 | -1.5 |
| NM_031492.2 | RBM4B | ILMN_29996 | -1.5 |
| NM_001967.3 | EIF4A2 | ILMN_5908 | -1.5 |
| XR_001271.1 | LOC441191 | ILMN_39396 | -1.51 |
| NM_013352.2 | DSE | ILMN_14589 | -1.51 |
| NM_000309.2 | PPOX | ILMN_9419 | -1.51 |
| NM_016297.2 | PCYOX1 | ILMN_15130 | -1.51 |
| NM_001918.2 | DBT | ILMN_169961 | -1.51 |
| NM_001151.2 | SLC25A4 | ILMN_2485 | -1.51 |
| NM_001008709.1 | PPP1CA | ILMN_29100 | -1.51 |
| NM_001398.2 | ECH1 | ILMN_28288 | -1.51 |
| NM_005896.2 | IDH1 | ILMN_14217 | -1.52 |
| NM_181306.1 | MRPL52 | ILMN_16276 | -1.52 |
| NM_015984.2 | UCHL5 | ILMN_3370 | -1.52 |
| NM_024099.3 | C11orf48 | ILMN_24145 | -1.52 |
| NM_014800.9 | ELMO1 | ILMN_33356 | -1.52 |
| NM_032574.2 | DPY30 | ILMN_18534 | -1.52 |
| NM_002338.2 | LSAMP | ILMN_861 | -1.53 |
| NM_022048.3 | CSNK1G1 | ILMN_19512 | -1.53 |
| NM_001042426.1 | CENPA | ILMN_180589 | -1.53 |
| NM_016499.3 | MGC13379 | ILMN_180361 | -1.53 |
| NM_012474.3 | UCK2 | ILMN_23283 | -1.53 |
| NM_016647.2 | C8orf55 | ILMN_25304 | -1.53 |
| NM_138787.2 | C11orf74 | ILMN_16125 | -1.53 |
| NM_021168.2 | RAB40C | ILMN_22367 | -1.53 |
| NM_015636.3 | EIF2B4 | ILMN_18552 | -1.53 |
| NM_001605.2 | AARS | ILMN_29917 | -1.53 |
| NM_001007256.1 | KLHDC9 | ILMN_13723 | -1.54 |
| NM_133375.2 | DIS3L | ILMN_29373 | -1.54 |
| NM_004435.2 | ENDOG | ILMN_26482 | -1.54 |
| XM_926382.2 | LOC642755 | ILMN_32084 | -1.54 |
| NM_198486.2 | RPL7L1 | ILMN_9155 | -1.54 |
| NM_198045.1 | ZDHHC16 | ILMN_948 | -1.54 |
| NM_005828.3 | WDR68 | ILMN_171190 | -1.54 |
| NM_018648.3 | NOLA3 | ILMN_14928 | -1.54 |
| NM_181876.2 | PPP2R2C | ILMN_15268 | -1.55 |
| NM_021242.4 | MID1IP1 | ILMN_161908 | -1.55 |
| NM_005057.2 | RBBP5 | ILMN_17778 | -1.55 |
| NM_016625.2 | RSRC1 | ILMN_14978 | -1.55 |
| NM_001080415.1 | SR140 | ILMN_169874 | -1.55 |
| NM_012289.3 | KEAP1 | ILMN_18799 | -1.55 |
| NM_003707.1 | RUVBL1 | ILMN_16596 | -1.55 |
| NM_138797.1 | ANKRD54 | ILMN_21813 | -1.56 |
| NM_003314.1 | TTC1 | ILMN_11292 | -1.56 |
| NM_003083.2 | SNAPC2 | ILMN_14587 | -1.56 |
| NM_002973.2 | ATXN2 | ILMN_164628 | -1.56 |
| NM_018044.2 | NSUN5 | ILMN_895 | -1.57 |
| NM_018199.2 | EXDL2 | ILMN_4351 | -1.57 |
| NM_024339.2 | THOC6 | ILMN_25260 | -1.57 |
| NM_030980.1 | ISG20L2 | ILMN_7800 | -1.57 |
| NM_000282.2 | PCCA | ILMN_6045 | -1.58 |
| NM_201280.1 | MUTED | ILMN_21576 | -1.58 |
| NM_005926.2 | MFAP1 | ILMN_20656 | -1.58 |
| NM_021925.2 | C2orf43 | ILMN_2189 | -1.58 |
| NM_005740.2 | DNAL4 | ILMN_22246 | -1.58 |
| NM_016561.1 | BFAR | ILMN_23440 | -1.58 |
| NM_018718.1 | TSGA14 | ILMN_11000 | -1.58 |
| XM_931224.1 | LOC283683 | ILMN_45961 | -1.59 |
| NM_001018020.1 | TPM1 | ILMN_14091 | -1.59 |
| XM_942540.1 | SAPS2 | ILMN_138398 | -1.59 |
| NM_016030.5 | TTC15 | ILMN_21565 | -1.59 |
| NM_016053.2 | CCDC53 | ILMN_25394 | -1.59 |
| NM_007111.3 | TFDP1 | ILMN_18662 | -1.59 |
| NM_005534.2 | IFNGR2 | ILMN_174164 | -1.59 |
| NM_014612.3 | FAM120A | ILMN_14224 | -1.59 |
| NM_152318.2 | C12orf45 | ILMN_25959 | -1.6 |
| NM_032848.1 | C12orf52 | ILMN_22595 | -1.6 |
| NM_213650.1 | SFXN4 | ILMN_10964 | -1.6 |
| NM_000100.2 | CSTB | ILMN_26819 | -1.6 |
| NM_016447.2 | MPP6 | ILMN_163134 | -1.61 |
| NM_003729.2 | RTCD1 | ILMN_11697 | -1.61 |
| NM_024766.2 | C2orf34 | ILMN_14025 | -1.61 |
| NM_002601.2 | PDE6D | ILMN_2430 | -1.61 |
| NM_198047.1 | HIBCH | ILMN_24888 | -1.61 |
| NM_181454.1 | MRPL55 | ILMN_26404 | -1.61 |
| NM_000701.6 | ATP1A1 | ILMN_677 | -1.61 |
| NM_052873.1 | C14orf179 | ILMN_22122 | -1.61 |
| NM_006178.1 | NSF | ILMN_23282 | -1.61 |
| XM_928247.1 | LOC441528 | ILMN_30629 | -1.62 |
| NM_021244.3 | RRAGD | ILMN_5663 | -1.62 |
| NM_032166.2 | ATRIP | ILMN_3211 | -1.62 |
| NM_017865.2 | ZNF692 | ILMN_22649 | -1.62 |
| NM_003946.3 | NOL3 | ILMN_10618 | -1.62 |
| NM_004130.2 | GYG1 | ILMN_22958 | -1.62 |
| NM_001033503.1 | SAR1B | ILMN_16595 | -1.62 |
| NM_003916.3 | AP1S2 | ILMN_3812 | -1.62 |
| NM_005998.3 | CCT3 | ILMN_173257 | -1.62 |
| NM_025049.2 | PIF1 | ILMN_7325 | -1.63 |
| NM_033402.3 | LRRCC1 | ILMN_15234 | -1.63 |
| NM_020385.2 | REXO4 | ILMN_29774 | -1.63 |
| NM_001031717.2 | CRELD1 | ILMN_14216 | -1.63 |
| NM_025205.3 | MED28 | ILMN_14574 | -1.63 |
| NM_001035505.1 | BOLA3 | ILMN_29223 | -1.63 |
| NM_003656.3 | CAMK1 | ILMN_21373 | -1.64 |
| XM_377476.4 | MGC57346 | ILMN_165970 | -1.64 |
| NM_181042.2 | PBRM1 | ILMN_16253 | -1.65 |
| NM_033492.1 | CDC2L1 | ILMN_20802 | -1.65 |
| NM_013247.4 | HTRA2 | ILMN_22612 | -1.65 |
| NM_203385.1 | RNH1 | ILMN_28630 | -1.65 |
| NM_020892.1 | DTX2 | ILMN_21612 | -1.65 |
| NM_005749.2 | TOB1 | ILMN_13735 | -1.65 |
| NM_015463.1 | C2orf32 | ILMN_1437 | -1.65 |
| NM_024516.2 | C16orf53 | ILMN_20272 | -1.65 |
| NM_005830.2 | MRPS31 | ILMN_6293 | -1.65 |
| NM_018170.3 | P15RS | ILMN_174036 | -1.65 |
| NM_014188.2 | SSU72 | ILMN_29116 | -1.65 |
| NM_001679.2 | ATP1B3 | ILMN_3785 | -1.66 |
| NR_001588.1 | SBDSP | ILMN_12233 | -1.66 |
| NM_001031812.2 | CSNK1G3 | ILMN_17895 | -1.66 |
| NM_182523.1 | C3orf68 | ILMN_4406 | -1.66 |
| NM_001040011.1 | C9orf119 | ILMN_306686 | -1.66 |
| NM_145080.3 | NSMCE1 | ILMN_27090 | -1.66 |
| NM_133172.2 | APBB3 | ILMN_14368 | -1.66 |
| NM_000076.1 | CDKN1C | ILMN_20689 | -1.67 |
| NM_003342.4 | UBE2G1 | ILMN_179729 | -1.67 |
| NM_007369.2 | GPR161 | ILMN_22837 | -1.67 |
| NM_005953.2 | MT2A | ILMN_11198 | -1.67 |
| NM_014412.2 | CACYBP | ILMN_27301 | -1.67 |
| NM_181528.2 | NAT5 | ILMN_43222 | -1.67 |
| NM_006409.2 | ARPC1A | ILMN_14839 | -1.67 |
| NM_015442.1 | CNOT10 | ILMN_698 | -1.68 |
| NM_015609.2 | C1orf144 | ILMN_5836 | -1.68 |
| NM_005723.2 | TSPAN5 | ILMN_8032 | -1.68 |
| NM_016390.2 | C9orf114 | ILMN_20184 | -1.68 |
| NM_015049.1 | TRAK2 | ILMN_21571 | -1.68 |
| NM_018244.3 | UQCC | ILMN_26543 | -1.68 |
| NM_016059.3 | PPIL1 | ILMN_30246 | -1.68 |
| NM_016319.1 | COPS7A | ILMN_13902 | -1.68 |
| NM_006014.3 | LAGE3 | ILMN_1071 | -1.68 |
| NM_004821.1 | HAND1 | ILMN_29799 | -1.68 |
| NM_018130.2 | SHQ1 | ILMN_29855 | -1.69 |
| NM_002755.2 | MAP2K1 | ILMN_164648 | -1.69 |
| NM_001025248.1 | DUT | ILMN_163345 | -1.69 |
| NM_005192.2 | CDKN3 | ILMN_4098 | -1.69 |
| NM_001002246.1 | ANAPC11 | ILMN_5565 | -1.69 |
| NM_153682.2 | PIGP | ILMN_18625 | -1.69 |
| NM_005082.4 | TRIM25 | ILMN_21751 | -1.7 |
| NM_145697.1 | CDCA1 | ILMN_17725 | -1.7 |
| XM_936354.2 | LOC642197 | ILMN_44406 | -1.7 |
| NM_006602.2 | TCFL5 | ILMN_12278 | -1.7 |
| NM_032376.2 | TMEM101 | ILMN_24128 | -1.7 |
| NM_015252.2 | EHBP1 | ILMN_8575 | -1.7 |
| NM_007342.1 | NUPL2 | ILMN_2154 | -1.7 |
| NM_001025238.1 | TSPAN4 | ILMN_26489 | -1.71 |
| NM_001005368.1 | ZNF32 | ILMN_181781 | -1.71 |
| NM_001017392.2 | SFRS14 | ILMN_17110 | -1.71 |
| NM_032361.1 | THOC3 | ILMN_17969 | -1.71 |
| NM_053056.2 | CCND1 | ILMN_166524 | -1.71 |
| NM_014964.3 | EPN2 | ILMN_21232 | -1.72 |
| NM_207291.1 | USF2 | ILMN_6055 | -1.72 |
| NM_018718.1 | TSGA14 | ILMN_11000 | -1.72 |
| NM_153824.1 | PYCR1 | ILMN_8761 | -1.72 |
| NM_001031726.2 | C19orf12 | ILMN_10211 | -1.72 |
| NM_198336.1 | INSIG1 | ILMN_12839 | -1.72 |
| NM_005694.1 | COX17 | ILMN_19252 | -1.72 |
| NM_021219.2 | JAM2 | ILMN_12795 | -1.73 |
| NM_004885.1 | NPFFR2 | ILMN_20676 | -1.73 |
| NM_004901.2 | ENTPD4 | ILMN_19012 | -1.73 |
| NM_003186.3 | TAGLN | ILMN_2335 | -1.73 |
| NM_012289.3 | KEAP1 | ILMN_18799 | -1.73 |
| NM_021076.2 | NEFH | ILMN_9306 | -1.73 |
| NM_001001998.1 | EXOSC10 | ILMN_26222 | -1.74 |
| NM_006036.3 | PREPL | ILMN_177009 | -1.74 |
| NM_006362.4 | NXF1 | ILMN_11773 | -1.74 |
| NM_018062.2 | FANCL | ILMN_24728 | -1.74 |
| NM_018845.2 | RAG1AP1 | ILMN_19038 | -1.74 |
| NM_001012413.1 | SGOL1 | ILMN_14464 | -1.75 |
| XM_001131304.1 | LOC728635 | ILMN_168315 | -1.75 |
| NM_006828.2 | ASCC3 | ILMN_174599 | -1.75 |
| NM_032041.1 | NCALD | ILMN_13197 | -1.75 |
| NM_001018109.1 | PIR | ILMN_13999 | -1.75 |
| NM_013328.2 | PYCR2 | ILMN_18209 | -1.75 |
| NM_018465.2 | C9orf46 | ILMN_17839 | -1.75 |
| NM_133458.2 | ZFP90 | ILMN_174886 | -1.75 |
| NM_006837.2 | COPS5 | ILMN_480 | -1.75 |
| NM_025129.3 | FUZ | ILMN_24173 | -1.76 |
| NM_004398.2 | DDX10 | ILMN_20779 | -1.76 |
| NM_018259.4 | TTC17 | ILMN_22995 | -1.76 |
| NM_002086.3 | GRB2 | ILMN_173749 | -1.76 |
| NM_003916.3 | AP1S2 | ILMN_3812 | -1.76 |
| NM_003273.2 | TM7SF2 | ILMN_28596 | -1.76 |
| NM_001031738.1 | TMEM150 | ILMN_20451 | -1.77 |
| NM_014039.2 | C11orf54 | ILMN_4783 | -1.77 |
| NM_012138.3 | AATF | ILMN_29906 | -1.77 |
| NM_022914.2 | ACD | ILMN_5907 | -1.77 |
| NM_015999.2 | ADIPOR1 | ILMN_4949 | -1.77 |
| NM_007083.3 | NUDT6 | ILMN_903 | -1.78 |
| NM_178507.2 | OAF | ILMN_12751 | -1.78 |
| NM_180976.1 | PPP2R5D | ILMN_2366 | -1.78 |
| NM_022334.3 | ITGB1BP1 | ILMN_13169 | -1.78 |
| NM_152274.2 | FAM58A | ILMN_3352 | -1.78 |
| NM_000599.2 | IGFBP5 | ILMN_168089 | -1.78 |
| NM_133371.2 | MYOZ3 | ILMN_21305 | -1.79 |
| NM_001634.4 | AMD1 | ILMN_21529 | -1.79 |
| NM_138701.1 | C7orf11 | ILMN_20229 | -1.79 |
| NM_006963.3 | ZNF22 | ILMN_165495 | -1.79 |
| NM_181702.1 | GEM | ILMN_16170 | -1.8 |
| NM_014933.2 | SEC31A | ILMN_23819 | -1.8 |
| NM_001545.1 | ICT1 | ILMN_11458 | -1.8 |
| NM_021254.1 | C21orf59 | ILMN_28603 | -1.8 |
| NM_033362.2 | MRPS12 | ILMN_19234 | -1.81 |
| NM_001040668.1 | BCL2L12 | ILMN_177176 | -1.81 |
| NM_032907.3 | UBL7 | ILMN_17890 | -1.81 |
| NM_020909.2 | EPB41L5 | ILMN_17196 | -1.81 |
| NM_001078651.1 | TMEM134 | ILMN_176754 | -1.81 |
| NM_021177.3 | LSM2 | ILMN_22587 | -1.81 |
| NM_006876.1 | B3GNT6 | ILMN_16433 | -1.82 |
| NM_006366.2 | CAP2 | ILMN_27367 | -1.83 |
| NM_152362.1 | TNFAIP8L1 | ILMN_3344 | -1.83 |
| NM_002712.1 | PPP1R7 | ILMN_29559 | -1.83 |
| NM_018186.2 | C1orf112 | ILMN_5134 | -1.83 |
| NM_012342.2 | BAMBI | ILMN_8469 | -1.83 |
| NM_175069.1 | APTX | ILMN_7416 | -1.83 |
| NM_016401.2 | C11orf73 | ILMN_22672 | -1.83 |
| XM_374020.4 | LOC375295 | ILMN_45377 | -1.83 |
| NM_000527.2 | LDLR | ILMN_10126 | -1.83 |
| NM_019095.3 | CRLS1 | ILMN_14031 | -1.83 |
| NM_001002019.1 | PUS1 | ILMN_13055 | -1.84 |
| NM_001001520.1 | HDGF2 | ILMN_8472 | -1.84 |
| NM_001005742.1 | GBA | ILMN_28933 | -1.84 |
| NM_024678.3 | NARS2 | ILMN_13605 | -1.84 |
| NM_032775.2 | KLHL22 | ILMN_17770 | -1.84 |
| NM_170784.1 | MKKS | ILMN_17701 | -1.84 |
| NM_032166.2 | ATRIP | ILMN_3211 | -1.85 |
| NM_148178.1 | C9orf23 | ILMN_3926 | -1.85 |
| NM_001003793.1 | RBMS3 | ILMN_16411 | -1.85 |
| NM_004577.3 | PSPH | ILMN_14445 | -1.85 |
| NM_022473.1 | ZFP106 | ILMN_6305 | -1.85 |
| NM_006834.2 | RAB32 | ILMN_784 | -1.85 |
| NM_002957.3 | RXRA | ILMN_6758 | -1.86 |
| NM_020463.1 | SMEK2 | ILMN_21228 | -1.86 |
| NM_017489.1 | TERF1 | ILMN_164297 | -1.86 |
| NM_184234.1 | RBM39 | ILMN_20330 | -1.86 |
| NM_007277.4 | EXOC3 | ILMN_6110 | -1.87 |
| NM_007155.4 | ZP3 | ILMN_17555 | -1.87 |
| NM_017693.2 | BIVM | ILMN_181297 | -1.87 |
| NM_018079.3 | SRBD1 | ILMN_28720 | -1.87 |
| NM_015200.1 | PDS5A | ILMN_30113 | -1.88 |
| NM_053067.1 | UBQLN1 | ILMN_9768 | -1.88 |
| XM_926382.2 | LOC642755 | ILMN_32084 | -1.88 |
| NM_003715.2 | USO1 | ILMN_23419 | -1.88 |
| NM_006178.1 | NSF | ILMN_23282 | -1.89 |
| NM_020187.2 | C3orf37 | ILMN_1343 | -1.89 |
| NM_006282.2 | STK4 | ILMN_21491 | -1.89 |
| NM_003800.3 | RNGTT | ILMN_17056 | -1.89 |
| NM_012111.1 | AHSA1 | ILMN_11051 | -1.89 |
| NM_213720.1 | C22orf16 | ILMN_25503 | -1.89 |
| NM_000854.2 | GSTT2 | ILMN_4850 | -1.9 |
| NM_130799.1 | MEN1 | ILMN_5184 | -1.9 |
| NR_001449.1 | TRK1 | ILMN_6493 | -1.91 |
| NM_004864.1 | GDF15 | ILMN_2688 | -1.91 |
| NM_013293.3 | TRA2A | ILMN_8090 | -1.91 |
| NM_078629.1 | MSL3L1 | ILMN_29354 | -1.91 |
| NM_001360.1 | DHCR7 | ILMN_2138 | -1.92 |
| NM_022065.4 | THADA | ILMN_22705 | -1.92 |
| NM_032459.1 | EFS | ILMN_17620 | -1.92 |
| NM_001080546.1 | LOC219854 | ILMN_168339 | -1.92 |
| NM_002528.4 | NTHL1 | ILMN_15981 | -1.92 |
| NM_006182.2 | DDR2 | ILMN_20698 | -1.92 |
| NM_152740.2 | HIBADH | ILMN_28448 | -1.92 |
| NM_006963.3 | ZNF22 | ILMN_165495 | -1.92 |
| NM_025233.5 | COASY | ILMN_13627 | -1.93 |
| NM_017843.3 | BCAS4 | ILMN_21706 | -1.93 |
| NM_001040668.1 | BCL2L12 | ILMN_177176 | -1.93 |
| NM_001080422.1 | FAM108A3 | ILMN_174864 | -1.93 |
| NM_024098.1 | CCDC86 | ILMN_27103 | -1.93 |
| NM_020119.3 | ZC3HAV1 | ILMN_13243 | -1.93 |
| NM_001889.2 | CRYZ | ILMN_30248 | -1.93 |
| NM_001007230.1 | SPOP | ILMN_12838 | -1.93 |
| NM_005398.4 | PPP1R3C | ILMN_4487 | -1.94 |
| NM_020235.3 | BBX | ILMN_28437 | -1.94 |
| NM_005035.3 | POLRMT | ILMN_168627 | -1.94 |
| NM_016042.2 | EXOSC3 | ILMN_174330 | -1.94 |
| NM_015935.4 | KIAA0859 | ILMN_172647 | -1.94 |
| NM_024647.4 | NUP43 | ILMN_28463 | -1.95 |
| NM_020701.2 | ISY1 | ILMN_17522 | -1.96 |
| NM_024585.2 | ARMC7 | ILMN_163623 | -1.96 |
| NM_001040056.1 | MAPK3 | ILMN_177323 | -1.96 |
| NM_032226.2 | ZCCHC7 | ILMN_21489 | -1.96 |
| NM_017802.2 | HEATR2 | ILMN_1114 | -1.96 |
| NM_030771.1 | CCDC34 | ILMN_2645 | -1.96 |
| NM_053067.1 | UBQLN1 | ILMN_9768 | -1.97 |
| NM_016551.1 | TM7SF3 | ILMN_7797 | -1.97 |
| NM_014109.2 | ATAD2 | ILMN_172027 | -1.97 |
| NM_001001396.1 | ATP2B4 | ILMN_4324 | -1.97 |
| NM_017812.2 | CHCHD3 | ILMN_23539 | -1.97 |
| NM_001037494.1 | DYNLL1 | ILMN_14802 | -1.97 |
| NM_001031706.1 | PLEKHB2 | ILMN_179121 | -1.98 |
| NM_006819.1 | STIP1 | ILMN_28761 | -1.98 |
| NM_145247.4 | C10orf78 | ILMN_1251 | -1.99 |
| XM_001132711.1 | RFNG | ILMN_168322 | -1.99 |
| NM_014026.3 | DCPS | ILMN_24626 | -2 |
| NM_004886.3 | APBA3 | ILMN_4538 | -2 |
| NM_198527.2 | HDDC3 | ILMN_29602 | -2 |
| NM_173510.1 | CCDC117 | ILMN_21814 | -2 |
| NM_002896.1 | RBM4 | ILMN_11057 | -2 |
| NM_182919.1 | TICAM1 | ILMN_11434 | -2 |
| NM_001914.2 | CYB5A | ILMN_25182 | -2 |
| NM_002067.1 | GNA11 | ILMN_25749 | -2 |
| NM_022473.1 | ZFP106 | ILMN_6305 | -2 |
| NM_198391.1 | FLRT3 | ILMN_23273 | -2 |
| NM_199235.1 | COLEC11 | ILMN_6793 | -2 |
| NM_001033566.1 | RHOT1 | ILMN_6821 | -2 |
| NM_004982.2 | KCNJ8 | ILMN_29993 | -2.01 |
| NM_007280.1 | OIP5 | ILMN_18200 | -2.01 |
| NM_033064.3 | ATCAY | ILMN_27014 | -2.02 |
| NM_002335.1 | LRP5 | ILMN_19887 | -2.02 |
| NM_005869.2 | SDCCAG10 | ILMN_3741 | -2.02 |
| XM_939682.1 | LOC149448 | ILMN_36821 | -2.02 |
| NM_006736.5 | DNAJB2 | ILMN_34421 | -2.02 |
| XM_926112.2 | LOC441155 | ILMN_37470 | -2.03 |
| NM_001007794.1 | CEPT1 | ILMN_15134 | -2.03 |
| NM_014180.2 | MRPL22 | ILMN_29349 | -2.03 |
| NM_004563.2 | PCK2 | ILMN_170268 | -2.03 |
| NM_176805.1 | MRPS11 | ILMN_19324 | -2.03 |
| NM_032638.3 | GATA2 | ILMN_20021 | -2.03 |
| NM_138809.3 | CMBL | ILMN_1485 | -2.03 |
| NM_001042549.1 | NSL1 | ILMN_164300 | -2.04 |
| NM_020147.2 | THAP10 | ILMN_182683 | -2.05 |
| NM_001031677.2 | RAB24 | ILMN_25731 | -2.05 |
| NM_006281.2 | STK3 | ILMN_26935 | -2.06 |
| NM_012475.4 | USP21 | ILMN_18019 | -2.06 |
| NM_013299.3 | SAC3D1 | ILMN_9385 | -2.06 |
| XM_498571.2 | LOC440160 | ILMN_33035 | -2.06 |
| NM_006644.2 | HSPH1 | ILMN_1157 | -2.06 |
| NM_012170.2 | FBXO22 | ILMN_5718 | -2.07 |
| NM_007342.1 | NUPL2 | ILMN_2154 | -2.07 |
| NM_001007239.1 | KIAA0859 | ILMN_25045 | -2.07 |
| NM_178314.2 | RILPL1 | ILMN_1609 | -2.08 |
| NM_205847.1 | GMPPA | ILMN_23338 | -2.08 |
| NM_175875.3 | SIX5 | ILMN_21099 | -2.08 |
| NM_003924.2 | PHOX2B | ILMN_14205 | -2.08 |
| NM_001042581.1 | SNUPN | ILMN_176923 | -2.09 |
| NM_197956.1 | C9orf90 | ILMN_16848 | -2.09 |
| NM_018023.3 | YEATS2 | ILMN_13204 | -2.09 |
| NM_000389.2 | CDKN1A | ILMN_16780 | -2.09 |
| NM_013388.4 | PREB | ILMN_6913 | -2.1 |
| NM_006745.3 | SC4MOL | ILMN_2770 | -2.1 |
| NM_153713.1 | LIX1L | ILMN_3572 | -2.1 |
| NM_021222.1 | PRUNE | ILMN_27601 | -2.1 |
| NM_006265.1 | RAD21 | ILMN_171453 | -2.1 |
| XM_930694.1 | LOC642477 | ILMN_36253 | -2.11 |
| NM_001014286.2 | FAM48A | ILMN_1616 | -2.12 |
| NM_024296.3 | CCDC28B | ILMN_26263 | -2.12 |
| NM_001048172.1 | MUTYH | ILMN_164733 | -2.12 |
| NM_001077394.1 | DPH5 | ILMN_175087 | -2.12 |
| NM_024599.3 | RHBDF2 | ILMN_177760 | -2.13 |
| NM_207346.2 | TSEN54 | ILMN_8569 | -2.13 |
| NM_018357.2 | LARP6 | ILMN_25584 | -2.13 |
| NM_001326.2 | CSTF3 | ILMN_27551 | -2.13 |
| NM_018480.2 | TMEM126B | ILMN_18826 | -2.13 |
| NM_016332.2 | SEPX1 | ILMN_7309 | -2.13 |
| NM_022893.2 | BCL11A | ILMN_17359 | -2.14 |
| NM_024813.1 | RPAP2 | ILMN_23904 | -2.14 |
| NM_015523.2 | REXO2 | ILMN_15016 | -2.14 |
| NM_001033566.1 | RHOT1 | ILMN_6821 | -2.15 |
| NM_023937.2 | MRPL34 | ILMN_5839 | -2.15 |
| NM_138458.2 | WDR92 | ILMN_37809 | -2.16 |
| NM_016558.2 | SCAND1 | ILMN_23327 | -2.16 |
| NM_012405.3 | ICMT | ILMN_31192 | -2.16 |
| NM_016086.2 | STYXL1 | ILMN_5068 | -2.16 |
| NM_001827.1 | CKS2 | ILMN_14702 | -2.16 |
| NM_022087.2 | GALNT11 | ILMN_5237 | -2.17 |
| NM_003542.3 | HIST1H4C | ILMN_30043 | -2.17 |
| NM_017958.1 | PLEKHB2 | ILMN_29704 | -2.18 |
| NM_032448.1 | FAM120B | ILMN_10767 | -2.18 |
| NM_058216.1 | RAD51C | ILMN_2944 | -2.19 |
| NM_024057.2 | NUP37 | ILMN_4147 | -2.19 |
| NM_005810.3 | KLRG1 | ILMN_12613 | -2.2 |
| NM_080651.1 | MED30 | ILMN_7158 | -2.2 |
| NM_024068.2 | OBFC2B | ILMN_20552 | -2.2 |
| NM_003746.1 | DNCL1 | ILMN_137049 | -2.2 |
| NM_207380.1 | C15orf52 | ILMN_1132 | -2.22 |
| NM_018297.2 | NGLY1 | ILMN_15318 | -2.22 |
| NM_001042401.1 | C21orf51 | ILMN_179828 | -2.23 |
| NM_178439.3 | GMCL1 | ILMN_3285 | -2.23 |
| NM_001190.2 | BCAT2 | ILMN_2261 | -2.23 |
| NM_031434.2 | TMUB1 | ILMN_11025 | -2.23 |
| NM_013300.1 | C12orf24 | ILMN_24807 | -2.24 |
| NM_006145.1 | DNAJB1 | ILMN_19740 | -2.27 |
| NM_024321.3 | RBM42 | ILMN_182570 | -2.27 |
| NM_001031684.1 | SFRS7 | ILMN_7620 | -2.27 |
| NM_006090.3 | CEPT1 | ILMN_14637 | -2.28 |
| NM_005833.2 | RABEPK | ILMN_4050 | -2.28 |
| XM_934985.1 | LOC400879 | ILMN_31001 | -2.28 |
| NM_032476.2 | MRPS6 | ILMN_17239 | -2.28 |
| NM_001535.2 | PRMT2 | ILMN_10737 | -2.29 |
| NM_016071.2 | MRPS33 | ILMN_4243 | -2.29 |
| NM_001112.2 | ADARB1 | ILMN_30004 | -2.3 |
| XM_935818.1 | FLJ20397 | ILMN_137080 | -2.3 |
| NM_001896.2 | CSNK2A2 | ILMN_16798 | -2.3 |
| NM_003400.3 | XPO1 | ILMN_16600 | -2.3 |
| NM_002225.2 | IVD | ILMN_13293 | -2.31 |
| NM_001535.2 | PRMT2 | ILMN_10737 | -2.31 |
| NM_018164.1 | C12orf11 | ILMN_14707 | -2.31 |
| NM_006310.2 | NPEPPS | ILMN_184074 | -2.31 |
| NM_001077268.1 | ZFYVE19 | ILMN_175347 | -2.32 |
| NM_001042588.1 | SNUPN | ILMN_178280 | -2.32 |
| NM_024527.4 | ABHD8 | ILMN_23791 | -2.32 |
| NM_148973.1 | TNFRSF25 | ILMN_14916 | -2.32 |
| NM_145647.2 | WDR67 | ILMN_20846 | -2.33 |
| NM_002692.2 | POLE2 | ILMN_19705 | -2.33 |
| NM_001813.2 | CENPE | ILMN_7509 | -2.33 |
| NM_024090.1 | ELOVL6 | ILMN_11340 | -2.34 |
| NM_001009608.1 | C20orf94 | ILMN_24801 | -2.35 |
| NM_023005.2 | BAZ1B | ILMN_16290 | -2.35 |
| NM_006416.3 | SLC35A1 | ILMN_23284 | -2.35 |
| NM_202468.1 | GIPC1 | ILMN_21354 | -2.36 |
| NM_138781.2 | LOC113386 | ILMN_12569 | -2.36 |
| NM_017905.3 | TMCO3 | ILMN_19218 | -2.37 |
| NM_198038.1 | NUDT9 | ILMN_12448 | -2.37 |
| NM_138418.2 | C16orf14 | ILMN_9509 | -2.39 |
| NM_138361.3 | LRSAM1 | ILMN_21244 | -2.39 |
| NM_201443.1 | TEAD4 | ILMN_21735 | -2.4 |
| NM_032346.1 | PDCD2L | ILMN_25365 | -2.4 |
| NM_001039141.1 | TRIOBP | ILMN_34620 | -2.4 |
| NM_002598.2 | PDCD2 | ILMN_5469 | -2.4 |
| NM_181702.1 | GEM | ILMN_16170 | -2.41 |
| NM_032728.2 | PPAPDC3 | ILMN_25638 | -2.42 |
| NM_001365.2 | DLG4 | ILMN_164548 | -2.42 |
| NM_180981.1 | MRPL52 | ILMN_3474 | -2.44 |
| NM_058216.1 | RAD51C | ILMN_2944 | -2.44 |
| NM_006158.2 | NEFL | ILMN_22054 | -2.45 |
| NM_032361.1 | THOC3 | ILMN_17969 | -2.45 |
| NM_005836.2 | HRSP12 | ILMN_8062 | -2.46 |
| NM_133646.2 | ZAK | ILMN_5666 | -2.47 |
| XM_944321.1 | LOC402560 | ILMN_42108 | -2.47 |
| NM_005692.3 | ABCF2 | ILMN_14116 | -2.47 |
| NM_020705.1 | TBC1D24 | ILMN_34755 | -2.49 |
| NM_173659.2 | RPUSD3 | ILMN_28804 | -2.49 |
| NM_021971.1 | GMPPB | ILMN_3929 | -2.5 |
| NM_004252.2 | SLC9A3R1 | ILMN_1421 | -2.5 |
| NM_173529.3 | C18orf54 | ILMN_168867 | -2.51 |
| NM_007167.2 | ZMYM6 | ILMN_1275 | -2.51 |
| NM_213596.1 | FOXN4 | ILMN_25618 | -2.53 |
| NM_003129.3 | SQLE | ILMN_521 | -2.53 |
| NM_001039703.1 | NBPF10 | ILMN_45673 | -2.53 |
| NM_001005498.2 | RHBDF2 | ILMN_23030 | -2.54 |
| NM_019116.2 | UBFD1 | ILMN_179383 | -2.54 |
| NM_018983.3 | NOLA1 | ILMN_14204 | -2.54 |
| NM_000051.3 | ATM | ILMN_162851 | -2.54 |
| NM_058246.3 | DNAJB6 | ILMN_7651 | -2.54 |
| NM_006703.2 | NUDT3 | ILMN_25244 | -2.55 |
| XM_941876.1 | BRI3BP | ILMN_139088 | -2.57 |
| NM_003062.1 | SLIT3 | ILMN_18656 | -2.57 |
| NM_007271.2 | STK38 | ILMN_8385 | -2.59 |
| NM_000628.3 | IL10RB | ILMN_26097 | -2.59 |
| NM_007308.1 | SNCA | ILMN_2235 | -2.6 |
| NM_001007157.1 | PHF14 | ILMN_2096 | -2.6 |
| NM_024516.2 | C16orf53 | ILMN_20272 | -2.62 |
| NM_018390.2 | PLCXD1 | ILMN_8273 | -2.62 |
| NM_020153.2 | C11orf60 | ILMN_171038 | -2.63 |
| NM_199487.1 | UQCC | ILMN_16175 | -2.64 |
| NM_003628.3 | PKP4 | ILMN_11784 | -2.65 |
| NM_020749.3 | MTUS1 | ILMN_4658 | -2.65 |
| NM_174942.1 | GAS2L3 | ILMN_5609 | -2.68 |
| NM_005652.2 | TERF2 | ILMN_21134 | -2.71 |
| NM_014847.2 | UBAP2L | ILMN_163836 | -2.72 |
| NM_014161.2 | MRPL18 | ILMN_14120 | -2.72 |
| NM_138444.3 | KCTD12 | ILMN_18501 | -2.75 |
| NM_002095.4 | GTF2E2 | ILMN_4316 | -2.76 |
| NM_178439.3 | GMCL1 | ILMN_3285 | -2.78 |
| NM_018473.2 | THEM2 | ILMN_27212 | -2.79 |
| NM_079837.2 | BANP | ILMN_8638 | -2.85 |
| NM_138720.1 | HIST1H2BD | ILMN_17622 | -2.86 |
| NM_001382.2 | DPAGT1 | ILMN_10306 | -2.87 |
| NM_005873.2 | RGS19 | ILMN_42727 | -2.87 |
| NM_006712.3 | FASTK | ILMN_11299 | -2.9 |
| NM_030805.2 | LMAN2L | ILMN_1985 | -2.91 |
| NM_002166.4 | ID2 | ILMN_28481 | -2.91 |
| NM_012475.4 | USP21 | ILMN_18019 | -2.92 |
| NM_001539.2 | DNAJA1 | ILMN_5819 | -2.92 |
| NM_014254.1 | TMEM5 | ILMN_26271 | -2.94 |
| NM_002028.3 | FNTB | ILMN_171968 | -2.97 |
| XM_926249.2 | LOC642852 | ILMN_40586 | -2.97 |
| NM_002643.3 | PIGF | ILMN_15261 | -2.98 |
| NM_001007157.1 | PHF14 | ILMN_2096 | -2.98 |
| NM_001008566.1 | TPST2 | ILMN_13248 | -2.99 |
| NM_019858.1 | GPR162 | ILMN_27943 | -2.99 |
| NM_005644.2 | TAF12 | ILMN_3797 | -3.02 |
| NM_004456.3 | EZH2 | ILMN_25740 | -3.02 |
| NM_033115.2 | MGC16169 | ILMN_16160 | -3.04 |
| NM_006597.3 | HSPA8 | ILMN_181529 | -3.04 |
| NM_000856.3 | GUCY1A3 | ILMN_11680 | -3.07 |
| NR_001445.1 | RN7SK | ILMN_14457 | -3.08 |
| NM_138720.1 | HIST1H2BD | ILMN_17622 | -3.08 |
| NM_182533.1 | C1orf86 | ILMN_2880 | -3.12 |
| NM_032772.3 | ZNF503 | ILMN_2048 | -3.14 |
| NM_014140.2 | SMARCAL1 | ILMN_19734 | -3.15 |
| NM_002613.3 | PDPK1 | ILMN_27765 | -3.17 |
| NM_014322.2 | OPN3 | ILMN_166169 | -3.19 |
| NM_000913.3 | OPRL1 | ILMN_6491 | -3.19 |
| NM_033212.2 | CCDC102A | ILMN_12942 | -3.24 |
| NM_015948.2 | SLC35B3 | ILMN_20545 | -3.27 |
| NM_138807.2 | C3orf31 | ILMN_9705 | -3.3 |
| XM_925818.1 | LOC642282 | ILMN_41968 | -3.32 |
| NM_000819.3 | GART | ILMN_22974 | -3.38 |
| NM_153201.1 | HSPA8 | ILMN_14829 | -3.42 |
| NM_003512.3 | HIST1H2AC | ILMN_26493 | -3.52 |
| NM_001037675.1 | NBPF20 | ILMN_26956 | -3.53 |
| NM_022079.2 | HERC4 | ILMN_8869 | -3.57 |
| NM_014941.1 | MORC2 | ILMN_12502 | -3.74 |
| NM_079837.2 | BANP | ILMN_8638 | -3.81 |
| NM_007198.2 | PROSC | ILMN_23472 | -3.82 |
| NM_001040142.1 | SCN2A | ILMN_167124 | -3.84 |
| NM_080723.3 | NRSN1 | ILMN_178353 | -4.12 |
| NM_004316.2 | ASCL1 | ILMN_23892 | -4.12 |
| NM_006860.2 | RABL4 | ILMN_4559 | -4.18 |
| NM_002167.2 | ID3 | ILMN_6829 | -4.56 |
| NM_002166.4 | ID2 | ILMN_28481 | -4.76 |
| NM_005345.4 | HSPA1A | ILMN_6623 | -6.07 |
| NM_005346.3 | HSPA1B | ILMN_25549 | -6.64 |
