# Supplementary material for: Minimal Peroxide Exposure of Neuronal Cells Induces Multifaceted Adaptive Responses
Source: PLoS One. 2010 Dec 17;5(12):e14352. doi: 10.1371/journal.pone.0014352 (PMC3003681; doi:10.1371/journal.pone.0014352)
Supplement: Table S11 — BDNF-significantly regulated genes after 8 hours of stimulation in the control state SH-SY5Y cells. Each significantly regulated gene is described via its accession number (ACCESSION), Gene Symbol (SYMBOL), Illumina array transcript designation (TRANSCRIPT). For each gene the z-ratio of expression compared to untreated cells after 8 hours of ligand stimulation is displayed (CTL BDNF 8). (1.26 MB DOC) [file pone.0014352.s018.doc]

**Table S11. BDNF-significantly regulated genes after 8 hours of stimulation in the control state SH-SY5Y cells**. Each significantly regulated gene is described via its accession number (ACCESSION), Gene Symbol (SYMBOL), Illumina array transcript designation (TRANSCRIPT). For each gene the z-ratio of expression compared to un-treated cells after 8 hours of ligand stimulation is displayed (CTL BDNF 8).

| **ACCESSION** | **SYMBOL** | **TRANSCRIPT** | **CTL BDNF 8** |
| --- | --- | --- | --- |
| NM_000584.2 | IL8 | ILMN_179575 | 8.23 |
| XM_935588.1 | LOC641848 | ILMN_45490 | 4.98 |
| NM_001003.2 | RPLP1 | ILMN_23181 | 4.74 |
| XR_015514.1 | LOC730746 | ILMN_163533 | 4.58 |
| NM_006157.2 | NELL1 | ILMN_2560 | 4.48 |
| NM_199436.1 | SPAST | ILMN_15461 | 4.35 |
| NM_006135.1 | CAPZA1 | ILMN_137637 | 4.32 |
| XM_375152.3 | LOC400304 | ILMN_46003 | 4.29 |
| XM_926231.1 | P704P | ILMN_36679 | 4.28 |
| NM_001099285.1 | PTMA | ILMN_306831 | 4.19 |
| XM_937113.2 | LOC647436 | ILMN_44829 | 4.12 |
| NM_148174.2 | AZIN1 | ILMN_4931 | 4.05 |
| NM_001077188.1 | HS6ST2 | ILMN_182242 | 4.04 |
| NR_003040.1 | LOC649946 | ILMN_169528 | 4.04 |
| NM_004768.2 | SFRS11 | ILMN_4847 | 4.03 |
| NR_002315.1 | LOC440926 | ILMN_19720 | 4.02 |
| NM_207035.1 | C1orf63 | ILMN_22593 | 4 |
| XR_017492.1 | LOC644330 | ILMN_164787 | 4 |
| NM_001001391.1 | CD44 | ILMN_10947 | 3.89 |
| NM_004156.2 | PPP2CB | ILMN_21592 | 3.88 |
| NM_006107.2 | CROP | ILMN_10300 | 3.88 |
| NM_001024921.2 | RPL9 | ILMN_8640 | 3.82 |
| XM_370865.4 | LOC388122 | ILMN_46143 | 3.79 |
| NM_002291.1 | LAMB1 | ILMN_182874 | 3.78 |
| NM_030793.3 | FBXO38 | ILMN_4373 | 3.72 |
| NM_001008219.1 | AMY1C | ILMN_28222 | 3.71 |
| XM_938089.2 | LOC643007 | ILMN_31054 | 3.69 |
| XM_933893.1 | LOC389672 | ILMN_35589 | 3.6 |
| NM_001040456.1 | RHBDD2 | ILMN_168345 | 3.6 |
| NM_001024646.1 | CLK1 | ILMN_27286 | 3.55 |
| NM_144736.3 | PRO1853 | ILMN_15591 | 3.53 |
| XM_292963.6 | LOC643997 | ILMN_39721 | 3.51 |
| NM_004038.3 | AMY1A | ILMN_176350 | 3.5 |
| XR_018327.1 | LOC648343 | ILMN_163789 | 3.48 |
| XM_938988.1 | LOC402221 | ILMN_35678 | 3.48 |
| XR_016048.1 | MGC40489 | ILMN_171153 | 3.44 |
| NM_020317.3 | C1orf63 | ILMN_22487 | 3.42 |
| NM_007285.6 | GABARAPL2 | ILMN_9805 | 3.4 |
| NM_001031710.1 | KLHL7 | ILMN_8698 | 3.31 |
| NM_002673.3 | PLXNB1 | ILMN_22628 | 3.3 |
| NM_000199.2 | SGSH | ILMN_7542 | 3.27 |
| NM_004779.4 | CNOT8 | ILMN_10063 | 3.25 |
| NM_005154.2 | USP8 | ILMN_15425 | 3.21 |
| NM_001496.3 | GFRA3 | ILMN_8392 | 3.2 |
| NM_006123.2 | IDS | ILMN_17605 | 3.18 |
| NM_004859.3 | CLTC | ILMN_171089 | 3.18 |
| NM_002737.2 | PRKCA | ILMN_24085 | 3.12 |
| NM_031943.1 | IFP38 | ILMN_9478 | 3.09 |
| NM_014363.3 | SACS | ILMN_180142 | 3.07 |
| NM_024663.3 | NPEPL1 | ILMN_175218 | 3.06 |
| NM_001080484.1 | KIAA1751 | ILMN_180591 | 3.05 |
| XM_940333.2 | LOC651202 | ILMN_37363 | 3.05 |
| XM_497072.2 | LOC389787 | ILMN_45784 | 3.04 |
| NM_178324.1 | SPTLC1 | ILMN_7889 | 3.04 |
| NM_175923.3 | MGC42630 | ILMN_138411 | 3.02 |
| NM_005245.3 | FAT | ILMN_24617 | 3.02 |
| NR_003028.1 | SNORA25 | ILMN_171656 | 2.99 |
| NM_004598.3 | SPOCK1 | ILMN_25886 | 2.99 |
| NM_000978.3 | RPL23 | ILMN_8866 | 2.98 |
| NM_003617.2 | RGS5 | ILMN_167992 | 2.96 |
| NM_181054.1 | HIF1A | ILMN_9514 | 2.95 |
| NM_052879.3 | LARP4 | ILMN_2132 | 2.95 |
| XM_941684.2 | LOC220433 | ILMN_46655 | 2.94 |
| NM_001040456.1 | RHBDD2 | ILMN_168345 | 2.93 |
| NM_005010.3 | NRCAM | ILMN_8955 | 2.92 |
| NM_002266.2 | KPNA2 | ILMN_14206 | 2.89 |
| NM_014016.2 | SACM1L | ILMN_19838 | 2.89 |
| NM_004238.1 | TRIP12 | ILMN_21755 | 2.88 |
| NM_003972.2 | BTAF1 | ILMN_8616 | 2.88 |
| NM_005721.3 | ACTR3 | ILMN_11792 | 2.87 |
| NM_001033506.1 | CSTF3 | ILMN_27049 | 2.86 |
| XM_936731.1 | LOC647673 | ILMN_33594 | 2.86 |
| NM_152398.2 | OCIAD2 | ILMN_18246 | 2.85 |
| XM_938297.1 | LOC402644 | ILMN_30715 | 2.85 |
| NM_001752.2 | CAT | ILMN_170506 | 2.83 |
| NM_173854.4 | SLC41A1 | ILMN_2825 | 2.83 |
| XM_935770.1 | LOC641992 | ILMN_31870 | 2.83 |
| NM_001035005.2 | C18orf32 | ILMN_26126 | 2.83 |
| XM_938599.2 | LOC441377 | ILMN_31681 | 2.83 |
| NM_001037533.1 | GON4L | ILMN_14180 | 2.82 |
| XR_015809.1 | LOC728973 | ILMN_168278 | 2.82 |
| NM_004145.2 | MYO9B | ILMN_25414 | 2.8 |
| NM_001017421.1 | FKSG30 | ILMN_2393 | 2.8 |
| NM_145913.2 | SLC5A8 | ILMN_7082 | 2.79 |
| NR_002203.1 | FTHL8 | ILMN_16227 | 2.78 |
| NM_000189.4 | HK2 | ILMN_164962 | 2.77 |
| NM_199053.1 | C4orf41 | ILMN_8562 | 2.77 |
| NM_024525.2 | TTC13 | ILMN_164005 | 2.77 |
| XM_938755.2 | LOC653773 | ILMN_44662 | 2.76 |
| XM_927235.1 | LOC643985 | ILMN_32781 | 2.75 |
| NM_001412.3 | EIF1AX | ILMN_22164 | 2.75 |
| NM_004508.2 | IDI1 | ILMN_20349 | 2.74 |
| NM_006004.1 | UQCRH | ILMN_138507 | 2.74 |
| NM_001275.3 | CHGA | ILMN_23390 | 2.73 |
| NM_006185.2 | NUMA1 | ILMN_25058 | 2.71 |
| NM_001627.2 | ALCAM | ILMN_164638 | 2.71 |
| XM_941195.2 | LOC388621 | ILMN_42661 | 2.67 |
| NM_012322.1 | LSM5 | ILMN_17896 | 2.66 |
| NM_001095.2 | ACCN2 | ILMN_27416 | 2.65 |
| NM_001079673.1 | FNDC3A | ILMN_167386 | 2.65 |
| NM_004786.1 | TXNL1 | ILMN_7321 | 2.64 |
| NM_016617.1 | UFM1 | ILMN_12438 | 2.63 |
| NM_001007246.1 | BRWD1 | ILMN_28841 | 2.63 |
| NM_012424.2 | RPS6KC1 | ILMN_165437 | 2.62 |
| XM_941155.2 | LOC651894 | ILMN_33374 | 2.61 |
| NM_003913.3 | PRPF4B | ILMN_139391 | 2.6 |
| NM_020133.2 | AGPAT4 | ILMN_24920 | 2.6 |
| NM_001077628.1 | APH1A | ILMN_180233 | 2.6 |
| NM_016038.2 | SBDS | ILMN_15766 | 2.6 |
| NM_032810.2 | ATAD1 | ILMN_175726 | 2.58 |
| NR_002205.1 | FTHL12 | ILMN_16447 | 2.58 |
| NM_014822.1 | SEC24D | ILMN_7795 | 2.57 |
| NR_002204.1 | FTHL11 | ILMN_16343 | 2.57 |
| XM_001133089.1 | LOC731640 | ILMN_161930 | 2.57 |
| NM_000786.2 | CYP51A1 | ILMN_161878 | 2.56 |
| NM_032788.1 | ZNF514 | ILMN_14476 | 2.55 |
| NM_015016.1 | MAST3 | ILMN_308510 | 2.55 |
| NM_003458.3 | BSN | ILMN_22754 | 2.55 |
| NM_177972.1 | TUB | ILMN_11520 | 2.55 |
| NM_005010.3 | NRCAM | ILMN_8955 | 2.55 |
| NM_003666.2 | BLZF1 | ILMN_21927 | 2.55 |
| NM_006045.1 | ATP9A | ILMN_176431 | 2.54 |
| NM_007055.2 | POLR3A | ILMN_175644 | 2.53 |
| NM_019892.3 | INPP5E | ILMN_11866 | 2.52 |
| NM_001083585.1 | RABEP1 | ILMN_307418 | 2.51 |
| NM_183422.1 | TSC22D1 | ILMN_166165 | 2.51 |
| NM_001677.3 | ATP1B1 | ILMN_25542 | 2.51 |
| NM_016424.3 | CROP | ILMN_11116 | 2.5 |
| NM_004071.2 | CLK1 | ILMN_162592 | 2.5 |
| NM_001005849.1 | SUMO2 | ILMN_16713 | 2.5 |
| NM_016608.1 | ARMCX1 | ILMN_9172 | 2.49 |
| NM_006360.3 | EIF3M | ILMN_19862 | 2.49 |
| XR_019339.1 | LOC643668 | ILMN_179350 | 2.48 |
| NM_017821.3 | RHBDL2 | ILMN_20003 | 2.48 |
| NM_004687.3 | MTMR4 | ILMN_163329 | 2.48 |
| NM_021239.1 | RBM25 | ILMN_18687 | 2.48 |
| XM_944716.1 | LOC440704 | ILMN_34488 | 2.47 |
| NM_006197.3 | PCM1 | ILMN_14472 | 2.47 |
| NM_015245.2 | ANKS1A | ILMN_25376 | 2.46 |
| NR_002201.1 | FTHL3 | ILMN_27691 | 2.44 |
| NM_002128.4 | HMGB1 | ILMN_23421 | 2.42 |
| NM_203364.2 | CAPRIN1 | ILMN_9771 | 2.42 |
| NM_005443.4 | PAPSS1 | ILMN_171260 | 2.4 |
| NM_080386.1 | TUBA3D | ILMN_30319 | 2.39 |
| NM_001031712.2 | TRMT11 | ILMN_8801 | 2.39 |
| NM_172014.1 | TNFSF14 | ILMN_9666 | 2.39 |
| NM_006421.3 | ARFGEF1 | ILMN_164295 | 2.38 |
| NM_173797.2 | PAPD4 | ILMN_2190 | 2.36 |
| NM_001788.4 | 7-Sep | ILMN_25070 | 2.35 |
| NM_001024071.1 | GCH1 | ILMN_14690 | 2.35 |
| NM_006572.3 | GNA13 | ILMN_173569 | 2.35 |
| NM_007373.2 | SHOC2 | ILMN_12623 | 2.34 |
| NM_016457.3 | PRKD2 | ILMN_23825 | 2.34 |
| NM_020452.2 | ATP8B2 | ILMN_19940 | 2.34 |
| XM_001132754.1 | LOC728734 | ILMN_169578 | 2.33 |
| XM_944104.2 | LOC653232 | ILMN_41197 | 2.33 |
| NM_002734.3 | PRKAR1A | ILMN_18925 | 2.32 |
| NR_002808.1 | C14orf85 | ILMN_14639 | 2.32 |
| NM_032810.2 | ATAD1 | ILMN_175726 | 2.31 |
| XR_018923.1 | LOC648210 | ILMN_162972 | 2.31 |
| NM_133459.1 | CCBE1 | ILMN_6075 | 2.3 |
| NM_002609.3 | PDGFRB | ILMN_25767 | 2.3 |
| NM_144967.2 | FLJ30058 | ILMN_29759 | 2.29 |
| NM_023012.4 | RSRC2 | ILMN_17793 | 2.29 |
| NM_019024.1 | HEATR5B | ILMN_183109 | 2.29 |
| NM_005781.4 | TNK2 | ILMN_5336 | 2.29 |
| NM_000971.3 | RPL7 | ILMN_26351 | 2.29 |
| NM_015690.2 | STK36 | ILMN_15506 | 2.28 |
| NM_019600.1 | KIAA1370 | ILMN_2444 | 2.27 |
| NM_003906.3 | MCM3AP | ILMN_19614 | 2.27 |
| NM_032242.2 | PLXNA1 | ILMN_912 | 2.26 |
| NM_001023567.2 | GOLGA8B | ILMN_14405 | 2.26 |
| NM_016028.4 | SUV420H1 | ILMN_29861 | 2.25 |
| NM_173518.2 | C8orf45 | ILMN_22241 | 2.25 |
| NM_001031.4 | RPS28 | ILMN_992 | 2.25 |
| NM_004458.1 | ACSL4 | ILMN_12915 | 2.24 |
| NM_018981.1 | DNAJC10 | ILMN_19735 | 2.24 |
| NM_080491.1 | GAB2 | ILMN_3317 | 2.24 |
| NM_001008735.1 | HMG1L1 | ILMN_22757 | 2.24 |
| NM_012223.2 | MYO1B | ILMN_175452 | 2.23 |
| NM_022910.1 | NDRG4 | ILMN_8824 | 2.23 |
| NM_014478.4 | RCP9 | ILMN_22485 | 2.23 |
| NM_001010915.1 | PTPLAD2 | ILMN_6355 | 2.22 |
| NM_006265.1 | RAD21 | ILMN_171453 | 2.22 |
| NM_001006.3 | RPS3A | ILMN_28872 | 2.22 |
| NM_001034194.1 | EXOSC9 | ILMN_26957 | 2.21 |
| XM_942687.1 | LOC654189 | ILMN_30702 | 2.21 |
| NM_198795.1 | TDRD1 | ILMN_162663 | 2.21 |
| NM_002473.3 | MYH9 | ILMN_183555 | 2.21 |
| NM_024612.3 | DHX40 | ILMN_1864 | 2.21 |
| XM_377933.3 | LOC402251 | ILMN_45342 | 2.21 |
| NM_020748.1 | INTS2 | ILMN_1948 | 2.2 |
| NM_152730.4 | C6orf170 | ILMN_17001 | 2.2 |
| NM_172097.1 | CATSPER2 | ILMN_23478 | 2.2 |
| NM_002160.2 | TNC | ILMN_14948 | 2.19 |
| NM_004897.2 | MINPP1 | ILMN_29353 | 2.18 |
| NM_030881.2 | DDX17 | ILMN_28024 | 2.18 |
| NM_007112.3 | THBS3 | ILMN_10000 | 2.17 |
| NM_018416.2 | FOXJ2 | ILMN_165896 | 2.17 |
| NM_013361.3 | ZNF223 | ILMN_166150 | 2.16 |
| NM_001819.1 | CHGB | ILMN_20449 | 2.16 |
| NM_014345.1 | ZNF318 | ILMN_29701 | 2.15 |
| NM_016623.3 | FAM49B | ILMN_14248 | 2.15 |
| NM_138394.2 | HNRPLL | ILMN_4564 | 2.15 |
| NM_025191.2 | EDEM3 | ILMN_15796 | 2.15 |
| NM_004779.4 | CNOT8 | ILMN_172926 | 2.15 |
| NM_001079537.1 | TRAPPC6B | ILMN_164643 | 2.14 |
| XM_937107.1 | LOC648057 | ILMN_32589 | 2.14 |
| NM_005128.2 | DOPEY2 | ILMN_164626 | 2.14 |
| NM_019591.2 | ZNF26 | ILMN_3233 | 2.14 |
| NM_152902.3 | TIPRL | ILMN_13476 | 2.13 |
| NM_021218.1 | C9orf80 | ILMN_27473 | 2.13 |
| NM_018479.2 | ECHDC1 | ILMN_1493 | 2.12 |
| NM_015503.1 | SH2B1 | ILMN_26582 | 2.12 |
| NM_025058.3 | TRIM46 | ILMN_18492 | 2.12 |
| XM_940209.1 | KIAA0194 | ILMN_37512 | 2.12 |
| NM_021942.4 | C4orf41 | ILMN_8900 | 2.12 |
| NM_203459.1 | CAMSAP1L1 | ILMN_14735 | 2.12 |
| NR_003659.1 | FAM39DP | ILMN_307683 | 2.12 |
| NM_014000.2 | VCL | ILMN_27566 | 2.12 |
| NM_020474.2 | GALNT1 | ILMN_164550 | 2.11 |
| NM_015130.2 | TBC1D9 | ILMN_25527 | 2.11 |
| NM_001251.2 | CD68 | ILMN_5188 | 2.11 |
| NM_005095.2 | ZMYM4 | ILMN_26259 | 2.11 |
| XM_929199.1 | LOC644250 | ILMN_30796 | 2.11 |
| NM_003086.2 | SNAPC4 | ILMN_180505 | 2.11 |
| NM_017514.2 | PLXNA3 | ILMN_162939 | 2.1 |
| NM_014056.1 | HIGD1A | ILMN_2731 | 2.1 |
| NM_001019.4 | RPS15A | ILMN_27642 | 2.1 |
| NM_014811.3 | KIAA0649 | ILMN_9360 | 2.09 |
| NM_016472.3 | C14orf129 | ILMN_7725 | 2.09 |
| NM_003589.2 | CUL4A | ILMN_28629 | 2.09 |
| NM_019106.4 | 3-Sep | ILMN_4065 | 2.09 |
| NM_001079673.1 | FNDC3A | ILMN_167386 | 2.08 |
| NM_020897.1 | HCN3 | ILMN_20127 | 2.08 |
| NM_021227.2 | DC2 | ILMN_24748 | 2.07 |
| NM_014946.3 | SPAST | ILMN_14993 | 2.07 |
| NM_173666.1 | DTWD2 | ILMN_25915 | 2.07 |
| XM_939687.2 | LOC653658 | ILMN_33948 | 2.07 |
| NM_014014.2 | ASCC3L1 | ILMN_18834 | 2.06 |
| NM_003082.2 | SNAPC1 | ILMN_177713 | 2.06 |
| NM_001077199.1 | SFRS12 | ILMN_180296 | 2.06 |
| NM_006472.2 | TXNIP | ILMN_9057 | 2.06 |
| NM_012433.2 | SF3B1 | ILMN_2494 | 2.06 |
| NM_018561.3 | USP49 | ILMN_24018 | 2.06 |
| NM_001632.3 | ALPP | ILMN_25184 | 2.06 |
| NR_001562.1 | ANXA2P1 | ILMN_10494 | 2.05 |
| NR_003277.1 | LOC728643 | ILMN_183126 | 2.05 |
| XM_371461.4 | KIAA1671 | ILMN_42090 | 2.04 |
| NM_025251.1 | KIAA1688 | ILMN_28510 | 2.04 |
| NM_014614.1 | PSME4 | ILMN_164803 | 2.04 |
| NM_019001.2 | XRN1 | ILMN_8924 | 2.03 |
| NM_006197.2 | PCM1 | ILMN_173657 | 2.03 |
| NM_033505.2 | SELI | ILMN_18750 | 2.02 |
| NM_020248.2 | CTNNBIP1 | ILMN_23888 | 2.02 |
| NM_015433.2 | FAM119B | ILMN_17350 | 2.02 |
| NM_002467.3 | MYC | ILMN_172213 | 2.02 |
| NR_002200.1 | FTHL2 | ILMN_15867 | 2.02 |
| NM_015308.1 | FNBP4 | ILMN_25895 | 2.02 |
| XM_939726.2 | LOC388532 | ILMN_45940 | 2.02 |
| NM_001078.2 | VCAM1 | ILMN_3875 | 2.01 |
| NM_025152.1 | NUBPL | ILMN_25397 | 2.01 |
| NM_003925.1 | MBD4 | ILMN_18891 | 2.01 |
| XM_932717.2 | LOC643224 | ILMN_34053 | 2 |
| NM_021130.3 | PPIA | ILMN_25214 | 2 |
| NM_033083.6 | EAF1 | ILMN_173601 | 2 |
| XM_935589.1 | LOC641849 | ILMN_45563 | 2 |
| NM_015306.1 | USP24 | ILMN_309418 | 2 |
| NM_016121.3 | KCTD3 | ILMN_179202 | 2 |
| NM_152322.2 | BTBD11 | ILMN_506 | 1.99 |
| NM_001347.2 | DGKQ | ILMN_27065 | 1.99 |
| NM_015902.4 | UBR5 | ILMN_178959 | 1.99 |
| NM_005669.4 | REEP5 | ILMN_21319 | 1.99 |
| NM_014949.2 | KIAA0907 | ILMN_6810 | 1.99 |
| NM_001418.3 | EIF4G2 | ILMN_19314 | 1.99 |
| NM_018343.1 | RIOK2 | ILMN_16482 | 1.98 |
| NM_002230.1 | JUP | ILMN_3789 | 1.98 |
| NM_001031623.2 | ZNF451 | ILMN_990 | 1.97 |
| NM_173602.2 | DIP2B | ILMN_179302 | 1.97 |
| NM_017641.2 | KIF21A | ILMN_25772 | 1.97 |
| NM_002473.3 | MYH9 | ILMN_183555 | 1.97 |
| NM_002685.2 | EXOSC10 | ILMN_25853 | 1.96 |
| NM_018263.4 | ASXL2 | ILMN_7971 | 1.95 |
| NM_001001787.1 | ATP1B1 | ILMN_10855 | 1.95 |
| NM_003069.2 | SMARCA1 | ILMN_181613 | 1.94 |
| NM_021045.1 | ZNF248 | ILMN_27704 | 1.93 |
| NM_173694.3 | ATP11C | ILMN_169690 | 1.93 |
| NM_022451.9 | NOC3L | ILMN_11360 | 1.93 |
| NM_016284.3 | CNOT1 | ILMN_169268 | 1.93 |
| NM_001006.3 | RPS3A | ILMN_28872 | 1.93 |
| NM_024909.1 | C6orf134 | ILMN_21139 | 1.92 |
| XM_945045.1 | LOC649679 | ILMN_34833 | 1.92 |
| NR_001283.1 | TOP1P2 | ILMN_4755 | 1.92 |
| XM_001133677.1 | LOC729264 | ILMN_170805 | 1.91 |
| NM_007346.2 | OGFR | ILMN_12520 | 1.91 |
| NM_031372.1 | HNRPDL | ILMN_15196 | 1.91 |
| NM_001039705.1 | TRO | ILMN_32618 | 1.91 |
| NR_002197.1 | LOC143543 | ILMN_17694 | 1.9 |
| NM_001018111.1 | PODXL | ILMN_24120 | 1.9 |
| NM_001100417.1 | C14orf130 | ILMN_307543 | 1.9 |
| NM_002687.3 | PNN | ILMN_24088 | 1.9 |
| NM_001080973.1 | IL17RD | ILMN_179882 | 1.9 |
| NM_176811.2 | NLRP8 | ILMN_169055 | 1.9 |
| NM_015026.1 | MON2 | ILMN_19004 | 1.89 |
| NM_018846.2 | KLHL7 | ILMN_21425 | 1.89 |
| NM_017991.3 | FLJ10081 | ILMN_469 | 1.89 |
| NM_001030.3 | RPS27 | ILMN_5932 | 1.89 |
| NM_001440.2 | EXTL3 | ILMN_10725 | 1.88 |
| NM_004788.2 | UBE4A | ILMN_175730 | 1.88 |
| NM_004698.1 | PRPF3 | ILMN_6388 | 1.88 |
| NM_003622.2 | PPFIBP1 | ILMN_172147 | 1.87 |
| NM_014071.2 | NCOA6 | ILMN_20599 | 1.87 |
| XM_937691.1 | LOC648622 | ILMN_42936 | 1.87 |
| NR_002182.1 | NACAP1 | ILMN_14666 | 1.86 |
| NM_001034996.1 | RPL14 | ILMN_2719 | 1.86 |
| NM_020728.1 | FAM62B | ILMN_19173 | 1.86 |
| NM_018044.2 | NSUN5 | ILMN_895 | 1.85 |
| NM_012326.2 | MAPRE3 | ILMN_1035 | 1.85 |
| NM_018639.3 | WSB2 | ILMN_162438 | 1.85 |
| NM_001037334.1 | USP14 | ILMN_12721 | 1.85 |
| XM_495939.3 | KIAA1545 | ILMN_40920 | 1.84 |
| NM_014771.2 | RNF40 | ILMN_5177 | 1.84 |
| NM_000938.1 | POLR2B | ILMN_12118 | 1.84 |
| NM_001387.2 | DPYSL3 | ILMN_23309 | 1.84 |
| NM_014962.2 | BTBD3 | ILMN_180757 | 1.83 |
| NM_001040439.1 | MAPK8IP3 | ILMN_174436 | 1.83 |
| NM_007144.2 | PCGF2 | ILMN_11878 | 1.83 |
| NM_032017.1 | STK40 | ILMN_25410 | 1.83 |
| NM_003234.1 | TFRC | ILMN_12909 | 1.83 |
| XM_930995.1 | LOC653086 | ILMN_31021 | 1.83 |
| NM_016245.3 | HSD17B11 | ILMN_12219 | 1.82 |
| NM_005342.2 | HMGB3 | ILMN_8326 | 1.82 |
| XM_937928.1 | LOC347376 | ILMN_31523 | 1.82 |
| NM_000572.2 | IL10 | ILMN_9173 | 1.82 |
| NM_012479.2 | YWHAG | ILMN_172249 | 1.82 |
| NM_003878.1 | GGH | ILMN_9870 | 1.81 |
| NM_013241.2 | FHOD1 | ILMN_14837 | 1.81 |
| NM_133462.2 | TTC14 | ILMN_6310 | 1.81 |
| NM_006628.4 | ARPP-19 | ILMN_2093 | 1.81 |
| NM_001079842.1 | OCIAD1 | ILMN_163712 | 1.81 |
| NM_024546.2 | C13orf7 | ILMN_137373 | 1.8 |
| NM_018318.3 | CCDC91 | ILMN_29700 | 1.8 |
| NM_000271.3 | NPC1 | ILMN_30618 | 1.8 |
| NM_004730.1 | ETF1 | ILMN_9222 | 1.8 |
| NM_004423.3 | DVL3 | ILMN_11726 | 1.8 |
| NM_144635.3 | FAM131A | ILMN_2542 | 1.8 |
| NM_198480.2 | ZNF615 | ILMN_26774 | 1.79 |
| NM_004906.3 | WTAP | ILMN_24202 | 1.79 |
| NM_015560.1 | OPA1 | ILMN_10977 | 1.79 |
| NM_003220.2 | TFAP2A | ILMN_17128 | 1.78 |
| NM_014382.2 | ATP2C1 | ILMN_16216 | 1.78 |
| NM_015477.1 | SIN3A | ILMN_14108 | 1.78 |
| NM_172249.1 | CSF2RA | ILMN_5061 | 1.78 |
| NM_022098.2 | XPNPEP3 | ILMN_23195 | 1.78 |
| NM_005497.3 | GJC1 | ILMN_3556 | 1.78 |
| NM_173602.2 | DIP2B | ILMN_24944 | 1.77 |
| NM_001031744.1 | LOC158160 | ILMN_21155 | 1.77 |
| NM_022173.1 | TIA1 | ILMN_29910 | 1.77 |
| NM_181673.1 | OGT | ILMN_4866 | 1.77 |
| NM_016257.2 | HPCAL4 | ILMN_25356 | 1.76 |
| NM_015289.2 | VPS39 | ILMN_5610 | 1.76 |
| NM_012400.2 | PLA2G2D | ILMN_163941 | 1.76 |
| NM_014810.3 | CEP350 | ILMN_19424 | 1.76 |
| NM_001011537.1 | FYTTD1 | ILMN_5513 | 1.76 |
| NM_020921.3 | NIN | ILMN_172996 | 1.76 |
| NM_014395.1 | DAPP1 | ILMN_24094 | 1.76 |
| NM_014765.1 | TOMM20 | ILMN_20433 | 1.76 |
| NM_022483.3 | C5orf28 | ILMN_5037 | 1.76 |
| NM_001995.2 | ACSL1 | ILMN_12367 | 1.75 |
| NM_003831.2 | RIOK3 | ILMN_19558 | 1.75 |
| NM_015475.3 | FAM98A | ILMN_16819 | 1.75 |
| NM_001031827.1 | BOLA2 | ILMN_4509 | 1.75 |
| XM_945571.1 | ANKRD13D | ILMN_138370 | 1.74 |
| NM_033426.2 | KIAA1737 | ILMN_24671 | 1.74 |
| NM_014779.2 | TSC22D2 | ILMN_5940 | 1.74 |
| NM_007049.2 | BTN2A1 | ILMN_6995 | 1.74 |
| NM_019119.3 | PCDHB9 | ILMN_23442 | 1.74 |
| NM_001039797.1 | FLJ46309 | ILMN_30948 | 1.74 |
| NM_178231.1 | ALS2CR14 | ILMN_947 | 1.73 |
| NM_000787.3 | DBH | ILMN_25962 | 1.73 |
| NM_004402.2 | DFFB | ILMN_14684 | 1.72 |
| NM_020040.3 | TUBB4Q | ILMN_177504 | 1.72 |
| NM_015085.3 | GARNL4 | ILMN_163593 | 1.72 |
| XM_944439.2 | LOC653994 | ILMN_38572 | 1.71 |
| NM_170686.1 | ZNF398 | ILMN_16491 | 1.71 |
| NM_033446.1 | FAM125B | ILMN_20760 | 1.71 |
| NM_145687.2 | MAP4K4 | ILMN_28871 | 1.71 |
| NM_012428.2 | NPTN | ILMN_175751 | 1.71 |
| NM_006999.3 | POLS | ILMN_866 | 1.71 |
| NM_022167.2 | XYLT2 | ILMN_26042 | 1.71 |
| NM_022494.1 | ZDHHC6 | ILMN_1193 | 1.71 |
| XR_017397.1 | LOC644029 | ILMN_163901 | 1.71 |
| NM_006145.1 | DNAJB1 | ILMN_19740 | 1.7 |
| NM_015447.1 | CAMSAP1 | ILMN_815 | 1.7 |
| NM_005385.3 | NKTR | ILMN_23378 | 1.7 |
| NM_017941.3 | C17orf80 | ILMN_21070 | 1.69 |
| NM_012089.1 | ABCB10 | ILMN_4642 | 1.69 |
| NM_014862.3 | ARNT2 | ILMN_13881 | 1.69 |
| NM_001080485.1 | ZNF275 | ILMN_180340 | 1.69 |
| XR_016986.1 | LOC643668 | ILMN_172192 | 1.69 |
| NM_001008661.1 | CCBL2 | ILMN_1120 | 1.69 |
| NM_004339.2 | PTTG1IP | ILMN_29076 | 1.69 |
| NM_032421.2 | CLIP2 | ILMN_14847 | 1.68 |
| NM_032826.3 | SLC35B4 | ILMN_19120 | 1.68 |
| NM_002959.4 | SORT1 | ILMN_165748 | 1.68 |
| NM_025132.3 | WDR19 | ILMN_11749 | 1.68 |
| XM_936495.2 | LOC647346 | ILMN_36174 | 1.68 |
| NM_000090.3 | COL3A1 | ILMN_182795 | 1.68 |
| NM_006148.1 | LASP1 | ILMN_27039 | 1.68 |
| NM_015496.3 | KIAA1429 | ILMN_21574 | 1.67 |
| NM_017741.3 | C4orf30 | ILMN_172318 | 1.67 |
| NM_001012643.2 | LOC339344 | ILMN_6535 | 1.67 |
| NM_005520.1 | HNRPH1 | ILMN_4782 | 1.67 |
| NM_001002878.1 | THOC5 | ILMN_13820 | 1.67 |
| NM_014747.2 | RIMS3 | ILMN_21581 | 1.67 |
| NM_031263.1 | HNRPK | ILMN_16515 | 1.67 |
| NM_000969.3 | RPL5 | ILMN_4986 | 1.67 |
| NM_032794.1 | SLC44A4 | ILMN_14709 | 1.67 |
| NM_020850.1 | RANBP10 | ILMN_21091 | 1.66 |
| NM_012463.2 | ATP6V0A2 | ILMN_23163 | 1.66 |
| NM_001042734.1 | SEC24B | ILMN_174527 | 1.66 |
| NM_018698.3 | NXT2 | ILMN_168294 | 1.66 |
| NM_005433.3 | YES1 | ILMN_183786 | 1.66 |
| NM_015001.2 | SPEN | ILMN_180751 | 1.66 |
| NM_002076.2 | GNS | ILMN_177670 | 1.66 |
| NM_033412.1 | MCART1 | ILMN_22327 | 1.66 |
| NM_182970.2 | RIMS4 | ILMN_16896 | 1.65 |
| NM_001204.5 | BMPR2 | ILMN_29007 | 1.65 |
| NM_019024.1 | HEATR5B | ILMN_25274 | 1.65 |
| NM_001034996.1 | RPL14 | ILMN_2719 | 1.65 |
| NM_020836.2 | BEGAIN | ILMN_10503 | 1.65 |
| NM_001006657.1 | WDR35 | ILMN_175554 | 1.65 |
| NM_005637.2 | SS18 | ILMN_6637 | 1.65 |
| NM_173614.2 | NOMO2 | ILMN_1736 | 1.65 |
| NM_003836.4 | DLK1 | ILMN_13065 | 1.65 |
| NM_025189.2 | ZNF430 | ILMN_24543 | 1.65 |
| NM_006731.2 | FKTN | ILMN_6512 | 1.65 |
| NM_032239.2 | LARP2 | ILMN_9962 | 1.64 |
| NM_016265.3 | ZNF12 | ILMN_27628 | 1.64 |
| NM_023080.1 | C8orf33 | ILMN_15901 | 1.64 |
| NM_003129.3 | SQLE | ILMN_183123 | 1.64 |
| NM_014671.1 | UBE3C | ILMN_9296 | 1.64 |
| XM_934113.1 | LOC653489 | ILMN_42664 | 1.64 |
| NM_033109.2 | PNPT1 | ILMN_22316 | 1.64 |
| NM_020755.2 | SERINC1 | ILMN_24825 | 1.63 |
| NM_153350.2 | FBXL16 | ILMN_17900 | 1.63 |
| NM_000743.2 | CHRNA3 | ILMN_23268 | 1.63 |
| NM_080927.3 | DCBLD2 | ILMN_175741 | 1.63 |
| NM_003200.1 | TCF3 | ILMN_173421 | 1.63 |
| NM_012302.2 | LPHN2 | ILMN_11901 | 1.63 |
| NM_130809.2 | PRRC1 | ILMN_24905 | 1.62 |
| NM_006123.2 | IDS | ILMN_17605 | 1.62 |
| NM_018097.1 | CEP27 | ILMN_15131 | 1.62 |
| NM_020859.1 | SHRM | ILMN_16821 | 1.62 |
| NM_139353.1 | TAF1C | ILMN_4122 | 1.62 |
| NM_001039755.1 | FLJ44124 | ILMN_44450 | 1.62 |
| NM_005800.3 | USPL1 | ILMN_27577 | 1.61 |
| NM_012063.1 | DNM1L | ILMN_15529 | 1.61 |
| NM_006011.3 | ST8SIA2 | ILMN_19287 | 1.61 |
| NM_015878.4 | AZIN1 | ILMN_4825 | 1.61 |
| NM_138477.2 | CDAN1 | ILMN_168162 | 1.61 |
| NM_024561.3 | NARG1L | ILMN_22547 | 1.6 |
| NM_152415.1 | VPS37A | ILMN_12702 | 1.6 |
| NM_198925.1 | SEMA4B | ILMN_25258 | 1.6 |
| NM_175085.1 | GART | ILMN_19282 | 1.6 |
| NM_000108.3 | DLD | ILMN_168272 | 1.6 |
| NM_002129.2 | HMGB2 | ILMN_3200 | 1.6 |
| NM_001042442.1 | CAST | ILMN_163030 | 1.59 |
| NM_130473.1 | MADD | ILMN_12717 | 1.59 |
| NM_015902.4 | UBR5 | ILMN_178959 | 1.59 |
| NM_004428.2 | EFNA1 | ILMN_14320 | 1.59 |
| NM_001002257.1 | LYCAT | ILMN_26201 | 1.59 |
| NM_015348.1 | TMEM131 | ILMN_308809 | 1.59 |
| NM_001040101.1 | D4S234E | ILMN_173747 | 1.59 |
| NM_015079.2 | TBC1D2B | ILMN_14085 | 1.58 |
| NM_006918.4 | SC5DL | ILMN_24287 | 1.58 |
| NM_005808.2 | CTDSPL | ILMN_510 | 1.58 |
| NM_178831.4 | GATS | ILMN_18755 | 1.58 |
| NM_007257.4 | PNMA2 | ILMN_10930 | 1.58 |
| NM_000368.3 | TSC1 | ILMN_182048 | 1.57 |
| NM_031452.2 | FAM103A1 | ILMN_28104 | 1.57 |
| NM_198097.1 | C7orf28B | ILMN_8655 | 1.57 |
| NM_022497.3 | MRPS25 | ILMN_20500 | 1.56 |
| NM_002254.6 | KIF3C | ILMN_22353 | 1.56 |
| NM_023072.1 | ZSWIM4 | ILMN_167043 | 1.56 |
| NM_001013716.1 | LOC441087 | ILMN_19369 | 1.56 |
| NM_053274.2 | GLMN | ILMN_38827 | 1.55 |
| NM_002577.3 | PAK2 | ILMN_163942 | 1.55 |
| NM_177965.2 | C8orf37 | ILMN_11118 | 1.55 |
| NM_006054.2 | RTN3 | ILMN_20331 | 1.54 |
| NM_031469.2 | SH3BGRL2 | ILMN_9801 | 1.54 |
| NM_004671.2 | PIAS2 | ILMN_11308 | 1.53 |
| NM_005088.2 | SFRS17A | ILMN_26209 | 1.53 |
| NM_144599.3 | NIPA1 | ILMN_8853 | 1.53 |
| NM_020447.3 | C15orf17 | ILMN_13536 | 1.53 |
| NM_153812.1 | PHF13 | ILMN_27355 | 1.53 |
| NM_006631.2 | ZNF266 | ILMN_12055 | 1.52 |
| NM_054013.2 | MGAT4B | ILMN_14669 | 1.52 |
| NM_152641.2 | ARID2 | ILMN_163259 | 1.51 |
| NM_032290.2 | ANKRD32 | ILMN_15362 | 1.51 |
| NM_182569.1 | GDPD1 | ILMN_172818 | 1.51 |
| NM_017819.2 | RG9MTD1 | ILMN_26970 | 1.51 |
| NM_206907.3 | PRKAA1 | ILMN_180991 | 1.51 |
| NM_001253.2 | CDC5L | ILMN_15507 | 1.51 |
| NM_003418.1 | CNBP | ILMN_9092 | 1.51 |
| NM_152224.1 | PPEF1 | ILMN_24147 | -1.5 |
| NM_003368.4 | USP1 | ILMN_5285 | -1.5 |
| NM_020175.1 | DUS3L | ILMN_3805 | -1.5 |
| NM_003977.1 | AIP | ILMN_13720 | -1.5 |
| NM_001625.2 | AK2 | ILMN_1688 | -1.5 |
| NM_005441.2 | CHAF1B | ILMN_165317 | -1.51 |
| NM_138484.2 | SGOL1 | ILMN_14008 | -1.51 |
| NM_023936.1 | MRPS34 | ILMN_5723 | -1.51 |
| NM_020808.3 | SIPA1L2 | ILMN_167573 | -1.51 |
| NM_001018020.1 | TPM1 | ILMN_14091 | -1.51 |
| NM_181306.1 | MRPL52 | ILMN_16276 | -1.51 |
| NM_013300.1 | C12orf24 | ILMN_24807 | -1.51 |
| NM_138787.2 | C11orf74 | ILMN_16125 | -1.51 |
| NM_014884.2 | SFRS14 | ILMN_10407 | -1.51 |
| NM_000100.2 | CSTB | ILMN_26819 | -1.51 |
| NM_001067.2 | TOP2A | ILMN_19849 | -1.51 |
| NM_145117.3 | NAV2 | ILMN_8536 | -1.52 |
| NM_001008697.1 | TFIP11 | ILMN_5509 | -1.52 |
| NM_198527.2 | HDDC3 | ILMN_29602 | -1.52 |
| NM_015386.2 | COG4 | ILMN_28901 | -1.52 |
| NM_032512.2 | PDZD4 | ILMN_3986 | -1.52 |
| NM_181454.1 | MRPL55 | ILMN_26404 | -1.52 |
| NM_016401.2 | C11orf73 | ILMN_22672 | -1.52 |
| NM_080651.1 | MED30 | ILMN_7158 | -1.52 |
| NM_144716.2 | CCDC12 | ILMN_20382 | -1.52 |
| NM_017907.1 | C11orf59 | ILMN_10434 | -1.52 |
| NM_000117.1 | EMD | ILMN_13626 | -1.52 |
| NM_005184.2 | CALM3 | ILMN_3508 | -1.52 |
| NM_018718.1 | TSGA14 | ILMN_11000 | -1.53 |
| NM_052844.3 | WDR34 | ILMN_25161 | -1.53 |
| NM_182513.1 | SPC24 | ILMN_174688 | -1.53 |
| NM_170738.1 | MRPL11 | ILMN_4211 | -1.53 |
| NM_058219.2 | EXOSC6 | ILMN_15247 | -1.53 |
| XM_001125904.1 | LOC730455 | ILMN_172199 | -1.53 |
| NM_003504.3 | CDC45L | ILMN_25781 | -1.53 |
| NM_002818.2 | PSME2 | ILMN_19572 | -1.53 |
| NM_080678.1 | UBE2F | ILMN_781 | -1.53 |
| NM_032635.2 | TMEM147 | ILMN_13729 | -1.53 |
| NM_020675.3 | SPC25 | ILMN_915 | -1.54 |
| NM_007022.3 | CYB561D2 | ILMN_26535 | -1.54 |
| NM_203385.1 | RNH1 | ILMN_28630 | -1.54 |
| NM_015169.3 | RRS1 | ILMN_176178 | -1.54 |
| NM_152653.2 | UBE2E2 | ILMN_29196 | -1.54 |
| NM_007097.2 | CLTB | ILMN_19099 | -1.54 |
| NM_080597.2 | OSBPL1A | ILMN_10951 | -1.54 |
| NM_022343.2 | C9orf19 | ILMN_12810 | -1.54 |
| NM_004111.4 | FEN1 | ILMN_162686 | -1.54 |
| NM_001453.1 | FOXC1 | ILMN_23624 | -1.54 |
| NM_080668.2 | CDCA5 | ILMN_3057 | -1.54 |
| NM_004111.4 | FEN1 | ILMN_162686 | -1.54 |
| NM_145871.1 | GSTZ1 | ILMN_19421 | -1.55 |
| NM_000943.4 | PPIC | ILMN_19075 | -1.55 |
| NM_016625.2 | RSRC1 | ILMN_14978 | -1.55 |
| NM_014459.2 | PCDH17 | ILMN_5341 | -1.55 |
| NM_152912.3 | MTIF3 | ILMN_16655 | -1.55 |
| NM_007051.2 | FAF1 | ILMN_25532 | -1.55 |
| NM_005382.1 | NEFM | ILMN_1804 | -1.55 |
| NM_005804.2 | DDX39 | ILMN_19976 | -1.55 |
| NM_213596.1 | FOXN4 | ILMN_25618 | -1.56 |
| NM_012384.2 | GMEB2 | ILMN_7174 | -1.56 |
| NM_004885.1 | NPFFR2 | ILMN_20676 | -1.56 |
| XM_173119.5 | LOC255130 | ILMN_42241 | -1.56 |
| NM_032772.3 | ZNF503 | ILMN_2048 | -1.56 |
| NM_032814.2 | TMEM118 | ILMN_21714 | -1.56 |
| NM_006833.4 | COPS6 | ILMN_14890 | -1.56 |
| NM_198434.1 | AURKA | ILMN_12352 | -1.56 |
| NM_138720.1 | HIST1H2BD | ILMN_17622 | -1.56 |
| NM_015190.3 | DNAJC9 | ILMN_28456 | -1.56 |
| NM_014463.1 | LSM3 | ILMN_23516 | -1.56 |
| NM_007083.3 | NUDT6 | ILMN_903 | -1.57 |
| NM_014638.2 | PLCH2 | ILMN_5494 | -1.57 |
| NM_025136.1 | OPA3 | ILMN_11296 | -1.57 |
| NM_014223.2 | NFYC | ILMN_5936 | -1.57 |
| NM_019103.2 | ZMAT5 | ILMN_13941 | -1.57 |
| NM_014254.1 | TMEM5 | ILMN_26271 | -1.57 |
| NM_194272.1 | RBPMS2 | ILMN_18109 | -1.57 |
| NM_001002836.2 | ZNF787 | ILMN_24445 | -1.57 |
| NM_001011.3 | RPS7 | ILMN_10275 | -1.57 |
| NM_006233.4 | POLR2I | ILMN_17223 | -1.57 |
| NM_133173.2 | APBB3 | ILMN_14486 | -1.58 |
| NM_020310.2 | MNT | ILMN_21283 | -1.58 |
| NM_014007.2 | ZBTB43 | ILMN_17837 | -1.58 |
| NM_018066.3 | ATPBD1B | ILMN_37626 | -1.58 |
| NM_015997.2 | C1orf66 | ILMN_20031 | -1.58 |
| NM_001042426.1 | CENPA | ILMN_180589 | -1.58 |
| NM_014183.2 | DYNLRB1 | ILMN_6713 | -1.58 |
| NM_052988.2 | CDK10 | ILMN_15483 | -1.58 |
| NM_007155.4 | ZP3 | ILMN_17555 | -1.58 |
| NM_004542.2 | NDUFA3 | ILMN_6292 | -1.58 |
| NM_199483.1 | C20orf24 | ILMN_5274 | -1.58 |
| NM_001012271.1 | BIRC5 | ILMN_20327 | -1.59 |
| NM_004059.4 | CCBL1 | ILMN_22741 | -1.59 |
| NM_001080848.1 | CSAG3B | ILMN_167646 | -1.59 |
| NM_017917.2 | PPP2R3C | ILMN_23821 | -1.59 |
| NM_024103.2 | SLC25A23 | ILMN_172134 | -1.59 |
| NM_199250.1 | C19orf48 | ILMN_17977 | -1.59 |
| NM_004512.3 | IL11RA | ILMN_3536 | -1.59 |
| NM_021242.4 | MID1IP1 | ILMN_161908 | -1.6 |
| NM_033115.2 | MGC16169 | ILMN_16160 | -1.6 |
| NM_004982.2 | KCNJ8 | ILMN_29993 | -1.6 |
| NM_020967.2 | NCOA5 | ILMN_182789 | -1.6 |
| NM_006854.3 | KDELR2 | ILMN_1810 | -1.6 |
| NM_153026.1 | PRICKLE1 | ILMN_15149 | -1.6 |
| NM_014170.2 | GTPBP8 | ILMN_27163 | -1.61 |
| NM_080546.3 | SLC44A1 | ILMN_23525 | -1.61 |
| NM_000190.3 | HMBS | ILMN_16358 | -1.61 |
| NM_032638.3 | GATA2 | ILMN_20021 | -1.61 |
| NM_003362.2 | UNG | ILMN_21638 | -1.61 |
| NM_003107.2 | SOX4 | ILMN_17456 | -1.61 |
| NM_002156.4 | HSPD1 | ILMN_178202 | -1.62 |
| NM_153682.2 | PIGP | ILMN_18625 | -1.62 |
| NM_024122.2 | APOO | ILMN_11248 | -1.62 |
| NM_145701.1 | CDCA4 | ILMN_5601 | -1.62 |
| NM_005077.3 | TLE1 | ILMN_10669 | -1.62 |
| NM_058216.1 | RAD51C | ILMN_2944 | -1.62 |
| NM_000820.1 | GAS6 | ILMN_10723 | -1.62 |
| NM_001008709.1 | PPP1CA | ILMN_29100 | -1.62 |
| NM_001157.2 | ANXA11 | ILMN_27543 | -1.63 |
| NM_022903.3 | CCDC71 | ILMN_21600 | -1.63 |
| XM_939319.2 | R3HCC1 | ILMN_36974 | -1.63 |
| NM_018410.3 | HJURP | ILMN_29337 | -1.63 |
| NM_024065.3 | PDCL3 | ILMN_30010 | -1.63 |
| NM_001025249.1 | DUT | ILMN_7273 | -1.63 |
| NM_014279.4 | OLFM1 | ILMN_27341 | -1.63 |
| NM_173633.2 | TMEM145 | ILMN_171010 | -1.63 |
| NM_138458.2 | WDR92 | ILMN_37809 | -1.64 |
| NM_004987.3 | LIMS1 | ILMN_11207 | -1.64 |
| NM_176787.4 | PIGN | ILMN_163610 | -1.64 |
| XM_925818.1 | LOC642282 | ILMN_41968 | -1.64 |
| NM_024333.1 | FSD1 | ILMN_13664 | -1.64 |
| NM_001040668.1 | BCL2L12 | ILMN_177176 | -1.64 |
| NM_001018115.1 | FANCD2 | ILMN_11844 | -1.64 |
| NM_004365.2 | CETN3 | ILMN_25663 | -1.64 |
| NM_015983.2 | UBE2D4 | ILMN_18182 | -1.64 |
| NM_004470.2 | FKBP2 | ILMN_17464 | -1.64 |
| NM_172251.1 | MRPL54 | ILMN_1467 | -1.64 |
| NM_001077446.1 | TSEN34 | ILMN_177054 | -1.64 |
| NM_016553.3 | NUP62 | ILMN_17816 | -1.64 |
| NM_006234.4 | POLR2J | ILMN_4217 | -1.65 |
| NM_001025238.1 | TSPAN4 | ILMN_26489 | -1.65 |
| NM_004577.3 | PSPH | ILMN_14445 | -1.65 |
| NM_178439.3 | GMCL1 | ILMN_3285 | -1.65 |
| NM_000905.2 | NPY | ILMN_11990 | -1.65 |
| NM_001031827.1 | BOLA2 | ILMN_4509 | -1.65 |
| NM_021132.1 | PPP3CB | ILMN_13398 | -1.65 |
| NM_002493.3 | NDUFB6 | ILMN_9342 | -1.65 |
| NM_021210.3 | TRAPPC1 | ILMN_8184 | -1.65 |
| NM_031266.2 | HNRNPAB | ILMN_757 | -1.65 |
| NM_002804.4 | PSMC3 | ILMN_22724 | -1.65 |
| NM_212552.2 | BOLA3 | ILMN_28776 | -1.65 |
| NM_021134.2 | MRPL23 | ILMN_17506 | -1.65 |
| NM_006409.2 | ARPC1A | ILMN_14839 | -1.65 |
| NM_002676.1 | PMM1 | ILMN_17005 | -1.66 |
| XM_171158.5 | MGC27348 | ILMN_33625 | -1.66 |
| NM_005589.2 | ALDH6A1 | ILMN_24260 | -1.66 |
| NM_002157.1 | HSPE1 | ILMN_2612 | -1.66 |
| NM_033251.1 | RPL13 | ILMN_2271 | -1.67 |
| NM_016558.2 | SCAND1 | ILMN_23327 | -1.67 |
| NM_001003897.1 | MANBAL | ILMN_11747 | -1.67 |
| NM_021944.1 | C14orf93 | ILMN_11237 | -1.67 |
| NM_021254.1 | C21orf59 | ILMN_28603 | -1.67 |
| NM_031298.2 | TMEM93 | ILMN_9888 | -1.67 |
| NM_033402.3 | LRRCC1 | ILMN_15234 | -1.68 |
| NM_006214.3 | PHYH | ILMN_18235 | -1.68 |
| NM_198038.1 | NUDT9 | ILMN_12448 | -1.68 |
| NM_020412.3 | CHMP1B | ILMN_27331 | -1.68 |
| NM_013248.2 | NXT1 | ILMN_26865 | -1.68 |
| NM_003310.1 | TSSC1 | ILMN_20023 | -1.68 |
| NM_001040139.1 | CKLF | ILMN_162861 | -1.68 |
| NM_002957.3 | RXRA | ILMN_6758 | -1.69 |
| NM_001009608.1 | C20orf94 | ILMN_24801 | -1.69 |
| NM_002897.3 | RBMS1 | ILMN_18726 | -1.69 |
| XM_001129423.1 | LOC729137 | ILMN_166772 | -1.69 |
| NM_194260.1 | UBE2I | ILMN_27308 | -1.69 |
| NM_001112.2 | ADARB1 | ILMN_30004 | -1.7 |
| NM_022370.2 | ROBO3 | ILMN_24988 | -1.7 |
| NM_014498.2 | GOLPH4 | ILMN_179486 | -1.7 |
| NM_001006610.1 | SIAH1 | ILMN_9220 | -1.7 |
| NM_000414.1 | HSD17B4 | ILMN_23623 | -1.7 |
| NM_001017963.1 | HSP90AA1 | ILMN_16669 | -1.71 |
| NM_021244.3 | RRAGD | ILMN_5663 | -1.71 |
| NM_012241.2 | SIRT5 | ILMN_18454 | -1.71 |
| NM_023077.1 | C1orf163 | ILMN_14119 | -1.71 |
| NM_003133.1 | SRP9 | ILMN_137290 | -1.71 |
| NM_032527.3 | ZGPAT | ILMN_23696 | -1.71 |
| NM_199192.1 | BRE | ILMN_15533 | -1.71 |
| NM_007310.1 | COMT | ILMN_26337 | -1.71 |
| NM_024640.3 | YRDC | ILMN_2794 | -1.71 |
| NM_005326.4 | HAGH | ILMN_22401 | -1.71 |
| NM_018840.2 | C20orf24 | ILMN_10676 | -1.71 |
| NM_004701.2 | CCNB2 | ILMN_15254 | -1.71 |
| NM_018840.2 | C20orf24 | ILMN_10676 | -1.71 |
| NM_000288.1 | PEX7 | ILMN_25066 | -1.72 |
| NM_006567.3 | FARS2 | ILMN_19613 | -1.72 |
| XR_015731.1 | LOC729101 | ILMN_163644 | -1.72 |
| NM_138794.2 | LYPLAL1 | ILMN_25005 | -1.72 |
| NM_015463.1 | C2orf32 | ILMN_1437 | -1.72 |
| NM_032476.2 | MRPS6 | ILMN_17239 | -1.72 |
| NR_002326.1 | SNORA64 | ILMN_19397 | -1.73 |
| NM_153044.1 | FLJ35801 | ILMN_23944 | -1.73 |
| NM_001003796.1 | NHP2L1 | ILMN_17720 | -1.73 |
| NM_032343.1 | CHCHD6 | ILMN_19685 | -1.73 |
| NM_001333.2 | CTSL2 | ILMN_22377 | -1.73 |
| NM_032169.4 | ACAD11 | ILMN_25425 | -1.73 |
| NM_199250.1 | C19orf48 | ILMN_17977 | -1.73 |
| NM_020449.2 | THOC2 | ILMN_162047 | -1.73 |
| NM_000505.3 | F12 | ILMN_181878 | -1.73 |
| NM_005103.3 | FEZ1 | ILMN_28992 | -1.73 |
| NM_002413.3 | MGST2 | ILMN_8759 | -1.74 |
| NM_018465.2 | C9orf46 | ILMN_17839 | -1.74 |
| XM_942540.1 | SAPS2 | ILMN_138398 | -1.74 |
| NM_003730.3 | RNASET2 | ILMN_412 | -1.74 |
| NR_003105.1 | ZWILCH | ILMN_166966 | -1.74 |
| XM_001129232.1 | LOC731049 | ILMN_180228 | -1.74 |
| NM_001042401.1 | C21orf51 | ILMN_179828 | -1.75 |
| NM_152308.1 | C16orf75 | ILMN_5047 | -1.75 |
| NM_178439.3 | GMCL1 | ILMN_3285 | -1.75 |
| NM_203457.1 | PPIE | ILMN_13153 | -1.75 |
| NM_014736.4 | KIAA0101 | ILMN_2026 | -1.75 |
| NM_001042476.1 | CARHSP1 | ILMN_164906 | -1.75 |
| NM_002484.2 | NUBP1 | ILMN_23098 | -1.76 |
| NM_024540.2 | MRPL24 | ILMN_29128 | -1.76 |
| NM_152318.2 | C12orf45 | ILMN_25959 | -1.77 |
| NM_031902.3 | MRPS5 | ILMN_14369 | -1.77 |
| NM_001031713.2 | CCDC90A | ILMN_9159 | -1.77 |
| NM_019554.2 | S100A4 | ILMN_20183 | -1.77 |
| NM_000714.4 | TSPO | ILMN_12726 | -1.77 |
| NM_198948.1 | NUDT1 | ILMN_8489 | -1.77 |
| NM_005723.2 | TSPAN5 | ILMN_8032 | -1.78 |
| NM_080916.1 | DGUOK | ILMN_172755 | -1.78 |
| NM_002263.2 | KIFC1 | ILMN_8595 | -1.78 |
| NM_003916.3 | AP1S2 | ILMN_3812 | -1.78 |
| NM_000389.2 | CDKN1A | ILMN_16780 | -1.78 |
| NM_024578.1 | OCEL1 | ILMN_17644 | -1.79 |
| NM_014170.2 | GTPBP8 | ILMN_27163 | -1.79 |
| NM_198391.1 | FLRT3 | ILMN_23273 | -1.79 |
| NM_032361.1 | THOC3 | ILMN_17969 | -1.79 |
| NM_013299.3 | SAC3D1 | ILMN_9385 | -1.79 |
| NM_001168.2 | BIRC5 | ILMN_20443 | -1.79 |
| NM_020155.2 | GPR137 | ILMN_24699 | -1.79 |
| NM_014026.3 | DCPS | ILMN_24626 | -1.8 |
| NM_012446.2 | SSBP2 | ILMN_5320 | -1.8 |
| NM_002613.3 | PDPK1 | ILMN_27765 | -1.8 |
| NM_001080422.1 | FAM108A3 | ILMN_174864 | -1.8 |
| NM_006427.2 | SIVA | ILMN_26581 | -1.8 |
| NM_001634.4 | AMD1 | ILMN_21529 | -1.8 |
| NM_024684.2 | C11orf67 | ILMN_26165 | -1.8 |
| NM_016098.1 | BRP44L | ILMN_4349 | -1.8 |
| NM_012485.1 | HMMR | ILMN_16900 | -1.8 |
| NM_177983.1 | PPM1G | ILMN_878 | -1.81 |
| NM_003053.1 | SLC18A1 | ILMN_23324 | -1.81 |
| NM_173517.3 | VKORC1L1 | ILMN_9384 | -1.81 |
| NM_002712.1 | PPP1R7 | ILMN_29559 | -1.81 |
| NM_022135.2 | POPDC2 | ILMN_17743 | -1.81 |
| NM_024955.4 | FOXRED2 | ILMN_165686 | -1.81 |
| XM_001128002.1 | LOC728153 | ILMN_168116 | -1.82 |
| NM_004615.2 | TSPAN7 | ILMN_20684 | -1.82 |
| NM_018466.3 | ALG13 | ILMN_3125 | -1.82 |
| NM_016118.3 | NUB1 | ILMN_23529 | -1.82 |
| NM_012321.2 | LSM4 | ILMN_5332 | -1.82 |
| XM_928013.1 | ACBD7 | ILMN_138784 | -1.83 |
| NM_024333.1 | FSD1 | ILMN_13664 | -1.83 |
| NM_004649.5 | C21orf33 | ILMN_28752 | -1.83 |
| NM_001444.1 | FABP5 | ILMN_27564 | -1.83 |
| NM_020470.1 | YIF1A | ILMN_29299 | -1.83 |
| XM_933956.1 | LOC644162 | ILMN_43225 | -1.83 |
| NM_032111.2 | MRPL14 | ILMN_15720 | -1.83 |
| NM_199069.1 | C3orf60 | ILMN_30348 | -1.83 |
| NM_153213.3 | ARHGEF19 | ILMN_20302 | -1.84 |
| NM_138720.1 | HIST1H2BD | ILMN_17622 | -1.84 |
| NM_053050.2 | MRPL53 | ILMN_25576 | -1.84 |
| NM_032361.1 | THOC3 | ILMN_17969 | -1.84 |
| NM_032334.1 | C8orf53 | ILMN_24637 | -1.85 |
| NM_016183.3 | MRTO4 | ILMN_1930 | -1.85 |
| NM_016126.1 | C1orf41 | ILMN_4683 | -1.85 |
| NM_016319.1 | COPS7A | ILMN_13902 | -1.85 |
| NM_001002018.1 | HCFC1R1 | ILMN_19015 | -1.85 |
| NM_016208.2 | VPS28 | ILMN_14412 | -1.86 |
| NM_001040409.1 | MTHFD2 | ILMN_167475 | -1.86 |
| NM_030771.1 | CCDC34 | ILMN_2645 | -1.86 |
| NM_033405.2 | PRIC285 | ILMN_10778 | -1.87 |
| NM_181702.1 | GEM | ILMN_16170 | -1.87 |
| NM_000156.4 | GAMT | ILMN_20028 | -1.87 |
| NM_020195.1 | C14orf124 | ILMN_4144 | -1.88 |
| NM_032907.3 | UBL7 | ILMN_17890 | -1.88 |
| NM_005749.2 | TOB1 | ILMN_13735 | -1.88 |
| NM_203456.1 | PPIE | ILMN_13734 | -1.88 |
| NM_145697.1 | CDCA1 | ILMN_17725 | -1.89 |
| NM_017425.2 | SPA17 | ILMN_9648 | -1.89 |
| NM_001620.1 | AHNAK | ILMN_28759 | -1.89 |
| NM_153768.1 | CABYR | ILMN_9439 | -1.89 |
| NM_022745.3 | ATPAF1 | ILMN_175478 | -1.89 |
| NM_005654.4 | NR2F1 | ILMN_177945 | -1.89 |
| NM_000101.2 | CYBA | ILMN_6945 | -1.89 |
| NM_001640.3 | APEH | ILMN_27694 | -1.89 |
| NM_002566.4 | P2RY11 | ILMN_12237 | -1.9 |
| XM_932890.2 | LOC388588 | ILMN_33026 | -1.9 |
| NM_004544.2 | NDUFA10 | ILMN_7463 | -1.9 |
| NM_002923.1 | RGS2 | ILMN_26119 | -1.9 |
| NM_015523.2 | REXO2 | ILMN_15016 | -1.9 |
| NM_001031684.1 | SFRS7 | ILMN_7620 | -1.9 |
| NM_148178.1 | C9orf23 | ILMN_3926 | -1.91 |
| NM_002028.3 | FNTB | ILMN_171968 | -1.91 |
| NM_017887.1 | C1orf123 | ILMN_28661 | -1.91 |
| NM_000485.2 | APRT | ILMN_4221 | -1.91 |
| NM_012215.2 | MGEA5 | ILMN_11399 | -1.91 |
| NM_001035505.1 | BOLA3 | ILMN_29223 | -1.91 |
| NM_000302.2 | PLOD1 | ILMN_20559 | -1.91 |
| NM_001398.2 | ECH1 | ILMN_28288 | -1.91 |
| NM_005793.3 | NME6 | ILMN_17980 | -1.92 |
| NM_020851.1 | ISLR2 | ILMN_19345 | -1.92 |
| NM_016050.2 | MRPL11 | ILMN_1149 | -1.92 |
| NM_014176.2 | UBE2T | ILMN_9573 | -1.92 |
| NM_014188.2 | SSU72 | ILMN_29116 | -1.92 |
| NM_002763.3 | PROX1 | ILMN_177185 | -1.92 |
| NM_001099660.1 | LRRN3 | ILMN_306943 | -1.92 |
| NM_024900.3 | PHF17 | ILMN_1535 | -1.93 |
| NM_016286.2 | DCXR | ILMN_17437 | -1.93 |
| NM_052969.1 | RPL39L | ILMN_26587 | -1.93 |
| NM_004891.2 | MRPL33 | ILMN_12897 | -1.93 |
| NM_001540.2 | HSPB1 | ILMN_28967 | -1.93 |
| NM_013406.1 | DHPS | ILMN_17692 | -1.94 |
| NM_005412.4 | SHMT2 | ILMN_19915 | -1.94 |
| XR_018614.1 | LOC391811 | ILMN_164838 | -1.94 |
| NM_005627.2 | SGK | ILMN_2451 | -1.95 |
| NM_198047.1 | HIBCH | ILMN_24888 | -1.95 |
| NM_030795.2 | STMN4 | ILMN_162349 | -1.95 |
| NM_001382.2 | DPAGT1 | ILMN_10306 | -1.95 |
| NM_015980.3 | HMP19 | ILMN_1495 | -1.95 |
| NM_017823.3 | DUSP23 | ILMN_6272 | -1.95 |
| NM_176805.1 | MRPS11 | ILMN_19324 | -1.96 |
| NM_024336.1 | IRX3 | ILMN_4354 | -1.96 |
| NM_032477.1 | MRPL41 | ILMN_12476 | -1.96 |
| NM_152773.2 | TCTEX1D2 | ILMN_19950 | -1.97 |
| XM_936240.1 | LOC653884 | ILMN_34094 | -1.97 |
| XM_930694.1 | LOC642477 | ILMN_36253 | -1.97 |
| NM_015426.2 | WDR51A | ILMN_23579 | -1.98 |
| NM_153682.2 | PIGP | ILMN_18625 | -1.98 |
| NM_012111.1 | AHSA1 | ILMN_11051 | -1.98 |
| NM_003860.2 | BANF1 | ILMN_13154 | -1.99 |
| NM_174893.1 | C17orf49 | ILMN_6984 | -1.99 |
| NM_012474.3 | UCK2 | ILMN_23283 | -1.99 |
| NM_181800.1 | UBE2C | ILMN_25999 | -1.99 |
| NM_024292.2 | UBL5 | ILMN_14261 | -2 |
| NM_001042426.1 | CENPA | ILMN_180589 | -2.01 |
| NM_003365.2 | UQCRC1 | ILMN_20071 | -2.01 |
| NM_017843.3 | BCAS4 | ILMN_21706 | -2.02 |
| NM_001979.4 | EPHX2 | ILMN_179814 | -2.02 |
| NM_013262.3 | MYLIP | ILMN_178445 | -2.02 |
| NM_020158.3 | EXOSC5 | ILMN_6934 | -2.02 |
| NM_007100.2 | ATP5I | ILMN_14284 | -2.02 |
| XM_941665.2 | LOC387763 | ILMN_43061 | -2.02 |
| NM_012177.2 | FBXO5 | ILMN_9763 | -2.03 |
| NM_001018109.1 | PIR | ILMN_13999 | -2.03 |
| NM_199184.1 | C6orf108 | ILMN_5240 | -2.03 |
| NM_138501.4 | GPSN2 | ILMN_6454 | -2.03 |
| NM_181876.2 | PPP2R2C | ILMN_15268 | -2.04 |
| NR_002797.1 | LOC255783 | ILMN_13825 | -2.04 |
| NM_017528.2 | WBSCR22 | ILMN_26330 | -2.04 |
| NM_005165.2 | ALDOC | ILMN_15767 | -2.04 |
| NM_001037633.1 | SIL1 | ILMN_10838 | -2.05 |
| NM_197956.1 | C9orf90 | ILMN_16848 | -2.05 |
| NM_003776.2 | MRPL40 | ILMN_21771 | -2.05 |
| NM_002523.1 | NPTX2 | ILMN_22638 | -2.05 |
| NM_001930.2 | DHPS | ILMN_3527 | -2.05 |
| NM_001010897.1 | SERP2 | ILMN_13346 | -2.06 |
| NM_016069.8 | Magmas | ILMN_21729 | -2.06 |
| NM_138401.2 | FAM125A | ILMN_16311 | -2.06 |
| NM_016098.1 | BRP44L | ILMN_4349 | -2.07 |
| NM_000465.1 | BARD1 | ILMN_1301 | -2.07 |
| NM_000856.3 | GUCY1A3 | ILMN_11680 | -2.08 |
| NM_021004.2 | DHRS4 | ILMN_10152 | -2.08 |
| NM_006963.3 | ZNF22 | ILMN_165495 | -2.08 |
| NM_001826.1 | CKS1B | ILMN_11313 | -2.08 |
| NM_080653.3 | ATP6V1E2 | ILMN_5551 | -2.09 |
| NM_001035513.1 | SDHC | ILMN_14364 | -2.09 |
| NM_006717.2 | SPIN1 | ILMN_23742 | -2.09 |
| NM_199191.1 | BRE | ILMN_15296 | -2.09 |
| NM_001031703.2 | TMEM103 | ILMN_40105 | -2.09 |
| NM_004615.2 | TSPAN7 | ILMN_20684 | -2.1 |
| NM_025263.2 | PRR3 | ILMN_21022 | -2.1 |
| NM_022770.2 | GINS3 | ILMN_7033 | -2.1 |
| NM_014241.3 | PTPLA | ILMN_24983 | -2.1 |
| NM_016095.1 | GINS2 | ILMN_26854 | -2.1 |
| NM_001002246.1 | ANAPC11 | ILMN_5565 | -2.1 |
| NM_001040668.1 | BCL2L12 | ILMN_177176 | -2.11 |
| NM_005552.4 | KLC1 | ILMN_22293 | -2.11 |
| NM_020529.1 | NFKBIA | ILMN_6745 | -2.11 |
| NM_004378.1 | CRABP1 | ILMN_12739 | -2.12 |
| NM_018837.2 | SULF2 | ILMN_18271 | -2.13 |
| NM_016390.2 | C9orf114 | ILMN_20184 | -2.13 |
| NM_006834.2 | RAB32 | ILMN_784 | -2.13 |
| NM_006182.2 | DDR2 | ILMN_20698 | -2.13 |
| NM_021952.2 | ELAVL4 | ILMN_17338 | -2.13 |
| NM_180703.1 | U1SNRNPBP | ILMN_13584 | -2.14 |
| NM_016374.5 | ARID4B | ILMN_162934 | -2.14 |
| NM_016108.2 | AIG1 | ILMN_22004 | -2.15 |
| NM_181803.1 | UBE2C | ILMN_6398 | -2.15 |
| NM_017996.2 | DET1 | ILMN_164954 | -2.16 |
| NM_005238.2 | ETS1 | ILMN_173009 | -2.16 |
| NM_180981.1 | MRPL52 | ILMN_3474 | -2.16 |
| NM_003916.3 | AP1S2 | ILMN_3812 | -2.16 |
| NM_145274.2 | TMEM99 | ILMN_25105 | -2.17 |
| NM_001024218.1 | GPHN | ILMN_29329 | -2.17 |
| NM_001025235.1 | TSPAN4 | ILMN_9326 | -2.17 |
| NM_005946.2 | MT1A | ILMN_1797 | -2.17 |
| NM_201443.1 | TEAD4 | ILMN_21735 | -2.18 |
| NM_016937.2 | POLA1 | ILMN_181974 | -2.18 |
| NM_017489.1 | TERF1 | ILMN_164297 | -2.19 |
| NM_012342.2 | BAMBI | ILMN_8469 | -2.19 |
| NM_205843.1 | NFIC | ILMN_22629 | -2.2 |
| NM_199287.2 | CCDC137 | ILMN_309720 | -2.2 |
| NM_018304.2 | PRR11 | ILMN_32619 | -2.2 |
| NM_003258.2 | TK1 | ILMN_30154 | -2.2 |
| NM_002598.2 | PDCD2 | ILMN_5469 | -2.2 |
| NM_012234.4 | RYBP | ILMN_13259 | -2.2 |
| NM_014865.2 | NCAPD2 | ILMN_26621 | -2.2 |
| NM_006556.3 | PMVK | ILMN_165582 | -2.21 |
| NM_003404.3 | YWHAB | ILMN_17127 | -2.21 |
| NM_006324.2 | CFDP1 | ILMN_23508 | -2.21 |
| NM_018663.1 | PXMP2 | ILMN_19172 | -2.21 |
| NM_058246.3 | DNAJB6 | ILMN_7651 | -2.21 |
| NM_001079863.1 | DBI | ILMN_173500 | -2.21 |
| NM_006158.2 | NEFL | ILMN_22054 | -2.22 |
| NM_152515.2 | CKAP2L | ILMN_28483 | -2.22 |
| NM_001878.2 | CRABP2 | ILMN_16252 | -2.22 |
| NM_138701.1 | C7orf11 | ILMN_20229 | -2.22 |
| NM_145806.2 | ZNF511 | ILMN_15566 | -2.22 |
| NM_005056.1 | JARID1A | ILMN_12150 | -2.22 |
| XM_934985.1 | LOC400879 | ILMN_31001 | -2.22 |
| NM_022906.2 | STAG3L4 | ILMN_307336 | -2.23 |
| NM_001495.4 | GFRA2 | ILMN_24176 | -2.23 |
| NM_003186.3 | TAGLN | ILMN_2335 | -2.23 |
| NM_004456.3 | EZH2 | ILMN_25740 | -2.23 |
| NM_001048172.1 | MUTYH | ILMN_164733 | -2.23 |
| NM_001326.2 | CSTF3 | ILMN_27551 | -2.23 |
| NM_004257.3 | TGFBRAP1 | ILMN_30176 | -2.24 |
| NM_007155.4 | ZP3 | ILMN_17555 | -2.24 |
| NM_004853.1 | STX8 | ILMN_9761 | -2.24 |
| NM_001017392.2 | SFRS14 | ILMN_17110 | -2.24 |
| NM_001827.1 | CKS2 | ILMN_14702 | -2.24 |
| NM_014142.2 | NUDT5 | ILMN_1656 | -2.25 |
| NM_198897.1 | FIBP | ILMN_8196 | -2.26 |
| NM_000820.1 | GAS6 | ILMN_10723 | -2.26 |
| NM_001889.2 | CRYZ | ILMN_30248 | -2.26 |
| XM_944321.1 | LOC402560 | ILMN_42108 | -2.27 |
| NM_001959.3 | EEF1B2 | ILMN_6380 | -2.27 |
| NM_021078.2 | GCN5L2 | ILMN_24446 | -2.27 |
| XM_934410.1 | LOC643995 | ILMN_31166 | -2.28 |
| NM_016644.1 | PRR16 | ILMN_4368 | -2.28 |
| NM_003211.3 | TDG | ILMN_29212 | -2.28 |
| NM_000154.1 | GALK1 | ILMN_18040 | -2.28 |
| NM_198486.2 | RPL7L1 | ILMN_9155 | -2.28 |
| NM_133465.2 | KIAA1958 | ILMN_17353 | -2.29 |
| NM_198954.1 | NUDT1 | ILMN_2361 | -2.29 |
| NM_005318.2 | H1F0 | ILMN_139403 | -2.29 |
| NM_022549.2 | FEZ1 | ILMN_419 | -2.3 |
| NM_000051.3 | ATM | ILMN_162851 | -2.3 |
| NM_138418.2 | C16orf14 | ILMN_9509 | -2.31 |
| NM_002338.2 | LSAMP | ILMN_861 | -2.31 |
| NM_020242.1 | KIF15 | ILMN_6188 | -2.32 |
| NM_005192.2 | CDKN3 | ILMN_4098 | -2.33 |
| NM_000476.1 | AK1 | ILMN_16785 | -2.34 |
| NM_002315.1 | LMO1 | ILMN_29972 | -2.35 |
| NM_033301.1 | RPL8 | ILMN_29819 | -2.35 |
| NM_003609.2 | HIRIP3 | ILMN_5112 | -2.36 |
| NM_000992.2 | RPL29 | ILMN_10772 | -2.37 |
| NM_000421.2 | KRT10 | ILMN_179036 | -2.37 |
| NM_032340.2 | C6orf125 | ILMN_21424 | -2.37 |
| NM_001914.2 | CYB5A | ILMN_25182 | -2.38 |
| NM_001123.2 | ADK | ILMN_4107 | -2.38 |
| NM_000485.2 | APRT | ILMN_4221 | -2.38 |
| NM_012227.1 | GTPBP6 | ILMN_9441 | -2.38 |
| NM_057159.2 | LPAR1 | ILMN_28278 | -2.39 |
| NM_001667.2 | ARL2 | ILMN_20438 | -2.39 |
| NM_054016.1 | FUSIP1 | ILMN_30145 | -2.4 |
| NM_004435.2 | ENDOG | ILMN_26482 | -2.4 |
| NM_021971.1 | GMPPB | ILMN_3929 | -2.41 |
| NM_199235.1 | COLEC11 | ILMN_6793 | -2.41 |
| NM_052969.1 | RPL39L | ILMN_26587 | -2.42 |
| NM_024482.1 | GMEB1 | ILMN_20915 | -2.43 |
| NM_138809.3 | CMBL | ILMN_1485 | -2.43 |
| NM_001014438.1 | CARS | ILMN_172747 | -2.44 |
| NM_012083.2 | FRAT2 | ILMN_164196 | -2.45 |
| NM_003707.1 | RUVBL1 | ILMN_16596 | -2.46 |
| NM_002801.2 | PSMB10 | ILMN_29653 | -2.46 |
| NM_001037675.1 | NBPF20 | ILMN_26956 | -2.46 |
| NM_015609.2 | C1orf144 | ILMN_5836 | -2.47 |
| NM_001034841.2 | LOC162073 | ILMN_3559 | -2.47 |
| NM_002487.2 | NDN | ILMN_23775 | -2.48 |
| NM_001040138.1 | CKLF | ILMN_162781 | -2.48 |
| XM_926249.2 | LOC642852 | ILMN_40586 | -2.49 |
| NM_003805.3 | CRADD | ILMN_12877 | -2.49 |
| XM_938497.2 | C6orf52 | ILMN_42173 | -2.5 |
| NM_153824.1 | PYCR1 | ILMN_8761 | -2.51 |
| NM_078629.1 | MSL3L1 | ILMN_29354 | -2.51 |
| NM_001018109.1 | PIR | ILMN_13999 | -2.51 |
| NM_004148.3 | NINJ1 | ILMN_21540 | -2.52 |
| NM_006515.1 | SETMAR | ILMN_17510 | -2.52 |
| NM_018663.1 | PXMP2 | ILMN_19172 | -2.52 |
| NM_005824.1 | LRRC17 | ILMN_162504 | -2.53 |
| NM_000992.2 | RPL29 | ILMN_10772 | -2.54 |
| NM_032747.2 | USMG5 | ILMN_10409 | -2.55 |
| NM_000022.2 | ADA | ILMN_8067 | -2.56 |
| NM_001031717.2 | CRELD1 | ILMN_14216 | -2.56 |
| NM_006860.2 | RABL4 | ILMN_4559 | -2.57 |
| NM_006014.3 | LAGE3 | ILMN_1071 | -2.57 |
| NM_017970.2 | C14orf102 | ILMN_22111 | -2.58 |
| NM_016086.2 | STYXL1 | ILMN_5068 | -2.59 |
| NM_017518.5 | UCHL5IP | ILMN_27285 | -2.59 |
| NM_138431.1 | MFSD3 | ILMN_22771 | -2.59 |
| NM_199071.2 | C21orf58 | ILMN_27743 | -2.62 |
| NM_003053.1 | SLC18A1 | ILMN_23324 | -2.62 |
| NM_001040167.1 | LFNG | ILMN_163755 | -2.63 |
| NM_019071.2 | ING3 | ILMN_177083 | -2.63 |
| XM_936103.1 | LOC642033 | ILMN_33652 | -2.64 |
| NM_001914.2 | CYB5A | ILMN_25182 | -2.64 |
| NM_025129.3 | FUZ | ILMN_24173 | -2.65 |
| NM_005327.2 | HADH | ILMN_13258 | -2.65 |
| NM_030919.2 | FAM83D | ILMN_16948 | -2.66 |
| NM_002014.2 | FKBP4 | ILMN_9429 | -2.67 |
| NM_001040142.1 | SCN2A | ILMN_167124 | -2.68 |
| NM_006703.2 | NUDT3 | ILMN_25244 | -2.68 |
| NM_005331.3 | HBQ1 | ILMN_24244 | -2.69 |
| NM_003656.3 | CAMK1 | ILMN_21373 | -2.7 |
| NM_024296.3 | CCDC28B | ILMN_26263 | -2.7 |
| NM_138765.2 | BAX | ILMN_11763 | -2.7 |
| NM_001002246.1 | ANAPC11 | ILMN_5565 | -2.71 |
| NM_006623.2 | PHGDH | ILMN_5800 | -2.71 |
| NM_017802.2 | HEATR2 | ILMN_1114 | -2.73 |
| NR_001445.1 | RN7SK | ILMN_14457 | -2.74 |
| NR_001449.1 | TRK1 | ILMN_6493 | -2.75 |
| NM_004316.2 | ASCL1 | ILMN_23892 | -2.75 |
| NM_000156.4 | GAMT | ILMN_20028 | -2.76 |
| NM_022893.2 | BCL11A | ILMN_17359 | -2.77 |
| NM_022743.1 | SMYD3 | ILMN_29453 | -2.77 |
| NM_006914.3 | RORB | ILMN_7297 | -2.77 |
| NM_020749.3 | MTUS1 | ILMN_4658 | -2.77 |
| NM_003542.3 | HIST1H4C | ILMN_30043 | -2.77 |
| NM_002093.2 | GSK3B | ILMN_7421 | -2.78 |
| NM_024011.2 | CDC2L2 | ILMN_20434 | -2.82 |
| NM_174942.1 | GAS2L3 | ILMN_5609 | -2.83 |
| NM_030928.2 | CDT1 | ILMN_18895 | -2.85 |
| NM_018983.3 | NOLA1 | ILMN_14204 | -2.85 |
| NM_205847.1 | GMPPA | ILMN_23338 | -2.85 |
| NM_005952.2 | MT1X | ILMN_16629 | -2.85 |
| NM_145080.3 | NSMCE1 | ILMN_27090 | -2.86 |
| NM_138493.2 | C6orf129 | ILMN_24513 | -2.87 |
| NM_001124.1 | ADM | ILMN_29514 | -2.88 |
| XM_926112.2 | LOC441155 | ILMN_37470 | -2.89 |
| NM_018473.2 | THEM2 | ILMN_27212 | -2.89 |
| XR_019449.1 | LOC644422 | ILMN_166674 | -2.91 |
| NM_003689.2 | AKR7A2 | ILMN_182370 | -2.92 |
| NM_002247.2 | KCNMA1 | ILMN_24236 | -2.93 |
| NM_005953.2 | MT2A | ILMN_11198 | -2.97 |
| NM_207376.1 | LOC387882 | ILMN_23241 | -3.03 |
| XM_001131304.1 | LOC728635 | ILMN_168315 | -3.04 |
| NM_001545.1 | ICT1 | ILMN_11458 | -3.04 |
| NM_000598.4 | IGFBP3 | ILMN_28010 | -3.06 |
| XM_001126211.1 | LOC727761 | ILMN_162963 | -3.09 |
| NM_033258.1 | GNG8 | ILMN_25463 | -3.1 |
| NM_019116.2 | UBFD1 | ILMN_179383 | -3.12 |
| NM_001382.2 | DPAGT1 | ILMN_10306 | -3.2 |
| NM_004175.3 | SNRPD3 | ILMN_163179 | -3.2 |
| NM_001002876.1 | CENPM | ILMN_12351 | -3.21 |
| NM_006265.1 | RAD21 | ILMN_171453 | -3.22 |
| NM_014169.2 | CHMP4A | ILMN_19959 | -3.24 |
| NM_017843.3 | BCAS4 | ILMN_21706 | -3.27 |
| NM_005694.1 | COX17 | ILMN_19252 | -3.29 |
| NM_021170.2 | HES4 | ILMN_18566 | -3.3 |
| NM_138444.3 | KCTD12 | ILMN_18501 | -3.4 |
| XM_935818.1 | FLJ20397 | ILMN_137080 | -3.48 |
| NM_001025248.1 | DUT | ILMN_163345 | -3.52 |
| NM_001500.2 | GMDS | ILMN_16535 | -3.53 |
| NM_024766.2 | C2orf34 | ILMN_14025 | -3.54 |
| NM_182533.1 | C1orf86 | ILMN_2880 | -3.54 |
| NM_006366.2 | CAP2 | ILMN_27367 | -3.7 |
| NM_007280.1 | OIP5 | ILMN_18200 | -3.74 |
| NM_133505.2 | DCN | ILMN_29913 | -3.78 |
| NM_000599.2 | IGFBP5 | ILMN_168089 | -3.94 |
| XM_374020.4 | LOC375295 | ILMN_45377 | -4.22 |
| NM_002166.4 | ID2 | ILMN_28481 | -5.12 |
| NM_002167.2 | ID3 | ILMN_6829 | -5.17 |
| NM_000599.2 | IGFBP5 | ILMN_168089 | -5.27 |
| NM_002166.4 | ID2 | ILMN_28481 | -7.23 |
