# Supplementary material for: Minimal Peroxide Exposure of Neuronal Cells Induces Multifaceted Adaptive Responses
Source: PLoS One. 2010 Dec 17;5(12):e14352. doi: 10.1371/journal.pone.0014352 (PMC3003681; doi:10.1371/journal.pone.0014352)
Supplement: Table S12 — BDNF-significantly regulated genes after 2 hours of stimulation in the CMP state SH-SY5Y cells. Each significantly regulated gene is described via its accession number (ACCESSION), Gene Symbol (SYMBOL), Illumina array transcript designation (TRANSCRIPT). For each gene the z-ratio of expression compared to untreated cells after 2 hours of ligand stimulation is displayed (CMP BDNF 8). (0.94 MB DOC) [file pone.0014352.s019.doc]

**Table S12. BDNF-significantly regulated genes after 2 hours of stimulation in the CMP state SH-SY5Y cells**. Each significantly regulated gene is described via its accession number (ACCESSION), Gene Symbol (SYMBOL), Illumina array transcript designation (TRANSCRIPT). For each gene the z-ratio of expression compared to un-treated cells after 2 hours of ligand stimulation is displayed (CMP BDNF 8).

| **ACCESSION** | **SYMBOL** | **TRANSCRIPT** | **CMP BDNF 2** |
| --- | --- | --- | --- |
| NM_001964.2 | EGR1 | ILMN_20932 | 12.36 |
| NM_001008219.1 | AMY1C | ILMN_28222 | 4.8 |
| NM_005324.3 | H3F3B | ILMN_26885 | 4.21 |
| NM_001300.4 | KLF6 | ILMN_17961 | 4.09 |
| XM_937850.1 | LOC285176 | ILMN_43277 | 3.79 |
| XM_942424.2 | LOC440589 | ILMN_39348 | 3.68 |
| NM_001008490.1 | KLF6 | ILMN_12381 | 3.59 |
| XM_944439.2 | LOC653994 | ILMN_38572 | 3.58 |
| NM_014817.3 | KIAA0644 | ILMN_164846 | 3.56 |
| NM_003110.4 | SP2 | ILMN_7882 | 3.52 |
| XM_941665.2 | LOC387763 | ILMN_43061 | 3.48 |
| NM_012215.2 | MGEA5 | ILMN_11399 | 3.42 |
| NM_001033506.1 | CSTF3 | ILMN_27049 | 3.41 |
| NM_004417.2 | DUSP1 | ILMN_20700 | 3.38 |
| XM_926231.1 | P704P | ILMN_36679 | 3.19 |
| NM_001005474.1 | NFKBIZ | ILMN_16362 | 3.13 |
| NM_002228.3 | JUN | ILMN_7746 | 3.13 |
| NM_006925.3 | SFRS5 | ILMN_34497 | 3.11 |
| XM_938988.1 | LOC402221 | ILMN_35678 | 3.04 |
| NM_001040456.1 | RHBDD2 | ILMN_168345 | 3.04 |
| NM_006275.4 | SFRS6 | ILMN_24964 | 3.03 |
| NM_015306.1 | USP24 | ILMN_309418 | 3.02 |
| NM_182776.1 | MCM7 | ILMN_1133 | 2.99 |
| NM_018697.3 | LANCL2 | ILMN_920 | 2.97 |
| XM_001129527.1 | KLF11 | ILMN_168976 | 2.94 |
| NM_001017421.1 | FKSG30 | ILMN_2393 | 2.93 |
| NM_020801.1 | ARRDC3 | ILMN_22538 | 2.91 |
| NM_002566.4 | P2RY11 | ILMN_12237 | 2.91 |
| NM_002874.3 | RAD23B | ILMN_19346 | 2.84 |
| NM_006925.3 | SFRS5 | ILMN_34497 | 2.81 |
| NM_001040456.1 | RHBDD2 | ILMN_168345 | 2.8 |
| NR_000011.1 | SNORA70 | ILMN_7210 | 2.73 |
| NM_148957.2 | TNFRSF19 | ILMN_28684 | 2.72 |
| NM_152322.2 | BTBD11 | ILMN_506 | 2.7 |
| NM_002673.3 | PLXNB1 | ILMN_22628 | 2.6 |
| NM_001080453.1 | INTS1 | ILMN_173681 | 2.52 |
| NM_013243.2 | SCG3 | ILMN_174345 | 2.51 |
| NM_018708.2 | FEM1A | ILMN_2838 | 2.48 |
| XM_940278.1 | LOC651149 | ILMN_44210 | 2.47 |
| NM_024909.1 | C6orf134 | ILMN_21139 | 2.4 |
| NM_002213.3 | ITGB5 | ILMN_24189 | 2.38 |
| XM_926036.1 | LOC653103 | ILMN_32029 | 2.38 |
| NM_016028.4 | SUV420H1 | ILMN_29861 | 2.37 |
| NM_020724.1 | RNF150 | ILMN_26801 | 2.37 |
| NR_002809.1 | LOC338799 | ILMN_15606 | 2.35 |
| NM_203499.1 | DDX42 | ILMN_1880 | 2.35 |
| NM_000787.3 | DBH | ILMN_25962 | 2.34 |
| XM_001134215.1 | PDPR | ILMN_162295 | 2.33 |
| NM_003131.2 | SRF | ILMN_22299 | 2.33 |
| NM_001008408.3 | RBM33 | ILMN_165407 | 2.33 |
| NM_001005753.1 | VPS24 | ILMN_29671 | 2.32 |
| NM_144582.2 | TEX261 | ILMN_27405 | 2.31 |
| NM_014330.2 | PPP1R15A | ILMN_1024 | 2.3 |
| NM_199043.1 | C14orf102 | ILMN_22442 | 2.29 |
| NM_138477.2 | CDAN1 | ILMN_168162 | 2.29 |
| NM_182687.1 | PKMYT1 | ILMN_1154 | 2.28 |
| XM_944104.2 | LOC653232 | ILMN_41197 | 2.28 |
| NM_207331.2 | LOC153561 | ILMN_1879 | 2.26 |
| NM_005667.2 | RNF103 | ILMN_17861 | 2.26 |
| NM_005334.2 | HCFC1 | ILMN_24237 | 2.26 |
| NM_006426.1 | DPYSL4 | ILMN_175746 | 2.26 |
| NM_170721.1 | MSI2 | ILMN_25750 | 2.24 |
| NM_000383.1 | AIRE | ILMN_179368 | 2.23 |
| XM_370865.4 | LOC388122 | ILMN_46143 | 2.22 |
| NR_002450.1 | SNORD68 | ILMN_25967 | 2.22 |
| NM_013412.1 | RABL2A | ILMN_12484 | 2.22 |
| NM_001677.3 | ATP1B1 | ILMN_25542 | 2.21 |
| NM_001013685.1 | LOC401357 | ILMN_29013 | 2.2 |
| XM_934471.1 | LOC399942 | ILMN_32988 | 2.18 |
| NM_182776.1 | MCM7 | ILMN_1133 | 2.17 |
| NM_145701.1 | CDCA4 | ILMN_5601 | 2.15 |
| NM_001280.1 | CIRBP | ILMN_24327 | 2.15 |
| NM_002959.4 | SORT1 | ILMN_165748 | 2.14 |
| NM_014694.2 | ADAMTSL2 | ILMN_697 | 2.13 |
| NM_004075.2 | CRY1 | ILMN_6263 | 2.13 |
| NM_002996.3 | CX3CL1 | ILMN_9636 | 2.13 |
| NM_015447.1 | CAMSAP1 | ILMN_815 | 2.13 |
| NM_178014.2 | TUBB | ILMN_23399 | 2.13 |
| NM_015348.1 | TMEM131 | ILMN_308809 | 2.12 |
| NM_005911.4 | MAT2A | ILMN_25630 | 2.12 |
| NM_032389.3 | ARFGAP2 | ILMN_12944 | 2.11 |
| NM_003119.2 | SPG7 | ILMN_26332 | 2.11 |
| NM_001614.2 | ACTG1 | ILMN_24353 | 2.11 |
| NM_001095.2 | ACCN2 | ILMN_27416 | 2.09 |
| XM_001127981.1 | LOC728014 | ILMN_169164 | 2.09 |
| NM_001621.2 | AHR | ILMN_138365 | 2.09 |
| NM_005993.4 | TBCD | ILMN_171092 | 2.08 |
| NM_014281.3 | PUF60 | ILMN_14897 | 2.08 |
| NM_078470.2 | COX15 | ILMN_13504 | 2.07 |
| NM_080702.2 | BAT3 | ILMN_4429 | 2.07 |
| NM_020410.1 | ATP13A1 | ILMN_12379 | 2.06 |
| NM_001014979.1 | LOC90835 | ILMN_8821 | 2.05 |
| XR_019339.1 | LOC643668 | ILMN_179350 | 2.05 |
| NM_022910.1 | NDRG4 | ILMN_8824 | 2.05 |
| NM_006516.1 | SLC2A1 | ILMN_421 | 2.04 |
| NM_001031827.1 | BOLA2 | ILMN_4509 | 2.04 |
| NM_032704.2 | TUBA1C | ILMN_28729 | 2.04 |
| NM_005157.3 | ABL1 | ILMN_4033 | 2.03 |
| NM_006009.2 | TUBA1A | ILMN_1089 | 2.03 |
| NM_001003.2 | RPLP1 | ILMN_23181 | 2.02 |
| NM_001029862.1 | ANKRD30B | ILMN_7263 | 2.02 |
| NM_001042465.1 | PSAP | ILMN_179975 | 2.02 |
| NM_006731.2 | FKTN | ILMN_6512 | 2.01 |
| XR_019152.1 | LOC644584 | ILMN_163817 | 2 |
| XM_001126418.1 | LOC727935 | ILMN_181411 | 2 |
| NR_003664.1 | LOC389517 | ILMN_307371 | 2 |
| NM_021009.1 | UBC | ILMN_160470 | 2 |
| XM_932717.2 | LOC643224 | ILMN_34053 | 1.99 |
| NM_006958.2 | ZNF16 | ILMN_17198 | 1.99 |
| NM_018050.2 | MANSC1 | ILMN_14144 | 1.98 |
| NM_001287.3 | CLCN7 | ILMN_8600 | 1.98 |
| NM_004091.2 | E2F2 | ILMN_19730 | 1.97 |
| NM_032794.1 | SLC44A4 | ILMN_14709 | 1.97 |
| NM_001046.2 | SLC12A2 | ILMN_14384 | 1.96 |
| NM_152834.2 | TMEM18 | ILMN_8053 | 1.96 |
| NM_001018052.1 | POLR3H | ILMN_8571 | 1.96 |
| NM_172014.1 | TNFSF14 | ILMN_9666 | 1.96 |
| NM_002482.2 | NASP | ILMN_21654 | 1.96 |
| NM_003086.2 | SNAPC4 | ILMN_180505 | 1.96 |
| NM_020933.2 | ZNF317 | ILMN_22884 | 1.95 |
| NM_022720.5 | DGCR8 | ILMN_1552 | 1.95 |
| NM_005386.2 | NNAT | ILMN_16198 | 1.94 |
| NM_153188.2 | TNPO1 | ILMN_29083 | 1.94 |
| NM_001008237.1 | TTC32 | ILMN_4829 | 1.93 |
| NM_175847.1 | PTBP1 | ILMN_20407 | 1.93 |
| NM_080386.1 | TUBA3D | ILMN_30319 | 1.92 |
| NM_003486.5 | SLC7A5 | ILMN_25446 | 1.92 |
| NM_005766.2 | FARP1 | ILMN_15608 | 1.92 |
| XM_926594.2 | LOC642502 | ILMN_31759 | 1.91 |
| NM_000617.1 | SLC11A2 | ILMN_10129 | 1.91 |
| NM_021009.3 | UBC | ILMN_8850 | 1.9 |
| NM_001496.3 | GFRA3 | ILMN_8392 | 1.89 |
| NM_020121.2 | UGCGL2 | ILMN_21506 | 1.89 |
| NM_001080485.1 | ZNF275 | ILMN_180340 | 1.89 |
| NM_006159.1 | NELL2 | ILMN_26383 | 1.89 |
| NM_014380.1 | NGFRAP1 | ILMN_7162 | 1.89 |
| NM_003045.3 | SLC7A1 | ILMN_162673 | 1.89 |
| NM_206852.1 | RTN1 | ILMN_3435 | 1.89 |
| NM_013336.3 | SEC61A1 | ILMN_9397 | 1.88 |
| NM_006372.3 | SYNCRIP | ILMN_28470 | 1.87 |
| NM_182492.1 | LRP5L | ILMN_650 | 1.86 |
| NM_031263.1 | HNRPK | ILMN_16515 | 1.86 |
| NM_001009936.1 | PHF19 | ILMN_20905 | 1.85 |
| XM_379215.2 | LOC132241 | ILMN_37830 | 1.85 |
| NM_181784.1 | SPRED2 | ILMN_12131 | 1.85 |
| NM_003966.2 | SEMA5A | ILMN_183828 | 1.85 |
| NM_005916.3 | MCM7 | ILMN_1986 | 1.85 |
| XM_930995.1 | LOC653086 | ILMN_31021 | 1.85 |
| NM_201559.2 | FOXO3 | ILMN_15525 | 1.85 |
| XR_019449.1 | LOC644422 | ILMN_166674 | 1.84 |
| NM_006739.3 | MCM5 | ILMN_20107 | 1.84 |
| NM_178231.1 | ALS2CR14 | ILMN_947 | 1.83 |
| NM_033419.3 | PERLD1 | ILMN_12215 | 1.82 |
| NM_015352.1 | POFUT1 | ILMN_7876 | 1.82 |
| XM_942991.2 | LOC642934 | ILMN_39429 | 1.82 |
| NM_001025091.1 | ABCF1 | ILMN_179040 | 1.82 |
| XM_497029.2 | LOC441408 | ILMN_31941 | 1.81 |
| NM_004516.2 | ILF3 | ILMN_12252 | 1.81 |
| NM_025084.1 | FLJ22795 | ILMN_1721 | 1.8 |
| NR_002139.1 | HCG4 | ILMN_12590 | 1.8 |
| NM_006997.2 | TACC2 | ILMN_16130 | 1.8 |
| NM_001924.2 | GADD45A | ILMN_17355 | 1.8 |
| XM_930178.1 | LOC645018 | ILMN_33646 | 1.8 |
| NM_033109.2 | PNPT1 | ILMN_22316 | 1.8 |
| NM_032195.1 | SON | ILMN_8462 | 1.79 |
| NM_001006115.2 | IHPK1 | ILMN_8379 | 1.79 |
| NM_017827.2 | SARS2 | ILMN_20962 | 1.79 |
| NM_003819.2 | PABPC4 | ILMN_18446 | 1.79 |
| NM_015720.1 | PODXL2 | ILMN_26815 | 1.78 |
| NM_014374.1 | REPIN1 | ILMN_1054 | 1.78 |
| NM_003713.3 | PPAP2B | ILMN_3538 | 1.77 |
| NM_178517.3 | PIGW | ILMN_162681 | 1.76 |
| NM_020822.1 | KCNT1 | ILMN_21599 | 1.76 |
| XM_929980.2 | LOC647000 | ILMN_34401 | 1.76 |
| NM_152280.2 | SYT11 | ILMN_23967 | 1.76 |
| NM_018150.2 | C1orf164 | ILMN_11157 | 1.75 |
| NR_003659.1 | FAM39DP | ILMN_307683 | 1.75 |
| NR_003264.1 | SDHALP1 | ILMN_175200 | 1.75 |
| NM_001294.1 | CLPTM1 | ILMN_22488 | 1.74 |
| NM_016481.3 | C9orf156 | ILMN_12842 | 1.73 |
| NM_005128.2 | DOPEY2 | ILMN_164626 | 1.73 |
| NM_020664.3 | DECR2 | ILMN_7935 | 1.73 |
| NM_006005.2 | WFS1 | ILMN_18545 | 1.73 |
| NM_004598.3 | SPOCK1 | ILMN_25886 | 1.72 |
| NM_005627.2 | SGK | ILMN_2451 | 1.72 |
| NM_016605.1 | FAM53C | ILMN_11637 | 1.72 |
| NM_003749.2 | IRS2 | ILMN_167991 | 1.72 |
| NM_016644.1 | PRR16 | ILMN_4368 | 1.72 |
| NM_031942.4 | CDCA7 | ILMN_33249 | 1.72 |
| NM_001008938.1 | CKAP5 | ILMN_12487 | 1.72 |
| NM_022553.4 | VPS52 | ILMN_11319 | 1.71 |
| NM_007367.2 | RALY | ILMN_29685 | 1.71 |
| NM_000790.2 | DDC | ILMN_13520 | 1.71 |
| XM_936467.2 | BEXL1 | ILMN_37437 | 1.71 |
| XR_018848.1 | LOC650369 | ILMN_169499 | 1.71 |
| NM_004859.3 | CLTC | ILMN_171089 | 1.7 |
| NM_032520.3 | GNPTG | ILMN_28173 | 1.7 |
| NM_000479.2 | AMH | ILMN_171371 | 1.7 |
| NM_024313.1 | MGC3731 | ILMN_137471 | 1.7 |
| NM_015124.2 | GRAMD4 | ILMN_12136 | 1.69 |
| NM_052917.2 | GALNT13 | ILMN_180483 | 1.69 |
| NM_023080.1 | C8orf33 | ILMN_15901 | 1.69 |
| NM_001039675.1 | UNC45A | ILMN_40255 | 1.69 |
| XM_940209.1 | KIAA0194 | ILMN_37512 | 1.69 |
| NM_004526.2 | MCM2 | ILMN_183916 | 1.69 |
| NM_001037283.1 | EIF3B | ILMN_1524 | 1.69 |
| NM_173042.2 | IL18BP | ILMN_30884 | 1.68 |
| NM_003183.4 | ADAM17 | ILMN_165100 | 1.68 |
| XM_001131589.1 | LOC643446 | ILMN_170528 | 1.68 |
| NM_001275.3 | CHGA | ILMN_23390 | 1.68 |
| NM_007349.3 | PAXIP1 | ILMN_1633 | 1.67 |
| NM_013291.2 | CPSF1 | ILMN_22094 | 1.67 |
| NM_006392.2 | NOL5A | ILMN_13841 | 1.67 |
| NM_178831.4 | GATS | ILMN_18755 | 1.67 |
| NM_001012614.1 | CTBP1 | ILMN_21952 | 1.67 |
| NM_020728.1 | FAM62B | ILMN_19173 | 1.67 |
| NM_005243.2 | EWSR1 | ILMN_17011 | 1.67 |
| NM_005561.2 | LAMP1 | ILMN_27826 | 1.67 |
| NM_032621.2 | BEX2 | ILMN_24134 | 1.66 |
| NM_016564.3 | CEND1 | ILMN_1492 | 1.65 |
| NM_002333.1 | LRP3 | ILMN_12327 | 1.65 |
| NM_001006610.1 | SIAH1 | ILMN_9220 | 1.65 |
| NM_006110.1 | CD2BP2 | ILMN_9406 | 1.65 |
| XR_017492.1 | LOC644330 | ILMN_164787 | 1.64 |
| NM_014747.2 | RIMS3 | ILMN_21581 | 1.64 |
| NM_148174.2 | AZIN1 | ILMN_4931 | 1.63 |
| NM_024519.2 | FAM65A | ILMN_17641 | 1.63 |
| NM_001567.2 | INPPL1 | ILMN_20903 | 1.63 |
| NM_004489.4 | GPS2 | ILMN_10256 | 1.63 |
| NM_001048201.1 | UHRF1 | ILMN_162952 | 1.63 |
| NM_006749.3 | SLC20A2 | ILMN_29659 | 1.62 |
| NM_018050.2 | MANSC1 | ILMN_14144 | 1.62 |
| NM_145294.4 | WDR90 | ILMN_29490 | 1.62 |
| NM_002048.1 | GAS1 | ILMN_175833 | 1.62 |
| NM_020211.1 | RGMA | ILMN_26077 | 1.61 |
| NM_003195.4 | TCEA2 | ILMN_10248 | 1.61 |
| NM_177414.1 | PPAP2B | ILMN_5681 | 1.61 |
| XM_945045.1 | LOC649679 | ILMN_34833 | 1.61 |
| NM_020307.2 | CCNL1 | ILMN_14683 | 1.61 |
| NM_022489.2 | C14orf173 | ILMN_41230 | 1.61 |
| NM_000274.1 | OAT | ILMN_8426 | 1.61 |
| NM_005255.1 | GAK | ILMN_21151 | 1.61 |
| NM_016333.2 | SRRM2 | ILMN_21088 | 1.61 |
| NM_021136.2 | RTN1 | ILMN_174587 | 1.61 |
| NM_002915.3 | RFC3 | ILMN_11616 | 1.6 |
| NM_018249.4 | CDK5RAP2 | ILMN_9876 | 1.6 |
| NM_005385.3 | NKTR | ILMN_23378 | 1.6 |
| NM_004968.2 | ICA1 | ILMN_29651 | 1.6 |
| NM_001386.4 | DPYSL2 | ILMN_9671 | 1.6 |
| NM_024900.3 | PHF17 | ILMN_1535 | 1.59 |
| NM_001642.1 | APLP2 | ILMN_19935 | 1.59 |
| NM_007055.2 | POLR3A | ILMN_1449 | 1.59 |
| NM_001014432.1 | AKT1 | ILMN_4841 | 1.59 |
| NM_024083.2 | ASPSCR1 | ILMN_9446 | 1.59 |
| NM_023009.4 | MARCKSL1 | ILMN_17241 | 1.59 |
| NM_001562.2 | IL18 | ILMN_167736 | 1.59 |
| NM_021190.1 | PTBP2 | ILMN_556 | 1.58 |
| XM_944429.1 | LOC653994 | ILMN_38337 | 1.58 |
| NM_014363.3 | SACS | ILMN_180142 | 1.57 |
| XM_931434.2 | LOC400027 | ILMN_35789 | 1.57 |
| NM_001077442.1 | HNRNPC | ILMN_165238 | 1.57 |
| NM_004656.2 | BAP1 | ILMN_17024 | 1.56 |
| NM_021190.1 | PTBP2 | ILMN_556 | 1.56 |
| NM_022781.4 | RNF38 | ILMN_40416 | 1.56 |
| NM_080491.1 | GAB2 | ILMN_3317 | 1.56 |
| NM_020246.2 | SLC12A9 | ILMN_12081 | 1.56 |
| NM_005104.2 | BRD2 | ILMN_13354 | 1.56 |
| NM_021737.1 | CLCN6 | ILMN_6195 | 1.55 |
| NM_020695.3 | REXO1 | ILMN_20923 | 1.54 |
| NR_003239.1 | SNHG11 | ILMN_165269 | 1.54 |
| NM_033063.1 | MAP6 | ILMN_6882 | 1.54 |
| NM_003565.1 | ULK1 | ILMN_2158 | 1.54 |
| NM_001111.3 | ADAR | ILMN_20593 | 1.54 |
| NM_002202.1 | ISL1 | ILMN_25965 | 1.54 |
| XM_495939.3 | KIAA1545 | ILMN_40920 | 1.53 |
| NM_006715.2 | MAN2C1 | ILMN_685 | 1.53 |
| NM_021070.2 | LTBP3 | ILMN_918 | 1.53 |
| NM_003076.3 | SMARCD1 | ILMN_16093 | 1.53 |
| NM_001034025.1 | ERP29 | ILMN_8686 | 1.53 |
| XM_934113.1 | LOC653489 | ILMN_42664 | 1.52 |
| NM_178014.2 | TUBB | ILMN_23399 | 1.52 |
| NM_013313.3 | YPEL1 | ILMN_26647 | 1.51 |
| NM_004560.2 | ROR2 | ILMN_22834 | 1.51 |
| NM_080670.2 | SLC35A4 | ILMN_8862 | 1.51 |
| NM_000743.2 | CHRNA3 | ILMN_23268 | 1.51 |
| NM_001017980.2 | LOC203547 | ILMN_163926 | 1.51 |
| NM_201398.1 | FLAD1 | ILMN_17093 | 1.51 |
| NM_001013839.1 | EXOC7 | ILMN_25212 | 1.51 |
| NM_172249.1 | CSF2RA | ILMN_5061 | 1.51 |
| NM_201397.1 | GPX1 | ILMN_10376 | 1.51 |
| NM_002938.2 | RNF4 | ILMN_26467 | 1.5 |
| NM_024698.4 | SLC25A22 | ILMN_13935 | 1.5 |
| NM_144594.1 | GTSF1 | ILMN_17221 | -1.5 |
| NM_019083.1 | CCDC76 | ILMN_2869 | -1.51 |
| NM_018062.2 | FANCL | ILMN_24728 | -1.51 |
| NM_002553.2 | ORC5L | ILMN_6212 | -1.51 |
| NM_006608.1 | PHTF1 | ILMN_25225 | -1.51 |
| NM_004156.2 | PPP2CB | ILMN_21592 | -1.51 |
| NM_024321.3 | RBM42 | ILMN_182570 | -1.51 |
| NM_018373.1 | SYNJ2BP | ILMN_8166 | -1.51 |
| XM_942540.1 | SAPS2 | ILMN_138398 | -1.52 |
| NM_194298.1 | SLC16A9 | ILMN_19723 | -1.52 |
| NM_001033925.1 | TIAL1 | ILMN_24357 | -1.52 |
| NM_018120.3 | ARMC1 | ILMN_14242 | -1.53 |
| NM_003945.3 | ATP6V0E1 | ILMN_8923 | -1.53 |
| NM_130442.2 | ELMO1 | ILMN_33821 | -1.53 |
| NM_003512.3 | HIST1H2AC | ILMN_26493 | -1.53 |
| NM_153201.1 | HSPA8 | ILMN_14829 | -1.53 |
| NM_194359.1 | RNF41 | ILMN_22848 | -1.53 |
| NM_001008566.1 | TPST2 | ILMN_13248 | -1.53 |
| NM_153018.2 | ZFP3 | ILMN_42182 | -1.53 |
| NM_001040668.1 | BCL2L12 | ILMN_177176 | -1.54 |
| NM_003077.2 | SMARCD2 | ILMN_14227 | -1.54 |
| NM_001012968.2 | SPIN4 | ILMN_4105 | -1.54 |
| NM_018170.2 | P15RS | ILMN_174036 | -1.55 |
| NM_024546.3 | RNF219 | ILMN_38012 | -1.55 |
| NM_133646.2 | ZAK | ILMN_5666 | -1.55 |
| NM_004048.2 | B2M | ILMN_19648 | -1.56 |
| NM_018640.3 | LMO3 | ILMN_15180 | -1.56 |
| NM_018115.2 | SDAD1 | ILMN_2166 | -1.56 |
| NM_021652.1 | SMA4 | ILMN_25253 | -1.56 |
| NM_014140.2 | SMARCAL1 | ILMN_19734 | -1.56 |
| NM_020463.1 | SMEK2 | ILMN_21228 | -1.56 |
| NM_033157.2 | CALD1 | ILMN_2136 | -1.57 |
| NM_001449.3 | FHL1 | ILMN_7975 | -1.57 |
| NM_006158.2 | NEFL | ILMN_22054 | -1.57 |
| NM_001033566.1 | RHOT1 | ILMN_6821 | -1.57 |
| NM_017909.1 | RMND1 | ILMN_29019 | -1.57 |
| NM_014933.2 | SEC31A | ILMN_23819 | -1.57 |
| NM_018428.2 | UTP6 | ILMN_18247 | -1.57 |
| NM_005791.1 | MPHOSPH10 | ILMN_161971 | -1.58 |
| NM_024632.4 | SAP30L | ILMN_18384 | -1.58 |
| NM_178123.3 | SESTD1 | ILMN_17887 | -1.58 |
| NM_153188.2 | TNPO1 | ILMN_29083 | -1.58 |
| NM_001077268.1 | ZFYVE19 | ILMN_175347 | -1.58 |
| NM_020375.2 | C12orf5 | ILMN_183781 | -1.59 |
| NM_012129.2 | CLDN12 | ILMN_11012 | -1.59 |
| NM_013316.2 | CNOT4 | ILMN_22777 | -1.59 |
| NM_001967.3 | EIF4A2 | ILMN_5908 | -1.59 |
| NM_014161.2 | MRPL18 | ILMN_14120 | -1.59 |
| NM_133371.2 | MYOZ3 | ILMN_21305 | -1.59 |
| NM_005833.2 | RABEPK | ILMN_4050 | -1.59 |
| NM_212552.2 | BOLA3 | ILMN_28776 | -1.6 |
| NM_005197.2 | CHES1 | ILMN_182611 | -1.6 |
| NM_024775.9 | GEMIN6 | ILMN_23187 | -1.6 |
| NM_025196.2 | GRPEL1 | ILMN_7749 | -1.6 |
| NM_153682.2 | PIGP | ILMN_18625 | -1.6 |
| NM_199462.1 | RIPK5 | ILMN_1333 | -1.6 |
| NM_032530.1 | ZNF594 | ILMN_309021 | -1.6 |
| NM_018369.1 | DEPDC1B | ILMN_16725 | -1.61 |
| NM_016297.2 | PCYOX1 | ILMN_15130 | -1.61 |
| NM_001082576.1 | RBM9 | ILMN_307545 | -1.61 |
| NM_022830.1 | TUT1 | ILMN_6523 | -1.61 |
| NM_032312.2 | YIPF4 | ILMN_16255 | -1.61 |
| NM_013300.1 | C12orf24 | ILMN_24807 | -1.62 |
| NM_001031713.2 | CCDC90A | ILMN_9159 | -1.62 |
| NM_018131.3 | CEP55 | ILMN_6470 | -1.62 |
| NM_017812.2 | CHCHD3 | ILMN_23539 | -1.62 |
| NM_017526.2 | LEPROT | ILMN_27032 | -1.62 |
| NM_002350.1 | LYN | ILMN_10095 | -1.62 |
| NM_017934.4 | PHIP | ILMN_171544 | -1.62 |
| NM_002918.3 | RFX1 | ILMN_178968 | -1.62 |
| NM_014305.2 | TGDS | ILMN_30985 | -1.62 |
| NM_016399.2 | TRIAP1 | ILMN_13988 | -1.62 |
| NM_017656.2 | ZNF562 | ILMN_23559 | -1.62 |
| NM_182648.1 | BAZ1A | ILMN_24991 | -1.63 |
| NM_017693.2 | BIVM | ILMN_181297 | -1.63 |
| NM_032120.1 | DKFZP564O0523 | ILMN_19468 | -1.63 |
| NM_016499.3 | MGC13379 | ILMN_180361 | -1.63 |
| NM_001080501.1 | MGC3196 | ILMN_181711 | -1.63 |
| NM_004885.1 | NPFFR2 | ILMN_20676 | -1.63 |
| NM_001033566.1 | RHOT1 | ILMN_6821 | -1.63 |
| NM_152313.2 | SLC36A4 | ILMN_13325 | -1.63 |
| NM_175907.3 | ZADH2 | ILMN_5633 | -1.63 |
| NM_032194.1 | BXDC1 | ILMN_29712 | -1.64 |
| NM_001037494.1 | DYNLL1 | ILMN_14802 | -1.64 |
| NM_001466.2 | FZD2 | ILMN_12499 | -1.64 |
| NM_004891.2 | MRPL33 | ILMN_12897 | -1.64 |
| NM_000913.3 | OPRL1 | ILMN_6491 | -1.64 |
| NM_032305.1 | POLR3GL | ILMN_23668 | -1.64 |
| NM_032826.3 | SLC35B4 | ILMN_19120 | -1.64 |
| NM_003143.1 | SSBP1 | ILMN_30122 | -1.64 |
| NM_005744.2 | ARIH1 | ILMN_16556 | -1.65 |
| XM_371655.3 | LOC389137 | ILMN_163284 | -1.65 |
| NM_002413.3 | MGST2 | ILMN_8759 | -1.65 |
| NM_000997.3 | RPL37 | ILMN_138392 | -1.65 |
| NM_175071.1 | APTX | ILMN_6739 | -1.66 |
| NM_032439.1 | PHYHIPL | ILMN_22045 | -1.66 |
| NM_003475.2 | RASSF7 | ILMN_12457 | -1.66 |
| NM_001003793.1 | RBMS3 | ILMN_16411 | -1.66 |
| NM_005652.2 | TERF2 | ILMN_21134 | -1.66 |
| NM_138390.2 | TMEM169 | ILMN_165130 | -1.66 |
| NM_023011.2 | UPF3A | ILMN_28964 | -1.66 |
| NM_018353.3 | C14orf106 | ILMN_5745 | -1.67 |
| NM_003503.2 | CDC7 | ILMN_20584 | -1.67 |
| NM_001896.2 | CSNK2A2 | ILMN_16798 | -1.67 |
| NM_001394.5 | DUSP4 | ILMN_17730 | -1.67 |
| NM_032138.3 | KBTBD7 | ILMN_181309 | -1.67 |
| NM_001003793.1 | RBMS3 | ILMN_16411 | -1.67 |
| NM_173647.2 | RNF149 | ILMN_10320 | -1.67 |
| NM_005680.1 | TAF1B | ILMN_13234 | -1.67 |
| NM_198434.1 | AURKA | ILMN_12352 | -1.68 |
| NM_006519.1 | DYNLT1 | ILMN_7436 | -1.68 |
| NM_003800.3 | RNGTT | ILMN_17056 | -1.68 |
| NM_006304.1 | SHFM1 | ILMN_26583 | -1.68 |
| NM_000819.3 | GART | ILMN_22974 | -1.69 |
| NM_015969.2 | MRPS17 | ILMN_26133 | -1.69 |
| NM_002717.2 | PPP2R2A | ILMN_24841 | -1.69 |
| NM_016625.2 | RSRC1 | ILMN_14978 | -1.69 |
| NM_018286.2 | TMEM100 | ILMN_4881 | -1.69 |
| NM_001078651.1 | TMEM134 | ILMN_176754 | -1.69 |
| NM_053067.1 | UBQLN1 | ILMN_9768 | -1.69 |
| NM_138418.2 | C16orf14 | ILMN_9509 | -1.7 |
| NM_024090.1 | ELOVL6 | ILMN_11340 | -1.7 |
| NM_024647.4 | NUP43 | ILMN_28463 | -1.7 |
| NM_016108.2 | AIG1 | ILMN_22004 | -1.71 |
| NM_019058.2 | DDIT4 | ILMN_13176 | -1.71 |
| NM_138794.2 | LYPLAL1 | ILMN_25005 | -1.71 |
| NM_014168.2 | METTL5 | ILMN_9336 | -1.71 |
| NM_145274.2 | TMEM99 | ILMN_25105 | -1.71 |
| NM_018244.3 | UQCC | ILMN_26543 | -1.71 |
| NM_005830.2 | MRPS31 | ILMN_6293 | -1.72 |
| NM_006310.2 | NPEPPS | ILMN_184074 | -1.72 |
| NM_152132.1 | PSMA3 | ILMN_16452 | -1.72 |
| NM_001007239.1 | KIAA0859 | ILMN_25045 | -1.73 |
| NM_003998.2 | NFKB1 | ILMN_161884 | -1.73 |
| NM_003620.2 | PPM1D | ILMN_163927 | -1.73 |
| NM_021652.2 | SMA4 | ILMN_25253 | -1.73 |
| NM_032361.1 | THOC3 | ILMN_17969 | -1.73 |
| NM_005192.2 | CDKN3 | ILMN_4098 | -1.74 |
| NM_006090.3 | CEPT1 | ILMN_14637 | -1.74 |
| NM_014463.1 | LSM3 | ILMN_23516 | -1.74 |
| NM_001031727.2 | MGC3207 | ILMN_2776 | -1.74 |
| NM_032673.2 | PCGF1 | ILMN_5720 | -1.74 |
| NM_001033925.1 | TIAL1 | ILMN_24357 | -1.74 |
| NM_152902.3 | TIPRL | ILMN_13476 | -1.74 |
| NM_014167.2 | CCDC59 | ILMN_12564 | -1.75 |
| NM_005680.1 | TAF1B | ILMN_13234 | -1.75 |
| NM_032574.2 | DPY30 | ILMN_18534 | -1.76 |
| NM_030917.2 | FIP1L1 | ILMN_6961 | -1.76 |
| NM_014939.2 | KIAA1012 | ILMN_20638 | -1.76 |
| NM_003744.5 | NUMB | ILMN_24350 | -1.76 |
| NM_005707.1 | PDCD7 | ILMN_179659 | -1.76 |
| NM_003864.3 | SAP30 | ILMN_31250 | -1.76 |
| NM_012319.2 | SLC39A6 | ILMN_170037 | -1.76 |
| NM_001039802.1 | CDC42 | ILMN_38161 | -1.77 |
| NM_020899.2 | ZBTB4 | ILMN_5011 | -1.77 |
| NM_033426.2 | KIAA1737 | ILMN_176915 | -1.78 |
| NM_001080546.1 | LOC219854 | ILMN_168339 | -1.78 |
| XM_943005.1 | LOC642236 | ILMN_31082 | -1.78 |
| XM_001130106.1 | LOC646463 | ILMN_181743 | -1.78 |
| NM_002408.3 | MGAT2 | ILMN_163431 | -1.78 |
| NM_015017.3 | USP33 | ILMN_176756 | -1.78 |
| NM_000465.1 | BARD1 | ILMN_1301 | -1.79 |
| NM_030805.2 | LMAN2L | ILMN_1985 | -1.79 |
| NM_006190.3 | ORC2L | ILMN_182860 | -1.79 |
| NM_018343.1 | RIOK2 | ILMN_16482 | -1.79 |
| NR_002166.1 | SEDLP | ILMN_1258 | -1.79 |
| NM_207037.1 | TCF12 | ILMN_182697 | -1.79 |
| NM_022037.1 | TIA1 | ILMN_30157 | -1.79 |
| NM_014886.3 | TINP1 | ILMN_8436 | -1.79 |
| NM_018480.2 | TMEM126B | ILMN_18826 | -1.79 |
| NM_001007278.1 | TRIM13 | ILMN_14225 | -1.79 |
| NM_015957.1 | APIP | ILMN_15379 | -1.8 |
| NM_001035505.1 | BOLA3 | ILMN_29223 | -1.8 |
| NM_152755.1 | CNPY4 | ILMN_15383 | -1.81 |
| NM_003583.2 | DYRK2 | ILMN_3688 | -1.81 |
| NM_024969.2 | FAM130A2 | ILMN_20070 | -1.81 |
| NM_004544.2 | NDUFA10 | ILMN_7463 | -1.81 |
| NM_152995.4 | NFXL1 | ILMN_43252 | -1.81 |
| NM_021222.1 | PRUNE | ILMN_27601 | -1.81 |
| NM_004180.2 | TANK | ILMN_164387 | -1.81 |
| NM_207038.1 | TCF12 | ILMN_17157 | -1.81 |
| NM_022079.2 | HERC4 | ILMN_8869 | -1.82 |
| NM_181725.2 | METTL2A | ILMN_23067 | -1.82 |
| NM_006191.2 | PA2G4 | ILMN_28541 | -1.82 |
| NM_014252.1 | SLC25A15 | ILMN_139066 | -1.82 |
| NM_006282.2 | STK4 | ILMN_21491 | -1.82 |
| NM_079837.2 | BANP | ILMN_8638 | -1.83 |
| NM_017915.2 | C12orf48 | ILMN_42497 | -1.83 |
| NM_005476.3 | GNE | ILMN_29772 | -1.83 |
| NM_006860.2 | RABL4 | ILMN_4559 | -1.83 |
| NM_003290.1 | TPM4 | ILMN_9334 | -1.83 |
| NM_022740.2 | HIPK2 | ILMN_29690 | -1.84 |
| NM_014672.2 | KIAA0391 | ILMN_30096 | -1.84 |
| NM_001037163.1 | MGC12966 | ILMN_182436 | -1.84 |
| NM_030660.2 | ATXN3 | ILMN_12637 | -1.85 |
| NM_003746.1 | DNCL1 | ILMN_137049 | -1.85 |
| NM_015475.3 | FAM98A | ILMN_16819 | -1.85 |
| NM_001005369.1 | MTIF2 | ILMN_165311 | -1.85 |
| NM_032169.4 | ACAD11 | ILMN_25425 | -1.86 |
| NM_016142.1 | HSD17B12 | ILMN_19305 | -1.86 |
| NM_015948.2 | SLC35B3 | ILMN_20545 | -1.86 |
| NM_139283.1 | PPTC7 | ILMN_11800 | -1.87 |
| NM_138720.1 | HIST1H2BD | ILMN_17622 | -1.88 |
| NM_006597.3 | HSPA8 | ILMN_181529 | -1.88 |
| NM_002486.4 | NCBP1 | ILMN_23411 | -1.88 |
| NM_199487.1 | UQCC | ILMN_16175 | -1.88 |
| NM_014885.3 | ANAPC10 | ILMN_2970 | -1.89 |
| NM_033405.2 | PRIC285 | ILMN_10778 | -1.89 |
| NM_012249.3 | RHOQ | ILMN_2265 | -1.89 |
| NM_144563.2 | RPIA | ILMN_23078 | -1.89 |
| NM_003452.2 | ZNF189 | ILMN_4798 | -1.89 |
| NM_006629.3 | ZNF271 | ILMN_11823 | -1.89 |
| NM_012482.3 | ZNF281 | ILMN_18970 | -1.89 |
| NM_052879.3 | LARP4 | ILMN_2132 | -1.9 |
| NM_018297.2 | NGLY1 | ILMN_15318 | -1.9 |
| NM_144726.1 | RNF145 | ILMN_27136 | -1.9 |
| NM_001080477.1 | ODZ3 | ILMN_179907 | -1.91 |
| NM_133462.2 | TTC14 | ILMN_6310 | -1.91 |
| XM_941876.1 | BRI3BP | ILMN_139088 | -1.92 |
| NM_016053.2 | CCDC53 | ILMN_25394 | -1.92 |
| NM_033319.1 | CENPL | ILMN_21203 | -1.92 |
| NM_017940.2 | NBPF1 | ILMN_163270 | -1.92 |
| NM_032728.2 | PPAPDC3 | ILMN_25638 | -1.92 |
| NM_016395.2 | PTPLAD1 | ILMN_9196 | -1.92 |
| NM_032026.2 | TATDN1 | ILMN_17501 | -1.92 |
| NM_002095.4 | GTF2E2 | ILMN_4316 | -1.93 |
| XM_928675.1 | LOC653308 | ILMN_43749 | -1.93 |
| NM_001002755.1 | NFU1 | ILMN_9748 | -1.93 |
| NM_013388.4 | PREB | ILMN_6913 | -1.93 |
| NM_012241.2 | SIRT5 | ILMN_18454 | -1.93 |
| NM_005868.4 | BET1 | ILMN_4222 | -1.94 |
| NM_032490.4 | C14orf142 | ILMN_166160 | -1.94 |
| NM_032299.2 | DCUN1D5 | ILMN_18117 | -1.94 |
| NM_002491.1 | NDUFB3 | ILMN_22320 | -1.94 |
| NM_007342.1 | NUPL2 | ILMN_2154 | -1.94 |
| NM_005131.2 | THOC1 | ILMN_19739 | -1.94 |
| NM_170662.3 | CBLB | ILMN_18286 | -1.95 |
| NM_139286.3 | CDC26 | ILMN_18022 | -1.95 |
| NM_181837.1 | ORC3L | ILMN_3770 | -1.95 |
| NM_002690.1 | POLB | ILMN_15404 | -1.95 |
| NM_053067.1 | UBQLN1 | ILMN_9768 | -1.95 |
| NM_001007027.2 | ALG8 | ILMN_176006 | -1.96 |
| NM_022346.3 | NCAPG | ILMN_23620 | -1.96 |
| NM_138797.1 | ANKRD54 | ILMN_21813 | -1.97 |
| NM_181708.1 | BCDIN3D | ILMN_18065 | -1.97 |
| NM_003642.2 | HAT1 | ILMN_24074 | -1.98 |
| NM_030808.3 | NDEL1 | ILMN_20362 | -1.98 |
| NM_014142.2 | NUDT5 | ILMN_1656 | -1.98 |
| NM_001042601.1 | TTC14 | ILMN_163780 | -1.98 |
| NM_198893.1 | ZNF160 | ILMN_25264 | -1.98 |
| NM_001866.2 | COX7B | ILMN_19298 | -1.99 |
| NM_017906.2 | PAK1IP1 | ILMN_24423 | -1.99 |
| NM_031307.2 | PUS3 | ILMN_175536 | -1.99 |
| NM_006602.2 | TCFL5 | ILMN_12278 | -1.99 |
| NM_182547.2 | TMED4 | ILMN_30359 | -1.99 |
| NM_014322.2 | OPN3 | ILMN_166169 | -2 |
| NM_020706.1 | SFRS15 | ILMN_18564 | -2 |
| NM_005087.2 | FXR1 | ILMN_18674 | -2.01 |
| NM_015480.1 | PVRL3 | ILMN_2284 | -2.01 |
| NM_024056.2 | TMEM106C | ILMN_7003 | -2.01 |
| NM_022087.2 | GALNT11 | ILMN_5237 | -2.02 |
| NM_000645.2 | AGL | ILMN_1173 | -2.03 |
| NM_001042426.1 | CENPA | ILMN_180589 | -2.03 |
| NM_001984.1 | ESD | ILMN_14903 | -2.03 |
| NM_016048.1 | ISOC1 | ILMN_15311 | -2.03 |
| NM_006210.1 | PEG3 | ILMN_27573 | -2.03 |
| NM_005402.2 | RALA | ILMN_164730 | -2.03 |
| NM_017446.3 | MRPL39 | ILMN_4651 | -2.04 |
| NM_199044.2 | NSUN4 | ILMN_23916 | -2.04 |
| NM_006811.2 | SERINC3 | ILMN_10490 | -2.04 |
| NM_002319.2 | LRCH4 | ILMN_139402 | -2.05 |
| NM_014046.2 | MRPS18B | ILMN_8749 | -2.05 |
| NM_024585.2 | ARMC7 | ILMN_163623 | -2.06 |
| NM_005188.2 | CBL | ILMN_172998 | -2.06 |
| NM_152789.2 | FAM133B | ILMN_1247 | -2.06 |
| NM_181702.1 | GEM | ILMN_16170 | -2.06 |
| NM_005708.2 | GPC6 | ILMN_16550 | -2.06 |
| NM_018847.2 | KLHL9 | ILMN_20376 | -2.06 |
| NM_001085363.1 | MEX3D | ILMN_307190 | -2.06 |
| NM_018361.2 | AGPAT5 | ILMN_9737 | -2.07 |
| NM_031267.1 | CDC2L5 | ILMN_29859 | -2.07 |
| XM_001125680.1 | LOC730432 | ILMN_165880 | -2.07 |
| NM_002491.1 | NDUFB3 | ILMN_22320 | -2.07 |
| NM_003729.2 | RTCD1 | ILMN_11697 | -2.07 |
| NM_018155.1 | SLC25A36 | ILMN_3338 | -2.07 |
| NM_207118.1 | GTF2H5 | ILMN_26206 | -2.08 |
| NM_004503.3 | HOXC6 | ILMN_15669 | -2.08 |
| NM_024697.1 | ZNF385D | ILMN_13191 | -2.08 |
| NM_015608.2 | C10orf137 | ILMN_22392 | -2.09 |
| NM_015395.1 | DKFZP434B0335 | ILMN_11830 | -2.09 |
| NM_006391.1 | IPO7 | ILMN_28842 | -2.09 |
| NM_006164.2 | NFE2L2 | ILMN_9669 | -2.09 |
| NM_005388.3 | PDCL | ILMN_34020 | -2.09 |
| NM_012110.2 | CHIC2 | ILMN_24345 | -2.1 |
| NM_012154.2 | EIF2C2 | ILMN_25413 | -2.1 |
| NM_018846.2 | KLHL7 | ILMN_21425 | -2.1 |
| NM_021970.2 | MAP2K1IP1 | ILMN_13073 | -2.1 |
| NM_205843.1 | NFIC | ILMN_22629 | -2.1 |
| NM_002669.2 | PLRG1 | ILMN_22972 | -2.1 |
| NM_001099222.1 | IFT74 | ILMN_306953 | -2.11 |
| NM_016020.1 | TFB1M | ILMN_3033 | -2.11 |
| NM_021156.2 | TXNDC13 | ILMN_23065 | -2.11 |
| XM_930884.1 | LOC653080 | ILMN_32261 | -2.12 |
| NM_145117.3 | NAV2 | ILMN_8536 | -2.12 |
| NM_006416.3 | SLC35A1 | ILMN_23284 | -2.12 |
| NM_031453.2 | FAM107B | ILMN_2236 | -2.13 |
| NM_003718.3 | CDC2L5 | ILMN_872 | -2.15 |
| NM_001013406.1 | KRIT1 | ILMN_15411 | -2.15 |
| NM_138798.1 | MITD1 | ILMN_27516 | -2.16 |
| NM_002870.2 | RAB13 | ILMN_26464 | -2.16 |
| NM_014487.3 | ZNF330 | ILMN_6878 | -2.16 |
| NM_170783.1 | ZNRD1 | ILMN_1419 | -2.17 |
| NM_033064.3 | ATCAY | ILMN_27014 | -2.18 |
| NM_152415.1 | VPS37A | ILMN_12702 | -2.18 |
| NM_001006622.1 | WDR33 | ILMN_6581 | -2.18 |
| NM_005513.1 | GTF2E1 | ILMN_175401 | -2.19 |
| NM_033402.3 | LRRCC1 | ILMN_15234 | -2.19 |
| NM_032280.1 | ZCCHC9 | ILMN_25119 | -2.2 |
| NM_015942.3 | MTERFD1 | ILMN_24756 | -2.22 |
| NM_006392.2 | NOL5A | ILMN_13841 | -2.22 |
| NM_006117.2 | PECI | ILMN_7427 | -2.23 |
| NM_012210.3 | TRIM32 | ILMN_14426 | -2.23 |
| XM_945544.1 | UBE2Z | ILMN_137054 | -2.23 |
| NM_201280.1 | MUTED | ILMN_21576 | -2.25 |
| NM_001002019.1 | PUS1 | ILMN_13055 | -2.25 |
| NM_014254.1 | TMEM5 | ILMN_26271 | -2.26 |
| NM_019116.2 | UBFD1 | ILMN_179383 | -2.27 |
| NM_025136.1 | OPA3 | ILMN_11296 | -2.28 |
| NM_016166.1 | PIAS1 | ILMN_16806 | -2.28 |
| NM_016010.1 | C8orf70 | ILMN_13979 | -2.29 |
| NM_020236.2 | MRPL1 | ILMN_22997 | -2.29 |
| NM_001003800.1 | BICD2 | ILMN_11310 | -2.31 |
| NM_003583.3 | DYRK2 | ILMN_3688 | -2.31 |
| NM_005885.2 | MARCH6 | ILMN_174083 | -2.32 |
| XR_015313.1 | LOC653080 | ILMN_172174 | -2.32 |
| NM_015523.2 | REXO2 | ILMN_15016 | -2.32 |
| NM_006745.3 | SC4MOL | ILMN_2770 | -2.32 |
| NM_024095.3 | ASB8 | ILMN_165486 | -2.33 |
| NM_017946.2 | FKBP14 | ILMN_18132 | -2.34 |
| NM_001007230.1 | SPOP | ILMN_12838 | -2.34 |
| NM_023010.2 | UPF3B | ILMN_28844 | -2.34 |
| NM_198088.1 | ZNF200 | ILMN_18094 | -2.34 |
| NM_032705.3 | C1orf97 | ILMN_6189 | -2.35 |
| NM_006294.2 | UQCRB | ILMN_26282 | -2.35 |
| NM_145644.1 | MRPL35 | ILMN_20736 | -2.36 |
| NM_016071.2 | MRPS33 | ILMN_4243 | -2.36 |
| NM_001042369.1 | TROVE2 | ILMN_179221 | -2.36 |
| NM_207418.2 | GCUD2 | ILMN_19354 | -2.37 |
| NM_021622.3 | PLEKHA1 | ILMN_9430 | -2.37 |
| NM_024071.2 | ZFYVE21 | ILMN_1317 | -2.37 |
| NM_198401.2 | ANKRD46 | ILMN_9031 | -2.38 |
| NM_002759.1 | EIF2AK2 | ILMN_168435 | -2.38 |
| NM_018357.2 | LARP6 | ILMN_25584 | -2.38 |
| NM_020799.2 | STAMBPL1 | ILMN_1387 | -2.38 |
| NM_017489.1 | TERF1 | ILMN_164297 | -2.38 |
| NM_017895.6 | DDX27 | ILMN_20732 | -2.39 |
| NM_032180.1 | FLJ13305 | ILMN_5829 | -2.4 |
| NM_017816.1 | LYAR | ILMN_23200 | -2.4 |
| NM_007198.2 | PROSC | ILMN_23472 | -2.4 |
| NM_001014812.1 | FAM96A | ILMN_13780 | -2.41 |
| NM_152912.3 | MTIF3 | ILMN_16655 | -2.41 |
| NM_006761.3 | YWHAE | ILMN_18524 | -2.41 |
| NM_006471.2 | MRCL3 | ILMN_25729 | -2.42 |
| NM_018246.2 | CCDC25 | ILMN_5229 | -2.43 |
| NM_012170.2 | FBXO22 | ILMN_5718 | -2.43 |
| NM_005413.1 | SIX3 | ILMN_26476 | -2.43 |
| NM_006281.2 | STK3 | ILMN_26935 | -2.43 |
| NM_014184.2 | CNIH4 | ILMN_9903 | -2.44 |
| XM_939954.2 | LOC388789 | ILMN_39285 | -2.44 |
| XM_929738.1 | LOC646786 | ILMN_38919 | -2.44 |
| XM_944786.1 | LOC650737 | ILMN_40280 | -2.44 |
| NM_006963.3 | ZNF22 | ILMN_165495 | -2.44 |
| NM_004428.2 | EFNA1 | ILMN_14320 | -2.45 |
| NR_003144.1 | LOC723972 | ILMN_180363 | -2.45 |
| NM_018473.2 | THEM2 | ILMN_27212 | -2.45 |
| NM_133465.2 | KIAA1958 | ILMN_17353 | -2.46 |
| NM_018390.2 | PLCXD1 | ILMN_8273 | -2.46 |
| NM_017816.1 | LYAR | ILMN_23200 | -2.47 |
| NM_001100164.1 | PHACTR2 | ILMN_307784 | -2.47 |
| NM_052857.2 | CCDC16 | ILMN_23839 | -2.48 |
| NM_014941.1 | MORC2 | ILMN_12502 | -2.48 |
| NM_007358.2 | MTF2 | ILMN_24749 | -2.48 |
| NM_005749.2 | TOB1 | ILMN_13735 | -2.48 |
| NM_004316.2 | ASCL1 | ILMN_23892 | -2.49 |
| NM_015542.2 | UPF2 | ILMN_21163 | -2.49 |
| NM_024057.2 | NUP37 | ILMN_4147 | -2.51 |
| NM_003002.1 | SDHD | ILMN_6353 | -2.51 |
| NM_003211.3 | TDG | ILMN_29212 | -2.51 |
| NM_152609.1 | C1orf71 | ILMN_21085 | -2.52 |
| NM_001918.2 | DBT | ILMN_169961 | -2.52 |
| NM_015314.2 | KIAA0895 | ILMN_28455 | -2.53 |
| NM_173510.1 | CCDC117 | ILMN_21814 | -2.54 |
| NM_014078.4 | MRPL13 | ILMN_17393 | -2.54 |
| NM_020235.3 | BBX | ILMN_28437 | -2.55 |
| NM_014007.2 | ZBTB43 | ILMN_17837 | -2.55 |
| NM_004365.2 | CETN3 | ILMN_25663 | -2.56 |
| NM_004901.2 | ENTPD4 | ILMN_19012 | -2.57 |
| NM_001827.1 | CKS2 | ILMN_14702 | -2.58 |
| NM_005836.2 | HRSP12 | ILMN_8062 | -2.59 |
| NM_005926.2 | MFAP1 | ILMN_20656 | -2.6 |
| NM_016042.2 | EXOSC3 | ILMN_174330 | -2.61 |
| NM_002338.2 | LSAMP | ILMN_861 | -2.63 |
| NM_016297.2 | PCYOX1 | ILMN_15130 | -2.63 |
| NM_014992.1 | DAAM1 | ILMN_183695 | -2.65 |
| NM_170783.1 | ZNRD1 | ILMN_1419 | -2.65 |
| NM_000628.3 | IL10RB | ILMN_26097 | -2.66 |
| NM_002225.2 | IVD | ILMN_13293 | -2.66 |
| NM_079837.2 | BANP | ILMN_8638 | -2.67 |
| NM_002568.3 | PABPC1 | ILMN_173094 | -2.67 |
| NM_014596.4 | ZNRD1 | ILMN_20009 | -2.67 |
| NM_000814.4 | GABRB3 | ILMN_19294 | -2.68 |
| NM_018844.2 | BCAP29 | ILMN_24686 | -2.7 |
| NM_001006684.1 | TCEAL8 | ILMN_12677 | -2.7 |
| NM_032320.5 | BTBD10 | ILMN_30066 | -2.71 |
| NM_152360.2 | ZNF573 | ILMN_23003 | -2.72 |
| NM_001012756.1 | ZNF260 | ILMN_172733 | -2.74 |
| NM_016561.1 | BFAR | ILMN_23440 | -2.75 |
| NM_020892.1 | DTX2 | ILMN_21612 | -2.75 |
| XM_942780.2 | SYNPO2 | ILMN_45907 | -2.75 |
| NM_005056.1 | JARID1A | ILMN_12150 | -2.76 |
| NM_001031706.1 | PLEKHB2 | ILMN_179121 | -2.76 |
| NM_018304.2 | PRR11 | ILMN_32619 | -2.76 |
| NM_203284.1 | RBPJ | ILMN_170184 | -2.76 |
| NM_012117.1 | CBX5 | ILMN_25072 | -2.77 |
| NM_016551.1 | TM7SF3 | ILMN_7797 | -2.77 |
| NM_020234.4 | DTWD1 | ILMN_3248 | -2.78 |
| NM_023071.1 | SPATS2 | ILMN_10985 | -2.78 |
| NM_145800.2 | SEPT6 | ILMN_29094 | -2.79 |
| NM_001007278.1 | TRIM13 | ILMN_14225 | -2.79 |
| NM_015888.4 | HOOK1 | ILMN_173692 | -2.81 |
| NM_020147.2 | THAP10 | ILMN_182683 | -2.81 |
| NM_001042549.1 | NSL1 | ILMN_164300 | -2.84 |
| NM_183399.1 | RNF14 | ILMN_7292 | -2.84 |
| NM_001008405.1 | BCAP29 | ILMN_24800 | -2.86 |
| NM_001827.1 | CKS2 | ILMN_14702 | -2.86 |
| NM_000856.3 | GUCY1A3 | ILMN_11680 | -2.86 |
| NM_012234.4 | RYBP | ILMN_13259 | -2.86 |
| NM_001040708.1 | HEY1 | ILMN_164416 | -2.9 |
| NM_138316.2 | PANK1 | ILMN_406 | -2.91 |
| NM_020242.1 | KIF15 | ILMN_6188 | -2.94 |
| NM_004401.2 | DFFA | ILMN_6993 | -2.95 |
| NM_004987.3 | LIMS1 | ILMN_11207 | -2.98 |
| NM_024813.1 | RPAP2 | ILMN_23904 | -2.98 |
| NM_016042.2 | EXOSC3 | ILMN_174330 | -2.99 |
| NM_016071.2 | MRPS33 | ILMN_4243 | -2.99 |
| NM_015599.1 | PGM3 | ILMN_14773 | -2.99 |
| NM_001033503.1 | SAR1B | ILMN_16595 | -3 |
| NM_182919.1 | TICAM1 | ILMN_11434 | -3.03 |
| NM_001007157.1 | PHF14 | ILMN_2096 | -3.04 |
| NM_012460.2 | TIMM9 | ILMN_9968 | -3.07 |
| NM_006145.1 | DNAJB1 | ILMN_19740 | -3.08 |
| NM_201262.1 | DNAJC12 | ILMN_18576 | -3.08 |
| XM_938779.1 | LOC653972 | ILMN_31111 | -3.09 |
| NM_002086.3 | GRB2 | ILMN_173749 | -3.11 |
| NM_001042370.1 | TROVE2 | ILMN_173505 | -3.13 |
| NM_001040285.1 | PAPD5 | ILMN_167231 | -3.14 |
| NM_001166.3 | BIRC2 | ILMN_23760 | -3.22 |
| NM_004982.2 | KCNJ8 | ILMN_29993 | -3.22 |
| NM_015942.3 | MTERFD1 | ILMN_174209 | -3.23 |
| NM_080597.2 | OSBPL1A | ILMN_10951 | -3.23 |
| NM_024749.2 | VASH2 | ILMN_3016 | -3.25 |
| NM_022731.2 | NUCKS1 | ILMN_17108 | -3.26 |
| NM_016277.3 | RAB23 | ILMN_177407 | -3.28 |
| NM_207350.1 | MGC72104 | ILMN_26269 | -3.32 |
| NM_024920.3 | DNAJB14 | ILMN_12080 | -3.33 |
| NM_020749.3 | MTUS1 | ILMN_4658 | -3.35 |
| NM_014865.2 | NCAPD2 | ILMN_26621 | -3.36 |
| NM_181702.1 | GEM | ILMN_16170 | -3.4 |
| NM_012433.2 | SF3B1 | ILMN_168075 | -3.41 |
| XM_926249.2 | LOC642852 | ILMN_40586 | -3.43 |
| NM_003542.3 | HIST1H4C | ILMN_30043 | -3.49 |
| NM_002897.3 | RBMS1 | ILMN_18726 | -3.51 |
| NM_199287.2 | CCDC137 | ILMN_309720 | -3.58 |
| NM_006914.3 | RORB | ILMN_7297 | -3.58 |
| NM_020449.2 | THOC2 | ILMN_162047 | -3.59 |
| NM_017958.1 | PLEKHB2 | ILMN_29704 | -3.6 |
| NM_033091.1 | TRIM4 | ILMN_8530 | -3.62 |
| NM_005909.3 | MAP1B | ILMN_28251 | -3.63 |
| NM_016587.2 | CBX3 | ILMN_11642 | -3.64 |
| NM_203390.2 | RBM12B | ILMN_174962 | -3.66 |
| NM_153333.2 | TCEAL8 | ILMN_12551 | -3.66 |
| NM_004792.2 | PPIG | ILMN_24595 | -3.7 |
| NM_005723.2 | TSPAN5 | ILMN_8032 | -3.71 |
| NM_003368.4 | USP1 | ILMN_5285 | -3.74 |
| NM_032334.1 | C8orf53 | ILMN_24637 | -3.75 |
| NM_001031723.1 | DNAJB14 | ILMN_9854 | -3.77 |
| NR_001445.1 | RN7SK | ILMN_14457 | -3.8 |
| NM_005713.1 | COL4A3BP | ILMN_10635 | -3.85 |
| NM_024011.2 | CDC2L2 | ILMN_20434 | -3.93 |
| NM_019067.4 | GNL3L | ILMN_181682 | -4.03 |
| XM_935818.1 | FLJ20397 | ILMN_137080 | -4.06 |
| NM_001438.2 | ESRRG | ILMN_29221 | -4.09 |
| NM_001039937.1 | INTS6 | ILMN_38649 | -4.13 |
| NM_002093.2 | GSK3B | ILMN_7421 | -4.14 |
| NM_005905.3 | SMAD9 | ILMN_28187 | -4.16 |
| NM_003344.2 | UBE2H | ILMN_163352 | -4.22 |
| NM_001039703.1 | NBPF10 | ILMN_45673 | -4.29 |
| NM_006717.2 | SPIN1 | ILMN_23742 | -4.3 |
| NM_178439.3 | GMCL1 | ILMN_3285 | -4.38 |
| NR_001449.1 | TRK1 | ILMN_6493 | -4.42 |
| NM_184234.1 | RBM39 | ILMN_20330 | -4.51 |
| NM_001040142.1 | SCN2A | ILMN_167124 | -4.6 |
| NM_078629.1 | MSL3L1 | ILMN_29354 | -4.64 |
| NM_004175.3 | SNRPD3 | ILMN_163179 | -4.65 |
| NM_005345.4 | HSPA1A | ILMN_6623 | -4.67 |
| NM_138444.3 | KCTD12 | ILMN_18501 | -4.67 |
| NM_004456.3 | EZH2 | ILMN_25740 | -4.74 |
| NM_004257.3 | TGFBRAP1 | ILMN_30176 | -4.74 |
| NM_006182.2 | DDR2 | ILMN_20698 | -4.75 |
| NM_016618.1 | KRCC1 | ILMN_25337 | -4.78 |
| NM_002763.3 | PROX1 | ILMN_177185 | -4.83 |
| XM_001129423.1 | LOC729137 | ILMN_166772 | -4.96 |
| NM_003358.1 | UGCG | ILMN_26228 | -4.97 |
| NM_178439.3 | GMCL1 | ILMN_3285 | -5.14 |
| NM_006265.1 | RAD21 | ILMN_171453 | -5.15 |
| NM_016374.5 | ARID4B | ILMN_162934 | -5.24 |
| NM_014498.2 | GOLPH4 | ILMN_179486 | -5.38 |
| NM_006630.1 | ZNF234 | ILMN_29233 | -5.44 |
| NM_001037675.1 | NBPF20 | ILMN_26956 | -5.48 |
| NM_001034841.2 | LOC162073 | ILMN_3559 | -5.74 |
| NR_003041.1 | SNORD13 | ILMN_168446 | -5.92 |
| NM_005346.3 | HSPA1B | ILMN_25549 | -5.99 |
| NM_016374.5 | ARID4B | ILMN_162934 | -6.25 |
