# Supplementary material for: Minimal Peroxide Exposure of Neuronal Cells Induces Multifaceted Adaptive Responses
Source: PLoS One. 2010 Dec 17;5(12):e14352. doi: 10.1371/journal.pone.0014352 (PMC3003681; doi:10.1371/journal.pone.0014352)
Supplement: Table S13 — BDNF-significantly regulated genes after 4 hours of stimulation in the CMP state SH-SY5Y cells. Each significantly regulated gene is described via its accession number (ACCESSION), Gene Symbol (SYMBOL), Illumina array transcript designation (TRANSCRIPT). For each gene the z-ratio of expression compared to untreated cells after 4 hours of ligand stimulation is displayed (CMP BDNF 4). (1.09 MB DOC) [file pone.0014352.s020.doc]

**Table S13 BDNF-significantly regulated genes after 4 hours of stimulation in the CMP state SH-SY5Y cells**. Each significantly regulated gene is described via its accession number (ACCESSION), Gene Symbol (SYMBOL), Illumina array transcript designation (TRANSCRIPT). For each gene the z-ratio of expression compared to un-treated cells after 4 hours of ligand stimulation is displayed (CMP BDNF 4).

| **ACCESSION** | **SYMBOL** | **TRANSCRIPT** | **CMP BDNF 4** |
| --- | --- | --- | --- |
| NM_000584.2 | IL8 | ILMN_179575 | 6.7 |
| XM_944439.2 | LOC653994 | ILMN_38572 | 5.19 |
| NM_005324.3 | H3F3B | ILMN_26885 | 4.85 |
| NM_016028.4 | SUV420H1 | ILMN_29861 | 4.83 |
| NM_014817.3 | KIAA0644 | ILMN_164846 | 4.77 |
| NM_148957.2 | TNFRSF19 | ILMN_28684 | 4.73 |
| NM_001033506.1 | CSTF3 | ILMN_27049 | 4.68 |
| NM_130435.2 | PTPRE | ILMN_23422 | 4.3 |
| NM_018697.3 | LANCL2 | ILMN_920 | 4.13 |
| NM_080797.2 | DIDO1 | ILMN_17046 | 4.09 |
| NM_002673.3 | PLXNB1 | ILMN_22628 | 3.97 |
| NM_001008219.1 | AMY1C | ILMN_28222 | 3.94 |
| NM_000617.1 | SLC11A2 | ILMN_10129 | 3.69 |
| NM_020724.1 | RNF150 | ILMN_26801 | 3.59 |
| NM_002160.2 | TNC | ILMN_14948 | 3.53 |
| NM_012215.2 | MGEA5 | ILMN_11399 | 3.51 |
| XM_001134215.1 | PDPR | ILMN_162295 | 3.47 |
| NM_152322.2 | BTBD11 | ILMN_506 | 3.44 |
| NM_002566.4 | P2RY11 | ILMN_12237 | 3.39 |
| NM_001040456.1 | RHBDD2 | ILMN_168345 | 3.38 |
| NM_153188.2 | TNPO1 | ILMN_29083 | 3.32 |
| NM_006997.2 | TACC2 | ILMN_16130 | 3.3 |
| NM_173042.2 | IL18BP | ILMN_30884 | 3.24 |
| NM_001012626.1 | LOC285074 | ILMN_21153 | 3.24 |
| NM_001001391.1 | CD44 | ILMN_10947 | 3.24 |
| NM_003972.2 | BTAF1 | ILMN_8616 | 3.2 |
| NM_024663.3 | NPEPL1 | ILMN_175218 | 3.17 |
| NM_003110.4 | SP2 | ILMN_7882 | 3.14 |
| NM_020162.2 | DHX33 | ILMN_1191 | 3.13 |
| NM_001008408.3 | RBM33 | ILMN_165407 | 3.11 |
| NM_201557.2 | FHL2 | ILMN_42988 | 3.04 |
| NM_080491.1 | GAB2 | ILMN_3317 | 3.03 |
| NM_004071.2 | CLK1 | ILMN_162592 | 3.02 |
| NM_001080485.1 | ZNF275 | ILMN_180340 | 3.01 |
| NM_133471.1 | KIAA1949 | ILMN_308966 | 2.92 |
| NM_000199.2 | SGSH | ILMN_7542 | 2.92 |
| NM_001077188.1 | HS6ST2 | ILMN_182242 | 2.91 |
| NM_016481.3 | C9orf156 | ILMN_12842 | 2.9 |
| NM_002874.3 | RAD23B | ILMN_19346 | 2.89 |
| NM_170600.1 | SH2D3C | ILMN_8703 | 2.89 |
| NM_182492.1 | LRP5L | ILMN_650 | 2.87 |
| NM_014000.2 | VCL | ILMN_27566 | 2.87 |
| NM_017757.2 | ZNF407 | ILMN_12747 | 2.83 |
| NM_032199.1 | ARID5B | ILMN_165822 | 2.83 |
| NM_138967.2 | SCAMP5 | ILMN_3255 | 2.81 |
| NM_020796.3 | SEMA6A | ILMN_11282 | 2.8 |
| NM_014014.2 | ASCC3L1 | ILMN_18834 | 2.8 |
| NM_033446.1 | FAM125B | ILMN_20760 | 2.79 |
| NM_015655.2 | ZNF337 | ILMN_3280 | 2.79 |
| NM_015447.1 | CAMSAP1 | ILMN_815 | 2.76 |
| NM_024735.2 | FBXO31 | ILMN_17806 | 2.75 |
| NM_005342.2 | HMGB3 | ILMN_8326 | 2.73 |
| NM_003086.2 | SNAPC4 | ILMN_180505 | 2.71 |
| NM_015906.3 | TRIM33 | ILMN_4131 | 2.71 |
| NM_015306.1 | USP24 | ILMN_309418 | 2.69 |
| NM_002213.3 | ITGB5 | ILMN_24189 | 2.66 |
| NM_006465.2 | ARID3B | ILMN_4032 | 2.65 |
| NM_001206.2 | KLF9 | ILMN_169601 | 2.64 |
| NM_024077.3 | SECISBP2 | ILMN_19156 | 2.64 |
| NM_001080453.1 | INTS1 | ILMN_173681 | 2.64 |
| NM_005189.1 | CBX2 | ILMN_28525 | 2.64 |
| NM_033419.3 | PERLD1 | ILMN_12215 | 2.63 |
| NM_014717.1 | ZNF536 | ILMN_179125 | 2.63 |
| NM_015352.1 | POFUT1 | ILMN_7876 | 2.6 |
| NM_012256.2 | ZNF212 | ILMN_14026 | 2.59 |
| NM_013276.2 | SHPK | ILMN_22706 | 2.58 |
| NM_015477.1 | SIN3A | ILMN_14108 | 2.58 |
| NM_004598.3 | SPOCK1 | ILMN_25886 | 2.57 |
| NM_006045.1 | ATP9A | ILMN_176431 | 2.57 |
| NM_002473.3 | MYH9 | ILMN_183555 | 2.56 |
| XM_926036.1 | LOC653103 | ILMN_32029 | 2.53 |
| NM_030806.3 | C1orf21 | ILMN_26434 | 2.53 |
| NM_004075.2 | CRY1 | ILMN_6263 | 2.52 |
| NM_032195.1 | SON | ILMN_8462 | 2.51 |
| NM_001077442.1 | HNRNPC | ILMN_165238 | 2.51 |
| NM_017635.3 | SUV420H1 | ILMN_174505 | 2.51 |
| NM_203499.1 | DDX42 | ILMN_1880 | 2.51 |
| NM_001040456.1 | RHBDD2 | ILMN_168345 | 2.5 |
| NM_003819.2 | PABPC4 | ILMN_18446 | 2.5 |
| XM_001134215.1 | PDPR | ILMN_162295 | 2.5 |
| NM_001440.2 | EXTL3 | ILMN_10725 | 2.49 |
| NM_005385.3 | NKTR | ILMN_23378 | 2.48 |
| NM_003622.2 | PPFIBP1 | ILMN_172147 | 2.47 |
| NM_004454.1 | ETV5 | ILMN_12676 | 2.47 |
| NM_016021.2 | UBE2J1 | ILMN_164177 | 2.46 |
| NM_015338.4 | ASXL1 | ILMN_183479 | 2.46 |
| NM_000292.1 | PHKA2 | ILMN_20799 | 2.46 |
| NM_001144.4 | AMFR | ILMN_22219 | 2.45 |
| NM_001300.4 | KLF6 | ILMN_17961 | 2.45 |
| NM_007005.3 | TLE4 | ILMN_14046 | 2.45 |
| NM_021090.3 | MTMR3 | ILMN_27578 | 2.45 |
| NM_020859.1 | SHRM | ILMN_16821 | 2.44 |
| XM_495939.3 | KIAA1545 | ILMN_40920 | 2.42 |
| NM_003458.3 | BSN | ILMN_22754 | 2.42 |
| NM_198267.1 | ING3 | ILMN_23155 | 2.41 |
| NM_022450.2 | RHBDF1 | ILMN_20892 | 2.4 |
| NM_002938.2 | RNF4 | ILMN_176496 | 2.39 |
| NM_032918.1 | RERG | ILMN_12434 | 2.39 |
| XM_925839.1 | LOC158301 | ILMN_38075 | 2.39 |
| NM_004671.2 | PIAS2 | ILMN_11308 | 2.38 |
| NM_201555.1 | FHL2 | ILMN_21541 | 2.38 |
| NM_003667.2 | LGR5 | ILMN_20455 | 2.37 |
| NM_199043.1 | C14orf102 | ILMN_22442 | 2.37 |
| NM_014330.2 | PPP1R15A | ILMN_1024 | 2.37 |
| NM_033631.2 | LUZP1 | ILMN_2667 | 2.36 |
| NM_014747.2 | RIMS3 | ILMN_21581 | 2.35 |
| NM_003131.2 | SRF | ILMN_22299 | 2.34 |
| NM_003565.1 | ULK1 | ILMN_2158 | 2.34 |
| NM_006159.1 | NELL2 | ILMN_26383 | 2.34 |
| NM_006739.3 | MCM5 | ILMN_20107 | 2.33 |
| NM_001031623.2 | ZNF451 | ILMN_990 | 2.33 |
| NM_003966.2 | SEMA5A | ILMN_183828 | 2.32 |
| NM_031954.3 | KCTD10 | ILMN_30217 | 2.32 |
| NM_018948.2 | ERRFI1 | ILMN_4328 | 2.32 |
| NM_170695.2 | TGIF1 | ILMN_162784 | 2.31 |
| NM_018202.3 | TMEM57 | ILMN_30191 | 2.31 |
| XM_379215.2 | LOC132241 | ILMN_37830 | 2.31 |
| NM_014694.2 | ADAMTSL2 | ILMN_697 | 2.31 |
| XM_001127981.1 | LOC728014 | ILMN_169164 | 2.3 |
| NM_006011.3 | ST8SIA2 | ILMN_19287 | 2.3 |
| NM_002972.1 | SBF1 | ILMN_22729 | 2.3 |
| NM_017921.1 | NPLOC4 | ILMN_12904 | 2.3 |
| NM_002959.4 | SORT1 | ILMN_165748 | 2.3 |
| NM_022781.4 | RNF38 | ILMN_40416 | 2.3 |
| NM_002482.2 | NASP | ILMN_21654 | 2.3 |
| NM_013313.3 | YPEL1 | ILMN_26647 | 2.3 |
| NM_181784.1 | SPRED2 | ILMN_12131 | 2.29 |
| XM_001126418.1 | LOC727935 | ILMN_181411 | 2.28 |
| NM_170721.1 | MSI2 | ILMN_25750 | 2.27 |
| NM_003461.4 | ZYX | ILMN_2137 | 2.27 |
| NM_020414.3 | DDX24 | ILMN_10146 | 2.26 |
| NM_002604.1 | PDE7A | ILMN_3430 | 2.26 |
| NM_014452.3 | TNFRSF21 | ILMN_9651 | 2.24 |
| NM_001013685.1 | LOC401357 | ILMN_29013 | 2.23 |
| NM_001013258.1 | ZNF789 | ILMN_11535 | 2.23 |
| NM_006372.3 | SYNCRIP | ILMN_28470 | 2.23 |
| NM_003565.1 | ULK1 | ILMN_2158 | 2.23 |
| NM_003119.2 | SPG7 | ILMN_26332 | 2.23 |
| NM_001020658.1 | PUM1 | ILMN_169279 | 2.23 |
| NM_022748.10 | TNS3 | ILMN_17676 | 2.22 |
| NM_152424.1 | FLJ39827 | ILMN_19358 | 2.22 |
| NM_178831.4 | GATS | ILMN_18755 | 2.22 |
| NM_003799.1 | RNMT | ILMN_23400 | 2.21 |
| NM_024612.3 | DHX40 | ILMN_1864 | 2.21 |
| NM_006715.2 | MAN2C1 | ILMN_685 | 2.2 |
| NM_153451.2 | ORAOV1 | ILMN_5733 | 2.19 |
| NM_144781.1 | PDCD2 | ILMN_16269 | 2.19 |
| NM_024656.2 | GLT25D1 | ILMN_15022 | 2.19 |
| NM_031469.2 | SH3BGRL2 | ILMN_9801 | 2.19 |
| NM_152280.2 | SYT11 | ILMN_23967 | 2.19 |
| NM_004330.1 | BNIP2 | ILMN_9985 | 2.19 |
| NM_023080.1 | C8orf33 | ILMN_15901 | 2.18 |
| NR_002809.1 | LOC338799 | ILMN_15606 | 2.18 |
| NM_022910.1 | NDRG4 | ILMN_8824 | 2.18 |
| NM_032520.3 | GNPTG | ILMN_28173 | 2.17 |
| NM_005475.1 | SH2B3 | ILMN_5130 | 2.17 |
| NM_012463.2 | ATP6V0A2 | ILMN_23163 | 2.17 |
| NM_001677.3 | ATP1B1 | ILMN_25542 | 2.17 |
| NM_014141.4 | CNTNAP2 | ILMN_176606 | 2.16 |
| NM_001048201.1 | UHRF1 | ILMN_162952 | 2.16 |
| NM_014838.2 | ZBED4 | ILMN_8641 | 2.14 |
| NM_003045.3 | SLC7A1 | ILMN_162673 | 2.14 |
| NM_172358.1 | CD46 | ILMN_4413 | 2.14 |
| NM_001287.3 | CLCN7 | ILMN_8600 | 2.13 |
| NM_201440.1 | PPHLN1 | ILMN_4445 | 2.13 |
| NM_006773.3 | DDX18 | ILMN_22238 | 2.13 |
| NM_014712.1 | SETD1A | ILMN_24764 | 2.13 |
| NR_003491.1 | MIAT | ILMN_308315 | 2.13 |
| NM_016143.3 | NSFL1C | ILMN_20493 | 2.12 |
| NM_015711.2 | GLTSCR1 | ILMN_18273 | 2.12 |
| NM_033389.2 | SSH2 | ILMN_8279 | 2.12 |
| XM_930411.1 | LOC645099 | ILMN_37678 | 2.12 |
| NM_006197.3 | PCM1 | ILMN_14472 | 2.12 |
| NM_015077.2 | SARM1 | ILMN_23861 | 2.11 |
| NM_145687.2 | MAP4K4 | ILMN_28871 | 2.11 |
| NM_014268.1 | MAPRE2 | ILMN_8637 | 2.11 |
| NM_014947.3 | FOXJ3 | ILMN_26064 | 2.11 |
| XM_942424.2 | LOC440589 | ILMN_39348 | 2.11 |
| NM_018708.2 | FEM1A | ILMN_2838 | 2.1 |
| NM_001002878.1 | THOC5 | ILMN_13820 | 2.1 |
| NM_025250.2 | TTYH3 | ILMN_22026 | 2.1 |
| NM_005667.2 | RNF103 | ILMN_17861 | 2.1 |
| XM_497029.2 | LOC441408 | ILMN_31941 | 2.09 |
| NM_138440.2 | VASN | ILMN_31069 | 2.09 |
| NM_203401.1 | STMN1 | ILMN_12586 | 2.09 |
| NM_006275.4 | SFRS6 | ILMN_24964 | 2.09 |
| NM_013450.2 | BAZ2B | ILMN_20026 | 2.09 |
| NM_003342.4 | UBE2G1 | ILMN_179729 | 2.07 |
| NM_020822.1 | KCNT1 | ILMN_21599 | 2.06 |
| NM_022720.5 | DGCR8 | ILMN_1552 | 2.06 |
| NM_006918.4 | SC5DL | ILMN_24287 | 2.06 |
| NM_175854.5 | PAN3 | ILMN_181888 | 2.06 |
| NM_206826.1 | GNL3 | ILMN_10491 | 2.06 |
| NM_024909.1 | C6orf134 | ILMN_21139 | 2.05 |
| NM_005245.3 | FAT | ILMN_24617 | 2.05 |
| NM_004091.2 | E2F2 | ILMN_19730 | 2.05 |
| NM_005819.4 | STX6 | ILMN_180926 | 2.05 |
| NM_018257.1 | PCMTD2 | ILMN_4945 | 2.05 |
| NM_003183.4 | ADAM17 | ILMN_165100 | 2.05 |
| NM_016284.3 | CNOT1 | ILMN_169268 | 2.05 |
| NM_015124.2 | GRAMD4 | ILMN_12136 | 2.04 |
| NM_004566.2 | PFKFB3 | ILMN_163833 | 2.04 |
| NM_005157.3 | ABL1 | ILMN_4033 | 2.04 |
| NM_152834.2 | TMEM18 | ILMN_8053 | 2.04 |
| NM_012401.2 | PLXNB2 | ILMN_308861 | 2.04 |
| NM_002687.3 | PNN | ILMN_24088 | 2.04 |
| NM_198925.1 | SEMA4B | ILMN_25258 | 2.03 |
| NM_025135.2 | FHOD3 | ILMN_163420 | 2.03 |
| NM_006618.3 | JARID1B | ILMN_14812 | 2.03 |
| NM_015560.1 | OPA1 | ILMN_10977 | 2.02 |
| NM_004687.3 | MTMR4 | ILMN_163329 | 2.02 |
| NM_152557.3 | ZNF746 | ILMN_25894 | 2.01 |
| NM_003012.3 | SFRP1 | ILMN_21487 | 2.01 |
| NM_201553.1 | FGL1 | ILMN_2104 | 2 |
| NM_017456.1 | PSCD1 | ILMN_16992 | 2 |
| NM_015144.2 | ZCCHC14 | ILMN_32176 | 2 |
| NM_133496.3 | SLC30A7 | ILMN_20389 | 2 |
| NM_182687.1 | PKMYT1 | ILMN_1154 | 2 |
| NM_006749.3 | SLC20A2 | ILMN_29659 | 1.99 |
| NM_003804.3 | RIPK1 | ILMN_24351 | 1.99 |
| NM_003913.3 | PRPF4B | ILMN_139391 | 1.99 |
| NM_001037639.1 | PARL | ILMN_13356 | 1.99 |
| NM_018482.2 | DDEF1 | ILMN_184045 | 1.99 |
| NM_182776.1 | MCM7 | ILMN_1133 | 1.98 |
| NM_002938.2 | RNF4 | ILMN_26467 | 1.98 |
| NM_015153.1 | PHF3 | ILMN_23658 | 1.98 |
| NM_024525.2 | TTC13 | ILMN_164005 | 1.97 |
| XM_931434.2 | LOC400027 | ILMN_35789 | 1.97 |
| NM_152562.2 | CDCA2 | ILMN_19331 | 1.96 |
| NM_001481.1 | GAS8 | ILMN_26809 | 1.95 |
| NM_020933.2 | ZNF317 | ILMN_22884 | 1.95 |
| NM_033020.2 | TRIM33 | ILMN_3792 | 1.95 |
| NM_005922.2 | MAP3K4 | ILMN_6743 | 1.95 |
| NM_198679.1 | RAPGEF1 | ILMN_177243 | 1.95 |
| NM_024653.3 | PRKRIP1 | ILMN_13077 | 1.95 |
| NM_017719.3 | SNRK | ILMN_5234 | 1.95 |
| NM_001007246.1 | BRWD1 | ILMN_28841 | 1.95 |
| NM_020770.1 | CGN | ILMN_22926 | 1.94 |
| NM_013243.2 | SCG3 | ILMN_174345 | 1.94 |
| NM_080670.2 | SLC35A4 | ILMN_8862 | 1.94 |
| NM_015995.2 | KLF13 | ILMN_16226 | 1.94 |
| NM_004064.2 | CDKN1B | ILMN_175665 | 1.94 |
| NM_001642.1 | APLP2 | ILMN_19935 | 1.94 |
| NM_001023587.1 | ABCC5 | ILMN_438 | 1.94 |
| NM_033285.2 | TP53INP1 | ILMN_16203 | 1.94 |
| XM_497182.3 | LOC644670 | ILMN_38310 | 1.94 |
| NM_201281.1 | MTMR2 | ILMN_24002 | 1.93 |
| NM_005688.2 | ABCC5 | ILMN_25223 | 1.93 |
| NM_006660.3 | CLPX | ILMN_28281 | 1.93 |
| XM_940209.1 | KIAA0194 | ILMN_37512 | 1.93 |
| NM_018254.2 | RCOR3 | ILMN_15381 | 1.93 |
| NM_015330.1 | SPECC1L | ILMN_168707 | 1.93 |
| NM_001003795.2 | GTF2IRD2B | ILMN_12811 | 1.92 |
| NM_019591.2 | ZNF26 | ILMN_3233 | 1.92 |
| NM_003486.5 | SLC7A5 | ILMN_25446 | 1.92 |
| NM_015346.2 | ZFYVE26 | ILMN_176163 | 1.92 |
| NM_201559.2 | FOXO3 | ILMN_15525 | 1.92 |
| NM_001496.3 | GFRA3 | ILMN_8392 | 1.91 |
| NM_006925.3 | SFRS5 | ILMN_34497 | 1.91 |
| NM_002473.3 | MYH9 | ILMN_183555 | 1.91 |
| NM_001068.2 | TOP2B | ILMN_7099 | 1.91 |
| NM_001033505.1 | CSTF3 | ILMN_26942 | 1.9 |
| NM_206852.1 | RTN1 | ILMN_3435 | 1.9 |
| NM_014614.1 | PSME4 | ILMN_164803 | 1.9 |
| NM_006141.2 | DYNC1LI2 | ILMN_183290 | 1.9 |
| NM_001093756.1 | FLJ13611 | ILMN_308292 | 1.89 |
| NM_018566.3 | YOD1 | ILMN_19081 | 1.89 |
| NM_024648.1 | FLJ22222 | ILMN_21603 | 1.89 |
| XM_001133202.1 | KIAA0363 | ILMN_166209 | 1.89 |
| NM_006123.2 | IDS | ILMN_17605 | 1.88 |
| NM_001081559.1 | CPSF4 | ILMN_178236 | 1.88 |
| NM_003275.2 | TMOD1 | ILMN_1052 | 1.88 |
| NM_004516.2 | ILF3 | ILMN_12252 | 1.88 |
| NM_025133.3 | FBXO11 | ILMN_18553 | 1.88 |
| NM_000787.3 | DBH | ILMN_25962 | 1.88 |
| NM_207331.2 | LOC153561 | ILMN_1879 | 1.88 |
| NM_152834.2 | TMEM18 | ILMN_8053 | 1.87 |
| XM_928464.1 | LOC146517 | ILMN_32888 | 1.87 |
| NM_001031685.2 | TP53BP2 | ILMN_9205 | 1.87 |
| NM_024900.3 | PHF17 | ILMN_1535 | 1.87 |
| NM_018416.2 | FOXJ2 | ILMN_165896 | 1.87 |
| NM_030621.2 | DICER1 | ILMN_1996 | 1.87 |
| NM_032776.1 | JMJD1C | ILMN_164120 | 1.87 |
| NM_014765.1 | TOMM20 | ILMN_20433 | 1.87 |
| NM_004467.3 | FGL1 | ILMN_25289 | 1.86 |
| NM_004393.2 | DAG1 | ILMN_16432 | 1.86 |
| NM_017644.3 | KLHL24 | ILMN_26914 | 1.86 |
| NM_015516.3 | TSKU | ILMN_29523 | 1.85 |
| NM_004090.2 | DUSP3 | ILMN_180655 | 1.85 |
| NM_005808.2 | CTDSPL | ILMN_510 | 1.85 |
| NM_199482.1 | PREI3 | ILMN_10571 | 1.85 |
| NM_152282.2 | ACPL2 | ILMN_138667 | 1.84 |
| XM_942544.2 | INTS1 | ILMN_38896 | 1.84 |
| NM_016644.1 | PRR16 | ILMN_4368 | 1.84 |
| NM_001031712.2 | TRMT11 | ILMN_8801 | 1.84 |
| NM_025195.2 | TRIB1 | ILMN_29203 | 1.84 |
| XM_290799.7 | ARHGAP23 | ILMN_162296 | 1.83 |
| NM_021931.2 | DHX35 | ILMN_18259 | 1.83 |
| XM_928905.2 | LOC645937 | ILMN_41912 | 1.82 |
| NM_052899.2 | GPRIN1 | ILMN_15887 | 1.82 |
| NM_002764.2 | PRPS1 | ILMN_161881 | 1.82 |
| NM_002333.1 | LRP3 | ILMN_12327 | 1.82 |
| NM_001294.1 | CLPTM1 | ILMN_22488 | 1.82 |
| NM_032242.2 | PLXNA1 | ILMN_912 | 1.82 |
| NM_006083.3 | IK | ILMN_27338 | 1.82 |
| NM_015720.1 | PODXL2 | ILMN_26815 | 1.82 |
| NM_001357.2 | DHX9 | ILMN_7196 | 1.81 |
| NM_001003795.2 | GTF2IRD2B | ILMN_12811 | 1.81 |
| NM_002374.3 | MAP2 | ILMN_38764 | 1.81 |
| NM_002915.3 | RFC3 | ILMN_11616 | 1.81 |
| NM_000391.3 | TPP1 | ILMN_19883 | 1.81 |
| NM_080702.2 | BAT3 | ILMN_4429 | 1.81 |
| NM_020651.2 | PELI1 | ILMN_11771 | 1.81 |
| NM_006985.1 | NPIP | ILMN_10143 | 1.81 |
| NM_152716.1 | PATL1 | ILMN_11588 | 1.81 |
| NM_030665.3 | RAI1 | ILMN_176671 | 1.8 |
| NM_174941.3 | M160 | ILMN_137163 | 1.8 |
| NM_017544.2 | NKRF | ILMN_20013 | 1.8 |
| NM_015433.2 | FAM119B | ILMN_17350 | 1.8 |
| NM_005386.2 | NNAT | ILMN_16198 | 1.8 |
| NM_057159.2 | LPAR1 | ILMN_28278 | 1.8 |
| NM_018029.3 | FLJ10213 | ILMN_29061 | 1.79 |
| NM_014264.3 | PLK4 | ILMN_167207 | 1.79 |
| NM_153812.1 | PHF13 | ILMN_27355 | 1.79 |
| NM_005112.4 | WDR1 | ILMN_14401 | 1.79 |
| NM_018031.2 | WDR6 | ILMN_16845 | 1.79 |
| NM_004192.1 | ASMTL | ILMN_15304 | 1.78 |
| NM_032239.2 | LARP2 | ILMN_9962 | 1.78 |
| NM_001008237.1 | TTC32 | ILMN_4829 | 1.77 |
| NM_018249.4 | CDK5RAP2 | ILMN_9876 | 1.77 |
| NM_003434.3 | ZNF133 | ILMN_5864 | 1.77 |
| NM_199245.1 | VAMP1 | ILMN_10901 | 1.77 |
| NM_018263.4 | ASXL2 | ILMN_7971 | 1.77 |
| NM_005920.2 | MEF2D | ILMN_3465 | 1.76 |
| XM_001127981.1 | LOC728014 | ILMN_169164 | 1.76 |
| NM_145287.2 | ZNF519 | ILMN_27951 | 1.76 |
| NM_021643.1 | TRIB2 | ILMN_163335 | 1.75 |
| NM_005911.4 | MAT2A | ILMN_25630 | 1.75 |
| NM_015085.3 | GARNL4 | ILMN_163593 | 1.75 |
| NM_078470.2 | COX15 | ILMN_13504 | 1.74 |
| NM_030941.1 | LOC81691 | ILMN_17520 | 1.74 |
| NM_014708.3 | KNTC1 | ILMN_25890 | 1.74 |
| XR_019152.1 | LOC644584 | ILMN_163817 | 1.73 |
| NM_014772.1 | KIAA0427 | ILMN_182540 | 1.73 |
| NM_031844.2 | HNRNPU | ILMN_3074 | 1.73 |
| NM_001006657.1 | WDR35 | ILMN_175554 | 1.73 |
| NM_003846.1 | PEX11B | ILMN_20603 | 1.73 |
| NM_006885.3 | ZFHX3 | ILMN_174159 | 1.73 |
| NM_153321.1 | PMP22 | ILMN_9212 | 1.72 |
| NM_021253.2 | TRIM39 | ILMN_165050 | 1.72 |
| NM_001039675.1 | UNC45A | ILMN_40255 | 1.72 |
| NM_016513.3 | ICK | ILMN_23886 | 1.72 |
| NM_198489.1 | CCDC84 | ILMN_6803 | 1.72 |
| NM_005243.2 | EWSR1 | ILMN_17011 | 1.72 |
| NM_006421.3 | ARFGEF1 | ILMN_164295 | 1.72 |
| NM_139353.1 | TAF1C | ILMN_4122 | 1.72 |
| NM_020664.3 | DECR2 | ILMN_7935 | 1.71 |
| NM_005334.2 | HCFC1 | ILMN_24237 | 1.71 |
| NM_017896.2 | C20orf11 | ILMN_27220 | 1.71 |
| NM_004634.2 | BRPF1 | ILMN_17537 | 1.71 |
| NM_015308.1 | FNBP4 | ILMN_25895 | 1.71 |
| NM_000153.2 | GALC | ILMN_28156 | 1.71 |
| NM_006283.1 | TACC1 | ILMN_20678 | 1.7 |
| NM_032776.1 | JMJD1C | ILMN_164120 | 1.7 |
| NM_004526.2 | MCM2 | ILMN_183916 | 1.7 |
| NM_015878.4 | AZIN1 | ILMN_4825 | 1.7 |
| NM_058172.3 | ANTXR2 | ILMN_165233 | 1.7 |
| NM_022459.4 | XPO4 | ILMN_164187 | 1.69 |
| NM_001669.2 | ARSD | ILMN_165491 | 1.69 |
| NM_133259.2 | LRPPRC | ILMN_23753 | 1.69 |
| NM_000479.2 | AMH | ILMN_171371 | 1.69 |
| NM_021737.1 | CLCN6 | ILMN_6195 | 1.68 |
| NM_014862.3 | ARNT2 | ILMN_13881 | 1.68 |
| NM_001034.1 | RRM2 | ILMN_18637 | 1.68 |
| NM_001078.2 | VCAM1 | ILMN_3875 | 1.68 |
| NM_001065.2 | TNFRSF1A | ILMN_173343 | 1.67 |
| NM_017566.2 | KLHDC4 | ILMN_8527 | 1.67 |
| NM_006052.1 | DSCR3 | ILMN_19785 | 1.67 |
| NM_001003805.1 | ATP5S | ILMN_1363 | 1.67 |
| NM_153273.3 | IHPK1 | ILMN_1661 | 1.67 |
| NM_005779.1 | LHFPL2 | ILMN_26106 | 1.67 |
| NM_007182.4 | RASSF1 | ILMN_8297 | 1.66 |
| NM_138930.2 | DIABLO | ILMN_19433 | 1.66 |
| NM_014729.2 | TOX | ILMN_16587 | 1.66 |
| NM_005207.2 | CRKL | ILMN_165503 | 1.66 |
| NM_139235.3 | NOL6 | ILMN_7349 | 1.66 |
| XR_018848.1 | LOC650369 | ILMN_169499 | 1.66 |
| NM_173797.2 | PAPD4 | ILMN_2190 | 1.66 |
| NM_001099270.1 | ZBTB34 | ILMN_307315 | 1.65 |
| NM_024581.4 | C6orf60 | ILMN_1091 | 1.65 |
| NM_005243.2 | EWSR1 | ILMN_17011 | 1.65 |
| NM_004263.3 | SEMA4F | ILMN_177045 | 1.65 |
| NM_005563.3 | STMN1 | ILMN_11775 | 1.65 |
| NM_001002878.1 | THOC5 | ILMN_13820 | 1.65 |
| NM_006925.3 | SFRS5 | ILMN_34497 | 1.65 |
| NM_005778.1 | RBM5 | ILMN_15629 | 1.65 |
| NM_020728.1 | FAM62B | ILMN_19173 | 1.65 |
| NR_003659.1 | FAM39DP | ILMN_307683 | 1.65 |
| NM_080927.3 | DCBLD2 | ILMN_175741 | 1.65 |
| NM_002915.3 | RFC3 | ILMN_11616 | 1.65 |
| XM_945571.1 | ANKRD13D | ILMN_138370 | 1.64 |
| NM_032486.2 | DCTN5 | ILMN_22431 | 1.64 |
| NM_005561.2 | LAMP1 | ILMN_27826 | 1.64 |
| NM_021070.2 | LTBP3 | ILMN_918 | 1.63 |
| NM_002771.2 | PRSS3 | ILMN_19426 | 1.63 |
| NM_177401.4 | MIDN | ILMN_6472 | 1.63 |
| NM_002076.2 | GNS | ILMN_177670 | 1.63 |
| NM_032590.3 | FBXL10 | ILMN_19365 | 1.63 |
| NM_052901.2 | SLC25A25 | ILMN_15060 | 1.62 |
| NM_004311.2 | ARL3 | ILMN_15691 | 1.62 |
| NM_182565.2 | FAM100B | ILMN_22874 | 1.62 |
| NM_006157.2 | NELL1 | ILMN_2560 | 1.62 |
| NM_000202.3 | IDS | ILMN_2023 | 1.62 |
| NM_005010.3 | NRCAM | ILMN_8955 | 1.62 |
| NM_004423.3 | DVL3 | ILMN_11726 | 1.62 |
| NM_024319.2 | C1orf35 | ILMN_28904 | 1.61 |
| NM_017741.3 | C4orf30 | ILMN_172318 | 1.61 |
| NM_199169.1 | PMEPA1 | ILMN_13834 | 1.61 |
| NM_177972.1 | TUB | ILMN_11520 | 1.61 |
| NM_004424.3 | E4F1 | ILMN_23848 | 1.61 |
| NM_006064.3 | RRAGB | ILMN_28228 | 1.61 |
| NM_016333.2 | SRRM2 | ILMN_21088 | 1.61 |
| NM_002035.1 | FVT1 | ILMN_5671 | 1.6 |
| NM_138774.2 | C19orf22 | ILMN_15785 | 1.6 |
| NM_014023.3 | WDR37 | ILMN_175566 | 1.6 |
| NM_145701.1 | CDCA4 | ILMN_5601 | 1.6 |
| NM_001093771.1 | TXNRD1 | ILMN_306750 | 1.6 |
| NM_014746.2 | RNF144 | ILMN_15740 | 1.6 |
| NM_145687.2 | MAP4K4 | ILMN_28871 | 1.6 |
| NM_003118.2 | SPARC | ILMN_1780 | 1.6 |
| NM_001356.3 | DDX3X | ILMN_183040 | 1.6 |
| NM_173515.2 | CNKSR3 | ILMN_25628 | 1.6 |
| NM_006009.2 | TUBA1A | ILMN_1089 | 1.6 |
| NM_031845.2 | MAP2 | ILMN_38825 | 1.59 |
| NM_001009937.1 | SLC25A26 | ILMN_15004 | 1.59 |
| NM_013336.3 | SEC61A1 | ILMN_9397 | 1.59 |
| NM_014727.1 | MLL4 | ILMN_28047 | 1.59 |
| NM_152570.1 | LINGO2 | ILMN_24238 | 1.59 |
| NM_019106.4 | SEPT3 | ILMN_4065 | 1.59 |
| NM_001032289.1 | SLC35A2 | ILMN_20544 | 1.59 |
| NM_015690.2 | STK36 | ILMN_15506 | 1.59 |
| NM_020941.1 | KIAA1602 | ILMN_309224 | 1.59 |
| NM_144566.1 | ZNF700 | ILMN_7926 | 1.59 |
| NM_001017980.2 | LOC203547 | ILMN_163926 | 1.59 |
| NM_004396.2 | DDX5 | ILMN_20253 | 1.59 |
| NM_015180.4 | SYNE2 | ILMN_183979 | 1.58 |
| NM_020310.2 | MNT | ILMN_21283 | 1.58 |
| NM_002657.2 | PLAGL2 | ILMN_23071 | 1.58 |
| NM_023080.1 | C8orf33 | ILMN_15901 | 1.58 |
| NM_182483.1 | NSFL1C | ILMN_25808 | 1.58 |
| NM_012257.3 | HBP1 | ILMN_167468 | 1.58 |
| NM_001013690.1 | LOC401720 | ILMN_21595 | 1.58 |
| NM_032308.1 | RPAIN | ILMN_15409 | 1.58 |
| NM_001387.2 | DPYSL3 | ILMN_23309 | 1.58 |
| NM_005238.2 | ETS1 | ILMN_173009 | 1.57 |
| NM_016605.1 | FAM53C | ILMN_11637 | 1.57 |
| NM_145914.2 | ZSCAN21 | ILMN_2862 | 1.57 |
| XM_001132754.1 | LOC728734 | ILMN_169578 | 1.57 |
| NM_014647.2 | KIAA0430 | ILMN_15427 | 1.57 |
| NM_001030272.1 | ARNTL | ILMN_6754 | 1.57 |
| NM_033394.1 | TANC1 | ILMN_182363 | 1.57 |
| NM_032424.1 | KIAA1826 | ILMN_22604 | 1.57 |
| NM_015446.3 | AHCTF1 | ILMN_164192 | 1.57 |
| NM_002268.3 | KPNA4 | ILMN_21107 | 1.57 |
| NM_006047.4 | RBM12 | ILMN_183773 | 1.56 |
| NM_003420.3 | ZNF35 | ILMN_180943 | 1.56 |
| NM_003906.3 | MCM3AP | ILMN_19614 | 1.56 |
| NM_001280.1 | CIRBP | ILMN_24327 | 1.56 |
| NM_024109.2 | C16orf68 | ILMN_12265 | 1.55 |
| NM_001031617.2 | COX19 | ILMN_15655 | 1.55 |
| NM_015044.3 | GGA2 | ILMN_17168 | 1.55 |
| NM_199420.3 | POLQ | ILMN_10389 | 1.55 |
| NM_032621.2 | BEX2 | ILMN_24134 | 1.55 |
| XM_929980.2 | LOC647000 | ILMN_34401 | 1.55 |
| NM_004656.2 | BAP1 | ILMN_17024 | 1.54 |
| NM_032626.5 | RBBP6 | ILMN_19179 | 1.54 |
| NM_015517.3 | MIZF | ILMN_3194 | 1.54 |
| NM_018992.2 | KCTD5 | ILMN_17737 | 1.54 |
| NM_014603.1 | CDR2L | ILMN_26231 | 1.54 |
| NM_017730.2 | QRICH1 | ILMN_22436 | 1.54 |
| NM_005124.2 | NUP153 | ILMN_11793 | 1.54 |
| NM_014607.3 | UBXD2 | ILMN_26387 | 1.54 |
| NM_014889.2 | PITRM1 | ILMN_22239 | 1.53 |
| NM_003047.2 | SLC9A1 | ILMN_166750 | 1.53 |
| NM_174891.3 | C14orf79 | ILMN_22555 | 1.53 |
| NM_001987.4 | ETV6 | ILMN_175744 | 1.53 |
| NM_001407.2 | CELSR3 | ILMN_162782 | 1.53 |
| NM_004078.1 | CSRP1 | ILMN_25451 | 1.53 |
| NM_022766.4 | CERK | ILMN_24122 | 1.53 |
| NM_001567.2 | INPPL1 | ILMN_20903 | 1.53 |
| NM_001013839.1 | EXOC7 | ILMN_25212 | 1.53 |
| NM_006571.2 | DCTN6 | ILMN_29012 | 1.53 |
| NM_207577.1 | MAP6 | ILMN_5510 | 1.53 |
| NM_001001787.1 | ATP1B1 | ILMN_10855 | 1.53 |
| NM_006516.1 | SLC2A1 | ILMN_421 | 1.53 |
| NM_003211.3 | TDG | ILMN_29212 | 1.53 |
| NM_133340.1 | RAD17 | ILMN_4423 | 1.52 |
| NM_001008490.1 | KLF6 | ILMN_12381 | 1.52 |
| NM_005137.2 | DGCR2 | ILMN_6763 | 1.52 |
| NM_173798.2 | ZCCHC12 | ILMN_7344 | 1.52 |
| NM_001039712.1 | DEDD | ILMN_41741 | 1.52 |
| NM_018445.4 | SELS | ILMN_15886 | 1.52 |
| NM_006955.1 | ZNF33B | ILMN_13648 | 1.52 |
| NM_014853.2 | SGSM2 | ILMN_9226 | 1.52 |
| NM_012260.2 | HACL1 | ILMN_180681 | 1.52 |
| NM_015534.4 | ZZZ3 | ILMN_14976 | 1.52 |
| NM_001013649.1 | LOC388969 | ILMN_138621 | 1.51 |
| NM_015327.1 | SMG5 | ILMN_10815 | 1.51 |
| NM_015455.3 | CNOT6 | ILMN_17926 | 1.51 |
| NM_001845.4 | COL4A1 | ILMN_24359 | 1.51 |
| NM_016061.1 | YPEL5 | ILMN_8828 | 1.51 |
| NM_002926.3 | RGS12 | ILMN_161894 | 1.5 |
| NM_006825.2 | CKAP4 | ILMN_182487 | 1.5 |
| XR_018793.1 | LOC400455 | ILMN_162119 | 1.5 |
| NM_152265.2 | BTF3L4 | ILMN_3105 | 1.5 |
| NM_021942.4 | C4orf41 | ILMN_8900 | 1.5 |
| NM_022037.1 | TIA1 | ILMN_30157 | -1.5 |
| NM_016498.3 | MTP18 | ILMN_13393 | -1.5 |
| NM_001034996.1 | RPL14 | ILMN_2719 | -1.51 |
| XM_001130192.1 | KIAA1160 | ILMN_162086 | -1.51 |
| NM_004477.2 | FRG1 | ILMN_11683 | -1.51 |
| NM_024632.4 | SAP30L | ILMN_18384 | -1.51 |
| NM_007308.1 | SNCA | ILMN_2235 | -1.51 |
| NM_006324.2 | CFDP1 | ILMN_23508 | -1.51 |
| NM_003848.1 | SUCLG2 | ILMN_20828 | -1.52 |
| NM_002685.2 | EXOSC10 | ILMN_25853 | -1.52 |
| NM_005896.2 | IDH1 | ILMN_14217 | -1.52 |
| NM_001014286.2 | FAM48A | ILMN_1616 | -1.52 |
| NM_001040056.1 | MAPK3 | ILMN_177323 | -1.53 |
| XM_926594.2 | LOC642502 | ILMN_31759 | -1.53 |
| NM_133646.2 | ZAK | ILMN_5666 | -1.53 |
| NM_018846.2 | KLHL7 | ILMN_21425 | -1.53 |
| NM_145255.2 | MRPL10 | ILMN_19178 | -1.53 |
| NM_004593.1 | SFRS10 | ILMN_22721 | -1.53 |
| NM_001017405.1 | MAEA | ILMN_4828 | -1.54 |
| XM_374020.4 | LOC375295 | ILMN_45377 | -1.54 |
| NM_176866.2 | PPA2 | ILMN_15275 | -1.54 |
| NM_030752.2 | TCP1 | ILMN_418 | -1.54 |
| NM_021177.3 | LSM2 | ILMN_22587 | -1.54 |
| NM_020195.1 | C14orf124 | ILMN_4144 | -1.55 |
| XM_001126212.1 | C7orf28B | ILMN_172434 | -1.55 |
| NM_013354.5 | CNOT7 | ILMN_7214 | -1.55 |
| NM_001042370.1 | TROVE2 | ILMN_173505 | -1.56 |
| NM_014168.2 | METTL5 | ILMN_9336 | -1.56 |
| NM_002755.2 | MAP2K1 | ILMN_164648 | -1.56 |
| NM_003009.2 | SEPW1 | ILMN_6755 | -1.56 |
| NM_018246.2 | CCDC25 | ILMN_5229 | -1.56 |
| NM_198047.1 | HIBCH | ILMN_24888 | -1.56 |
| NM_148973.1 | TNFRSF25 | ILMN_14916 | -1.56 |
| NM_015525.2 | IBTK | ILMN_164723 | -1.56 |
| XM_944104.2 | LOC653232 | ILMN_41197 | -1.56 |
| NM_015609.2 | C1orf144 | ILMN_5836 | -1.57 |
| NM_020147.2 | THAP10 | ILMN_182683 | -1.57 |
| NM_145644.1 | MRPL35 | ILMN_20736 | -1.57 |
| NM_181306.1 | MRPL52 | ILMN_16276 | -1.57 |
| NM_018334.3 | LRRN3 | ILMN_174401 | -1.57 |
| NM_019858.1 | GPR162 | ILMN_27943 | -1.57 |
| XM_496446.3 | LOC440737 | ILMN_39347 | -1.57 |
| NM_201443.1 | TEAD4 | ILMN_21735 | -1.58 |
| NM_002154.3 | HSPA4 | ILMN_166427 | -1.58 |
| NM_138418.2 | C16orf14 | ILMN_9509 | -1.58 |
| NM_003077.2 | SMARCD2 | ILMN_14227 | -1.58 |
| NM_001321.1 | CSRP2 | ILMN_3862 | -1.58 |
| NM_016142.1 | HSD17B12 | ILMN_19305 | -1.58 |
| NM_031267.1 | CDC2L5 | ILMN_29859 | -1.58 |
| XM_929199.1 | LOC644250 | ILMN_30796 | -1.59 |
| NM_006441.1 | MTHFS | ILMN_1014 | -1.59 |
| NM_005869.2 | SDCCAG10 | ILMN_3741 | -1.59 |
| NM_006282.2 | STK4 | ILMN_21491 | -1.59 |
| NM_031452.2 | FAM103A1 | ILMN_28104 | -1.59 |
| NM_001031.4 | RPS28 | ILMN_992 | -1.59 |
| NR_002190.1 | SUMO1P3 | ILMN_16906 | -1.59 |
| NM_018561.3 | USP49 | ILMN_24018 | -1.59 |
| NM_003677.3 | DENR | ILMN_181187 | -1.59 |
| NM_001382.2 | DPAGT1 | ILMN_10306 | -1.6 |
| NM_017910.2 | FLJ20628 | ILMN_29305 | -1.6 |
| NM_014142.2 | NUDT5 | ILMN_1656 | -1.6 |
| NM_080651.1 | MED30 | ILMN_7158 | -1.6 |
| NM_016308.1 | CMPK1 | ILMN_12452 | -1.6 |
| NM_173666.1 | DTWD2 | ILMN_25915 | -1.6 |
| NM_031298.2 | TMEM93 | ILMN_9888 | -1.6 |
| NM_001033503.1 | SAR1B | ILMN_16595 | -1.6 |
| NM_024678.3 | NARS2 | ILMN_13605 | -1.6 |
| NM_030660.2 | ATXN3 | ILMN_12637 | -1.61 |
| NM_024516.2 | C16orf53 | ILMN_20272 | -1.61 |
| NR_002201.1 | FTHL3 | ILMN_27691 | -1.61 |
| NM_001918.2 | DBT | ILMN_169961 | -1.61 |
| NM_002266.2 | KPNA2 | ILMN_14206 | -1.61 |
| NM_006391.1 | IPO7 | ILMN_28842 | -1.61 |
| NM_001042678.1 | RHOC | ILMN_162499 | -1.61 |
| NM_014933.2 | SEC31A | ILMN_23819 | -1.61 |
| NM_012482.3 | ZNF281 | ILMN_18970 | -1.61 |
| NM_052857.2 | CCDC16 | ILMN_23839 | -1.62 |
| NM_015423.2 | AASDHPPT | ILMN_25075 | -1.62 |
| NM_000984.5 | RPL23A | ILMN_9569 | -1.62 |
| XM_929420.1 | LOC653377 | ILMN_43949 | -1.62 |
| NM_032728.2 | PPAPDC3 | ILMN_25638 | -1.63 |
| NM_080386.1 | TUBA3D | ILMN_30319 | -1.63 |
| NM_015888.4 | HOOK1 | ILMN_173692 | -1.63 |
| NM_001013701.1 | LOC440157 | ILMN_20657 | -1.63 |
| NM_175875.3 | SIX5 | ILMN_21099 | -1.64 |
| NM_001813.2 | CENPE | ILMN_7509 | -1.64 |
| NM_016297.2 | PCYOX1 | ILMN_15130 | -1.64 |
| NM_000230.1 | LEP | ILMN_10827 | -1.64 |
| NM_146387.1 | MRPL4 | ILMN_13422 | -1.64 |
| XM_930029.1 | LOC647037 | ILMN_32596 | -1.64 |
| NM_020463.1 | SMEK2 | ILMN_21228 | -1.64 |
| NM_003143.1 | SSBP1 | ILMN_30122 | -1.64 |
| NM_007358.2 | MTF2 | ILMN_24749 | -1.65 |
| XM_935589.1 | LOC641849 | ILMN_45563 | -1.65 |
| NM_001033566.1 | RHOT1 | ILMN_6821 | -1.65 |
| NM_001540.2 | HSPB1 | ILMN_28967 | -1.65 |
| NM_001112.2 | ADARB1 | ILMN_30004 | -1.66 |
| NM_001914.2 | CYB5A | ILMN_25182 | -1.66 |
| NM_012170.2 | FBXO22 | ILMN_5718 | -1.66 |
| NM_198038.1 | NUDT9 | ILMN_12448 | -1.66 |
| NM_182533.1 | C1orf86 | ILMN_2880 | -1.66 |
| NM_014078.4 | MRPL13 | ILMN_17393 | -1.66 |
| NM_001037277.1 | GGPS1 | ILMN_4748 | -1.66 |
| NM_182679.1 | GPATCH4 | ILMN_839 | -1.67 |
| NM_003342.4 | UBE2G1 | ILMN_179729 | -1.67 |
| NM_000997.3 | RPL37 | ILMN_138392 | -1.67 |
| NM_014039.2 | C11orf54 | ILMN_4783 | -1.67 |
| XM_937928.1 | LOC347376 | ILMN_31523 | -1.67 |
| NM_018229.2 | C14orf108 | ILMN_180528 | -1.67 |
| NM_004879.3 | EI24 | ILMN_8791 | -1.67 |
| NM_004462.3 | FDFT1 | ILMN_24060 | -1.67 |
| NM_001031703.2 | TMEM103 | ILMN_40105 | -1.68 |
| NM_182569.1 | GDPD1 | ILMN_172818 | -1.68 |
| NM_138809.3 | CMBL | ILMN_1485 | -1.68 |
| NM_006761.3 | YWHAE | ILMN_18524 | -1.69 |
| NM_001040668.1 | BCL2L12 | ILMN_177176 | -1.69 |
| NM_030940.3 | ISCA1 | ILMN_171173 | -1.69 |
| NM_014169.2 | CHMP4A | ILMN_19959 | -1.7 |
| NM_016558.2 | SCAND1 | ILMN_23327 | -1.7 |
| NM_006366.2 | CAP2 | ILMN_27367 | -1.7 |
| NM_015480.1 | PVRL3 | ILMN_2284 | -1.7 |
| NM_001080546.1 | LOC219854 | ILMN_168339 | -1.7 |
| NM_022087.2 | GALNT11 | ILMN_5237 | -1.7 |
| NM_017526.2 | LEPROT | ILMN_27032 | -1.7 |
| NR_001562.1 | ANXA2P1 | ILMN_10494 | -1.7 |
| NM_032439.1 | PHYHIPL | ILMN_22045 | -1.7 |
| NM_002669.2 | PLRG1 | ILMN_22972 | -1.7 |
| NM_017819.2 | RG9MTD1 | ILMN_26970 | -1.71 |
| NM_207380.1 | C15orf52 | ILMN_1132 | -1.71 |
| NM_016042.2 | EXOSC3 | ILMN_174330 | -1.71 |
| NM_145800.2 | SEPT6 | ILMN_29094 | -1.71 |
| NM_024540.2 | MRPL24 | ILMN_29128 | -1.71 |
| NM_015984.2 | UCHL5 | ILMN_3370 | -1.71 |
| NM_018480.2 | TMEM126B | ILMN_18826 | -1.71 |
| NM_016039.1 | C14orf166 | ILMN_14906 | -1.72 |
| NM_174921.1 | C4orf34 | ILMN_6140 | -1.72 |
| NM_005810.3 | KLRG1 | ILMN_12613 | -1.72 |
| NM_001007230.1 | SPOP | ILMN_12838 | -1.72 |
| NM_020529.1 | NFKBIA | ILMN_6745 | -1.72 |
| NM_207350.1 | MGC72104 | ILMN_26269 | -1.72 |
| NM_152524.3 | SGOL2 | ILMN_743 | -1.72 |
| NM_031314.1 | HNRPC | ILMN_24356 | -1.73 |
| NM_014933.2 | SEC31A | ILMN_23819 | -1.73 |
| NM_138361.3 | LRSAM1 | ILMN_21244 | -1.73 |
| NM_012111.1 | AHSA1 | ILMN_11051 | -1.73 |
| NM_032574.2 | DPY30 | ILMN_18534 | -1.73 |
| NM_002129.2 | HMGB2 | ILMN_3200 | -1.73 |
| NR_002200.1 | FTHL2 | ILMN_15867 | -1.74 |
| NM_181705.1 | LOC90624 | ILMN_11045 | -1.74 |
| NM_001039379.1 | C20orf191 | ILMN_25650 | -1.74 |
| NM_031943.1 | IFP38 | ILMN_9478 | -1.74 |
| NM_001003897.1 | MANBAL | ILMN_11747 | -1.74 |
| NM_020235.3 | BBX | ILMN_28437 | -1.74 |
| NM_012460.2 | TIMM9 | ILMN_9968 | -1.74 |
| XM_941195.2 | LOC388621 | ILMN_42661 | -1.74 |
| NM_170784.1 | MKKS | ILMN_17701 | -1.75 |
| NM_198486.2 | RPL7L1 | ILMN_9155 | -1.75 |
| NM_001031684.1 | SFRS7 | ILMN_7620 | -1.75 |
| NM_002350.1 | LYN | ILMN_10095 | -1.75 |
| NM_138458.2 | WDR92 | ILMN_37809 | -1.76 |
| NM_001003793.1 | RBMS3 | ILMN_16411 | -1.76 |
| NM_139286.3 | CDC26 | ILMN_18022 | -1.76 |
| NM_020385.2 | REXO4 | ILMN_29774 | -1.77 |
| NM_002528.4 | NTHL1 | ILMN_15981 | -1.77 |
| NM_003403.3 | YY1 | ILMN_4019 | -1.77 |
| NM_001011537.1 | FYTTD1 | ILMN_5513 | -1.78 |
| NM_058181.1 | C21orf57 | ILMN_21121 | -1.78 |
| NM_205847.1 | GMPPA | ILMN_23338 | -1.78 |
| NM_001326.2 | CSTF3 | ILMN_27551 | -1.78 |
| NM_000971.3 | RPL7 | ILMN_26351 | -1.78 |
| NM_005188.2 | CBL | ILMN_172998 | -1.79 |
| NM_000527.2 | LDLR | ILMN_10126 | -1.79 |
| NM_000599.2 | IGFBP5 | ILMN_168089 | -1.79 |
| NM_016098.1 | BRP44L | ILMN_4349 | -1.8 |
| XM_936354.2 | LOC642197 | ILMN_44406 | -1.81 |
| NM_203284.1 | RBPJ | ILMN_170184 | -1.81 |
| XM_925818.1 | LOC642282 | ILMN_41968 | -1.81 |
| NM_003746.1 | DNCL1 | ILMN_137049 | -1.81 |
| NM_020548.4 | DBI | ILMN_30320 | -1.82 |
| NM_023077.1 | C1orf163 | ILMN_14119 | -1.82 |
| NM_001031677.2 | RAB24 | ILMN_25731 | -1.82 |
| NM_018390.2 | PLCXD1 | ILMN_8273 | -1.82 |
| NM_014161.2 | MRPL18 | ILMN_14120 | -1.82 |
| XM_929862.1 | LOC646900 | ILMN_44661 | -1.82 |
| NM_001008800.1 | CCT3 | ILMN_24878 | -1.82 |
| NM_015934.3 | NOP5/NOP58 | ILMN_4530 | -1.82 |
| NM_002004.2 | FDPS | ILMN_18516 | -1.83 |
| NR_002197.1 | LOC143543 | ILMN_17694 | -1.83 |
| NM_016098.1 | BRP44L | ILMN_4349 | -1.83 |
| NM_018079.3 | SRBD1 | ILMN_28720 | -1.83 |
| XM_001133534.1 | ATP1B3 | ILMN_163124 | -1.83 |
| XM_001133089.1 | LOC731640 | ILMN_161930 | -1.83 |
| XR_019071.1 | LOC642333 | ILMN_183964 | -1.84 |
| XM_001132569.1 | LOC730130 | ILMN_162537 | -1.84 |
| NM_173659.2 | RPUSD3 | ILMN_28804 | -1.84 |
| NM_006191.2 | PA2G4 | ILMN_28541 | -1.84 |
| NM_016395.2 | PTPLAD1 | ILMN_9196 | -1.85 |
| NM_001097615.1 | POLR2J3 | ILMN_308895 | -1.85 |
| NM_021825.3 | CCDC90B | ILMN_6208 | -1.86 |
| NM_033405.2 | PRIC285 | ILMN_10778 | -1.86 |
| NM_052879.3 | LARP4 | ILMN_2132 | -1.86 |
| NM_032476.2 | MRPS6 | ILMN_17239 | -1.86 |
| NM_012484.1 | HMMR | ILMN_17450 | -1.86 |
| NM_001037442.1 | RUFY3 | ILMN_28746 | -1.87 |
| NM_021244.3 | RRAGD | ILMN_5663 | -1.87 |
| NM_058216.1 | RAD51C | ILMN_2944 | -1.87 |
| NM_007369.2 | GPR161 | ILMN_22837 | -1.87 |
| NM_003002.1 | SDHD | ILMN_6353 | -1.87 |
| NM_013300.1 | C12orf24 | ILMN_24807 | -1.87 |
| NM_004774.2 | PPARBP | ILMN_14182 | -1.88 |
| NM_003729.2 | RTCD1 | ILMN_11697 | -1.88 |
| NM_133459.1 | CCBE1 | ILMN_6075 | -1.88 |
| NM_021156.2 | TXNDC13 | ILMN_23065 | -1.88 |
| NM_000645.2 | AGL | ILMN_1173 | -1.89 |
| NM_001938.2 | DR1 | ILMN_182864 | -1.89 |
| NM_018244.3 | UQCC | ILMN_26543 | -1.89 |
| XM_943005.1 | LOC642236 | ILMN_31082 | -1.9 |
| NM_017946.2 | FKBP14 | ILMN_18132 | -1.9 |
| NM_005873.2 | RGS19 | ILMN_42727 | -1.9 |
| NM_145697.1 | CDCA1 | ILMN_17725 | -1.9 |
| NM_006281.2 | STK3 | ILMN_26935 | -1.9 |
| NM_152755.1 | CNPY4 | ILMN_15383 | -1.9 |
| NM_001005369.1 | MTIF2 | ILMN_165311 | -1.9 |
| NM_182810.1 | ATF4 | ILMN_23435 | -1.91 |
| NM_053067.1 | UBQLN1 | ILMN_9768 | -1.91 |
| NM_033115.2 | MGC16169 | ILMN_16160 | -1.91 |
| NM_021971.1 | GMPPB | ILMN_3929 | -1.91 |
| NM_145117.3 | NAV2 | ILMN_8536 | -1.92 |
| NM_006870.3 | DSTN | ILMN_13158 | -1.92 |
| NM_001042401.1 | C21orf51 | ILMN_179828 | -1.92 |
| NM_025233.5 | COASY | ILMN_13627 | -1.92 |
| NM_001031677.2 | RAB24 | ILMN_25731 | -1.92 |
| XM_936354.2 | LOC642197 | ILMN_44406 | -1.93 |
| NM_003800.3 | RNGTT | ILMN_17056 | -1.93 |
| NM_018004.1 | TMEM45A | ILMN_30168 | -1.93 |
| NM_148178.1 | C9orf23 | ILMN_3926 | -1.94 |
| NM_014170.2 | GTPBP8 | ILMN_27163 | -1.94 |
| NM_001007157.1 | PHF14 | ILMN_2096 | -1.94 |
| NM_018227.5 | UBA6 | ILMN_16506 | -1.95 |
| NM_005476.3 | GNE | ILMN_29772 | -1.95 |
| XR_015809.1 | LOC728973 | ILMN_168278 | -1.95 |
| NM_015449.2 | C1orf43 | ILMN_933 | -1.96 |
| XM_939682.1 | LOC149448 | ILMN_36821 | -1.96 |
| NM_138720.1 | HIST1H2BD | ILMN_17622 | -1.96 |
| NM_003750.2 | EIF3A | ILMN_25761 | -1.96 |
| NM_001025248.1 | DUT | ILMN_163345 | -1.96 |
| NM_001535.2 | PRMT2 | ILMN_10737 | -1.97 |
| NM_002643.3 | PIGF | ILMN_15261 | -1.97 |
| NM_006265.1 | RAD21 | ILMN_171453 | -1.97 |
| NM_001031744.1 | LOC158160 | ILMN_21155 | -1.98 |
| NM_013388.4 | PREB | ILMN_6913 | -1.98 |
| NM_180981.1 | MRPL52 | ILMN_3474 | -1.98 |
| NM_004252.2 | SLC9A3R1 | ILMN_1421 | -1.98 |
| NM_001007793.1 | BUB3 | ILMN_5688 | -1.99 |
| NM_006963.3 | ZNF22 | ILMN_165495 | -1.99 |
| NM_005388.3 | PDCL | ILMN_34020 | -2 |
| NM_001100164.1 | PHACTR2 | ILMN_307784 | -2.01 |
| NM_014184.2 | CNIH4 | ILMN_9903 | -2.01 |
| NM_003400.3 | XPO1 | ILMN_16600 | -2.01 |
| NM_018847.2 | KLHL9 | ILMN_20376 | -2.02 |
| NR_000029.1 | RPL23AP7 | ILMN_3185 | -2.02 |
| NM_030752.2 | TCP1 | ILMN_418 | -2.02 |
| NM_000051.3 | ATM | ILMN_162851 | -2.03 |
| NM_005644.2 | TAF12 | ILMN_3797 | -2.03 |
| XM_942442.1 | LOC654121 | ILMN_35777 | -2.03 |
| NM_001003712.1 | OSBPL8 | ILMN_29956 | -2.04 |
| NM_020150.3 | SAR1A | ILMN_17495 | -2.04 |
| XM_936215.1 | LOC653874 | ILMN_35327 | -2.05 |
| XR_015313.1 | LOC653080 | ILMN_172174 | -2.05 |
| NM_145647.2 | WDR67 | ILMN_20846 | -2.05 |
| NM_024527.4 | ABHD8 | ILMN_23791 | -2.05 |
| NM_006644.2 | HSPH1 | ILMN_1157 | -2.05 |
| NM_198402.2 | PTPLB | ILMN_183743 | -2.05 |
| NM_152912.3 | MTIF3 | ILMN_16655 | -2.06 |
| NM_058216.1 | RAD51C | ILMN_2944 | -2.06 |
| NM_005413.1 | SIX3 | ILMN_26476 | -2.07 |
| NM_002613.3 | PDPK1 | ILMN_27765 | -2.07 |
| NM_001082576.1 | RBM9 | ILMN_307545 | -2.07 |
| NM_021622.3 | PLEKHA1 | ILMN_9430 | -2.07 |
| XM_939687.2 | LOC653658 | ILMN_33948 | -2.07 |
| XR_018327.1 | LOC648343 | ILMN_163789 | -2.08 |
| NM_001889.2 | CRYZ | ILMN_30248 | -2.08 |
| NM_001099222.1 | IFT74 | ILMN_306953 | -2.09 |
| NM_017802.2 | HEATR2 | ILMN_1114 | -2.09 |
| NM_003924.2 | PHOX2B | ILMN_172224 | -2.09 |
| NM_002870.2 | RAB13 | ILMN_26464 | -2.09 |
| NM_016071.2 | MRPS33 | ILMN_4243 | -2.1 |
| NM_005056.1 | JARID1A | ILMN_12150 | -2.1 |
| NR_001568.1 | BCYRN1 | ILMN_21987 | -2.11 |
| NM_001018109.1 | PIR | ILMN_13999 | -2.11 |
| NM_201262.1 | DNAJC12 | ILMN_18576 | -2.11 |
| NM_017958.1 | PLEKHB2 | ILMN_29704 | -2.12 |
| NM_001014438.1 | CARS | ILMN_172747 | -2.12 |
| NM_022048.3 | CSNK1G1 | ILMN_19512 | -2.12 |
| NM_000978.3 | RPL23 | ILMN_8866 | -2.13 |
| NM_032490.4 | C14orf142 | ILMN_166160 | -2.13 |
| NM_001014812.1 | FAM96A | ILMN_13780 | -2.13 |
| NM_016551.1 | TM7SF3 | ILMN_7797 | -2.14 |
| NM_012319.2 | SLC39A6 | ILMN_170037 | -2.14 |
| XM_936240.1 | LOC653884 | ILMN_34094 | -2.14 |
| NM_024051.2 | C7orf24 | ILMN_2391 | -2.14 |
| NM_198336.1 | INSIG1 | ILMN_12839 | -2.14 |
| NM_002095.4 | GTF2E2 | ILMN_4316 | -2.15 |
| NM_004365.2 | CETN3 | ILMN_25663 | -2.15 |
| NM_005842.2 | SPRY2 | ILMN_19344 | -2.16 |
| NM_025191.2 | EDEM3 | ILMN_15796 | -2.17 |
| NM_015475.3 | FAM98A | ILMN_16819 | -2.17 |
| NM_032226.2 | ZCCHC7 | ILMN_21489 | -2.17 |
| NM_001033566.1 | RHOT1 | ILMN_6821 | -2.17 |
| NR_002308.1 | LOC442454 | ILMN_309609 | -2.17 |
| NM_053067.1 | UBQLN1 | ILMN_9768 | -2.18 |
| NM_016824.3 | ADD3 | ILMN_3908 | -2.18 |
| NR_003040.1 | LOC649946 | ILMN_169528 | -2.18 |
| NM_015017.3 | USP33 | ILMN_176756 | -2.19 |
| NM_003715.2 | USO1 | ILMN_23419 | -2.19 |
| NM_015395.1 | DKFZP434B0335 | ILMN_11830 | -2.2 |
| NM_001039141.1 | TRIOBP | ILMN_34620 | -2.2 |
| NM_001008735.1 | HMG1L1 | ILMN_22757 | -2.2 |
| XM_942780.2 | SYNPO2 | ILMN_45907 | -2.2 |
| XM_940333.2 | LOC651202 | ILMN_37363 | -2.2 |
| NM_003403.3 | YY1 | ILMN_4019 | -2.2 |
| NM_024749.2 | VASH2 | ILMN_3016 | -2.2 |
| NM_002759.1 | EIF2AK2 | ILMN_168435 | -2.21 |
| NM_182547.2 | TMED4 | ILMN_30359 | -2.21 |
| XR_018325.1 | LOC644131 | ILMN_166020 | -2.21 |
| NM_018983.3 | NOLA1 | ILMN_14204 | -2.22 |
| NM_021127.1 | PMAIP1 | ILMN_25637 | -2.22 |
| NM_003107.2 | SOX4 | ILMN_17456 | -2.22 |
| NM_007167.2 | ZMYM6 | ILMN_1275 | -2.22 |
| NM_000076.1 | CDKN1C | ILMN_20689 | -2.22 |
| NM_000913.3 | OPRL1 | ILMN_6491 | -2.23 |
| NM_001008566.1 | TPST2 | ILMN_13248 | -2.23 |
| NM_006392.2 | NOL5A | ILMN_13841 | -2.23 |
| NM_002486.4 | NCBP1 | ILMN_23411 | -2.23 |
| NM_006745.3 | SC4MOL | ILMN_2770 | -2.24 |
| NM_003864.3 | SAP30 | ILMN_31250 | -2.24 |
| XM_938755.2 | LOC653773 | ILMN_44662 | -2.24 |
| NM_003729.1 | RTCD1 | ILMN_11697 | -2.26 |
| NM_004544.2 | NDUFA10 | ILMN_7463 | -2.26 |
| NM_016297.2 | PCYOX1 | ILMN_15130 | -2.27 |
| NM_012342.2 | BAMBI | ILMN_8469 | -2.27 |
| NM_021227.2 | DC2 | ILMN_24748 | -2.27 |
| XM_933893.1 | LOC389672 | ILMN_35589 | -2.28 |
| NM_017915.2 | C12orf48 | ILMN_42497 | -2.28 |
| NM_018639.3 | WSB2 | ILMN_162438 | -2.29 |
| NR_002204.1 | FTHL11 | ILMN_16343 | -2.3 |
| XM_001133534.1 | ATP1B3 | ILMN_163124 | -2.3 |
| NM_004792.2 | PPIG | ILMN_24595 | -2.31 |
| NM_001034996.1 | RPL14 | ILMN_2719 | -2.31 |
| NM_032361.1 | THOC3 | ILMN_17969 | -2.31 |
| NM_001008405.1 | BCAP29 | ILMN_24800 | -2.31 |
| XM_935588.1 | LOC641848 | ILMN_45490 | -2.31 |
| XM_942669.1 | LOC654194 | ILMN_31988 | -2.34 |
| NM_201280.1 | MUTED | ILMN_21576 | -2.35 |
| NM_006914.3 | RORB | ILMN_7297 | -2.35 |
| NM_020119.3 | ZC3HAV1 | ILMN_13243 | -2.36 |
| NM_001024921.2 | RPL9 | ILMN_8640 | -2.36 |
| NM_024813.1 | RPAP2 | ILMN_23904 | -2.36 |
| NR_002182.1 | NACAP1 | ILMN_14666 | -2.37 |
| NM_018844.2 | BCAP29 | ILMN_24686 | -2.37 |
| NM_000051.3 | ATM | ILMN_162851 | -2.37 |
| XM_945544.1 | UBE2Z | ILMN_137054 | -2.38 |
| NM_005754.2 | G3BP1 | ILMN_1152 | -2.38 |
| NM_005708.2 | GPC6 | ILMN_16550 | -2.39 |
| NM_014322.2 | OPN3 | ILMN_166169 | -2.39 |
| NM_025136.1 | OPA3 | ILMN_11296 | -2.39 |
| NM_007198.2 | PROSC | ILMN_23472 | -2.39 |
| NM_152789.2 | FAM133B | ILMN_1247 | -2.39 |
| XM_938089.2 | LOC643007 | ILMN_31054 | -2.4 |
| XM_941876.1 | BRI3BP | ILMN_139088 | -2.4 |
| NM_000814.4 | GABRB3 | ILMN_19294 | -2.4 |
| NM_024920.3 | DNAJB14 | ILMN_12080 | -2.42 |
| NM_024090.1 | ELOVL6 | ILMN_11340 | -2.42 |
| NM_014941.1 | MORC2 | ILMN_12502 | -2.43 |
| NM_006601.4 | PTGES3 | ILMN_3176 | -2.43 |
| NM_031966.2 | CCNB1 | ILMN_177161 | -2.43 |
| NM_006158.2 | NEFL | ILMN_22054 | -2.44 |
| NM_001634.4 | AMD1 | ILMN_21529 | -2.44 |
| NM_006597.3 | HSPA8 | ILMN_181529 | -2.44 |
| NM_005443.4 | PAPSS1 | ILMN_171260 | -2.45 |
| NM_001012968.2 | SPIN4 | ILMN_4105 | -2.45 |
| NM_017958.1 | PLEKHB2 | ILMN_29704 | -2.46 |
| NM_014140.2 | SMARCAL1 | ILMN_19734 | -2.46 |
| XM_497072.2 | LOC389787 | ILMN_45784 | -2.46 |
| NM_000819.3 | GART | ILMN_22974 | -2.47 |
| NM_005909.3 | MAP1B | ILMN_28251 | -2.48 |
| NM_022079.2 | HERC4 | ILMN_8869 | -2.48 |
| NR_002205.1 | FTHL12 | ILMN_16447 | -2.48 |
| NM_001121.2 | ADD3 | ILMN_4026 | -2.49 |
| NM_018473.2 | THEM2 | ILMN_27212 | -2.49 |
| NM_174909.3 | TMEM167 | ILMN_6582 | -2.49 |
| NM_004987.3 | LIMS1 | ILMN_11207 | -2.5 |
| NM_032138.3 | KBTBD7 | ILMN_181309 | -2.5 |
| NM_020153.2 | C11orf60 | ILMN_171038 | -2.51 |
| NM_005749.2 | TOB1 | ILMN_13735 | -2.51 |
| NM_015449.2 | C1orf43 | ILMN_933 | -2.51 |
| NM_001037494.1 | DYNLL1 | ILMN_14802 | -2.51 |
| NM_014620.4 | HOXC4 | ILMN_16005 | -2.53 |
| NM_004627.2 | WRB | ILMN_12263 | -2.53 |
| XM_371655.3 | LOC389137 | ILMN_163284 | -2.53 |
| NM_023071.1 | SPATS2 | ILMN_10985 | -2.54 |
| NM_006117.2 | PECI | ILMN_7427 | -2.54 |
| NM_001003793.1 | RBMS3 | ILMN_16411 | -2.54 |
| XM_941684.2 | LOC220433 | ILMN_46655 | -2.54 |
| NM_017812.2 | CHCHD3 | ILMN_23539 | -2.55 |
| NM_015948.2 | SLC35B3 | ILMN_20545 | -2.55 |
| NM_033212.2 | CCDC102A | ILMN_12942 | -2.56 |
| NM_079837.2 | BANP | ILMN_8638 | -2.56 |
| NM_001008783.1 | SLC35D3 | ILMN_16642 | -2.56 |
| NM_005836.2 | HRSP12 | ILMN_8062 | -2.56 |
| NM_138807.2 | C3orf31 | ILMN_9705 | -2.57 |
| NR_002315.1 | LOC440926 | ILMN_19720 | -2.59 |
| NM_014865.2 | NCAPD2 | ILMN_26621 | -2.59 |
| NM_001008735.1 | HMG1L1 | ILMN_22757 | -2.6 |
| NM_178314.2 | RILPL1 | ILMN_1609 | -2.61 |
| XM_938599.2 | LOC441377 | ILMN_31681 | -2.61 |
| NR_002203.1 | FTHL8 | ILMN_16227 | -2.61 |
| NM_014033.3 | METTL7A | ILMN_40171 | -2.62 |
| NM_033064.3 | ATCAY | ILMN_27014 | -2.64 |
| NM_001010982.2 | AFMID | ILMN_5520 | -2.65 |
| NR_002205.1 | FTHL12 | ILMN_16447 | -2.65 |
| NM_183422.1 | TSC22D1 | ILMN_166165 | -2.66 |
| NM_001040285.1 | PAPD5 | ILMN_167231 | -2.66 |
| NM_018297.2 | NGLY1 | ILMN_15318 | -2.66 |
| NM_002338.2 | LSAMP | ILMN_861 | -2.67 |
| NM_015523.2 | REXO2 | ILMN_15016 | -2.67 |
| NM_012117.1 | CBX5 | ILMN_25072 | -2.67 |
| NM_014060.1 | MCTS1 | ILMN_13725 | -2.68 |
| NM_005713.1 | COL4A3BP | ILMN_10635 | -2.69 |
| NM_002157.1 | HSPE1 | ILMN_2612 | -2.69 |
| NM_001031706.1 | PLEKHB2 | ILMN_179121 | -2.71 |
| NM_001539.2 | DNAJA1 | ILMN_5819 | -2.71 |
| NM_001786.2 | CDC2 | ILMN_24793 | -2.72 |
| NM_001003.2 | RPLP1 | ILMN_23181 | -2.72 |
| XM_926370.1 | LOC642989 | ILMN_33765 | -2.73 |
| NM_002225.2 | IVD | ILMN_13293 | -2.75 |
| NM_005723.2 | TSPAN5 | ILMN_8032 | -2.75 |
| NM_079837.2 | BANP | ILMN_8638 | -2.76 |
| NM_198391.1 | FLRT3 | ILMN_23273 | -2.76 |
| NM_024516.2 | C16orf53 | ILMN_20272 | -2.76 |
| NM_018164.1 | C12orf11 | ILMN_14707 | -2.76 |
| NM_017895.6 | DDX27 | ILMN_20732 | -2.77 |
| NM_016048.1 | ISOC1 | ILMN_15311 | -2.78 |
| NM_001042549.1 | NSL1 | ILMN_164300 | -2.79 |
| NM_006860.2 | RABL4 | ILMN_4559 | -2.83 |
| NM_005833.2 | RABEPK | ILMN_4050 | -2.83 |
| NM_002897.3 | RBMS1 | ILMN_18726 | -2.85 |
| NR_001283.1 | TOP1P2 | ILMN_4755 | -2.85 |
| XM_930884.1 | LOC653080 | ILMN_32261 | -2.86 |
| NM_022173.1 | TIA1 | ILMN_29910 | -2.86 |
| NM_020749.3 | MTUS1 | ILMN_4658 | -2.87 |
| NM_032180.1 | FLJ13305 | ILMN_5829 | -2.88 |
| NM_000856.3 | GUCY1A3 | ILMN_11680 | -2.88 |
| NM_019116.2 | UBFD1 | ILMN_179383 | -2.89 |
| NM_012241.2 | SIRT5 | ILMN_18454 | -2.9 |
| NM_022652.2 | DUSP6 | ILMN_5926 | -2.91 |
| NM_016587.2 | CBX3 | ILMN_11642 | -2.94 |
| NM_007280.1 | OIP5 | ILMN_18200 | -2.96 |
| NM_138720.1 | HIST1H2BD | ILMN_17622 | -2.96 |
| XM_936103.1 | LOC642033 | ILMN_33652 | -2.97 |
| NM_001017369.1 | SC4MOL | ILMN_2901 | -2.97 |
| NM_014254.1 | TMEM5 | ILMN_26271 | -2.97 |
| NM_020242.1 | KIF15 | ILMN_6188 | -2.97 |
| NM_080597.2 | OSBPL1A | ILMN_10951 | -2.98 |
| NM_024570.1 | RNASEH2B | ILMN_20578 | -3 |
| NM_003512.3 | HIST1H2AC | ILMN_26493 | -3 |
| NM_012433.2 | SF3B1 | ILMN_168075 | -3.06 |
| XM_930694.1 | LOC642477 | ILMN_36253 | -3.06 |
| NM_030881.2 | DDX17 | ILMN_28024 | -3.07 |
| NM_002167.2 | ID3 | ILMN_6829 | -3.08 |
| NM_032334.1 | C8orf53 | ILMN_24637 | -3.1 |
| NM_001031723.1 | DNAJB14 | ILMN_9854 | -3.13 |
| NM_004901.2 | ENTPD4 | ILMN_19012 | -3.13 |
| NM_019067.4 | GNL3L | ILMN_181682 | -3.15 |
| NM_003368.4 | USP1 | ILMN_5285 | -3.16 |
| XR_015514.1 | LOC730746 | ILMN_163533 | -3.16 |
| NM_004401.2 | DFFA | ILMN_6993 | -3.18 |
| XM_926249.2 | LOC642852 | ILMN_40586 | -3.21 |
| NM_001005849.1 | SUMO2 | ILMN_16713 | -3.22 |
| NM_153201.1 | HSPA8 | ILMN_14829 | -3.22 |
| NM_001031726.2 | C19orf12 | ILMN_10211 | -3.26 |
| NR_001449.1 | TRK1 | ILMN_6493 | -3.26 |
| NM_014612.3 | FAM120A | ILMN_14224 | -3.28 |
| NM_002086.3 | GRB2 | ILMN_173749 | -3.29 |
| NM_080723.3 | NRSN1 | ILMN_178353 | -3.35 |
| NM_003542.3 | HIST1H4C | ILMN_30043 | -3.35 |
| NM_199287.2 | CCDC137 | ILMN_309720 | -3.36 |
| NR_003287.1 | LOC100008589 | ILMN_177351 | -3.37 |
| NM_017489.1 | TERF1 | ILMN_164297 | -3.38 |
| NM_181725.2 | METTL2A | ILMN_23067 | -3.38 |
| NM_018304.2 | PRR11 | ILMN_32619 | -3.38 |
| NR_003144.1 | LOC723972 | ILMN_180363 | -3.39 |
| NM_153333.2 | TCEAL8 | ILMN_12551 | -3.39 |
| XM_934985.1 | LOC400879 | ILMN_31001 | -3.41 |
| NM_205843.1 | NFIC | ILMN_22629 | -3.44 |
| XM_938779.1 | LOC653972 | ILMN_31111 | -3.45 |
| NM_006004.1 | UQCRH | ILMN_138507 | -3.48 |
| NM_184234.1 | RBM39 | ILMN_20330 | -3.49 |
| NM_181077.2 | GOLGA8A | ILMN_2827 | -3.54 |
| XM_944321.1 | LOC402560 | ILMN_42108 | -3.54 |
| NM_003358.1 | UGCG | ILMN_26228 | -3.59 |
| NM_012234.4 | RYBP | ILMN_13259 | -3.59 |
| NM_006630.1 | ZNF234 | ILMN_29233 | -3.62 |
| NM_002568.3 | PABPC1 | ILMN_173094 | -3.63 |
| NM_024011.2 | CDC2L2 | ILMN_20434 | -3.65 |
| NM_203390.2 | RBM12B | ILMN_174962 | -3.69 |
| NM_174942.1 | GAS2L3 | ILMN_5609 | -3.7 |
| NM_006182.2 | DDR2 | ILMN_20698 | -3.74 |
| NM_002093.2 | GSK3B | ILMN_7421 | -3.75 |
| XM_928075.2 | LOC643287 | ILMN_37869 | -3.76 |
| NM_001438.2 | ESRRG | ILMN_29221 | -3.82 |
| NM_005905.3 | SMAD9 | ILMN_28187 | -3.89 |
| XM_935818.1 | FLJ20397 | ILMN_137080 | -3.9 |
| NM_002763.3 | PROX1 | ILMN_177185 | -3.93 |
| NM_016618.1 | KRCC1 | ILMN_25337 | -3.96 |
| XM_929738.1 | LOC646786 | ILMN_38919 | -3.98 |
| NM_004257.3 | TGFBRAP1 | ILMN_30176 | -4.1 |
| NM_004316.2 | ASCL1 | ILMN_23892 | -4.11 |
| NM_001040142.1 | SCN2A | ILMN_167124 | -4.17 |
| NM_001099285.1 | PTMA | ILMN_306831 | -4.2 |
| NM_001007157.1 | PHF14 | ILMN_2096 | -4.25 |
| NM_016374.5 | ARID4B | ILMN_162934 | -4.29 |
| XM_001129423.1 | LOC729137 | ILMN_166772 | -4.31 |
| NM_006630.1 | ZNF234 | ILMN_29233 | -4.35 |
| XM_933956.1 | LOC644162 | ILMN_43225 | -4.35 |
| NM_003344.2 | UBE2H | ILMN_163352 | -4.39 |
| NM_178439.3 | GMCL1 | ILMN_3285 | -4.39 |
| NM_002166.4 | ID2 | ILMN_28481 | -4.41 |
| NM_178439.3 | GMCL1 | ILMN_3285 | -4.49 |
| NM_022731.2 | NUCKS1 | ILMN_17108 | -4.51 |
| NM_004175.3 | SNRPD3 | ILMN_163179 | -4.54 |
| NM_002166.4 | ID2 | ILMN_28481 | -4.56 |
| XM_498571.2 | LOC440160 | ILMN_33035 | -4.58 |
| NM_020449.2 | THOC2 | ILMN_162047 | -4.59 |
| NM_006717.2 | SPIN1 | ILMN_23742 | -4.75 |
| NM_016374.5 | ARID4B | ILMN_162934 | -4.78 |
| NR_003041.1 | SNORD13 | ILMN_168446 | -4.91 |
| NM_138444.3 | KCTD12 | ILMN_18501 | -4.91 |
| XM_930284.1 | LOC441763 | ILMN_36192 | -4.94 |
| NM_001039703.1 | NBPF10 | ILMN_45673 | -5 |
| NM_001034841.2 | LOC162073 | ILMN_3559 | -5.01 |
| NM_005346.3 | HSPA1B | ILMN_25549 | -5.18 |
| NR_001445.1 | RN7SK | ILMN_14457 | -5.22 |
| NM_078629.1 | MSL3L1 | ILMN_29354 | -5.24 |
| NM_004456.3 | EZH2 | ILMN_25740 | -5.28 |
| NM_014498.2 | GOLPH4 | ILMN_179486 | -5.35 |
| NM_006265.1 | RAD21 | ILMN_171453 | -5.44 |
| NM_001037675.1 | NBPF20 | ILMN_26956 | -5.75 |
| NM_005345.4 | HSPA1A | ILMN_6623 | -7.21 |
