# Supplementary material for: Minimal Peroxide Exposure of Neuronal Cells Induces Multifaceted Adaptive Responses
Source: PLoS One. 2010 Dec 17;5(12):e14352. doi: 10.1371/journal.pone.0014352 (PMC3003681; doi:10.1371/journal.pone.0014352)
Supplement: Table S14 — BDNF-significantly regulated genes after 8 hours of stimulation in the CMP state SH-SY5Y cells. Each significantly regulated gene is described via its accession number (ACCESSION), Gene Symbol (SYMBOL), Illumina array transcript designation (TRANSCRIPT). For each gene the z-ratio of expression compared to untreated cells after 8 hours of ligand stimulation is displayed (CMP BDNF 8). (1.15 MB DOC) [file pone.0014352.s021.doc]

**Table S14. BDNF-significantly regulated genes after 8 hours of stimulation in the CMP state SH-SY5Y cells**. Each significantly regulated gene is described via its accession number (ACCESSION), Gene Symbol (SYMBOL), Illumina array transcript designation (TRANSCRIPT). For each gene the z-ratio of expression compared to un-treated cells after 8 hours of ligand stimulation is displayed (CMP BDNF 8).

| **ACCESSION** | **SYMBOL** | **TRANSCRIPT** | **CMP BDNF 8** |
| --- | --- | --- | --- |
| NM_000584.2 | IL8 | ILMN_179575 | 7.83 |
| NM_001001391.1 | CD44 | ILMN_10947 | 4.54 |
| NM_001077188.1 | HS6ST2 | ILMN_182242 | 4.45 |
| NM_001008219.1 | AMY1C | ILMN_28222 | 4 |
| NM_001033506.1 | CSTF3 | ILMN_27049 | 3.97 |
| NM_004598.3 | SPOCK1 | ILMN_25886 | 3.85 |
| NM_207035.1 | C1orf63 | ILMN_22593 | 3.8 |
| NM_024663.3 | NPEPL1 | ILMN_175218 | 3.79 |
| NM_001496.3 | GFRA3 | ILMN_8392 | 3.78 |
| NM_001040456.1 | RHBDD2 | ILMN_168345 | 3.77 |
| NM_006157.2 | NELL1 | ILMN_2560 | 3.73 |
| NM_015690.2 | STK36 | ILMN_15506 | 3.69 |
| NM_001040456.1 | RHBDD2 | ILMN_168345 | 3.53 |
| NM_020162.2 | DHX33 | ILMN_1191 | 3.51 |
| NM_001007246.1 | BRWD1 | ILMN_28841 | 3.46 |
| NM_002160.2 | TNC | ILMN_14948 | 3.45 |
| NM_001078.2 | VCAM1 | ILMN_3875 | 3.42 |
| NM_015306.1 | USP24 | ILMN_309418 | 3.42 |
| NM_152322.2 | BTBD11 | ILMN_506 | 3.35 |
| NM_022910.1 | NDRG4 | ILMN_8824 | 3.35 |
| NM_003972.2 | BTAF1 | ILMN_8616 | 3.34 |
| NM_004071.2 | CLK1 | ILMN_162592 | 3.29 |
| NM_000199.2 | SGSH | ILMN_7542 | 3.17 |
| NM_025074.4 | FRAS1 | ILMN_165073 | 3.16 |
| NM_020897.1 | HCN3 | ILMN_20127 | 3.1 |
| NM_006197.3 | PCM1 | ILMN_14472 | 3.1 |
| NM_078470.2 | COX15 | ILMN_13504 | 3.07 |
| NM_004567.2 | PFKFB4 | ILMN_163968 | 3.07 |
| NR_003491.1 | MIAT | ILMN_308315 | 3.05 |
| NM_018416.2 | FOXJ2 | ILMN_165896 | 3.03 |
| NM_005922.2 | MAP3K4 | ILMN_6743 | 2.98 |
| NM_003458.3 | BSN | ILMN_22754 | 2.97 |
| NM_003913.3 | PRPF4B | ILMN_139391 | 2.95 |
| NM_178831.4 | GATS | ILMN_18755 | 2.94 |
| NM_015433.2 | FAM119B | ILMN_17350 | 2.9 |
| NM_006045.1 | ATP9A | ILMN_176431 | 2.88 |
| NM_015308.1 | FNBP4 | ILMN_25895 | 2.87 |
| NM_019024.1 | HEATR5B | ILMN_183109 | 2.86 |
| NM_002673.3 | PLXNB1 | ILMN_22628 | 2.86 |
| NM_006421.3 | ARFGEF1 | ILMN_164295 | 2.82 |
| NM_178526.1 | SLC25A42 | ILMN_5992 | 2.81 |
| NM_024525.2 | TTC13 | ILMN_164005 | 2.81 |
| NM_203364.2 | CAPRIN1 | ILMN_9771 | 2.77 |
| NM_003119.2 | SPG7 | ILMN_26332 | 2.76 |
| NM_002975.2 | CLEC11A | ILMN_29894 | 2.73 |
| NM_005224.2 | ARID3A | ILMN_18757 | 2.72 |
| NM_015317.1 | PUM2 | ILMN_12220 | 2.7 |
| NM_017514.2 | PLXNA3 | ILMN_162939 | 2.69 |
| NM_021942.4 | C4orf41 | ILMN_8900 | 2.69 |
| NM_020133.2 | AGPAT4 | ILMN_24920 | 2.68 |
| NM_023080.1 | C8orf33 | ILMN_15901 | 2.67 |
| NM_001986.1 | ETV4 | ILMN_25435 | 2.65 |
| XM_930411.1 | LOC645099 | ILMN_37678 | 2.64 |
| NM_015878.4 | AZIN1 | ILMN_4825 | 2.62 |
| NM_014000.2 | VCL | ILMN_27566 | 2.61 |
| NM_005436.2 | CCDC6 | ILMN_19206 | 2.61 |
| NM_015477.1 | SIN3A | ILMN_14108 | 2.57 |
| NM_031469.2 | SH3BGRL2 | ILMN_9801 | 2.57 |
| NM_005385.3 | NKTR | ILMN_23378 | 2.57 |
| NM_015042.1 | ZNF609 | ILMN_28956 | 2.53 |
| NM_001080493.1 | HSZFP36 | ILMN_180853 | 2.53 |
| NM_003635.2 | NDST2 | ILMN_21220 | 2.52 |
| XM_945571.1 | ANKRD13D | ILMN_138370 | 2.52 |
| NM_001083585.1 | RABEP1 | ILMN_307418 | 2.52 |
| NM_001031712.2 | TRMT11 | ILMN_8801 | 2.52 |
| NM_001002878.1 | THOC5 | ILMN_13820 | 2.5 |
| XM_940209.1 | KIAA0194 | ILMN_37512 | 2.49 |
| NM_001077442.1 | HNRNPC | ILMN_165238 | 2.48 |
| NM_173602.2 | DIP2B | ILMN_24944 | 2.47 |
| NM_024909.1 | C6orf134 | ILMN_21139 | 2.46 |
| NM_004036.3 | ADCY3 | ILMN_26929 | 2.44 |
| NM_033426.2 | KIAA1737 | ILMN_24671 | 2.43 |
| NM_003589.2 | CUL4A | ILMN_28629 | 2.43 |
| NM_005808.2 | CTDSPL | ILMN_510 | 2.43 |
| NM_001010927.2 | TIAM2 | ILMN_9891 | 2.41 |
| NM_025132.3 | WDR19 | ILMN_11749 | 2.41 |
| NM_198480.2 | ZNF615 | ILMN_26774 | 2.4 |
| XM_001133202.1 | KIAA0363 | ILMN_166209 | 2.4 |
| NM_024612.3 | DHX40 | ILMN_1864 | 2.4 |
| NM_015346.2 | ZFYVE26 | ILMN_176163 | 2.39 |
| NM_001012643.2 | LOC339344 | ILMN_6535 | 2.39 |
| NM_144566.1 | ZNF700 | ILMN_7926 | 2.38 |
| NM_015636.3 | EIF2B4 | ILMN_18552 | 2.38 |
| NM_032830.1 | CIRH1A | ILMN_2574 | 2.38 |
| NM_033063.1 | MAP6 | ILMN_6882 | 2.37 |
| NM_002959.4 | SORT1 | ILMN_165748 | 2.36 |
| NM_006767.3 | LZTR1 | ILMN_18977 | 2.36 |
| NM_018246.2 | CCDC25 | ILMN_5229 | 2.36 |
| NM_022494.1 | ZDHHC6 | ILMN_1193 | 2.35 |
| NM_203459.1 | CAMSAP1L1 | ILMN_14735 | 2.35 |
| NM_017522.3 | LRP8 | ILMN_18319 | 2.34 |
| NM_015655.2 | ZNF337 | ILMN_3280 | 2.34 |
| NM_006011.3 | ST8SIA2 | ILMN_19287 | 2.34 |
| NM_007171.2 | POMT1 | ILMN_18145 | 2.32 |
| NM_006918.4 | SC5DL | ILMN_24287 | 2.32 |
| NM_015245.2 | ANKS1A | ILMN_25376 | 2.31 |
| NM_173797.2 | PAPD4 | ILMN_2190 | 2.31 |
| NM_152716.1 | PATL1 | ILMN_11588 | 2.31 |
| NM_153188.2 | TNPO1 | ILMN_29083 | 2.31 |
| NM_020724.1 | RNF150 | ILMN_26801 | 2.29 |
| NM_001347.2 | DGKQ | ILMN_27065 | 2.28 |
| NM_005781.4 | TNK2 | ILMN_5336 | 2.28 |
| NM_199245.1 | VAMP1 | ILMN_10901 | 2.27 |
| NM_001144.4 | AMFR | ILMN_22219 | 2.27 |
| NM_001280.1 | CIRBP | ILMN_24327 | 2.27 |
| NM_019001.2 | XRN1 | ILMN_8924 | 2.26 |
| NM_053274.2 | GLMN | ILMN_38827 | 2.26 |
| NM_152398.2 | OCIAD2 | ILMN_18246 | 2.25 |
| NM_022450.2 | RHBDF1 | ILMN_20892 | 2.24 |
| NM_003846.1 | PEX11B | ILMN_20603 | 2.24 |
| NM_018999.2 | KIAA1128 | ILMN_173411 | 2.23 |
| NM_080927.3 | DCBLD2 | ILMN_175741 | 2.23 |
| NM_004273.2 | CHST3 | ILMN_7808 | 2.23 |
| NM_020447.3 | C15orf17 | ILMN_13536 | 2.23 |
| NM_002473.3 | MYH9 | ILMN_183555 | 2.23 |
| NM_015115.1 | DCUN1D4 | ILMN_9395 | 2.23 |
| NM_015026.1 | MON2 | ILMN_19004 | 2.22 |
| NM_201281.1 | MTMR2 | ILMN_24002 | 2.22 |
| NM_015906.3 | TRIM33 | ILMN_4131 | 2.22 |
| NM_001001132.1 | ITSN1 | ILMN_10040 | 2.21 |
| NM_003475.2 | RASSF7 | ILMN_12457 | 2.21 |
| NM_002482.2 | NASP | ILMN_21654 | 2.21 |
| NM_002076.2 | GNS | ILMN_177670 | 2.21 |
| NM_032810.2 | ATAD1 | ILMN_175726 | 2.21 |
| NM_001387.2 | DPYSL3 | ILMN_23309 | 2.21 |
| NM_004402.2 | DFFB | ILMN_14684 | 2.2 |
| NM_005688.2 | ABCC5 | ILMN_25223 | 2.2 |
| NM_181673.1 | OGT | ILMN_4866 | 2.2 |
| NM_001017391.1 | SULT1A4 | ILMN_20533 | 2.2 |
| NM_002609.3 | PDGFRB | ILMN_25767 | 2.2 |
| NM_002890.1 | RASA1 | ILMN_26241 | 2.2 |
| NM_014614.1 | PSME4 | ILMN_164803 | 2.2 |
| NM_001002878.1 | THOC5 | ILMN_13820 | 2.2 |
| NM_134426.2 | SLC26A6 | ILMN_5076 | 2.19 |
| NM_022497.3 | MRPS25 | ILMN_20500 | 2.19 |
| NM_014805.2 | EPM2AIP1 | ILMN_9770 | 2.19 |
| NM_019024.1 | HEATR5B | ILMN_25274 | 2.18 |
| NM_014487.3 | ZNF330 | ILMN_6878 | 2.18 |
| NM_016121.3 | KCTD3 | ILMN_179202 | 2.18 |
| XM_001132754.1 | LOC728734 | ILMN_169578 | 2.17 |
| NM_001092.3 | ABR | ILMN_23502 | 2.17 |
| NM_014071.2 | NCOA6 | ILMN_20599 | 2.16 |
| NM_014603.1 | CDR2L | ILMN_26231 | 2.16 |
| XM_371461.4 | KIAA1671 | ILMN_42090 | 2.15 |
| NM_152511.3 | DUSP18 | ILMN_9044 | 2.15 |
| NM_002158.3 | FOXN2 | ILMN_167513 | 2.15 |
| NM_181722.2 | LOC285908 | ILMN_180740 | 2.14 |
| NM_006715.2 | MAN2C1 | ILMN_685 | 2.14 |
| NM_018343.1 | RIOK2 | ILMN_16482 | 2.14 |
| NM_000743.2 | CHRNA3 | ILMN_23268 | 2.14 |
| NM_016284.3 | CNOT1 | ILMN_169268 | 2.14 |
| NM_018072.4 | HEATR1 | ILMN_7614 | 2.14 |
| NM_001095.2 | ACCN2 | ILMN_27416 | 2.13 |
| NM_020246.2 | SLC12A9 | ILMN_12081 | 2.13 |
| NM_020414.3 | DDX24 | ILMN_10146 | 2.13 |
| NM_138452.1 | DHRS1 | ILMN_15545 | 2.12 |
| NM_001032293.2 | ZNF207 | ILMN_21705 | 2.12 |
| NM_001406.3 | EFNB3 | ILMN_17706 | 2.12 |
| NM_133340.1 | RAD17 | ILMN_4423 | 2.11 |
| NM_018330.4 | KIAA1598 | ILMN_4741 | 2.11 |
| NM_025058.3 | TRIM46 | ILMN_18492 | 2.11 |
| NM_014853.2 | SGSM2 | ILMN_9226 | 2.11 |
| NM_006285.2 | TESK1 | ILMN_5444 | 2.1 |
| NM_181782.2 | NCOA7 | ILMN_9525 | 2.1 |
| NM_002268.3 | KPNA4 | ILMN_21107 | 2.1 |
| NM_001023567.2 | GOLGA8B | ILMN_14405 | 2.1 |
| NM_173602.2 | DIP2B | ILMN_179302 | 2.09 |
| NM_001008408.3 | RBM33 | ILMN_165407 | 2.09 |
| NM_001304.3 | CPD | ILMN_163103 | 2.09 |
| NM_133471.1 | KIAA1949 | ILMN_308966 | 2.09 |
| NM_005109.2 | OXSR1 | ILMN_13172 | 2.09 |
| NM_014666.2 | CLINT1 | ILMN_12809 | 2.09 |
| NM_000271.3 | NPC1 | ILMN_30618 | 2.08 |
| NM_003047.2 | SLC9A1 | ILMN_166750 | 2.08 |
| NM_004055.4 | CAPN5 | ILMN_30845 | 2.08 |
| NM_080730.2 | IFFO | ILMN_42149 | 2.08 |
| NM_001068.2 | TOP2B | ILMN_7099 | 2.08 |
| NM_000787.3 | DBH | ILMN_25962 | 2.08 |
| NM_003342.4 | UBE2G1 | ILMN_179729 | 2.07 |
| NM_004423.3 | DVL3 | ILMN_11726 | 2.07 |
| NM_007055.2 | POLR3A | ILMN_1449 | 2.07 |
| NM_024519.2 | FAM65A | ILMN_17641 | 2.07 |
| NM_032590.3 | FBXL10 | ILMN_19365 | 2.07 |
| NM_014747.2 | RIMS3 | ILMN_21581 | 2.07 |
| NM_016472.3 | C14orf129 | ILMN_7725 | 2.06 |
| NM_002035.1 | FVT1 | ILMN_5671 | 2.05 |
| NM_013243.2 | SCG3 | ILMN_174345 | 2.05 |
| NM_001006657.1 | WDR35 | ILMN_175554 | 2.05 |
| NM_138967.2 | SCAMP5 | ILMN_3255 | 2.05 |
| NM_025133.3 | FBXO11 | ILMN_18553 | 2.05 |
| NM_003462.3 | DNALI1 | ILMN_24038 | 2.04 |
| NM_030808.3 | NDEL1 | ILMN_20362 | 2.04 |
| NM_175859.1 | CTPS2 | ILMN_8874 | 2.04 |
| NM_003565.1 | ULK1 | ILMN_2158 | 2.04 |
| NM_006426.1 | DPYSL4 | ILMN_175746 | 2.04 |
| NM_015352.1 | POFUT1 | ILMN_7876 | 2.03 |
| NM_006083.3 | IK | ILMN_27338 | 2.03 |
| NM_032620.1 | GTPBP3 | ILMN_13264 | 2.03 |
| NM_005506.2 | SCARB2 | ILMN_12802 | 2.03 |
| NM_020789.2 | IGSF9 | ILMN_22415 | 2.02 |
| NM_006465.2 | ARID3B | ILMN_4032 | 2.02 |
| NM_015157.1 | PHLDB1 | ILMN_3997 | 2.02 |
| NM_144781.1 | PDCD2 | ILMN_16269 | 2.02 |
| NM_003086.2 | SNAPC4 | ILMN_180505 | 2.02 |
| NM_002319.2 | LRCH4 | ILMN_139402 | 2.01 |
| NM_001031623.2 | ZNF451 | ILMN_990 | 2.01 |
| NM_138774.2 | C19orf22 | ILMN_15785 | 2.01 |
| NM_002451.3 | MTAP | ILMN_163674 | 2.01 |
| NM_080927.3 | DCBLD2 | ILMN_175741 | 2.01 |
| NM_016282.2 | AK3 | ILMN_6776 | 2.01 |
| NM_002162.2 | ICAM3 | ILMN_9707 | 2 |
| NM_002972.1 | SBF1 | ILMN_22729 | 2 |
| NM_015455.3 | CNOT6 | ILMN_17926 | 2 |
| NM_052897.3 | MBD6 | ILMN_162772 | 2 |
| NM_006380.2 | APPBP2 | ILMN_170911 | 2 |
| NM_015144.2 | ZCCHC14 | ILMN_32176 | 2 |
| NM_018044.2 | NSUN5 | ILMN_895 | 1.99 |
| NM_024048.2 | MGC3020 | ILMN_29369 | 1.99 |
| NM_014984.2 | AZI1 | ILMN_3856 | 1.99 |
| NM_139353.1 | TAF1C | ILMN_4122 | 1.99 |
| NM_006148.1 | LASP1 | ILMN_27039 | 1.99 |
| NM_015902.4 | UBR5 | ILMN_178959 | 1.98 |
| NM_014867.1 | KBTBD11 | ILMN_20625 | 1.98 |
| NM_014701.2 | KIAA0256 | ILMN_23132 | 1.98 |
| NM_014141.4 | CNTNAP2 | ILMN_176606 | 1.98 |
| NM_014708.3 | KNTC1 | ILMN_25890 | 1.98 |
| NM_018263.4 | ASXL2 | ILMN_7971 | 1.98 |
| NM_001012516.1 | ITM2C | ILMN_27531 | 1.98 |
| NM_013241.2 | FHOD1 | ILMN_14837 | 1.97 |
| NM_175085.1 | GART | ILMN_19282 | 1.97 |
| NM_018054.4 | ARHGAP17 | ILMN_9156 | 1.97 |
| NM_020728.1 | FAM62B | ILMN_19173 | 1.97 |
| NM_032621.2 | BEX2 | ILMN_24134 | 1.97 |
| NM_001481.1 | GAS8 | ILMN_26809 | 1.96 |
| NM_033419.3 | PERLD1 | ILMN_12215 | 1.96 |
| NM_000292.1 | PHKA2 | ILMN_20799 | 1.96 |
| NM_006795.2 | EHD1 | ILMN_17263 | 1.96 |
| NM_016040.3 | TMED5 | ILMN_28500 | 1.96 |
| NM_032788.1 | ZNF514 | ILMN_14476 | 1.95 |
| XM_495939.3 | KIAA1545 | ILMN_40920 | 1.95 |
| NM_181050.1 | AXIN1 | ILMN_6274 | 1.95 |
| NM_006005.2 | WFS1 | ILMN_18545 | 1.95 |
| NM_025134.4 | CHD9 | ILMN_15866 | 1.95 |
| XM_001132495.1 | SLC26A11 | ILMN_167531 | 1.94 |
| NM_018566.3 | YOD1 | ILMN_19081 | 1.93 |
| NM_130463.2 | ATP6V1G2 | ILMN_18630 | 1.93 |
| NM_024698.4 | SLC25A22 | ILMN_13935 | 1.93 |
| NM_207514.1 | DEF8 | ILMN_175985 | 1.93 |
| NM_032434.2 | ZNF512 | ILMN_5859 | 1.93 |
| NM_014974.1 | DIP2C | ILMN_16576 | 1.92 |
| NM_003166.3 | SULT1A3 | ILMN_28760 | 1.92 |
| NM_016004.2 | IFT52 | ILMN_7299 | 1.92 |
| NM_000153.2 | GALC | ILMN_28156 | 1.92 |
| NM_019106.4 | 3-Sep | ILMN_4065 | 1.92 |
| NM_001042537.1 | SLC9A6 | ILMN_183311 | 1.91 |
| NM_030793.3 | FBXO38 | ILMN_4373 | 1.91 |
| NM_006145.1 | DNAJB1 | ILMN_19740 | 1.91 |
| NM_014631.2 | SH3PXD2A | ILMN_17160 | 1.91 |
| NM_144635.3 | FAM131A | ILMN_2542 | 1.91 |
| NM_206852.1 | RTN1 | ILMN_3435 | 1.91 |
| NM_018256.2 | WDR12 | ILMN_14410 | 1.91 |
| NM_024910.1 | ZNF767 | ILMN_28810 | 1.9 |
| NM_001013258.1 | ZNF789 | ILMN_11535 | 1.9 |
| NM_014817.3 | KIAA0644 | ILMN_164846 | 1.9 |
| NM_024319.2 | C1orf35 | ILMN_28904 | 1.9 |
| NM_022662.2 | ANAPC1 | ILMN_164277 | 1.9 |
| NM_207577.1 | MAP6 | ILMN_5510 | 1.9 |
| NM_001440.2 | EXTL3 | ILMN_10725 | 1.9 |
| NM_005669.4 | REEP5 | ILMN_21319 | 1.9 |
| NM_004458.1 | ACSL4 | ILMN_12915 | 1.89 |
| NM_001080485.1 | ZNF275 | ILMN_180340 | 1.89 |
| NM_001083946.1 | C2orf56 | ILMN_307130 | 1.89 |
| NM_001020658.1 | PUM1 | ILMN_169279 | 1.89 |
| XM_944439.2 | LOC653994 | ILMN_38572 | 1.88 |
| NM_139321.1 | ATRN | ILMN_6053 | 1.88 |
| NM_001013690.1 | LOC401720 | ILMN_21595 | 1.88 |
| NM_025251.1 | KIAA1688 | ILMN_28510 | 1.88 |
| NM_015447.1 | CAMSAP1 | ILMN_815 | 1.88 |
| NM_018121.2 | C10orf6 | ILMN_24540 | 1.88 |
| NM_000617.1 | SLC11A2 | ILMN_10129 | 1.88 |
| NM_015153.1 | PHF3 | ILMN_23658 | 1.88 |
| NM_003966.2 | SEMA5A | ILMN_183828 | 1.87 |
| NM_001001794.2 | FAM116B | ILMN_17332 | 1.87 |
| NM_001040439.1 | MAPK8IP3 | ILMN_174436 | 1.87 |
| NM_212469.1 | CHKA | ILMN_28401 | 1.87 |
| NM_033631.2 | LUZP1 | ILMN_2667 | 1.87 |
| NM_003906.3 | MCM3AP | ILMN_19614 | 1.87 |
| NM_014691.2 | AQR | ILMN_24923 | 1.87 |
| NM_001037163.1 | MGC12966 | ILMN_182436 | 1.87 |
| NM_013233.2 | STK39 | ILMN_19845 | 1.87 |
| NM_003234.1 | TFRC | ILMN_12909 | 1.87 |
| NM_014382.2 | ATP2C1 | ILMN_16216 | 1.86 |
| NM_006159.1 | NELL2 | ILMN_26383 | 1.86 |
| NM_012433.2 | SF3B1 | ILMN_2494 | 1.86 |
| NM_002868.2 | RAB5B | ILMN_3818 | 1.85 |
| NM_005090.2 | PLA2G4B | ILMN_6705 | 1.84 |
| NM_033389.2 | SSH2 | ILMN_8279 | 1.84 |
| NM_014946.3 | SPAST | ILMN_14993 | 1.84 |
| NM_004140.3 | LLGL1 | ILMN_18915 | 1.84 |
| NM_017806.1 | LIME1 | ILMN_28410 | 1.84 |
| NM_003161.2 | RPS6KB1 | ILMN_162232 | 1.84 |
| NM_033446.1 | FAM125B | ILMN_20760 | 1.83 |
| NM_017925.4 | DENND4C | ILMN_2455 | 1.83 |
| NM_133371.2 | MYOZ3 | ILMN_21305 | 1.83 |
| NM_018171.3 | APPL2 | ILMN_14197 | 1.83 |
| NM_002737.2 | PRKCA | ILMN_24085 | 1.83 |
| NM_014607.3 | UBXD2 | ILMN_26387 | 1.83 |
| NM_014772.1 | KIAA0427 | ILMN_182540 | 1.82 |
| NM_003184.3 | TAF2 | ILMN_177935 | 1.82 |
| NM_032421.2 | CLIP2 | ILMN_14847 | 1.82 |
| NM_199324.1 | OTUD4 | ILMN_25194 | 1.82 |
| NM_014969.4 | WDR47 | ILMN_163009 | 1.82 |
| NM_014138.3 | FAM156A | ILMN_18863 | 1.82 |
| NM_032520.3 | GNPTG | ILMN_28173 | 1.82 |
| NM_001012626.1 | LOC285074 | ILMN_21153 | 1.81 |
| NM_017810.2 | ZNF434 | ILMN_13809 | 1.81 |
| XM_290799.7 | ARHGAP23 | ILMN_162296 | 1.81 |
| NM_019591.2 | ZNF26 | ILMN_3233 | 1.81 |
| NM_013276.2 | SHPK | ILMN_22706 | 1.81 |
| NM_012419.4 | RGS17 | ILMN_25210 | 1.81 |
| XM_936495.2 | LOC647346 | ILMN_36174 | 1.81 |
| NM_032875.1 | FBXL20 | ILMN_11563 | 1.8 |
| NM_003980.3 | MAP7 | ILMN_681 | 1.8 |
| NM_173630.2 | RTTN | ILMN_5471 | 1.8 |
| NM_198925.1 | SEMA4B | ILMN_25258 | 1.8 |
| NM_005095.2 | ZMYM4 | ILMN_26259 | 1.8 |
| NM_015636.2 | EIF2B4 | ILMN_139347 | 1.8 |
| NM_133328.2 | DEDD2 | ILMN_12562 | 1.79 |
| NM_012119.3 | CCRK | ILMN_39653 | 1.79 |
| NM_001014979.1 | LOC90835 | ILMN_8821 | 1.79 |
| NM_014829.2 | DDX46 | ILMN_165992 | 1.79 |
| NM_002556.2 | OSBP | ILMN_19982 | 1.79 |
| NM_001007075.1 | KLHL5 | ILMN_414 | 1.79 |
| NM_015565.1 | ZNF294 | ILMN_5529 | 1.78 |
| NM_003434.3 | ZNF133 | ILMN_5864 | 1.78 |
| NM_003461.4 | ZYX | ILMN_2137 | 1.78 |
| NM_015942.3 | MTERFD1 | ILMN_24756 | 1.78 |
| NM_006618.3 | JARID1B | ILMN_14812 | 1.78 |
| NM_014671.1 | UBE3C | ILMN_9296 | 1.78 |
| NM_020944.2 | GBA2 | ILMN_5969 | 1.77 |
| NM_003935.3 | TOP3B | ILMN_25533 | 1.77 |
| NM_016277.3 | RAB23 | ILMN_177407 | 1.77 |
| NM_014746.2 | RNF144 | ILMN_15740 | 1.77 |
| NM_006416.3 | SLC35A1 | ILMN_23284 | 1.77 |
| NM_003565.1 | ULK1 | ILMN_2158 | 1.77 |
| NM_014014.2 | ASCC3L1 | ILMN_18834 | 1.76 |
| NM_016577.3 | RAB6B | ILMN_177099 | 1.76 |
| NM_004398.2 | DDX10 | ILMN_20779 | 1.76 |
| NM_001006115.2 | IHPK1 | ILMN_8379 | 1.76 |
| NM_002230.1 | JUP | ILMN_3789 | 1.76 |
| NM_001008239.2 | C18orf25 | ILMN_981 | 1.76 |
| NM_001008938.1 | CKAP5 | ILMN_12487 | 1.76 |
| NM_007257.4 | PNMA2 | ILMN_10930 | 1.76 |
| NM_001037533.1 | GON4L | ILMN_14180 | 1.75 |
| NM_020814.1 | 4-Mar | ILMN_1823 | 1.75 |
| NM_015316.2 | PPP1R13B | ILMN_13872 | 1.75 |
| NM_003861.1 | WDR22 | ILMN_22204 | 1.75 |
| NM_152654.2 | DAND5 | ILMN_10342 | 1.75 |
| NM_031844.2 | HNRNPU | ILMN_3074 | 1.75 |
| NM_001080392.1 | KIAA1147 | ILMN_167929 | 1.75 |
| NM_018223.1 | CHFR | ILMN_26892 | 1.75 |
| NM_024046.3 | CAMKV | ILMN_24127 | 1.75 |
| NM_001677.3 | ATP1B1 | ILMN_25542 | 1.75 |
| NR_002809.1 | LOC338799 | ILMN_15606 | 1.74 |
| NM_015500.1 | C2CD2 | ILMN_182120 | 1.74 |
| NM_207443.1 | FLJ45244 | ILMN_12880 | 1.74 |
| NM_001080453.1 | INTS1 | ILMN_173681 | 1.74 |
| NM_020773.1 | TBC1D14 | ILMN_165668 | 1.74 |
| NM_020921.3 | NIN | ILMN_172996 | 1.74 |
| NM_005964.1 | MYH10 | ILMN_23305 | 1.74 |
| NM_173854.4 | SLC41A1 | ILMN_2825 | 1.73 |
| NM_032017.1 | STK40 | ILMN_25410 | 1.73 |
| NM_018357.2 | LARP6 | ILMN_25584 | 1.73 |
| NM_030918.5 | SNX27 | ILMN_17828 | 1.73 |
| NM_024610.4 | HSPBAP1 | ILMN_23171 | 1.72 |
| NM_003621.1 | PPFIBP2 | ILMN_183115 | 1.72 |
| NM_015016.1 | MAST3 | ILMN_308510 | 1.72 |
| NM_001099270.1 | ZBTB34 | ILMN_307315 | 1.72 |
| NM_015289.2 | VPS39 | ILMN_5610 | 1.72 |
| NM_020799.2 | STAMBPL1 | ILMN_1387 | 1.72 |
| NM_007346.2 | OGFR | ILMN_12520 | 1.72 |
| NM_002771.2 | PRSS3 | ILMN_19426 | 1.72 |
| NM_005243.2 | EWSR1 | ILMN_17011 | 1.72 |
| NM_001013635.2 | LOC387856 | ILMN_30286 | 1.71 |
| NM_014982.2 | PCNX | ILMN_4895 | 1.71 |
| NM_005010.3 | NRCAM | ILMN_8955 | 1.71 |
| NM_001567.2 | INPPL1 | ILMN_20903 | 1.71 |
| NM_173546.1 | KLHDC8B | ILMN_6513 | 1.71 |
| NM_014329.3 | EDC4 | ILMN_21643 | 1.71 |
| NR_003659.1 | FAM39DP | ILMN_307683 | 1.71 |
| NM_182492.1 | LRP5L | ILMN_650 | 1.7 |
| NM_173510.1 | CCDC117 | ILMN_21814 | 1.7 |
| NM_033505.2 | SELI | ILMN_18750 | 1.7 |
| NM_016028.4 | SUV420H1 | ILMN_29861 | 1.7 |
| NM_001704.1 | BAI3 | ILMN_1159 | 1.7 |
| NM_020892.1 | DTX2 | ILMN_21612 | 1.7 |
| NM_001032287.1 | NR2C1 | ILMN_14578 | 1.7 |
| NM_018445.4 | SELS | ILMN_15886 | 1.7 |
| NM_005766.2 | FARP1 | ILMN_15608 | 1.7 |
| NM_032853.2 | MUM1 | ILMN_162947 | 1.7 |
| NM_000319.3 | PEX5 | ILMN_29393 | 1.7 |
| NM_012463.2 | ATP6V0A2 | ILMN_23163 | 1.69 |
| NM_173514.1 | SLC38A9 | ILMN_10221 | 1.69 |
| NM_005819.4 | STX6 | ILMN_180926 | 1.69 |
| NM_014975.1 | MAST1 | ILMN_14016 | 1.69 |
| NM_014689.2 | DOCK10 | ILMN_15188 | 1.69 |
| NM_001003786.1 | LYK5 | ILMN_2367 | 1.69 |
| NM_018708.2 | FEM1A | ILMN_2838 | 1.69 |
| NM_203401.1 | STMN1 | ILMN_12586 | 1.69 |
| XR_018848.1 | LOC650369 | ILMN_169499 | 1.69 |
| NM_145687.2 | MAP4K4 | ILMN_28871 | 1.68 |
| NM_153812.1 | PHF13 | ILMN_27355 | 1.68 |
| NM_004428.2 | EFNA1 | ILMN_14320 | 1.68 |
| NM_003131.2 | SRF | ILMN_22299 | 1.68 |
| NM_005088.2 | SFRS17A | ILMN_26209 | 1.68 |
| NM_005207.2 | CRKL | ILMN_165503 | 1.68 |
| NM_005112.4 | WDR1 | ILMN_14401 | 1.68 |
| NM_001692.3 | ATP6V1B1 | ILMN_28016 | 1.67 |
| NM_006997.2 | TACC2 | ILMN_16130 | 1.67 |
| NM_014700.2 | RAB11FIP3 | ILMN_7754 | 1.67 |
| NM_172358.1 | CD46 | ILMN_4413 | 1.67 |
| NM_015164.1 | PLEKHM2 | ILMN_308799 | 1.67 |
| NM_032172.1 | USP42 | ILMN_162869 | 1.67 |
| NM_004698.1 | PRPF3 | ILMN_6388 | 1.67 |
| NM_033428.1 | C9orf123 | ILMN_176681 | 1.66 |
| NM_024077.3 | SECISBP2 | ILMN_19156 | 1.66 |
| NM_022371.3 | TOR3A | ILMN_21685 | 1.66 |
| NM_021168.2 | RAB40C | ILMN_22367 | 1.66 |
| NM_079837.2 | BANP | ILMN_8638 | 1.66 |
| XM_001127981.1 | LOC728014 | ILMN_169164 | 1.65 |
| NM_015577.1 | RAI14 | ILMN_4560 | 1.65 |
| NM_005927.3 | MFAP3 | ILMN_14961 | 1.65 |
| XM_001133677.1 | LOC729264 | ILMN_170805 | 1.65 |
| NM_016219.2 | MAN1B1 | ILMN_27649 | 1.65 |
| NM_006695.3 | RUNDC3A | ILMN_9016 | 1.65 |
| NM_019852.3 | METTL3 | ILMN_13907 | 1.65 |
| NM_012208.2 | HARS2 | ILMN_12435 | 1.65 |
| NM_003406.2 | YWHAZ | ILMN_11028 | 1.65 |
| NM_003818.2 | CDS2 | ILMN_18323 | 1.64 |
| NM_032323.1 | TMEM79 | ILMN_13555 | 1.64 |
| NM_144664.3 | FAM76B | ILMN_22478 | 1.64 |
| NM_018257.1 | PCMTD2 | ILMN_4945 | 1.64 |
| NM_003292.2 | TPR | ILMN_179238 | 1.64 |
| NM_018846.2 | KLHL7 | ILMN_21425 | 1.63 |
| NM_207660.2 | ZC3H14 | ILMN_23275 | 1.63 |
| NM_021090.3 | MTMR3 | ILMN_27578 | 1.63 |
| NM_080491.1 | GAB2 | ILMN_3317 | 1.63 |
| NM_022307.1 | ICA1 | ILMN_5081 | 1.63 |
| NM_015318.2 | ARHGEF18 | ILMN_4153 | 1.63 |
| NM_020362.3 | C1orf128 | ILMN_14240 | 1.63 |
| NM_080604.1 | TJAP1 | ILMN_565 | 1.63 |
| NM_014422.2 | PIB5PA | ILMN_8156 | 1.62 |
| NM_001013649.1 | LOC388969 | ILMN_138621 | 1.62 |
| NM_001079514.1 | UBN1 | ILMN_172742 | 1.62 |
| NM_133496.3 | SLC30A7 | ILMN_20389 | 1.62 |
| NM_016841.2 | MAPT | ILMN_3284 | 1.61 |
| NM_032444.2 | BTBD12 | ILMN_23717 | 1.61 |
| NM_016018.4 | PHF20L1 | ILMN_164472 | 1.61 |
| NM_022494.1 | ZDHHC6 | ILMN_1193 | 1.61 |
| NM_030806.3 | C1orf21 | ILMN_26434 | 1.61 |
| NM_032424.1 | KIAA1826 | ILMN_22604 | 1.61 |
| NM_014791.2 | MELK | ILMN_161976 | 1.61 |
| NM_005255.1 | GAK | ILMN_21151 | 1.61 |
| NM_003385.4 | VSNL1 | ILMN_14653 | 1.6 |
| NM_177972.1 | TUB | ILMN_11520 | 1.6 |
| NM_003166.3 | SULT1A3 | ILMN_28760 | 1.6 |
| NM_007271.2 | STK38 | ILMN_8385 | 1.6 |
| NM_003799.1 | RNMT | ILMN_23400 | 1.6 |
| NM_133443.1 | GPT2 | ILMN_5354 | 1.6 |
| NM_004444.4 | EPHB4 | ILMN_11176 | 1.59 |
| NM_007171.3 | POMT1 | ILMN_18145 | 1.59 |
| NM_005920.2 | MEF2D | ILMN_3465 | 1.59 |
| NM_002926.3 | RGS12 | ILMN_161894 | 1.59 |
| NM_004206.2 | SEC22C | ILMN_15895 | 1.59 |
| NM_005147.3 | DNAJA3 | ILMN_10747 | 1.59 |
| NM_018170.3 | P15RS | ILMN_174036 | 1.59 |
| NM_020215.2 | C14orf132 | ILMN_29055 | 1.59 |
| NM_003819.2 | PABPC4 | ILMN_18446 | 1.59 |
| XM_001127981.1 | LOC728014 | ILMN_169164 | 1.58 |
| NM_014811.3 | KIAA0649 | ILMN_9360 | 1.58 |
| NM_021070.2 | LTBP3 | ILMN_918 | 1.58 |
| NM_014862.3 | ARNT2 | ILMN_13881 | 1.58 |
| NM_016437.1 | TUBG2 | ILMN_13533 | 1.58 |
| NM_017991.3 | FLJ10081 | ILMN_469 | 1.58 |
| NM_001845.4 | COL4A1 | ILMN_24359 | 1.58 |
| NM_023080.1 | C8orf33 | ILMN_15901 | 1.58 |
| NM_021136.2 | RTN1 | ILMN_174587 | 1.58 |
| NM_133482.1 | RAD50 | ILMN_13599 | 1.57 |
| NM_153451.2 | ORAOV1 | ILMN_5733 | 1.57 |
| XM_497029.2 | LOC441408 | ILMN_31941 | 1.57 |
| NM_003130.2 | SRI | ILMN_170642 | 1.57 |
| NM_004467.3 | FGL1 | ILMN_25289 | 1.57 |
| NM_004332.1 | BPHL | ILMN_27041 | 1.57 |
| NM_003217.2 | TEGT | ILMN_21150 | 1.57 |
| NM_017566.2 | KLHDC4 | ILMN_8527 | 1.56 |
| NM_153331.2 | KCTD6 | ILMN_15146 | 1.56 |
| NM_174891.3 | C14orf79 | ILMN_22555 | 1.56 |
| NM_017412.2 | FZD3 | ILMN_18644 | 1.56 |
| NM_014906.3 | PPM1E | ILMN_27552 | 1.56 |
| NM_022742.3 | CCDC136 | ILMN_183005 | 1.56 |
| NM_022495.5 | C14orf135 | ILMN_25282 | 1.56 |
| NM_152424.1 | FLJ39827 | ILMN_19358 | 1.55 |
| NM_207112.1 | HAGHL | ILMN_15830 | 1.55 |
| NM_007200.3 | AKAP13 | ILMN_28017 | 1.55 |
| NM_003183.4 | ADAM17 | ILMN_165100 | 1.55 |
| NM_021203.2 | SRPRB | ILMN_2452 | 1.55 |
| NM_000859.1 | HMGCR | ILMN_165658 | 1.55 |
| NM_014765.1 | TOMM20 | ILMN_20433 | 1.55 |
| NM_001009555.2 | SH3D19 | ILMN_9084 | 1.54 |
| NM_078481.2 | CD97 | ILMN_26363 | 1.54 |
| NM_015088.2 | TNRC6B | ILMN_18527 | 1.54 |
| NM_144607.3 | CYB5D1 | ILMN_26624 | 1.54 |
| XM_945430.1 | SSR2 | ILMN_138339 | 1.54 |
| NM_013254.2 | TBK1 | ILMN_11417 | 1.54 |
| NM_007185.3 | TNRC4 | ILMN_8091 | 1.54 |
| NM_025204.2 | TRABD | ILMN_25172 | 1.54 |
| NM_022451.9 | NOC3L | ILMN_11360 | 1.54 |
| NM_003136.2 | SRP54 | ILMN_6959 | 1.54 |
| NM_002477.1 | MYL5 | ILMN_21416 | 1.53 |
| NM_173685.1 | NSMCE2 | ILMN_15101 | 1.53 |
| XM_379215.2 | LOC132241 | ILMN_37830 | 1.53 |
| NM_004818.2 | DDX23 | ILMN_162862 | 1.53 |
| NM_006123.2 | IDS | ILMN_17605 | 1.53 |
| NM_000123.2 | ERCC5 | ILMN_13388 | 1.53 |
| NM_005419.2 | STAT2 | ILMN_19030 | 1.53 |
| NM_001040101.1 | D4S234E | ILMN_173747 | 1.53 |
| NM_001005744.1 | NUMB | ILMN_27669 | 1.52 |
| NM_001012398.1 | AKTIP | ILMN_18535 | 1.52 |
| NM_018469.3 | TEX2 | ILMN_27579 | 1.52 |
| NM_018249.4 | CDK5RAP2 | ILMN_9876 | 1.52 |
| NM_005730.3 | CTDSP2 | ILMN_169361 | 1.52 |
| NM_015509.2 | NECAP1 | ILMN_27077 | 1.52 |
| NM_014634.2 | PPM1F | ILMN_14794 | 1.52 |
| NM_015330.1 | SPECC1L | ILMN_168707 | 1.52 |
| NM_003171.2 | SUPV3L1 | ILMN_23318 | 1.51 |
| NM_017921.1 | NPLOC4 | ILMN_12904 | 1.51 |
| NM_003185.3 | TAF4 | ILMN_167910 | 1.51 |
| NM_001008566.1 | TPST2 | ILMN_13248 | 1.51 |
| NM_024653.3 | PRKRIP1 | ILMN_13077 | 1.51 |
| NM_002744.4 | PRKCZ | ILMN_21284 | 1.51 |
| NM_017896.2 | C20orf11 | ILMN_27220 | 1.51 |
| NM_003664.3 | AP3B1 | ILMN_4527 | 1.51 |
| NM_016121.3 | KCTD3 | ILMN_179202 | 1.51 |
| NM_005827.1 | SLC35B1 | ILMN_17389 | 1.51 |
| NM_032308.1 | RPAIN | ILMN_15409 | 1.51 |
| NM_173798.2 | ZCCHC12 | ILMN_7344 | 1.5 |
| NM_052917.2 | GALNT13 | ILMN_180483 | 1.5 |
| NM_014917.2 | NTNG1 | ILMN_2708 | 1.5 |
| NM_018697.3 | LANCL2 | ILMN_920 | 1.5 |
| NM_001071775.1 | LOC440145 | ILMN_163591 | -1.5 |
| NM_001031677.2 | RAB24 | ILMN_25731 | -1.5 |
| NM_030660.2 | ATXN3 | ILMN_12637 | -1.51 |
| NM_176866.2 | PPA2 | ILMN_15275 | -1.51 |
| NM_002350.1 | LYN | ILMN_10095 | -1.51 |
| NM_007155.4 | ZP3 | ILMN_17555 | -1.52 |
| XM_173119.5 | LOC255130 | ILMN_42241 | -1.52 |
| NM_016055.4 | MRPL48 | ILMN_24933 | -1.52 |
| NM_014241.3 | PTPLA | ILMN_24983 | -1.52 |
| NM_018847.2 | KLHL9 | ILMN_20376 | -1.52 |
| NM_002129.2 | HMGB2 | ILMN_3200 | -1.52 |
| NM_001037633.1 | SIL1 | ILMN_10838 | -1.53 |
| NM_024598.2 | C16orf57 | ILMN_11644 | -1.53 |
| NM_012067.2 | AKR7A3 | ILMN_173325 | -1.53 |
| NM_000820.1 | GAS6 | ILMN_10723 | -1.53 |
| NM_006392.2 | NOL5A | ILMN_13841 | -1.53 |
| NM_053050.2 | MRPL53 | ILMN_25576 | -1.53 |
| NM_001080484.1 | KIAA1751 | ILMN_180591 | -1.53 |
| NM_005022.2 | PFN1 | ILMN_2354 | -1.53 |
| NM_005326.4 | HAGH | ILMN_22401 | -1.54 |
| NM_145255.2 | MRPL10 | ILMN_19178 | -1.54 |
| NM_001031717.2 | CRELD1 | ILMN_14216 | -1.54 |
| NM_017915.2 | C12orf48 | ILMN_42497 | -1.54 |
| NM_004879.3 | EI24 | ILMN_8791 | -1.54 |
| NM_206861.1 | TACC2 | ILMN_24004 | -1.55 |
| NM_004378.1 | CRABP1 | ILMN_12739 | -1.55 |
| NM_018122.3 | DARS2 | ILMN_183877 | -1.55 |
| NM_031208.1 | FAHD1 | ILMN_24688 | -1.55 |
| NM_005331.3 | HBQ1 | ILMN_24244 | -1.55 |
| NM_170738.1 | MRPL11 | ILMN_4211 | -1.55 |
| NM_003135.1 | SRP19 | ILMN_5419 | -1.55 |
| NM_000820.1 | GAS6 | ILMN_10723 | -1.55 |
| NM_199246.1 | CCNG1 | ILMN_9417 | -1.55 |
| NM_024301.3 | FKRP | ILMN_173675 | -1.56 |
| NM_002840.3 | PTPRF | ILMN_171198 | -1.56 |
| NM_001080540.1 | ISCA1L | ILMN_179661 | -1.56 |
| NM_001914.2 | CYB5A | ILMN_25182 | -1.56 |
| NM_006449.3 | CDC42EP3 | ILMN_166034 | -1.56 |
| NM_002805.4 | PSMC5 | ILMN_14538 | -1.56 |
| NM_014459.2 | PCDH17 | ILMN_5341 | -1.57 |
| NM_013412.1 | RABL2A | ILMN_12484 | -1.57 |
| NM_018229.2 | C14orf108 | ILMN_180528 | -1.57 |
| NM_004885.1 | NPFFR2 | ILMN_20676 | -1.57 |
| NM_175901.3 | LOC283932 | ILMN_1857 | -1.57 |
| NR_002207.1 | CSNK2A1P | ILMN_16686 | -1.57 |
| NM_003707.1 | RUVBL1 | ILMN_16596 | -1.57 |
| NM_001030.3 | RPS27 | ILMN_5932 | -1.57 |
| XM_377933.3 | LOC402251 | ILMN_45342 | -1.57 |
| NM_153768.1 | CABYR | ILMN_9439 | -1.58 |
| NM_006713.2 | SUB1 | ILMN_27043 | -1.58 |
| NR_003273.1 | SRP14P1 | ILMN_173927 | -1.58 |
| NM_181800.1 | UBE2C | ILMN_25999 | -1.58 |
| NM_032138.3 | KBTBD7 | ILMN_181309 | -1.59 |
| NM_020310.2 | MNT | ILMN_21283 | -1.59 |
| NM_005371.4 | METTL1 | ILMN_178633 | -1.59 |
| NM_017835.1 | C21orf59 | ILMN_12103 | -1.59 |
| NM_012460.2 | TIMM9 | ILMN_9968 | -1.59 |
| NM_203433.1 | PSMG1 | ILMN_13497 | -1.59 |
| NM_006022.2 | TSC22D1 | ILMN_26720 | -1.59 |
| NM_000108.3 | DLD | ILMN_168272 | -1.59 |
| NM_003852.3 | TRIM24 | ILMN_26118 | -1.6 |
| NM_054016.1 | FUSIP1 | ILMN_30145 | -1.6 |
| NM_006294.2 | UQCRB | ILMN_26282 | -1.6 |
| NM_001168.2 | BIRC5 | ILMN_20443 | -1.6 |
| NM_001970.3 | EIF5A | ILMN_26100 | -1.6 |
| NM_018718.1 | TSGA14 | ILMN_11000 | -1.61 |
| NM_001014832.1 | PAK4 | ILMN_24614 | -1.61 |
| NM_001080546.1 | LOC219854 | ILMN_168339 | -1.61 |
| NM_018243.2 | 11-Sep | ILMN_27161 | -1.61 |
| NM_198189.2 | COPS8 | ILMN_176757 | -1.61 |
| NM_005721.3 | ACTR3 | ILMN_11792 | -1.61 |
| XM_001126211.1 | LOC727761 | ILMN_162963 | -1.61 |
| NR_001561.1 | CYCSL1 | ILMN_10377 | -1.61 |
| NM_004763.3 | ITGB1BP1 | ILMN_17602 | -1.62 |
| XM_926231.1 | P704P | ILMN_36679 | -1.63 |
| NM_001382.2 | DPAGT1 | ILMN_10306 | -1.63 |
| NM_080655.1 | C9orf30 | ILMN_15668 | -1.63 |
| NM_053053.2 | TADA1L | ILMN_25791 | -1.63 |
| NM_006196.2 | PCBP1 | ILMN_7900 | -1.63 |
| NM_006112.2 | PPIE | ILMN_12919 | -1.64 |
| NM_000076.1 | CDKN1C | ILMN_20689 | -1.64 |
| NM_013262.3 | MYLIP | ILMN_178445 | -1.64 |
| NM_001040668.1 | BCL2L12 | ILMN_177176 | -1.64 |
| NM_020192.2 | C7orf36 | ILMN_6410 | -1.64 |
| NM_033064.3 | ATCAY | ILMN_27014 | -1.65 |
| NM_001031711.1 | ERGIC1 | ILMN_7272 | -1.65 |
| NM_016824.3 | ADD3 | ILMN_3908 | -1.65 |
| NM_006769.2 | LMO4 | ILMN_183982 | -1.65 |
| NM_058246.3 | DNAJB6 | ILMN_7651 | -1.65 |
| NM_181702.1 | GEM | ILMN_16170 | -1.65 |
| NM_013338.3 | ALG5 | ILMN_10548 | -1.65 |
| NM_001873.1 | CPE | ILMN_12337 | -1.65 |
| XM_930344.2 | LOC644934 | ILMN_43758 | -1.65 |
| XM_944104.2 | LOC653232 | ILMN_41197 | -1.65 |
| NM_023077.1 | C1orf163 | ILMN_14119 | -1.66 |
| NM_004544.2 | NDUFA10 | ILMN_7463 | -1.66 |
| NM_001006684.1 | TCEAL8 | ILMN_12677 | -1.66 |
| NM_012207.1 | HNRPH3 | ILMN_8162 | -1.66 |
| NM_002266.2 | KPNA2 | ILMN_14206 | -1.66 |
| NM_001017963.1 | HSP90AA1 | ILMN_16669 | -1.67 |
| NM_024482.1 | GMEB1 | ILMN_20915 | -1.68 |
| NM_001031726.2 | C19orf12 | ILMN_10211 | -1.68 |
| NM_001625.2 | AK2 | ILMN_1688 | -1.69 |
| NR_001562.1 | ANXA2P1 | ILMN_10494 | -1.69 |
| NM_015523.2 | REXO2 | ILMN_15016 | -1.69 |
| NM_018840.2 | C20orf24 | ILMN_10676 | -1.69 |
| NM_004792.2 | PPIG | ILMN_24595 | -1.7 |
| NM_024069.2 | C19orf50 | ILMN_13066 | -1.7 |
| NM_001042678.1 | RHOC | ILMN_162499 | -1.7 |
| NM_024710.1 | ISOC2 | ILMN_27084 | -1.7 |
| NM_001878.2 | CRABP2 | ILMN_16252 | -1.7 |
| NM_006807.3 | CBX1 | ILMN_162583 | -1.7 |
| NM_001412.3 | EIF1AX | ILMN_22164 | -1.7 |
| NM_138501.4 | GPSN2 | ILMN_6454 | -1.7 |
| NM_177983.1 | PPM1G | ILMN_878 | -1.71 |
| NM_032928.2 | TMEM141 | ILMN_27026 | -1.71 |
| NM_012343.3 | NNT | ILMN_183201 | -1.71 |
| NM_014078.4 | MRPL13 | ILMN_17393 | -1.71 |
| NM_003776.2 | MRPL40 | ILMN_21771 | -1.71 |
| NM_006854.3 | KDELR2 | ILMN_1810 | -1.71 |
| NM_145806.2 | ZNF511 | ILMN_15566 | -1.71 |
| XM_929420.1 | LOC653377 | ILMN_43949 | -1.71 |
| XM_939726.2 | LOC388532 | ILMN_45940 | -1.71 |
| NM_005680.1 | TAF1B | ILMN_13234 | -1.72 |
| NM_032829.1 | C12orf34 | ILMN_22158 | -1.72 |
| NM_004870.2 | MPDU1 | ILMN_28782 | -1.72 |
| NM_032026.1 | TATDN1 | ILMN_17501 | -1.72 |
| NM_170711.1 | DAZAP1 | ILMN_8299 | -1.72 |
| XM_001133534.1 | ATP1B3 | ILMN_163124 | -1.72 |
| NR_002190.1 | SUMO1P3 | ILMN_16906 | -1.72 |
| XM_939687.2 | LOC653658 | ILMN_33948 | -1.72 |
| NM_014914.2 | CENTG2 | ILMN_138241 | -1.73 |
| NM_005413.1 | SIX3 | ILMN_26476 | -1.73 |
| NM_003766.2 | BECN1 | ILMN_27297 | -1.73 |
| XM_929199.1 | LOC644250 | ILMN_30796 | -1.73 |
| NM_198391.1 | FLRT3 | ILMN_23273 | -1.73 |
| NM_017917.2 | PPP2R3C | ILMN_23821 | -1.73 |
| NM_002157.1 | HSPE1 | ILMN_2612 | -1.73 |
| NM_032312.2 | YIPF4 | ILMN_16255 | -1.74 |
| NM_003750.2 | EIF3A | ILMN_25761 | -1.74 |
| NM_002154.3 | HSPA4 | ILMN_166427 | -1.75 |
| NM_152344.2 | LSM12 | ILMN_1510 | -1.75 |
| NM_025233.5 | COASY | ILMN_13627 | -1.75 |
| NM_000551.2 | VHL | ILMN_21046 | -1.75 |
| NM_018369.1 | DEPDC1B | ILMN_16725 | -1.75 |
| NM_016097.3 | IER3IP1 | ILMN_21844 | -1.75 |
| NM_001014812.1 | FAM96A | ILMN_13780 | -1.75 |
| NM_024104.3 | C19orf42 | ILMN_26408 | -1.75 |
| NM_001924.2 | GADD45A | ILMN_17355 | -1.75 |
| NM_207350.1 | MGC72104 | ILMN_26269 | -1.75 |
| NM_020529.1 | NFKBIA | ILMN_6745 | -1.75 |
| NM_178812.2 | MTDH | ILMN_8610 | -1.76 |
| NM_000051.3 | ATM | ILMN_162851 | -1.76 |
| NM_022767.2 | ISG20L1 | ILMN_12401 | -1.76 |
| NM_181077.2 | GOLGA8A | ILMN_2827 | -1.77 |
| NM_006327.2 | TIMM23 | ILMN_22871 | -1.77 |
| NM_052969.1 | RPL39L | ILMN_26587 | -1.77 |
| NM_001031.4 | RPS28 | ILMN_992 | -1.77 |
| NM_003860.2 | BANF1 | ILMN_13154 | -1.78 |
| NM_003404.3 | YWHAB | ILMN_17127 | -1.78 |
| NM_019071.2 | ING3 | ILMN_177083 | -1.78 |
| NM_001008405.1 | BCAP29 | ILMN_24800 | -1.78 |
| NM_000230.1 | LEP | ILMN_10827 | -1.78 |
| NM_001031706.1 | PLEKHB2 | ILMN_179121 | -1.78 |
| XM_944786.1 | LOC650737 | ILMN_40280 | -1.78 |
| NM_001002246.1 | ANAPC11 | ILMN_5565 | -1.78 |
| NM_003104.3 | SORD | ILMN_162054 | -1.79 |
| NM_003642.2 | HAT1 | ILMN_24074 | -1.79 |
| NM_005032.3 | PLS3 | ILMN_1428 | -1.79 |
| NM_030752.2 | TCP1 | ILMN_418 | -1.79 |
| NM_001044387.1 | ZNF557 | ILMN_180266 | -1.8 |
| NM_001034996.1 | RPL14 | ILMN_2719 | -1.8 |
| NM_153188.2 | TNPO1 | ILMN_29083 | -1.8 |
| XM_943005.1 | LOC642236 | ILMN_31082 | -1.81 |
| NM_005589.2 | ALDH6A1 | ILMN_24260 | -1.81 |
| NM_016625.2 | RSRC1 | ILMN_14978 | -1.81 |
| NM_022745.3 | ATPAF1 | ILMN_175478 | -1.81 |
| NM_000984.5 | RPL23A | ILMN_9569 | -1.81 |
| NM_014183.2 | DYNLRB1 | ILMN_6713 | -1.81 |
| NM_014933.2 | SEC31A | ILMN_23819 | -1.82 |
| NM_207376.1 | LOC387882 | ILMN_23241 | -1.82 |
| NM_182533.1 | C1orf86 | ILMN_2880 | -1.82 |
| XM_934920.2 | LOC645317 | ILMN_36956 | -1.82 |
| NM_001040138.1 | CKLF | ILMN_162781 | -1.82 |
| NM_000288.1 | PEX7 | ILMN_25066 | -1.83 |
| XM_945544.1 | UBE2Z | ILMN_137054 | -1.83 |
| NM_004354.1 | CCNG2 | ILMN_10201 | -1.83 |
| NM_001121.2 | ADD3 | ILMN_4026 | -1.83 |
| NM_198156.1 | VHL | ILMN_21388 | -1.83 |
| NM_198486.2 | RPL7L1 | ILMN_9155 | -1.83 |
| NM_006601.4 | PTGES3 | ILMN_3176 | -1.83 |
| NM_031263.1 | HNRPK | ILMN_16515 | -1.84 |
| NM_006556.3 | PMVK | ILMN_165582 | -1.84 |
| NM_018137.1 | PRMT6 | ILMN_29888 | -1.84 |
| XM_941155.2 | LOC651894 | ILMN_33374 | -1.84 |
| XM_941195.2 | LOC388621 | ILMN_42661 | -1.84 |
| NM_020368.1 | UTP3 | ILMN_22785 | -1.85 |
| XR_018327.1 | LOC648343 | ILMN_163789 | -1.85 |
| NM_004866.4 | SCAMP1 | ILMN_169565 | -1.85 |
| NM_018204.2 | CKAP2 | ILMN_168115 | -1.85 |
| NM_001031720.2 | GSTCD | ILMN_18274 | -1.86 |
| NM_004435.2 | ENDOG | ILMN_26482 | -1.86 |
| NM_001039802.1 | CDC42 | ILMN_38161 | -1.86 |
| NM_001017392.2 | SFRS14 | ILMN_17110 | -1.87 |
| NM_001914.2 | CYB5A | ILMN_25182 | -1.87 |
| XM_937928.1 | LOC347376 | ILMN_31523 | -1.87 |
| NM_145080.3 | NSMCE1 | ILMN_27090 | -1.88 |
| NM_003689.2 | AKR7A2 | ILMN_182370 | -1.88 |
| NM_006963.3 | ZNF22 | ILMN_165495 | -1.88 |
| NM_032439.1 | PHYHIPL | ILMN_22045 | -1.88 |
| NM_001037442.1 | RUFY3 | ILMN_28746 | -1.89 |
| NM_001007793.1 | BUB3 | ILMN_5688 | -1.89 |
| NM_032356.3 | LSMD1 | ILMN_25444 | -1.89 |
| NM_025263.2 | PRR3 | ILMN_21022 | -1.9 |
| NM_014367.3 | C3orf28 | ILMN_24382 | -1.9 |
| NM_020532.4 | RTN4 | ILMN_164893 | -1.9 |
| NM_002014.2 | FKBP4 | ILMN_9429 | -1.9 |
| NM_016185.2 | HN1 | ILMN_3023 | -1.9 |
| XM_944489.1 | LOC651064 | ILMN_40866 | -1.9 |
| NM_031423.3 | NUF2 | ILMN_16808 | -1.91 |
| NM_130783.3 | TSPAN18 | ILMN_14181 | -1.91 |
| NM_080821.2 | C20orf108 | ILMN_25852 | -1.91 |
| NM_004064.2 | CDKN1B | ILMN_175665 | -1.91 |
| NM_153026.1 | PRICKLE1 | ILMN_15149 | -1.91 |
| NM_012484.1 | HMMR | ILMN_17450 | -1.91 |
| NM_000982.3 | RPL21 | ILMN_10150 | -1.91 |
| NM_001500.2 | GMDS | ILMN_16535 | -1.92 |
| NM_002613.3 | PDPK1 | ILMN_27765 | -1.92 |
| XM_942442.1 | LOC654121 | ILMN_35777 | -1.92 |
| NM_015971.2 | MRPS7 | ILMN_7005 | -1.92 |
| NM_181042.2 | PBRM1 | ILMN_16253 | -1.93 |
| NM_004615.2 | TSPAN7 | ILMN_20684 | -1.93 |
| XM_935589.1 | LOC641849 | ILMN_45563 | -1.93 |
| NM_004616.2 | TSPAN8 | ILMN_578 | -1.93 |
| NM_006392.2 | NOL5A | ILMN_13841 | -1.93 |
| NM_024056.2 | TMEM106C | ILMN_7003 | -1.94 |
| NM_005517.2 | HMGN2 | ILMN_12363 | -1.94 |
| XM_938755.2 | LOC653773 | ILMN_44662 | -1.94 |
| NM_006554.3 | MTX2 | ILMN_17112 | -1.95 |
| NM_016098.1 | BRP44L | ILMN_4349 | -1.96 |
| NM_015017.3 | USP33 | ILMN_176756 | -1.96 |
| NM_024920.3 | DNAJB14 | ILMN_12080 | -1.96 |
| NM_002338.2 | LSAMP | ILMN_861 | -1.96 |
| NM_001031713.2 | CCDC90A | ILMN_9159 | -1.96 |
| NM_024333.1 | FSD1 | ILMN_13664 | -1.97 |
| XM_001133089.1 | LOC731640 | ILMN_161930 | -1.97 |
| NM_015480.1 | PVRL3 | ILMN_2284 | -1.98 |
| NM_005441.2 | CHAF1B | ILMN_165317 | -1.98 |
| NM_015386.2 | COG4 | ILMN_28901 | -1.98 |
| XM_937113.2 | LOC647436 | ILMN_44829 | -1.98 |
| NM_005345.4 | HSPA1A | ILMN_6623 | -1.99 |
| NR_001568.1 | BCYRN1 | ILMN_21987 | -1.99 |
| NM_001018109.1 | PIR | ILMN_13999 | -1.99 |
| NM_052857.2 | CCDC16 | ILMN_23839 | -2 |
| NM_001042370.1 | TROVE2 | ILMN_173505 | -2 |
| NM_006054.2 | RTN3 | ILMN_20331 | -2 |
| NM_004627.2 | WRB | ILMN_12263 | -2 |
| NM_134265.2 | WSB1 | ILMN_5396 | -2 |
| NM_001018109.1 | PIR | ILMN_13999 | -2 |
| NM_003924.2 | PHOX2B | ILMN_172224 | -2 |
| NM_203284.1 | RBPJ | ILMN_170184 | -2.01 |
| NM_016448.1 | DTL | ILMN_29702 | -2.01 |
| NM_000856.3 | GUCY1A3 | ILMN_11680 | -2.01 |
| NM_032747.2 | USMG5 | ILMN_10409 | -2.02 |
| NM_002882.2 | RANBP1 | ILMN_25664 | -2.02 |
| NM_007236.3 | CHP | ILMN_23083 | -2.03 |
| NM_020150.3 | SAR1A | ILMN_17495 | -2.03 |
| NM_000814.4 | GABRB3 | ILMN_19294 | -2.03 |
| NM_006870.3 | DSTN | ILMN_13158 | -2.03 |
| NM_012482.3 | ZNF281 | ILMN_18970 | -2.03 |
| NM_145117.3 | NAV2 | ILMN_8536 | -2.04 |
| NM_017812.2 | CHCHD3 | ILMN_23539 | -2.04 |
| NM_182649.1 | PCNA | ILMN_6858 | -2.04 |
| XM_001133534.1 | ATP1B3 | ILMN_163124 | -2.04 |
| NR_002187.2 | LOC286016 | ILMN_16558 | -2.04 |
| NM_001003.2 | RPLP1 | ILMN_23181 | -2.04 |
| NM_184234.1 | RBM39 | ILMN_20330 | -2.05 |
| NM_001540.2 | HSPB1 | ILMN_28967 | -2.05 |
| NM_022135.2 | POPDC2 | ILMN_17743 | -2.06 |
| XM_496446.3 | LOC440737 | ILMN_39347 | -2.06 |
| NM_139207.1 | NAP1L1 | ILMN_5405 | -2.07 |
| XR_016048.1 | MGC40489 | ILMN_171153 | -2.07 |
| NM_005749.2 | TOB1 | ILMN_13735 | -2.07 |
| NM_001040139.1 | CKLF | ILMN_162861 | -2.07 |
| NM_003366.2 | UQCRC2 | ILMN_10929 | -2.08 |
| NM_002759.1 | EIF2AK2 | ILMN_168435 | -2.08 |
| NM_006014.3 | LAGE3 | ILMN_1071 | -2.08 |
| NM_005188.2 | CBL | ILMN_172998 | -2.09 |
| NM_018193.2 | FANCI | ILMN_15143 | -2.09 |
| NM_015895.3 | GMNN | ILMN_20255 | -2.09 |
| NM_016039.1 | C14orf166 | ILMN_14906 | -2.09 |
| NM_022173.1 | TIA1 | ILMN_29910 | -2.1 |
| NM_152773.2 | TCTEX1D2 | ILMN_19950 | -2.1 |
| NM_033258.1 | GNG8 | ILMN_25463 | -2.1 |
| NM_030752.2 | TCP1 | ILMN_418 | -2.11 |
| XM_944991.1 | C14orf82 | ILMN_36432 | -2.12 |
| XM_001134259.1 | LOC732165 | ILMN_170212 | -2.12 |
| NM_016047.3 | SF3B14 | ILMN_12279 | -2.12 |
| NM_006265.1 | RAD21 | ILMN_171453 | -2.12 |
| XM_001131304.1 | LOC728635 | ILMN_168315 | -2.13 |
| NM_005824.1 | LRRC17 | ILMN_162504 | -2.13 |
| NM_004470.2 | FKBP2 | ILMN_17464 | -2.13 |
| NM_015449.2 | C1orf43 | ILMN_933 | -2.13 |
| NM_004935.2 | CDK5 | ILMN_9211 | -2.13 |
| NM_016395.2 | PTPLAD1 | ILMN_9196 | -2.13 |
| NM_001100164.1 | PHACTR2 | ILMN_307784 | -2.14 |
| NM_005708.2 | GPC6 | ILMN_16550 | -2.14 |
| NM_001040034.1 | CD63 | ILMN_167218 | -2.14 |
| XM_292963.6 | LOC643997 | ILMN_39721 | -2.14 |
| NM_198954.1 | NUDT1 | ILMN_2361 | -2.15 |
| NM_002897.3 | RBMS1 | ILMN_18726 | -2.15 |
| NM_002004.2 | FDPS | ILMN_18516 | -2.15 |
| NM_133505.2 | DCN | ILMN_29913 | -2.15 |
| NM_024122.2 | APOO | ILMN_11248 | -2.16 |
| NM_001003793.1 | RBMS3 | ILMN_16411 | -2.16 |
| NM_002951.2 | RPN2 | ILMN_30123 | -2.16 |
| NM_003403.3 | YY1 | ILMN_4019 | -2.16 |
| NM_006366.2 | CAP2 | ILMN_27367 | -2.17 |
| NM_002225.2 | IVD | ILMN_13293 | -2.17 |
| NM_022549.2 | FEZ1 | ILMN_419 | -2.17 |
| NM_001495.4 | GFRA2 | ILMN_24176 | -2.18 |
| NM_022743.1 | SMYD3 | ILMN_29453 | -2.18 |
| NM_024292.2 | UBL5 | ILMN_14261 | -2.18 |
| NM_145800.2 | 6-Sep | ILMN_29094 | -2.18 |
| NM_133459.1 | CCBE1 | ILMN_6075 | -2.18 |
| NM_005842.2 | SPRY2 | ILMN_19344 | -2.18 |
| NM_014033.3 | METTL7A | ILMN_40171 | -2.19 |
| NM_001979.4 | EPHX2 | ILMN_179814 | -2.19 |
| NM_012319.2 | SLC39A6 | ILMN_170037 | -2.19 |
| XM_942669.1 | LOC654194 | ILMN_31988 | -2.19 |
| NM_007280.1 | OIP5 | ILMN_18200 | -2.2 |
| NM_017526.2 | LEPROT | ILMN_27032 | -2.2 |
| NM_001008800.1 | CCT3 | ILMN_24878 | -2.2 |
| XM_936240.1 | LOC653884 | ILMN_34094 | -2.2 |
| XM_930884.1 | LOC653080 | ILMN_32261 | -2.21 |
| XM_940610.1 | LOC651453 | ILMN_32585 | -2.21 |
| NM_020189.4 | ENY2 | ILMN_21796 | -2.21 |
| NM_004401.2 | DFFA | ILMN_6993 | -2.22 |
| NM_014620.4 | HOXC4 | ILMN_16005 | -2.22 |
| NM_001124.1 | ADM | ILMN_29514 | -2.22 |
| NM_003864.3 | SAP30 | ILMN_31250 | -2.22 |
| CD673408 |  | ILMN_114494 | -2.23 |
| NM_000814.4 | GABRB3 | ILMN_19294 | -2.23 |
| NM_145697.1 | CDCA1 | ILMN_17725 | -2.25 |
| NM_006527.2 | SLBP | ILMN_3687 | -2.25 |
| NM_001024921.2 | RPL9 | ILMN_8640 | -2.26 |
| NM_024766.2 | C2orf34 | ILMN_14025 | -2.27 |
| NM_016937.2 | POLA1 | ILMN_181974 | -2.28 |
| XM_001126212.1 | C7orf28B | ILMN_172434 | -2.28 |
| NM_004316.2 | ASCL1 | ILMN_23892 | -2.28 |
| XM_935588.1 | LOC641848 | ILMN_45490 | -2.28 |
| NM_001080477.1 | ODZ3 | ILMN_179907 | -2.29 |
| NM_020236.2 | MRPL1 | ILMN_22997 | -2.29 |
| NM_013354.5 | CNOT7 | ILMN_7214 | -2.29 |
| NM_032340.2 | C6orf125 | ILMN_21424 | -2.29 |
| NM_005318.2 | H1F0 | ILMN_139403 | -2.29 |
| NM_025136.1 | OPA3 | ILMN_11296 | -2.3 |
| NM_030940.3 | ISCA1 | ILMN_171173 | -2.3 |
| NM_005994.3 | TBX2 | ILMN_18788 | -2.3 |
| NM_001640.3 | APEH | ILMN_27694 | -2.3 |
| NM_030881.2 | DDX17 | ILMN_28024 | -2.3 |
| NM_005654.4 | NR2F1 | ILMN_177945 | -2.31 |
| NM_004987.3 | LIMS1 | ILMN_11207 | -2.32 |
| NM_033402.3 | LRRCC1 | ILMN_15234 | -2.32 |
| XM_497072.2 | LOC389787 | ILMN_45784 | -2.32 |
| NM_004774.2 | PPARBP | ILMN_14182 | -2.33 |
| XM_933893.1 | LOC389672 | ILMN_35589 | -2.34 |
| XM_937706.1 | LOC648638 | ILMN_41215 | -2.34 |
| NM_183422.1 | TSC22D1 | ILMN_166165 | -2.35 |
| NM_025129.3 | FUZ | ILMN_24173 | -2.36 |
| NM_001003897.1 | MANBAL | ILMN_11747 | -2.36 |
| NM_003583.3 | DYRK2 | ILMN_3688 | -2.36 |
| NM_153682.2 | PIGP | ILMN_18625 | -2.36 |
| NM_182547.2 | TMED4 | ILMN_30359 | -2.36 |
| NM_021170.2 | HES4 | ILMN_18566 | -2.37 |
| NM_002923.1 | RGS2 | ILMN_26119 | -2.37 |
| NM_007358.2 | MTF2 | ILMN_24749 | -2.37 |
| NM_006026.2 | H1FX | ILMN_26614 | -2.37 |
| XM_945045.1 | LOC649679 | ILMN_34833 | -2.38 |
| NM_138809.3 | CMBL | ILMN_1485 | -2.38 |
| NM_001634.4 | AMD1 | ILMN_21529 | -2.38 |
| NM_181054.1 | HIF1A | ILMN_9514 | -2.38 |
| NM_032361.1 | THOC3 | ILMN_17969 | -2.38 |
| NM_001040285.1 | PAPD5 | ILMN_167231 | -2.4 |
| NR_002201.1 | FTHL3 | ILMN_27691 | -2.4 |
| NM_005443.4 | PAPSS1 | ILMN_171260 | -2.4 |
| NM_002013.2 | FKBP3 | ILMN_7680 | -2.4 |
| XR_019449.1 | LOC644422 | ILMN_166674 | -2.4 |
| NM_004891.2 | MRPL33 | ILMN_12897 | -2.41 |
| NM_016048.1 | ISOC1 | ILMN_15311 | -2.41 |
| NM_205843.1 | NFIC | ILMN_22629 | -2.42 |
| NM_006117.2 | PECI | ILMN_7427 | -2.42 |
| NM_000978.3 | RPL23 | ILMN_8866 | -2.42 |
| NM_002247.2 | KCNMA1 | ILMN_24236 | -2.43 |
| NM_004343.2 | CALR | ILMN_18909 | -2.43 |
| NM_001040142.1 | SCN2A | ILMN_167124 | -2.43 |
| NM_001866.2 | COX7B | ILMN_19298 | -2.43 |
| NM_015609.2 | C1orf144 | ILMN_5836 | -2.44 |
| NM_005713.1 | COL4A3BP | ILMN_10635 | -2.44 |
| NM_139283.1 | PPTC7 | ILMN_11800 | -2.45 |
| NM_152362.1 | TNFAIP8L1 | ILMN_3344 | -2.45 |
| NM_024540.2 | MRPL24 | ILMN_29128 | -2.45 |
| NM_001013699.1 | LOC440093 | ILMN_19743 | -2.46 |
| NM_001097615.1 | POLR2J3 | ILMN_308895 | -2.47 |
| NM_001034996.1 | RPL14 | ILMN_2719 | -2.48 |
| NM_006191.2 | PA2G4 | ILMN_28541 | -2.48 |
| NM_021825.3 | CCDC90B | ILMN_6208 | -2.49 |
| NM_012433.2 | SF3B1 | ILMN_168075 | -2.49 |
| NM_017958.1 | PLEKHB2 | ILMN_29704 | -2.51 |
| NM_001786.2 | CDC2 | ILMN_24793 | -2.51 |
| NM_012117.1 | CBX5 | ILMN_25072 | -2.52 |
| XM_926370.1 | LOC642989 | ILMN_33765 | -2.52 |
| NR_002308.1 | LOC442454 | ILMN_309609 | -2.52 |
| NM_199287.2 | CCDC137 | ILMN_309720 | -2.54 |
| NM_017819.2 | RG9MTD1 | ILMN_26970 | -2.54 |
| XM_926249.2 | LOC642852 | ILMN_40586 | -2.54 |
| NM_003583.2 | DYRK2 | ILMN_3688 | -2.54 |
| XR_015809.1 | LOC728973 | ILMN_168278 | -2.55 |
| NM_001014438.1 | CARS | ILMN_172747 | -2.56 |
| NM_002870.2 | RAB13 | ILMN_26464 | -2.56 |
| NM_005192.2 | CDKN3 | ILMN_4098 | -2.56 |
| NM_001545.1 | ICT1 | ILMN_11458 | -2.56 |
| NM_003211.3 | TDG | ILMN_29212 | -2.56 |
| NM_005694.1 | COX17 | ILMN_19252 | -2.56 |
| NM_001031723.1 | DNAJB14 | ILMN_9854 | -2.57 |
| NM_001321.1 | CSRP2 | ILMN_3862 | -2.57 |
| NM_001333.2 | CTSL2 | ILMN_22377 | -2.57 |
| NM_006429.2 | CCT7 | ILMN_22959 | -2.57 |
| NM_016108.2 | AIG1 | ILMN_22004 | -2.58 |
| NM_005077.3 | TLE1 | ILMN_10669 | -2.58 |
| NM_006860.2 | RABL4 | ILMN_4559 | -2.58 |
| NM_014142.2 | NUDT5 | ILMN_1656 | -2.58 |
| NM_000971.3 | RPL7 | ILMN_26351 | -2.6 |
| XM_934410.1 | LOC643995 | ILMN_31166 | -2.61 |
| NM_006158.2 | NEFL | ILMN_22054 | -2.61 |
| NM_021156.2 | TXNDC13 | ILMN_23065 | -2.62 |
| NM_005056.1 | JARID1A | ILMN_12150 | -2.62 |
| NR_002200.1 | FTHL2 | ILMN_15867 | -2.63 |
| NR_002204.1 | FTHL11 | ILMN_16343 | -2.63 |
| NR_003040.1 | LOC649946 | ILMN_169528 | -2.63 |
| NM_001010982.2 | AFMID | ILMN_5520 | -2.64 |
| NM_020548.4 | DBI | ILMN_30320 | -2.65 |
| NM_031453.2 | FAM107B | ILMN_2236 | -2.67 |
| NM_014170.2 | GTPBP8 | ILMN_27163 | -2.68 |
| XR_015514.1 | LOC730746 | ILMN_163533 | -2.68 |
| NM_138418.2 | C16orf14 | ILMN_9509 | -2.72 |
| NM_001009608.1 | C20orf94 | ILMN_24801 | -2.72 |
| NM_001007214.1 | CACYBP | ILMN_16795 | -2.73 |
| NM_031314.1 | HNRPC | ILMN_24356 | -2.73 |
| XM_941684.2 | LOC220433 | ILMN_46655 | -2.74 |
| NR_001283.1 | TOP1P2 | ILMN_4755 | -2.75 |
| XM_930029.1 | LOC647037 | ILMN_32596 | -2.75 |
| NM_012322.1 | LSM5 | ILMN_17896 | -2.75 |
| NM_005909.3 | MAP1B | ILMN_28251 | -2.76 |
| NM_015480.1 | PVRL3 | ILMN_2284 | -2.77 |
| NM_174909.3 | TMEM167 | ILMN_6582 | -2.77 |
| XM_942780.2 | SYNPO2 | ILMN_45907 | -2.78 |
| NM_005754.2 | G3BP1 | ILMN_1152 | -2.78 |
| NM_018246.2 | CCDC25 | ILMN_5229 | -2.8 |
| NM_014170.2 | GTPBP8 | ILMN_27163 | -2.81 |
| NM_006324.2 | CFDP1 | ILMN_23508 | -2.81 |
| NR_002205.1 | FTHL12 | ILMN_16447 | -2.81 |
| XM_940333.2 | LOC651202 | ILMN_37363 | -2.81 |
| NM_019067.4 | GNL3L | ILMN_181682 | -2.83 |
| NM_018639.3 | WSB2 | ILMN_162438 | -2.85 |
| NM_016618.1 | KRCC1 | ILMN_25337 | -2.85 |
| NM_005388.3 | PDCL | ILMN_34020 | -2.86 |
| NM_014007.2 | ZBTB43 | ILMN_17837 | -2.86 |
| NM_182810.1 | ATF4 | ILMN_23435 | -2.87 |
| NM_014060.1 | MCTS1 | ILMN_13725 | -2.88 |
| NM_000599.2 | IGFBP5 | ILMN_168089 | -2.88 |
| NM_020640.2 | DCUN1D1 | ILMN_410 | -2.89 |
| NR_001449.1 | TRK1 | ILMN_6493 | -2.9 |
| NM_080597.2 | OSBPL1A | ILMN_10951 | -2.9 |
| NM_001039703.1 | NBPF10 | ILMN_45673 | -2.9 |
| NM_020749.3 | MTUS1 | ILMN_4658 | -2.91 |
| XM_930694.1 | LOC642477 | ILMN_36253 | -2.91 |
| NM_018334.3 | LRRN3 | ILMN_174401 | -2.91 |
| NM_017895.6 | DDX27 | ILMN_20732 | -2.92 |
| NM_014169.2 | CHMP4A | ILMN_19959 | -2.93 |
| NM_020040.3 | TUBB4Q | ILMN_177504 | -2.93 |
| XR_018325.1 | LOC644131 | ILMN_166020 | -2.93 |
| XM_929862.1 | LOC646900 | ILMN_44661 | -2.94 |
| NM_014865.2 | NCAPD2 | ILMN_26621 | -2.94 |
| NM_003107.2 | SOX4 | ILMN_17456 | -2.94 |
| NM_024011.2 | CDC2L2 | ILMN_20434 | -2.98 |
| NM_152789.2 | FAM133B | ILMN_1247 | -2.99 |
| NR_002203.1 | FTHL8 | ILMN_16227 | -2.99 |
| NM_005723.2 | TSPAN5 | ILMN_8032 | -3 |
| NM_018304.2 | PRR11 | ILMN_32619 | -3.01 |
| NR_002204.1 | FTHL11 | ILMN_16343 | -3.01 |
| NM_015934.3 | NOP5/NOP58 | ILMN_4530 | -3.02 |
| XM_938297.1 | LOC402644 | ILMN_30715 | -3.02 |
| NM_006914.3 | RORB | ILMN_7297 | -3.03 |
| NM_006630.1 | ZNF234 | ILMN_29233 | -3.04 |
| NM_178439.3 | GMCL1 | ILMN_3285 | -3.04 |
| NR_003144.1 | LOC723972 | ILMN_180363 | -3.06 |
| XM_938089.2 | LOC643007 | ILMN_31054 | -3.08 |
| NM_019116.2 | UBFD1 | ILMN_179383 | -3.1 |
| NM_012342.2 | BAMBI | ILMN_8469 | -3.11 |
| XM_929738.1 | LOC646786 | ILMN_38919 | -3.13 |
| NM_001008735.1 | HMG1L1 | ILMN_22757 | -3.14 |
| XM_944321.1 | LOC402560 | ILMN_42108 | -3.15 |
| NM_080386.1 | TUBA3D | ILMN_30319 | -3.17 |
| NM_001025248.1 | DUT | ILMN_163345 | -3.17 |
| NM_000587.2 | C7 | ILMN_15063 | -3.22 |
| NM_005238.2 | ETS1 | ILMN_173009 | -3.23 |
| XM_001132569.1 | LOC730130 | ILMN_162537 | -3.23 |
| NM_001005849.1 | SUMO2 | ILMN_16713 | -3.23 |
| NM_018983.3 | NOLA1 | ILMN_14204 | -3.24 |
| XM_936215.1 | LOC653874 | ILMN_35327 | -3.24 |
| NM_001037675.1 | NBPF20 | ILMN_26956 | -3.24 |
| NM_002086.3 | GRB2 | ILMN_173749 | -3.25 |
| NM_017802.2 | HEATR2 | ILMN_1114 | -3.26 |
| XM_938599.2 | LOC441377 | ILMN_31681 | -3.27 |
| XM_934985.1 | LOC400879 | ILMN_31001 | -3.33 |
| NR_002182.1 | NACAP1 | ILMN_14666 | -3.34 |
| NM_016587.2 | CBX3 | ILMN_11642 | -3.37 |
| NM_024051.2 | C7orf24 | ILMN_2391 | -3.38 |
| XM_374020.4 | LOC375295 | ILMN_45377 | -3.38 |
| NM_002157.1 | HSPE1 | ILMN_2612 | -3.39 |
| NM_004901.2 | ENTPD4 | ILMN_19012 | -3.4 |
| NM_203390.2 | RBM12B | ILMN_174962 | -3.41 |
| NM_000598.4 | IGFBP3 | ILMN_28010 | -3.43 |
| NR_002315.1 | LOC440926 | ILMN_19720 | -3.44 |
| NM_003368.4 | USP1 | ILMN_5285 | -3.49 |
| NM_004544.2 | NDUFA10 | ILMN_7463 | -3.49 |
| NR_002205.1 | FTHL12 | ILMN_16447 | -3.49 |
| NM_016374.5 | ARID4B | ILMN_162934 | -3.52 |
| NM_003344.2 | UBE2H | ILMN_163352 | -3.55 |
| NM_014170.2 | GTPBP8 | ILMN_27163 | -3.58 |
| NM_153333.2 | TCEAL8 | ILMN_12551 | -3.6 |
| XM_498571.2 | LOC440160 | ILMN_33035 | -3.61 |
| NM_178439.3 | GMCL1 | ILMN_3285 | -3.63 |
| NM_001008735.1 | HMG1L1 | ILMN_22757 | -3.63 |
| NM_002167.2 | ID3 | ILMN_6829 | -3.68 |
| XM_933956.1 | LOC644162 | ILMN_43225 | -3.72 |
| XM_936103.1 | LOC642033 | ILMN_33652 | -3.73 |
| XM_930284.1 | LOC441763 | ILMN_36192 | -3.74 |
| NM_006004.1 | UQCRH | ILMN_138507 | -3.74 |
| XM_935818.1 | FLJ20397 | ILMN_137080 | -3.76 |
| NM_017489.1 | TERF1 | ILMN_164297 | -3.77 |
| XM_001129423.1 | LOC729137 | ILMN_166772 | -3.8 |
| NM_002568.3 | PABPC1 | ILMN_173094 | -3.81 |
| NM_020705.1 | TBC1D24 | ILMN_34755 | -3.86 |
| NR_003287.1 | LOC100008589 | ILMN_177351 | -3.87 |
| NM_024570.1 | RNASEH2B | ILMN_20578 | -3.88 |
| XM_938779.1 | LOC653972 | ILMN_31111 | -3.91 |
| NM_005905.3 | SMAD9 | ILMN_28187 | -3.93 |
| NM_016374.5 | ARID4B | ILMN_162934 | -3.95 |
| NM_004456.3 | EZH2 | ILMN_25740 | -3.95 |
| NM_001034841.2 | LOC162073 | ILMN_3559 | -4.06 |
| NM_003358.1 | UGCG | ILMN_26228 | -4.07 |
| NM_174942.1 | GAS2L3 | ILMN_5609 | -4.07 |
| NM_001438.2 | ESRRG | ILMN_29221 | -4.07 |
| NM_181332.1 | NLGN4X | ILMN_27075 | -4.09 |
| NM_020242.1 | KIF15 | ILMN_6188 | -4.09 |
| XM_928075.2 | LOC643287 | ILMN_37869 | -4.09 |
| NM_006630.1 | ZNF234 | ILMN_29233 | -4.11 |
| NM_003542.3 | HIST1H4C | ILMN_30043 | -4.24 |
| NM_022652.2 | DUSP6 | ILMN_5926 | -4.35 |
| NM_078629.1 | MSL3L1 | ILMN_29354 | -4.37 |
| NM_020449.2 | THOC2 | ILMN_162047 | -4.38 |
| NR_003041.1 | SNORD13 | ILMN_168446 | -4.44 |
| NM_032334.1 | C8orf53 | ILMN_24637 | -4.49 |
| NM_006182.2 | DDR2 | ILMN_20698 | -4.5 |
| NM_012234.4 | RYBP | ILMN_13259 | -4.58 |
| NM_002093.2 | GSK3B | ILMN_7421 | -4.68 |
| NM_004257.3 | TGFBRAP1 | ILMN_30176 | -4.71 |
| NM_000599.2 | IGFBP5 | ILMN_168089 | -4.89 |
| NM_002763.3 | PROX1 | ILMN_177185 | -4.93 |
| NM_022731.2 | NUCKS1 | ILMN_17108 | -5 |
| NM_014498.2 | GOLPH4 | ILMN_179486 | -5.18 |
| NM_006265.1 | RAD21 | ILMN_171453 | -5.27 |
| NR_001445.1 | RN7SK | ILMN_14457 | -5.29 |
| NM_006717.2 | SPIN1 | ILMN_23742 | -5.35 |
| NM_004175.3 | SNRPD3 | ILMN_163179 | -5.77 |
| NM_002166.4 | ID2 | ILMN_28481 | -6.05 |
| NM_001099285.1 | PTMA | ILMN_306831 | -6.05 |
| NM_002166.4 | ID2 | ILMN_28481 | -6.24 |
| NM_138444.3 | KCTD12 | ILMN_18501 | -6.51 |
