# Supplementary material for: Minimal Peroxide Exposure of Neuronal Cells Induces Multifaceted Adaptive Responses
Source: PLoS One. 2010 Dec 17;5(12):e14352. doi: 10.1371/journal.pone.0014352 (PMC3003681; doi:10.1371/journal.pone.0014352)
Supplement: Table S15 — Common 2 hour MeCh-regulated gene series. MeCh-regulated genes that were significantly elevated or reduced compared to the respective unstimulated control cells in both the control (untreated: MeCh-2h-Control vs. Control-Control) and CMP state SH-SY5Y cells (MeCh-2h-CMP vs. Control-CMP). The series number refers to the simplistic relationships between the degree of regulation of the respective genes and the cellular state (untreated or CMP). Series 1 (both upregulated) - MeCh-2h-CMP vs. Control-CMP > MeCh-2h-Control vs. Control-Control; Series 2 (both upregulated) - MeCh-2h-Control vs. Control-Control > MeCh-2h-CMP vs. Control-CMP; Series 3 (both downregulated) - MeCh-2h-Control vs. Control-Control > MeCh-2h-CMP vs. Control-CMP; Series 4 (both downregulated) - MeCh-2h-CMP vs. Control-CMP > MeCh-2h-Control vs. Control-Control; Series 5 (downregulated in control, upregulated in CMP); Series 6 (upregulated in control, down regulated in CMP). (0.42 MB DOC) [file pone.0014352.s022.doc]

**Table S15. Common 2 hour MeCh-regulated gene series.** MeCh-regulated genes that were significantly elevated or reduced compared to the respective unstimulated control cells in both the control (untreated: *MeCh-2h-Control vs. Control-Control*) and CMP state SH-SY5Y cells (*MeCh-2h-CMP vs. Control-CMP*). The series number refers to the simplistic relationships between the degree of regulation of the respective genes and the cellular state (untreated or CMP). Series 1 (both upregulated) - *MeCh-2h-CMP vs. Control-CMP* > *MeCh-2h-Control vs. Control-Control*; Series 2 (both upregulated) - *MeCh-2h-Control vs. Control-Control* > *MeCh-2h-CMP vs. Control-CMP*; Series 3 (both downregulated) - *MeCh-2h-Control vs. Control-Control* > *MeCh-2h-CMP vs. Control-CMP*; Series 4 (both downregulated) - *MeCh-2h-CMP vs. Control-CMP* > *MeCh-2h-Control vs. Control-Control*; Series 5 (downregulated in control, upregulated in CMP); Series 6 (upregulated in control, down regulated in CMP).

| **SYMBOL** | **MeCh-2h-CMP vs. Control-CMP** | **MeCh-2h-Control vs. Control-Control** | **Series #** |
| --- | --- | --- | --- |
| IL8 | 6.845278435 | 6.520041167 | **1** |
| H3F3B | 5.70380288 | 5.09983169 | **1** |
| KIAA0644 | 5.345106781 | 3.594671002 | **1** |
| MGEA5 | 4.872843542 | 2.92057767 | **1** |
| LANCL2 | 4.321443637 | 2.904097932 | **1** |
| INTS1 | 4.106563215 | 3.416018035 | **1** |
| KLF11 | 3.944674267 | 2.312236024 | **1** |
| LRP5L | 3.782011179 | 2.419792806 | **1** |
| SLC11A2 | 3.744786328 | 1.902506021 | **1** |
| CSTF3 | 3.674630221 | 3.225241062 | **1** |
| RAD23B | 3.663814151 | 3.017873643 | **1** |
| IL18BP | 3.627537714 | 2.698488566 | **1** |
| SUV420H1 | 3.583109945 | 3.309701583 | **1** |
| TTC32 | 3.552632382 | 2.73031545 | **1** |
| SPRY1 | 3.548668864 | 2.846397224 | **1** |
| ARRDC3 | 3.451238161 | 2.486334281 | **1** |
| TNFRSF19 | 3.42481695 | 2.366027841 | **1** |
| ADAMTS1 | 3.389545744 | 1.871647172 | **1** |
| PLXNB1 | 3.364080503 | 2.484213627 | **1** |
| RNF150 | 3.18711031 | 2.172262454 | **1** |
| ITGB5 | 3.18275435 | 2.197691849 | **1** |
| SGK | 3.144015663 | 2.996873909 | **1** |
| SFRS6 | 3.137639171 | 1.965175421 | **1** |
| PSME4 | 3.116000385 | 1.863125649 | **1** |
| USP24 | 3.085540612 | 1.605144945 | **1** |
| RIMS3 | 3.078623357 | 1.603617849 | **1** |
| CAMSAP1 | 3.063241421 | 2.234426296 | **1** |
| IRS2 | 2.970177076 | 2.51726415 | **1** |
| ACACA | 2.967524434 | 1.848459108 | **1** |
| ASCC3L1 | 2.954320945 | 2.056479227 | **1** |
| ABL1 | 2.849799008 | 1.897335988 | **1** |
| ACACA | 2.830004568 | 2.187473191 | **1** |
| LOC285074 | 2.810833746 | 2.468944558 | **1** |
| FAT | 2.749990347 | 1.920163708 | **1** |
| MEF2D | 2.739535657 | 1.874379114 | **1** |
| PHF17 | 2.641146498 | 1.774252195 | **1** |
| GATS | 2.639538181 | 1.785912167 | **1** |
| LOC285176 | 2.635429951 | 1.86545602 | **1** |
| DUSP3 | 2.612810812 | 1.814036402 | **1** |
| SPG7 | 2.603728098 | 2.029275468 | **1** |
| ABCA3 | 2.595562315 | 1.67531128 | **1** |
| FEM1A | 2.579660084 | 1.882309414 | **1** |
| SNAPC4 | 2.542364803 | 2.12910558 | **1** |
| C9orf156 | 2.52873207 | 1.738349997 | **1** |
| SYT11 | 2.520494625 | 1.788804725 | **1** |
| HCFC1 | 2.503977052 | 2.386591943 | **1** |
| PTP4A2 | 2.423198767 | 1.655361092 | **1** |
| CSF2RA | 2.403621286 | 1.503857382 | **1** |
| SFRS5 | 2.402414707 | 2.2475768 | **1** |
| CLEC16A | 2.390151197 | 2.143034352 | **1** |
| CX3CL1 | 2.379550874 | 1.626230654 | **1** |
| MSI2 | 2.364318572 | 1.837918674 | **1** |
| ICA1 | 2.343598087 | 2.141658572 | **1** |
| GAS1 | 2.314745411 | 1.889014974 | **1** |
| POLR3H | 2.311332638 | 1.710330405 | **1** |
| KCNT1 | 2.310079479 | 2.034816409 | **1** |
| ADAR | 2.291834517 | 1.61426476 | **1** |
| CLCN6 | 2.281724939 | 1.951391042 | **1** |
| RBM33 | 2.269618193 | 1.690529389 | **1** |
| ING3 | 2.236937945 | 1.619316768 | **1** |
| RAPGEF1 | 2.223731894 | 1.63977283 | **1** |
| ZNF16 | 2.201443878 | 1.543295094 | **1** |
| CPSF1 | 2.12988565 | 1.683740798 | **1** |
| PPHLN1 | 2.126566739 | 1.886437906 | **1** |
| PFKFB3 | 2.116365373 | 1.727274266 | **1** |
| FLJ38717 | 2.008038819 | 1.732145442 | **1** |
| HNRPK | 1.962864789 | 1.681890415 | **1** |
| REPIN1 | 1.958631168 | 1.942116828 | **1** |
| FLJ35801 | 1.951789496 | 1.561297688 | **1** |
| PRDM8 | 1.944328773 | 1.755574759 | **1** |
| TOP3A | 1.936118133 | 1.751297394 | **1** |
| P704P | 1.935190828 | 1.734449211 | **1** |
| UHRF1 | 1.929611912 | 1.684217719 | **1** |
| C14orf173 | 1.91659738 | 1.617357054 | **1** |
| RAI1 | 1.84717064 | 1.610070842 | **1** |
| TRRAP | 1.841480303 | 1.665641756 | **1** |
| ULK1 | 1.80511803 | 1.772824544 | **1** |
| SUV420H1 | 1.800862641 | 1.771369527 | **1** |
| KIAA0363 | 1.786401054 | 1.739586243 | **1** |
| SON | 1.780730874 | 1.563521299 | **1** |
| SIAH1 | 1.765156753 | 1.700718542 | **1** |
| ZGPAT | 1.754236962 | 1.617171375 | **1** |
| AKAP13 | 1.743465134 | 1.513324178 | **1** |
| ULK1 | 1.739497186 | 1.602860979 | **1** |
| MAPRE2 | 1.716396812 | 1.549679325 | **1** |
| GCH1 | 1.689187789 | 1.652834151 | **1** |
| SDC1 | 1.679412721 | 1.597700044 | **1** |
| EGR1 | 14.65846509 | 17.38659085 | **2** |
| NFKBIZ | 4.841539088 | 6.827315785 | **2** |
| LOC653994 | 5.063268451 | 6.305238009 | **2** |
| KLF6 | 4.225117642 | 5.541527822 | **2** |
| KLF6 | 3.738451772 | 5.497957169 | **2** |
| DUSP1 | 3.510941995 | 4.153932569 | **2** |
| GADD45A | 2.371036475 | 3.929903028 | **2** |
| P2RY11 | 3.353753306 | 3.838124553 | **2** |
| PPP1R15A | 2.748362589 | 3.737509247 | **2** |
| MNT | 2.927211989 | 3.718531911 | **2** |
| CALD1 | 3.269172649 | 3.717370033 | **2** |
| LOC387763 | 2.957103898 | 3.714609178 | **2** |
| MCM7 | 2.266010951 | 3.643731114 | **2** |
| LOC338758 | 2.394621659 | 3.332528416 | **2** |
| ZYX | 1.902069167 | 3.29343872 | **2** |
| ZNF789 | 2.651224633 | 3.265707952 | **2** |
| LOC401357 | 2.091575223 | 3.196904172 | **2** |
| ZFHX3 | 1.733019055 | 3.130133676 | **2** |
| NGFRAP1 | 2.620758078 | 3.077815545 | **2** |
| DBH | 1.786403563 | 3.000565563 | **2** |
| LOC727935 | 2.281850187 | 2.966188798 | **2** |
| LOC653103 | 2.794144108 | 2.942910646 | **2** |
| CIRBP | 1.807376612 | 2.812479591 | **2** |
| SNORA70 | 2.276953867 | 2.745515995 | **2** |
| C14orf102 | 2.435828826 | 2.721012837 | **2** |
| PIGW | 2.615109478 | 2.686219911 | **2** |
| SYNCRIP | 2.473120318 | 2.677238836 | **2** |
| RHBDD2 | 2.27593047 | 2.576450553 | **2** |
| LAMA5 | 2.207753079 | 2.551457217 | **2** |
| PERLD1 | 2.306337248 | 2.491151539 | **2** |
| NR2F1 | 1.696104851 | 2.46806891 | **2** |
| GNG11 | 2.017856705 | 2.440796479 | **2** |
| SFRS5 | 2.314094119 | 2.416121234 | **2** |
| LOC402221 | 1.592920117 | 2.39667549 | **2** |
| SLC7A5 | 1.614745618 | 2.365869503 | **2** |
| DYNLL2 | 1.576203398 | 2.353064089 | **2** |
| SRF | 2.299691266 | 2.351934979 | **2** |
| ADAMTSL2 | 2.069558203 | 2.341108878 | **2** |
| ZC3H5 | 1.708786231 | 2.286000807 | **2** |
| SP2 | 2.030546275 | 2.233271751 | **2** |
| UBC | 1.617514079 | 2.224954063 | **2** |
| SBF1 | 2.047314181 | 2.202225667 | **2** |
| CLCN7 | 1.842500334 | 2.176102481 | **2** |
| MYLIP | 1.883659654 | 2.112296994 | **2** |
| DECR2 | 1.945328581 | 2.080677988 | **2** |
| SNORD68 | 1.848244894 | 2.080516875 | **2** |
| MYH9 | 1.861020586 | 2.079328499 | **2** |
| KLHDC4 | 1.68158997 | 2.051494999 | **2** |
| TSKU | 1.520851902 | 2.033775518 | **2** |
| INPPL1 | 1.713633438 | 2.026011008 | **2** |
| E4F1 | 1.807123451 | 1.990872356 | **2** |
| RNF165 | 1.784558049 | 1.954555909 | **2** |
| PTBP1 | 1.558702122 | 1.952823095 | **2** |
| FOXO3 | 1.860594992 | 1.941705219 | **2** |
| TACC2 | 1.507980144 | 1.824427738 | **2** |
| GMEB2 | 1.751889531 | 1.783953488 | **2** |
| REXO1 | 1.578598035 | 1.728776679 | **2** |
| ARID3B | 1.583247831 | 1.716325153 | **2** |
| ADNP2 | 1.580853346 | 1.712580618 | **2** |
| AFG3L2 | 1.579607829 | 1.627482062 | **2** |
| SPECC1L | 1.578434311 | 1.585557283 | **2** |
| LOC196752 | 1.509304658 | 1.5409643 | **2** |
| HSPA1B | -7.397511554 | -7.325561498 | **3** |
| ARID4B | -4.659354819 | -3.070512512 | **3** |
| MSL3L1 | -3.936022196 | -1.760796842 | **3** |
| DNAJB1 | -3.643495656 | -2.133977657 | **3** |
| ZNRD1 | -3.471270303 | -2.964544139 | **3** |
| GEM | -3.233530908 | -2.81259686 | **3** |
| ZNF234 | -3.209708261 | -3.148717229 | **3** |
| RABL4 | -3.167964496 | -1.995902133 | **3** |
| BCL11A | -3.135318838 | -2.840581906 | **3** |
| LOC339344 | -3.095507138 | -2.944922685 | **3** |
| DLG4 | -2.995705015 | -2.292822627 | **3** |
| OPN3 | -2.968291539 | -1.738470476 | **3** |
| HOXC4 | -2.898792281 | -1.756337407 | **3** |
| ZNRD1 | -2.862253818 | -2.696948987 | **3** |
| BANP | -2.822551133 | -2.050113525 | **3** |
| GMCL1 | -2.80101468 | -2.418902232 | **3** |
| GEM | -2.783843192 | -2.361755934 | **3** |
| BRI3BP | -2.75865895 | -1.893338559 | **3** |
| STAMBPL1 | -2.746869722 | -2.478985988 | **3** |
| C6orf166 | -2.715827613 | -2.298341811 | **3** |
| PLCXD1 | -2.643625717 | -1.922119337 | **3** |
| DDIT4 | -2.610200659 | -2.136836445 | **3** |
| TUT1 | -2.576702595 | -1.908940811 | **3** |
| MORC2 | -2.562331922 | -2.501996289 | **3** |
| LYAR | -2.561317058 | -2.291358787 | **3** |
| FZD2 | -2.536484555 | -1.652699425 | **3** |
| COPG2 | -2.534552369 | -2.374616372 | **3** |
| CKS2 | -2.477626713 | -2.019777699 | **3** |
| USP21 | -2.469735162 | -2.078285358 | **3** |
| THEM2 | -2.46760148 | -2.156485843 | **3** |
| SIRT5 | -2.454635559 | -1.533118718 | **3** |
| C20orf94 | -2.453949733 | -1.902957363 | **3** |
| CSNK2A2 | -2.437318487 | -1.8199675 | **3** |
| PDCD2L | -2.434988884 | -1.804598707 | **3** |
| FBXO22 | -2.429657515 | -1.541830157 | **3** |
| LMNB1 | -2.423989118 | -1.742333456 | **3** |
| CETN3 | -2.396430947 | -2.259760724 | **3** |
| C11orf60 | -2.392249487 | -2.055302284 | **3** |
| SC65 | -2.39155824 | -1.709659366 | **3** |
| ANKRD54 | -2.365661616 | -1.758392903 | **3** |
| TERF2 | -2.360688696 | -2.34375976 | **3** |
| TRIM46 | -2.352736516 | -1.69046921 | **3** |
| COL4A3BP | -2.344802028 | -1.795728309 | **3** |
| KLHL9 | -2.308219556 | -1.830675737 | **3** |
| PREB | -2.297095458 | -1.86169068 | **3** |
| SNHG3-RCC1 | -2.277608101 | -1.917163355 | **3** |
| RAB24 | -2.227847849 | -1.725861375 | **3** |
| DBT | -2.183401668 | -1.667206502 | **3** |
| TRIOBP | -2.163789838 | -1.983291527 | **3** |
| ZCCHC8 | -2.142462934 | -1.891105734 | **3** |
| NBPF1 | -2.134219349 | -1.602466517 | **3** |
| SUGT1 | -2.108667609 | -2.04985954 | **3** |
| PIF1 | -2.093337312 | -1.724408414 | **3** |
| FKBP14 | -2.061227178 | -1.698313909 | **3** |
| SGOL1 | -2.005527715 | -1.848104469 | **3** |
| PPP3R1 | -1.993264497 | -1.885732679 | **3** |
| UPF2 | -1.970635082 | -1.704657205 | **3** |
| SPATA5L1 | -1.941474117 | -1.607871938 | **3** |
| RILPL1 | -1.89272207 | -1.842506061 | **3** |
| PPP2R2A | -1.869092683 | -1.790442341 | **3** |
| PDCD7 | -1.805367736 | -1.547325727 | **3** |
| FAM58A | -1.719398791 | -1.548869341 | **3** |
| C17orf63 | -1.683035513 | -1.618597421 | **3** |
| TMEM79 | -1.678677682 | -1.532801407 | **3** |
| GPAM | -1.67234836 | -1.515032934 | **3** |
| MED28 | -1.649180079 | -1.615098227 | **3** |
| HSPA1A | -5.220916828 | -5.976379008 | **4** |
| INTS6 | -2.967749751 | -5.005607165 | **4** |
| KCNJ8 | -3.029911923 | -4.655907359 | **4** |
| PANK1 | -2.636825362 | -4.387508951 | **4** |
| EXOSC3 | -4.080583555 | -4.340580104 | **4** |
| ASCL1 | -3.663674515 | -4.108159569 | **4** |
| RAB23 | -3.079104814 | -4.105617644 | **4** |
| TRIM4 | -2.75782209 | -4.101549013 | **4** |
| MTERFD1 | -3.306064256 | -3.926907965 | **4** |
| ZMYM6 | -3.118394026 | -3.837495281 | **4** |
| ZNF234 | -3.096343497 | -3.807268699 | **4** |
| KRCC1 | -2.620334068 | -3.790315543 | **4** |
| ZCCHC9 | -2.321992626 | -3.578621679 | **4** |
| TAF1A | -2.937254254 | -3.530800378 | **4** |
| ARMC7 | -3.425538448 | -3.513025258 | **4** |
| RNF14 | -1.970594619 | -3.44882563 | **4** |
| MUTED | -2.093068609 | -3.364671802 | **4** |
| SPOP | -2.62336417 | -3.346180246 | **4** |
| MTERFD1 | -2.112616839 | -3.341587892 | **4** |
| BFAR | -2.839380862 | -3.309904572 | **4** |
| PROSC | -3.02631563 | -3.283386533 | **4** |
| PHF14 | -1.889942005 | -3.262460705 | **4** |
| SAR1B | -2.218503275 | -3.25131903 | **4** |
| HOXC6 | -2.794510217 | -3.243782859 | **4** |
| TRIM13 | -2.880630251 | -3.227786846 | **4** |
| AURKA | -1.930025422 | -3.211388016 | **4** |
| DTX2 | -2.980447823 | -3.126320165 | **4** |
| MGC12966 | -2.770299207 | -3.117452472 | **4** |
| NUP37 | -2.304588842 | -3.084270079 | **4** |
| CCDC117 | -2.048134165 | -3.080548833 | **4** |
| HEY1 | -2.43368366 | -3.048143213 | **4** |
| GUCY1A3 | -2.654947465 | -3.033515836 | **4** |
| THAP10 | -2.294154332 | -3.016605648 | **4** |
| MITD1 | -2.032934001 | -3.004233861 | **4** |
| ZNF330 | -1.701632858 | -2.986288426 | **4** |
| KIAA0895 | -1.85450335 | -2.948948118 | **4** |
| MFAP1 | -1.912746165 | -2.934336955 | **4** |
| KBTBD7 | -1.982729185 | -2.924205935 | **4** |
| FAM48A | -2.790010453 | -2.889847721 | **4** |
| ANKRD46 | -1.598300436 | -2.865660253 | **4** |
| IL10RB | -2.221039073 | -2.835682913 | **4** |
| TIMM9 | -2.772978394 | -2.795062475 | **4** |
| ZFYVE21 | -2.172610761 | -2.778778545 | **4** |
| ZFP3 | -1.962994991 | -2.771650192 | **4** |
| TICAM1 | -2.380574961 | -2.748574354 | **4** |
| LOC653820 | -1.847628731 | -2.732243505 | **4** |
| NUPL2 | -1.770020093 | -2.70743977 | **4** |
| CNIH4 | -1.640196721 | -2.693555572 | **4** |
| TRK1 | -2.270636315 | -2.686601194 | **4** |
| TRIM32 | -2.384858012 | -2.682169159 | **4** |
| LMAN2L | -2.272057279 | -2.682133809 | **4** |
| FLRT3 | -2.100389942 | -2.650954361 | **4** |
| DTWD1 | -2.619167294 | -2.649049344 | **4** |
| HSPA8 | -1.998819263 | -2.64429698 | **4** |
| EXOSC3 | -2.249575585 | -2.606379985 | **4** |
| KIAA1279 | -2.17917296 | -2.597893946 | **4** |
| WDR67 | -2.320985306 | -2.595198928 | **4** |
| PHF23 | -1.947719771 | -2.593789873 | **4** |
| GART | -1.892171568 | -2.549865693 | **4** |
| RASSF7 | -2.116655617 | -2.537539003 | **4** |
| ASB8 | -2.345913909 | -2.537302166 | **4** |
| BCDIN3D | -1.608386457 | -2.526947861 | **4** |
| C8orf41 | -1.982667174 | -2.50639387 | **4** |
| ZNRD1 | -2.362517119 | -2.504483623 | **4** |
| C16orf72 | -2.091328934 | -2.502417238 | **4** |
| PHF14 | -2.180383255 | -2.47362292 | **4** |
| MTIF3 | -1.96859334 | -2.462471165 | **4** |
| C3orf31 | -1.708155676 | -2.452468172 | **4** |
| CKS2 | -2.219705923 | -2.426798613 | **4** |
| UPF3B | -1.729497691 | -2.426456804 | **4** |
| BTBD10 | -1.868428714 | -2.404254618 | **4** |
| C9orf23 | -2.252752499 | -2.403473325 | **4** |
| HSPA8 | -1.986930405 | -2.401687196 | **4** |
| HRSP12 | -1.887808673 | -2.38663502 | **4** |
| ATP6V1D | -1.690671175 | -2.378865879 | **4** |
| NRSN1 | -1.787523941 | -2.349015715 | **4** |
| KRIT1 | -1.843355429 | -2.344879187 | **4** |
| GTF2E1 | -1.927131683 | -2.343216505 | **4** |
| MRPS31 | -1.585859432 | -2.312990081 | **4** |
| CEPT1 | -1.569496001 | -2.303303373 | **4** |
| ASB8 | -2.139136433 | -2.28421457 | **4** |
| LYAR | -1.848994235 | -2.266008025 | **4** |
| GEMIN6 | -1.557396252 | -2.214150061 | **4** |
| PRUNE | -1.501134146 | -2.212681318 | **4** |
| LOC401720 | -1.605630969 | -2.209389713 | **4** |
| SGOL1 | -1.87363872 | -2.166845842 | **4** |
| ZNF260 | -1.768431313 | -2.147297425 | **4** |
| DNAJB2 | -1.665599464 | -2.130575403 | **4** |
| BTBD7 | -1.94226872 | -2.124289698 | **4** |
| GTF2E2 | -1.811446756 | -2.082461964 | **4** |
| RTCD1 | -1.932799768 | -2.076740308 | **4** |
| TMEM22 | -1.698548763 | -2.058930782 | **4** |
| OIP5 | -1.879631612 | -2.058863842 | **4** |
| C1orf131 | -1.945009681 | -2.047568087 | **4** |
| TBPL1 | -1.804592108 | -2.044864927 | **4** |
| PIAS1 | -1.930198764 | -2.025359473 | **4** |
| CENPA | -1.562400603 | -1.966322614 | **4** |
| LYPLAL1 | -1.662209738 | -1.957916487 | **4** |
| NDEL1 | -1.699765217 | -1.953220442 | **4** |
| LOC730432 | -1.685055674 | -1.936674094 | **4** |
| ZFYVE19 | -1.84862877 | -1.916590635 | **4** |
| C8orf70 | -1.89597539 | -1.898055332 | **4** |
| C1orf181 | -1.747520608 | -1.888384498 | **4** |
| DCUN1D3 | -1.773322398 | -1.853769518 | **4** |
| NSUN4 | -1.741065162 | -1.807575385 | **4** |
| LIX1L | -1.521339348 | -1.804923797 | **4** |
| C20orf108 | -1.572625136 | -1.792428566 | **4** |
| REXO2 | -1.662137297 | -1.765237108 | **4** |
| DYNLL1 | -1.648439335 | -1.737915706 | **4** |
| LOC644096 | -1.720748786 | -1.734499387 | **4** |
| RALA | -1.640137572 | -1.702419132 | **4** |
| RAD51C | -1.581612326 | -1.683161343 | **4** |
| KIAA1160 | -1.500727191 | -1.676601845 | **4** |
| DNAJA1 | -1.587843181 | -1.662840119 | **4** |
| FAM120B | -1.581633737 | -1.626283262 | **4** |
| LOC642333 | -1.526810479 | -1.618699458 | **4** |
| ZAK | -1.517092544 | -1.563825579 | **4** |
| TSC22D1 | 1.942527676 | -2.317259743 | **5** |
