# Supplementary material for: Minimal Peroxide Exposure of Neuronal Cells Induces Multifaceted Adaptive Responses
Source: PLoS One. 2010 Dec 17;5(12):e14352. doi: 10.1371/journal.pone.0014352 (PMC3003681; doi:10.1371/journal.pone.0014352)
Supplement: Table S16 — Common 4 hour MeCh-regulated gene series. MeCh-regulated genes that were significantly elevated or reduced compared to the respective unstimulated control cells in both the control (untreated: MeCh-4h-Control vs. Control-Control) and CMP state SH-SY5Y cells (MeCh-4h-CMP vs. Control-CMP). The series number refers to the simplistic relationships between the degree of regulation of the respective genes and the cellular state (untreated or CMP). Series 1 (both upregulated) - MeCh-4h-CMP vs. Control-CMP > MeCh-4h-Control vs. Control-Control; Series 2 (both upregulated) - MeCh-4h-Control vs. Control-Control > MeCh-4h-CMP vs. Control-CMP; Series 3 (both downregulated) - MeCh-4h-Control vs. Control-Control > MeCh-4h-CMP vs. Control-CMP; Series 4 (both downregulated) - MeCh-4h-CMP vs. Control-CMP > MeCh-4h-Control vs. Control-Control; Series 5 (downregulated in control, upregulated in CMP); Series 6 (upregulated in control, down regulated in CMP). (0.57 MB DOC) [file pone.0014352.s023.doc]

**Table S16. Common 4 hour MeCh-regulated gene series.** MeCh-regulated genes that were significantly elevated or reduced compared to the respective unstimulated control cells in both the control (untreated: *MeCh-4h-Control vs. Control-Control*) and CMP state SH-SY5Y cells (*MeCh-4h-CMP vs. Control-CMP*). The series number refers to the simplistic relationships between the degree of regulation of the respective genes and the cellular state (untreated or CMP). Series 1 (both upregulated) - *MeCh-4h-CMP vs. Control-CMP* > *MeCh-4h-Control vs. Control-Control*; Series 2 (both upregulated) - *MeCh-4h-Control vs. Control-Control* > *MeCh-4h-CMP vs. Control-CMP*; Series 3 (both downregulated) - *MeCh-4h-Control vs. Control-Control* > *MeCh-4h-CMP vs. Control-CMP*; Series 4 (both downregulated) - *MeCh-4h-CMP vs. Control-CMP* > *MeCh-4h-Control vs. Control-Control*; Series 5 (downregulated in control, upregulated in CMP); Series 6 (upregulated in control, down regulated in CMP).

| **SYMBOL** | **MeCh-2h-CMP vs. Control-CMP** | **MeCh-2h-Control vs. Control-Control** | **Series #** |
| --- | --- | --- | --- |
| IL8 | 9.967503371 | 8.121248925 | **1** |
| H3F3B | 5.865971189 | 5.017475127 | **1** |
| LOC653994 | 5.304875227 | 4.424538869 | **1** |
| TNFRSF19 | 4.992371528 | 4.625852969 | **1** |
| SUV420H1 | 4.53908486 | 4.077204259 | **1** |
| KIAA0644 | 4.44391343 | 4.229498973 | **1** |
| SLC11A2 | 4.397010962 | 3.112565403 | **1** |
| MGEA5 | 4.356441422 | 2.884526905 | **1** |
| CSTF3 | 4.352204665 | 4.05300002 | **1** |
| TNPO1 | 4.199990834 | 2.268816846 | **1** |
| LANCL2 | 3.978332813 | 3.708263282 | **1** |
| BTBD11 | 3.841609184 | 2.010274024 | **1** |
| FAT | 3.687702272 | 3.063968651 | **1** |
| CD44 | 3.667476011 | 2.687669029 | **1** |
| NPEPL1 | 3.662110686 | 3.4170133 | **1** |
| CAMSAP1 | 3.590667751 | 2.491130209 | **1** |
| RNF150 | 3.551995498 | 3.168357384 | **1** |
| LRP5L | 3.528629639 | 3.410748012 | **1** |
| ERRFI1 | 3.311637282 | 2.715347441 | **1** |
| ZNF275 | 3.283935443 | 2.705168286 | **1** |
|  | 3.282735452 | 3.186510063 | **1** |
| RAD23B | 3.255997395 | 2.775585072 | **1** |
| SEMA6A | 3.212086234 | 2.684442843 | **1** |
| IHPK1 | 3.157639403 | 2.645226155 | **1** |
| CRY1 | 3.153647502 | 2.651325673 | **1** |
| BTAF1 | 3.135431077 | 2.334335769 | **1** |
| LOC727935 | 3.115031077 | 3.079787192 | **1** |
| ZNF789 | 3.111509344 | 2.562977461 | **1** |
| ARID5B | 3.108725909 | 2.427296747 | **1** |
| TNFSF14 | 3.088148584 | 1.68893009 | **1** |
| P2RY11 | 3.067152598 | 2.72203885 | **1** |
| IL18BP | 3.000236853 | 2.457928681 | **1** |
| RBM33 | 2.987672329 | 2.969656805 | **1** |
| TNS3 | 2.984151289 | 2.588117935 | **1** |
| LOC285074 | 2.954088404 | 2.74638582 | **1** |
| RIMS3 | 2.849243296 | 2.444369286 | **1** |
| RNF165 | 2.768991133 | 2.667842302 | **1** |
| ASCC3L1 | 2.745099051 | 2.078446379 | **1** |
| ATP6V0A2 | 2.684021475 | 2.077547398 | **1** |
| RHBDL2 | 2.674537755 | 1.852180372 | **1** |
| C1orf21 | 2.659783294 | 1.577650545 | **1** |
| SLC7A1 | 2.612585591 | 1.906104978 | **1** |
| LOC146517 | 2.590421104 | 2.113756793 | **1** |
| ZNF212 | 2.578944921 | 2.304817079 | **1** |
| SYT11 | 2.568795034 | 1.885339937 | **1** |
| LOC401357 | 2.560747229 | 2.4269337 | **1** |
| SYNCRIP | 2.553870231 | 2.507730013 | **1** |
| SPG7 | 2.524538397 | 1.780695455 | **1** |
| ZNF536 | 2.512976649 | 1.793141988 | **1** |
| GATS | 2.508954483 | 1.945326386 | **1** |
| ARID3B | 2.494256313 | 2.120449858 | **1** |
| SIPA1L2 | 2.465829585 | 2.385084689 | **1** |
| C6orf134 | 2.463867216 | 2.436490912 | **1** |
| CSF2RA | 2.40592538 | 1.690916753 | **1** |
| ASB1 | 2.405280359 | 1.593013935 | **1** |
| PDE7A | 2.383449306 | 1.712979049 | **1** |
| HNRPK | 2.360427247 | 1.828350016 | **1** |
| HMGB3 | 2.357865687 | 1.775726236 | **1** |
| PPM1A | 2.355704983 | 2.017354565 | **1** |
| C14orf102 | 2.353740366 | 2.028704947 | **1** |
| MTMR3 | 2.332229355 | 2.195950852 | **1** |
| KIAA0194 | 2.328583996 | 1.519950257 | **1** |
| ATP9A | 2.295831647 | 2.149534029 | **1** |
| ORAOV1 | 2.287974805 | 2.048922483 | **1** |
| SHPK | 2.261903578 | 2.097644889 | **1** |
| FOXJ3 | 2.228004752 | 1.860486395 | **1** |
| FKTN | 2.194548289 | 1.850667261 | **1** |
| PHF17 | 2.180362769 | 1.955308143 | **1** |
| MEF2D | 2.170389437 | 1.994246729 | **1** |
| FEM1A | 2.153376223 | 1.808430478 | **1** |
| LOC400027 | 2.108422245 | 1.753229675 | **1** |
| C3orf59 | 2.102995357 | 1.55564364 | **1** |
| NOL6 | 2.069152191 | 1.82024496 | **1** |
| DNAJB6 | 2.065785898 | 1.600046097 | **1** |
| C8orf33 | 2.055157994 | 1.596582199 | **1** |
| GCH1 | 2.053370814 | 2.043048226 | **1** |
| BAT3 | 2.04919598 | 1.617446026 | **1** |
| DDX42 | 2.029877609 | 1.83712408 | **1** |
| ZBED4 | 2.014623448 | 1.972157435 | **1** |
| ANKRD30B | 2.003834819 | 1.541452363 | **1** |
| DHX9 | 1.956042446 | 1.68062021 | **1** |
| DDX18 | 1.915269449 | 1.862847267 | **1** |
| UBE2G1 | 1.91409993 | 1.63939975 | **1** |
| GMEB2 | 1.906667355 | 1.792535083 | **1** |
| ETV5 | 1.891226808 | 1.780896281 | **1** |
| TSKU | 1.884739386 | 1.606193647 | **1** |
| THOC5 | 1.879333328 | 1.710262908 | **1** |
| MAPRE2 | 1.878705042 | 1.788016978 | **1** |
| PIK4CA | 1.856703361 | 1.761705785 | **1** |
| GNPTG | 1.837562369 | 1.731067817 | **1** |
| TUB | 1.828064181 | 1.695573381 | **1** |
| CATSPER2 | 1.80959706 | 1.559368059 | **1** |
| ANKRD11 | 1.807750148 | 1.634108318 | **1** |
| CDCA4 | 1.806518181 | 1.619154155 | **1** |
| SIN3A | 1.799783538 | 1.709309044 | **1** |
| WASF3 | 1.793319258 | 1.500251802 | **1** |
| FOXO3 | 1.773296967 | 1.690429482 | **1** |
| SFRS5 | 1.704316131 | 1.701090985 | **1** |
| PRKRIP1 | 1.696468565 | 1.55184953 | **1** |
| STOX2 | 1.696281065 | 1.633866313 | **1** |
| UHRF1 | 1.691655223 | 1.67308455 | **1** |
| UBN1 | 1.688687553 | 1.540450791 | **1** |
| MCM2 | 1.664064159 | 1.528052978 | **1** |
| LOC401720 | 1.613754388 | 1.553384794 | **1** |
| GLTSCR1 | 1.547209788 | 1.50534307 | **1** |
| PLXNB1 | 3.433852051 | 4.325179185 | **2** |
| GAB2 | 3.376485803 | 3.846280525 | **2** |
| CALD1 | 3.561935556 | 3.840212319 | **2** |
| KLF13 | 1.929954385 | 3.807341968 | **2** |
| SNAPC4 | 3.259467438 | 3.681657245 | **2** |
| RHBDD2 | 2.283789945 | 3.681128425 | **2** |
| ZYX | 2.585467309 | 3.668826145 | **2** |
| TNC | 3.149400445 | 3.57811147 | **2** |
| RHBDD2 | 3.275918219 | 3.559770661 | **2** |
| MYH9 | 2.558270024 | 3.439989217 | **2** |
| TACC2 | 1.547493451 | 3.360560057 | **2** |
| SP2 | 1.962726621 | 3.309480542 | **2** |
| MNT | 2.165957654 | 3.245076 | **2** |
| INTS1 | 3.170486738 | 3.207390127 | **2** |
| ITGB5 | 3.001033538 | 3.099227381 | **2** |
| SGSH | 1.786481821 | 3.07003324 | **2** |
| SUV420H1 | 2.948829912 | 3.036293008 | **2** |
| CBX2 | 2.230065571 | 3.028878231 | **2** |
| TGIF1 | 1.972510494 | 3.013664309 | **2** |
| MCM5 | 1.710074499 | 2.980462811 | **2** |
| KLF6 | 2.512886434 | 2.980073137 | **2** |
| TTC32 | 2.374397548 | 2.943957351 | **2** |
| FHL2 | 2.251507375 | 2.863767822 | **2** |
| VCL | 2.713221138 | 2.85843985 | **2** |
| ZNF407 | 1.954409631 | 2.829063169 | **2** |
| FHL2 | 2.605783477 | 2.828995094 | **2** |
| PDPR | 2.439300138 | 2.817084938 | **2** |
| KLF6 | 2.091116301 | 2.794999709 | **2** |
| CEBPB | 2.234814269 | 2.7577845 | **2** |
| ZNF536 | 2.404540441 | 2.732066845 | **2** |
| C9orf156 | 2.604910645 | 2.704479857 | **2** |
| HCFC1 | 2.25445926 | 2.692937142 | **2** |
| LOC338758 | 2.249288187 | 2.691438607 | **2** |
| KIAA1545 | 1.671895381 | 2.687508546 | **2** |
| ULK1 | 1.796453315 | 2.673001552 | **2** |
| PERLD1 | 1.961483195 | 2.649664695 | **2** |
| LOC653103 | 2.265183977 | 2.641153676 | **2** |
| CIRBP | 2.087284203 | 2.631205954 | **2** |
| FBXO31 | 2.294114234 | 2.609354605 | **2** |
| ERF | 2.17738045 | 2.544349909 | **2** |
| PFKFB3 | 2.177910171 | 2.542671379 | **2** |
| ABL1 | 2.409742779 | 2.516732594 | **2** |
| RHBDF1 | 2.293551768 | 2.495428254 | **2** |
| DUSP3 | 2.430752525 | 2.491533173 | **2** |
| TMEM57 | 1.900264239 | 2.468303851 | **2** |
| FAM125B | 1.757162086 | 2.465235479 | **2** |
| NGFRAP1 | 1.932782353 | 2.445869491 | **2** |
| DGCR8 | 1.650805367 | 2.443289765 | **2** |
| C4orf30 | 1.771531483 | 2.432915082 | **2** |
| SLC25A26 | 1.91788707 | 2.40613496 | **2** |
| MSI2 | 1.879451388 | 2.403465169 | **2** |
| ANKRD13D | 2.187446817 | 2.399998509 | **2** |
| FKSG30 | 1.646271931 | 2.399591508 | **2** |
| GNG11 | 1.568120004 | 2.382447404 | **2** |
| RNF4 | 2.20143078 | 2.382086955 | **2** |
| LOC132241 | 2.024034969 | 2.378886405 | **2** |
| ASXL1 | 1.609166717 | 2.351295424 | **2** |
| PPFIBP1 | 2.240899815 | 2.300092339 | **2** |
| ABCC5 | 1.627550322 | 2.279265068 | **2** |
| VGLL4 | 1.862754765 | 2.247955566 | **2** |
| PPP1R15A | 2.24102498 | 2.247011343 | **2** |
| BRPF1 | 2.051111053 | 2.246800615 | **2** |
| CTNNBIP1 | 2.012720181 | 2.230763699 | **2** |
| NDRG4 | 1.998319718 | 2.224812271 | **2** |
| ULK1 | 1.827443748 | 2.222355499 | **2** |
| SECISBP2 | 2.174763843 | 2.208682611 | **2** |
| SBF1 | 1.616686421 | 2.20335149 | **2** |
| ATP5S | 1.693088281 | 2.202770896 | **2** |
| OCIAD2 | 1.982378891 | 2.197698115 | **2** |
| CLCN7 | 1.909283381 | 2.196582075 | **2** |
| RNF4 | 1.872159915 | 2.194737521 | **2** |
| KCNT1 | 1.574591503 | 2.185137604 | **2** |
| SLC10A4 | 1.609046024 | 2.181797238 | **2** |
| SEC61A1 | 1.856365744 | 2.11824533 | **2** |
| E4F1 | 1.910308352 | 2.117901275 | **2** |
| ATP1B1 | 1.682845566 | 2.105791386 | **2** |
| AMFR | 1.760532969 | 2.096830736 | **2** |
| INPPL1 | 1.544774662 | 2.094382442 | **2** |
| PDCD2 | 1.555376963 | 2.093657189 | **2** |
| CPSF4 | 1.791451874 | 2.076016712 | **2** |
| KIAA1949 | 1.846349796 | 2.066230203 | **2** |
| SRF | 1.636756566 | 2.051230454 | **2** |
| PMEPA1 | 1.59726796 | 2.050801805 | **2** |
| SPECC1L | 1.814930115 | 2.022453417 | **2** |
| ZCCHC14 | 1.544946053 | 2.004227877 | **2** |
| GRAMD4 | 1.914651153 | 1.999724708 | **2** |
| ZNF746 | 1.84858063 | 1.986186978 | **2** |
| SPRED2 | 1.809800944 | 1.980252465 | **2** |
| ARHGAP23 | 1.59217144 | 1.962205406 | **2** |
| RAI1 | 1.820790523 | 1.942028155 | **2** |
| PARL | 1.693792892 | 1.92593875 | **2** |
| KLHDC4 | 1.525351998 | 1.920212037 | **2** |
| RAPGEF1 | 1.568970723 | 1.906235619 | **2** |
| LAMP1 | 1.600964466 | 1.905792093 | **2** |
| CDK6 | 1.808962884 | 1.871115292 | **2** |
| COX19 | 1.613971459 | 1.796653506 | **2** |
| C7orf20 | 1.568409837 | 1.791127755 | **2** |
| NSFL1C | 1.516133255 | 1.78593647 | **2** |
| ADAR | 1.743039851 | 1.771817326 | **2** |
| ZC3H5 | 1.605409423 | 1.757706741 | **2** |
| MAP2 | 1.531670285 | 1.713753158 | **2** |
| RGS12 | 1.599096515 | 1.6997112 | **2** |
| SPEN | 1.651771672 | 1.697999947 | **2** |
| THOC5 | 1.603128336 | 1.67695485 | **2** |
| TP53BP2 | 1.60485175 | 1.663996867 | **2** |
| SON | 1.603265399 | 1.625187637 | **2** |
| RASSF1 | 1.51171055 | 1.619001801 | **2** |
| HSPA1B | -6.541865574 | -6.131840102 | **3** |
| MSL3L1 | -4.873880015 | -2.096470066 | **3** |
| MORC2 | -4.269393971 | -3.630609613 | **3** |
| NBPF20 | -4.183066846 | -1.60841843 | **3** |
| ID3 | -4.134502859 | -3.968058918 | **3** |
| RABL4 | -4.103738188 | -2.640283661 | **3** |
| EZH2 | -4.014259015 | -2.573894744 | **3** |
| HIST1H2AC | -3.961026292 | -2.434939753 | **3** |
| MTUS1 | -3.720644523 | -1.829167634 | **3** |
| THEM2 | -3.656471657 | -3.269557994 | **3** |
| ID2 | -3.645077298 | -3.00038748 | **3** |
| HIST1H2BD | -3.595745478 | -2.206530913 | **3** |
| HIST1H2BD | -3.584320368 | -3.075203994 | **3** |
| OPN3 | -3.486451143 | -3.140094754 | **3** |
| RGS19 | -3.344300394 | -1.77692589 | **3** |
| TERF1 | -3.222139009 | -2.470710076 | **3** |
| UBFD1 | -3.212346466 | -2.858733829 | **3** |
| C9orf23 | -3.140648837 | -1.609343413 | **3** |
| C16orf14 | -3.133496558 | -1.818333648 | **3** |
| BRI3BP | -3.122972293 | -2.105976674 | **3** |
| TMEM5 | -3.022403267 | -2.958200113 | **3** |
| UBAP2L | -3.01336589 | -1.537633743 | **3** |
| LOC440160 | -2.997853396 | -2.481343409 | **3** |
| LOC402560 | -2.992070605 | -2.507108824 | **3** |
| TRIOBP | -2.985801577 | -2.025679087 | **3** |
| DYNLL1 | -2.961222401 | -2.086682789 | **3** |
| RAD21 | -2.909556343 | -1.667273353 | **3** |
| GMCL1 | -2.889191058 | -2.6165007 | **3** |
| OIP5 | -2.844890451 | -2.793695357 | **3** |
| LOC441763 | -2.836902127 | -1.536151411 | **3** |
| PPAPDC3 | -2.81986219 | -2.339009726 | **3** |
| RILPL1 | -2.803693301 | -2.53398913 | **3** |
| PLCXD1 | -2.79153244 | -1.644477305 | **3** |
| C16orf53 | -2.775071169 | -2.254245884 | **3** |
| C11orf60 | -2.720860607 | -2.535315686 | **3** |
| CCDC34 | -2.705838136 | -1.950237215 | **3** |
| LOC375295 | -2.699352767 | -2.407643393 | **3** |
| DNAJB6 | -2.682558101 | -1.918295781 | **3** |
| GMCL1 | -2.672987027 | -2.588033435 | **3** |
| BANP | -2.662042456 | -2.382024258 | **3** |
| ZNF503 | -2.655035409 | -2.35518269 | **3** |
| LOC642282 | -2.653206316 | -1.564534595 | **3** |
| RAB24 | -2.634508176 | -1.902876051 | **3** |
| RABEPK | -2.621272123 | -2.526289814 | **3** |
| GMPPB | -2.571782421 | -1.775236128 | **3** |
| C21orf51 | -2.554733804 | -2.14215892 | **3** |
| ABHD8 | -2.552370437 | -1.731405543 | **3** |
| DNCL1 | -2.523920927 | -2.439861774 | **3** |
| TM7SF3 | -2.512981335 | -2.19077678 | **3** |
| TAF12 | -2.511323588 | -2.398041796 | **3** |
| IPO7 | -2.510987691 | -1.796554549 | **3** |
| C16orf53 | -2.509049222 | -1.878525875 | **3** |
| CETN3 | -2.504063798 | -2.465567134 | **3** |
| FBXO22 | -2.496798194 | -1.538343379 | **3** |
| BFAR | -2.488143879 | -1.779815691 | **3** |
| NTHL1 | -2.451159038 | -1.607409305 | **3** |
| C1orf86 | -2.440732274 | -2.269189065 | **3** |
| MTIF3 | -2.401589409 | -2.234828154 | **3** |
| LRSAM1 | -2.391254705 | -2.040550967 | **3** |
| LOC400879 | -2.362029088 | -1.626966522 | **3** |
| DPY30 | -2.360625292 | -2.110870073 | **3** |
| C11orf73 | -2.347987762 | -1.547919732 | **3** |
| EXOSC3 | -2.334802628 | -2.212208796 | **3** |
| MRPS33 | -2.32190077 | -2.294541715 | **3** |
| KCTD12 | -2.319207626 | -1.519874741 | **3** |
| MRPL22 | -2.31166933 | -1.683040973 | **3** |
| CDKN3 | -2.310473525 | -1.695992934 | **3** |
| GALNT11 | -2.303698158 | -1.962223466 | **3** |
| MRPL52 | -2.291183748 | -1.891899649 | **3** |
| THOC2 | -2.290648393 | -1.809053424 | **3** |
| NSL1 | -2.251940199 | -1.857657276 | **3** |
| THOC3 | -2.228716144 | -1.651167441 | **3** |
| APTX | -2.207482755 | -1.843867617 | **3** |
| TIMM9 | -2.160272462 | -1.899800567 | **3** |
| MRPL18 | -2.151080712 | -1.865607771 | **3** |
| PPM1G | -2.149414414 | -1.744408366 | **3** |
| PCCA | -2.110747734 | -1.726052737 | **3** |
| CSNK2A2 | -2.110531671 | -1.681126598 | **3** |
| RBM4 | -2.103126662 | -1.779693271 | **3** |
| TRK1 | -2.093690122 | -1.584213325 | **3** |
| LSM2 | -2.062020058 | -1.672674221 | **3** |
| CHMP4A | -2.054556895 | -1.583513168 | **3** |
| BOLA3 | -2.032146182 | -1.626319442 | **3** |
| MFAP1 | -1.992292207 | -1.910392842 | **3** |
| SEPX1 | -1.98492438 | -1.946554223 | **3** |
| PHOX2B | -1.983719966 | -1.843886571 | **3** |
| C1orf43 | -1.982263371 | -1.539077849 | **3** |
| ZFYVE19 | -1.968457533 | -1.864684555 | **3** |
| NUDT5 | -1.947121819 | -1.591301206 | **3** |
| MGST2 | -1.930371832 | -1.707181189 | **3** |
| ANAPC11 | -1.928637453 | -1.563176896 | **3** |
| PDE6D | -1.923286108 | -1.52148202 | **3** |
| MRPL24 | -1.905497879 | -1.767370766 | **3** |
| CISD1 | -1.899998767 | -1.504194423 | **3** |
| MED30 | -1.896586859 | -1.859197021 | **3** |
| BUB3 | -1.847775329 | -1.595598974 | **3** |
| PHYHIPL | -1.823975231 | -1.652237187 | **3** |
| C3orf68 | -1.809911476 | -1.505931841 | **3** |
| POLDIP3 | -1.766364154 | -1.542942731 | **3** |
| LOC642333 | -1.756844503 | -1.603740826 | **3** |
| KLHL9 | -1.657557336 | -1.613379285 | **3** |
| MED28 | -1.628640918 | -1.592117062 | **3** |
| STYXL1 | -1.554395849 | -1.52922004 | **3** |
| HSPA1A | -5.726487151 | -6.900450388 | **4** |
| PHF14 | -3.000795094 | -5.070790925 | **4** |
| NRSN1 | -4.086201051 | -4.921554606 | **4** |
| HRSP12 | -2.53787216 | -4.357808486 | **4** |
| ASCL1 | -4.093617222 | -4.303173979 | **4** |
| HSPA8 | -3.352196485 | -4.225928354 | **4** |
| SLC35B3 | -2.8992749 | -4.070804139 | **4** |
| ELOVL6 | -2.80959157 | -4.037084588 | **4** |
| SMARCAL1 | -3.017734542 | -4.020867798 | **4** |
| MGC16169 | -2.235772147 | -3.943095928 | **4** |
| PROSC | -3.22343945 | -3.90804478 | **4** |
| GART | -3.736025834 | -3.797633331 | **4** |
| ID2 | -3.241457353 | -3.785585205 | **4** |
| DNAJA1 | -2.526524171 | -3.754592265 | **4** |
| HSPA8 | -2.904307019 | -3.754111997 | **4** |
| C3orf31 | -3.318241802 | -3.718118489 | **4** |
| RAD51C | -3.10959217 | -3.698205569 | **4** |
| GUCY1A3 | -2.680690308 | -3.694106718 | **4** |
| SCN2A | -3.433886731 | -3.618006351 | **4** |
| FLRT3 | -2.666775884 | -3.59606895 | **4** |
| BANP | -3.366924577 | -3.564546439 | **4** |
| TMEM126B | -1.874247707 | -3.498072813 | **4** |
| PHF14 | -2.7127768 | -3.468663972 | **4** |
| CCDC102A | -2.868705643 | -3.466067006 | **4** |
| CEPT1 | -2.377270476 | -3.438088583 | **4** |
| NARS2 | -1.752538753 | -3.430265276 | **4** |
| SLC35D3 | -2.555239812 | -3.426089222 | **4** |
| SLC35A1 | -2.567863944 | -3.370847579 | **4** |
| TPST2 | -2.364940338 | -3.342396287 | **4** |
| ZCCHC7 | -2.862694089 | -3.339820536 | **4** |
| USO1 | -1.595083013 | -3.310543069 | **4** |
| RAD51C | -2.532199673 | -3.303229414 | **4** |
| CRYZ | -1.503648287 | -3.295184877 | **4** |
| FOXN4 | -2.281109326 | -3.276550671 | **4** |
| XPO1 | -2.191132165 | -3.270006031 | **4** |
| GTF2E2 | -2.55773132 | -3.224909588 | **4** |
| PIGF | -2.947337167 | -3.192037784 | **4** |
| LMAN2L | -2.999575495 | -3.173750249 | **4** |
| CHCHD3 | -2.385411782 | -3.107227456 | **4** |
| SLC9A3R1 | -2.718560998 | -3.079859151 | **4** |
| CSTF3 | -2.034239942 | -3.057645409 | **4** |
| NUP37 | -1.737522878 | -3.038909347 | **4** |
| ZMYM6 | -2.70040163 | -3.028458087 | **4** |
| SC4MOL | -2.05878204 | -2.988466022 | **4** |
| INSIG1 | -1.578060586 | -2.984598954 | **4** |
| C12orf11 | -1.906030555 | -2.984275114 | **4** |
| RNGTT | -2.185654716 | -2.973627116 | **4** |
| ADD3 | -1.694232375 | -2.968375816 | **4** |
| MKKS | -1.807881598 | -2.940229873 | **4** |
| ZAK | -1.898868098 | -2.929629867 | **4** |
| CCDC53 | -2.169684153 | -2.924779864 | **4** |
| REXO2 | -2.348059194 | -2.915065507 | **4** |
| SC4MOL | -2.677278189 | -2.874212658 | **4** |
| GPR162 | -2.670201054 | -2.833520845 | **4** |
| KLHL7 | -1.698204705 | -2.790955743 | **4** |
| MRPL13 | -1.806576335 | -2.787430718 | **4** |
| HSPH1 | -1.854121563 | -2.767369356 | **4** |
| IL10RB | -2.458074779 | -2.760340104 | **4** |
| HIBCH | -1.970362023 | -2.751278886 | **4** |
| RHOT1 | -1.504382823 | -2.742181367 | **4** |
| SDCCAG10 | -1.99832826 | -2.737333202 | **4** |
| MRPL35 | -2.010906972 | -2.711093182 | **4** |
| RHOT1 | -1.661482856 | -2.678167726 | **4** |
| ATM | -1.89822539 | -2.639155822 | **4** |
| SRBD1 | -1.557704459 | -2.623115171 | **4** |
| SAR1B | -1.651916571 | -2.58680977 | **4** |
| ALG8 | -1.663202215 | -2.562097317 | **4** |
| NCBP1 | -1.565468403 | -2.556557388 | **4** |
| KLRG1 | -2.457337841 | -2.541748624 | **4** |
| OPRL1 | -2.406190611 | -2.525272152 | **4** |
| C15orf52 | -1.921671178 | -2.520810587 | **4** |
| SFRS7 | -2.203513206 | -2.492385179 | **4** |
| CENPE | -1.849357152 | -2.486861111 | **4** |
| NUDT9 | -1.638204036 | -2.48071667 | **4** |
| DDX10 | -1.517909113 | -2.480662769 | **4** |
| PKP4 | -2.054195292 | -2.459205143 | **4** |
| TRIM4 | -1.808874868 | -2.448331993 | **4** |
| PIGP | -2.139634202 | -2.445651428 | **4** |
| LOC219854 | -2.231640808 | -2.444379486 | **4** |
| ZC3HAV1 | -1.622435429 | -2.422530819 | **4** |
| STK4 | -1.570402472 | -2.41787454 | **4** |
| P15RS | -1.705920138 | -2.405714818 | **4** |
| TMEM99 | -1.895386936 | -2.405303947 | **4** |
| RPUSD3 | -2.366257616 | -2.398935042 | **4** |
| C1orf25 | -1.518460438 | -2.373406306 | **4** |
| SIX5 | -1.609321782 | -2.368782634 | **4** |
| THAP10 | -2.129587554 | -2.349428688 | **4** |
| ZNF22 | -1.889896307 | -2.343790495 | **4** |
| PANK1 | -1.658373289 | -2.337911378 | **4** |
| DPH5 | -1.795289887 | -2.337087657 | **4** |
| SQLE | -1.552822635 | -2.315938833 | **4** |
| SHQ1 | -1.718303471 | -2.260894547 | **4** |
| BCAP29 | -1.85565034 | -2.259136922 | **4** |
| MGC57346 | -1.89320916 | -2.257842168 | **4** |
| MRPS6 | -2.159332655 | -2.257765061 | **4** |
| KCNJ8 | -1.730717572 | -2.251488545 | **4** |
| LARP6 | -1.96914839 | -2.244038588 | **4** |
| C14orf142 | -1.810191811 | -2.237155716 | **4** |
| NOL4 | -1.933959338 | -2.231255518 | **4** |
| GNE | -1.802849723 | -2.219264418 | **4** |
| MRPS31 | -1.672611757 | -2.200998339 | **4** |
| RN7SK | -2.15102007 | -2.20052815 | **4** |
| SPOP | -1.539983974 | -2.178627992 | **4** |
| DUSP6 | -1.975740988 | -2.127950056 | **4** |
| MRPL18 | -1.869786104 | -2.124781794 | **4** |
| NUPL2 | -1.962922975 | -2.10906348 | **4** |
| NSF | -1.923533118 | -2.108115687 | **4** |
| LOC642197 | -2.014626592 | -2.105445659 | **4** |
| PDCD2 | -1.781007387 | -2.103107478 | **4** |
| SFRS14 | -1.665186584 | -2.087453377 | **4** |
| FNTB | -1.723182043 | -2.087277508 | **4** |
| LOC113386 | -1.888407598 | -2.079497551 | **4** |
| RTCD1 | -1.883014712 | -2.076448015 | **4** |
| CRLS1 | -1.610640459 | -2.067545158 | **4** |
| CNPY4 | -2.017889818 | -2.067323841 | **4** |
| ZNF32 | -1.715295892 | -2.053449475 | **4** |
| TCFL5 | -1.819990228 | -2.040985217 | **4** |
| PPP2R2C | -1.947441382 | -2.040825164 | **4** |
| METTL5 | -1.66926926 | -2.035949948 | **4** |
| LDLR | -1.706048073 | -2.034014987 | **4** |
| GTSF1 | -1.56431998 | -2.009611195 | **4** |
| FAM120B | -1.810717587 | -1.997331887 | **4** |
| CKS2 | -1.689625594 | -1.966964189 | **4** |
| SNCA | -1.815937418 | -1.950458035 | **4** |
| KIAA0859 | -1.521609431 | -1.937042978 | **4** |
| RHBDF2 | -1.626995499 | -1.925609778 | **4** |
| C9orf90 | -1.580625202 | -1.924498578 | **4** |
| TEAD4 | -1.649438884 | -1.924267032 | **4** |
| CKS2 | -1.752030646 | -1.890641321 | **4** |
| LOC389137 | -1.841126767 | -1.889786735 | **4** |
| HDDC3 | -1.841996547 | -1.88367902 | **4** |
| LOC642197 | -1.68257 | -1.86777132 | **4** |
| CBX1 | -1.611642887 | -1.833787301 | **4** |
| MUTYH | -1.634443901 | -1.817678366 | **4** |
| C9orf114 | -1.591890027 | -1.796707488 | **4** |
| DPAGT1 | -1.531700466 | -1.785977867 | **4** |
| RBMS3 | -1.697548063 | -1.784821452 | **4** |
| UBQLN1 | -1.59362376 | -1.759590923 | **4** |
| TSGA14 | -1.684191584 | -1.758490373 | **4** |
| APBA3 | -1.689728495 | -1.745205529 | **4** |
| DIS3L | -1.670787158 | -1.728615379 | **4** |
| BCAP29 | -1.582049374 | -1.715347856 | **4** |
| IDH1 | -1.53771776 | -1.629139335 | **4** |
| BRP44L | -1.585758829 | -1.62571359 | **4** |
| CBL | -1.601347016 | -1.614126879 | **4** |
| UBE2G1 | -1.503108279 | -1.586710323 | **4** |
| LOC642033 | -1.601124434 | 1.683441057 | **6** |
