# Supplementary material for: Minimal Peroxide Exposure of Neuronal Cells Induces Multifaceted Adaptive Responses
Source: PLoS One. 2010 Dec 17;5(12):e14352. doi: 10.1371/journal.pone.0014352 (PMC3003681; doi:10.1371/journal.pone.0014352)
Supplement: Table S17 — Common 8 hour MeCh-regulated gene series. MeCh-regulated genes that were significantly elevated or reduced compared to the respective unstimulated control cells in both the control (untreated: MeCh-8h-Control vs. Control-Control) and CMP state SH-SY5Y cells (MeCh-8h-CMP vs. Control-CMP). The series number refers to the simplistic relationships between the degree of regulation of the respective genes and the cellular state (untreated or CMP). Series 1 (both upregulated) - MeCh-8h-CMP vs. Control-CMP > MeCh-8h-Control vs. Control-Control; Series 2 (both upregulated) - MeCh-8h-Control vs. Control-Control > MeCh-8h-CMP vs. Control-CMP; Series 3 (both downregulated) - MeCh-8h-Control vs. Control-Control > MeCh-8h-CMP vs. Control-CMP; Series 4 (both downregulated) - MeCh-8h-CMP vs. Control-CMP > MeCh-8h-Control vs. Control-Control; Series 5 (downregulated in control, upregulated in CMP); Series 6 (upregulated in control, down regulated in CMP). (0.57 MB DOC) [file pone.0014352.s024.doc]

**Table S17. Common 8 hour MeCh-regulated gene series.** MeCh-regulated genes that were significantly elevated or reduced compared to the respective unstimulated control cells in both the control (untreated: *MeCh-8h-Control vs. Control-Control*) and CMP state SH-SY5Y cells (*MeCh-8h-CMP vs. Control-CMP*). The series number refers to the simplistic relationships between the degree of regulation of the respective genes and the cellular state (untreated or CMP). Series 1 (both upregulated) - *MeCh-8h-CMP vs. Control-CMP* > *MeCh-8h-Control vs. Control-Control*; Series 2 (both upregulated) - *MeCh-8h-Control vs. Control-Control* > *MeCh-8h-CMP vs. Control-CMP*; Series 3 (both downregulated) - *MeCh-8h-Control vs. Control-Control* > *MeCh-8h-CMP vs. Control-CMP*; Series 4 (both downregulated) - *MeCh-8h-CMP vs. Control-CMP* > *MeCh-8h-Control vs. Control-Control*; Series 5 (downregulated in control, upregulated in CMP); Series 6 (upregulated in control, down regulated in CMP).

| **SYMBOL** | **MeCh-2h-CMP vs. Control-CMP** | **MeCh-2h-Control vs. Control-Control** | **Series #** |
| --- | --- | --- | --- |
| IL8 | 10.06318104 | 9.127222671 | **1** |
| AMY1C | 4.602609676 | 2.276431547 | **1** |
| NPEPL1 | 4.598963048 | 3.523835754 | **1** |
| CD44 | 4.487017976 | 3.688572432 | **1** |
| C1orf63 | 4.240542193 | 2.997791584 | **1** |
| HS6ST2 | 4.151649147 | 3.647962462 | **1** |
| ANKRD13D | 3.92214271 | 2.177297845 | **1** |
| CSTF3 | 3.801048872 | 3.451164686 | **1** |
| STK36 | 3.655970187 | 3.156856836 | **1** |
| TTC13 | 3.372815457 | 1.797472719 | **1** |
| NDRG4 | 3.30441631 | 2.882504872 | **1** |
| SNAPC4 | 3.282206617 | 2.310451753 | **1** |
| TNK2 | 3.25130642 | 2.988752139 | **1** |
| C6orf134 | 3.225852235 | 2.757525403 | **1** |
| AGPAT4 | 3.216915953 | 2.68607746 | **1** |
| VCL | 3.202132944 | 2.689387879 | **1** |
| FAT | 3.174380487 | 2.653286393 | **1** |
| ACCN2 | 3.168699224 | 2.580200262 | **1** |
| BTBD11 | 3.158623509 | 2.414940271 | **1** |
| HCN3 | 3.155514953 | 2.793479434 | **1** |
| USP24 | 3.121714608 | 2.132406176 | **1** |
| FOXJ2 | 3.120603058 | 2.140014842 | **1** |
| PLXNA3 | 3.10751249 | 2.919011841 | **1** |
| LOC401720 | 3.101195196 | 2.186136549 | **1** |
| BRWD1 | 3.089550434 | 2.709031509 | **1** |
| MAN2C1 | 3.084143921 | 1.885310286 | **1** |
| CAPRIN1 | 3.049560004 | 2.074606772 | **1** |
| KIAA0194 | 3.024675621 | 2.334986342 | **1** |
| BTAF1 | 2.996336667 | 2.398069893 | **1** |
| PFKFB4 | 2.983573499 | 2.789083334 | **1** |
| ZYX | 2.929818883 | 2.593470412 | **1** |
| CTDSPL | 2.91733368 | 2.46135338 | **1** |
| ARFGEF1 | 2.910934619 | 2.435197918 | **1** |
| GATS | 2.859920657 | 2.368971919 | **1** |
| PDGFRB | 2.857519339 | 2.679608421 | **1** |
| FNBP4 | 2.826306032 | 1.7998931 | **1** |
| DTX2 | 2.815538991 | 1.84155767 | **1** |
| PRKCA | 2.78096471 | 2.753856899 | **1** |
| DFFB | 2.752008078 | 2.593407242 | **1** |
| ABCC5 | 2.727247727 | 2.199894041 | **1** |
| MAST1 | 2.704440824 | 1.902487727 | **1** |
| VAMP1 | 2.650968895 | 2.270427404 | **1** |
| PRO1853 | 2.649399579 | 1.597541896 | **1** |
| INTS1 | 2.646501746 | 2.21045514 | **1** |
| NDST2 | 2.644851345 | 1.693776836 | **1** |
| ATP1B1 | 2.635551619 | 2.375542633 | **1** |
| POLR3A | 2.618180706 | 1.958757989 | **1** |
| LYK5 | 2.59058771 | 1.917441717 | **1** |
| MAP3K4 | 2.543574558 | 2.442798004 | **1** |
| RBM33 | 2.536115313 | 1.979624545 | **1** |
| ZNF789 | 2.482291801 | 1.720308185 | **1** |
| DENND4C | 2.455917128 | 1.74248351 | **1** |
| FRAS1 | 2.444515973 | 1.574727407 | **1** |
| NSUN5 | 2.434768802 | 2.147287277 | **1** |
| IHPK1 | 2.418971119 | 1.94414226 | **1** |
| GART | 2.40092216 | 1.754315175 | **1** |
| SLC9A1 | 2.385811228 | 2.101414376 | **1** |
| RGS12 | 2.374052695 | 2.104740754 | **1** |
| XRN1 | 2.350105437 | 1.660490436 | **1** |
| C8orf33 | 2.344599652 | 2.113015455 | **1** |
| SPG7 | 2.342951805 | 1.913649013 | **1** |
| CHFR | 2.333349984 | 1.808457816 | **1** |
| NDEL1 | 2.324075186 | 2.00580665 | **1** |
| SH3BGRL2 | 2.297828662 | 1.83625929 | **1** |
| PLA2G4B | 2.275378512 | 1.525826665 | **1** |
| DPYSL3 | 2.269794684 | 2.254905302 | **1** |
| CLCN6 | 2.245129762 | 2.0331587 | **1** |
| C15orf17 | 2.243122511 | 2.053596001 | **1** |
| KIAA0363 | 2.229060107 | 1.848375383 | **1** |
| AK3 | 2.208597685 | 1.730987393 | **1** |
| AMFR | 2.207320649 | 2.159165268 | **1** |
| LOC401357 | 2.190688189 | 1.908735679 | **1** |
| CAMSAP1 | 2.179569246 | 2.105056104 | **1** |
| DPYSL4 | 2.175348468 | 1.950458126 | **1** |
| SELO | 2.147088239 | 1.999347862 | **1** |
| LUZP1 | 2.145792455 | 2.086665681 | **1** |
| COX15 | 2.135724091 | 1.875035265 | **1** |
| AXIN1 | 2.134171907 | 2.065251608 | **1** |
| LOC146517 | 2.133107662 | 1.505864255 | **1** |
| ATP6V1B1 | 2.126774908 | 1.822732584 | **1** |
| CCDC136 | 2.11418375 | 1.569205373 | **1** |
| CHKA | 2.113363595 | 2.061777079 | **1** |
| PHLDB1 | 2.103583932 | 1.987897196 | **1** |
| SLC26A6 | 2.09979464 | 1.736702867 | **1** |
| ATP8B2 | 2.097727007 | 1.860579812 | **1** |
| ASXL2 | 2.080458054 | 1.946885144 | **1** |
| ABR | 2.053952986 | 1.899206084 | **1** |
| CNOT1 | 2.022856251 | 1.701970179 | **1** |
| NOMO2 | 2.020479338 | 1.540186938 | **1** |
| WFS1 | 2.011592831 | 1.911370646 | **1** |
| GNS | 2.005910415 | 1.551997059 | **1** |
| EDC4 | 2.005765947 | 1.963451234 | **1** |
| ADCY3 | 2.001665778 | 1.671182888 | **1** |
| ZNF133 | 1.999031016 | 1.860923768 | **1** |
| LANCL2 | 1.970876729 | 1.797037403 | **1** |
| BTN2A1 | 1.963351841 | 1.752773621 | **1** |
| TRO | 1.956571467 | 1.745036494 | **1** |
| WDR90 | 1.953987186 | 1.678615354 | **1** |
| D4S234E | 1.949882381 | 1.583776402 | **1** |
| SLC35E1 | 1.938417062 | 1.720644991 | **1** |
| ZFYVE26 | 1.929557174 | 1.91558266 | **1** |
| PEX11B | 1.928725039 | 1.626619074 | **1** |
| ATP6V0A1 | 1.925472504 | 1.619198678 | **1** |
| C1orf35 | 1.922153317 | 1.891539593 | **1** |
| TMEM185A | 1.920082876 | 1.629304178 | **1** |
| LAMA5 | 1.907621207 | 1.554809526 | **1** |
| SIN3A | 1.889275594 | 1.869618088 | **1** |
| MBD6 | 1.883735542 | 1.778880479 | **1** |
| FAM116B | 1.882541887 | 1.785418081 | **1** |
| EFNB3 | 1.867908056 | 1.653552417 | **1** |
| INPPL1 | 1.85583649 | 1.725680452 | **1** |
| MTAP | 1.850895753 | 1.667356067 | **1** |
| CTPS2 | 1.839181469 | 1.721032727 | **1** |
| SFRS17A | 1.835619996 | 1.56057755 | **1** |
| VASH1 | 1.827546735 | 1.5960662 | **1** |
| NCKIPSD | 1.80936841 | 1.79558824 | **1** |
| CCDC25 | 1.786919154 | 1.699087781 | **1** |
| ZNF275 | 1.767979551 | 1.614207326 | **1** |
| ITSN1 | 1.764747518 | 1.728273649 | **1** |
| TIAM2 | 1.721190626 | 1.535115353 | **1** |
| DHRS1 | 1.718628909 | 1.635860654 | **1** |
| SLC26A11 | 1.705323903 | 1.544984995 | **1** |
| RNF150 | 1.705196374 | 1.572081763 | **1** |
| ARHGAP17 | 1.695709552 | 1.637806958 | **1** |
| U2AF1L2 | 1.689813842 | 1.634113993 | **1** |
| GCH1 | 1.63760209 | 1.571428641 | **1** |
| SPECC1L | 1.623749208 | 1.613882716 | **1** |
| SYNCRIP | 1.599150353 | 1.511464215 | **1** |
| NELL1 | 3.840256565 | 5.106507048 | **2** |
| RHBDD2 | 4.176487578 | 4.919405276 | **2** |
| GFRA3 | 3.874913185 | 4.711756968 | **2** |
| RHBDD2 | 3.586057391 | 4.101666532 | **2** |
| PLXNB1 | 3.510738301 | 3.958317284 | **2** |
| SGSH | 3.130702223 | 3.864792996 | **2** |
| TNC | 3.542507738 | 3.784306435 | **2** |
| LOC339344 | 2.257159912 | 3.54470996 | **2** |
| OCIAD2 | 2.983264985 | 3.534389262 | **2** |
| TRIM46 | 2.599599881 | 3.397829991 | **2** |
| SPOCK1 | 3.351983746 | 3.397194464 | **2** |
| FAM125B | 1.775304346 | 3.328585772 | **2** |
| FAM119B | 2.725393633 | 3.274157276 | **2** |
| GAB2 | 2.219442661 | 3.264883868 | **2** |
| ANKS1A | 2.443977855 | 3.205449142 | **2** |
| ATP9A | 2.694617131 | 3.159493804 | **2** |
| LOC728734 | 2.614817687 | 3.148234031 | **2** |
| BSN | 2.851823293 | 3.114910206 | **2** |
| DGKQ | 2.413173644 | 3.106000258 | **2** |
| JUP | 1.640178969 | 3.087141637 | **2** |
| MYH9 | 2.477254105 | 2.975167862 | **2** |
| MLL4 | 1.565364407 | 2.963291785 | **2** |
| GON4L | 2.851012338 | 2.960864503 | **2** |
| SLC41A1 | 2.137715088 | 2.9347896 | **2** |
| KIAA1545 | 1.514096037 | 2.928884821 | **2** |
| ZCCHC14 | 2.091876825 | 2.888572988 | **2** |
| VCAM1 | 2.726963546 | 2.842944091 | **2** |
| KIAA0649 | 1.667527159 | 2.830460315 | **2** |
| DEDD2 | 1.889263187 | 2.808565177 | **2** |
| NSFL1C | 1.933632124 | 2.805387812 | **2** |
| MAPK8IP3 | 2.108567104 | 2.795942286 | **2** |
| GARNL4 | 1.931634066 | 2.795237584 | **2** |
| KIAA1688 | 2.5705437 | 2.790086509 | **2** |
| PHF13 | 2.046620338 | 2.786484581 | **2** |
| HEATR5B | 1.846460591 | 2.75600532 | **2** |
| POMT1 | 1.896742178 | 2.71971397 | **2** |
| CLIP2 | 1.668918737 | 2.71044648 | **2** |
| PCGF2 | 1.845941206 | 2.708185996 | **2** |
| RABGAP1 | 2.152280773 | 2.700662461 | **2** |
| SLC25A42 | 2.171281616 | 2.699914113 | **2** |
| MGC3020 | 2.109141973 | 2.677178695 | **2** |
| FHOD1 | 1.683735954 | 2.661802124 | **2** |
| INPP5E | 1.766884346 | 2.657482378 | **2** |
| IDS | 2.056861055 | 2.655272072 | **2** |
| EPHB4 | 2.071235744 | 2.649506193 | **2** |
| FBXO31 | 1.58323303 | 2.609649169 | **2** |
| SORT1 | 2.070003855 | 2.605390899 | **2** |
| STK40 | 1.892854786 | 2.592697473 | **2** |
| THOC5 | 1.909257407 | 2.584221622 | **2** |
| HEATR5B | 1.84514108 | 2.570975122 | **2** |
| LOC387856 | 1.843242088 | 2.570803359 | **2** |
| FBXL16 | 1.916044419 | 2.559109533 | **2** |
| ARID3A | 2.388782245 | 2.53599238 | **2** |
| TESK1 | 1.896247137 | 2.486779245 | **2** |
| KIAA1671 | 1.955974702 | 2.476945646 | **2** |
| TUB | 1.559238273 | 2.475643525 | **2** |
| FAM65A | 2.353387324 | 2.456810745 | **2** |
| FAM131A | 1.920623615 | 2.439691529 | **2** |
| MYADM | 2.012708786 | 2.437359642 | **2** |
| LOC728014 | 1.571538836 | 2.436107083 | **2** |
| SBF1 | 2.421506731 | 2.432902686 | **2** |
| GAS8 | 2.156677197 | 2.421280499 | **2** |
| DNAJB1 | 2.341130521 | 2.41549016 | **2** |
| 3-Sep | 2.043405397 | 2.387289687 | **2** |
| MCM3AP | 2.311112482 | 2.366068874 | **2** |
| ARHGEF18 | 1.977294652 | 2.364272986 | **2** |
| RAI1 | 1.821934743 | 2.360623393 | **2** |
| ZNF207 | 1.555665883 | 2.348819276 | **2** |
| BANP | 1.895057931 | 2.33975891 | **2** |
| NPC1 | 1.850381598 | 2.338982586 | **2** |
| PDCD2 | 2.297981303 | 2.323300187 | **2** |
| BEGAIN | 1.930697674 | 2.317475528 | **2** |
| SECISBP2 | 1.739367262 | 2.315201922 | **2** |
| ASCC3L1 | 1.914758615 | 2.312099441 | **2** |
| THBS3 | 1.843262235 | 2.293928895 | **2** |
| ST8SIA2 | 2.186628906 | 2.276721853 | **2** |
| AZI1 | 2.058448725 | 2.272724405 | **2** |
| LOC653103 | 1.962176377 | 2.270031794 | **2** |
| RANBP10 | 1.532434695 | 2.269847399 | **2** |
| CIRBP | 2.224231548 | 2.257336432 | **2** |
| CPXM1 | 1.519147967 | 2.256049862 | **2** |
| LTBP3 | 1.57518307 | 2.249981038 | **2** |
| OSBPL7 | 1.8777133 | 2.246932262 | **2** |
| DBH | 1.729963712 | 2.239493665 | **2** |
| KBTBD11 | 1.923186274 | 2.237561086 | **2** |
| CNTNAP2 | 1.677972606 | 2.234098901 | **2** |
| ARHGAP23 | 1.778117715 | 2.210869631 | **2** |
| XYLT2 | 2.126945514 | 2.207717987 | **2** |
| CD97 | 1.894154062 | 2.186577897 | **2** |
| UBN1 | 1.991792414 | 2.158977066 | **2** |
| POLS | 1.796158766 | 2.124599983 | **2** |
| THOC5 | 1.976018947 | 2.122946085 | **2** |
| FAM62B | 1.605206709 | 2.108391029 | **2** |
| FAM57A | 1.588261134 | 2.099086099 | **2** |
| RASSF7 | 1.86599904 | 2.08438951 | **2** |
| MIZF | 1.685674111 | 2.078551194 | **2** |
| IFFO | 1.981815619 | 2.069914169 | **2** |
| SULT1A3 | 1.727636546 | 2.067441128 | **2** |
| PRSS3 | 2.035104973 | 2.051693425 | **2** |
| PERLD1 | 1.53965918 | 2.041448521 | **2** |
| C2CD2 | 1.70872465 | 2.032724766 | **2** |
| GLTSCR1 | 1.641496567 | 2.027798015 | **2** |
| KLHDC8B | 1.799109277 | 2.010896155 | **2** |
| IGSF9 | 1.740623919 | 2.005751972 | **2** |
| ITM2C | 1.817265628 | 2.000502128 | **2** |
| LASP1 | 1.920121296 | 1.999497795 | **2** |
| GAS1 | 1.67536026 | 1.990121778 | **2** |
| SELI | 1.918752964 | 1.978452403 | **2** |
| CCRK | 1.882487228 | 1.977051832 | **2** |
| TRMT11 | 1.867415122 | 1.94343528 | **2** |
| C4orf30 | 1.917244602 | 1.931936722 | **2** |
| IK | 1.662282362 | 1.907016559 | **2** |
| RIMS3 | 1.787283389 | 1.897927163 | **2** |
| EXOSC9 | 1.704413518 | 1.887804279 | **2** |
| PATL1 | 1.676113734 | 1.882869809 | **2** |
| ZNF207 | 1.874006722 | 1.882128018 | **2** |
| FAM39DP | 1.828905393 | 1.863761191 | **2** |
| MAPT | 1.576115037 | 1.860318675 | **2** |
| UBE2G1 | 1.681664704 | 1.85647163 | **2** |
| DAGLB | 1.557555016 | 1.848662497 | **2** |
| DHX40 | 1.57147005 | 1.848068032 | **2** |
| GTPBP3 | 1.80191298 | 1.846400867 | **2** |
| C8orf33 | 1.605717077 | 1.83122738 | **2** |
| PPP1R13B | 1.734871154 | 1.822765698 | **2** |
| BANP | 1.642754068 | 1.819697601 | **2** |
| RAB11FIP3 | 1.773872758 | 1.801225738 | **2** |
| KLHDC4 | 1.502417145 | 1.794586921 | **2** |
| ANAPC1 | 1.581969534 | 1.769228209 | **2** |
| SULT1A1 | 1.638001766 | 1.755088005 | **2** |
| L2HGDH | 1.657067846 | 1.712345125 | **2** |
| ZNF585A | 1.680388982 | 1.695902648 | **2** |
| KIAA1737 | 1.543498556 | 1.693962636 | **2** |
| PEX5 | 1.583059795 | 1.643709713 | **2** |
| C14orf79 | 1.546679287 | 1.640098733 | **2** |
| SLC15A4 | 1.564879838 | 1.622770398 | **2** |
| PLD5 | 1.517884269 | 1.607197691 | **2** |
| EXOC7 | 1.570578927 | 1.588726519 | **2** |
| BPHL | 1.5816462 | 1.584462158 | **2** |
| ARID3B | 1.527648422 | 1.572638246 | **2** |
| ID2 | -5.879942146 | -5.788984308 | **3** |
| IGFBP5 | -5.713526054 | -4.896290702 | **3** |
| KCTD12 | -5.403311848 | -3.743321082 | **3** |
| ID3 | -4.589445535 | -4.576602798 | **3** |
| RORB | -4.422824266 | -3.473139041 | **3** |
| LOC375295 | -4.093444965 | -3.448888482 | **3** |
| BAMBI | -3.83590307 | -3.311783848 | **3** |
| PIR | -3.76374987 | -1.625659058 | **3** |
| SOX4 | -3.724636679 | -1.917882405 | **3** |
| IGFBP5 | -3.685107185 | -3.054171684 | **3** |
| DCN | -3.591147262 | -3.426845115 | **3** |
| TERF1 | -3.545077649 | -1.949212515 | **3** |
| DUT | -3.472135671 | -2.805501018 | **3** |
| DDR2 | -3.453907531 | -2.016944722 | **3** |
| MSL3L1 | -3.451847396 | -2.082294804 | **3** |
| RABL4 | -3.403492654 | -1.94886679 | **3** |
| KCNMA1 | -3.39510647 | -2.825581281 | **3** |
| C16orf14 | -3.394494664 | -2.33194324 | **3** |
| UBFD1 | -3.368727922 | -2.553181385 | **3** |
| THOC2 | -3.276152109 | -1.811682437 | **3** |
| IPO7 | -3.14723692 | -1.749566301 | **3** |
| LOC402560 | -3.14129231 | -2.13371675 | **3** |
| C2orf34 | -3.092217512 | -2.44837809 | **3** |
| SMYD3 | -3.074294612 | -2.99384976 | **3** |
| CHCHD3 | -3.071361023 | -1.982503967 | **3** |
| MTUS1 | -3.071268587 | -1.83288116 | **3** |
| ICT1 | -3.071245964 | -2.267667895 | **3** |
| RN7SK | -3.062616345 | -2.80179768 | **3** |
| DUSP6 | -3.040486492 | -2.122509072 | **3** |
| LOC644162 | -3.018132415 | -2.484584539 | **3** |
| C1orf86 | -3.013118714 | -2.336283008 | **3** |
| GMDS | -3.010153296 | -2.321224939 | **3** |
| SCN2A | -2.973044882 | -2.15810129 | **3** |
| GINS3 | -2.968812815 | -1.643157134 | **3** |
| NEFL | -2.955609311 | -2.218852825 | **3** |
| USP1 | -2.952201108 | -1.763861312 | **3** |
| PPM1G | -2.904736791 | -2.881593733 | **3** |
| GEM | -2.90028812 | -1.758884694 | **3** |
| CHMP4A | -2.899852855 | -2.000221407 | **3** |
| MRPL33 | -2.877664152 | -2.103851524 | **3** |
| COX17 | -2.850640169 | -2.554289801 | **3** |
| HIST1H4C | -2.834749911 | -2.683184008 | **3** |
| HSP90AA1 | -2.822299278 | -2.492805639 | **3** |
| TSPAN7 | -2.811609567 | -1.605931371 | **3** |
| HEATR2 | -2.800123711 | -2.047105644 | **3** |
| SYNPO2 | -2.799344712 | -2.080474951 | **3** |
| NUDT5 | -2.784268282 | -2.390407908 | **3** |
| CSTF3 | -2.763974964 | -2.230742771 | **3** |
| CTSL2 | -2.763897297 | -2.118092617 | **3** |
| ASCL1 | -2.753322224 | -2.670450845 | **3** |
| LOC723972 | -2.752848528 | -1.948971847 | **3** |
| PROX1 | -2.750073786 | -2.670858527 | **3** |
| C1orf144 | -2.697060649 | -1.912327387 | **3** |
| SULF2 | -2.68338461 | -1.848747133 | **3** |
| CCDC90A | -2.673489704 | -2.05966295 | **3** |
| CMBL | -2.535202504 | -2.268700864 | **3** |
| TRK1 | -2.530860564 | -2.181754741 | **3** |
| TCTEX1D2 | -2.524500471 | -1.534265958 | **3** |
| MYLIP | -2.516201219 | -2.083428408 | **3** |
| EZH2 | -2.480699543 | -2.163412769 | **3** |
| PPP2R2C | -2.479745181 | -1.607760635 | **3** |
| DNAJB6 | -2.472665411 | -1.971287527 | **3** |
| GFRA2 | -2.468300936 | -1.640993824 | **3** |
| LRRC17 | -2.465343958 | -2.34995369 | **3** |
| FSTL5 | -2.464692348 | -2.177679605 | **3** |
| YWHAB | -2.454539097 | -1.85813236 | **3** |
| FSD1 | -2.454239643 | -2.065960751 | **3** |
| APOO | -2.452758822 | -1.987786382 | **3** |
| CCDC28B | -2.4524308 | -1.636030688 | **3** |
| ATPAF1 | -2.451570217 | -2.437535528 | **3** |
| FLRT3 | -2.428183147 | -2.043085691 | **3** |
| GTSF1 | -2.426339182 | -1.865775394 | **3** |
| ADM | -2.412061993 | -2.028197167 | **3** |
| LOC728635 | -2.404011866 | -1.752603438 | **3** |
| STYXL1 | -2.383426992 | -2.193606071 | **3** |
| CYB5A | -2.332053765 | -1.616176615 | **3** |
| PRMT6 | -2.317639709 | -1.615653089 | **3** |
| LRRCC1 | -2.278663007 | -2.07347339 | **3** |
| CENPM | -2.27194087 | -2.230179299 | **3** |
| LZIC | -2.259561496 | -2.031687616 | **3** |
| TOP2A | -2.23517014 | -2.018626119 | **3** |
| DCPS | -2.217819474 | -1.508290398 | **3** |
| BRP44L | -2.19558859 | -1.948491281 | **3** |
| CKLF | -2.174854435 | -1.777356271 | **3** |
| GPSN2 | -2.17229576 | -1.712141624 | **3** |
| HADH | -2.167839425 | -1.736359184 | **3** |
| TSGA14 | -2.117791889 | -1.584472127 | **3** |
| ANAPC11 | -2.108835928 | -1.979626806 | **3** |
| HSPB1 | -2.091854233 | -2.012768184 | **3** |
| CRELD1 | -2.089636997 | -1.693899022 | **3** |
| NUDT3 | -2.050494584 | -1.647934626 | **3** |
| SETMAR | -2.046342935 | -1.741189254 | **3** |
| THEM2 | -1.99962389 | -1.576134903 | **3** |
| C20orf103 | -1.991537006 | -1.702661035 | **3** |
| UNG | -1.991518069 | -1.63931724 | **3** |
| AK1 | -1.986351296 | -1.926940363 | **3** |
| MRPL35 | -1.985715971 | -1.738422413 | **3** |
| BOLA3 | -1.945716581 | -1.59768881 | **3** |
| ALDH6A1 | -1.896510297 | -1.716687757 | **3** |
| TK1 | -1.894162625 | -1.80524921 | **3** |
| TSGA14 | -1.841224041 | -1.508238376 | **3** |
| AP1S2 | -1.810874818 | -1.536069633 | **3** |
| CKLF | -1.807546681 | -1.668464357 | **3** |
| AKR7A2 | -1.802328466 | -1.628559076 | **3** |
| C20orf94 | -1.802277836 | -1.636724921 | **3** |
| PDCL | -1.796417196 | -1.777389351 | **3** |
| CNOT7 | -1.792792497 | -1.65573651 | **3** |
| CYB5A | -1.767407344 | -1.608454182 | **3** |
| BIRC5 | -1.76684212 | -1.594325963 | **3** |
| RBM15 | -1.760560145 | -1.595927488 | **3** |
| HNRPC | -1.756432544 | -1.549331426 | **3** |
| DNCL1 | -1.686158502 | -1.510309798 | **3** |
| PECI | -1.683662311 | -1.518107947 | **3** |
| KDELR2 | -1.673742965 | -1.558079664 | **3** |
| GPHN | -1.665649393 | -1.573675872 | **3** |
| GMPPB | -1.612825637 | -1.601156059 | **3** |
| LOC255130 | -1.573504489 | -1.527834128 | **3** |
| ID2 | -6.510949296 | -6.574339722 | **4** |
| IGFBP3 | -3.914987053 | -3.993051492 | **4** |
| ESRRG | -2.679496307 | -3.912744008 | **4** |
| DPAGT1 | -1.806470402 | -3.822442107 | **4** |
| CAP2 | -3.301268616 | -3.665522843 | **4** |
| LOC644422 | -2.012009593 | -3.554921553 | **4** |
| LRRN3 | -2.660612108 | -3.257703314 | **4** |
| CRYZ | -2.285436328 | -3.138549169 | **4** |
| LRRN3 | -2.853282013 | -3.13668731 | **4** |
| FUZ | -2.05227496 | -3.045022812 | **4** |
| LOC653874 | -1.599732923 | -3.044388808 | **4** |
| OIP5 | -2.547638923 | -2.910969534 | **4** |
| PPP2R3C | -2.449334361 | -2.909673367 | **4** |
| LPAR1 | -2.385388937 | -2.894432778 | **4** |
| AIG1 | -2.702803114 | -2.882845537 | **4** |
| POLA1 | -2.798512734 | -2.848101067 | **4** |
| PIR | -1.956868923 | -2.824275003 | **4** |
| FBXO5 | -2.610359923 | -2.814079964 | **4** |
| SPRY2 | -2.0957756 | -2.799310693 | **4** |
| ADK | -2.16131971 | -2.697845532 | **4** |
| ISOC1 | -2.40120263 | -2.650165724 | **4** |
| PLS3 | -1.926982894 | -2.609211193 | **4** |
| CDKN3 | -2.17441396 | -2.512870254 | **4** |
| AMD1 | -2.266832783 | -2.504469858 | **4** |
| CDT1 | -2.323486387 | -2.503232684 | **4** |
| LOC653884 | -2.174303477 | -2.503223837 | **4** |
| C7orf24 | -2.071798691 | -2.431540727 | **4** |
| RBMS3 | -2.230552729 | -2.391778815 | **4** |
| HIBCH | -1.699333304 | -2.383807394 | **4** |
| SSBP2 | -1.908559701 | -2.379798217 | **4** |
| PMAIP1 | -2.098247499 | -2.362289476 | **4** |
| HSD17B4 | -1.642740944 | -2.357103264 | **4** |
| HSPE1 | -1.885783258 | -2.326631105 | **4** |
| UBE2C | -1.963482659 | -2.319729788 | **4** |
| SIRT5 | -2.025390649 | -2.315490761 | **4** |
| LOC727761 | -1.933460404 | -2.306962528 | **4** |
| C14orf124 | -1.594868282 | -2.304727187 | **4** |
| ATM | -1.983822072 | -2.296716708 | **4** |
| HSPE1 | -1.638629998 | -2.275675515 | **4** |
| RGS4 | -2.038309115 | -2.272749124 | **4** |
| SFRS14 | -1.647814834 | -2.245199835 | **4** |
| C14orf102 | -1.745164739 | -2.244430426 | **4** |
| FKBP4 | -2.189431024 | -2.241475624 | **4** |
| PTPLA | -2.15679184 | -2.238765318 | **4** |
| RSRC1 | -2.166230141 | -2.236725394 | **4** |
| FEZ1 | -1.871749735 | -2.235988046 | **4** |
| UBE2C | -1.798058218 | -2.22497264 | **4** |
| DTL | -1.638169078 | -2.21757242 | **4** |
| PTEN | -1.746717976 | -2.209997159 | **4** |
| DPAGT1 | -1.798968732 | -2.202139021 | **4** |
| UBL7 | -1.900130651 | -2.201202408 | **4** |
| LOC653080 | -1.537820366 | -2.197997262 | **4** |
| C6orf125 | -2.074391074 | -2.187749252 | **4** |
| PDCD2 | -2.03259103 | -2.184789786 | **4** |
| SDHC | -1.70550032 | -2.179454055 | **4** |
| H1F0 | -2.044986857 | -2.13266399 | **4** |
| MRPL24 | -1.805247301 | -2.09981774 | **4** |
| PIGP | -2.0844859 | -2.090223651 | **4** |
| ING3 | -1.950649572 | -2.076459966 | **4** |
| LOC643995 | -1.821764681 | -2.060522209 | **4** |
| PHYHIPL | -1.921787025 | -2.048561998 | **4** |
| BCL11A | -1.953992068 | -2.046071226 | **4** |
| LOC387882 | -2.030731156 | -2.042870877 | **4** |
| PRR16 | -1.808112508 | -2.03763498 | **4** |
| HERC5 | -1.608533914 | -2.010277954 | **4** |
| PHYH | -1.695580806 | -1.953530024 | **4** |
| CDKN1A | -1.647380645 | -1.88303619 | **4** |
| UBL5 | -1.755830157 | -1.878172937 | **4** |
| ZWILCH | -1.771984654 | -1.875273656 | **4** |
| CBX1 | -1.670458343 | -1.848044718 | **4** |
| BCAP29 | -1.767727859 | -1.820127076 | **4** |
| ADA | -1.667643765 | -1.813233632 | **4** |
| UBE2T | -1.599922205 | -1.783233385 | **4** |
| NSMCE1 | -1.727596325 | -1.773338441 | **4** |
| PAICS | -1.701186429 | -1.702774293 | **4** |
| YRDC | -1.538433916 | -1.693696786 | **4** |
| MEIS2 | -1.625389539 | -1.661573528 | **4** |
| TYMS | -1.516889367 | -1.645042134 | **4** |
| FOXRED2 | -1.621009159 | -1.634100519 | **4** |
| SLC44A1 | -1.601700701 | -1.617183938 | **4** |
| NCAM1 | -1.566809498 | -1.588196973 | **4** |
