# Supplementary material for: Minimal Peroxide Exposure of Neuronal Cells Induces Multifaceted Adaptive Responses
Source: PLoS One. 2010 Dec 17;5(12):e14352. doi: 10.1371/journal.pone.0014352 (PMC3003681; doi:10.1371/journal.pone.0014352)
Supplement: Table S18 — Common 2 hour BDNF-regulated gene series. BDNF-regulated genes that were significantly elevated or reduced compared to the respective unstimulated control cells in both the control (untreated: BDNF-2h-Control vs. Control-Control) and CMP state SH-SY5Y cells (BDNF-2h-CMP vs. Control-CMP). The series number refers to the simplistic relationships between the degree of regulation of the respective genes and the cellular state (untreated or CMP). Series 1 (both upregulated) - BDNF-2h-CMP vs. Control-CMP > BDNF-2h-Control vs. Control-Control; Series 2 (both upregulated) - BDNF-2h-Control vs. Control-Control > BDNF-2h-CMP vs. Control-CMP; Series 3 (both downregulated) - BDNF-2h-Control vs. Control-Control > BDNF-2h-CMP vs. Control-CMP; Series 4 (both downregulated) - BDNF-2h-CMP vs. Control-CMP > BDNF-2h-Control vs. Control-Control; Series 5 (downregulated in control, upregulated in CMP); Series 6 (upregulated in control, down regulated in CMP). (0.46 MB DOC) [file pone.0014352.s025.doc]

**Table S18. Common 2 hour BDNF-regulated gene series.** BDNF-regulated genes that were significantly elevated or reduced compared to the respective unstimulated control cells in both the control (untreated: *BDNF-2h-Control vs. Control-Control*) and CMP state SH-SY5Y cells (*BDNF-2h-CMP vs. Control-CMP*). The series number refers to the simplistic relationships between the degree of regulation of the respective genes and the cellular state (untreated or CMP). Series 1 (both upregulated) - *BDNF-2h-CMP vs. Control-CMP* > *BDNF-2h-Control vs. Control-Control*; Series 2 (both upregulated) - *BDNF-2h-Control vs. Control-Control* > *BDNF-2h-CMP vs. Control-CMP*; Series 3 (both downregulated) - *BDNF-2h-Control vs. Control-Control* > *BDNF-2h-CMP vs. Control-CMP*; Series 4 (both downregulated) - *BDNF-2h-CMP vs. Control-CMP* > *BDNF-2h-Control vs. Control-Control*; Series 5 (downregulated in control, upregulated in CMP); Series 6 (upregulated in control, down regulated in CMP).

| **Gene Symbol** | **BDNF-2h-CMP vs. Control-CMP** | **BDNF-2h-Control vs. Control-Control** | **Series #** |
| --- | --- | --- | --- |
| LOC285176 | 3.790812306 | 3.273516604 | **1** |
| KLF6 | 3.594851225 | 3.457862021 | **1** |
| LOC653994 | 3.584118898 | 2.694342928 | **1** |
| KIAA0644 | 3.557121426 | 3.094026501 | **1** |
| SP2 | 3.517957622 | 2.414832163 | **1** |
| LOC387763 | 3.478364665 | 3.216606118 | **1** |
| P704P | 3.188628161 | 3.081024579 | **1** |
| JUN | 3.129176837 | 2.855713274 | **1** |
| SFRS5 | 3.107548924 | 1.537980024 | **1** |
| LOC402221 | 3.038460093 | 2.886909437 | **1** |
| RHBDD2 | 3.036152732 | 2.306470297 | **1** |
| SFRS6 | 3.034500044 | 1.903886458 | **1** |
| USP24 | 3.021430086 | 1.805920923 | **1** |
| LANCL2 | 2.970237529 | 2.439109298 | **1** |
| KLF11 | 2.937121178 | 2.570960831 | **1** |
| FKSG30 | 2.928795726 | 2.020505931 | **1** |
| ARRDC3 | 2.909818 | 2.326182401 | **1** |
| RAD23B | 2.838224644 | 2.540504388 | **1** |
| SFRS5 | 2.814727775 | 1.876814099 | **1** |
| SNORA70 | 2.73391049 | 1.877284658 | **1** |
| TNFRSF19 | 2.723410145 | 2.177595365 | **1** |
| BTBD11 | 2.702492539 | 1.665195381 | **1** |
| FEM1A | 2.475204042 | 1.770337774 | **1** |
| LOC651149 | 2.473202648 | 1.821526017 | **1** |
| C6orf134 | 2.400937248 | 1.627422067 | **1** |
| LOC653103 | 2.382892579 | 2.1834555 | **1** |
| ITGB5 | 2.37953385 | 2.008351703 | **1** |
| RNF150 | 2.372831837 | 2.111637073 | **1** |
| DDX42 | 2.352962886 | 1.701572777 | **1** |
| RBM33 | 2.33301742 | 1.959549007 | **1** |
| ATP1B1 | 2.207422351 | 1.864050351 | **1** |
| ADAMTSL2 | 2.133199446 | 1.95499374 | **1** |
| CAMSAP1 | 2.128430014 | 1.890729275 | **1** |
| SPG7 | 2.107163199 | 1.652429969 | **1** |
| ATP13A1 | 2.055470083 | 1.639260767 | **1** |
| SLC2A1 | 2.038172473 | 1.663443326 | **1** |
| LOC389517 | 1.997830741 | 1.951942356 | **1** |
| LOC727935 | 1.995900304 | 1.782830907 | **1** |
| CLCN7 | 1.976029598 | 1.710102335 | **1** |
| NASP | 1.95915905 | 1.745744635 | **1** |
| ZNF317 | 1.950470642 | 1.676629447 | **1** |
| SLC7A5 | 1.919196088 | 1.870024652 | **1** |
| SLC11A2 | 1.906033839 | 1.876495826 | **1** |
| SLC7A1 | 1.887912801 | 1.574948248 | **1** |
| ZNF275 | 1.886655532 | 1.846783606 | **1** |
| POFUT1 | 1.815894435 | 1.64243379 | **1** |
| TACC2 | 1.800953547 | 1.680564627 | **1** |
| SYT11 | 1.75634278 | 1.663032545 | **1** |
| CTBP1 | 1.667294351 | 1.625952365 | **1** |
| UHRF1 | 1.631566555 | 1.594949431 | **1** |
| GAS1 | 1.620916049 | 1.56697825 | **1** |
| EGR1 | 12.35771719 | 16.31681529 | **2** |
| H3F3B | 4.212161357 | 5.272416119 | **2** |
| LOC388122 | 2.219383624 | 4.811793845 | **2** |
| RPLP1 | 2.017398851 | 4.749309092 | **2** |
| LOC644330 | 1.64009833 | 4.566848752 | **2** |
| NFKBIZ | 3.127831356 | 4.345858702 | **2** |
| KLF6 | 4.090522716 | 4.123286424 | **2** |
| TNFSF14 | 1.96077951 | 3.634557863 | **2** |
| DUSP1 | 3.37596396 | 3.614867357 | **2** |
| MGEA5 | 3.423645271 | 3.575286843 | **2** |
| CLTC | 1.702101697 | 3.564074834 | **2** |
| LOC653086 | 1.845151768 | 3.512094876 | **2** |
| DOPEY2 | 1.730136642 | 3.511805815 | **2** |
| CSTF3 | 3.414218296 | 3.470093185 | **2** |
| CSF2RA | 1.514759835 | 3.433748464 | **2** |
| ALS2CR14 | 1.82799185 | 3.395191469 | **2** |
| AZIN1 | 1.626559294 | 3.358387112 | **2** |
| INTS1 | 2.524728056 | 3.327877874 | **2** |
| LOC653489 | 1.519145768 | 3.250206071 | **2** |
| PLXNB1 | 2.596528261 | 3.238504413 | **2** |
| LOC649679 | 1.605079537 | 3.228840008 | **2** |
| TUBA3D | 1.91925753 | 3.181800621 | **2** |
| RABL2A | 2.217720716 | 3.181217856 | **2** |
| P2RY11 | 2.909171108 | 3.09191086 | **2** |
| AHR | 2.094510702 | 3.055593653 | **2** |
| TTC32 | 1.927474575 | 3.026446265 | **2** |
| LOC643668 | 2.047237931 | 2.946128412 | **2** |
| LOC653232 | 2.276210165 | 2.902894064 | **2** |
| IL18BP | 1.684345681 | 2.854311793 | **2** |
| BAT3 | 2.073924716 | 2.853899294 | **2** |
| BOLA2 | 2.038548044 | 2.831263886 | **2** |
| AIRE | 2.227707543 | 2.770748282 | **2** |
| ANKRD30B | 2.017462806 | 2.753558222 | **2** |
| PNPT1 | 1.795388777 | 2.749986878 | **2** |
| LOC645018 | 1.797120984 | 2.714309811 | **2** |
| FKTN | 2.008091372 | 2.707923931 | **2** |
| NOL5A | 1.66858481 | 2.649568894 | **2** |
| NGFRAP1 | 1.893782374 | 2.633886923 | **2** |
| CDAN1 | 2.293651452 | 2.611241086 | **2** |
| LOC153561 | 2.259829731 | 2.608065543 | **2** |
| PERLD1 | 1.820293735 | 2.597832525 | **2** |
| C14orf102 | 2.29300481 | 2.564040743 | **2** |
| SUV420H1 | 2.366563082 | 2.545645507 | **2** |
| ARFGAP2 | 2.108460696 | 2.513504592 | **2** |
| IL18 | 1.590669024 | 2.470315982 | **2** |
| SLC44A4 | 1.970505385 | 2.459119415 | **2** |
| KIAA1545 | 1.530148317 | 2.453977195 | **2** |
| GAB2 | 1.56311924 | 2.431096864 | **2** |
| PTBP2 | 1.561466737 | 2.416906373 | **2** |
| FLJ22795 | 1.803462393 | 2.412784764 | **2** |
| HCFC1 | 2.261423291 | 2.404223364 | **2** |
| ACCN2 | 2.087131935 | 2.399840621 | **2** |
| PPP1R15A | 2.302403131 | 2.376387022 | **2** |
| SYNCRIP | 1.871535859 | 2.364597937 | **2** |
| SACS | 1.566210528 | 2.350964399 | **2** |
| KCNT1 | 1.75783639 | 2.31973731 | **2** |
| AMH | 1.697239165 | 2.313456477 | **2** |
| LOC643224 | 1.988825512 | 2.301793778 | **2** |
| SGK | 1.724217325 | 2.29774011 | **2** |
| LRP5L | 1.856186125 | 2.288910489 | **2** |
| SDHALP1 | 1.751905899 | 2.285970722 | **2** |
| LOC642502 | 1.908194117 | 2.285084344 | **2** |
| CX3CL1 | 2.127648293 | 2.257367724 | **2** |
| ADAR | 1.542171515 | 2.248717179 | **2** |
| LOC401357 | 2.19547483 | 2.215136588 | **2** |
| ROR2 | 1.506928489 | 2.168917805 | **2** |
| ABL1 | 2.028250308 | 2.105784639 | **2** |
| ADAM17 | 1.683717469 | 2.087524063 | **2** |
| ZNF16 | 1.991018193 | 2.06514087 | **2** |
| KIAA0194 | 1.692706462 | 2.047047521 | **2** |
| SNAPC4 | 1.960621302 | 2.024612057 | **2** |
| DGCR8 | 1.952252037 | 2.010859705 | **2** |
| CDK5RAP2 | 1.600183099 | 1.958114335 | **2** |
| PTBP2 | 1.581046059 | 1.950872979 | **2** |
| GRAMD4 | 1.690896511 | 1.900993329 | **2** |
| NKTR | 1.598744715 | 1.856232634 | **2** |
| REXO1 | 1.541893107 | 1.844899559 | **2** |
| RIMS3 | 1.63648659 | 1.821510504 | **2** |
| PIGW | 1.759225381 | 1.810592864 | **2** |
| IRS2 | 1.719562362 | 1.785819759 | **2** |
| SMARCD1 | 1.534423602 | 1.721094118 | **2** |
| CPSF1 | 1.667479268 | 1.716730384 | **2** |
| CLCN6 | 1.54907515 | 1.673660611 | **2** |
| CHRNA3 | 1.51482429 | 1.672055003 | **2** |
| RNF38 | 1.559541944 | 1.657354022 | **2** |
| ULK1 | 1.540804016 | 1.625750244 | **2** |
| MAP6 | 1.540107078 | 1.567439814 | **2** |
| ARID4B | -6.25184049 | -3.097905518 | **3** |
| NBPF20 | -5.478570573 | -1.75877595 | **3** |
| ZNF234 | -5.435142606 | -2.981351626 | **3** |
| ARID4B | -5.241110981 | -2.157507484 | **3** |
| RAD21 | -5.152560323 | -2.125303035 | **3** |
| GMCL1 | -5.142109564 | -2.23539012 | **3** |
| UGCG | -4.966167812 | -1.524405505 | **3** |
| HOXC4 | -4.891363415 | -2.092318487 | **3** |
| KRCC1 | -4.777684827 | -3.066214019 | **3** |
| EZH2 | -4.736496348 | -3.106788961 | **3** |
| LOC440160 | -4.730850271 | -1.819574727 | **3** |
| HSPA1A | -4.665849309 | -4.035602144 | **3** |
| MSL3L1 | -4.642516258 | -2.367278299 | **3** |
| SCN2A | -4.601666215 | -2.145299206 | **3** |
| RBM39 | -4.505971765 | -1.66333347 | **3** |
| TRK1 | -4.423541897 | -4.397544896 | **3** |
| GMCL1 | -4.377665756 | -1.736197093 | **3** |
| INTS6 | -4.126091564 | -3.742548993 | **3** |
| LOC644162 | -3.950803004 | -1.579429551 | **3** |
| CDC2L2 | -3.929949945 | -2.047513641 | **3** |
| LOC441763 | -3.893759549 | -2.860961771 | **3** |
| COL4A3BP | -3.85000714 | -1.802052152 | **3** |
| RN7SK | -3.801299245 | -3.412622594 | **3** |
| USP1 | -3.744280921 | -1.605457244 | **3** |
| TRIM4 | -3.616567327 | -3.43613606 | **3** |
| GAS2L3 | -3.587073978 | -2.130504225 | **3** |
| RBMS1 | -3.510541662 | -1.655911391 | **3** |
| HIST1H4C | -3.487189802 | -2.862007341 | **3** |
| MTUS1 | -3.352018022 | -2.772044457 | **3** |
| LOC642477 | -3.319516941 | -1.524816192 | **3** |
| MGC72104 | -3.31588904 | -1.97891165 | **3** |
| C8orf70 | -3.18686792 | -1.975168488 | **3** |
| LOC653820 | -3.155285817 | -2.832977661 | **3** |
| C12orf11 | -3.142919006 | -2.760819323 | **3** |
| PAPD5 | -3.136354184 | -1.535932345 | **3** |
| TROVE2 | -3.133387417 | -1.749573482 | **3** |
| AMD1 | -3.128410654 | -2.997509226 | **3** |
| TIMM9 | -3.069723407 | -1.978692442 | **3** |
| TICAM1 | -3.026605171 | -2.364318707 | **3** |
| SAR1B | -3.003602016 | -2.343950432 | **3** |
| LOC402560 | -2.95474199 | -1.721089118 | **3** |
| PANK1 | -2.907401274 | -2.657105711 | **3** |
| HEY1 | -2.9032777 | -2.707152347 | **3** |
| GUCY1A3 | -2.862687763 | -2.307844053 | **3** |
| CKS2 | -2.85667935 | -2.295644764 | **3** |
| NSL1 | -2.843436687 | -1.876247219 | **3** |
| RNF14 | -2.84332927 | -2.2923855 | **3** |
| THAP10 | -2.806779867 | -2.800226084 | **3** |
| TRIM13 | -2.788937931 | -2.156414143 | **3** |
| DTWD1 | -2.777574525 | -1.87233747 | **3** |
| RBPJ | -2.761316722 | -1.555723552 | **3** |
| PLEKHB2 | -2.755270756 | -1.832038086 | **3** |
| BFAR | -2.751538044 | -1.856145611 | **3** |
| ZNF260 | -2.737557323 | -2.15563171 | **3** |
| ZNF573 | -2.716747687 | -2.133810013 | **3** |
| BTBD10 | -2.711276047 | -2.070237443 | **3** |
| BANP | -2.672321531 | -1.513669956 | **3** |
| IL10RB | -2.662118542 | -2.237131156 | **3** |
| C11orf54 | -2.647383046 | -2.1428618 | **3** |
| ZNRD1 | -2.645874061 | -2.424034239 | **3** |
| EXOSC3 | -2.605028535 | -2.298632254 | **3** |
| MFAP1 | -2.600198908 | -2.49288654 | **3** |
| HRSP12 | -2.588129575 | -2.351244186 | **3** |
| CKS2 | -2.575060996 | -2.569729174 | **3** |
| KIAA0895 | -2.529355794 | -2.338437917 | **3** |
| DBT | -2.520573437 | -1.903917772 | **3** |
| MORC2 | -2.484792235 | -2.323448281 | **3** |
| TOB1 | -2.484624039 | -1.847149891 | **3** |
| LYAR | -2.465863135 | -1.919331027 | **3** |
| PLCXD1 | -2.461117128 | -2.441364978 | **3** |
| THEM2 | -2.446046404 | -2.391555126 | **3** |
| CNIH4 | -2.440834848 | -1.737727725 | **3** |
| ZNF22 | -2.438484652 | -2.009344462 | **3** |
| C3orf31 | -2.436008514 | -2.401907893 | **3** |
| STK3 | -2.428798996 | -2.101643172 | **3** |
| SIX3 | -2.427969795 | -1.654844221 | **3** |
| ANKRD46 | -2.383008052 | -2.053535319 | **3** |
| PLEKHA1 | -2.373112843 | -1.986604075 | **3** |
| ZFYVE21 | -2.371764714 | -2.114623708 | **3** |
| MRPL35 | -2.361490521 | -1.938168603 | **3** |
| FKBP14 | -2.339730634 | -1.664242992 | **3** |
| UPF3B | -2.335585757 | -1.901484472 | **3** |
| REXO2 | -2.322524497 | -1.879229532 | **3** |
| SC4MOL | -2.316516525 | -1.91094186 | **3** |
| C8orf41 | -2.307967817 | -1.982627524 | **3** |
| C12orf29 | -2.28394885 | -1.615169806 | **3** |
| PIAS1 | -2.279256477 | -1.514888671 | **3** |
| UBFD1 | -2.267230696 | -1.83498508 | **3** |
| TMEM5 | -2.25627933 | -1.854171045 | **3** |
| MUTED | -2.247568771 | -1.96008547 | **3** |
| C1orf131 | -2.243171432 | -2.20639602 | **3** |
| TRIM32 | -2.229341181 | -1.936694818 | **3** |
| GTF2E1 | -2.189272377 | -2.067660188 | **3** |
| VPS37A | -2.1847224 | -2.157685039 | **3** |
| SLC35A1 | -2.122916093 | -1.779464254 | **3** |
| CHIC2 | -2.097933881 | -2.059505487 | **3** |
| KLHL9 | -2.060549002 | -1.503901339 | **3** |
| MEX3D | -2.057051974 | -1.783582294 | **3** |
| C2orf44 | -2.037028115 | -1.836023424 | **3** |
| NSUN4 | -2.036603735 | -1.90944254 | **3** |
| MRPL39 | -2.035900252 | -2.023218819 | **3** |
| RALA | -2.033188169 | -2.02536762 | **3** |
| GALNT11 | -2.016523265 | -1.548663306 | **3** |
| FXR1 | -2.005957634 | -1.544062893 | **3** |
| CDC26 | -1.953406852 | -1.567750119 | **3** |
| POLB | -1.94728416 | -1.680312738 | **3** |
| NUPL2 | -1.941015648 | -1.918562709 | **3** |
| BET1 | -1.93554658 | -1.612289975 | **3** |
| PREB | -1.925235254 | -1.64232909 | **3** |
| BRI3BP | -1.916866645 | -1.602300582 | **3** |
| ANAPC10 | -1.892181853 | -1.79743011 | **3** |
| C1orf63 | -1.83897348 | -1.795429262 | **3** |
| PRUNE | -1.808796408 | -1.554033805 | **3** |
| TINP1 | -1.794528422 | -1.558633288 | **3** |
| LMAN2L | -1.788302995 | -1.578382604 | **3** |
| KIAA1737 | -1.779601911 | -1.589299387 | **3** |
| PDCD7 | -1.763768027 | -1.510691871 | **3** |
| DPY30 | -1.76161873 | -1.621981645 | **3** |
| NUMB | -1.756932463 | -1.540452719 | **3** |
| CEPT1 | -1.736372382 | -1.665390401 | **3** |
| NPEPPS | -1.72264929 | -1.571988965 | **3** |
| LYPLAL1 | -1.707840123 | -1.530718377 | **3** |
| ELOVL6 | -1.701123655 | -1.559757167 | **3** |
| TMEM134 | -1.693670928 | -1.575476669 | **3** |
| TERF2 | -1.657964204 | -1.559366508 | **3** |
| RASSF7 | -1.657814936 | -1.582610312 | **3** |
| ARIH1 | -1.646651867 | -1.503654569 | **3** |
| FZD2 | -1.637840849 | -1.510547729 | **3** |
| CCDC90A | -1.618955057 | -1.533238325 | **3** |
| ZFYVE19 | -1.583865331 | -1.515909851 | **3** |
| LOC642197 | -1.560717212 | -1.550315717 | **3** |
| HIBCH | -1.543536099 | -1.514816469 | **3** |
| HSPA1B | -5.991961764 | -6.394144766 | **4** |
| EXOSC3 | -2.990064212 | -4.399963617 | **4** |
| KCNJ8 | -3.219637206 | -4.38703632 | **4** |
| RAB23 | -3.280100648 | -4.085568519 | **4** |
| ZMYM6 | -3.590742875 | -3.816159039 | **4** |
| DNAJB1 | -3.078844566 | -3.778991758 | **4** |
| GEM | -3.403647832 | -3.478238027 | **4** |
| GEM | -2.057924618 | -3.459420288 | **4** |
| ASCL1 | -2.486290144 | -3.412848036 | **4** |
| MTERFD1 | -3.231811821 | -3.346695668 | **4** |
| LYAR | -2.40159938 | -3.333335346 | **4** |
| NUP37 | -2.507850909 | -3.129126328 | **4** |
| ZNRD1 | -2.667960889 | -3.078717054 | **4** |
| ZNF330 | -2.164998912 | -3.071443618 | **4** |
| STAMBPL1 | -2.379454663 | -2.965172217 | **4** |
| MTERFD1 | -2.224811535 | -2.964452217 | **4** |
| DTX2 | -2.746328141 | -2.941405439 | **4** |
| ARMC7 | -2.057343756 | -2.881278908 | **4** |
| CCDC117 | -2.536911285 | -2.863906363 | **4** |
| NDEL1 | -1.982002605 | -2.853704682 | **4** |
| SLC35B3 | -1.857427275 | -2.804069561 | **4** |
| BANP | -1.834096868 | -2.803082543 | **4** |
| SPOP | -2.338777523 | -2.729388978 | **4** |
| BCDIN3D | -1.968949366 | -2.699926867 | **4** |
| ZCCHC9 | -2.195837055 | -2.699717112 | **4** |
| HIST1H2AC | -1.52610562 | -2.681401715 | **4** |
| DDIT4 | -1.713936934 | -2.679580794 | **4** |
| MGC12966 | -1.84344489 | -2.662835594 | **4** |
| LOC339344 | -2.503260927 | -2.62562177 | **4** |
| FBXO22 | -2.427805998 | -2.597803185 | **4** |
| MITD1 | -2.160295252 | -2.578275768 | **4** |
| PROSC | -2.404206364 | -2.557678816 | **4** |
| LARP6 | -2.377986726 | -2.542717263 | **4** |
| HOXC6 | -2.079837376 | -2.539592408 | **4** |
| C16orf72 | -2.128107967 | -2.534621505 | **4** |
| FIP1L1 | -1.764442995 | -2.481960384 | **4** |
| MRPL18 | -1.586228989 | -2.446098586 | **4** |
| DUSP4 | -1.668407384 | -2.44599315 | **4** |
| APIP | -1.801911672 | -2.431419504 | **4** |
| CENPA | -2.029928387 | -2.429549388 | **4** |
| SGOL1 | -1.638257227 | -2.427830052 | **4** |
| AURKA | -1.68346825 | -2.396488673 | **4** |
| ZNRD1 | -2.170146488 | -2.390399693 | **4** |
| OPN3 | -2.000723351 | -2.389390023 | **4** |
| CCDC76 | -1.507989467 | -2.386580766 | **4** |
| PUS1 | -2.25369057 | -2.345570421 | **4** |
| RTCD1 | -2.067763235 | -2.340453488 | **4** |
| C1orf25 | -2.043556084 | -2.337500579 | **4** |
| GART | -1.691783363 | -2.323389897 | **4** |
| C6orf166 | -1.779609113 | -2.319708711 | **4** |
| CNOT4 | -1.591606716 | -2.22186383 | **4** |
| GTF2E2 | -1.934300202 | -2.205450742 | **4** |
| ZNF594 | -1.603078501 | -2.202036278 | **4** |
| WDR33 | -2.177261397 | -2.198887145 | **4** |
| NFKB1 | -1.730508679 | -2.193257915 | **4** |
| KLHL7 | -2.101830457 | -2.184748148 | **4** |
| TMEM22 | -2.054911151 | -2.175541002 | **4** |
| C18orf54 | -2.06877328 | -2.166618333 | **4** |
| TMEM126B | -1.791190281 | -2.164295148 | **4** |
| RIOK2 | -1.786198155 | -2.154383282 | **4** |
| NPFFR2 | -1.625633574 | -2.128462106 | **4** |
| SIRT5 | -1.930036615 | -2.119257736 | **4** |
| DNCL1 | -1.848242679 | -2.030764064 | **4** |
| ANKRD54 | -1.96839977 | -2.021983173 | **4** |
| BOLA3 | -1.800521148 | -2.017209116 | **4** |
| ZFP3 | -1.528711889 | -1.996779894 | **4** |
| MRPS31 | -1.716767545 | -1.995551092 | **4** |
| RABL4 | -1.83343574 | -1.987025558 | **4** |
| HERC4 | -1.824321773 | -1.975453536 | **4** |
| KIAA0859 | -1.732546237 | -1.972933805 | **4** |
| ZNF189 | -1.889400357 | -1.972108852 | **4** |
| HIST1H2BD | -1.884293378 | -1.968820247 | **4** |
| GEMIN6 | -1.596036218 | -1.962088287 | **4** |
| HSPA8 | -1.875055009 | -1.947275977 | **4** |
| C11orf60 | -1.558900921 | -1.937945818 | **4** |
| RNF149 | -1.666605561 | -1.930260759 | **4** |
| ORC5L | -1.507290823 | -1.921409138 | **4** |
| TIA1 | -1.788777961 | -1.907243922 | **4** |
| BOLA3 | -1.599565012 | -1.905321601 | **4** |
| STK4 | -1.822915598 | -1.900799119 | **4** |
| ZAK | -1.550046003 | -1.899855416 | **4** |
| RNF219 | -1.546563789 | -1.89871149 | **4** |
| CSNK2A2 | -1.66930032 | -1.885401479 | **4** |
| TGDS | -1.620638487 | -1.860759106 | **4** |
| PPP2CB | -1.512389564 | -1.853120652 | **4** |
| FANCL | -1.514845365 | -1.826657132 | **4** |
| TMEM99 | -1.71023285 | -1.789093022 | **4** |
| C9orf23 | -1.745573562 | -1.779077574 | **4** |
| PHYHIPL | -1.663086467 | -1.738105444 | **4** |
| TAF12 | -1.500794366 | -1.73678846 | **4** |
| CEP55 | -1.619185575 | -1.70358076 | **4** |
| TUT1 | -1.609697246 | -1.702308012 | **4** |
| TPST2 | -1.52764716 | -1.68103525 | **4** |
| C21orf51 | -1.587996887 | -1.675295755 | **4** |
| NEFL | -1.574546583 | -1.667841397 | **4** |
| RABEPK | -1.59149614 | -1.602323851 | **4** |
| MYOZ3 | -1.589181168 | -1.591967491 | **4** |
| RBM42 | -1.512161125 | -1.570923413 | **4** |
| POLE2 | -1.513321349 | -1.51369411 | **4** |
