# Supplementary material for: Minimal Peroxide Exposure of Neuronal Cells Induces Multifaceted Adaptive Responses
Source: PLoS One. 2010 Dec 17;5(12):e14352. doi: 10.1371/journal.pone.0014352 (PMC3003681; doi:10.1371/journal.pone.0014352)
Supplement: Table S19 — Common 4 hour BDNF-regulated gene series. BDNF-regulated genes that were significantly elevated or reduced compared to the respective unstimulated control cells in both the control (untreated: BDNF-4h-Control vs. Control-Control) and CMP state SH-SY5Y cells (BDNF-4h-CMP vs. Control-CMP). The series number refers to the simplistic relationships between the degree of regulation of the respective genes and the cellular state (untreated or CMP). Series 1 (both upregulated) - BDNF-4h-CMP vs. Control-CMP > BDNF-4h-Control vs. Control-Control; Series 2 (both upregulated) - BDNF-4h-Control vs. Control-Control > BDNF-4h-CMP vs. Control-CMP; Series 3 (both downregulated) - BDNF-4h-Control vs. Control-Control > BDNF-4h-CMP vs. Control-CMP; Series 4 (both downregulated) - BDNF-4h-CMP vs. Control-CMP > BDNF-4h-Control vs. Control-Control; Series 5 (downregulated in control, upregulated in CMP); Series 6 (upregulated in control, down regulated in CMP). (0.46 MB DOC) [file pone.0014352.s026.doc]

**Table S19 Common 4 hour BDNF-regulated gene series.** BDNF-regulated genes that were significantly elevated or reduced compared to the respective unstimulated control cells in both the control (untreated: *BDNF-4h-Control vs. Control-Control*) and CMP state SH-SY5Y cells (*BDNF-4h-CMP vs. Control-CMP*). The series number refers to the simplistic relationships between the degree of regulation of the respective genes and the cellular state (untreated or CMP). Series 1 (both upregulated) - *BDNF-4h-CMP vs. Control-CMP* > *BDNF-4h-Control vs. Control-Control*; Series 2 (both upregulated) - *BDNF-4h-Control vs. Control-Control* > *BDNF-4h-CMP vs. Control-CMP*; Series 3 (both downregulated) - *BDNF-4h-Control vs. Control-Control* > *BDNF-4h-CMP vs. Control-CMP*; Series 4 (both downregulated) - *BDNF-4h-CMP vs. Control-CMP* > *BDNF-4h-Control vs. Control-Control*; Series 5 (downregulated in control, upregulated in CMP); Series 6 (upregulated in control, down regulated in CMP).

| **Gene Symbol** | **BDNF-4h-CMP vs. Control-CMP** | **BDNF-4h-Control vs. Control-Control** | **Series #** |
| --- | --- | --- | --- |
| LOC653994 | 5.185015977 | 4.317946887 | **1** |
| SUV420H1 | 4.825999114 | 4.67715949 | **1** |
| KIAA0644 | 4.769520658 | 3.965212545 | **1** |
| TNFRSF19 | 4.727342742 | 4.453958209 | **1** |
| LANCL2 | 4.125293613 | 3.285723649 | **1** |
| PLXNB1 | 3.973059146 | 3.327530217 | **1** |
| AMY1C | 3.936290582 | 2.201528043 | **1** |
| SLC11A2 | 3.694826107 | 3.491942969 | **1** |
| RNF150 | 3.592802505 | 2.67793168 | **1** |
| TNC | 3.53141719 | 1.747939199 | **1** |
| MGEA5 | 3.508915498 | 3.238887097 | **1** |
| BTBD11 | 3.440439183 | 2.524720592 | **1** |
| P2RY11 | 3.389680266 | 2.471241119 | **1** |
| RHBDD2 | 3.376798058 | 2.575609705 | **1** |
| TNPO1 | 3.316994959 | 3.28438645 | **1** |
| TACC2 | 3.297294376 | 1.865343354 | **1** |
| IL18BP | 3.239236246 | 1.845074312 | **1** |
| LOC285074 | 3.238199473 | 2.739420206 | **1** |
| CD44 | 3.238129251 | 2.343744276 | **1** |
| BTAF1 | 3.195969707 | 2.60539098 | **1** |
| NPEPL1 | 3.166136262 | 2.607074395 | **1** |
| SP2 | 3.142673526 | 2.065350945 | **1** |
| RBM33 | 3.109798878 | 2.13410318 | **1** |
| FHL2 | 3.040021779 | 1.957273609 | **1** |
| CLK1 | 3.02338074 | 2.192553661 | **1** |
| ZNF275 | 3.006194729 | 2.245011043 | **1** |
| HS6ST2 | 2.913360478 | 1.997856135 | **1** |
| C9orf156 | 2.896228757 | 1.959205471 | **1** |
| VCL | 2.87137715 | 2.65753103 | **1** |
| LRP5L | 2.868153013 | 2.309899426 | **1** |
| ARID5B | 2.832377577 | 2.070055557 | **1** |
| SEMA6A | 2.802053851 | 1.950290655 | **1** |
| ASCC3L1 | 2.801578617 | 2.446948758 | **1** |
| CAMSAP1 | 2.757150974 | 2.650879363 | **1** |
| FBXO31 | 2.753699234 | 1.985764442 | **1** |
| HMGB3 | 2.726851314 | 1.601401117 | **1** |
| TRIM33 | 2.713344739 | 2.112761455 | **1** |
| ITGB5 | 2.664591851 | 2.601627681 | **1** |
| CBX2 | 2.641830847 | 2.191810527 | **1** |
| SECISBP2 | 2.636027325 | 1.80148285 | **1** |
| ZNF536 | 2.634507111 | 1.561245094 | **1** |
| PERLD1 | 2.630016671 | 2.249158268 | **1** |
| SHPK | 2.58490427 | 1.609372776 | **1** |
| ATP9A | 2.567479971 | 1.593445049 | **1** |
| MYH9 | 2.556303842 | 1.891811662 | **1** |
| LOC653103 | 2.528395103 | 1.929259726 | **1** |
| HNRNPC | 2.513396639 | 2.116640313 | **1** |
| SUV420H1 | 2.50766754 | 2.368741077 | **1** |
| NKTR | 2.481672368 | 1.64746977 | **1** |
| UBE2J1 | 2.461663419 | 1.692116817 | **1** |
| TLE4 | 2.450381502 | 1.745873439 | **1** |
| KLF6 | 2.449743013 | 1.59934703 | **1** |
| MTMR3 | 2.445120768 | 2.283716077 | **1** |
| ING3 | 2.407754325 | 2.023925658 | **1** |
| RERG | 2.389964261 | 2.212785849 | **1** |
| RNF4 | 2.387012555 | 2.091706352 | **1** |
| PIAS2 | 2.379214157 | 1.902077973 | **1** |
| PPP1R15A | 2.370872254 | 1.589124157 | **1** |
| C14orf102 | 2.367598609 | 2.007695688 | **1** |
| RIMS3 | 2.350257988 | 2.054707473 | **1** |
| ERRFI1 | 2.324481891 | 2.192963074 | **1** |
| KCTD10 | 2.316491395 | 1.554147076 | **1** |
| TGIF1 | 2.312324044 | 1.73426117 | **1** |
| ADAMTSL2 | 2.30581769 | 1.622862348 | **1** |
| NASP | 2.304556995 | 2.059400903 | **1** |
| LOC728014 | 2.302661912 | 1.638408871 | **1** |
| SPRED2 | 2.293447022 | 1.857344139 | **1** |
| LOC727935 | 2.279098606 | 2.008537884 | **1** |
| MSI2 | 2.274069052 | 1.753945801 | **1** |
| ZNF789 | 2.230184034 | 2.045272216 | **1** |
| FLJ39827 | 2.219714259 | 1.518755772 | **1** |
| TNS3 | 2.218717603 | 1.931879411 | **1** |
| DHX40 | 2.214415502 | 2.056248121 | **1** |
| SH3BGRL2 | 2.193283488 | 1.890694116 | **1** |
| BNIP2 | 2.189617907 | 1.901193872 | **1** |
| SYT11 | 2.188956602 | 2.027945269 | **1** |
| ORAOV1 | 2.188308498 | 1.56808692 | **1** |
| C8orf33 | 2.179346297 | 1.647708024 | **1** |
| ATP1B1 | 2.172655246 | 1.98298443 | **1** |
| SLC7A1 | 2.13643697 | 2.054039523 | **1** |
| PPHLN1 | 2.128926357 | 1.718883533 | **1** |
| FOXJ3 | 2.110037462 | 1.781911819 | **1** |
| RNF103 | 2.097057042 | 1.503972007 | **1** |
| LOC441408 | 2.089813333 | 1.879897368 | **1** |
| BAZ2B | 2.087783334 | 2.046963352 | **1** |
| UBE2G1 | 2.068679841 | 1.942177675 | **1** |
| C6orf134 | 2.05443728 | 2.018815515 | **1** |
| TMEM18 | 2.043088711 | 1.959297118 | **1** |
| PFKFB3 | 2.042951193 | 1.689973732 | **1** |
| ABL1 | 2.036933298 | 1.830137609 | **1** |
| OPA1 | 2.0214748 | 1.978932448 | **1** |
| SLC30A7 | 1.997857754 | 1.589276222 | **1** |
| PRKRIP1 | 1.954852309 | 1.622399019 | **1** |
| ZNF317 | 1.952544697 | 1.634249429 | **1** |
| TRIM33 | 1.949596669 | 1.791666838 | **1** |
| ABCC5 | 1.942308841 | 1.622145986 | **1** |
| ZNF26 | 1.917894556 | 1.745126196 | **1** |
| GFRA3 | 1.906402192 | 1.768734428 | **1** |
| PSME4 | 1.904120795 | 1.823133206 | **1** |
| YOD1 | 1.889422134 | 1.752590117 | **1** |
| DBH | 1.880184072 | 1.697130266 | **1** |
| LOC146517 | 1.872970511 | 1.592213415 | **1** |
| PHF17 | 1.87235201 | 1.567476317 | **1** |
| BAT3 | 1.814436871 | 1.557633721 | **1** |
| MAP2 | 1.812507484 | 1.780272276 | **1** |
| MEF2D | 1.764509336 | 1.529718127 | **1** |
| BRPF1 | 1.711399956 | 1.609888058 | **1** |
| DECR2 | 1.709819982 | 1.51032235 | **1** |
| PAPD4 | 1.662724276 | 1.63045831 | **1** |
| THOC5 | 1.653650088 | 1.598297417 | **1** |
| RRAGB | 1.610316691 | 1.51092874 | **1** |
| E4F1 | 1.609670378 | 1.512175378 | **1** |
| IL8 | 6.698729633 | 7.139263759 | **2** |
| H3F3B | 4.84966617 | 5.416383235 | **2** |
| CSTF3 | 4.678439799 | 4.721537949 | **2** |
| RAD23B | 2.893976641 | 3.509298513 | **2** |
| GAB2 | 3.029086128 | 3.063350813 | **2** |
| FAT | 2.047575871 | 3.020520604 | **2** |
| TTC32 | 1.774836775 | 2.904482104 | **2** |
| SNAPC4 | 2.714156896 | 2.831141326 | **2** |
| INTS1 | 2.644483452 | 2.829325205 | **2** |
| CRY1 | 2.516843178 | 2.761189822 | **2** |
| FHL2 | 2.378644964 | 2.73253201 | **2** |
| ZNF212 | 2.586972242 | 2.717617185 | **2** |
| PNN | 2.035999706 | 2.662748437 | **2** |
| SFRS6 | 2.091906168 | 2.572525159 | **2** |
| LARP2 | 1.776997393 | 2.527654904 | **2** |
| RHBDD2 | 2.499844036 | 2.501165426 | **2** |
| SYNCRIP | 2.227711999 | 2.46419201 | **2** |
| C4orf30 | 1.611763567 | 2.451015266 | **2** |
| ZNF451 | 2.330143035 | 2.38917809 | **2** |
| ZYX | 2.272403259 | 2.3059487 | **2** |
| AHCTF1 | 1.572076181 | 2.232306712 | **2** |
| KLHL24 | 1.864520067 | 2.194556732 | **2** |
| NELL1 | 1.619808672 | 2.160030052 | **2** |
| TRMT11 | 1.83973144 | 2.122719035 | **2** |
| CSTF3 | 1.898982294 | 2.108854527 | **2** |
| ETV6 | 1.530105417 | 2.079479852 | **2** |
| RFC3 | 1.810179318 | 2.062242857 | **2** |
| LOC400027 | 1.974446978 | 2.025744669 | **2** |
| CNKSR3 | 1.601380039 | 1.973212193 | **2** |
| HCFC1 | 1.714227322 | 1.950279784 | **2** |
| JMJD1C | 1.870187754 | 1.880248462 | **2** |
| MNT | 1.579222399 | 1.844741406 | **2** |
| HBP1 | 1.577316979 | 1.766542131 | **2** |
| NOL6 | 1.6573768 | 1.756935354 | **2** |
| TOX | 1.661425092 | 1.725026829 | **2** |
| TUB | 1.611998344 | 1.683964073 | **2** |
| LOC650369 | 1.661077622 | 1.66640918 | **2** |
| DDX3X | 1.601223845 | 1.607921648 | **2** |
| ZNF35 | 1.564276136 | 1.587072293 | **2** |
| HSPA1A | -7.214643601 | -6.073929442 | **3** |
| NBPF20 | -5.745383058 | -3.533957253 | **3** |
| RAD21 | -5.442394006 | -2.096167951 | **3** |
| EZH2 | -5.279946113 | -3.015563001 | **3** |
| MSL3L1 | -5.242002716 | -1.912411344 | **3** |
| RN7SK | -5.21750905 | -3.084269346 | **3** |
| NBPF10 | -4.998306944 | -2.527325122 | **3** |
| KCTD12 | -4.908637725 | -2.754267143 | **3** |
| LOC440160 | -4.578381979 | -2.063351907 | **3** |
| GMCL1 | -4.493189175 | -2.230056092 | **3** |
| ID2 | -4.407292743 | -2.913282122 | **3** |
| GMCL1 | -4.386588363 | -2.779985273 | **3** |
| PHF14 | -4.253328402 | -2.603318254 | **3** |
| SCN2A | -4.170009072 | -3.836581694 | **3** |
| FLJ20397 | -3.895916799 | -2.303638683 | **3** |
| DDR2 | -3.741686328 | -1.920433497 | **3** |
| GAS2L3 | -3.704880213 | -2.681513051 | **3** |
| LOC402560 | -3.539315694 | -2.471262869 | **3** |
| RBM39 | -3.487887735 | -1.857816926 | **3** |
| LOC400879 | -3.410328195 | -2.27890504 | **3** |
| TERF1 | -3.376596536 | -1.860725651 | **3** |
| HIST1H4C | -3.348410391 | -2.17176518 | **3** |
| GRB2 | -3.286875196 | -1.757887822 | **3** |
| FAM120A | -3.282937349 | -1.589375492 | **3** |
| TRK1 | -3.262873631 | -1.913438843 | **3** |
| C19orf12 | -3.261175201 | -1.723662196 | **3** |
| LOC642852 | -3.214377247 | -2.9708255 | **3** |
| ENTPD4 | -3.134141335 | -1.733902391 | **3** |
| LOC642477 | -3.055002985 | -2.106626515 | **3** |
| TMEM5 | -2.967711781 | -2.939379806 | **3** |
| HIST1H2BD | -2.961983178 | -2.859893035 | **3** |
| OIP5 | -2.959114516 | -2.014196584 | **3** |
| UBFD1 | -2.890012422 | -2.543681592 | **3** |
| MTUS1 | -2.869172417 | -2.645770678 | **3** |
| RABEPK | -2.830319729 | -2.275436913 | **3** |
| NSL1 | -2.787924608 | -2.03649031 | **3** |
| C12orf11 | -2.762616511 | -2.3142434 | **3** |
| FLRT3 | -2.762560992 | -1.997442958 | **3** |
| C16orf53 | -2.757055571 | -1.646133185 | **3** |
| IVD | -2.751083308 | -2.311839149 | **3** |
| TSPAN5 | -2.750745563 | -1.68319303 | **3** |
| PLEKHB2 | -2.714245817 | -1.979979421 | **3** |
| LSAMP | -2.669591141 | -1.534622157 | **3** |
| REXO2 | -2.665113508 | -2.136819146 | **3** |
| NGLY1 | -2.660277698 | -2.217752839 | **3** |
| ATCAY | -2.640030611 | -2.017723092 | **3** |
| RILPL1 | -2.605497368 | -2.079076748 | **3** |
| HRSP12 | -2.559966853 | -2.457422471 | **3** |
| CHCHD3 | -2.545213823 | -1.971973801 | **3** |
| RBMS3 | -2.535230347 | -1.852581141 | **3** |
| DYNLL1 | -2.512694076 | -1.971423801 | **3** |
| TOB1 | -2.509511126 | -1.649456328 | **3** |
| PLEKHB2 | -2.462924663 | -2.183279515 | **3** |
| AMD1 | -2.437304713 | -1.788445961 | **3** |
| ELOVL6 | -2.424325445 | -2.341206907 | **3** |
| RPAP2 | -2.360613131 | -2.135043488 | **3** |
| ZC3HAV1 | -2.358787505 | -1.928759614 | **3** |
| MUTED | -2.350711526 | -1.582590763 | **3** |
| THOC3 | -2.313759672 | -1.711008223 | **3** |
| BAMBI | -2.268349745 | -1.829344497 | **3** |
| PCYOX1 | -2.26804733 | -1.511038171 | **3** |
| SC4MOL | -2.239252391 | -2.096582808 | **3** |
| CDKN1C | -2.222274643 | -1.671654034 | **3** |
| USO1 | -2.190587349 | -1.883823984 | **3** |
| UBQLN1 | -2.177477333 | -1.97236266 | **3** |
| ZCCHC7 | -2.169749594 | -1.963326269 | **3** |
| RHOT1 | -2.165194323 | -2.151590632 | **3** |
| TM7SF3 | -2.144368851 | -1.966251049 | **3** |
| INSIG1 | -2.142126844 | -1.719148325 | **3** |
| CSNK1G1 | -2.118939411 | -1.532039854 | **3** |
| HEATR2 | -2.092227711 | -1.9622642 | **3** |
| CRYZ | -2.078619253 | -1.934136194 | **3** |
| ZNF22 | -1.99081581 | -1.918626834 | **3** |
| DUT | -1.956325779 | -1.687853594 | **3** |
| C9orf23 | -1.939591394 | -1.845281423 | **3** |
| RNGTT | -1.92805889 | -1.887380747 | **3** |
| UBQLN1 | -1.906315332 | -1.883528204 | **3** |
| CDCA1 | -1.901301847 | -1.697462436 | **3** |
| UQCC | -1.893882609 | -1.681598755 | **3** |
| RTCD1 | -1.882153173 | -1.609693243 | **3** |
| GPR161 | -1.870688484 | -1.674065205 | **3** |
| RRAGD | -1.868713332 | -1.617855978 | **3** |
| LOC642197 | -1.809469749 | -1.695966755 | **3** |
| IGFBP5 | -1.793127537 | -1.782507683 | **3** |
| REXO4 | -1.772987029 | -1.6303306 | **3** |
| RPL7L1 | -1.745412738 | -1.541275749 | **3** |
| DPY30 | -1.733134574 | -1.52496241 | **3** |
| UCHL5 | -1.709771381 | -1.52336716 | **3** |
| UBE2G1 | -1.667788113 | -1.666718676 | **3** |
| DBT | -1.607988408 | -1.512482404 | **3** |
| MRPL52 | -1.569635454 | -1.522053369 | **3** |
| IDH1 | -1.522266694 | -1.522177871 | **3** |
| HSPA1B | -5.175009342 | -6.635112568 | **4** |
| ID2 | -4.558214893 | -4.758271105 | **4** |
| ID3 | -3.080224354 | -4.560916944 | **4** |
| RABL4 | -2.828150114 | -4.179547061 | **4** |
| ASCL1 | -4.107811144 | -4.117677711 | **4** |
| NRSN1 | -3.351516013 | -4.115651715 | **4** |
| PROSC | -2.394586285 | -3.819015178 | **4** |
| BANP | -2.757830822 | -3.807910663 | **4** |
| MORC2 | -2.433636787 | -3.742135667 | **4** |
| HERC4 | -2.480952451 | -3.568042345 | **4** |
| HIST1H2AC | -3.001551044 | -3.515958624 | **4** |
| HSPA8 | -3.222809855 | -3.422768642 | **4** |
| GART | -2.4697501 | -3.375749464 | **4** |
| LOC642282 | -1.812682948 | -3.321972593 | **4** |
| C3orf31 | -2.56948417 | -3.300543769 | **4** |
| SLC35B3 | -2.54657241 | -3.271499667 | **4** |
| CCDC102A | -2.559682466 | -3.240707756 | **4** |
| OPN3 | -2.39327229 | -3.190224804 | **4** |
| OPRL1 | -2.232704558 | -3.188258916 | **4** |
| PDPK1 | -2.068702394 | -3.171366008 | **4** |
| SMARCAL1 | -2.461330209 | -3.15214454 | **4** |
| C1orf86 | -1.663299556 | -3.116124308 | **4** |
| HIST1H2BD | -1.96094237 | -3.076439706 | **4** |
| GUCY1A3 | -2.879620028 | -3.065160726 | **4** |
| HSPA8 | -2.441336691 | -3.037775647 | **4** |
| MGC16169 | -1.911508585 | -3.03670108 | **4** |
| TAF12 | -2.028939791 | -3.01511503 | **4** |
| TPST2 | -2.233324225 | -2.993184377 | **4** |
| GPR162 | -1.573732438 | -2.985795344 | **4** |
| PHF14 | -1.940202426 | -2.983401814 | **4** |
| PIGF | -1.968999679 | -2.982547652 | **4** |
| DNAJA1 | -2.706449973 | -2.920025509 | **4** |
| DPAGT1 | -1.595753909 | -2.870982326 | **4** |
| RGS19 | -1.897885549 | -2.868883823 | **4** |
| BANP | -2.56375405 | -2.850044901 | **4** |
| THEM2 | -2.489605533 | -2.789618701 | **4** |
| GTF2E2 | -2.145485889 | -2.759198533 | **4** |
| C11orf60 | -2.509703872 | -2.625386502 | **4** |
| C16orf53 | -1.608730818 | -2.618972622 | **4** |
| PLCXD1 | -1.820368131 | -2.616439036 | **4** |
| SNCA | -1.507047194 | -2.596834115 | **4** |
| BRI3BP | -2.404631669 | -2.568700538 | **4** |
| NOLA1 | -2.219960803 | -2.54367368 | **4** |
| ATM | -2.369122417 | -2.537685689 | **4** |
| ZMYM6 | -2.220794743 | -2.512230061 | **4** |
| GMPPB | -1.914057652 | -2.500149839 | **4** |
| SLC9A3R1 | -1.981374626 | -2.496956021 | **4** |
| RPUSD3 | -1.842394838 | -2.487049127 | **4** |
| ZAK | -1.525347506 | -2.465629344 | **4** |
| NEFL | -2.438220675 | -2.45409875 | **4** |
| MRPL52 | -1.976336756 | -2.443116519 | **4** |
| RAD51C | -1.868238759 | -2.437217304 | **4** |
| PPAPDC3 | -1.626326368 | -2.419202549 | **4** |
| TEAD4 | -1.582216385 | -2.404543667 | **4** |
| TRIOBP | -2.202899191 | -2.39542037 | **4** |
| LRSAM1 | -1.732926265 | -2.389485202 | **4** |
| C16orf14 | -1.582843963 | -2.386681291 | **4** |
| NUDT9 | -1.655007286 | -2.370754823 | **4** |
| WDR67 | -2.053479757 | -2.332825593 | **4** |
| CENPE | -1.640489102 | -2.329735711 | **4** |
| TNFRSF25 | -1.555951013 | -2.316750488 | **4** |
| ABHD8 | -2.04979431 | -2.316638201 | **4** |
| PRMT2 | -1.966815421 | -2.307592521 | **4** |
| ADARB1 | -1.657501651 | -2.301624391 | **4** |
| XPO1 | -2.006772815 | -2.296758045 | **4** |
| MRPS33 | -2.097324173 | -2.285568021 | **4** |
| MRPS6 | -1.862480145 | -2.281957865 | **4** |
| SFRS7 | -1.752490948 | -2.266117379 | **4** |
| C12orf24 | -1.871951282 | -2.239252911 | **4** |
| C21orf51 | -1.915077861 | -2.22794769 | **4** |
| C15orf52 | -1.714516757 | -2.216606641 | **4** |
| KLRG1 | -1.724479792 | -2.204572091 | **4** |
| MED30 | -1.602761933 | -2.202937719 | **4** |
| DNCL1 | -1.811246889 | -2.197966987 | **4** |
| RAD51C | -2.059357446 | -2.189970331 | **4** |
| GALNT11 | -1.702238288 | -2.171668096 | **4** |
| WDR92 | -1.764558818 | -2.164048695 | **4** |
| SCAND1 | -1.698268461 | -2.162668053 | **4** |
| CSTF3 | -1.777084378 | -2.134085556 | **4** |
| TMEM126B | -1.71015781 | -2.126027261 | **4** |
| FAM48A | -1.521729394 | -2.117720619 | **4** |
| PREB | -1.975013351 | -2.098587911 | **4** |
| GMPPA | -1.780117178 | -2.084968441 | **4** |
| SIX5 | -1.643095914 | -2.07603359 | **4** |
| FBXO22 | -1.664905276 | -2.074111033 | **4** |
| STK3 | -1.897714606 | -2.064603216 | **4** |
| HSPH1 | -2.047197982 | -2.0556083 | **4** |
| RAB24 | -1.92233272 | -2.05427374 | **4** |
| THAP10 | -1.568509427 | -2.048389711 | **4** |
| CMBL | -1.678218305 | -2.028427381 | **4** |
| SDCCAG10 | -1.586364979 | -2.018765743 | **4** |
| LOC149448 | -1.963250441 | -2.01728044 | **4** |
| CYB5A | -1.657481922 | -2.003469753 | **4** |
| RHOT1 | -1.654014341 | -2.000217264 | **4** |
| MAPK3 | -1.533485533 | -1.963212653 | **4** |
| EXOSC3 | -1.706241417 | -1.94440102 | **4** |
| BBX | -1.736532026 | -1.937172894 | **4** |
| COASY | -1.918893073 | -1.934908327 | **4** |
| SPOP | -1.721869668 | -1.925414177 | **4** |
| NTHL1 | -1.77380078 | -1.924310727 | **4** |
| LOC219854 | -1.703544967 | -1.917551667 | **4** |
| STK4 | -1.589392168 | -1.894475428 | **4** |
| AHSA1 | -1.734703938 | -1.887720518 | **4** |
| SRBD1 | -1.82951759 | -1.870406597 | **4** |
| SMEK2 | -1.644417199 | -1.861481626 | **4** |
| NARS2 | -1.601597225 | -1.844738053 | **4** |
| MKKS | -1.750312178 | -1.836216847 | **4** |
| LDLR | -1.792916555 | -1.833742217 | **4** |
| CAP2 | -1.703056197 | -1.832584514 | **4** |
| LOC375295 | -1.538462795 | -1.830870174 | **4** |
| LSM2 | -1.544151934 | -1.808864459 | **4** |
| BCL2L12 | -1.686235086 | -1.806922796 | **4** |
| SEC31A | -1.729325644 | -1.796372189 | **4** |
| C11orf54 | -1.672461142 | -1.765308874 | **4** |
| MAP2K1 | -1.558695758 | -1.687331627 | **4** |
| C1orf144 | -1.568867929 | -1.680599367 | **4** |
| SAR1B | -1.597905926 | -1.61959709 | **4** |
| HIBCH | -1.562159061 | -1.605660493 | **4** |
| PTMA | -4.200971811 | 4.772243326 | **6** |
| LOC643287 | -3.760487725 | 2.08489721 | **6** |
| SUMO2 | -3.224343901 | 2.883939123 | **6** |
| LOC730746 | -3.156337114 | 3.908645223 | **6** |
| DDX17 | -3.065131005 | 1.847242901 | **6** |
| RNASEH2B | -2.996507193 | 2.244315841 | **6** |
| TIA1 | -2.856680107 | 1.556013866 | **6** |
| RPLP1 | -2.718651646 | 5.380460392 | **6** |
| MCTS1 | -2.684690044 | 1.808802133 | **6** |
| TSC22D1 | -2.659042193 | 3.507003705 | **6** |
| FTHL12 | -2.646734298 | 2.861393649 | **6** |
| METTL7A | -2.623804153 | 2.082030541 | **6** |
| FTHL8 | -2.613864708 | 2.968372736 | **6** |
| LOC441377 | -2.607662563 | 2.641320733 | **6** |
| HMG1L1 | -2.601657922 | 3.442773134 | **6** |
| LOC220433 | -2.535213175 | 3.509761937 | **6** |
| FTHL12 | -2.476930712 | 2.512552856 | **6** |
| LOC389787 | -2.462764154 | 2.67403021 | **6** |
| PAPSS1 | -2.445714372 | 2.755098231 | **6** |
| LOC643007 | -2.398995864 | 3.964952728 | **6** |
| NACAP1 | -2.374416424 | 2.672928641 | **6** |
| RPL9 | -2.360755324 | 3.953360598 | **6** |
| LOC641848 | -2.305889772 | 5.538194602 | **6** |
| FTHL11 | -2.299866805 | 2.557715027 | **6** |
| WSB2 | -2.291947539 | 2.288530511 | **6** |
| LOC389672 | -2.281298639 | 3.659625023 | **6** |
| DC2 | -2.271517719 | 2.485324671 | **6** |
| LOC653773 | -2.23726168 | 2.451786013 | **6** |
| HMG1L1 | -2.201831364 | 1.999877209 | **6** |
| YY1 | -2.19858571 | 1.578888657 | **6** |
| LOC651202 | -2.197808702 | 3.2881942 | **6** |
| LOC649946 | -2.179838014 | 3.966780944 | **6** |
| RPL23 | -2.125948313 | 2.576776469 | **6** |
| LOC648343 | -2.082220013 | 3.646256552 | **6** |
| LOC653658 | -2.073147174 | 2.026668044 | **6** |
| TCP1 | -2.020726556 | 1.817304841 | **6** |
| RAD21 | -1.974200906 | 2.746908951 | **6** |
| LOC728973 | -1.953992704 | 2.87103608 | **6** |
| CCBE1 | -1.875475154 | 2.381074184 | **6** |
| LOC731640 | -1.834870398 | 2.676493702 | **6** |
| LOC646900 | -1.817557895 | 1.820016081 | **6** |
| RPL7 | -1.778156021 | 2.590657038 | **6** |
| FTHL2 | -1.743575132 | 2.857236855 | **6** |
| IFP38 | -1.738976699 | 2.719028404 | **6** |
| LOC388621 | -1.738731491 | 2.95604781 | **6** |
| HMGB2 | -1.725466966 | 2.88635239 | **6** |
| HNRPC | -1.725418539 | 1.704976324 | **6** |
| RG9MTD1 | -1.705461171 | 1.68350053 | **6** |
| ANXA2P1 | -1.700803362 | 2.477454114 | **6** |
| LOC347376 | -1.667987049 | 3.123884649 | **6** |
| LOC641849 | -1.650571622 | 2.460869388 | **6** |
| LEP | -1.637931086 | 2.414258737 | **6** |
| TUBA3D | -1.631215174 | 3.182121401 | **6** |
| FTHL3 | -1.610399358 | 3.575145728 | **6** |
| DTWD2 | -1.595826661 | 2.752560217 | **6** |
| RPS28 | -1.5908175 | 2.192968171 | **6** |
| USP49 | -1.589811692 | 2.618301871 | **6** |
| LOC644250 | -1.587080527 | 2.90352751 | **6** |
| LOC653232 | -1.563733786 | 2.838020652 | **6** |
| LOC642502 | -1.527448181 | 1.816934799 | **6** |
| FRG1 | -1.513767906 | 1.584358495 | **6** |
| RPL14 | -1.513439263 | 3.20664144 | **6** |
