# Supplementary material for: Minimal Peroxide Exposure of Neuronal Cells Induces Multifaceted Adaptive Responses
Source: PLoS One. 2010 Dec 17;5(12):e14352. doi: 10.1371/journal.pone.0014352 (PMC3003681; doi:10.1371/journal.pone.0014352)
Supplement: Table S20 — Common 8 hour BDNF-regulated gene series. BDNF-regulated genes that were significantly elevated or reduced compared to the respective unstimulated control cells in both the control (untreated: BDNF-8h-Control vs. Control-Control) and CMP state SH-SY5Y cells (BDNF-8h-CMP vs. Control-CMP). The series number refers to the simplistic relationships between the degree of regulation of the respective genes and the cellular state (untreated or CMP). Series 1 (both upregulated) - BDNF-8h-CMP vs. Control-CMP > BDNF-8h-Control vs. Control-Control; Series 2 (both upregulated) - BDNF-8h-Control vs. Control-Control > BDNF-8h-CMP vs. Control-CMP; Series 3 (both downregulated) - BDNF-8h-Control vs. Control-Control > BDNF-8h-CMP vs. Control-CMP; Series 4 (both downregulated) - BDNF-8h-CMP vs. Control-CMP > BDNF-8h-Control vs. Control-Control; Series 5 (downregulated in control, upregulated in CMP); Series 6 (upregulated in control, down regulated in CMP). (0.46 MB DOC) [file pone.0014352.s027.doc]

**Table S20. Common 8 hour BDNF-regulated gene series.** BDNF-regulated genes that were significantly elevated or reduced compared to the respective unstimulated control cells in both the control (untreated: *BDNF-8h-Control vs. Control-Control*) and CMP state SH-SY5Y cells (*BDNF-8h-CMP vs. Control-CMP*). The series number refers to the simplistic relationships between the degree of regulation of the respective genes and the cellular state (untreated or CMP). Series 1 (both upregulated) - *BDNF-8h-CMP vs. Control-CMP* > *BDNF-8h-Control vs. Control-Control*; Series 2 (both upregulated) - *BDNF-8h-Control vs. Control-Control* > *BDNF-8h-CMP vs. Control-CMP*; Series 3 (both downregulated) - *BDNF-8h-Control vs. Control-Control* > *BDNF-8h-CMP vs. Control-CMP*; Series 4 (both downregulated) - *BDNF-8h-CMP vs. Control-CMP* > *BDNF-8h-Control vs. Control-Control*; Series 5 (downregulated in control, upregulated in CMP); Series 6 (upregulated in control, down regulated in CMP).

| **SYMBOL** | **BDNF-8h-CMP vs. Control-CMP** | **BDNF-8h-Control vs. Control-Control** | **Series #** |
| --- | --- | --- | --- |
| CD44 | 4.536392577 | 3.893344883 | **1** |
| HS6ST2 | 4.454144607 | 4.037498281 | **1** |
| AMY1C | 4.003692192 | 3.706523479 | **1** |
| CSTF3 | 3.969423782 | 2.856272207 | **1** |
| SPOCK1 | 3.854115856 | 2.990363678 | **1** |
| NPEPL1 | 3.788056702 | 3.056747261 | **1** |
| GFRA3 | 3.777435426 | 3.203399788 | **1** |
| RHBDD2 | 3.773534322 | 3.60332561 | **1** |
| STK36 | 3.688218106 | 2.280542563 | **1** |
| RHBDD2 | 3.527519644 | 2.925142745 | **1** |
| BRWD1 | 3.462021544 | 2.63484342 | **1** |
| TNC | 3.452968596 | 2.19286815 | **1** |
| VCAM1 | 3.41956509 | 2.012642176 | **1** |
| USP24 | 3.416703908 | 2.001043861 | **1** |
| BTBD11 | 3.350457857 | 1.991057873 | **1** |
| NDRG4 | 3.347847856 | 2.227817096 | **1** |
| BTAF1 | 3.344767401 | 2.879401731 | **1** |
| CLK1 | 3.289012185 | 2.500710883 | **1** |
| PCM1 | 3.10392584 | 2.470181305 | **1** |
| HCN3 | 3.098382632 | 2.083424695 | **1** |
| FOXJ2 | 3.029553721 | 2.171945932 | **1** |
| BSN | 2.967953297 | 2.546990434 | **1** |
| PRPF4B | 2.953109102 | 2.598377615 | **1** |
| GATS | 2.939089178 | 1.579363731 | **1** |
| FAM119B | 2.896294354 | 2.024072983 | **1** |
| ATP9A | 2.883088657 | 2.543974762 | **1** |
| FNBP4 | 2.873842721 | 2.023666179 | **1** |
| HEATR5B | 2.862952665 | 2.287627797 | **1** |
| ARFGEF1 | 2.815010319 | 2.38056004 | **1** |
| TTC13 | 2.805823067 | 2.767819924 | **1** |
| CAPRIN1 | 2.774346893 | 2.421486707 | **1** |
| PLXNA3 | 2.691851924 | 2.099454853 | **1** |
| C4orf41 | 2.690615282 | 2.121890675 | **1** |
| AGPAT4 | 2.68223838 | 2.596670011 | **1** |
| C8orf33 | 2.666004638 | 1.636614287 | **1** |
| AZIN1 | 2.624167311 | 1.613926897 | **1** |
| VCL | 2.608282706 | 2.118353501 | **1** |
| SIN3A | 2.572761595 | 1.777710579 | **1** |
| NKTR | 2.572302182 | 1.700976656 | **1** |
| SH3BGRL2 | 2.571149533 | 1.538923379 | **1** |
| ANKRD13D | 2.522970505 | 1.73574332 | **1** |
| RABEP1 | 2.521854076 | 2.505189007 | **1** |
| TRMT11 | 2.515668936 | 2.392550755 | **1** |
| KIAA0194 | 2.485930054 | 2.116878687 | **1** |
| DIP2B | 2.474057985 | 1.773770852 | **1** |
| C6orf134 | 2.457868431 | 1.92131189 | **1** |
| CTDSPL | 2.433253021 | 1.579495986 | **1** |
| KIAA1737 | 2.426341902 | 1.74425388 | **1** |
| WDR19 | 2.405614757 | 1.68307711 | **1** |
| ZNF615 | 2.40428037 | 1.786716178 | **1** |
| DHX40 | 2.403903221 | 2.210814899 | **1** |
| LOC339344 | 2.387515924 | 1.666198251 | **1** |
| SORT1 | 2.355929396 | 1.677315815 | **1** |
| CAMSAP1L1 | 2.348992685 | 2.122450892 | **1** |
| ST8SIA2 | 2.340446609 | 1.605529121 | **1** |
| SC5DL | 2.322831057 | 1.582510666 | **1** |
| DGKQ | 2.275107091 | 1.985868185 | **1** |
| XRN1 | 2.264727913 | 2.030488496 | **1** |
| GLMN | 2.256549601 | 1.546588392 | **1** |
| C15orf17 | 2.230151996 | 1.533737772 | **1** |
| MYH9 | 2.22511446 | 2.210840519 | **1** |
| MON2 | 2.218527083 | 1.88937457 | **1** |
| GNS | 2.214336822 | 1.656720611 | **1** |
| DPYSL3 | 2.208752333 | 1.839139467 | **1** |
| THOC5 | 2.203480339 | 1.665910764 | **1** |
| DFFB | 2.201256787 | 1.724233109 | **1** |
| PSME4 | 2.195202531 | 2.035098937 | **1** |
| MRPS25 | 2.187848018 | 1.561370633 | **1** |
| HEATR5B | 2.177388342 | 1.646352613 | **1** |
| NCOA6 | 2.157716336 | 1.874331742 | **1** |
| KIAA1671 | 2.145566525 | 2.040408504 | **1** |
| RIOK2 | 2.140912921 | 1.983111122 | **1** |
| CHRNA3 | 2.139490377 | 1.627611671 | **1** |
| CNOT1 | 2.138639855 | 1.93032987 | **1** |
| DIP2B | 2.094157503 | 1.967080226 | **1** |
| DBH | 2.084658326 | 1.733701257 | **1** |
| NPC1 | 2.07976253 | 1.801662695 | **1** |
| DVL3 | 2.071266752 | 1.804177826 | **1** |
| RIMS3 | 2.069746747 | 1.67065086 | **1** |
| WDR35 | 2.05033605 | 1.653004728 | **1** |
| ZNF451 | 2.007590823 | 1.966750057 | **1** |
| DCBLD2 | 2.0051398 | 1.629176459 | **1** |
| TAF1C | 1.994249881 | 1.6246115 | **1** |
| LASP1 | 1.989326572 | 1.683416654 | **1** |
| NSUN5 | 1.985684818 | 1.850785251 | **1** |
| UBR5 | 1.984774068 | 1.594337667 | **1** |
| ASXL2 | 1.984337503 | 1.947215811 | **1** |
| GART | 1.972712851 | 1.595469044 | **1** |
| FHOD1 | 1.971456605 | 1.8148646 | **1** |
| FAM62B | 1.968783384 | 1.861891228 | **1** |
| KIAA1545 | 1.946801627 | 1.843772702 | **1** |
| FAM131A | 1.908377925 | 1.797489226 | **1** |
| DNAJB1 | 1.907243319 | 1.696579072 | **1** |
| EXTL3 | 1.902279423 | 1.876785032 | **1** |
| ZNF275 | 1.886484498 | 1.691735685 | **1** |
| CAMSAP1 | 1.884740487 | 1.701416558 | **1** |
| LOC653994 | 1.883162768 | 1.713282518 | **1** |
| TFRC | 1.869774149 | 1.825990225 | **1** |
| MAPK8IP3 | 1.866150551 | 1.833667888 | **1** |
| ATP2C1 | 1.859942789 | 1.777115393 | **1** |
| FAM125B | 1.830983122 | 1.706991584 | **1** |
| CLIP2 | 1.81954322 | 1.683888864 | **1** |
| LOC647346 | 1.810162183 | 1.682966285 | **1** |
| SEMA4B | 1.800250766 | 1.603169156 | **1** |
| UBE3C | 1.778901854 | 1.640834544 | **1** |
| PNMA2 | 1.763988328 | 1.577661363 | **1** |
| ATP6V0A2 | 1.686724268 | 1.656522328 | **1** |
| PHF13 | 1.680991336 | 1.534654759 | **1** |
| IL8 | 7.825073827 | 8.228386347 | **2** |
| NELL1 | 3.731738549 | 4.484201589 | **2** |
| C1orf63 | 3.8022539 | 3.999484735 | **2** |
| PLXNB1 | 2.855683273 | 3.297798539 | **2** |
| SGSH | 3.170363604 | 3.272460284 | **2** |
| PRKCA | 1.828828677 | 3.119765646 | **2** |
| OCIAD2 | 2.248460857 | 2.853054357 | **2** |
| SLC41A1 | 1.734685689 | 2.827583818 | **2** |
| GON4L | 1.751523941 | 2.815492519 | **2** |
| ACCN2 | 2.1258432 | 2.653743154 | **2** |
| NRCAM | 1.709753298 | 2.553079753 | **2** |
| TUB | 1.604357826 | 2.551496152 | **2** |
| MAST3 | 1.719328311 | 2.551277934 | **2** |
| ZNF514 | 1.95065014 | 2.550815521 | **2** |
| ATP1B1 | 1.745486764 | 2.506070783 | **2** |
| ANKS1A | 2.310689606 | 2.458929775 | **2** |
| PAPD4 | 2.310080823 | 2.357408334 | **2** |
| LOC728734 | 2.17096497 | 2.332468631 | **2** |
| PDGFRB | 2.197017602 | 2.304007361 | **2** |
| TNK2 | 2.284359602 | 2.29237592 | **2** |
| MCM3AP | 1.874723815 | 2.273609215 | **2** |
| GOLGA8B | 2.095977419 | 2.259764135 | **2** |
| SUV420H1 | 1.704424169 | 2.24881531 | **2** |
| ACSL4 | 1.885473909 | 2.242852153 | **2** |
| GAB2 | 1.625579344 | 2.236552658 | **2** |
| ZNF26 | 1.81157373 | 2.144294914 | **2** |
| TRIM46 | 2.113867732 | 2.122841169 | **2** |
| SNAPC4 | 2.015753922 | 2.114134441 | **2** |
| ZMYM4 | 1.799167992 | 2.110951827 | **2** |
| 3-Sep | 1.922489441 | 2.093846659 | **2** |
| KIAA0649 | 1.584883954 | 2.085523033 | **2** |
| SPAST | 1.841922083 | 2.070790233 | **2** |
| ASCC3L1 | 1.760153554 | 2.060006011 | **2** |
| SF3B1 | 1.860945248 | 2.059249533 | **2** |
| KIAA1688 | 1.879881218 | 2.04389327 | **2** |
| SELI | 1.70131833 | 2.017049703 | **2** |
| KCTD3 | 1.506965818 | 1.999247422 | **2** |
| JUP | 1.757735935 | 1.975919283 | **2** |
| NOC3L | 1.535039038 | 1.926953384 | **2** |
| OGFR | 1.7222668 | 1.911168282 | **2** |
| LOC729264 | 1.651079365 | 1.907591017 | **2** |
| FLJ10081 | 1.579578669 | 1.894424369 | **2** |
| KLHL7 | 1.631800119 | 1.893706192 | **2** |
| PRPF3 | 1.665731998 | 1.878322483 | **2** |
| STK40 | 1.733936756 | 1.828838436 | **2** |
| TOMM20 | 1.553460752 | 1.764408114 | **2** |
| VPS39 | 1.721140381 | 1.763943416 | **2** |
| MAP4K4 | 1.682471507 | 1.707580987 | **2** |
| ARNT2 | 1.577267141 | 1.692847926 | **2** |
| IDS | 1.526297821 | 1.620493974 | **2** |
| D4S234E | 1.533324487 | 1.590800854 | **2** |
| KCTD12 | -6.508390185 | -3.402546876 | **3** |
| ID2 | -6.046852352 | -5.115615994 | **3** |
| SNRPD3 | -5.765334234 | -3.204674571 | **3** |
| SPIN1 | -5.35111538 | -2.086113892 | **3** |
| RN7SK | -5.287589531 | -2.742574923 | **3** |
| RAD21 | -5.26754377 | -3.220992973 | **3** |
| GOLPH4 | -5.181735348 | -1.703429539 | **3** |
| PROX1 | -4.930629409 | -1.915873455 | **3** |
| GSK3B | -4.683353598 | -2.775460439 | **3** |
| DDR2 | -4.501536837 | -2.134557969 | **3** |
| C8orf53 | -4.487713153 | -1.852994725 | **3** |
| THOC2 | -4.377146639 | -1.725776189 | **3** |
| MSL3L1 | -4.373323838 | -2.506434614 | **3** |
| HIST1H4C | -4.239432424 | -2.77449547 | **3** |
| KIF15 | -4.094875109 | -2.323916806 | **3** |
| GAS2L3 | -4.070750631 | -2.831256832 | **3** |
| LOC162073 | -4.05712348 | -2.465884585 | **3** |
| ARID4B | -3.954303166 | -2.135425782 | **3** |
| EZH2 | -3.953573824 | -2.228305145 | **3** |
| LOC729137 | -3.801187492 | -1.686049666 | **3** |
| TERF1 | -3.770253839 | -2.190580412 | **3** |
| FLJ20397 | -3.76304722 | -3.478668666 | **3** |
| LOC642033 | -3.733538636 | -2.63993857 | **3** |
| LOC644162 | -3.715951502 | -1.82968582 | **3** |
| GMCL1 | -3.628753807 | -1.645681227 | **3** |
| GTPBP8 | -3.579096974 | -1.78596324 | **3** |
| USP1 | -3.49270911 | -1.501387602 | **3** |
| NDUFA10 | -3.49110621 | -1.900501625 | **3** |
| IGFBP3 | -3.434846648 | -3.06083548 | **3** |
| LOC400879 | -3.333799868 | -2.22225974 | **3** |
| HEATR2 | -3.25840813 | -2.730349597 | **3** |
| NBPF20 | -3.238682031 | -2.457046016 | **3** |
| NOLA1 | -3.237502199 | -2.847777789 | **3** |
| LOC402560 | -3.147369581 | -2.26969841 | **3** |
| BAMBI | -3.106434828 | -2.194415583 | **3** |
| GMCL1 | -3.036804975 | -1.753196072 | **3** |
| RORB | -3.033162662 | -2.773576538 | **3** |
| PRR11 | -3.009831287 | -2.20387214 | **3** |
| TSPAN5 | -2.998992354 | -1.775929108 | **3** |
| CDC2L2 | -2.983560128 | -2.818076363 | **3** |
| NCAPD2 | -2.936344138 | -2.202749687 | **3** |
| SOX4 | -2.935672651 | -1.607696459 | **3** |
| LOC642477 | -2.911390208 | -1.967008651 | **3** |
| MTUS1 | -2.905011413 | -2.765284731 | **3** |
| OSBPL1A | -2.904742007 | -1.541994349 | **3** |
| TRK1 | -2.895247635 | -2.747769342 | **3** |
| ZBTB43 | -2.856878954 | -1.582037005 | **3** |
| CFDP1 | -2.807106458 | -2.212071905 | **3** |
| C20orf94 | -2.720574188 | -1.686747939 | **3** |
| C16orf14 | -2.716000451 | -2.308587857 | **3** |
| GTPBP8 | -2.682958675 | -1.61363232 | **3** |
| JARID1A | -2.621582834 | -2.215512206 | **3** |
| NEFL | -2.608600352 | -2.223735134 | **3** |
| LOC643995 | -2.606797624 | -2.275716285 | **3** |
| RABL4 | -2.58148963 | -2.571757248 | **3** |
| AIG1 | -2.580724471 | -2.148210002 | **3** |
| NUDT5 | -2.576665815 | -2.253174101 | **3** |
| CTSL2 | -2.570442303 | -1.730983267 | **3** |
| TDG | -2.563608625 | -2.275414733 | **3** |
| CDKN3 | -2.560788238 | -2.328714804 | **3** |
| CARS | -2.557094301 | -2.442633682 | **3** |
| LOC642852 | -2.543551925 | -2.491879969 | **3** |
| CCDC137 | -2.542602979 | -2.20424002 | **3** |
| MRPL24 | -2.452329603 | -1.764296684 | **3** |
| NFIC | -2.421161529 | -2.203801686 | **3** |
| MRPL33 | -2.408981795 | -1.929503345 | **3** |
| AMD1 | -2.384122397 | -1.801518189 | **3** |
| THOC3 | -2.383849087 | -1.794074813 | **3** |
| PIGP | -2.362152417 | -1.977384455 | **3** |
| MANBAL | -2.358153942 | -1.668472575 | **3** |
| LIMS1 | -2.316321346 | -1.644244024 | **3** |
| LRRCC1 | -2.31587828 | -1.679186411 | **3** |
| NR2F1 | -2.310550374 | -1.892117314 | **3** |
| OPA3 | -2.304404757 | -1.565061804 | **3** |
| APEH | -2.300192411 | -1.894157875 | **3** |
| H1F0 | -2.287522706 | -2.286772201 | **3** |
| POLA1 | -2.281085236 | -2.179626199 | **3** |
| CDCA1 | -2.245892989 | -1.887692955 | **3** |
| LOC653884 | -2.198180768 | -1.970839565 | **3** |
| EPHX2 | -2.191068792 | -2.016331102 | **3** |
| UBL5 | -2.17752059 | -2.001051676 | **3** |
| APOO | -2.163918469 | -1.616718802 | **3** |
| RBMS1 | -2.151983007 | -1.68923131 | **3** |
| FKBP2 | -2.133424452 | -1.643879565 | **3** |
| TCTEX1D2 | -2.095624425 | -1.970366872 | **3** |
| TOB1 | -2.067887926 | -1.881073383 | **3** |
| CKLF | -2.065610773 | -1.682393845 | **3** |
| POPDC2 | -2.060487038 | -1.812689166 | **3** |
| HSPB1 | -2.053392455 | -1.932795933 | **3** |
| NAV2 | -2.03648538 | -1.523589885 | **3** |
| CHAF1B | -1.984599864 | -1.511511169 | **3** |
| COG4 | -1.984217255 | -1.516558665 | **3** |
| FSD1 | -1.972970328 | -1.644417853 | **3** |
| CCDC90A | -1.964989607 | -1.772411257 | **3** |
| TSPAN7 | -1.927253097 | -1.821326222 | **3** |
| PDPK1 | -1.924429011 | -1.801382373 | **3** |
| PRICKLE1 | -1.905965731 | -1.601212507 | **3** |
| PEX7 | -1.832992432 | -1.722396235 | **3** |
| DYNLRB1 | -1.81464511 | -1.58198897 | **3** |
| RSRC1 | -1.813701083 | -1.547365641 | **3** |
| ALDH6A1 | -1.806034671 | -1.657138115 | **3** |
| HSPE1 | -1.725370089 | -1.657568117 | **3** |
| KDELR2 | -1.712390972 | -1.602639291 | **3** |
| TSGA14 | -1.614473774 | -1.526875637 | **3** |
| MNT | -1.592680569 | -1.579403229 | **3** |
| PCDH17 | -1.574272016 | -1.545182464 | **3** |
| NPFFR2 | -1.566856121 | -1.557628339 | **3** |
| MRPL11 | -1.550793091 | -1.52865171 | **3** |
| ID2 | -6.244998041 | -7.234205457 | **4** |
| IGFBP5 | -4.893558265 | -5.271697281 | **4** |
| ID3 | -3.680967755 | -5.170067182 | **4** |
| LOC375295 | -3.381462208 | -4.223246641 | **4** |
| IGFBP5 | -2.876714099 | -3.939886784 | **4** |
| DCN | -2.154688887 | -3.78202603 | **4** |
| OIP5 | -2.196529382 | -3.743417842 | **4** |
| CAP2 | -2.165769199 | -3.701532944 | **4** |
| C2orf34 | -2.271255669 | -3.543241515 | **4** |
| C1orf86 | -1.8207776 | -3.537373666 | **4** |
| GMDS | -1.917834567 | -3.528905683 | **4** |
| DUT | -3.170542829 | -3.520392488 | **4** |
| HES4 | -2.365796705 | -3.299662023 | **4** |
| COX17 | -2.560463188 | -3.29339189 | **4** |
| CHMP4A | -2.927507174 | -3.244984516 | **4** |
| DPAGT1 | -1.628129824 | -3.197630255 | **4** |
| UBFD1 | -3.096304211 | -3.122016743 | **4** |
| GNG8 | -2.099650515 | -3.096570116 | **4** |
| LOC727761 | -1.60710004 | -3.086706667 | **4** |
| ICT1 | -2.560379808 | -3.044809546 | **4** |
| LOC728635 | -2.129848443 | -3.041158641 | **4** |
| LOC387882 | -1.815481883 | -3.034172073 | **4** |
| KCNMA1 | -2.427971177 | -2.926400697 | **4** |
| AKR7A2 | -1.881911513 | -2.915878008 | **4** |
| LOC644422 | -2.396357881 | -2.91155551 | **4** |
| ADM | -2.21903064 | -2.87945251 | **4** |
| NSMCE1 | -1.875037909 | -2.862034958 | **4** |
| SMYD3 | -2.184071497 | -2.770251129 | **4** |
| ASCL1 | -2.283011171 | -2.751506783 | **4** |
| HBQ1 | -1.547923984 | -2.691852215 | **4** |
| SCN2A | -2.430951307 | -2.684645175 | **4** |
| FKBP4 | -1.897674872 | -2.669563157 | **4** |
| FUZ | -2.359591195 | -2.646262958 | **4** |
| CYB5A | -1.557929979 | -2.639357589 | **4** |
| ING3 | -1.780008919 | -2.632180035 | **4** |
| LAGE3 | -2.083794044 | -2.569795853 | **4** |
| CRELD1 | -1.543915975 | -2.561547326 | **4** |
| USMG5 | -2.020966541 | -2.552124829 | **4** |
| LRRC17 | -2.128697584 | -2.533453881 | **4** |
| PIR | -2.000990129 | -2.508417212 | **4** |
| CKLF | -1.818541782 | -2.481788099 | **4** |
| C1orf144 | -2.441544732 | -2.473322915 | **4** |
| GMEB1 | -1.681485137 | -2.433704587 | **4** |
| CMBL | -2.377528916 | -2.429459995 | **4** |
| FUSIP1 | -1.604922104 | -2.401538785 | **4** |
| ENDOG | -1.858832403 | -2.398259734 | **4** |
| CYB5A | -1.87344343 | -2.375562563 | **4** |
| C6orf125 | -2.292512037 | -2.367826493 | **4** |
| LSAMP | -1.962672329 | -2.306447085 | **4** |
| FEZ1 | -2.172729694 | -2.300033728 | **4** |
| NUDT1 | -2.146657728 | -2.286276179 | **4** |
| RPL7L1 | -1.830837458 | -2.28329635 | **4** |
| GAS6 | -1.552435403 | -2.255878356 | **4** |
| ZP3 | -1.517408295 | -2.240798796 | **4** |
| SFRS14 | -1.865608196 | -2.240161769 | **4** |
| GFRA2 | -2.176413297 | -2.232608766 | **4** |
| ZNF511 | -1.707059937 | -2.221086684 | **4** |
| CRABP2 | -1.696711821 | -2.219061621 | **4** |
| DNAJB6 | -1.647299681 | -2.212806224 | **4** |
| YWHAB | -1.775340314 | -2.210721026 | **4** |
| PMVK | -1.844576766 | -2.207055287 | **4** |
| CRABP1 | -1.552859841 | -2.115033356 | **4** |
| NFKBIA | -1.753691042 | -2.108802815 | **4** |
| PRR3 | -1.897802748 | -2.103451856 | **4** |
| PTPLA | -1.51969985 | -2.101046864 | **4** |
| ANAPC11 | -1.779030481 | -2.099877437 | **4** |
| ZNF22 | -1.876224259 | -2.083955056 | **4** |
| GUCY1A3 | -2.006803228 | -2.079598453 | **4** |
| BRP44L | -1.955017461 | -2.065374175 | **4** |
| SIL1 | -1.533868794 | -2.054451962 | **4** |
| MRPL40 | -1.708086017 | -2.048944751 | **4** |
| GPSN2 | -1.696978426 | -2.029531318 | **4** |
| PIR | -1.989560878 | -2.02720369 | **4** |
| MYLIP | -1.63707081 | -2.017609221 | **4** |
| BANF1 | -1.780007386 | -1.990784254 | **4** |
| UBE2C | -1.584742614 | -1.986373286 | **4** |
| RPL39L | -1.765627953 | -1.933124522 | **4** |
| REXO2 | -1.690269626 | -1.900469687 | **4** |
| ATPAF1 | -1.807717623 | -1.893743272 | **4** |
| CABYR | -1.581245632 | -1.888169009 | **4** |
| GEM | -1.647233292 | -1.868099762 | **4** |
| PPM1G | -1.712273045 | -1.809837276 | **4** |
| FLRT3 | -1.731365759 | -1.793324023 | **4** |
| BIRC5 | -1.598084345 | -1.788155378 | **4** |
| C20orf24 | -1.690249505 | -1.714610552 | **4** |
| HSP90AA1 | -1.673375618 | -1.709232082 | **4** |
| C1orf163 | -1.655641218 | -1.708293099 | **4** |
| BCL2L12 | -1.635002278 | -1.636232807 | **4** |
| GAS6 | -1.534215625 | -1.62302965 | **4** |
| LOC255130 | -1.524349167 | -1.559812546 | **4** |
| PTMA | -6.052918901 | 4.191177203 | **6** |
| HMG1L1 | -3.633865187 | 2.244505076 | **6** |
| NACAP1 | -3.336685685 | 1.858793425 | **6** |
| LOC441377 | -3.26769905 | 2.834103714 | **6** |
| SUMO2 | -3.227665817 | 2.499576187 | **6** |
| TUBA3D | -3.171518738 | 2.391045086 | **6** |
| LOC643007 | -3.081024396 | 3.693235079 | **6** |
| LOC402644 | -3.024708873 | 2.852941483 | **6** |
| FTHL8 | -2.991290978 | 2.779897359 | **6** |
| TUBB4Q | -2.926308163 | 1.72446909 | **6** |
| WSB2 | -2.851912607 | 1.845675292 | **6** |
| FTHL12 | -2.80768404 | 2.579819658 | **6** |
| LOC651202 | -2.806119415 | 3.04859858 | **6** |
| TOP1P2 | -2.751984927 | 1.917273475 | **6** |
| LSM5 | -2.750395028 | 2.66457338 | **6** |
| LOC220433 | -2.74497897 | 2.941196412 | **6** |
| LOC730746 | -2.680137925 | 4.576198946 | **6** |
| FTHL11 | -2.634880938 | 2.57386554 | **6** |
| LOC649946 | -2.631203135 | 4.043108109 | **6** |
| FTHL2 | -2.625808831 | 2.017029182 | **6** |
| RPL7 | -2.60290101 | 2.285162913 | **6** |
| LOC728973 | -2.548649128 | 2.821676384 | **6** |
| RG9MTD1 | -2.543571776 | 1.510389529 | **6** |
| RPL23 | -2.419332042 | 2.975161015 | **6** |
| PAPSS1 | -2.404607915 | 2.404909194 | **6** |
| FTHL3 | -2.397161142 | 2.439760904 | **6** |
| HIF1A | -2.378252716 | 2.948489074 | **6** |
| LOC649679 | -2.37785608 | 1.915440726 | **6** |
| TSC22D1 | -2.34601113 | 2.508356297 | **6** |
| LOC389672 | -2.336875831 | 3.596508372 | **6** |
| LOC389787 | -2.317881123 | 3.037786968 | **6** |
| DDX17 | -2.303652558 | 2.177235044 | **6** |
| LOC641848 | -2.283534928 | 4.977933919 | **6** |
| RPL9 | -2.260921546 | 3.81986329 | **6** |
| CCBE1 | -2.181120654 | 2.299237955 | **6** |
| LOC643997 | -2.136637228 | 3.506595116 | **6** |
| RAD21 | -2.119407333 | 2.219378779 | **6** |
| TIA1 | -2.097794341 | 1.771787321 | **6** |
| MGC40489 | -2.067482968 | 3.436729644 | **6** |
| RPLP1 | -2.037883194 | 4.738197537 | **6** |
| RTN3 | -1.997708739 | 1.535618018 | **6** |
| LOC647436 | -1.980435202 | 4.118363864 | **6** |
| LOC731640 | -1.97205853 | 2.573111445 | **6** |
| LOC653773 | -1.944432494 | 2.760341685 | **6** |
| LOC641849 | -1.929964292 | 1.996574664 | **6** |
| LOC347376 | -1.86752479 | 1.818203435 | **6** |
| LOC648343 | -1.848907577 | 3.4766104 | **6** |
| LOC388621 | -1.843728686 | 2.667514697 | **6** |
| LOC651894 | -1.83900107 | 2.61301437 | **6** |
| RPL14 | -1.800380928 | 1.855339473 | **6** |
| RPS28 | -1.771686242 | 2.250654613 | **6** |
| LOC644250 | -1.731971957 | 2.114082944 | **6** |
| LOC653658 | -1.71603221 | 2.067718584 | **6** |
| LOC388532 | -1.713823263 | 2.022864043 | **6** |
| ANXA2P1 | -1.693130457 | 2.050144982 | **6** |
| LOC653232 | -1.650139266 | 2.327441873 | **6** |
| P704P | -1.62609259 | 4.278708299 | **6** |
| ACTR3 | -1.613322905 | 2.873212371 | **6** |
| DLD | -1.593107497 | 1.601135534 | **6** |
| RPS27 | -1.574259175 | 1.894076025 | **6** |
| LOC402251 | -1.571503649 | 2.213622745 | **6** |
| KIAA1751 | -1.532412813 | 3.046063326 | **6** |
| HMGB2 | -1.515545821 | 1.59658254 | **6** |
