# Supplementary material for: Minimal Peroxide Exposure of Neuronal Cells Induces Multifaceted Adaptive Responses
Source: PLoS One. 2010 Dec 17;5(12):e14352. doi: 10.1371/journal.pone.0014352 (PMC3003681; doi:10.1371/journal.pone.0014352)
Supplement: Table S21 — Regulated proteins on Panorama cell signaling antibody array following CMP treatment. The expression ratio between that in CMP versus control (CTL) conditions was measured from multiple (n = 4) array experiments and is expressed as a mean ratio with a calculated standard error of that meam (SEM). (0.10 MB DOC) [file pone.0014352.s028.doc]

**Table S21. Regulated proteins on Panorama® cell signaling antibody array following CMP treatment.** The expression ratio between that in CMP versus control (CTL) conditions was measured from multiple (n=4) array experiments and is expressed as a mean ratio with a calculated standard error of that meam (SEM).

| **Symbol** | **Protein** | **CMP vs. CTL ratio** | **SEM** |
| --- | --- | --- | --- |
| H3F3A | Acetyl & phospho Histone H3 | 0.086809773 | 0.043955486 |
| AP1B1 | Adaptin b1+b2 | 1.833391552 | 0.057743723 |
| APP | Amyloid Precursor Protein (APP) | 0.557121153 | 0.034657563 |
| APP | Amyloid Precursor Protein (APP) C-terminal | 0.363551737 | 0.005799546 |
| AIFM1 | Apoptosis Inducing Factor (AIF) | 2.909811673 | 0.053777837 |
| PSCD2 | ARNO (Cytohesin 2) | 1.535371952 | 0.008283662 |
| TUBA4A | b Tubulin polyglutamylated | 0.014402025 | 0.018034954 |
| BCL10 | Bcl-10 | 0.448412759 | 0.060415681 |
| BCL2L1 | Bcl-x | 0.317269363 | 0.034170028 |
| BCL2L1 | Bcl-XL | 0.269638403 | 0.009469811 |
| ABL1 | c-Abl oncogene 1, receptor tyrosine kinase | 2.286223412 | 0.007794841 |
| CALM1 | Calmodulin | 0.72900675 | 0.005295876 |
| CANX | Calnexin | 1.462158783 | 0.019551743 |
| CNN1 | Calponin | 1.38351882 | 0.020571472 |
| CALR | Calreticulin | 0.680791 | 0.007216732 |
| CAMK4 | CAM Kinase IV (AY-18) | 0.274120365 | 0.051400064 |
| CASP10 | Caspase 10 | 0.053720507 | 0.022307915 |
| CASP3 | Caspase 3 | 0.423885122 | 0.042805559 |
| CASP6 | Caspase 6 | 0.190482781 | 0.022144418 |
| CASP9 | Caspase 9 | 0.048313608 | 0.044794751 |
| CTSD | Cathepsin D | 0.430826819 | 0.061039749 |
| CDC25 | Cell division cycle 25 homolog C | 1.465697546 | 0.004999788 |
| CDC7 | CDC7 Kinase | 0.006505978 | 8.42362E-05 |
| CFL1 | Cofilin | 0.292287882 | 0.032520804 |
| GJB1 | Connexin 32 | 1.916527823 | 0.069881565 |
| CCNB1 | Cyclin B1 | 0.194936484 | 0.057266325 |
| CCND2 | Cyclin D2 | 0.05368953 | 0.058653485 |
| DES | Desmin | 1.264472879 | 0.01479081 |
| DMD | Dystrophin | 1.205731251 | 0.00148489 |
| EGFR | EGF receptor | 0.04880526 | 0.00941819 |
| ERK5 | ERK5 (BIG MAPK-BMK1) | 0.280767016 | 0.028821472 |
| ESR1 | Estrogen Receptor (ER) | 0.386305382 | 0.023633069 |
| RASA3 | RAS p21 protein activator 3 | 1.270185602 | 0.013737342 |
| GRIN2A | Glutamate receptor NMDAR 2a | 0.077840636 | 0.049926437 |
| NOS2A | Nitric oxide synthase 2, inducible | 1.844853006 | 0.049535904 |
| INA | Internexin α  | 0.022776466 | 0.030680253 |
| MAPK8 | JNK activated (diphosphorylated JNK) | 0.048786203 | 0.059846354 |
| KIF3A | Kinesin family member 3A | 0.041971983 | 0.013029662 |
| MAP1A | Microtubule-associated protein 1A | 0.028976005 | 0.02300759 |
| MAP2 | Microtubule-associated protein 2 | 1.71191869 | 0.015780125 |
| TRKA | Nerve Growth Factor Receptor | 0.239443725 | 0.048946904 |
| NUTF2 | Nuclear transport factor 2 | 1.827268116 | 0.009682798 |
| CTNND1 | Catenin (cadherin-associated protein), delta 1 | 1.392216132 | 0.008199366 |
| CDKN2A | Cyclin-dependent kinase inhibitor 2A | 0.258928347 | 0.016561865 |
| CDKN2D | Cyclin-dependent kinase inhibitor 2D | 0.048344492 | 0.052622187 |
| CDKN1C | Cyclin-dependent kinase inhibitor 1C | 3.569410826 | 0.025161828 |
| PAK1 | p21 protein (Cdc42/Rac)-activated kinase 1: phospho (pS212) | 0.032370293 | 0.028761269 |
| PAR4 | Prostate apoptosis response 4 | 0.461702701 | 0.022019343 |
| PLA2G5 | Phospholipase A2 group V | 0.261947094 | 0.016547139 |
| PLCG1 | Phospholipase C γ1 | 0.03869713 | 0.034718775 |
| Ser-Phos | Phosphoserine | 0.212851224 | 0.017561129 |
| AKT1 | v-akt murine thymoma viral oncogene homolog 1 | 0.065148902 | 0.005105934 |
| AKT1 | AKT1 phosphoserine 473 (pS473) | 0.014750923 | 0.017629493 |
| PRKCA | Protein kinase C α  | 0.390622955 | 0.05926712 |
| PRKCG | Protein kinase C γ | 0.054853348 | 0.065918742 |
| CASP8 | Procaspase 8 | 0.308753189 | 0.020059377 |
| PYK2 | Proline-rick tyrosine kinase 2: Phospho (pY881) | 0.124121241 | 0.00550445 |
| RAN | Ras-related nuclear protein | 0.104672305 | 0.027505711 |
| S100A1 | S-100 calcium-binding protein 1 | 0.052681992 | 0.035219878 |
| SMAC/DIABLO | Second mitochondria-derived activator of caspase | 2.96054132 | 0.024642363 |
| SNAP25 | Synaptosomal-associated protein, 25-Kda | 0.493738605 | 0.023128428 |
| STX1A | Syntaxin | 1.876552889 | 0.024750385 |
| SNCB | Synuclein β | 1.801752008 | 0.0268012 |
| TPM1 | Tropomyosin | 0.018450677 | 0.018591603 |
| TPH1 | Tryptophane Hydroxylase | 1.579126779 | 0.069946632 |
| VCL | Vinculin | 1.363501138 | 0.00642067 |
