# Supplementary material for: Minimal Peroxide Exposure of Neuronal Cells Induces Multifaceted Adaptive Responses
Source: PLoS One. 2010 Dec 17;5(12):e14352. doi: 10.1371/journal.pone.0014352 (PMC3003681; doi:10.1371/journal.pone.0014352)
Supplement: Table S22 — Network analysis of antibody signaling array CMP-significantly regulated proteins. Specific correlated networks were generated using an un-biased algorithm using Ingenuity Pathway Analysis (IPA). The highest network scores indicate the probabilistic likelihood of the specific protein network functionally dominating the dataset. Pre-determined functional networks are significantly populated with molecules (proteins) from the input experimental dataset. The network score is a function of the number and rarity of the specific experimental Focus molecules that populate the specific network. Predicted functions are derived from proprietary IPA software. (0.27 MB DOC) [file pone.0014352.s029.doc]

**Table S22. Network analysis of antibody signaling array CMP-significantly regulated proteins.** Specific correlated networks were generated using an un-biased algorithm using Ingenuity Pathway Analysis (IPA). The highest network scores indicate the probabilistic likelihood of the specific protein network functionally dominating the dataset. Pre-determined functional networks are significantly populated with molecules (proteins) from the input experimental dataset. The network score is a function of the number and rarity of the specific experimental Focus molecules that populate the specific network. Predicted functions are derived from proprietary IPA software.

| **Network Score** | **Focus Molecules** | **Molecules in Network**  **(Focus Bold)** | **Top Predicted Functions** |
| --- | --- | --- | --- |
|  |  |  |  |
| **27** | ***14*** | Alpha tubulin | *Nervous System Development and Function* |
|  |  | **APP** | *Cell Morphology* |
|  |  | **CALR** | *Cell-To-Cell Signaling and Interaction* |
|  |  | **CANX** |  |
|  |  | Ck2 |  |
|  |  | Collagen type I |  |
|  |  | **CTNND1** |  |
|  |  | **EGFR** |  |
|  |  | **ESR1** |  |
|  |  | **GJB1** |  |
|  |  | **H3F3A (includes EG:3020)** |  |
|  |  | hCG |  |
|  |  | Histone h3 |  |
|  |  | Histone h4 |  |
|  |  | Ifn gamma |  |
|  |  | IgG |  |
|  |  | IL12 (complex) |  |
|  |  | Importin alpha |  |
|  |  | Insulin |  |
|  |  | Integrin |  |
|  |  | Lh |  |
|  |  | **MAP1A** |  |
|  |  | Mapk |  |
|  |  | **MAPK8** |  |
|  |  | **NOS2** |  |
|  |  | **NUTF2** |  |
|  |  | P38 MAPK |  |
|  |  | Rac |  |
|  |  | **RAN** |  |
|  |  | RNA polymerase II |  |
|  |  | STAT5a/b |  |
|  |  | Tgf beta |  |
|  |  | **TUBA4A** |  |
|  |  | Vegf |  |
|  |  |  |  |
| **25** | ***14*** | **ACTA1** | *Cellular Assembly and Organization* |
|  |  | Actin | *Skeletal and Muscular System Development and Function* |
|  |  | Alpha actin | *Tissue Morphology* |
|  |  | Alpha catenin |  |
|  |  | Ap1 gamma |  |
|  |  | BCR |  |
|  |  | **CALM1** |  |
|  |  | CaMKII |  |
|  |  | CD3 |  |
|  |  | CD8 |  |
|  |  | **CDKN2D** |  |
|  |  | **CFL1** |  |
|  |  | **CNN1** |  |
|  |  | Cofilin |  |
|  |  | **DES** |  |
|  |  | **DMD** |  |
|  |  | Endothelin |  |
|  |  | F Actin |  |
|  |  | G-Actin |  |
|  |  | Ige |  |
|  |  | **MAP2** |  |
|  |  | N-type Calcium Channel |  |
|  |  | NCK |  |
|  |  | NFAT (complex) |  |
|  |  | NFkB (complex) |  |
|  |  | Pak, |  |
|  |  | **PAK1** |  |
|  |  | **PLCG1** |  |
|  |  | Rock |  |
|  |  | **S100A1** |  |
|  |  | **SNAP25** |  |
|  |  | **STX1A** |  |
|  |  | TCR |  |
|  |  | **TPM1** |  |
|  |  | VAV |  |
|  |  |  |  |
| **19** | ***11*** | **AIFM1** | *Cell Morphology* |
|  |  | Akt | *DNA:* |
|  |  | **AKT1** | *Replication* |
|  |  | Ap1 | *Recombination* |
|  |  | **BCL10** | *Repair* |
|  |  | c-Src | *Post-Translational Modification* |
|  |  | Calcineurin protein(s) |  |
|  |  | **CAMK4** |  |
|  |  | **CASP3** |  |
|  |  | **CASP6** |  |
|  |  | **CASP8** |  |
|  |  | **CASP9** |  |
|  |  | **CASP10** |  |
|  |  | CASP3/6/7 |  |
|  |  | Caspase 8/10 |  |
|  |  | **CTSD** |  |
|  |  | Cytochrome c |  |
|  |  | DNAJ |  |
|  |  | Estrogen Receptor |  |
|  |  | Growth hormone |  |
|  |  | HDL |  |
|  |  | Hsp27 |  |
|  |  | Hsp70 |  |
|  |  | Hsp90 |  |
|  |  | IFN Beta |  |
|  |  | IKK (complex) |  |
|  |  | Interferon alpha |  |
|  |  | Jnk |  |
|  |  | LDL |  |
|  |  | NMDA Receptor |  |
|  |  | peptidase |  |
|  |  | **SNCB** |  |
|  |  | SRC |  |
|  |  | Tnf receptor |  |
|  |  |  |  |
| **18** | ***10*** | 14-3-3 | *Cell Cycle* |
|  |  | **ABL1** | *Cancer* |
|  |  | **BCL2L1** | *Genetic Disorder* |
|  |  | Caspase |  |
|  |  | Caspase 3/7 |  |
|  |  | **CCNB1** |  |
|  |  | CCND2 |  |
|  |  | Cdc2 |  |
|  |  | **CDC7** |  |
|  |  | **CDC25C** |  |
|  |  | **CDKN1C** |  |
|  |  | **CDKN2A** |  |
|  |  | Creb |  |
|  |  | Cyclin A |  |
|  |  | Cyclin B |  |
|  |  | Cyclin D |  |
|  |  | Cyclin E |  |
|  |  | **CYTH2** |  |
|  |  | E2f |  |
|  |  | ERK1/2 |  |
|  |  | Laminin |  |
|  |  | MAP2K1/2 |  |
|  |  | Mek |  |
|  |  | NGF |  |
|  |  | p70 S6k |  |
|  |  | Pdgf |  |
|  |  | PDGF BB |  |
|  |  | PLA2 |  |
|  |  | **PLA2G5** |  |
|  |  | PP2A |  |
|  |  | Raf |  |
|  |  | Ras |  |
|  |  | Rb |  |
|  |  | Scf |  |
|  |  | Shc |  |
|  |  |  |  |
| **7** | ***5*** | **AP1B1** | *Lipid Metabolism* |
|  |  | B4GALNT1 | *Small Molecule Biochemistry* |
|  |  | BTNL2 | *Cellular Function and Maintenance* |
|  |  | CAPG |  |
|  |  | CCDC80 |  |
|  |  | CCL6 |  |
|  |  | CCRN4L |  |
|  |  | CD6 |  |
|  |  | CD276 |  |
|  |  | CD1B |  |
|  |  | CD1C |  |
|  |  | CREB1 |  |
|  |  | CST7 |  |
|  |  | CTSZ (includes EG:1522) |  |
|  |  | CXCL16 |  |
|  |  | DUSP14 |  |
|  |  | GBP1 (includes EG:14468) |  |
|  |  | HRAS |  |
|  |  | HRSP12 |  |
|  |  | IFI203 |  |
|  |  | IL4 |  |
|  |  | IL17B |  |
|  |  | IL17C |  |
|  |  | **INA** |  |
|  |  | KCNC1 |  |
|  |  | **KIF3A** |  |
|  |  | KIF3C |  |
|  |  | KLRB1C (includes EG:17059) |  |
|  |  | **RASA3** |  |
|  |  | SERPIND1 |  |
|  |  | TCRB-V8.3 |  |
|  |  | TNF |  |
|  |  | **TPH1** |  |
|  |  | TYMP |  |
|  |  | VIPR2 |  |
|  |  |  |  |
| **5** | ***4*** | 2-amino-3-phosphonopropionic acid | *Organismal Development* |
|  |  | 24,25-dihydroxyvitamin D3 | *Cardiovascular Disease* |
|  |  | 26s Proteasome | *Cellular Compromise* |
|  |  | ADD2 |  |
|  |  | Adducin |  |
|  |  | AKR1C14 |  |
|  |  | C8ORF4 |  |
|  |  | Calmodulin |  |
|  |  | Collagen(s) |  |
|  |  | CORO1B |  |
|  |  | ERK |  |
|  |  | Focal adhesion kinase |  |
|  |  | FSH |  |
|  |  | FZD3 |  |
|  |  | FZD6 |  |
|  |  | G protein beta gamma |  |
|  |  | Gat |  |
|  |  | Gpcr |  |
|  |  | **GRIN2A** |  |
|  |  | IL1 |  |
|  |  | KISS1R |  |
|  |  | PI3K |  |
|  |  | PITPNB |  |
|  |  | Pka |  |
|  |  | Pkc(s) |  |
|  |  | PLC |  |
|  |  | **PRKCA** |  |
|  |  | **PRKCG** |  |
|  |  | PRKD3 |  |
|  |  | Sapk |  |
|  |  | SLC20A2 |  |
|  |  | STAT |  |
|  |  | Trk Receptor |  |
|  |  | **VCL** |  |
